# Nonheme iron catalyst mimics heme-dependent haloperoxidase for efficient bromination and oxidation

**DOI:** 10.1126/sciadv.adq0028

**Published:** 2024-12-04

**Authors:** Guodong Zhao, Huiling Dong, Kang Xue, Shaoyan Lou, Rui Qi, Xiaohui Zhang, Zhuo Cao, Qi Qin, Bingqing Yi, Haimin Lei, Rongbiao Tong

**Affiliations:** ^1^ School of Chinese Materia Medica, Beijing University of Chinese Medicine, Beijing 102488, China.; ^2^ Engineering Research Center for Pharmaceutics of Chinese Materia Medica and New Drug Development, Ministry of Education, Beijing, 100029, China.; ^3^ Department of Neurology and Innovation center for neurological disorders, Xuanwu Hospital, Capital Medical University, Beijing, 100029, China.; ^4^ Department of Chemistry, The Hong Kong University of Science and Technology, Clearwater Bay, Kowloon, Hong Kong, China.

## Abstract

The [Fe]/H_2_O_2_ oxidation system has found wide applications in chemistry and biology. Halogenation with this [Fe]/H_2_O_2_ oxidation protocol and halide (X^−^) in the biological system is well established with the identification of heme-iron–dependent haloperoxidases. However, mimicking such halogenation process is rarely explored for practical use in organic synthesis. Here, we report the development of a nonheme iron catalyst that mimics the heme-iron–dependent haloperoxidases to catalyze the generation of HOBr from H_2_O_2_/Br^−^ with high efficiency. We discovered that a tridentate terpyridine (TPY) ligand designed for Fenton chemistry was optimal for FeBr_3_ to form a stable nonheme iron catalyst [Fe(TPY)Br_3_], which catalyzed arene bromination, Hunsdiecker-type decarboxylative bromination, bromolactonization, and oxidation of sulfides and thiols. Mechanistic studies revealed that Fenton chemistry ([Fe]/H_2_O_2_) might operate to generate hydroxyl radical (HO^•^), which oxidize bromide ion [Br^−^] into reactive HOBr. This nonheme iron catalyst represents a biomimetic model for heme-iron–dependent haloperoxidases with potential applications in organic synthesis, drug discovery, and biology.

## Teaser

Hijacking Fenton chemistry enables mimicking haloperoxidases for bromination and oxidation.

## INTRODUCTION

**Fig. 1.**
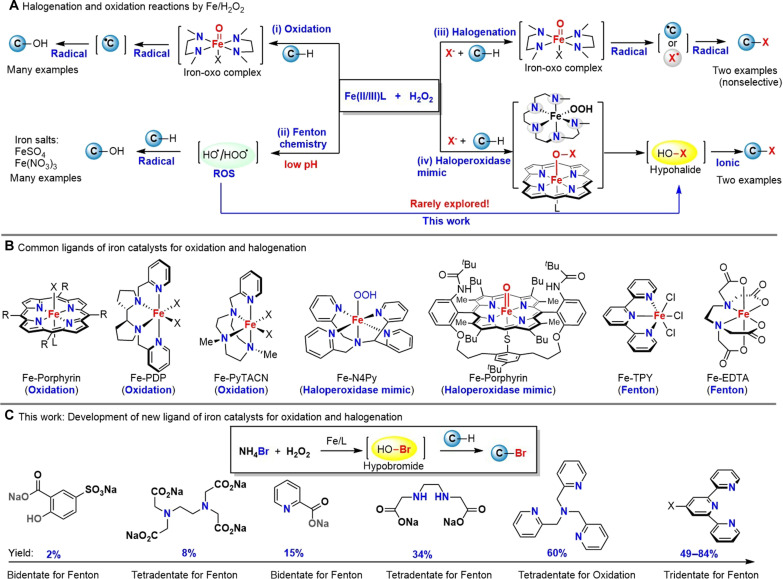
The [Fe]/H_2_O_2_ oxidation system in organic synthesis, representative ligands for iron, and this work. (**A**) Halogenation and oxidation reactions catalyzed by [Fe]/H_2_O_2_. (**B**) Common ligands of iron catalysts for oxidation and halogenation. (**C**) This work: Development of new ligand of iron catalysts for oxidation and halogenation.

The [Fe]/H_2_O_2_ oxidation system (Fig. 1A) is a powerful method for oxidation of organic compounds and has found wide applications in chemistry and biology (*1*–*8*). Heme-dependent peroxidases (*9*, *10*) (i.e., cytochrome c peroxidase) and haloperoxidases (*11*–*13*) [i.e., chloroperoxidase (CPO)] are representative enzymes of the [Fe]/H_2_O_2_ oxidation system in biology, which play an important role in various biological processes including biosynthesis and degradation of organic molecules. Organic chemists extensively exploit the high oxidation potential of the [Fe]/H_2_O_2_ system in many oxidation/halogenation processes (Fig. 1A) (*14*–*29*). For examples, the [Fe]/H_2_O_2_ system has been used for alkene epoxidation (*14*, *15*)/dihydroxylation (*16*–*18*), C─H oxidation (*19*–*22*), halogenation (*23*–*25*), sulfide/thiol oxidation (*26*), alcohol oxidation (*27*, *28*), indole oxidation (*29*), etc. Most [Fe]/H_2_O_2_ reactions proceed in a radical mechanism [Fig. 1A, (i) oxidation, (ii) Fenton chemistry, and (iii) halogenation] through in situ generation of a reactive high valent iron-oxo complex or hydroxyl/hydroperoxyl radical (Fenton chemistry) (*1*–*8*), which usually causes low chemo- and regioselectivity and/or hardly tolerates typical organic functional groups. Only two examples (*24*, *25*) of the [Fe]/H_2_O_2_ system were reported to produce hypohalide [i.e., hypochlorous acid (HOCl) and hypobromous acid (HOBr)] and halogenate arene C─H or enols through an ionic mechanism, which closely mimicked the heme-iron–dependent haloperoxidase for electrophilic halogenation [Fig. 1A, (iv) haloperoxidase mimic]. Unfortunately, these two biomimetic models are rarely used in related halogenation reactions. It was noted that the pentadentate iron complexes [Fe═O versus Fe─OOH] were synthesized by de Visser and colleagues (*24*) for mechanistic studies on ionic and radical pathways of chlorination using anisole as the sole substrate. The porphyrin-based iron catalysts by Wagenknecht and Woggon (*25*) were excellent biomimetic models of CPO, and five substrates consisting of 2-chloro-5,5-dimethyl-1,3-dimedone (MCD), 1,3-cyclopentanedione, anisole, toluene, and cyclohexanone were successfully halogenated with hydrogen peroxide under acidic condition (AcOH or BF_3_-Et_2_O). However, the nine-step synthesis of these porphyrin-based iron catalysts is nontrivial and lack flexibility, which might restrict their applications in organic synthesis. A practically useful iron catalyst for electrophilic halogenation with hydrogen peroxide and halide is highly desirable and becomes the aim of this work to bridge the gap between mechanistic study and synthetic utility.

It is well established that the reactivity and mechanistic pathway of the [Fe]/H_2_O_2_ system are primarily governed by the iron’s ligand (Fig. 1B). Inspired by nature’s heme-dependent oxygenases (*30*), peroxidases (*9*, *10*), and haloperoxidases (*11*–*13*), many nitrogen-based tetradentate ligands such as porphyrin (*4*), White-Chen catalyst (*19*, *20*) (Fe-PDP), and FePyTACN (*1*, *2*) are well designed and tuned for selective radical C─H oxidation and/or halogenation through in situ generation of high-valent Fe^IV/V^═O complexes (*31*, *32*). Nitrogen-based polydentate (*33*) (Fe-EDTA) and tridentate ligands (*34*, *35*) (Fe-TPY) as well as some inorganic ligands including sulfate, nitrate, and halides favored the generation of hydroxyl/hydroperoxyl radical via Fenton chemistry. However, the violent reactivity of hydroxy radical (*36*) (redox potential of 2.8 V is only second to fluorine 3.0 V) renders Fenton chemistry useful in degradation of organic compounds (environmental science) but ineffective for chemo-, regio-, and/or stereoselective oxidation of structurally complex organic molecules. The nitrogen-based pentadentate (*24*) (Fe-N4Py) and porphyrin-thiolate (*25*) were found to form iron complexes with distinct reactivity: catalyze the reaction of halide with H_2_O_2_ to generate electrophilic hypohalide. Here, we examined extensively different iron ligands (Fig. 1C) and discovered that the tridentate ligand TPY for Fenton chemistry was optimal for FeBr_3_ to form a stable nonheme iron complex that catalyzed the reaction of hydrogen peroxide with bromide under mild condition to generate the reactive hypobromous acid for arene bromination, Hunsdiecker-type decarboxylative bromination, bromolactonization of alkene-tethered carboxylic acids, and oxidation of sulfides and thiols. This new Fe(TYP)Br_3_/H_2_O_2_/NH_4_Br protocol addresses many limitations of our previous Fenton-bromide (FeBr_3_/H_2_O_2_) (*37*, *38*) and demonstrated superior substrate scope and efficiency than the prior two enzymatic models (*24*, *25*) of the [Fe]/H_2_O_2_ system.

## RESULTS

### Identification of terpyridine-iron complex as catalyst for efficient in situ generation of HOBr from H_2_O_2_ and NH_4_Br

**Fig. 2.**
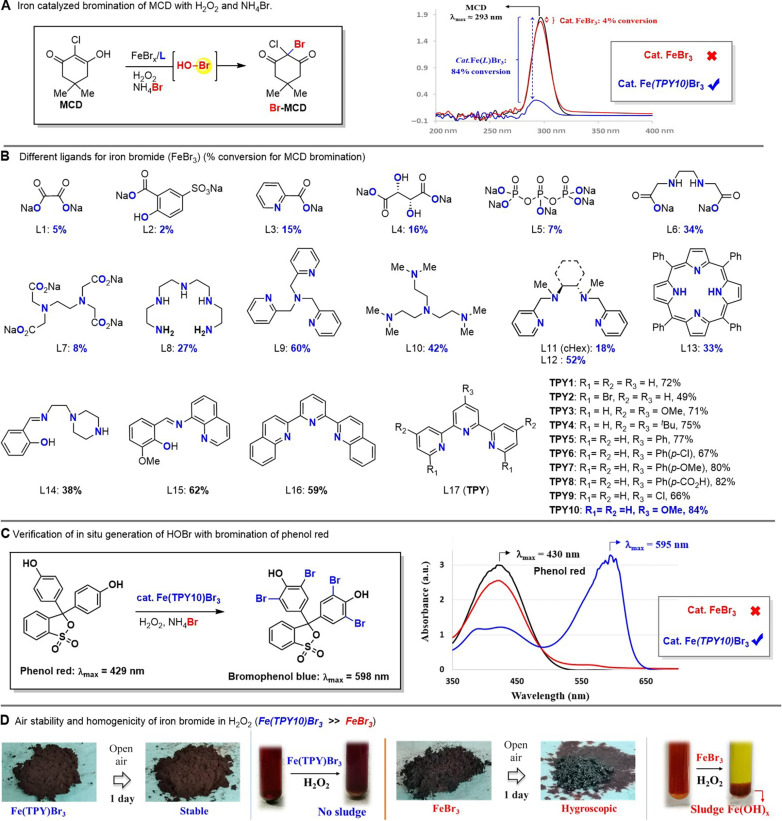
Identification of terpyridine-iron complex as catalyst for efficient in situ generation of HOBr from H_2_O_2_ and NH_4_Br. (**A**) Iron-catalyzed bromination of MCD with H_2_O_2_ and NH_4_Br. (**B**) Different ligands for FeBr_3_ and their catalytic conversion yields for MCD bromination. (**C**) Verification of in situ generation of HOBr with bromination of phenol red. a.u., arbitrary units. (**D**) Air stability and homogenicity of iron bromide in H_2_O_2_. Cat., catalysis.

We chose bromination of MCD into Br-MCD as our model reaction to evaluate the catalytic efficiency of various iron complexes for in situ generation of hypobromous acid (HOBr) from H_2_O_2_ and NH_4_Br because MCD (enol form) can effectively capture the electrophilic [Br^+^] (e.g., HOBr) and MCD bromination is a standard assay for evaluation of haloperoxidase activity (*39*). Additional advantage of using MCD bromination is that the bromination efficiency can be easily assessed by ultraviolet-visible (UV-vis) spectral analysis through measurement of absorption coefficient difference at ~290 nm [ε(MCD) =200 × 100 M^−1^ cm^−1^ versus ε(Br-MCD) = 100 M^−1^ cm^−1^]. Different iron ligands were first complexed with FeBr_3_ (FeBr_2_ was inferior in many cases, see the Supplementary Materials for details) and used as a catalyst (5 mol %) for MCD bromination, and the MCD conversion was presented in Fig. 2B.

We examined four different types of iron ligands: bidentate L1-L3 [e.g., oxalate, 5-sulfosalicylic acid (*40*), and picolinic acid (*41*)]; tridentate L14-L17 {e.g., (*E*)-2-[[(2-piperazin-1-yl)ethylimino]methyl]phenol, 2-methoxy-6-[(quinolin-8-ylimino)methyl]phenol, and terpyridines (*34*, *35*)}; tetradentate L4, L6, and L9-L13 [e.g., tartaric acid sodium salt, ethylenediamine-*N*,*N*′-diacetic acid sodium salt, tris(2-pyridylmethyl)amine, tris (dimethylaminoethyl)amine, bis-pyridine-1,2-diamines, and tetraphenyl porphyrin]; and polydentate L5 and L7-L8 [tripolyphosphate, ethylenediaminetetraacetate (*33*), and tetraethylenepentamine]. MCD bromination with these iron complexes as the catalyst suggested that the tridentate and tetradentate ligands (up to 72%) were superior to bidentate and polydentate ones (up to 27%) (Fig. 2B). This finding was notably inconsistent with the relative stability of iron complexes in the literatures (*42*): polydentate > tetradentate > tridentate > bidentate. We concentrated our efforts on further optimization of tridentate terpyridines (TPYs; L17) for FeBr_3_. It should be noted that TPY-Fe complexes had been designed and used to improve Fenton chemistry for degradation of pollutants and sewage treatment (*34*, *35*). Bromo substituent on the ortho-position of pyridine (R_1_ = Br, TPY2) was detrimental to the MCD bromination (49%) as compared to parent TPY1 (72%). Electron-donating groups (OMe and *t*-Bu) at pyridine (TPY3 and TPY4) did not enhance substantially the conversion of MCD (71 to 75%). We found that the para-substitution (R_3_) on the central pyridine had noticeable effect on the catalytic efficiency of the iron complex, and a series of 4′-substituted TPYs (i.e., 4′-Ph, 4′-aryl, 4′-furanyl, 4′-thiophenyl, 4′-Cl, and 4′-OMe) were synthesized for evaluation (see the Supplementary Materials for more TPYs). 4′-Furanyl and 4′-thiophenyl substituents were slightly less effective (63 to 65%; see the Supplementary Materials) than the parent TPY1 (72%), while 4′-phenyl group (TPY5) had a slim advantageous effect (77%). Electronic effects were also investigated on the phenyl ring with both electron-withdrawing (TPY6) and electron-donating groups (TPY7), and the result showed that EDG performed better than EWG (67% versus 80%). Last, we discovered that the strong electron-donating methoxy (OMe) at the para-position of central pyridine (TPY10) profoundly boosted the MCD conversion (84%). TPY10 was identified to be the optimal chelating ligand of FeBr_3_ for MCD bromination. Magnetic moment determination using Evans method (*43*) showed that Fe(TPY10)Br_3_ complex is a high-spin ferric species with relative low value of μ_eff_ (4.96 μ_B_). It was noteworthy that “ligandless” FeBr_3_ was ineffective for this MCD bromination with only 4% conversion, which was about 21 times less efficient than Fe(TPY10)Br_3_.

To further verify the catalytic efficiency of Fe(TPY10)Br_3_ for in situ generation of HOBr, phenol red (*44*) (standard chemical probe for in situ HOBr) was used for bromination with H_2_O_2_ and NH_4_Br (Fig. 2C). The bromination occurred rapidly to produce tetrabromophenol blue, which absorbed UV-vis light at 595 nm (λ_max_ = 598 nm) (*45*) (phenol red: λ_max_ = 429 nm). In contrast, FeBr_3_ without nitrogen-based ligand was not an effective catalyst for bromination of phenol red with H_2_O_2_ and NH_4_Br under otherwise identical condition (absorbance at λ_595nm_ was negligible). Last, we observed that the Fe(TPY10)Br_3_ complex was air stable and could form a homogenous solution with H_2_O_2_ for >12 hours (Fig. 2D), which differed from ligandless hygroscopic FeBr_3_ that reacted with H_2_O_2_ (within 10 min) to generate sludge [e.g., Fe(OH)_3_]. These physical and chemical properties might account for the superior catalytic efficiency of Fe(TPY10)Br_3_ over FeBr_3_.

### Applications of nonheme iron catalyst Fe(TPY10)Br_3_ as a functional mimic of heme-iron–dependent haloperoxidase for bromination and oxidation

#### Arene bromination

**Fig. 3.**
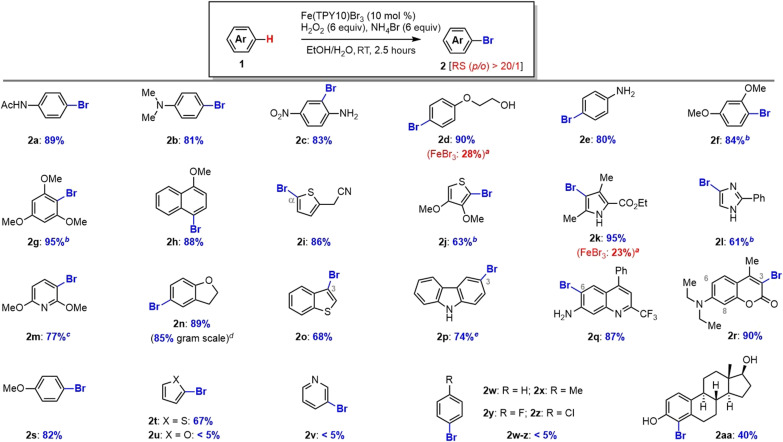
Arene bromination with Fe(TPY10)Br_3_/H_2_O_2_/NH_4_Br. Reaction condition: Arene (1 mmol), Fe(TPY10)Br_3_ (0.1 equiv), NH_4_Br (6.0 equiv), H_2_O_2_ (6.0 equiv), EtOH/H_2_O (3/1, 10 ml), 2.5 hours at room temperature (RT). *^a^*FeBr_3_ (0.1 equiv) was added. *^b^*Less Fe(TPY10)Br_3_ (0.05 equiv) was needed. *^c^*Fe(TPY10)Br_3_ (0.15 equiv) was needed. *^d^*Yield obtained from gram-scale reaction of **1n** (1.2 g). *^e^*Acetone/H_2_O (3/1, 10 ml) was used as the solvent. RS (*p*/*o*), Regioselectivity (para/ortho).

With the identification of nonheme iron catalyst [Fe(TPY10)Br_3_] as an effective functional mimic of haloperoxidase, we set out to investigate its catalytic efficiency for arene bromination with H_2_O_2_ and NH_4_Br. Arylbromides are an important class of organic compounds with diverse application in organic synthesis as exemplified by a plethora of transition metal-catalyzed cross-coupling reactions (*46*) using arylbromides as the electrophiles (i.e., Suzuki-Miyaura, Negishi, Stille, Heck, Sonogashira, and Buchwald-Hartwig). The arene bromination was performed with [Fe(TPY)Br_3_] (10 mol %) catalyst in EtOH/H_2_O (v/v, 3:1) at room temperature (RT) for ~2.5 hours (Fig. 3). We found that electron-rich arenes (**2a**-**2h** and **2s**) and heterocycles (**2i**-**2r** and **2t**) were suitable for efficient bromination with our catalytic system, delivering the mono-bromo arenes with excellent regioselectivity (>20:1) in high yields. Notably, oxidation-sensitive functional groups such as tertiary and primary amines (**2b**-**2c**), primary alcohol (**2d**), and complex steroid scaffold (**2aa**) were well tolerated in excellent yields. The efficient and regioselective bromination of thiophenes (**2i**, **2j**, and **2t**), pyrrole (**2k**), imidazole (**2l**), pyridine (**2m**), dihydrobenzofuran (**2n**), benzothiophene (**2o**), carbazole (**2p**), quinoline (**2q**), and coumarin (**2r**) demonstrated the powerfulness and usefulness of this catalytic method. However, no product was detected for the electron-deficient arenes (e.g., unbiased pyridine, **2v**; benzene, **2w**; toluene, **2x**; fluorobenzene, **2y**; and chlorobenzene, **2z**), which suggested the electrophilic nature of bromination reaction with in situ–generated brominating species (e.g., HOBr) (Note: Hammett slope ρ of −2.78). It was noted that furan did not undergo the bromination (**2u**), which might be attributed to the oxidative ring opening (*47*–*49*). For comparison, bromination with FeBr_3_ instead of [Fe(TPY10)Br_3_] proceeded with much lower yields as illustrated by **2d** (28%) and **2k** (23%), which were consistent with results of bromination of MCD and phenol red (Fig. 2) and those of our previous work (*37*, *38*).

#### Hunsdiecker-type decarboxylative bromination

**Fig. 4.**
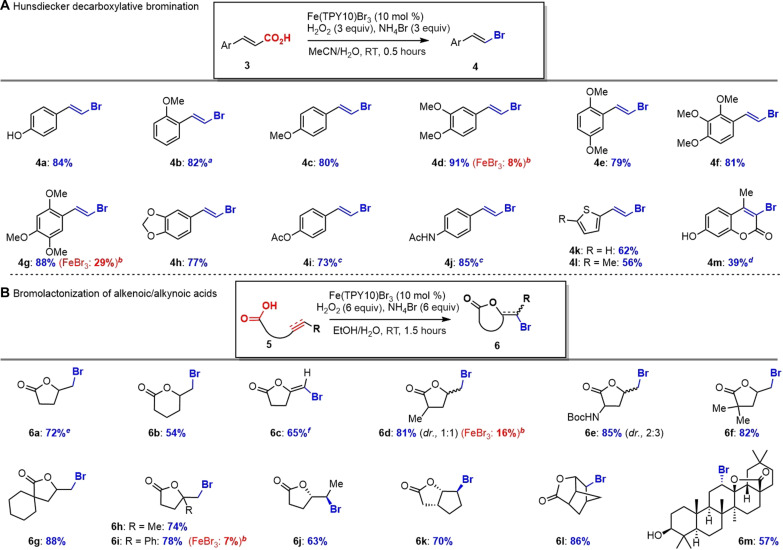
Hunsdiecker-type decarboxylative bromination of cinnamic acid and bromolactonization of alkenoic/alkynoic acids. (**A**) Condition: Cinnamic acid (1 mmol), Fe(TPY10)Br_3_ (0.1 equiv), NH_4_Br (3.0 equiv), H_2_O_2_ (3.0 equiv), MeCN/H_2_O (3/1, 10 ml), 0.5 hours at RT. (**B**) Condition: Alkenoic/alkynoic acid (1 mmol), Fe(TPY10)Br_3_ (0.1 equiv), NH_4_Br (6.0 equiv), H_2_O_2_ (6.0 equiv), EtOH/H_2_O (3/1, 10 ml), 1.5 hours at RT. *^a^*EtOH/H_2_O (3/1, 10 ml) was the solvent. *^b^*FeBr_3_ (0.1 equiv) was added. *^c^*NH_4_Br (6.0 equiv) and H_2_O_2_ (6.0 equiv) were needed. *^d^*LiOAc (1.0 equiv) was needed. *^e^*H_2_O (10 ml) was the solvent. *^f^*DCM/H_2_O (3/1, 10 ml) was the solvent.

We were very interested in Hunsdiecker-type decarboxylative bromination (*50*, *51*) of α,β-unsaturated carboxylic acids (*52*–*54*) with our newly established Fe(TPY10)Br_3_(cat.)/H_2_O_2_/NH_4_Br (Fig. 4A) because (i) the resulting vinylbromides are extremely useful functional groups with extensive applications in organic synthesis, (ii) previous functional mimics of haloperoxidases have not demonstrated the efficacy with this reaction, and (iii) our Fenton-bromide (CeBr_3_/H_2_O_2_ or FeBr_2_/H_2_O_2_) was not effective to promote such Hunsdiecker bromination [**4d** (8%) and **4g** (29%)]. To our delight, decarboxylative bromination of various trans-cinnamic acids containing different substituents on the benzene (**4a**-**4j**) was carried out successfully with excellent yields (73 to 91%), and only trans-configuration of the styrenyl bromides was obtained on the basis of analysis of nuclear magnetic resonance (NMR) coupling constant (see details in the Supplementary Materials). Notably, phenol (**4a**) survived without oxidation or bromination and other cinnamic acids bearing electron-rich groups including (mono*-*, di*-*, and tri-) methoxy group (**4a**-**4g**), dioxole (**4h**), OAc (**4i**), and NHAc (**4j**) on the benzene moiety underwent smooth Hunsdiecker-type decarboxylative bromination without detection of arene bromination. In addition, our method successfully enabled decarboxylative bromination of two thienyl propenoic acids with good yields [**4k** (62%) and **4l** (56%)] as well as complex coumarin-3-carboxylic acid (**3m**).

#### Bromolactonization

The successful Hunsdiecker-type decarboxylative bromination of trans-cinnamic acids prompted us to explore the possible bromolactonization of alkene-/alkyne-tethered carboxylic acids (alkenoic acids) (*55*), which differed from cinnamic acids by two or three saturated carbon linkage of the alkene and carboxylic acid functional groups. Such structural difference might give rise to a competition of decarboxylation and bromination to produce a mixture of products. Notably, the vanadium-dependent CPO from *Curvularia inaequalis* (CiVCPO) (*56*) and two functional mimics (V_2_O_5_) (*57*, *58*) have demonstrated efficient halolactonization with H_2_O_2_ and halide. However, there is no report on the related reaction with heme-dependent haloperoxidase or its functional mimics (iron-based catalysts) including our Fenton-halide method. Eleven alkenoic acids, one alkynoic acid, and complex oleanolic acid (**5m**) were examined for possible bromolactonization with our Fe(TPY10)Br_3_ catalyst, and the results are presented in Fig. 4B. All alkenoic/alkynoic acids underwent smooth bromolactonization to provide the corresponding lactones with good to excellent yields (up to 88%). The trans-configuration of the bromo and lactone oxygen within **6c** and **6j**-**6m** suggested an ionic [Br^+^] mechanism, consistent with iron-catalyzed in situ generation of HOBr from H_2_O_2_ and NH_4_Br. As a comparison, Fenton-halide (FeBr_3_/H_2_O_2_) delivered poor yield of the lactones [**6d** (16%) and **6i** (7%)]. These results further corroborated the higher catalytic efficiency of Fe(TPY)Br_3_ than FeBr_3_.

#### Sulfide/thiol oxidation

**Fig. 5.**
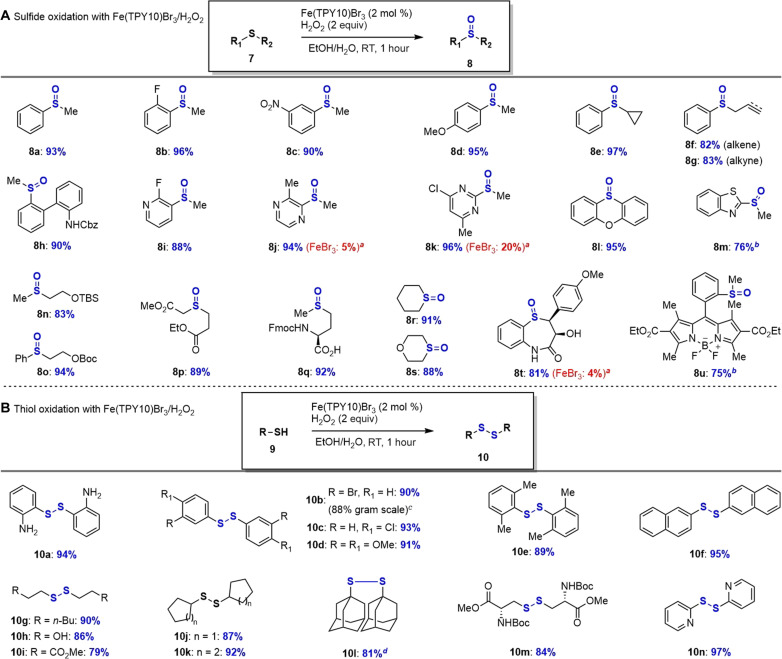
Fe(TPY10)Br_3_-catalyzed sulfide/thiol oxidation. (**A**) Condition: Sulfide (1 mmol), Fe(TPY10)Br_3_ (0.02 equiv), H_2_O_2_ (2.0 equiv), EtOH/H_2_O (3/1, 10 ml), 1 hour at RT. (**B**) Condition: Thiol (1 mmol), Fe(TPY10)Br_3_ (0.02 equiv), H_2_O_2_ (1.5 equiv), THF (10 ml), 0.5 hours at RT. *^a^*FeBr_3_ (0.02 equiv) was added. *^b^*Fe(TPY10)Br_3_ (0.03 equiv) and H_2_O_2_ (4.0 equiv) were needed. *^c^*Yield obtained from 1.89 g of **9b**. *^d^*Fe(TPY10)Br_3_ (0.03 equiv) and H_2_O_2_ (3.0 equiv) were needed.

It was noted that bromination of arenes, cinnamic acids, and alkenoic acids produced bromo-containing products (Figs. 3 and 4) and thus required stoichiometric bromide source (NH_4_Br) in addition to the catalytic amount of bromide from Fe(TPY10)Br_3_ catalyst. We envisioned that the reaction of Fe(TPY10)Br_3_ and H_2_O_2_ might generate the reactive brominating species (RBS; HOBr) without additional bromide source. If RBS (HOBr) can be used as a catalyst but not a stoichiometric reagent for an oxidation reaction that yields the product without bromide, then Fe(TPY10)Br_3_ could be used as a dual function catalyst (both iron and bromide act as a catalyst) for RBS-mediated oxidation reaction using H_2_O_2_ as terminal green oxidant. This dual catalysis from a single catalyst [Fe(TPY10)Br_3_] would be mechanistically interesting for its synergistic and cooperative manner. To verify this hypothesis, we chose sulfide oxidation to sulfoxide and thiol oxidation to disulfide (Fig. 5) (*59*). One challenge of using an iron catalyst for sulfur oxidation lies on the well-known strong complexation of iron with sulfide/thiols, which deactivated (poisoned) the iron catalyst (*60*), although a few iron catalysts were designed and developed for oxidation of sulfides with hydrogen peroxides (*61*, *62*).

We first examined the sulfide oxidation (Fig. 5A) with Fe(TPY10)Br_3_(2 mol %)/H_2_O_2_ to sulfoxides, which play an important role in organic synthesis [e.g., various dimethyl sulfoxide (DMSO)–based oxidations (*63*–*65*) including Swern, Parikh-Doering, Moffatt oxidation, Pummerer, and Mislow-Evans rearrangements]. Twenty sulfides were subjected to oxidation using 2 mol % Fe(TPY10)Br_3_ as the dual function catalyst at RT and the corresponding sulfoxides were obtained with high yields (75%–97%) without over-oxidation. The higher reactivity of sulfides/thiols toward HOBr than arenes/alkenes (Figs. 3 and 4) permitted a lower loading of our iron catalyst (2 mol %). It was noted that the electron-rich arene moieties (**8d**, **8h**, **8l**, and **8t**) and alkene/alkyne of sulfides (**8f** and **8 g**) were not brominated or oxidized as competition with sulfide oxidation. It was noteworthy that common protecting groups such as Cbz (**8h**), TBS (**8n**), Boc (**8o**), and Fmoc (**8q**) were tolerated in this oxidation. Sulfides containing heterocycle ring such as pyridine (**8i**), pyrazine (**8j**), pyrimidine (**8k**), benzothiazole (**8m**), and boron dipyrromethene (**8u**) were suitable for oxidation (yield up to 96%). Comparison of catalytic efficiency between Fe(TPY10)Br_3_ and FeBr_3_ was conducted with heterocycles (pyrazine **8j**, pyrimidine **8k**, and lactam **8t**), which apparently indicated that Fe(TPY10)Br_3_ outperformed FeBr_3_ with up to 20 time (5% → 94%).

Next, we investigated thiol oxidation to disulfides, which play a vital role in some research fields including protein stabilization (*66*), rechargeable lithium batteries formation (*67*), and controlled drug delivery system development (*68*). As shown in Fig. 5B, both aromatic (**10a**-**10f**) and aliphatic thiols (**10g**-**10 m**) were dimerized successfully using Fe(TPY10)Br_3_ (2 mol %)/H_2_O_2_. The tolerance of oxidation-sensitive functional groups such as free amine (**10a**), free alcohol (**10h**), and electron-rich arene (**10d**) was noteworthy. Oxidative dimerization of naphthalene thiol (**9f**), heterocycle (**9n**), and sterically hindered adamantane (**9l**) was achieved with high yields (up to 97%). Efficient disulfide formation of cysteine derivatives (**10m**) by Fe(TPY10)Br_3_(2 mol %)/H_2_O_2_ might be applicable to other cysteine-containing polypeptides and proteins to study and elucidate their biological functions.

### Mechanistic study and hypothesis

**Fig. 6.**
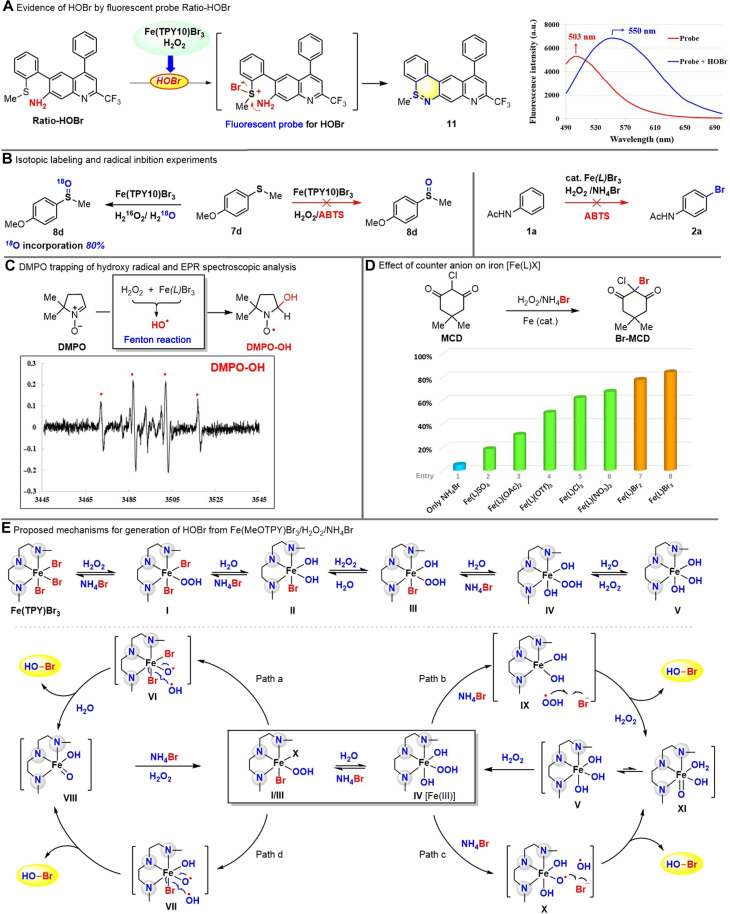
Control experiments and proposed mechanism for our Fe(TPY)Br_3_/H_2_O_2_. Note: 5% yield was used in the chart for the yields lower than 5%. (**A**) Evidence of HOBr by fluorescent probe Ratio-HOBr. (**B**) Isotopic labeling and radical inhibition experiments. (**C**) DMPO trapping of hydroxy radical and EPR spectroscopic analysis. (**D**) Effect of counter anion on iron [Fe(L)X]. (**E**) Proposed mechanisms for generation of HOBr from Fe(MeOTPY)Br_3_/H_2_O_2_/NH_4_Br.

The reaction of Fe(II/III) with hydrogen peroxide is a powerful method to generate high-valent iron [Fe(IV/V)] species or highly reactive hydroxyl/hydroperoxyl radicals (Fig. 1A). In the presence of halide, the oxidation of halide could occur to generate halide radical (X^⦁^) or hypohalide (HOX) (*24*, *25*) [Fig. 1 (iii) and (iv)] for halogenation or oxidation. Clear differentiation of these two pathways was challenging because halide radical might dimerize and then react with water to form hypohalide, while hypohalide could undergo homolytic cleavage to generate halide radical. Although we recognized the difficulty for such mechanistic investigations, some controlled experiments were designed and performed. In addition to the capture of HOBr with MCD and phenol red (Fig. 2, A and C), we used Ratio-HOBr (*69*), a specific and real-time fluorescent probe for HOBr detection with quick response, high sensitivity, and selectivity, to confirm the generation of HOBr from Fe(TPY10)Br_3_(6 mol %)/H_2_O_2_, which would be expected to support the ionic mechanism of sulfide/thiol oxidation. Our experimental results (Fig. 6A) substantiated that Fe(TPY10)Br_3_(6 mol %)/H_2_O_2_ generated HOBr and oxidized the Ratio-HOBr into compound **11** (*69*) with maximum emission at 550 nm [NMR, High-resolution mass spectrum (HRMS), and infrared spectra for **11** were provided in the Supplementary Materials]. To further investigate whether Fe(TPY10)Br_3_(cat.)/H_2_O_2_ generated HOBr or high-valent Fe^IV/V^═O as the active oxidant for sulfide oxidation, we carried out the isotope labeling experiment for oxidation of sulfide **7d** in the presence of H_2_^18^O. It was found that sulfoxide **8d** incorporated 80% of ^18^O (mass spectrum), which supported HOBr as the active oxidant for sulfide oxidation (detail explanation and mechanism in the Supplementary Materials) according to the mechanistic difference of sulfide oxidation with high-valent iron (Fe═O) and HOBr. In addition, 2,2′-azino-bis(3-ethylbenzothiazoline-6-sulfonic acid) diammonium salt, a specific Fenton oxidant (HO^•^) scavenger, was found to inhibit not only sulfide oxidation but also arene bromination (Fig. 6B). This finding suggested that hydroxyl radical (HO^•^) was generated from both Fe(TPY10)Br_3_(cat.)/H_2_O_2_ and Fe(TPY10)Br_3_(cat.)/H_2_O_2_/NH_4_Br probably via the Fenton chemistry mechanism. To further support the involvement of the hydroxyl radical (HO^•^), we used 3,4-dihydro-2,3-dimethyl-2H-pyrrole 1-oxide (DMPO) to trap HO^•^ and recorded the electron paramagnetic resonance (EPR) spectrum of the DMPO-OH adduct (Fig. 6C). The characteristic 1:2:2:1 quartet (indicated by red points) with the hyperfine coupling constants of α_N_ = α^β^_H_ = 14.9 G (*70*) was in good agreement with DMPO-OH radical (from DMPO and hydroxy radical), which confirmed the presence of hydroxy radical in our Fe(TPY10)Br_3_/H_2_O_2_ system. The hydroperoxyl radical (HOO^•^), another characteristic reactive species from Fenton chemistry, was also captured by DMPO to generate DMPO-OOH adduct (detailed information in the Supplementary Materials).

To gain some insights into the effect of iron and its counter anion on the generation of HOBr, we examined different iron salts to form the iron-TPY complexes for MCD bromination (Fig. 6D). Without iron salt, the combination of NH_4_Br and H_2_O_2_ could not promote any bromination (<1%). The TPY complexes of FeSO_4_, Fe(OTf)_3_, Fe(OAc)_2_, FeCl_3_, and Fe(NO_3_)_3_ could catalyze the MCD bromination but were inferior to the Fe(TPY)Br_2_ or Fe(TPY)Br_3_. These results indicated that the Fe(II/III)’s counter anion (SO_4_^2−^, ^−^OAc, ^−^OTf, Cl^−^, NO_3_^−^, and Br^−^) has a notable influence, and Fe─Br bond was not essential but would be advantageous to the in situ generation of HOBr and bromination.

Although a detailed mechanism for oxidation with [Fe]/H_2_O_2_ system was rarely provided in the literature and the real active species (high-valent iron, radical, or ionic, etc.) are still controversial and complicated, which remains to be clarified with more experimental evidence. With extensive experimental results and accumulative understanding, we proposed a detailed mechanism that might account for the observation in bromination and oxidation with Fe(TPY)Br_3_/H_2_O_2_ system in the absence/presence of NH_4_Br (Fig. 6E). We believed that Fe(TYP)Br_3_ would undergo non-oxidation “ligand” exchange with water, hydrogen peroxide, and bromide in the aqueous solution to generate more than five possible intermediates (only showed I to V). When hydroperoxyl was bonded to iron (Fe─OOH), a redox reaction could occur to generate reactive species for oxidation. Therefore, I, III, and IV could serve as reactive intermediates to initiate the redox reaction as depicted in Fig. 6E: I → VI → VIII (path a); III → VII → VIII (path d); IV → X/IX → V (paths b and c). As Fe─Br bond was beneficial in the controlled experiment (Fig. 6D), intermediate I was believed to be the initial stage that underwent a redox reaction via homolytic cleavage (*5*) of FeO─OH bond (VI) (path a) and hydroxyl radical (Fig. 6, B and C) rebound to Fe─Br with release of the RBS HOBr (Fig. 6A) (*24*). Intermediate II from the initial stage was converted into III, which could either generate HOBr as intermediate I did (path a) or form intermediate IV through ligand exchange. Intermediate IV might be the real active catalyst when bromide was present as a catalyst [Fe(TPY)Br_3_(cat)/H_2_O_2_] for oxidation (sulfide/thiol oxidation; Fig. 5). Two possible pathways were proposed for generation of HOBr from intermediate IV. Path b featured the in situ generation of reactive hydroperoxyl radical (HOO^•^), which oxidized bromide into bromine radical via electron transfer process (*71*). Bromine radical could dimerize to produce bromine that reacted with water to generate HOBr. Path c proceeded with generation of hydroxy radical (X), which oxidized bromide into HOBr. Our current results could not differentiate these two pathways (*24*), although the hydroxyl radical was confirmed by our experiment, but its origin (from VI or X) could not be verified. Intermediate V and its isomeric form (water as the ligand under aqueous condition) could be the final byproduct [Fe(TPY10)(OH)_3_] from the iron catalyst [Fe(TPY10)Br_3_], and this final Fe(III) oxidation state was characterized by low-temperature EPR and Mössbauer spectra (*72*–*76*) (also detailed in the Supplementary Materials).

## DISCUSSION

We report the development of a nonheme iron catalyst that mimics the heme-iron–dependent haloperoxidases to catalyze the generation of hypobromous acid (HOBr) from H_2_O_2_/Br^−^ with high efficiency under mild condition for bromination and oxidation. We discovered that TPY ligand designed for Fenton chemistry was optimal for FeBr_3_ to form a stable nonheme iron catalyst [Fe(TPY)Br_3_], which catalyzed arene bromination, Hunsdiecker-type decarboxylative bromination, bromolactonization, and oxidation of sulfides and thiols. Mechanistic studies revealed that Fenton chemistry ([Fe]/H_2_O_2_) might operate to generate hydroxyl radical (HO^•^), which oxidize bromide ion [Br^−^] into reactive [Br^+^] (i.e., HOBr). This previously unidentified nonheme iron catalyst represents a biomimetic model for heme-iron–dependent haloperoxidases with potential applications in organic synthesis, drug discovery, and biology.

## MATERIALS AND METHODS

### General experimental procedures

Reactions were carried out in a round-bottom flask with vigorous stirring at RT with open-air condition, unless otherwise noted. Reactions were monitored by thin-layer chromatography (0.25 mm) on pre-coated silica gel plates. Flash chromatography was performed with silica gel 60 (particle size of 0.040 to 0.062 mm). ^1^H- and ^13^C-NMR spectra were recorded on a 400-MHz spectrometer (400 MHz for ^1^H and 100 MHz for ^13^C). Mass spectra were detected by electrospray ionization–time of flight (Agilent 6520 or G6125B) or gas chromatography–mass spectrometry (Agilent 7890A/5975C). Infrared spectrometry was recorded on Nicolet iS10 FT-IR (Thermo Fisher Scientific). UV-vis spectra were recorded on UV-2550 spectrophotometer (Shimadzu). Fluorescence emission spectra were recorded on LS-45 fluorescence spectrophotometer (PerkinElmer). EPR was recorded on E500 (Bruker). Elemental analysis data were obtained on a Perkin-Elmer 2400 CHN elemental analyzer. Solution magnetic moments were determined in DMSO-*d*_6_ or D_2_O at RT by the Evans method. Electrochemical measurements were performed on a CHI 660e electrochemical workstation (Shanghai Chen Hua Instrument Co. Ltd.). ^57^Fe Mössbauer spectrum was recorded on a conventional spectrometer with alternating constant acceleration of the γ-source (^57^Co/Rh, 0.925 gigabecquerels).

### General procedure for the bromination of arene

To a stirred solution of aromatic ring (1 mmol), NH_4_Br (294 mg, 3 mmol) and Fe(TPY10)Br_3_ (55.8 mg, 0.1 mmol) in EtOH/H_2_O (3/1, 10 ml) were added H_2_O_2_ (30%, 0.3 ml, 3 mmol) dropwise. After completion of the addition, the homogenous mixture was stirred vigorously at RT for 1.5 hours. Then, additional NH_4_Br (294 mg, 3 mmol) and H_2_O_2_ (30%, 0.3 ml, 3 mmol) were added, and the resulting mixture was continuously stirred at RT for 1 hour. The reaction was quenched by aqueous Na_2_S_2_O_3_ solution (0.2 M, 50 ml) and ethyl acetate (50 ml). The organic fractions were collected, and the aqueous phase was extracted with ethyl acetate (two times, 20 ml). The combined organic fractions were washed with H_2_O, dried over Na_2_SO_4_, filtered, and concentrated under reduced pressure. The resulting residue was purified by flash column chromatography.

### General procedure for sulfide oxidation

To a stirred solution of sulfide (1 mmol) and Fe(TPY10)Br_3_ (11.2 mg, 0.02 mmol) in EtOH/H_2_O (3/1, 10 ml) was added H_2_O_2_ (30%, 0.2 ml, 2 mmol) dropwise. After completion of the addition, the reaction mixture was stirred at RT for 1 hour. The reaction was quenched by aqueous Na_2_S_2_O_3_ solution (0.1 M, 50 ml) and ethyl acetate (50 ml). The organic fractions were collected, and the aqueous phase was extracted with ethyl acetate (two times, 20 ml). The combined organic fractions were washed with brine, dried over Na_2_SO_4_, filtered, and concentrated under reduced pressure. The resulting residue was purified by flash column chromatography.
