# Supplementary material for: Nonheme iron catalyst mimics heme-dependent haloperoxidase for efficient bromination and oxidation
Source: Sci Adv. 2024 Dec 4;10(49):eadq0028. doi: 10.1126/sciadv.adq0028 (PMC11616719; doi:10.1126/sciadv.adq0028)
Supplement: Supplementary file 1 — Supplementary Text Figs. S1 to S6 Tables S1 to S5 [file sciadv.adq0028_sm.pdf]

Science Advances
AAAS

Supplementary Materials for

Nonheme iron catalyst mimics heme-dependent haloperoxidase for efficient bromination and oxidation

Guodong Zhao *et al.*

Corresponding author: Guodong Zhao, 202001036@bucm.edu.cn; Haimin Lei, hm_lei@126.com; Rongbiao Tong, rtong@ust.hk

Sci. Adv. **10**, eadq0028 (2024)
DOI: 10.1126/sciadv.adq0028

This PDF file includes:

Supplementary Text
Figs. S1 to S6
Tables S1 to S5

1. General Information

Reactions were carried out in a round-bottom flask with vigorous stirring at room temperature with open-air condition, unless otherwise noted. Anhydrous methanol (MeOH), ethanol (EtOH), acetone, acetonitrile (MeCN), dichloromethane (DCM), dimethyl sulfoxide (DMSO), 1-butanol (*n*-BuOH) and toluene were used as received without further purification unless otherwise noted. Extra dry tetrahydrofuran (THF), *N,N*-dimethylformamide (DMF), $FeBr_2$ and $FeBr_3$ were purchased from Sigma-Aldrich. Solvents were used as received from commercial suppliers without prior purification for workup, extraction and column chromatography. Reactions were monitored by thin-layer chromatography (TLC, 0.25 mm) on pre-coated silica gel plates. Flash chromatography was performed with silica gel 60 (particle size 0.040-0.062 mm). ^{1}H- and ^{13}C-NMR spectra were recorded on a 400 MHz spectrometer (400 MHz for ^{1}H, 100 MHz for ^{13}C). Chemical shifts are reported in parts per million (ppm) as values relative to the internal chloroform (7.26 ppm for ^{1}H and 77.16 ppm for ^{13}C) or DMSO (2.50 ppm for ^{1}H and 39.52 ppm for ^{13}C). Abbreviations for signal coupling are as follows: s, singlet; d, doublet; t, triplet; q, quartet; m, multiplet. Mass spectra were detected by ESI-TOF (Agilent 6520 or G6125B), TSQ Quantum Access MAX triple quadrupole (Thermo Fisher Scientific) or GC-MS (Agilent 7890A/5975C). Infrared spectrometry was recorded on Nicolet iS10 FT-IR (Thermo). UV-Vis spectra were recorded on UV-2550 spectrophotometer (Shimadzu). Fluorescence emission spectra were recorded on LS-45 fluorescence spectrophotometer (PerkinElmer). Optical rotations were measured on Autopol I polarimeter (Rudolph Research Analytical) with $[\alpha]_D$ values reported in degrees. Melting points were determined on MP70 instrument (Mettler Toledo) without correction. Electron paramagnetic resonance (EPR) was recorded on E500 (Bruker). Elemental analysis data were obtained on a Perkin-Elmer 2400 CHN elemental analyzer. Solution magnetic moments were determined in DMSO-d_6 or D_2O at RT using the Evans method(*43*). Electrochemical measurements were performed on a CHI 660e electrochemical workstation (Shanghai Chen Hua Instrument Co., Ltd.). ^{57}Fe Mössbauer spectrum was recorded on a conventional spectrometer with alternating constant acceleration of the *γ*-source (^{57}Co/Rh, 0.925 GBq), which was kept at room temperature.

2. Ligand screening for the in-situ generation of Br^+.

2.1 Ligands preparation

MCD was synthesized according the published literature. And ligands were synthesized as described below, for which **L11** (Eur. J. Org. Chem. 2017, 6942), **L15** (Polyhedron, 2015, 94, 75), **TPY2** (Eur. J. Org. Chem. 2008, 2008, 3976), **TPY5** (RSC Adv. 2019, 9, 5158), **TPY6** (Tetrahedron 2007, 63, 381) and **TPY7** (Chem-Asian J. 2018, 13, 3169) were synthesized according to the published literature, and other ligands and reagents described in this manuscript can be purchased directly from Sigma-Aldrich Co. **L2** and **L6** were synthesized according to the published literature (Polyhedron 2001, 20, 1079) with simple acid-base (acid: 5-sulfosalicylic acid or ethylenediamine-*N,N'*-diacetic acid; base: 2 equivalent NaOH) reaction.

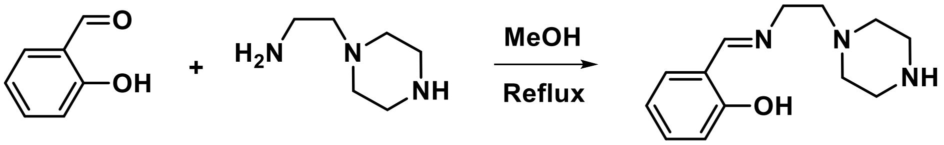

Synthesis of L14: To a stirred solution of salicylaldehyde **S1** (366 mg, 3 mmol) in anhydrous MeOH (20 mL) was added 1-(2-aminoethyl)-piperazine **S2** (388 mg, 3 mmol). After completion of the addition, the reaction mixture was allowed to reflux for 1 h. Then, the mixture was concentrated under reduced pressure, which was pure enough without further purification.

2-[[2-(Piperazin-1-yl)ethylimino]methyl]phenol (L14)

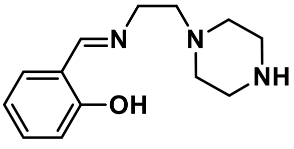

L14

L14 was a light-yellow oil (672 mg, 96%), which was pure enough for NMR detection. ^{1}H-NMR (400 MHz, $CDCl_3$) δ: 13.42 (brs, 1H), 8.34 (s, 1H), 7.31-7.26 (m, 1H), 7.23 (dd, J = 7.7, 1.7 Hz, 1H), 6.94 (dd, J = 8.3, 1.1 Hz, 1H), 6.85 (td, J = 7.5, 1.1 Hz, 1H), 3.72 (t, J = 6.6 Hz, 2H), 2.91-2.85 (m, 4H), 2.67 (t, J = 6.9 Hz, 2H), 2.49 (s, 4H). ^{13}C-NMR (100 MHz, $CDCl_3$) δ: 165.6, 161.3, 132.3, 131.3, 118.9, 118.6, 117.1, 59.4, 57.0, 54.8, 46.2. IR 3209.2, 2937.0, 2909.2, 1629.1, 1580.9, 1495.6, 1454.5, 1276.7, 1144.0, 1032.5, 838.5, 755.5 cm^{-1}. HRMS (ESI$^+$) (m/z) calcd. for $C_{13}H_{20}N_3O$ [M+H]$^+$ 234.1601; found 234.1600.

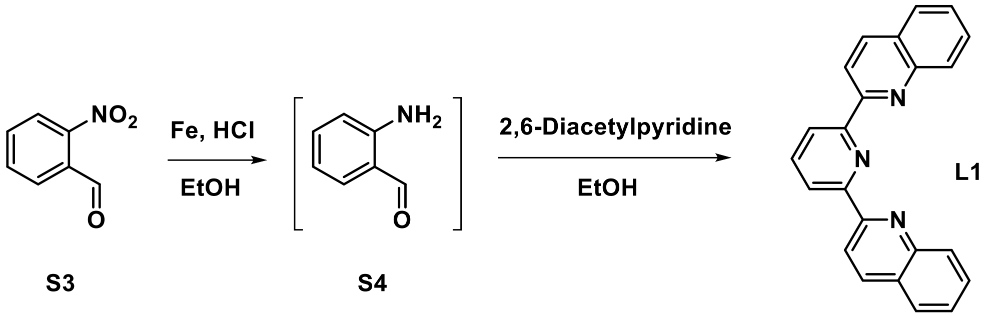

Synthesis of L16: To a stirred solution of *o*-nitrobenzaldehyde **S3** (756 mg, 5 mmol) in EtOH (25 mL) was added iron powder (2.8 g, 50 mmol) and 0.1 M HCl (10 mL). After completion of the addition, the reaction mixture was allowed to stir at 95°C for 2 h, followed by the addition of 2,6-diacetylpyridine (408 mg, 2.5 mmol) and KOH (842 mg, 15 mmol). Then, the mixture was stirred at 95°C for 4 h, cooled to room temperature, and concentrated under reduced pressure. The above residue was diluted by H_2O (100 mL) and ethyl acetate (100 mL). The organic fractions were collected, and the aqueous phase was extracted with ethyl acetate (2 ×20 mL). The combined organic fractions were washed with H_2O, dried over Na_2SO_4, filtered, and concentrated under reduced pressure.

2,6-Di(quinolin-2-yl)pyridine (L16)

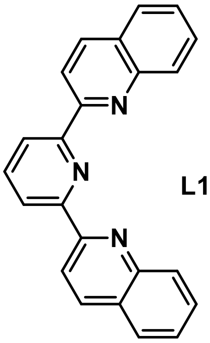

L16

L16 was purified by recrystallization from CH_2Cl_2 as a white solid (342 mg, 41%). M.P. 225-227 °C. ^{1}H-NMR (400 MHz, $CDCl_3$) δ: 8.90 (d, J = 8.6 Hz, 2H), 8.75 (d, J = 7.7 Hz, 2H), 8.62 (d, J = 8.6 Hz, 2H), 8.25 (s, 1H), 8.17 (d, J = 8.2 Hz, 2H), 8.09 (d, J = 7.9 Hz, 2H), 7.86 (s, 2H), 7.69 (s, 2H). ^{13}C-NMR (100 MHz, $CDCl_3$) δ: 156.3, 155.6, 148.1, 138.1, 136.9, 130.0, 129.7, 128.5, 127.8, 126.9, 122.2, 119.2. IR 3666.1, 2979.7, 2903.2, 1592.3, 1550.5, 1498.5, 1425.9, 1407.9, 1317.7, 1239.2, 1116.7, 1076.3, 811.6, 780.8, 730.4 cm^{-1}. HRMS (ESI$^+$) (m/z) calcd. for $C_{23}H_{16}N_3$ [M+H]$^+$ 334.1339; found 334.1339.

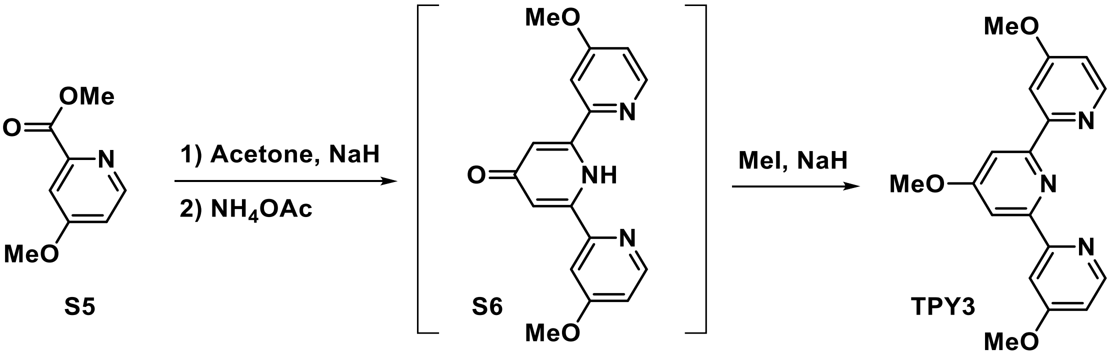

Synthesis of TPY3: A solution of methyl 4-methoxy-2-picolinate **S5** (836 mg, 5 mmol) and acetone (148 μL, 2 mmol) in dry THF (5 mL) was added dropwise to a stirred suspension of NaH (60%, 400 mg, 10 mmol) in THF (2 mL) under an N_2 atmosphere at rt. Then, the mixture was stirred at reflux for 2 h followed by solvent

evaporation under vacuo, and the resulting residue was slowly treated with water and acidified by 5% AcOH aqueous solution to pH 6.5. The obtained yellow solid was filtrated, washed by water and dried under vacuum, followed by the addition of EtOH (10 mL) and NH_4OAc (14 mmol, 1.08 g). The resulting residue was stirred at reflux for 2 h, concentrated in vacuo to half the original volume and cooled to rt. The solid that precipitated was collected by filtration, washed with Et_2O, dried under vacuum to furnish crude **S6** (309 mg). To a stirred solution of crude **S9** (309 mg, 1 mmol) in anhydrous DMF (10 mL) was added NaH (60%, 52 mg, 1.3 mmol). After completion of the addition, the reaction mixture was allowed to stir at 0°C for 15 min, followed by the addition of MeI (81 μL, 1.3 mmol) and the resulting mixture was stirred at rt for 1 h. Then, the reaction was quenched by H_2O (100 mL) and EtOAc (100 mL). The organic fractions were collected, and the aqueous phase was extracted with ethyl acetate (2 ×20 mL). The combined organic fractions were washed with H_2O, dried over Na_2SO_4, filtered, and concentrated under reduced pressure.

4,4',4"-Trimethoxy-2,2':6',2"-terpyridine (TPY3)

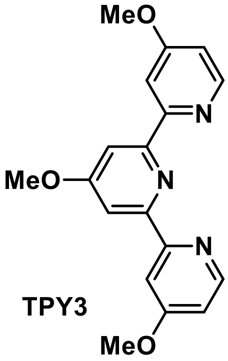

TPY3

TPY3 was purified by flash column chromatography (ethyl acetate/dichloromethane = 1:5 to 1:2) as a white solid (291 mg, 45% for 3 steps). M.P. 176-178 °C. ^{1}H-NMR (400 MHz, DMSO) δ: 8.52 (d, J = 5.7 Hz, 2H), 8.09 (d, J = 2.5 Hz, 2H), 7.95 (s, 2H), 7.07 (dd, J = 5.7, 2.6 Hz, 2H), 3.98 (s, 3H), 3.95 (s, 6H). ^{13}C-NMR (100 MHz, DMSO) δ: 167.3, 166.3, 156.6, 156.4, 150.6, 110.6, 106.7, 106.6, 55.6, 55.4. IR 3007.2, 2919.2, 2840.6, 1583.6, 1558.1, 1460.4, 1407.7, 1377.8, 1293.1, 1232.5, 1024.3, 818.1, 614.7, 455.2 cm^{-1}. HRMS (ESI^{+}) (m/z) calcd. for $C_{18}H_{18}N_3O_3$ $[M+H]^{+}$ 324.1343; found 324.1343.

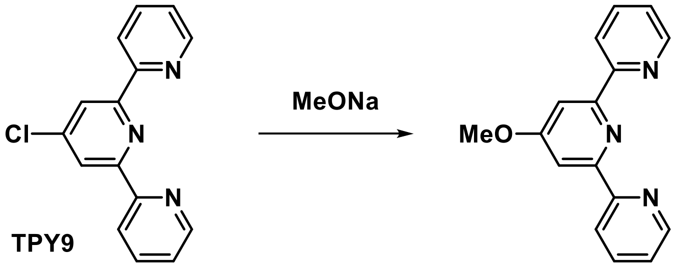

TPY9 TPY10

Synthesis of TPY10: To a stirred solution of commercially available **TPY9** (268 mg, 1 mmol) in anhydrous DMF (5 mL) was added MeONa (216 mg, 4 mmol). After completion of the addition, the reaction mixture was allowed to stir at 120°C for 4 h. After cooling, the solution was diluted by H_2O (50 mL) and EtOAc (50 mL). The organic fractions were collected, and the aqueous phase was extracted with ethyl acetate (2 ×20 mL). The combined organic fractions were washed with H_2O, dried over Na_2SO_4, filtered, and concentrated under reduced pressure.

4'-Methoxy-2,2':6',2"-terpyridine (TPY10)

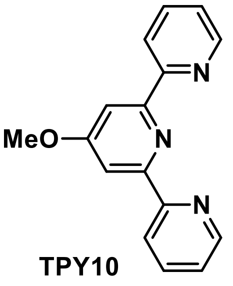

TPY10

TPY10 was purified by flash column chromatography (ethyl acetate/hexane = 1:5 to 1:1) as a light-yellow oil (184 mg, 70%). And the yield was almost identical (67%, 3.53 g) when large-scale reaction (**TPY9**: 20 mmol) was carried out. ^{1}H-NMR (400 MHz, DMSO) δ: 8.71 (d, *J* = 3.1 Hz, 2H), 8.60 (d, *J* = 7.9 Hz, 2H), 7.97 (d, *J* = 8.8 Hz, 4H), 7.48 (dd, *J* = 7.5, 4.8 Hz, 2H), 3.98 (s, 3H). ^{13}C-NMR (100 MHz, DMSO) δ: 167.3, 156.6, 154.8, 149.2, 137.3, 124.5, 120.9, 106.3, 55.6. IR 3364.8, 2920.3, 1567.0, 1462.7, 1406.0, 1356.6, 1249.6, 1205.7, 1028.4, 892.0, 785.3, 738.8 cm^{-1}. HRMS (ESI^{+}) (m/z) calcd. for $C_{16}H_{14}N_3O$ [M+H]$^{+}$ 264.1131; found 264.1131.

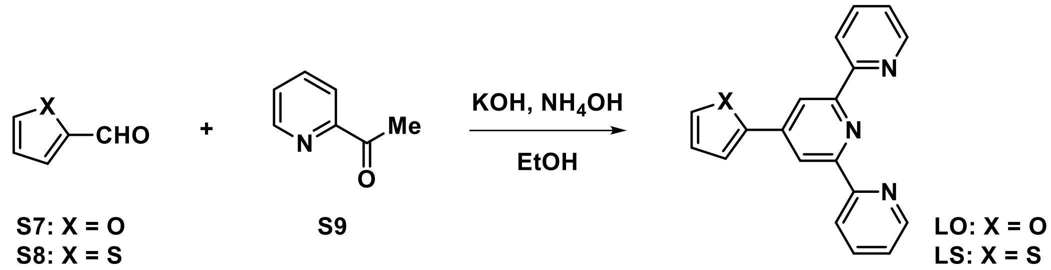

Synthesis of LO or LS: To a stirred solution of furan-2-carbaldehyde **S7** (480 mg, 5 mmol) or thiophene-2-carbaldehyde **S8** (561 mg, 5 mmol) in anhydrous EtOH (25 mL) was added 2-acetylpyridine **S9** (1.21 g, 10 mmol), KOH (0.77 g, 13.7 mmol) and aqueous NH_4OH (25%, 15 mL). After completion of the addition, the reaction mixture was allowed to stir at 70°C for 3 h, followed by filtration to get the final products.

4'-(2-Furyl)-2,2':6',2"-terpyridine (LO)

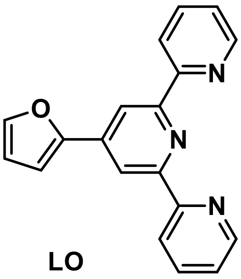

LO

LO was purified by recrystallization from CH_2Cl_2/hexane (5:1, 10 mL) as a white solid (703 mg, 47%), M.P. 224-226 °C. ^{1}H-NMR (400 MHz, DMSO) δ: 8.74 (ddd, *J* = 4.8, 1.8, 0.9 Hz, 2H), 8.66 (s, 2H), 8.61 (d, *J* = 8.0 Hz, 2H), 8.01 (td, *J* = 7.7, 1.8 Hz, 2H), 7.95 (dd, *J* = 1.8, 0.7 Hz, 1H), 7.51 (ddd, *J* = 7.5, 4.7, 1.2 Hz, 2H), 7.44 (dd, *J* = 3.5, 0.7 Hz, 1H), 6.73 (dd, *J* = 3.5, 1.8 Hz, 1H). ^{13}C-NMR (100 MHz, DMSO) δ: 155.6, 154.7, 150.7, 149.3, 145.0, 138.9, 137.4, 124.5, 120.8, 114.1, 112.7, 110.3. IR 3666.3, 2979.2, 2903.0, 1607.1, 1580.4, 1543.6, 1460.8, 1400.8, 1372.2, 1257.0, 1067.3, 1014.6, 886.6, 782.7, 728.0 cm^{-1}. HRMS (ESI^{+}) (m/z) calcd. for $C_{19}H_{14}N_3O$ [M+H]$^{+}$ 300.1131; found 300.1132.

4'-(2-Thiophenyl)-2,2':6',2"-terpyridine (LS)

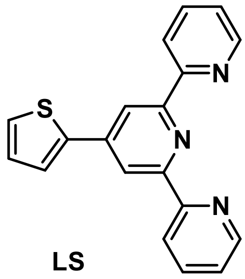

LS was purified by recrystallization from CH_2Cl_2 as a white solid (568 mg, 36%). M.P. 217-219 °C. ^{1}H-NMR (400 MHz, $CDCl_3$) δ: 8.75 (d, *J* = 4.7 Hz, 2H), 8.61 (d, *J* = 7.8 Hz, 4H), 8.01 (d, *J* = 8.1 Hz, 2H), 7.92 (d, *J* = 3.7 Hz, 1H), 7.79 (d, *J* = 4.9 Hz, 1H), 7.56-7.46 (m, 2H), 7.26 (d, *J* = 4.5 Hz, 1H). ^{13}C-NMR (100 MHz, $CDCl_3$) δ: 156.14, 156.13, 149.2, 143.5, 142.0, 137.0, 128.4, 127.2, 126.0, 124.0, 121.5, 117.3. IR 3667.2, 2979.3, 2903.5, 1572.4, 1456.7, 1391.3, 1256.3, 1229.2, 1050.4, 787.1, 704.1, 614.7 cm^{-1}. HRMS (ESI^+) (m/z) calcd. for $C_{19}H_{14}N_3S$ $[M+H]^+$ 316.0903; found 316.0903.

2.2 Iron complex formation and its catalytic efficiency for the MCD bromination.

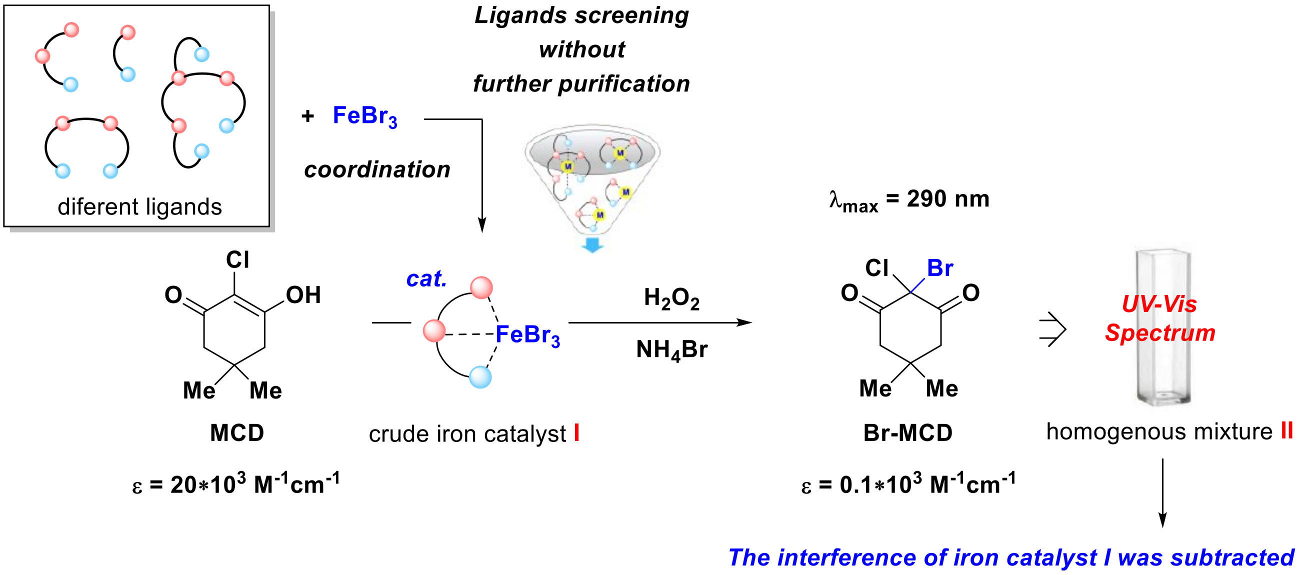

Ligand screening procedure: To a stirred solution of different ligand (0.2 mmol) in acetonitrile (MeCN, 4 mL) or H_2O (4 mL) was added $FeBr_3$ (59.1 mg, 0.2 mmol). After completion of the addition, the mixture was allowed to stir overnight from room temperature (rt) to 60°C under nitrogen, accelerating a thorough coordination (detected by TLC). Subsequently, the complex was concentrated and dried under vacuum to furnish a crude iron catalyst **I** with almost quantitative yield (~100%), for which 5.5 μmol of crude iron catalyst **I** was taken out and transferred to a flask, followed by the addition of MeCN/H_2O (3/1, 40 mL), 2-chloro-5,5-dimethyl-1,3-dimedone (MCD, 19 mg, 0.11 mmol), NH_4Br (54 mg, 0.55 mmol) and H_2O_2 (30%, 55 μL, 0.55 mmol). After completion of the addition, the homogenous mixture **II** was stirred vigorously at rt for 0.5 h. Then, 0.2 mL of the above mixture **II** was taken out, diluted by MeCN/H_2O (3/1, 3.8 mL) and analyzed by UV-Vis spectroscopy immediately. Ligands **L1**, **L2**, **L3**, **L4**, **L5**, **L6** and **L7** were coordinated with $FeBr_3$ in the solvent of H_2O. Ligands **L8**, **L9**, **L14**, **L15**, **L16** and **L17** were coordinated with $FeBr_3$ in the solvent of MeCN.

Notably, the interference of different iron catalysts and ligands for UV-Vis spectrum was excluded: UV-Vis absorptions of iron complex [crude residue **I** (6.88 μM) in MeCN/H_2O (3/1)] were subtracted simultaneously

for all the spectra provided in this paper to suppress the interference of different iron catalyst, and 6.88 µM was identical to the final concentration of diluted iron catalyst following the above procedure.

Table S1. Absorbances at λ_{max} = 293 nm and the conversion yields for the MCD bromination using different iron catalysts

Catalyst	Absorbance	Conversion
No	1.85	0.0%
$FeBr_3$	1.77	4.3%
Fe(**L1**)Br_3	1.75	5.4%
Fe(**L2**)Br_3	1.81	2.2%
Fe(**L3**)Br_3	1.57	15.1%
Fe(**L4**)Br_3	1.56	15.7%
Fe(**L5**)Br_3	1.73	6.5%
Fe(**L6**)Br_3	1.22	34.1%
Fe(**L7**)Br_3	1.71	7.6%
Fe(**L8**)Br_3	1.35	27.0%
Fe(**L9**)Br_3	0.75	59.5%
Fe(**L10**)Br_3	1.08	41.6%
Fe(**L11**)Br_3	1.51	18.4%
Fe(**L12**)Br_3	0.89	51.9%
Fe(**L13**)Br_3	1.24	32.9%
Fe(**L14**)Br_3	1.15	37.8%

Catalyst	Absorbance	Conversion
Fe(**L15**)Br_3	0.71	61.6%
Fe(**L16**)Br_3	0.76	58.9%
Fe(**TPY1**)Br_3	0.51	72.4%
Fe(**TPY2**)Br_3	0.95	48.6%
Fe(**TPY3**)Br_3	0.53	71.4%
Fe(**TPY4**)Br_3	0.47	74.6%
Fe(**TPY5**)Br_3	0.43	76.8%
Fe(**TPY6**)Br_3	0.61	67.0%
Fe(**TPY7**)Br_3	0.38	79.5%
Fe(**TPY8**)Br_3	0.34	81.6%
Fe(**TPY9**)Br_3	0.63	65.9%
Fe(**TPY10**)Br_3	0.29	84.3%
Fe(**TPY10**)**Br_2**	0.41	77.8%
Fe(**LO**)Br_3	0.69	62.7%
Fe(**LS**)Br_3	0.65	64.9%

Absorbances at λ_{max} = 293 nm were given as above, and the conversion yield was calculated: (1.85 - Absorbance)/1.85 × 100%, for which 1.85 was the absorbance at 293 nm without the addition of any catalyst, NH_4Br and H_2O_2. Thus, the greater loss of absorbance at 293 nm the higher conversion yield assessed.

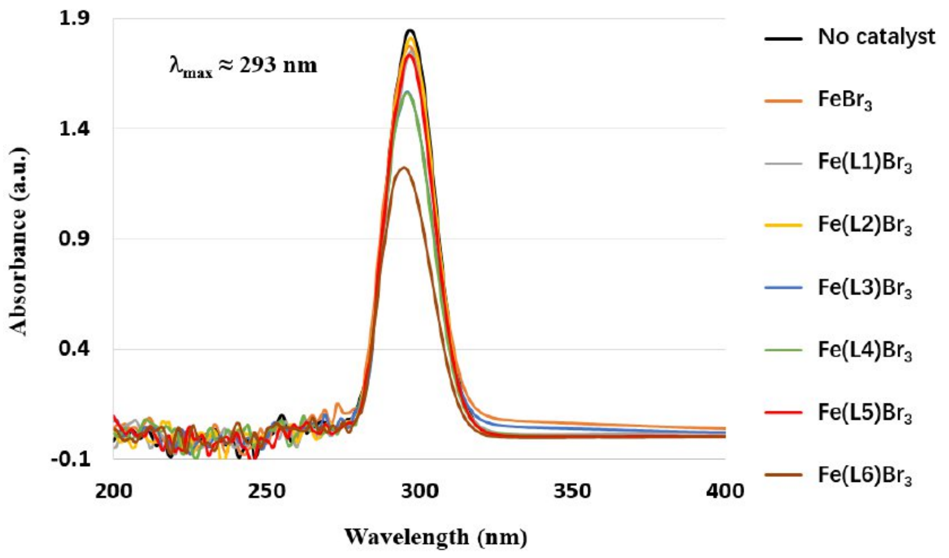

Figure S1. UV-Vis spectra for MCD bromination using catalysts: No catalyst, $FeBr_3$, Fe(**L1**)Br_3, Fe(**L2**)Br_3, Fe(**L3**)Br_3, Fe(**L4**)Br_3, Fe(**L5**)Br_3 and Fe(**L6**)Br_3

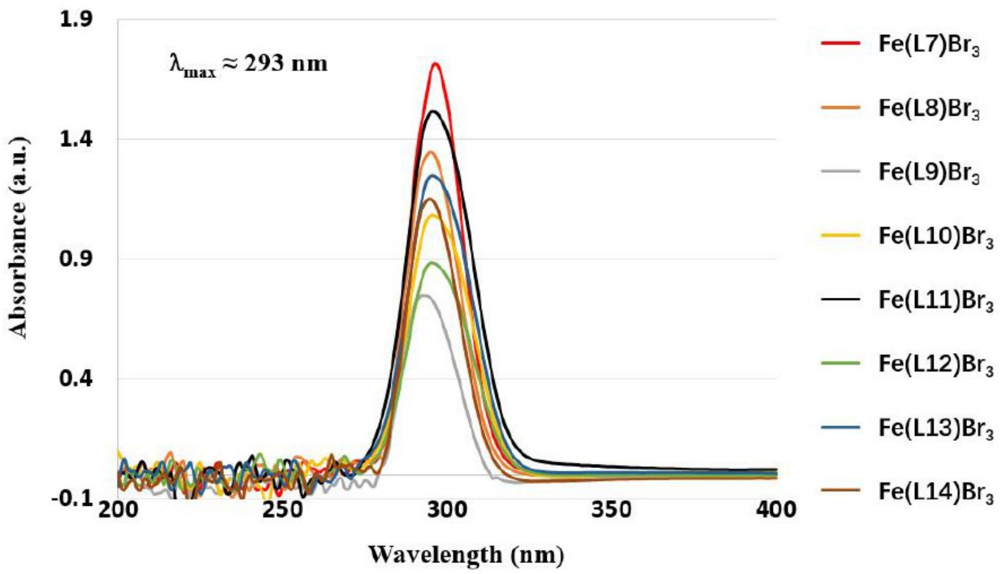

Figure S2. UV-Vis spectra for MCD bromination using catalysts: Fe(**L7**)Br_3, Fe(**L8**)Br_3, Fe(**L9**)Br_3, Fe(**L10**)Br_3, Fe(**L11**)Br_3, Fe(**L12**)Br_3, Fe(**L13**)Br_3 and Fe(**L14**)Br_3

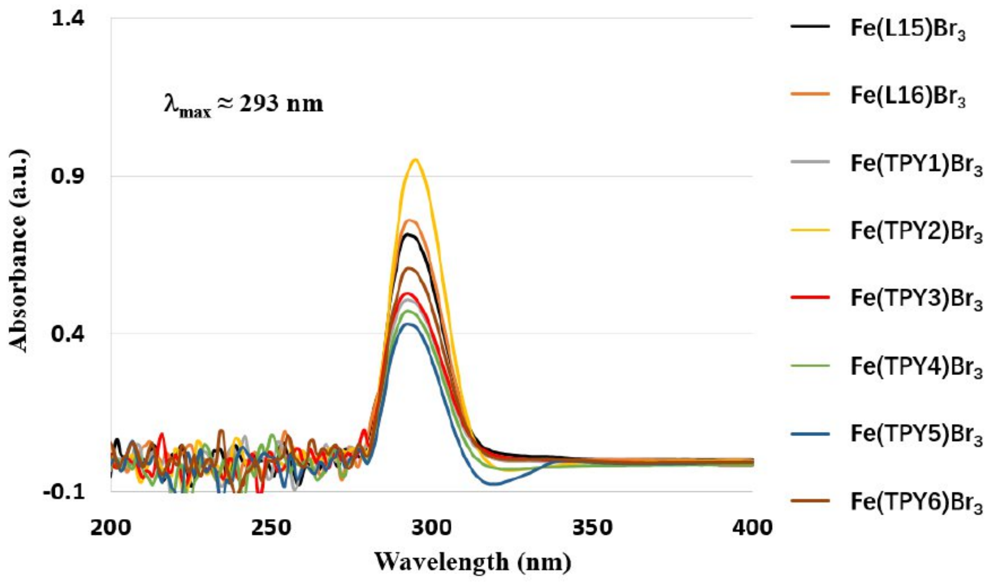

Figure S3. UV-Vis spectra for MCD bromination using catalysts: Fe(**L15**)Br_3, Fe(**L16**)Br_3, Fe(**TPY1**)Br_3, Fe(**TPY2**)Br_3, Fe(**TPY3**)Br_3, Fe(**TPY4**)Br_3, Fe(**LTPY5**)Br_3 and Fe(**TPY6**)Br_3

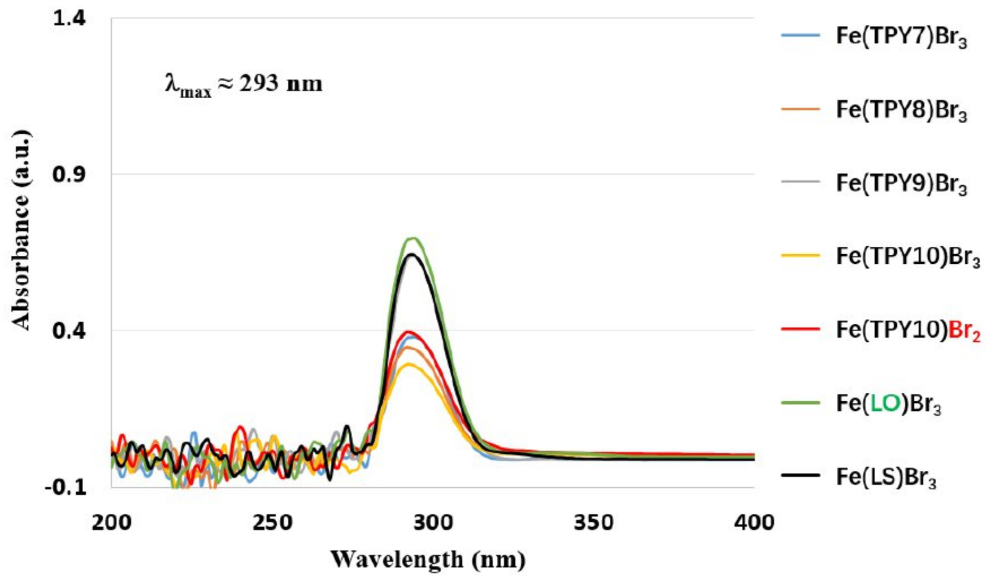

Figure S4. UV-Vis spectra for MCD bromination using iron catalysts: Fe(**TPY7**)Br_3, Fe(**TPY8**)Br_3, Fe(**TPY9**)Br_3, Fe(**TPY10**)Br_3, Fe(**TPY10**)Br_2, Fe(**LO**)Br_3 and Fe(**LS**)Br_3

2.3 Characterization of representative iron catalysts.

Notably, crude iron complex **I** without further purification was efficient for the ligand screening in MCD bromination, however, the crude iron coordination was not suitable for further detail characterization particularly for the efficient TPY analogues (**TPY1** to **LTPY10**) and $FeBr_3$. To furnish neat NMR spectra, iron catalysts were recrystallized from MeCN to eliminate the strong paramagnetism of remaining $FeBr_3$. Even though, catalysts **Fe(L16)Br$_3$**, **Fe(TPY2)Br$_3$**, **Fe(TPY8)Br$_3$** and **Fe(LO)Br$_3$** cannot be locked by ^{1}H-NMR, and

all their ^{13}C-NMR cannot be locked owing to the high concentration of iron complex, expect for **Fe(TPY1)Br$_3$** and **Fe(TPY10)Br$_3$**. HRMS, IR and melting point were also carried out for the representative iron catalysts, and the coordination with $FeBr_3$ was listed below with TLC for representative TPY ligands (**TPY1**, **TPY3**, **TPY4**, **TPY9** and **TPY10**). All the iron catalysts listed below were synthesized according to the above **ligand screening procedure** using different ligand (0.2 mmol) and $FeBr_3$ (59.1 mg, 0.2 mmol) in the solvent of MeCN (4 mL).

Fe[2,6-di(quinolin-2-yl)pyridine]Br$_3$ [Fe(L16)Br$_3$]

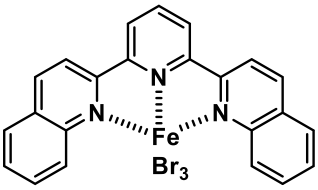

Fe(L16)Br$_3$

Fe(L16)Br$_3$ was recrystallized from MeCN as a brown solid (104 mg, 83%). M.P. > 350°C. IR 3071.2, 1591.1, 1562.9, 1514.0, 1484.1, 1435.0, 1380.4, 1347.4, 1270.6, 1218.6, 1192.8, 1145.7, 842.9, 812.4, 782.5 cm^{-1}. HRMS (ESI$^+$) (m/z) calcd. for $C_{23}H_{15}Br_2FeN_3$ [M-Br]$^+$ 548.8956; found 548.8950. Elemental analysis (%) calcd. for $C_{23}H_{15}Br_3FeN_3$: C, 43.92; H, 2.40; N, 6.68; found: C, 43.81; H, 2.37; N, 6.70. μ_{eff} = 4.50 μ_B (DMSO-d_6, 300K, Evans method).

Fe(2,2':6',2"-terpyridine)Br$_3$ [Fe(TPY1)Br$_3$]

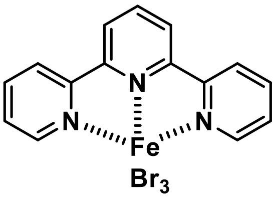

Fe(TPY1)Br$_3$

Fe(TPY1)Br$_3$ was recrystallized from MeCN as a black solid (93 mg, 88%). M.P. > 350°C. ^1H-NMR (400 MHz, DMSO) δ: 9.33-9.25 (m, 2H), 8.87-8.80 (m, 3H), 8.00 (t, J = 7.7 Hz, 2H), 7.21-7.11 (m, 4H). ^{13}C-NMR (100 MHz, DMSO) δ: 159.6, 157.6, 152.7, 138.8, 138.1, 127.6, 124.0, 123.9. IR 3364.0, 3068.4, 1596.8, 1570.3, 1471.1, 1443.9, 1394.7, 1315.0, 1244.1, 1159.8, 1020.7, 775.3, 730.3, 647.2 cm^{-1}. HRMS (ESI$^+$) (m/z) calcd. for $C_{15}H_{11}Br_2FeN_3$ [M-Br]$^+$ 448.8643; found 448.8642. ^{79}Br and ^{81}Br are equally abundant in the nature. Elemental analysis (%) calcd. for $C_{15}H_{11}Br_3FeN_3$: C, 34.07; H, 2.10; N, 7.95; found: C, 34.22; H, 2.09; N, 7.87. μ_{eff} = 5.22 μ_B (D$_2$O, 300K, Evans method).

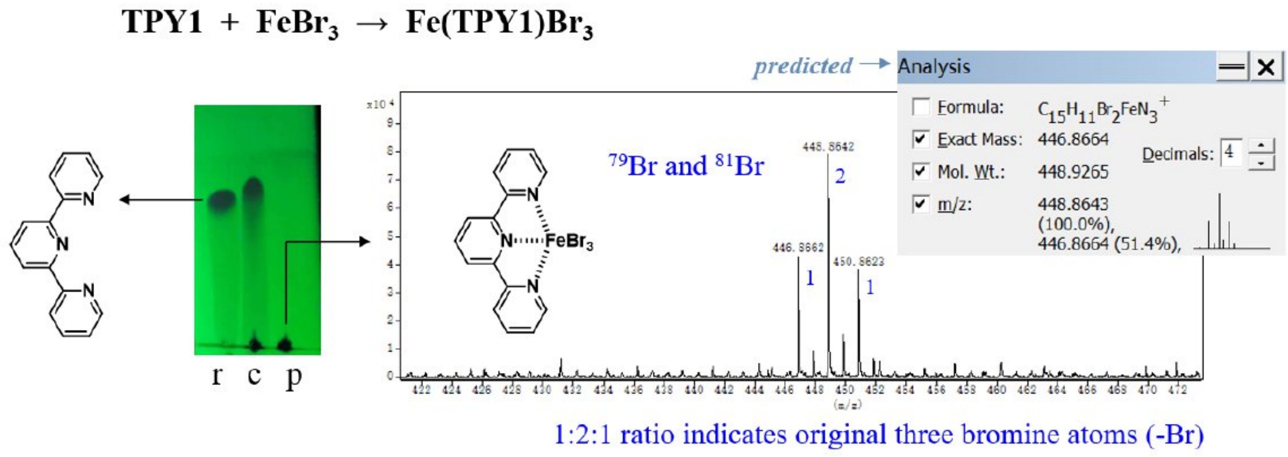

Fe[6,6"-dibromo-2,2':6',2"-terpyridine]Br$_3$ [Fe(TPY2)Br$_3$]

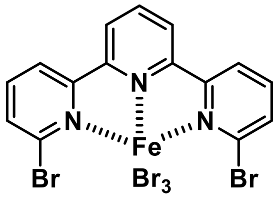

Fe(TPY2)Br3

Fe(TPY2)Br$_3$ was recrystallized from MeCN as a red-brown solid (100 mg, 73%). M.P. 240-242°C. IR 3076.7, 1551.3, 1453.2, 1422.9, 1387.0, 1243.4, 1180.0, 1134.6, 1067.5, 1021.1, 996.7, 853.0, 783.4 cm^{-1}. HRMS (ESI$^+$) (m/z) calcd. for $C_{15}H_9Br_4FeN_3$ [M-Br]$^+$ 606.6833; found 606.6847. Elemental analysis (%) calcd. for $C_{15}H_9Br_5FeN_3$: C, 26.24; H, 1.32; N, 6.12; found: C, 26.17; H, 1.40; N, 6.23. μ_{eff} = 5.32 μ_B (DMSO-d_6, 300K, Evans method).

Fe[4,4',4"-Trimethoxy-2,2':6',2"-terpyridine]Br$_3$ [Fe(TPY3)Br$_3$]

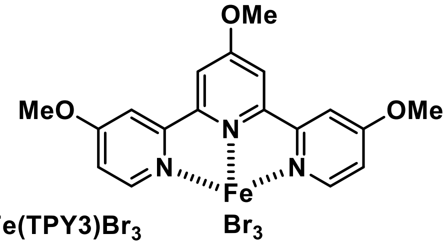

Fe(TPY3)Br3

Fe(TPY3)Br$_3$ was recrystallized from MeCN as a black solid (111 mg, 90%). M.P. > 350°C. ^{1}H-NMR (400 MHz, DMSO) δ: 8.99 (s, 2H), 8.49 (s, 2H), 6.87 (s, 2H), 6.75 (s, 2H), 4.38 (s, 3H), 3.86 (s, 6H). IR 3063.5, 1601.0, 1557.3, 1471.3, 1433.4, 1407.0, 1325.9, 1283.1, 1218.4, 1028.1, 855.9 cm^{-1}. HRMS (ESI$^+$) (m/z) calcd. for $C_{18}H_{17}Br_2FeN_3O_3$ [M-Br]$^+$ 538.8960; found 538.8954. ^{79}Br and ^{81}Br are equally abundant in the nature. Elemental analysis (%) calcd. for $C_{18}H_{17}Br_3FeN_3O_3$: C, 34.93; H, 2.77; N, 6.79; found: C, 34.88; H, 2.74; N, 6.82. μ_{eff} = 2.97 μ_B (D_2O, 300K, Evans method).

TPY3 + FeBr$_3$ → Fe(TPY3)Br$_3$

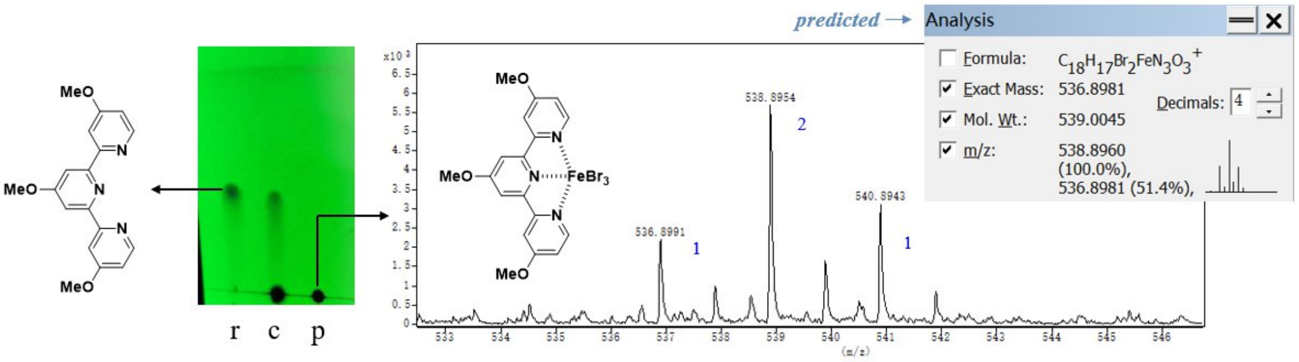

Fe[4,4',4"-tri(*tert*-butyl)-2,2':6',2"-terpyridine]Br$_3$ [Fe(TPY4)Br$_3$]

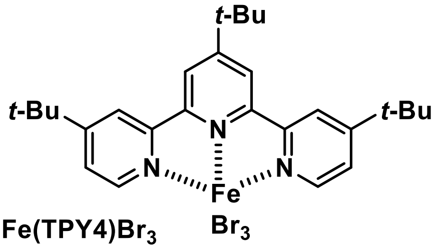

Fe(TPY4)Br3

Fe(TPY4)Br$_3$ was recrystallized from MeCN as a purple solid (120 mg, 86%). M.P. > 350°C. ^{1}H NMR (400 MHz, DMSO) δ: 9.50-8.70 (m, 4H), 7.25-6.75 (m, 4H), 1.80 (brs, 9H), 1.24 (brs, 18H). IR 3782.0, 3420.5, 2960.1, 2906.0, 2868.1, 1610.1, 1544.8, 1475.8, 1405.2, 1367.2, 1252.5, 1126.4, 1025.7, 896.4, 851.6, 568.6 cm^{-1}. HRMS (ESI$^+$) (m/z) calcd. for $C_{27}H_{35}Br_2FeN_3$ [M-Br]$^+$ 617.0521; found 617.0522. ^{79}Br and ^{81}Br are equally abundant in the nature. Elemental analysis (%) calcd. for $C_{27}H_{35}Br_3FeN_3$: C, 46.52; H, 5.06; N, 6.03; found: C, 46.59; H, 4.97; N, 6.11. μ_{eff} = 4.44 μ_B (DMSO-d_6, 300K, Evans method).

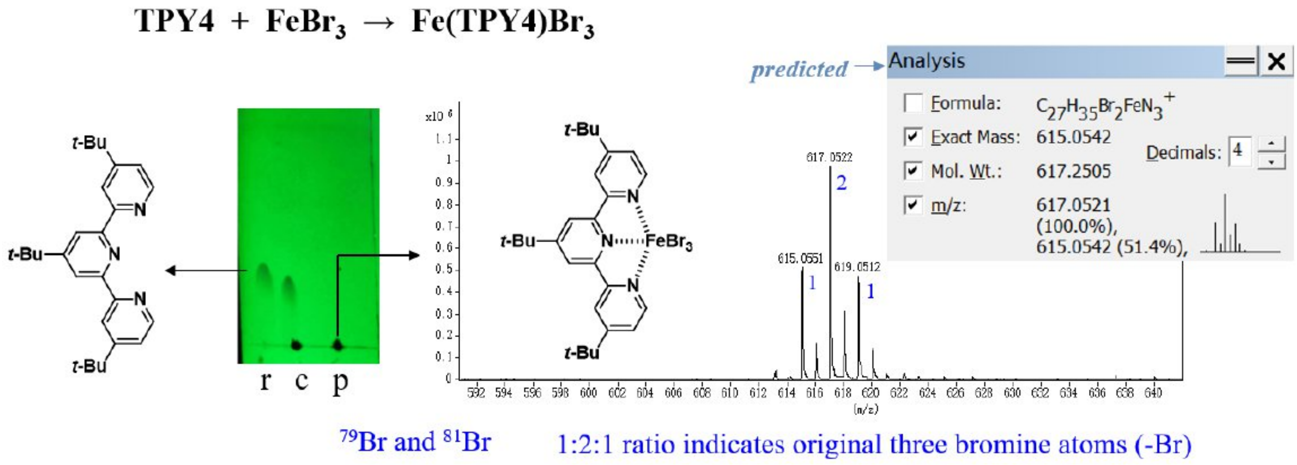

Fe[4'-phenyl-2,2':6',2"-terpyridine]Br$_3$ [Fe(TPY5)Br$_3$]

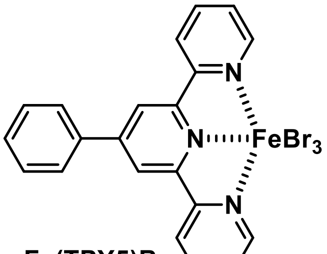

Fe(TPY5)Br$_3$

Fe(TPY5)Br$_3$ was recrystallized from MeCN as a black solid (100 mg, 83%). M.P. 320-322°C. ^{1}H NMR (400 MHz, DMSO) δ: 9.69 (brs, 2H), 9.09 (brs, 2H), 8.56 (brs, 2H), 8.04 (brs, 2H), 7.90-7.60 (m, 3H), 7.30-7.15 (m, 4H). IR 3056.5, 1602.3, 1545.5, 1472.3, 1412.9, 1244.4, 1156.1, 1021.6, 792.5, 762.4, 692.2 cm^{-1}. HRMS (ESI$^+$) (m/z) calcd. for $C_{21}H_{15}Br_2FeN_3$ [M-Br]$^+$ 524.8956; found 524.8957. Elemental analysis (%) calcd. for $C_{21}H_{15}Br_3FeN_3$: C, 41.70; H, 2.50; N, 6.95; found: C, 41.57; H, 2.41; N, 7.04. μ_{eff} = 2.57 μ_B (DMSO-d_6, 300K, Evans method).

Fe[4'-(4-chlorophenyl)-2,2':6',2"-terpyridine]Br$_3$ [Fe(TPY6)Br$_3$]

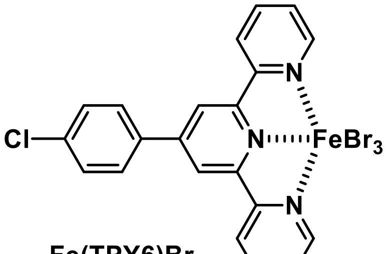

Fe(TPY6)Br$_3$

Fe(TPY6)Br$_3$ was recrystallized from MeCN as a black solid (115 mg, 90%). M.P. > 350 °C. NMR (400 MHz, DMSO) δ: 9.71 (brs, 2H), 9.08 (brs, 2H), 8.62 (brs, 2H), 8.10-7.90 (m, 4H), 7.30-7.15 (m, 4H). IR 3058.3, 2999.1, 1600.9, 1545.0, 1473.6, 1427.3, 1392.5, 1241.1, 1088.7, 1016.2, 825.6, 785.9, 750.7 cm^{-1}. HRMS (ESI$^+$) (m/z) calcd. for $C_{21}H_{14}Br_2ClFeN_3$ [M-Br]$^+$ 558.8566; found 558.8558. Elemental analysis (%) calcd. for $C_{21}H_{14}Br_3ClFeN_3$: C, 39.45; H, 2.21; N, 6.57; found: C, 39.55; H, 2.22; N, 6.59. μ_{eff} = 4.40 μ_B (DMSO-d_6, 300K, Evans method).

Fe[4'-(4-methoxyphenyl)-2,2':6',2"-terpyridine]Br$_3$ [Fe(TPY7)Br$_3$]

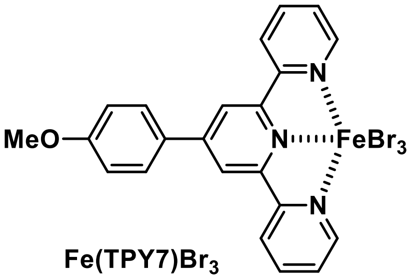

Fe(TPY7)Br$_3$

Fe(TPY7)Br$_3$ was recrystallized from MeCN as a black solid (116 mg, 91%). M.P. > 350°C. ^{1}H NMR (400 MHz, DMSO) δ: 9.64 (brs, 2H), 9.08 (brs, 2H), 8.57 (brs, 2H), 8.03 (brs, 2H), 7.45-7.11 (m, 6H), 3.98 (brs, 3H). IR 3667.7, 2979.8, 2902.6, 1595.0, 1515.4, 1468.7, 1406.8, 1242.9, 1181.6, 1064.0, 1023.5, 829.3, 785.9, 518.4 cm^{-1}. HRMS (ESI$^+$) (m/z) calcd. for $C_{22}H_{17}Br_2FeN_3O$ [M-Br]$^+$ 554.9062; found 554.9074. Elemental analysis (%) calcd. for $C_{22}H_{17}Br_3FeN_3O$: C, 41.62; H, 2.70; N, 6.62; found: C, 41.75; H, 2.69; N, 6.61. μ_{eff} = 2.16 μ_{B} (DMSO-d_6, 300K, Evans method).

Fe[4'-(4-carboxyphenyl)-2,2':6',2"-terpyridine]Br$_3$ [Fe(TPY8)Br$_3$]

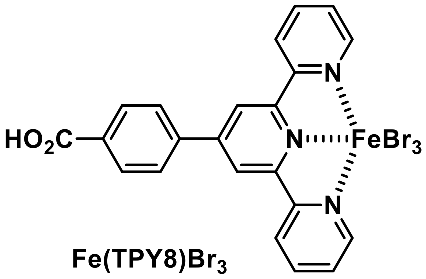

Fe(TPY8)Br$_3$

Fe(TPY8)Br$_3$ was recrystallized from MeCN as a brown solid (94.7 mg, 73%). M.P. > 350°C. IR 3060.0, 2819.8, 2670.1, 2538.1, 1686.4, 1598.2, 1525.6, 1473.9, 1424.6, 1404.5, 1292.8, 1019.9, 942.8, 862.6, 777.6 cm^{-1}. HRMS (ESI$^+$) (m/z) calcd. for $C_{22}H_{15}Br_2FeN_3O_2$ [M-Br]$^+$ 568.8854; found 568.8870. Elemental analysis (%) calcd. for $C_{22}H_{15}Br_3FeN_3O_2$: C, 40.72; H, 2.33; N, 6.48; found: C, 40.90; H, 2.41; N, 6.59. μ_{eff} = 6.27 μ_{B} (DMSO-d_6, 300K, Evans method).

Fe[4'-(chloro)-2,2':6',2"-terpyridine]Br$_3$ [Fe(TPY9)Br$_3$]

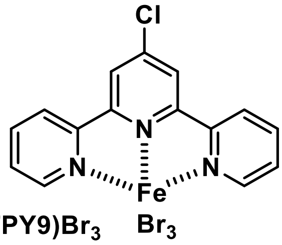

Fe(TPY9)Br$_3$

Fe(TPY9)Br$_3$ was recrystallized from MeCN as a black solid. (92.4 mg, 82%). M.P. 312-314°C. ^{1}H-NMR (400 MHz, DMSO) δ: 9.53 (brs, 1H), 9.14-8.19 (m, 4H), 8.02 (brs, 2H), 7.60-7.10 (m, 3H). IR 3377.5, 3047.4, 1593.0, 1557.4, 1472.5, 1418.4, 1243.7, 1113.3, 1019.5, 826.2, 787.1, 750.9 cm^{-1}. HRMS (ESI$^+$) (m/z) calcd. for $C_{15}H_{10}Br_2ClFeN_3$ [M-Br]$^+$ 482.8253; found 482.8253. Elemental analysis (%) calcd. for $C_{15}H_{10}Br_3ClFeN_3$: C, 31.99; H, 1.79; N, 7.46; found: C, 31.85; H, 1.80; N, 7.53. μ_{eff} = 4.66 μ_{B} (D_2O, 300K, Evans method).

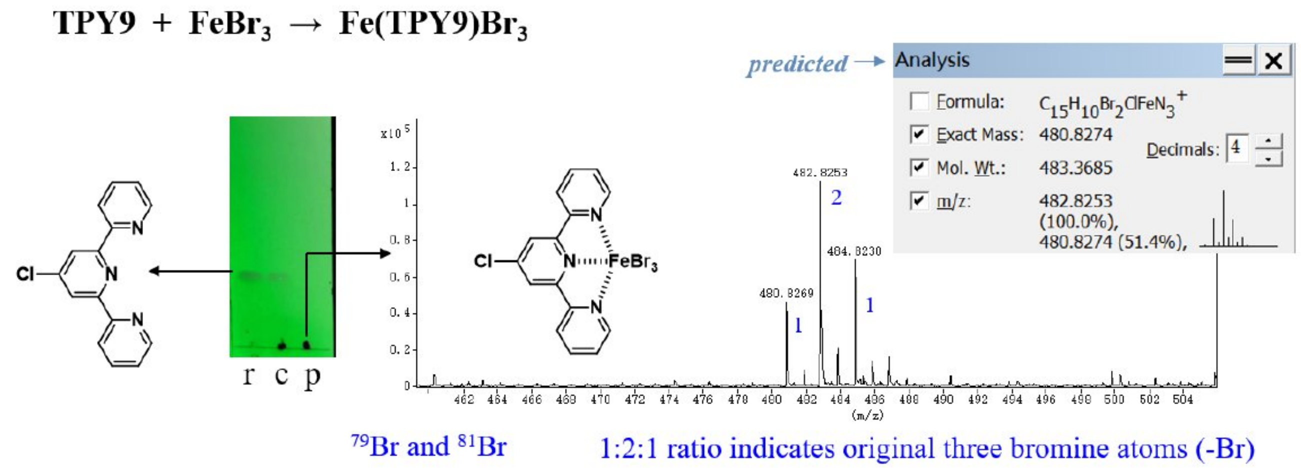

Fe[4'-(methoxy)-2,2':6',2"-terpyridine]Br$_3$ [Fe(TPY10)Br$_3$]

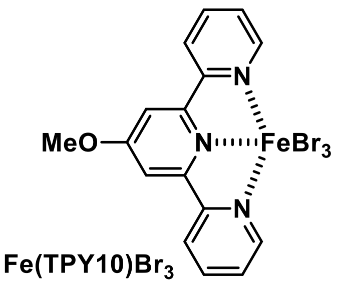

Fe(TPY10)Br$_3$ was recrystallized from MeCN as a black solid. (99.5 mg, 89%). And the yield was almost identical (90%, 2.52 g) when large-scale reaction (**TPY10**: 5 mmol) was carried out. ^{1}H-NMR (400 MHz, DMSO) δ: 8.93 (d, J = 28.2 Hz, 4H), 7.97 (brs, 2H), 7.22 (brs, 4H), 4.39 (brs, 3H). ^{13}C-NMR (100 MHz, DMSO) δ: 170.2, 160.0, 157.6, 152.8, 138.3, 127.3, 123.7, 111.3, 57.7. IR 3373.4, 3056.5, 1605.5, 1559.3, 1475.1, 1436.8, 1408.12, 1221.9, 1157.6, 1024.9, 853.4, 792.7, 755.3 cm^{-1}. HRMS (ESI$^+$) (m/z) calcd. for $C_{16}H_{13}Br_2FeN_3O$ [M-Br]$^+$ 478.8749; found 478.8749. Elemental analysis (%) calcd. for $C_{16}H_{13}Br_3FeN_3O$: C, 34.39; H, 2.34; N, 7.52; found: C, 34.47; H, 2.36; N, 7.44. μ_{eff} = 4.96 μ_B (DMSO-d_6, 300K, Evans method).

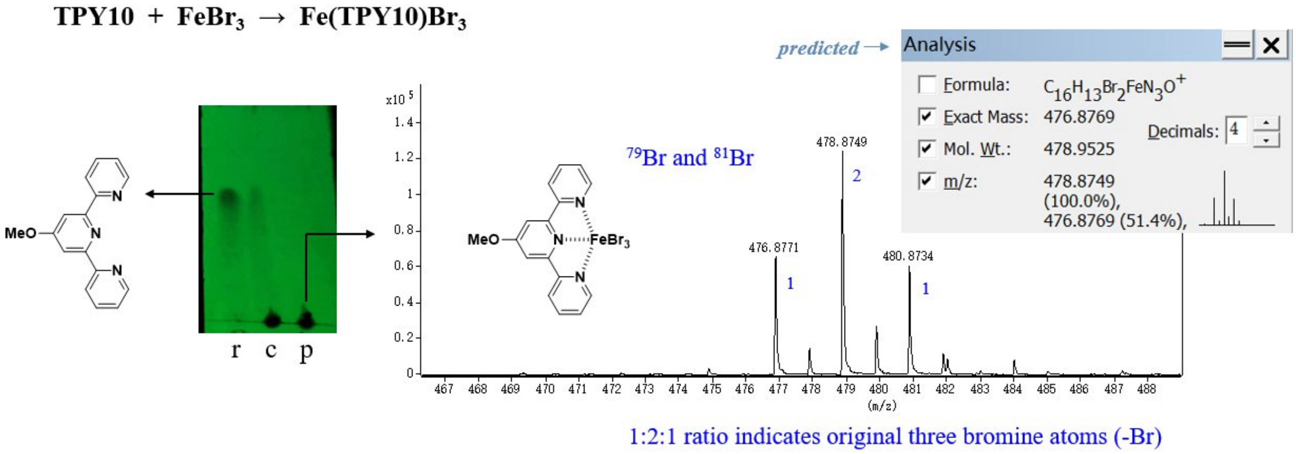

Fe[4'-(2-furyl)-2,2':6',2"-terpyridine]Br$_3$ [Fe(LO)Br$_3$]

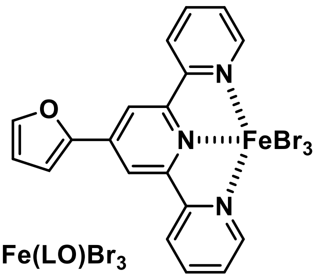

Fe(LO)Br$_3$

Fe(LO)Br$_3$ was recrystallized from MeCN as a black solid (101 mg, 85%). M.P. 314-316°C. IR 3050.8, 3019.6, 1606.5, 1543.1, 1480.1, 1429.6, 1251.6, 1152.6, 1081.3, 1018.2, 883.0, 788.9, 748.7 cm^{-1}. HRMS (ESI$^+$) (m/z) calcd. for $C_{19}H_{13}Br_2FeN_3O$ [M-Br]$^+$ 514.8749; found 514.8758. Elemental analysis (%) calcd. for $C_{19}H_{13}Br_3FeN_3O$: C, 38.36; H, 2.20; N, 7.06; found: C, 38.50; H, 2.27; N, 7.11. μ_{eff} = 2.19 μ_B (DMSO-d_6, 300K, Evans method).

Fe[4'-(2-thiophenyl)-2,2':6',2"-terpyridine]Br$_3$ [Fe(LS)Br$_3$]

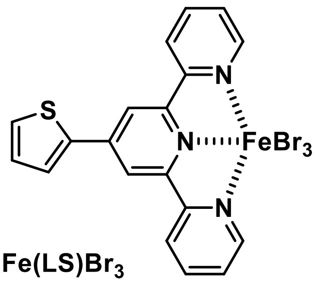

Fe(LS)Br$_3$

Fe(LS)Br$_3$ was recrystallized from MeCN as a black solid (103 mg, 84%). M.P. > 350°C. ^{1}H-NMR (400 MHz, DMSO) δ: 9.51 (s, 2H), 9.07 (d, J = 7.9 Hz, 2H), 8.58 (s, 1H), 8.06 (d, J = 25.3 Hz, 3H), 7.56–7.48 (m, 1H), 7.35–7.10 (m, 4H). IR 3666.4, 3392.9, 2979.7, 2903.1, 1603.8, 1529.2, 1469.0, 1424.6, 1245.0, 1054.8, 1026.5, 785.8, 750.2, 710.0 cm^{-1}. HRMS (ESI$^+$) (m/z) calcd. for $C_{19}H_{13}Br_2FeN_3S$ [M-Br]$^+$ 530.8520; found 530.8514. Elemental analysis (%) calcd. for $C_{19}H_{13}Br_3FeN_3S$: C, 37.35; H, 2.14; N, 6.88; found: C, 37.41; H, 2.10; N, 6.93. μ_{eff} = 2.64 μ_B (DMSO-d_6, 300K, Evans method).

2.4 Characterization of Br-MCD

Synthesis and characterization of Br-MCD: To a stirred solution of MCD (40 mg, 0.23 mmol), NH_4Br (108 mg, 1.1 mmol) and Fe(TPY10)Br$_3$ (5.6 mg, 0.01 mmol) in MeCN/H_2O (3/1, 30 mL) was added H_2O_2 (30%, 110 µL, 1.1 mmol) dropwise. After completion of the addition, the mixture was stirred vigorously at rt for 1 h, followed by the addition of ethyl acetate (100 mL), hexane (50 mL) and H_2O (100 mL). The organic fractions were collected, and the aqueous phase was extracted with ethyl acetate/hexane (1/1, 2 ×30 mL). The combined organic fractions were washed with H_2O, dried over Na_2SO_4, filtered, and concentrated under reduced pressure. Notably, Br-MCD was unstable for long-time and high temperature concentration, and the column chromatography should be quick.

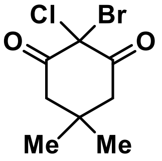

Br-MCD

Br-MCD was purified by flash column chromatography (ethyl acetate/hexane = 1:10 to 1:5) as a white solid (44.3 mg, 76%). M.P. 124-126°C. ^{1}H-NMR (400 MHz, $CDCl_3$) δ: 3.38 (dd, J = 14.6 Hz, J = 1.0 Hz, 2H), 2.65 (d, J = 14.6 Hz, 2H), 1.19 (s, 3H), 0.85 (s, 3H). ^{13}C-NMR (100 MHz, $CDCl_3$) δ: 192.7, 74.2, 48.9, 30.8, 29.8, 26.1. IR 2920.3, 2851.1, 1715.1, 1639.0, 1459.2, 1419.7, 1312.5, 1198.3, 1061.6, 998.7, 724.3, 614.0, 571.4 cm^{-1}. HRMS (ESI$^+$) (m/z) calcd. for $C_8H_{11}BrClO_2$ [M+H]$^+$ 252.9625; found 252.9628.

3. Catalytic efficiency of $Fe(TPY10)Br_3$ for in-situ Br^+ generation (bromination of phenol red) versus stripped $FeBr_3$.

General Procedure: To a stirred solution of phenol red (10 mg, 28 μmol) in MeCN/H_2O (3/1, 10 mL) were added catalyst [no catalyst, $FeBr_3$ (1.66 mg, 5.6 μmol) or $Fe(TPY10)Br_3$ (3.13 mg, 5.6 μmol)], bromide source: NH_4Br (118 mg, 1.2 mmol), and oxidant: H_2O_2 (30%, 0.12 mL, 1.2 mmol). After completion of the addition, the homogenous mixture was stirred vigorously at rt for 0.5 h. Then, 0.3 mL of the above residue was taken out, diluted by MeCN/H_2O (3/1, 5.7 mL) and analyzed by UV-Vis spectroscopy immediately. Notably, the interference of iron catalyst $Fe(TPY10)Br_3$ for UV-Vis spectrum was excluded: UV-Vis absorptions of iron complex [$Fe(TPY10)Br_3$ (28 µM) in MeCN/H_2O (3/1)] were subtracted simultaneously for the spectra provided in this paper, and 28 µM was identical to the final concentration of diluted iron catalyst following the above procedure.

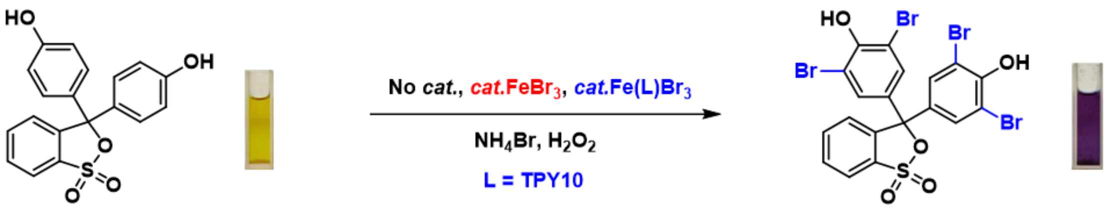

Phenol red λ_{max} = 429 nm Bromophenol blue λ_{max} = 598 nm

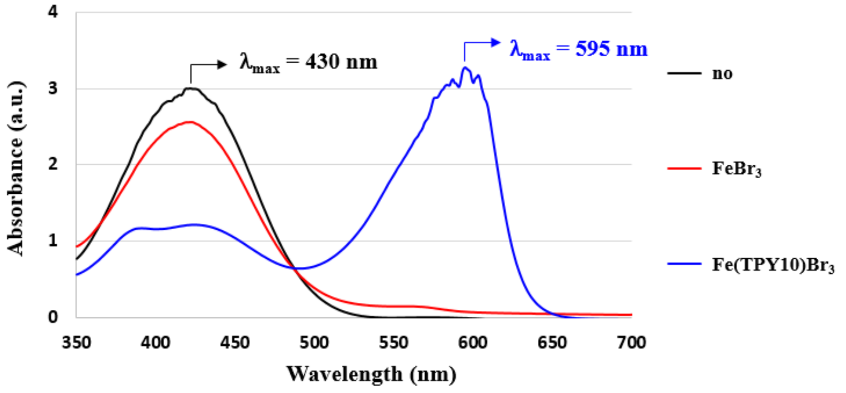

For the reaction catalyzed by $Fe(TPY10)Br_3$, HRMS of the reaction mixture showed the molecular ion peaks at m/e 666, 668, 670, 672, and 674 in a ratio of 1:4:6:4:1, indicating the presence of four bromine atoms (^{79}Br and ^{81}Br are equally abundant in the nature).

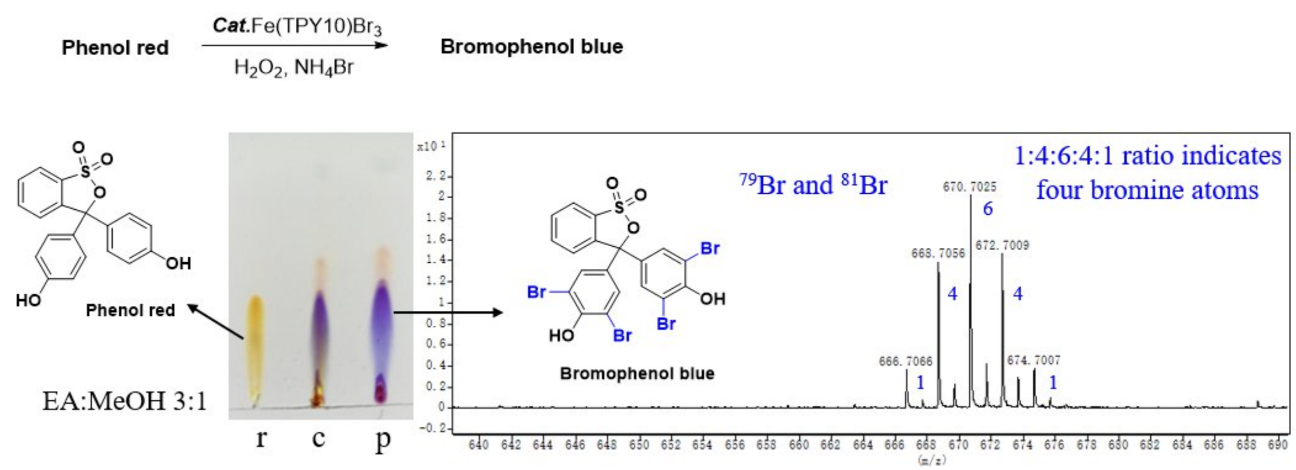

4. The physical property and stability comparison between Fe(TPY10)Br_3 and stripped $FeBr_3$.

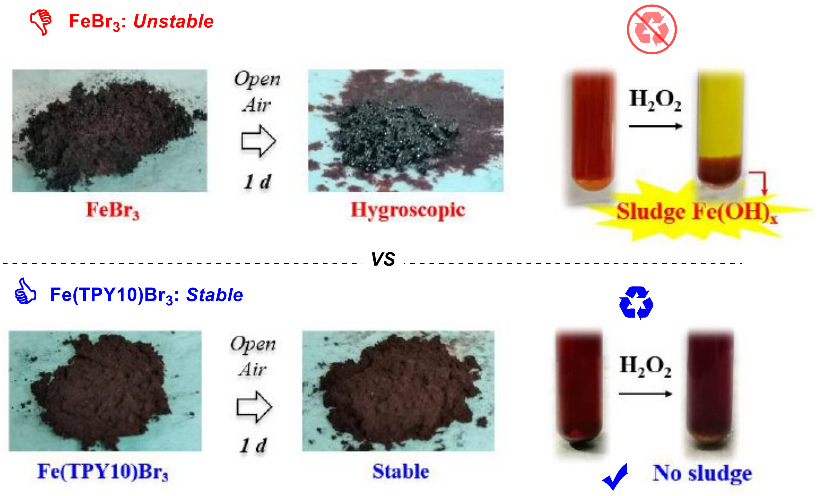

$FeBr_3$ (10 mg) or Fe(TPY10)Br_3 (10 mg) was stored under open-air condition (temperature: 25°C; relative humidity: 50%) for 1 d, and the representative statuses were listed in above figure. The results showed that $FeBr_3$ is very hygroscopic with lots of deliquescence on the paper, whereas Fe(TPY10)Br_3 is very stable with almost no change during the process.

The turnover status of above catalysts ($FeBr_3$ and Fe(TPY10)Br_3) in the presence of H_2O_2 and NH_4Br were also investigated as follows: to a stirred solution of catalyst: $FeBr_3$ (10 mg, 0.034 mmol) or Fe(TPY10)Br_3 (10 mg, 0.018 mmol) in MeCN (7 mL) were added NH_4Br (160 mg, 1.63 mmol) and H_2O_2 (30%, 0.17 mL, 1.7 mmol). After completion of the addition, the reaction mixture was stirred for 10 min at rt. And the results showed that lots of sludges $Fe(OH)_3$/Fe_2O_3 were precipitated out of the solution for $FeBr_3$, which is detrimental for the recycling, stability and turnover number of $FeBr_3$, whereas Fe(TPY10)Br_3 is always homogenous throughout the process (even after overnight stirring). Therefore, coordinated complex Fe(TPY10)Br_3 was a more efficient catalyst versus bare $FeBr_3$.

5. Application of *cat.*Fe(TPY10)Br_3 for the bromination of aromatic ring.

5.1 Optimization for the bromination of aromatic ring.

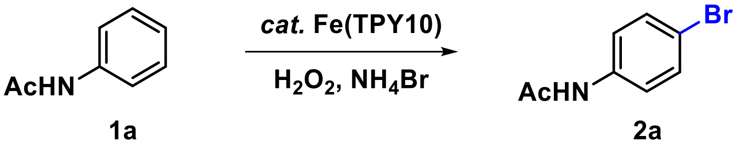

Screening Procedure: To a stirred solution of **1a** (27 mg, 0.2 mmol), NH_4Br (58.7 mg, 0.6 mmol) and *cat.*Fe(TPY10)Br_3 (11.2 mg, 0.02 mmol) in different solvent (2 mL) was added H_2O_2 (30%, 60 μL, 0.6 mmol) dropwise. After completion of the addition, the reaction mixture was allowed to stir at room temperature (rt) for 1 h. Then, additional NH_4Br (58.7 mg, 0.6 mmol) and H_2O_2 (30%, 60 μL, 0.6 mmol) were added, and the resulting mixture was continuously stirred at rt for another 0.5 h. The reaction was quenched by dilute aqueous $Na_2S_2O_3$ solution (0.1 M, 20 mL) and ethyl acetate (20 mL). The organic fractions were collected, and the aqueous phase was extracted with ethyl acetate (2 ×10 mL). The combined organic fractions were washed with brine, dried over Na_2SO_4, filtered, and concentrated under reduced pressure. The crude residue was eluted by hexane/ethyl acetate (1:1) through a thin layer of silica gel to remove Fe(TPY10)Br_3, which was interferential for the ^{1}H-NMR analysis.

Table S2. Optimization for the bromination of **1a**

entry	*Catalyst (0.1 eq)*	*Solvent (v/v)*	*Yield[a] (%)*
1	Fe(TPY10)Br_3	DMF/H_2O (3/1)	23
2	Fe(TPY10)Br_3	DCM/H_2O (3/1)	31
3	Fe(TPY10)Br_3	*t*-BuOH/H_2O (3/1)	48
4	Fe(TPY10)Br_3	THF/H_2O (3/1)	55
5	Fe(TPY10)Br_3	Acetone/H_2O (3/1)	74
6	Fe(TPY10)Br_3	MeCN/H_2O (3/1)	87
**7**	**Fe(TPY10)Br_3**	**EtOH/H_2O (3/1)**	**89**
8	$FeBr_3$	EtOH/H_2O (3/1)	26
9	No catalyst	EtOH/H_2O (3/1)	trace
10	Fe(TPY10)Br_3	EtOH	52
11	Fe(TPY10)Br_3	H_2O	30

[a]Yield was determined by ^{1}H-NMR analysis of the crude reaction mixture using CH_2Br_2 as the internal reference.

5.2 Detail information for the bromination of aromatic ring.

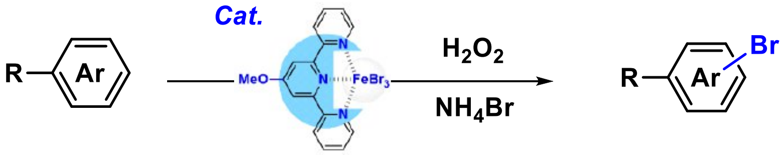

General Procedure I: To a stirred solution of aromatic ring (1 mmol), NH_4Br (294 mg, 3 mmol) and Fe(TPY10)Br_3 (55.8 mg, 0.1 mmol) in EtOH/H_2O (3/1, 10 mL) was added H_2O_2 (30%, 0.3 mL, 3 mmol) dropwise. After completion of the addition, the homogenous mixture was stirred vigorously at rt for 1.5 h. Then, additional NH_4Br (294 mg, 3 mmol) and H_2O_2 (30%, 0.3 mL, 3 mmol) were added, and the resulting mixture was continuously stirred at rt for 1 h. The reaction was quenched by aqueous $Na_2S_2O_3$ solution (0.2 M, 50 mL) and ethyl acetate (50 mL). The organic fractions were collected, and the aqueous phase was extracted with ethyl acetate (2 ×20 mL). The combined organic fractio ns were washed with H_2O, dried over Na_2SO_4, filtered, and concentrated under reduced pressure. Notably, substrate **1q** (Anal. Chem. 2020, 92, 1883) was prepared according to the published literature, and other reagents can be purchased directly from Sigma-Aldrich Co.

***N*-(4-bromophenyl)acetamide (2a)**

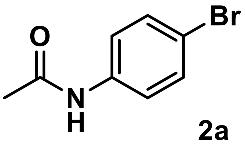

2a

2a was purified by flash column chromatography (hexane/ethyl acetate = 5:1 to 2:1) as a white solid (191 mg, 89%). M.P. 168-169°C. ^{1}H-NMR (400 MHz, $CDCl_3$) δ: 7.46-7.36 (m, 4H), 2.17 (s, 3H). ^{13}C-NMR (100 MHz, $CDCl_3$) δ: 168.3, 137.0, 132.0, 121.4, 116.9, 24.6. IR 3287.4, 3178.5, 1661.9, 1589.1, 1476.2, 1380.2, 1296.2, 1243.1, 816.2, 733.0, 496.3 cm^{-1}. HRMS (ESI$^+$) (m/z) calcd. for C_8H_9BrNO $[M+H]^+$ 213.9862; found 213.9861.

4-Bromo-*N,N*-dimethylaniline (2b)

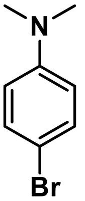

2b

2b was purified by flash column chromatography (hexane to hexane/ethyl acetate = 30:1) as a white solid (162 mg, 81%). M.P. 52-54°C. ^{1}H-NMR (400 MHz, $CDCl_3$) δ: 7.29 (d, *J* = 9.0 Hz, 2H), 6.58 (d, *J* = 9.0 Hz, 2H), 2.91 (s, 6H). ^{13}C-NMR (100 MHz, $CDCl_3$) δ: 149.5, 131.7, 114.1, 108.5, 40.6. IR 3084.6, 2887.5, 2800.4, 1580.8, 1484.6, 1339.9, 1224.3, 1061.5, 798.1, 504.2 cm^{-1}. HRMS (ESI$^+$) (m/z) calcd. for $C_8H_{11}BrN$ $[M+H]^+$ 200.0069; found 200.0075.

2-Bromo-4-nitroaniline (2c)

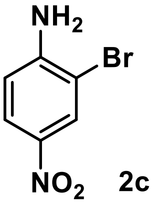

2c

2c was purified by flash column chromatography (hexane/ethyl acetate = 3:1 to 1:1) as a yellow solid (180 mg, 83%). M.P. 104-105°C. ^{1}H-NMR (400 MHz, $CDCl_3$) δ: 8.35 (d, *J* = 2.5 Hz, 1H), 8.01 (dd, *J* = 8.9, 2.5 Hz, 1H), 6.74 (d, *J* = 9.0 Hz, 1H), 4.87 (brs, 2H). ^{13}C-NMR (100 MHz, $CDCl_3$) δ: 149.9, 139.3, 129.4, 125.1, 113.6, 107.2. IR 3480.8, 3368.1, 1616.7, 1578.8, 1478.0, 1299.1, 1256.0, 1115.7, 818.2, 743.2 cm^{-1}. HRMS (ESI$^+$) (m/z) calcd. for $C_6H_6BrN_2O_2$ $[M+H]^+$ 216.9607; found 216.9604.

2-(4-Bromophenoxy)ethanol (2d)

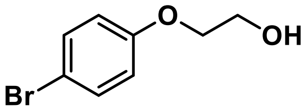

2d

$FeBr_3$ (29.6 mg, 0.1 mmol) was used as the control catalyst following **General Procedure I**. **2d** was purified by flash column chromatography (hexane/ethyl acetate = 10:1 to 3:1) as a light yellow oil [Fe(TPY10)Br_3: 195 mg, 90%; $FeBr_3$: 60.8 mg, 28%]. ^{1}H-NMR (400 MHz, $CDCl_3$) δ: 7.37 (d, J = 8.9 Hz, 2H), 6.79 (d, J = 8.9 Hz, 2H), 4.04 (dd, J = 5.2, 3.7 Hz, 2H), 3.95 (dd, J = 5.2, 3.7 Hz, 2H), 2.15 (brs, 1H). ^{13}C-NMR (100 MHz, $CDCl_3$) δ: 157.9, 132.5, 116.5, 113.4, 69.6, 61.5. IR 3357.7, 2931.4, 2873.8, 1587.2, 1525.4, 1241.3, 1070.8, 908.4, 821.3, 640.4, 507.0 cm^{-1}. HRMS (ESI$^+$) (m/z) calcd. for $C_8H_{10}BrO_2$ [M+H]$^+$ 216.9859; found 216.9855.

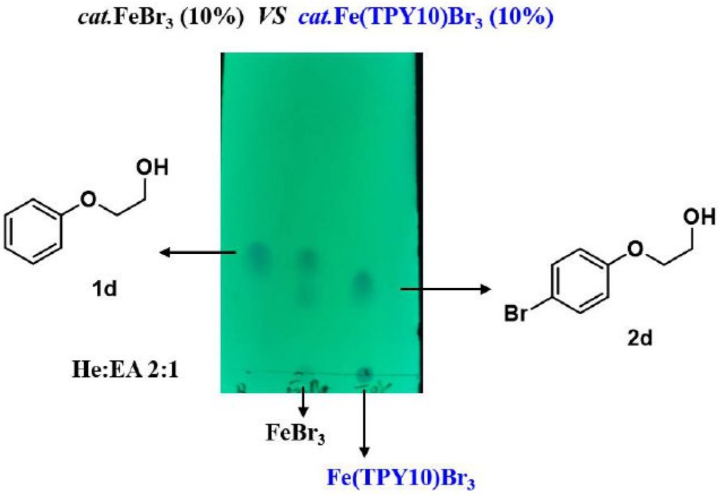

4-bromoaniline (2e)

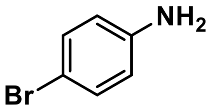

2e

2e was purified by flash column chromatography (hexane/ethyl acetate = 5:1 to 3:1) as a white solid (138 mg, 80%). M.P. 61-63 °C. ^{1}H-NMR (400 MHz, $CDCl_3$) δ: 7.26 (d, J = 8.7 Hz, 2H), 6.58 (d, J = 8.7 Hz, 2H), 3.66 (s, 2H). ^{13}C-NMR (100 MHz, $CDCl_3$) δ: 145.5, 132.0, 116.7, 110.2. IR 3464.0, 3373.5, 1874.9, 1605.6, 1482.9, 1276.4, 1176.5, 1118.5, 1065.7, 814.9cm^{-1}. HRMS(ESI$^+$) (m/z) calcd. for C_6H_7BrN [M+H]$^+$ 171.9756; found 171.9759.

1-Bromo-2,4-dimethoxybenzene (2f)

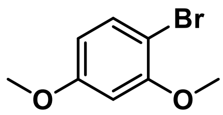

2f

Only Fe(TPY10)Br_3 (27.9 mg, 0.05 mmol), NH_4Br (294 mg, 3 mmol) and H_2O_2 (30%, 0.3 mL, 3 mmol) were needed and the reaction time was 1 h. **2f** was purified by flash column chromatography (hexane to hexane/ethyl acetate = 5:1) as a light yellow oil (182 mg, 84%). ^{1}H-NMR (400 MHz, $CDCl_3$) δ: 7.39 (d, J = 8.7 Hz, 1H), 6.47 (d, J = 2.7 Hz, 1H), 6.38 (dd, J = 8.8, 2.7 Hz, 1H), 3.85 (s, 3H), 3.78 (s, 3H). ^{13}C-NMR (100 MHz, $CDCl_3$) δ: 160.3, 156.6, 133.2, 106.0, 102.4, 100.0, 56.2, 55.6. IR 3003.8, 2939.6, 2836.7, 1585.0, 1483.8, 1458.5, 1206.0, 1159.4, 1024.6, 825.0, 607.6 cm^{-1}. HRMS (ESI$^+$) (m/z) calcd. for $C_8H_{10}BrO_2$ [M+H]$^+$ 216.9859; found 216.9863

2-Bromo-1,3,5-trimethoxybenzene (2g)

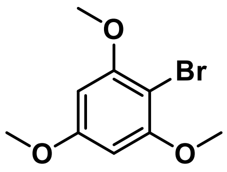

2g

Only $Fe(TPY10)Br_3$ (27.9 mg, 0.05 mmol), NH_4Br (294 mg, 3 mmol) and H_2O_2 (30%, 0.3 mL, 3 mmol) were needed and the reaction time was 1 h. **2g** was purified by flash column chromatography (hexane to hexane/ethyl acetate = 5:1) as a white solid (235 mg, 95%). M.P. 92-93°C. ^{1}H-NMR (400 MHz, $CDCl_3$) δ: 6.12 (s, 2H), 3.83 (s, 6H), 3.77 (s, 3H). ^{13}C-NMR (100 MHz, $CDCl_3$) δ: 160.5, 157.4, 91.8, 91.6, 56.3, 55.5. IR 3003.0, 2940.1, 2840.9, 1582.5, 1457.7, 1409.4, 1157.5, 1117.0, 805.7, 665.4, 629.4 cm^{-1}. HRMS (ESI$^+$) (m/z) calcd. for $C_9H_{11}BrNaO_3$ $[M+Na]^+$ 268.9784; found 268.9781.

1-Bromo-4-methoxynaphthalene (2h)

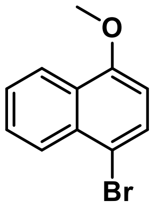

2h

2h was purified by flash column chromatography (hexane to hexane/ethyl acetate = 10:1) as a light yellow oil (209 mg, 88%). ^{1}H-NMR (400 MHz, $CDCl_3$) δ: 8.31 (dd, J = 8.4, 0.8 Hz, 1H), 8.21 (dd, J = 8.5, 0.9 Hz, 1H), 7.67 (d, J = 8.3 Hz, 1H), 7.64 (ddd, J = 8.4, 6.8, 1.3 Hz, 1H), 7.55 (ddd, J = 8.2, 6.8, 1.3 Hz, 1H), 6.65 (d, J = 8.2 Hz, 1H), 3.97 (s, 3H). ^{13}C-NMR (100 MHz, $CDCl_3$) δ: 155.2, 132.4, 129.5, 127.8, 126.9, 126.8, 126.0, 122.5, 113.2, 104.5, 55.6. IR 3066.2, 2944.1, 2838.5, 1584.5, 1452.6, 1370.6, 1247.3, 1083.6, 904.8, 805.9, 760.5, 624.1 cm^{-1}. HRMS (ESI$^+$) (m/z) calcd. for $C_{11}H_{10}BrO$ $[M+H]^+$ 236.9910; found 236.9908.

2-(5-bromothiophen-2-yl)acetonitrile (2i)

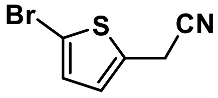

2i

2i was purified by flash column chromatography (hexane to hexane/ethyl acetate = 5:1) as a colorless oil (174 mg, 86%). ^{1}H-NMR (400 MHz, $CDCl_3$) δ: 6.94 (d, J = 3.8 Hz, 1H), 6.83 (d, J = 3.8 Hz, 1H), 3.83 (s, 2H). ^{13}C-NMR (100 MHz, $CDCl_3$) δ: 132.3, 130.1, 127.7, 116.3, 112.4, 18.9. IR 3098.1, 2925.9, 2855.2, 2254.2, 1542.7, 1478.0, 1051.4, 968.9, 797.4, 458.3 cm^{-1}. HRMS (ESI$^+$) (m/z) calcd. for C_6H_5BrNS $[M+H]^+$ 201.9321; found 201.9330.

2-Bromo-3,4-dimethoxythiophene (2j)

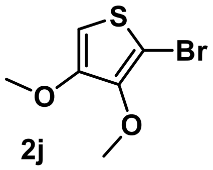

2j

Only $Fe(TPY10)Br_3$ (27.9 mg, 0.05 mmol), NH_4Br (294 mg, 3 mmol) and H_2O_2 (30%, 0.3 mL, 3 mmol) were needed and the reaction time was 1 h. **2j** was purified by flash column chromatography (hexane/ethyl acetate = 10:1 to 3:1) as a light yellow oil (141 mg, 63%). ^{1}H-NMR (400 MHz, $CDCl_3$) δ: 6.15 (s, 1H), 3.91 (s, 3H), 3.82 (s, 3H). ^{13}C-NMR (100 MHz, $CDCl_3$) δ: 150.1, 145.8, 97.2, 96.3, 60.8, 57.3. IR 3113.1, 3003.4, 2937.0, 2831.8, 1497.2, 1382.1, 1202.9, 1151.1, 1012.0, 865.7, 680.8 cm^{-1}. HRMS (ESI$^+$) (m/z) calcd. for $C_6H_8BrO_2S$ $[M+H]^+$ 222.9423; found 222.9433.

Ethyl 4-Bromo-3,5-dimethyl-1*H*-pyrrole-2-carboxylate (2k)

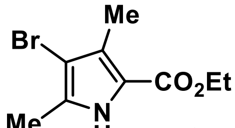

2k

$FeBr_3$ (29.6 mg, 0.1 mmol) was used as the control catalyst following **General Procedure I**. **2k** was purified by flash column chromatography (hexane to hexane/ethyl acetate = 10:1 to 2:1) as a light pink solid [Fe(TPY10)Br_3: 234 mg, 95%; $FeBr_3$: 56.6 mg, 23%]. M.P. 144-146°C. ^{1}H-NMR (400 MHz, DMSO) δ: 11.74 (s, 1H), 4.22 (q, J = 7.1 Hz, 2H), 2.19 (s, 3H), 2.16 (s, 3H), 1.28 (t, J = 7.1 Hz, 3H). ^{13}C-NMR (100 MHz, DMSO) δ: 160.7, 131.5, 125.9, 117.2, 99.6, 59.8, 14.9, 12.08, 12.00. IR 3291.8, 2979.9, 2910.0, 1664.0, 1433.2, 1374.7, 1267.0, 1104.1, 762.4, 690.0, 573.7 cm^{-1}. HRMS (ESI$^+$) (m/z) calcd. for $C_9H_{13}BrNO_2$ [M+H]$^+$ 246.0124; found 246.0131.

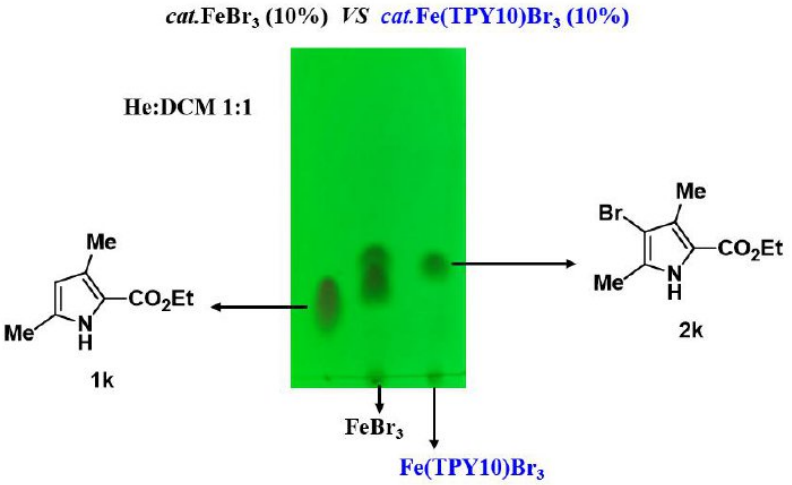

4-Bromo-2-phenyl-1*H*-imidazole (2l)

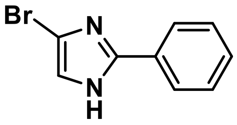

2l

Only Fe(TPY10)Br_3 (27.9 mg, 0.05 mmol), NH_4Br (294 mg, 3 mmol) and H_2O_2 (30%, 0.3 mL, 3 mmol) were needed and the reaction time was 1 h. **2l** was purified by flash column chromatography (hexane to hexane/ethyl acetate = 10:1 to 2:1) as a white solid (136 mg, 61%). M.P. 206-208°C. ^{1}H-NMR (400 MHz, DMSO) δ: 12.89 (brs, 1H), 7.91-7.88 (m, 2H), 7.50-7.41 (m, 3H), 7.42 (d, J = 2.0 Hz, 1H). ^{13}C NMR (100 MHz, DMSO) δ: 145.8, 129.7, 128.8, 128.6, 124.8, 117.3, 114.8. IR 3382.0, 3147.1, 1641.9, 1553.6, 1455.4, 1393.5, 1266.6, 1118.3, 770.1, 696.0, 515.6 cm^{-1}. HRMS (ESI$^+$) (m/z) calcd. for $C_9H_8BrN_2$ [M+H]$^+$ 222.9865; found 222.9859.

3-Bromo-2,6-dimethoxypyridine (2m)

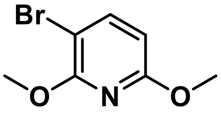

2m

Additional Fe(TPY10)Br_3 (27.9 mg, 0.05 mmol), NH_4Br (294 mg, 3 mmol) and H_2O_2 (30%, 0.3 mL, 3 mmol) were needed. **2m** was purified by flash column chromatography (hexane/ethyl acetate = 10:1 to 3:1) as a light yellow oil (168 mg, 77%). ^{1}H-NMR (400 MHz, $CDCl_3$) δ: 7.63 (d, J = 8.3 Hz, 1H), 6.23 (d, J = 8.2 Hz, 1H), 4.00 (s, 3H), 3.90 (s, 3H). ^{13}C-NMR (100 MHz, $CDCl_3$) δ 162.2, 158.6, 143.8, 102.8, 95.5, 54.4, 53.9. IR 2947.4, 2860.8, 1575.7, 1457.7, 1377.4, 1310.7, 1016.8, 803.0, 734.3, 678.9, 506.7 cm^{-1}. HRMS (ESI$^+$) (m/z) calcd. for $C_7H_9BrNO_2$ [M+H]$^+$ 217.9811; found 217.9816.

5-Bromo-2,3-dihydrobenzofuran (2n)

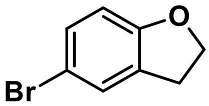

2n

2n was purified by flash column chromatography (hexane to hexane/ethyl acetate = 20:1) as a white solid (177 mg, 89%). M.P. 50-52°C. ^{1}H-NMR (400 MHz, $CDCl_3$) δ: 7.28-7.25 (m, 1H), 7.20-7.15 (m, 1H), 6.66 (d, J = 8.4 Hz, 1H), 4.57 (t, J = 8.7 Hz, 2H), 3.20 (t, J = 8.7 Hz, 2H). ^{13}C-NMR (100 MHz, $CDCl_3$) δ: 159.3, 130.7, 129.5, 127.9, 112.1, 110.9, 71.6, 29.7. IR 3042.1, 2977.5, 2896.2, 1463.3, 1417.6, 1222.2, 1144.6, 1093.1, 924.2, 893.3, 634.9, 537.5 cm^{-1}. HRMS (ESI$^+$) (m/z) calcd. for C_8H_8BrO [M+H]$^+$ 198.9753; found 198.9758.

3-bromobenzo[b]thiophene (2o)

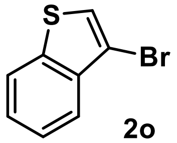

2o was purified by flash column chromatography (hexane) as a light yellow oil (145 mg, 68%). ^{1}H-NMR (400 MHz, $CDCl_3$) δ: 7.83-7.76 (m, 2H), 7.42 (ddd, J = 8.1, 7.1, 1.2 Hz, 1H), 7.39-7.33 (m, 2H). ^{13}C-NMR (100 MHz, $CDCl_3$) δ: 138.6, 137.6, 125.4, 125.1, 123.6, 123.1, 122.8, 107.8. IR 3103.6, 3058.2, 1527.8, 1458.6, 1314.3, 1249.1, 926.2, 817.5, 735.8, 712.3, 495.0 cm^{-1}. HRMS (ESI$^+$) (m/z) calcd. for C_8H_6BrS [M+H]$^+$ 212.9368; found 212.9365.

3-Bromo-9*H*-carbazole (2p)

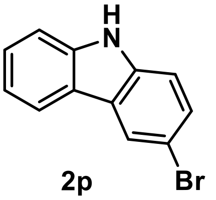

Acetone/H_2O (3/1, 10 mL) was used as the solvent according to **General Procedure I**. **2p** was purified by flash column chromatography (hexane/ethyl acetate = 10:1 to 3:1) as a white solid (182 mg, 74%). M.P. 201-203°C. ^{1}H-NMR (400 MHz, DMSO) δ: 11.44 (s, 1H), 8.36 (d, J = 2.0 Hz, 1H), 8.17 (d, J = 7.8 Hz, 1H), 7.55-7.39 (m, 4H), 7.18 (t, J = 7.4 Hz, 1H). ^{13}C-NMR (100 MHz, DMSO) δ: 140.1, 138.4, 127.8, 126.3, 124.4, 122.7, 121.4, 120.7, 118.9, 112.9, 111.2, 110.5. IR 3395.8, 1564.8, 1432.4, 1321.0, 1101.2, 1409.4, 1042.5, 921.8, 803.3, 713.2, 567.1 cm^{-1}. HRMS (ESI$^+$) (m/z) calcd. for $C_{12}H_9BrN$ [M+H]$^+$ 245.9913; found 245.9918.

6-Bromo-4-phenyl-2-(trifluoromethyl)quinolin-7-amine (2q)

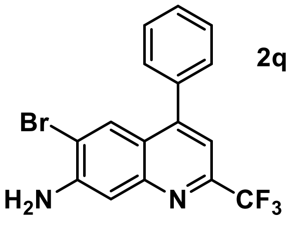

2q was purified by flash column chromatography (hexane to hexane/ethyl acetate = 10:1 to 3:1) as a light yellow solid (319 mg, 87%). M.P. 162-163°C. ^{1}H-NMR (400 MHz, $CDCl_3$) δ: 8.35–8.30 (m, 2H), 8.01 (s, 1H), 7.93-7.87 (m, 1H), 7.61-7.49 (m, 3H), 7.12 (d, J = 9.0 Hz, 1H), 4.71 (brs, 2H). ^{13}C-NMR (100 MHz, $CDCl_3$) δ: 157.0, 147.2, 146.3, 138.3, 135.0 (q, $^2J_{C-F}$ = 31.5 Hz), 130.2, 129.0, 127.5, 123.6 (q, $^1J_{C-F}$ = 275.9 Hz), 122.2 (q, $^4J_{C-F}$ = 2.5 Hz), 119.0, 116.4, 112.2 (q, $^3J_{C-F}$ = 5.3 Hz), 106.1. ^{19}F-NMR (376 MHz, $CDCl_3$) δ: -60.95 (d, $^4J_{F-H}$ = 2.0 Hz). IR 3425.7, 3379.0, 3325.9, 1624.6, 1596.2, 1361.8, 1152.7, 1151.1, 1118.6, 818.8, 680.2 cm^{-1}. HRMS (ESI$^+$) (m/z) calcd. for $C_{16}H_{11}BrF_3N_2$ [M+H]$^+$ 367.0052; found 367.0060.

3-Bromo-7-(diethylamino)-4-methyl-2*H*-chromen-2-one (2r)

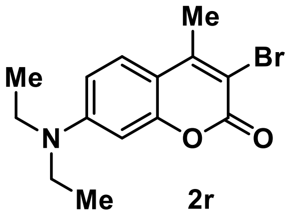

2r

2r was purified by flash column chromatography (hexane/ethyl acetate = 10:1 to 2:1) as a light yellow solid (279 mg, 90%). M.P. 104-106°C. ^{1}H-NMR (400 MHz, $CDCl_3$) δ: 7.40 (d, *J* = 9.1 Hz, 1H), 6.59 (dd, *J* = 9.1, 2.6 Hz, 1H), 6.46 (d, *J* = 2.6 Hz, 1H), 3.40 (q, *J* = 7.1 Hz, 4H), 2.50 (s, 3H), 1.20 (t, *J* = 7.1 Hz, 6H). ^{13}C-NMR (100 MHz, $CDCl_3$) δ: 158.2, 154.5, 151.6, 150.7, 126.2, 109.2, 109.0, 105.7, 97.4, 44.9, 19.2, 12.5. IR 3065.5, 2959.8, 1776.1, 1713.0, 1609.7, 1576.3, 1434.5, 1411.8, 1376.2, 1351.0, 1280.4, 1261.6, 821.8, 800.4, 775.8 cm^{-1}. HRMS (ESI$^+$) (m/z) calcd. for $C_{14}H_{17}BrNO_2$ $[M+H]^+$ 310.0437; found 310.0440.

4-Bromoanisole (2s)

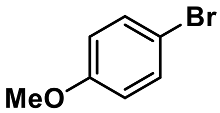

2s

2s was purified by flash column chromatography (hexane/ethyl acetate = 10:1) as a light yellow oil (153 mg, 82%). ^{1}H-NMR (400 MHz, $CDCl_3$) δ: 7.41-7.34 (m, 2H), 6.81-6.75 (m, 2H), 3.78 (s, 3H). ^{13}C-NMR (100 MHz, $CDCl_3$) δ: 158.7, 132.2, 115.7, 112.8, 55.4. IR 3070.4, 2902.3, 2835.3, 1583.6, 1484.8, 1288.1, 1240.7, 1173.9, 1029.2, 817.0, 597.0, 504.3 cm^{-1}. HRMS (ESI$^+$) (m/z) calcd. for C_7H_8BrO $[M+H]^+$ 186.9753; found 186.9757.

4-Bromothiophene (2t)

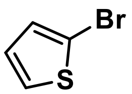

2t

2t was purified by flash column chromatography (hexane to hexane/ethyl acetate = 10:1) as a light yellow oil (109 mg, 67%). ^{1}H-NMR (400 MHz, $CDCl_3$) δ: 7.23 (dd, *J* = 5.6, 1.3 Hz, 1H), 7.06 (dd, *J* = 3.7, 1.4 Hz, 1H), 6.87 (dd, *J* = 5.6, 3.7 Hz, 1H). ^{13}C-NMR (100 MHz, $CDCl_3$) δ: 129.8, 127.6, 127.0, 112.2. IR 3103.8, 2875.2, 1537.4, 1402.1, 1245.8, 1044.0, 970.2, 839.0, 681.5 cm^{-1}. HRMS (ESI$^+$) (m/z) calcd. for C_4H_4BrS $[M+H]^+$ 162.9212; found 162.9216.

4-Bromoestradiol (2aa)

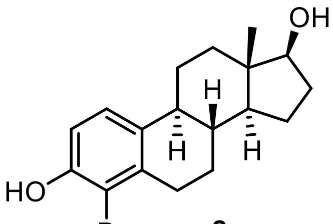

2aa

2aa was purified by flash column chromatography (hexane/ethyl acetate = 5:1) as a light yellow oil (141 mg, 40%). ^{1}H-NMR (400 MHz, DMSO) δ: 9.78 (s, 1H), 7.16-7.02 (m, 1H), 6.75 (d, *J* = 8.5 Hz, 1H), 4.47 (d, *J* = 4.8 Hz, 1H), 3.52 (td, *J* = 8.5, 4.8 Hz, 1H), 2.81 (dd, *J* = 18.1, 4.6 Hz, 1H), 2.63-2.52 (m, 1H), 2.24 (dd, *J* = 13.4, 3.7 Hz, 1H), 2.09 (d, *J* = 9.7 Hz, 1H), 1.86 (qt, *J* = 12.6, 3.7 Hz, 3H), 1.63-1.52 (m, 1H), 1.43-1.03 (m, 7H), 0.65 (s, 3H). ^{13}C-NMR (100 MHz, DMSO) δ: 152.2, 136.8, 133.2, 125.4, 113.6, 113.0, 80.5, 49.9, 44.1, 43.2, 38.2, 37.0, 31.3, 30.4, 27.5, 26.7, 23.2, 11.7. IR 3443.9, 3251.9, 2922.3, 2859.4, 1601.1, 1562.3, 1469.5, 1325.5, 1050.5, 821.0, 795.1, 609.0 cm^{-1}. HRMS (ESI$^+$) (m/z) calcd. for $C_{18}H_{24}BrO_2$ $[M+H]^+$ 351.0954; found 351.0955.

6. Application of *cat.*Fe(TPY10)Br$_3$ for the decarboxylative bromination of cinnamic acid.

6.1 Reagent preparation

Substrate **3m** (Biorg. Med. Chem. 2013, 21, 7107) was prepared according to the published literature, and other reagents can be purchased directly from Sigma-Aldrich Co.

6.2 Detail information for the decarboxylative bromination of cinnamic acid

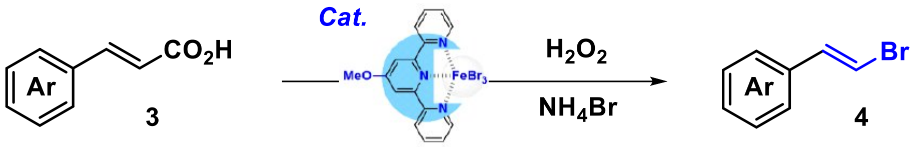

General Procedure II: To a stirred solution of α,β-unsaturated carboxylic acid **3** (1 mmol), NH_4Br (294 mg, 3 mmol) and Fe(TPY10)Br_3 (55.8 mg, 0.1 mmol) in $MeCN/H_2O$ (3/1, 10 mL) was added H_2O_2 (30%, 0.3 mL, 3 mmol) dropwise. After completion of the addition, the homogenous mixture was stirred vigorously at rt for 0.5 h. Then reaction was quenched by aqueous $Na_2S_2O_3$ solution (0.1 M, 50 mL) and ethyl acetate (50 mL). The organic fractions were collected, and the aqueous phase was extracted with ethyl acetate (2 × 20 mL). The combined organic fractions were washed with H_2O, dried over Na_2SO_4, filtered, and concentrated under reduced pressure.

(*E*)-β-Bromo-4-hydroxystyrene (4a)

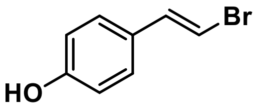

4a

4a was purified by flash column chromatography (ethyl acetate/hexane = 1:10 to 1:3) as a white solid (167 mg, 84%). M.P. 68-70°C. ^{1}H-NMR (400 MHz, $CDCl_3$) δ: 7.19 (d, *J* = 8.6 Hz, 2H), 7.03 (d, *J* = 13.9 Hz, 1H), 6.79 (d, *J* = 8.6 Hz, 2H), 6.61 (d, *J* = 14.0 Hz, 1H), 4.93 (brs, 1H). ^{13}C-NMR (100 MHz, $CDCl_3$) δ: 155.8, 136.6, 129.2, 127.7, 115.8, 104.3. IR 3245.2, 3068.9, 2922.1, 1600.1, 1582.3, 1508.6, 1451.3, 1384.8, 1285.1, 1218.8, 944.8, 928.8, 837.9, 778.7, 507.4 cm^{-1}. HRMS (ESI$^+$) (m/z) calcd. for C_8H_8BrO $[M+H]^+$ 198.9753; found 198.9760.

(*E*)-β-Bromo-2-methoxystyrene (4b)

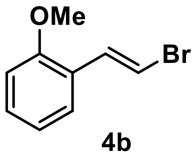

4b

$EtOH/H_2O$ (3/1, 10 mL) was used as the solvent according to **General Procedure II**. **4b** was purified by flash column chromatography (ethyl acetate/hexane = 1:10 to 1:3) as a colorless oil (175 mg, 82%). ^{1}H-NMR (400 MHz, $CDCl_3$) δ: 7.34 (d, *J* = 14.0 Hz, 1H), 7.31-7.27 (m, 2H), 6.98-6.89 (m, 3H), 3.89 (s, 3H). ^{13}C-NMR (100 MHz, $CDCl_3$) δ: 156.7, 133.2, 129.4, 128.1, 124.9, 120.9, 111.1, 108.0, 55.5. IR 3380.4, 3068.4, 3002.5, 2948.9, 2835.9, 1596.2, 1476.1, 1452.2, 1243.8, 1108.8, 1021.6, 940.5, 744.7, 593.1, 540.5 cm^{-1}. HRMS (ESI$^+$) (m/z) calcd. for $C_9H_{10}BrO$ $[M+H]^+$ 212.9910; found 212.9914.

(*E*)-β-Bromo-4-methoxystyrene (4c)

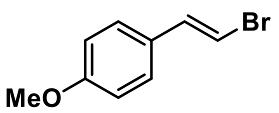

4c

4c was purified by flash column chromatography (ethyl acetate/hexane = 1:10 to 1:2) as a white solid (170 mg, 80%). M.P. 53-55°C. ^{1}H-NMR (400 MHz, $CDCl_3$) δ: 7.26-7.21 (m, 2H), 7.04 (d, J = 13.9 Hz, 1H), 6.88-6.83 (m, 2H), 6.61 (d, J = 13.9 Hz, 1H), 3.81 (s, 3H). ^{13}C-NMR (100 MHz, $CDCl_3$) δ: 159.8, 136.7, 128.9, 127.5, 114.3, 104.1, 55.4. IR 3065.8, 2956.9, 2930.7, 2835.8, 1600.0, 1506.8, 1456.4, 1250.5, 1109.5, 1025.7, 948.9, 927.0, 834.4, 773.8, 522.8 cm^{-1}. HRMS (ESI^{+}) (m/z) calcd. for $C_9H_{10}BrO$ $[M+H]^+$ 212.9910; found 212.9917.

(*E*)-β-Bromo-3,4-dimethoxystyrene (4d)

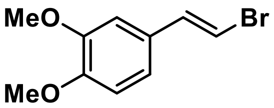

4d

$FeBr_3$ (29.6 mg, 0.1 mmol) was used as the control catalyst according to **General Procedure II**. **4d** was purified by flash column chromatography (ethyl acetate/hexane = 1:10 to 1:5) as a white solid [Fe(TPY10)Br_3: 221 mg, 91%; $FeBr_3$: 19.4 mg, 8%]. M.P. 54-56°C. ^{1}H-NMR (400 MHz, $CDCl_3$) δ: 7.03 (d, J = 13.9 Hz, 1H), 6.88–6.79 (m, 3H), 6.62 (d, J = 13.9 Hz, 1H), 3.884 (s, 3H), 3.877 (s, 3H). ^{13}C-NMR (100 MHz, $CDCl_3$) δ: 149.4, 149.2, 136.9, 129.1, 119.5, 111.2, 108.6, 104.3, 56.03, 55.98. IR 3077.0, 2950.9, 2834.0, 1507.5, 1459.8, 1412.5, 1251.6, 1198.8, 1132.2, 1018.0, 937.0, 851.8, 766.6 cm^{-1}. HRMS (ESI^{+}) (m/z) calcd. for $C_{10}H_{12}BrO_2$ $[M+H]^+$ 243.0015; found 243.0015.

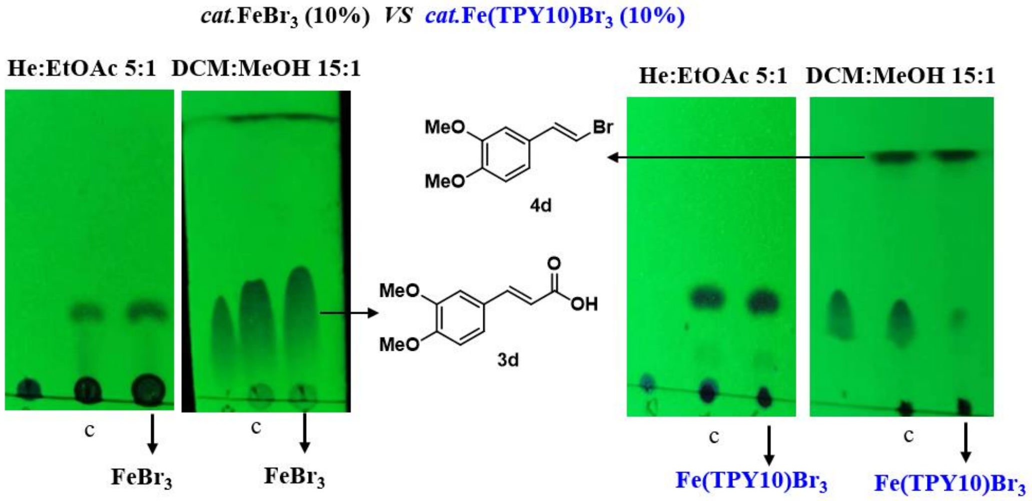

(*E*)-β-Bromo-2,5-dimethoxystyrene (4e)

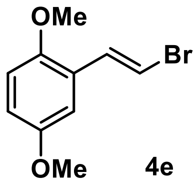

4e

4e was purified by flash column chromatography (ethyl acetate/hexane = 1:10 to 1:3) as a white solid (192 mg, 79%). M.P. 50-52°C. ^{1}H-NMR (400 MHz, $CDCl_3$) δ: 7.29 (d, J = 14.0 Hz, 1H), 6.91 (d, J = 13.9 Hz, 1H), 6.85-6.80 (m, 3H), 3.81 (s, 3H), 3.77 (s, 3H). ^{13}C-NMR (100 MHz, $CDCl_3$) δ: 153.7, 151.1, 133.0, 125.5, 114.2, 113.4, 112.3, 108.4, 56.2, 55.9. IR 2945.1, 2833.5, 1493.2, 1455.8, 1275.1, 1220.2, 1171.6, 1120.9, 1036.6, 939.3, 800.6, 702.9, 602.2 cm^{-1}. HRMS (ESI^{+}) (m/z) calcd. for $C_{10}H_{12}BrO_2$ $[M+H]^+$ 243.0015; found 243.0013.

(*E*)-β-Bromo-2,3,4-trimethoxystyrene (4f)

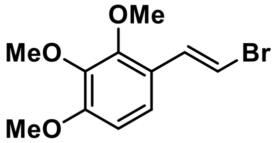

4f

4f was purified by flash column chromatography (ethyl acetate/hexane = 1:10 to 1:3) as a white solid (221 mg, 81%). M.P. 96-98°C. ^{1}H-NMR (400 MHz, $CDCl_3$) δ: 7.20 (d, *J* = 13.9 Hz, 1H), 6.98 (d, *J* = 8.7 Hz, 1H), 6.77 (d, *J* = 14.0 Hz, 1H), 6.63 (d, *J* = 8.7 Hz, 1H), 3.87 (s, 3H), 3.86 (s, 6H). ^{13}C-NMR (100 MHz, $CDCl_3$) δ: 153.9, 151.3, 142.5, 132.3, 123.0, 121.9, 107.7, 106.2, 61.1, 61.0, 56.1. IR 3069.9, 2938.9, 2837.2, 1716.0, 1583.1, 1453.9, 1409.7, 1275.3, 1200.2, 1087.5, 1029.5, 941.6, 787.0, 691.2, 503.7 cm^{-1}. HRMS (ESI^{+}) (m/z) calcd. for $C_{11}H_{14}BrO_3$ $[M+H]^{+}$ 273.0121; found 273.0121.

(*E*)-β-Bromo-2,4,5-trimethoxystyrene (4g)

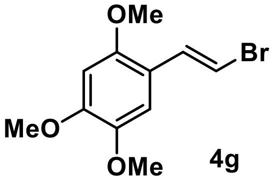

4g

$FeBr_3$ (29.6 mg, 0.1 mmol) was used as the control catalyst according to **General Procedure II**. **4g** was purified by flash column chromatography (ethyl acetate/hexane = 1:10 to 1:2) as a light-yellow oil [Fe(TPY10)Br_3: 240 mg, 88%; $FeBr_3$: 79.2 mg, 29%]. ^{1}H-NMR (400 MHz, $CDCl_3$) δ: 7.24 (d, *J* = 13.8 Hz, 1H), 6.80-6.70 (m, 2H), 6.48 (s, 1H), 3.89 (s, 3H), 3.84 (s, 3H), 3.83 (s, 3H). ^{13}C-NMR (100 MHz, $CDCl_3$) δ: 151.5, 150.0, 143.3, 132.5, 116.6, 111.0, 105.5, 97.5, 56.7, 56.5, 56.2. IR 3077.0, 2933.4, 2837.2, 1604.7, 1507.1, 1452.8, 1398.3, 1306.8, 1195.9, 1118.7, 1028.6, 937.4, 815.4, 768.0, 651.2 cm^{-1}. HRMS (ESI^{+}) (m/z) calcd. for $C_{11}H_{14}BrO_3$ $[M+H]^{+}$ 273.0121; found 273.0122.

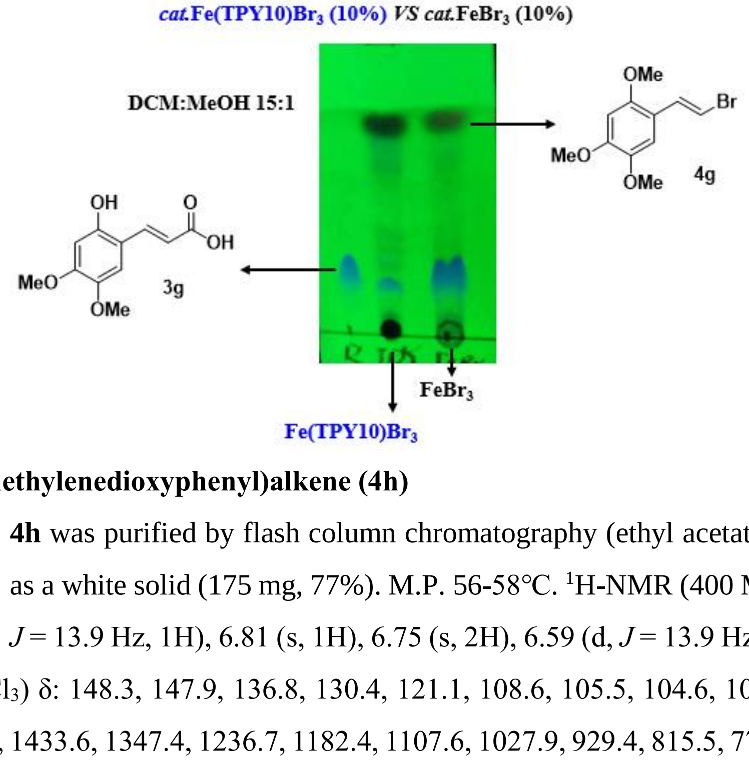

(*E*)-1-Bromo-2-(3,4-methylenedioxyphenyl)alkene (4h)

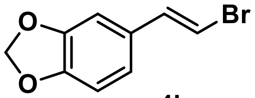

4h

4h was purified by flash column chromatography (ethyl acetate/hexane = 1:10 to 1:3) as a white solid (175 mg, 77%). M.P. 56-58°C. ^{1}H-NMR (400 MHz, $CDCl_3$) δ: 7.00 (d, *J* = 13.9 Hz, 1H), 6.81 (s, 1H), 6.75 (s, 2H), 6.59 (d, *J* = 13.9 Hz, 1H), 5.96 (s, 2H). ^{13}C-NMR (100 MHz, $CDCl_3$) δ: 148.3, 147.9, 136.8, 130.4, 121.1, 108.6, 105.5, 104.6, 101.4. IR 3081.8, 2996.5, 2907.3, 1597.8, 1486.3, 1433.6, 1347.4, 1236.7, 1182.4, 1107.6, 1027.9, 929.4, 815.5, 774.6, 506.1 cm^{-1}. HRMS (ESI^{+}) (m/z) calcd. for $C_9H_8BrO_2$ $[M+H]^{+}$ 226.9702; found 226.9711.

(*E*)-4-(2-Bromovinyl)phenyl acetate (4i)

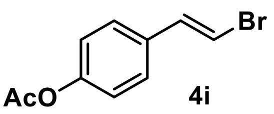

Additional NH_4Br (294 mg, 3 mmol) and H_2O_2 (30%, 0.3 mL, 3 mmol) were needed. **4i** was purified by flash column chromatography (ethyl acetate/hexane = 1:10 to 1:2) as a white solid (176 mg, 73%). M.P. 66-68°C. ^{1}H-NMR (400 MHz, $CDCl_3$) δ: 7.30 (d, *J* = 8.2 Hz, 2H), 7.15-7.00 (m, 3H), 6.73 (d, *J* = 13.7 Hz, 1H), 2.30 (s, 3H). ^{13}C-NMR (100 MHz, $CDCl_3$) δ: 169.4, 150.6, 136.3, 133.8, 127.3, 122.1, 106.8, 21.3. IR 3397.7, 2920.7, 1750.9, 1605.7, 1501.3, 1364.0, 1211.9, 1097.0, 946.2, 908.6, 843.5, 605.3, 514.7 cm^{-1}. HRMS (ESI$^+$) (m/z) calcd. for $C_{10}H_{10}BrO_2$ [M+H]$^+$ 240.9859; found 240.9857.

(*E*)-*N*-[4-(2-bromovinyl)phenyl]acetamide (4j)

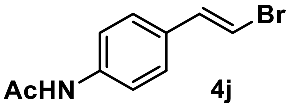

Additional NH_4Br (294 mg, 3 mmol) and H_2O_2 (30%, 0.3 mL, 3 mmol) were needed. **4j** was purified by flash column chromatography (ethyl acetate/dichloromethane = 1:20 to 1:10) as a white solid (204 mg, 85%) M.P. 173-175°C. ^{1}H-NMR (400 MHz, $CDCl_3$) δ: 7.47 (d, *J* = 8.3 Hz, 2H), 7.32 (s, 1H), 7.24 (d, *J* = 8.5 Hz, 2H), 7.04 (d, *J* = 14.0 Hz, 1H), 6.69 (d, *J* = 14.0 Hz, 1H), 2.17 (s, 3H). ^{13}C-NMR (100 MHz, $CDCl_3$) δ: 168.4, 138.0, 136.6, 132.1, 126.9, 120.0, 105.8, 24.8. IR 3292.4, 2918.6, 2852.0, 1658.5, 1594.5, 1530.8, 1402.9, 1364.3, 1315.5, 1256.4, 1218.6, 932.0, 832.8, 738.5, 516.6 cm^{-1}. HRMS (ESI$^+$) (m/z) calcd. for $C_{10}H_{11}BrNO$ [M+H]$^+$ 240.0019; found 240.0020.

(*E*)-2-(2-bromovinyl)thiophene (4k)

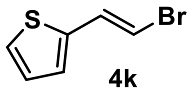

4k was purified by flash column chromatography (hexane to ethyl acetate/hexane = 1:30) as a colorless oil (117 mg, 62%). ^{1}H-NMR (400 MHz, $CDCl_3$) δ: 7.23-7.17 (m, 2H), 7.00-6.95 (m, 2H), 6.63 (d, *J* = 13.9 Hz, 1H). ^{13}C-NMR (100 MHz, $CDCl_3$) δ: 140.0, 130.4, 127.6, 126.2, 125.2, 105.3. HRMS (ESI$^+$) (m/z) calcd. for C_6H_6BrS [M+H]$^+$ 188.9368; found 188.9371. IR 3062.1, 2920.6, 2852.5, 1595.2, 1423.0, 1232.8, 1206.4, 1079.5, 1043.3, 923.1, 764.9, 690.0, 579.2 cm^{-1}. HRMS (ESI$^+$) (m/z) calcd. for C_6H_6BrS [M+H]$^+$ 188.9368; found 188.9362.

(*E*)-2-(2-bromovinyl)-5-methylthiophene (4l)

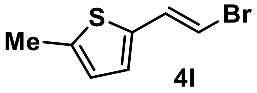

4l was purified by flash column chromatography (hexane) as a colorless oil (114 mg, 56%). ^{1}H-NMR (400 MHz, $CDCl_3$) δ: 7.09 (d, *J* = 13.8 Hz, 1H), 6.74 (d, *J* = 3.5 Hz, 1H), 6.63-6.60 (m, 1H), 6.47 (d, *J* = 13.8 Hz, 1H), 2.44 (d, *J* = 1.1 Hz, 3H). ^{13}C-NMR (100 MHz, $CDCl_3$) δ: 140.2, 138.0, 130.7, 126.5, 125.7, 103.8, 15.7. IR 3060.8, 2918.1, 2857.6, 1736.1, 1685.1, 1594.6, 1461.5, 1225.1, 1164.6, 1113.0, 1040.0 922.7, 792.0, 762.1, 673.0 cm^{-1}. HRMS (ESI$^+$) (m/z) calcd. for C_7H_8BrS [M+H]$^+$ 202.9525; found 202.9532.

3-Bromo-7-hydroxyl-4-methyl-2*H*-chromen-2-one (4m)

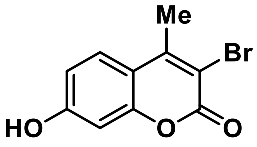

4m

Additional LiOAc (66 mg, 1 mmol) was needed. **4m** was purified by flash column chromatography (hexane/ethyl acetate = 10:1 to 2:1) as a colorless oil (99.5 mg, 39%). ^{1}H-NMR (400 MHz, DMSO) δ: 10.67 (s, 1H), 7.70 (d, *J* = 8.9 Hz, 1H), 6.83 (dd, *J* = 8.8, 2.4 Hz, 1H), 6.74 (d, *J* = 2.3 Hz, 1H), 2.54 (s, 3H). ^{13}C-NMR (100 MHz, DMSO) δ: 161.4, 156.5, 153.1, 152.1, 127.4, 113.5, 111.8, 107.5, 102.0, 19.3. IR 3225.8, 3076.3, 2882.3, 2803.1, 1690.5, 1607.1, 1539.0, 1441.0, 1305.6, 1260.9, 1137.2, 812.5, 749.7, 675.2 cm^{-1}. HRMS (ESI$^+$) (m/z) calcd. for $C_{10}H_8BrO_3$ [M+H]$^+$ 254.9651; found 254.9655.

7. Application of *cat.*Fe(TPY10)Br$_3$ for the bromolactonization of unsaturated carboxylic acids.

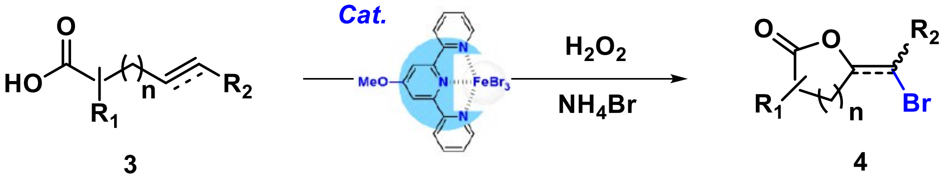

General Procedure III: To a stirred solution of unsaturated carboxylic acid **3** (1 mmol), NH_4Br (294 mg, 3 mmol) and Fe(TPY10)Br$_3$ (55.8 mg, 0.1 mmol) in EtOH/H_2O (3/1, 10 mL) was added H_2O_2 (30%, 0.3 mL, 3 mmol) dropwise. After completion of the addition, the homogenous mixture was stirred vigorously at rt for 0.5 h. Then, additional NH_4Br (294 mg, 3 mmol) and H_2O_2 (30%, 0.3 mL, 3 mmol) were added, and the resulting mixture was continuously stirred at rt for 1 h. The reaction was quenched by aqueous $Na_2S_2O_3$ solution (0.2 M, 50 mL) and ethyl acetate (50 mL). The organic fractions were collected, and the aqueous phase was extracted with ethyl acetate (2 × 20 mL). The combined organic fractions were washed with H_2O, dried over Na_2SO_4, filtered, and concentrated under reduced pressure.

5-Bromomethyldihydrofuran-2(3*H*)-one (6a)

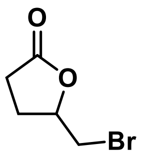

6a

H_2O (10 mL) was used as the solvent according to **General Procedure III**. **6a** was purified by flash column chromatography (hexane/ethyl acetate = 5:1 to 1:1) as a colorless oil (129 mg, 72%). ^{1}H-NMR (400 MHz, $CDCl_3$) δ: 4.78-4.70 (m, 1H), 3.59-3.50 (m, 2H), 2.70-2.51 (m, 2H), 2.43 (dddd, *J* = 13.1, 9.9, 7.3, 5.5 Hz, 1H), 2.11 (dddd, *J* = 13.1, 10.2, 8.0, 6.5 Hz, 1H). ^{13}C-NMR (100 MHz, $CDCl_3$) δ: 176.3, 77.9, 34.2, 28.5, 26.3. IR 2960.8, 1767.7, 1455.5, 1420.1, 1340.0, 1254.4, 1161.6, 1023.2, 987.6, 913.4, 647.5 cm^{-1}. HRMS (ESI$^+$) (m/z) calcd. for $C_5H_8BrO_2$ [M+H]$^+$ 178.9702; found 178.9702.

6-(Bromomethyl)tetrahydro-2*H*-pyran-2-one (6b)

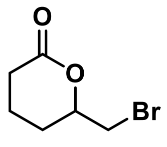

6b

6b was purified by flash column chromatography (hexane/ethyl acetate = 10:1 to 3:1) as a light yellow oil (104 mg, 54%). ^{1}H-NMR (400 MHz, $CDCl_3$) δ: 4.50-4.44 (m, 1H), 3.48 (dd, *J* =10.8 Hz, 4.9 Hz, 1H), 3.47 (dd, *J* = 10.8 Hz, 6.0 Hz, 1H), 2.58 (ddd, *J* = 17.9, 6.7, 4.8 Hz, 1H), 2.43 (ddd, *J* = 17.7, 9.6, 7.0 Hz, 1H), 2.07 (dq, *J* = 13.3, 4.2 Hz, 1H), 2.01-1.78 (m, 2H), 1.68 (dtd, *J* = 13.0, 10.9, 5.2 Hz, 1H). ^{13}C-NMR (100 MHz, $CDCl_3$) δ: 170.6, 78.6, 34.0, 29.4, 26.3, 18.2. IR 2925.7, 1713.9, 1242.0, 1167.8, 1093.2, 1044.4, 745.2, 657.3 cm^{-1}. HRMS (ESI^+) (m/z) calcd. for $C_6H_{10}BrO_2$ $[M+H]^+$ 192.9859; found 192.9865.

(*E*)-5-(bromomethylene)dihydrofuran-2(3*H*)-one (6c)

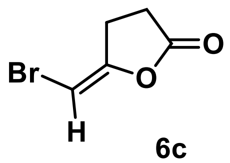

6c

DCM/H_2O (3/1, 10 mL) was used as the solvent according to **General Procedure III**. **6c** (Chem. Commun. 2011, 47, 12086) was purified by flash column chromatography (hexane/ethyl acetate = 10:1 to 2:1) as a light yellow oil (115 mg, 65%). ^{1}H-NMR (400 MHz, $CDCl_3$) δ: 5.95 (t, *J* = 2.4 Hz, 1H), 2.92-2.85 (m, 2H), 2.76-2.65 (m, 2H). ^{13}C-NMR (100 MHz, $CDCl_3$) δ: 174.2, 152.6, 85.3, 27.2, 24.9. IR 2922.2, 2853.1, 1713.0, 1400.8, 1175.5, 1079.6, 1018.0, 923.0, 843.9, 715.9, 499.0 cm^{-1}. HRMS (ESI^+) (m/z) calcd. for $C_5H_5BrNaO_2$ $[M+Na]^+$ 198.9365; found 198.9373.

5-(Bromomethyl)-3-methyldihydrofuran-2(3*H*)-one (6d)

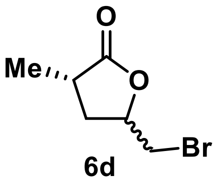

6d

$FeBr_3$ (29.6 mg, 0.1 mmol) was used as the control catalyst according to **General Procedure III**, and **6d** (J. Chem. Res. 2010, 34, 167) was purified by flash column chromatography (hexane to hexane/ethyl acetate = 4:1) as a yellow oil [Fe(TPY10)Br_3: 156 mg (*trans*:*cis* = 1:1), 81%; $FeBr_3$: 30.9 mg, 16%]. ^{1}H-NMR (400 MHz, $CDCl_3$) δ: 4.63-4.67 (m, 0.5H), 4.57-4.50 (m, 0.5H), 3.55-3.45 (m, 2H), 2.83-2.74 (m, 0.5H), 2.74-2.65 (m, 0.5H), 2.61-2.54 (m, 0.5H), 2.39-2.32 (m, 0.5H), 2.08-2.01 (m, 0.5H), 1.71-1.63 (m, 0.5H), 1.25 (d, *J* = 7.2 Hz, 3H). ^{13}C-NMR (100 MHz, $CDCl_3$) δ: 179.2, 178.4, 75.9, 75.8, 35.7, 35.6, 33.9, 33.8, 33.4, 16.2, 15.1. IR 2975.4, 2936.2, 2879.5, 1765.4, 1453.3, 1344.2, 1153.9, 1010.8, 926.6, 655.7cm^{-1}. HRMS (ESI^+) (m/z) calcd. for $C_6H_{10}BrO_2$ $[M+H]^+$ 192.9859; found 192.9867.

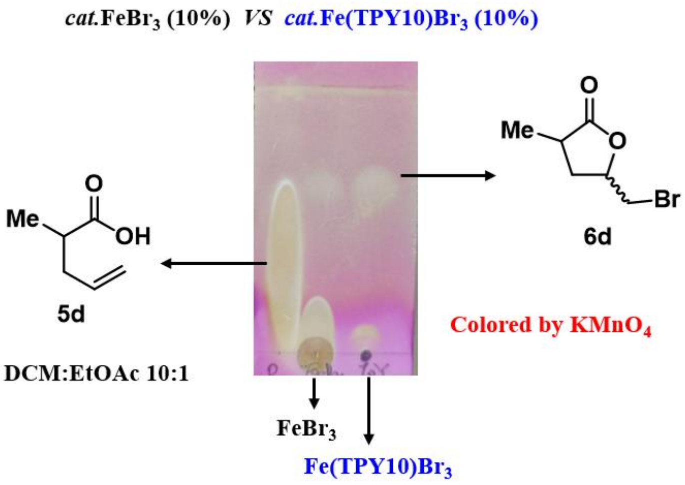

3-(*tert*-Butoxycarbonyl)amino-5-bromomethyldihydrofuran-2(3*H*)-one (6e)

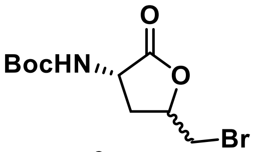

6e

6e (J. Am. Chem. Soc. 1989, 111, 2582) was purified by flash column chromatography (hexane/ethyl acetate = 10:1 to 2:1) as a light yellow oil [250 mg (*trans:cis* = 2:3), 85%]. ^{1}H NMR (400 MHz, $CDCl_3$) δ: 5.18-5.14 (m, 1H), 4.94-4-87 (m, 0.4H, *trans*), 4.66-4.58 (m, 0.6H, *cis*), 4.50-4.32 (m, 1H), 3.62-3.50 (m, 2H), 2.96-2.85 (m, 0.6H, *cis*), 2.70-2.55 (m, 0.4H, *trans*), 2.49-2.35 (m, 0.4H), 2.05–1.96 (m, 0.6H),1.45 (brs, 9H). ^{13}C-NMR (100 MHz, $CDCl_3$) δ: 175.0, 174.2, 155.3, 81.5, 80.5, 75.8, 75.5, 51.1, 49.4, 34.7, 34.5, 32.9, 32.3, 28.3. IR 3353.3, 2975.6, 2930.3, 1784.1, 1514.1, 1362.5, 1285.6, 1161.5, 1013.2, 615.1 cm^{-1}. HRMS (ESI$^+$) (m/z) calcd. for $C_{10}H_{17}BrNO_4$ $[M+H]^+$ 294.0335; found 294.0335.

5-(Bromomethyl)-3,3-dimethyldihydrofuran-2(3*H*)-one (6f)

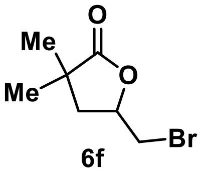

6f

6f was purified by flash column chromatography (hexane/ethyl acetate = 10:1 to 2:1) as a light yellow oil (170 mg, 82%). ^{1}H-NMR (400 MHz, $CDCl_3$) δ: 4.58 (dtd, *J* = 9.4, 6.1, 5.0 Hz, 1H), 3.50 (dd, *J* = 10.8 Hz, 5.1 Hz, 1H), 3.46 (dd, *J* =10.8 Hz, 5.9 Hz, 1H), 2.21 (dd, *J* = 13.0, 6.4 Hz, 1H), 1.88 (dd, *J* = 13.0, 9.5 Hz, 1H), 1.23 (s, 3H), 1.21 (s, 3H). ^{13}C-NMR (100 MHz, $CDCl_3$) δ: 180.9, 74.7, 41.7, 40.5, 33.8, 24.9, 24.8. IR 2970.4, 2933.5, 2874.1, 1766.2, 1459.5, 1202.8, 1108.8 1018.3, 853.0 cm^{-1}. HRMS (ESI$^+$) (m/z) calcd. for $C_7H_{11}BrNaO_2$ $[M+Na]^+$ 228.9835; found 228.9831.

3-(Bromomethyl)-2-oxaspiro[4.5]decan-1-one (6g)

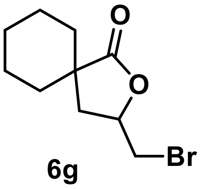

6g

6g was purified by flash column chromatography (hexane to hexane/ethyl acetate = 10:1 to 3:1) as a light yellow oil (217 mg, 88%). ^{1}H-NMR (400 MHz, $CDCl_3$) δ: 4.60-4.53 (m, 1H), 3.51 (dd, *J* = 10.7, 5.5 Hz, 1H), 3.47 (dd, *J* = 10.8, 6.4 Hz, 1H), 2.40 (dd, *J* = 13.1, 6.7 Hz, 1H), 1.83-1.64 (m, 4H), 1.61-1.46 (m, 4H), 1.39-1.16 (m, 3H). ^{13}C-NMR (100 MHz, $CDCl_3$)

δ: 180.5, 75.0, 45.0, 37.9, 34.2, 34.0, 32.2, 25.2, 22.1, 22.0. IR 2928.7, 2856.5, 1761.7, 1448.0, 1187.8, 1157.9, 1017.1, 936.8, 733.2, 657.2cm^{-1}. HRMS (ESI$^+$) (m/z) calcd. for $C_{10}H_{15}BrNaO_2$ $[M+Na]^+$ 269.0148; found 269.0153

5-(Bromomethyl)-5-methyldihydrofuran-2(3*H*)-one (6h)

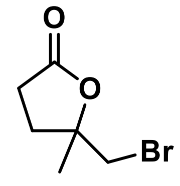

6h

6h was purified by flash column chromatography (hexane/ethyl acetate = 5:1 to 1:1) as a light yellow oil (143 mg, 74%). ^{1}H-NMR (400 MHz, $CDCl_3$) δ: 3.51 (d, J = 10.8 Hz, 1H), 3.45 (d, J = 11.1 Hz, 1H), 2.75-2.55 (m, 2H), 2.35 (ddd, J = 13.3, 10.3, 6.9 Hz, 1H), 2.06 (ddd, J = 13.2, 10.0, 6.9 Hz, 1H), 1.54 (s, 3H). ^{13}C-NMR (100 MHz, $CDCl_3$) δ: 175.9, 84.1, 39.6, 31.6, 29.2, 25.4. IR 2979.5, 1766.5, 1278.6, 1162.7, 1068.4, 943.2, 647.5, 514.9 cm^{-1}. HRMS (ESI$^+$) (m/z) calcd. for $C_6H_{10}BrO_2$ $[M+H]^+$ 192.9859; found 192.9864.

5-(Bromomethyl)-5-phenyldihydrofuran-2(3*H*)-one (6i)

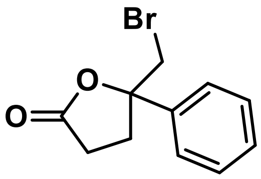

6i

$FeBr_3$ (29.6 mg, 0.1 mmol) was used as the control catalyst according to **General Procedure III**. **6i** was purified by flash column chromatography (hexane/ethyl acetate = 10:1 to 3:1) as a yellow oil [Fe(TPY10)Br_3: 199 mg, 78%; $FeBr_3$: 17.9 mg, 7%]. ^{1}H-NMR (400 MHz, $CDCl_3$) δ: 7.43-7.32 (m, 5H), 3.74 (d, J = 11.3 Hz, 1H), 3.69 (d, J = 11.4 Hz, 1H), 2.90-2.73 (m, 2H), 2.61-2.47 (m, 2H). ^{13}C-NMR (100 MHz, $CDCl_3$) δ: 175.6, 140.8, 129.0, 128.8, 125.0, 86.5, 41.1, 32.5, 29.2. IR 3060.2, 3029.9, 2960.8, 2926.0, 1774.0 1451.3, 1154.4, 1034.3, 925.5, 699.0, 661.8 cm^{-1}. HRMS (ESI$^+$) (m/z) calcd. for $C_{11}H_{12}BrO_2$ $[M+H]^+$ 255.0015; found 255.0021.

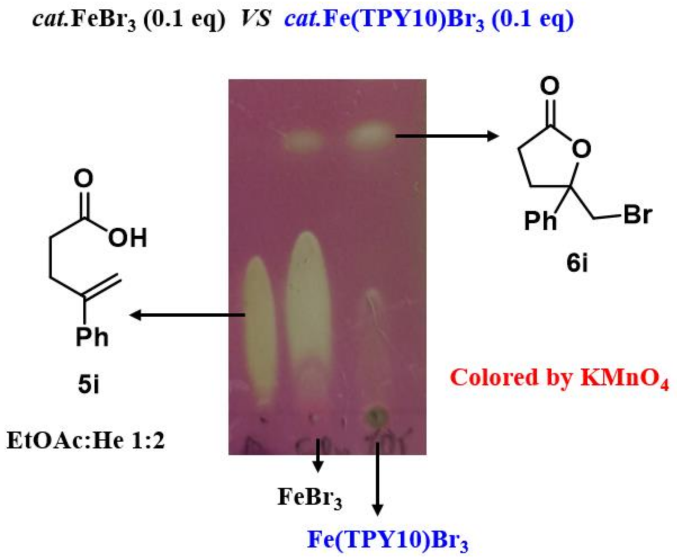

(±)-5-(1-Bromomethyl)dihydrofuran-2(3*H*)-one (6j)

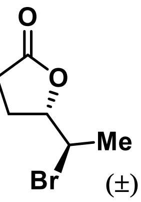

6j

6j (Angew. Chem. Int. Ed. 2018, 57, 13863) was purified by flash column chromatography (hexane to hexane/ethyl acetate = 5:1) as a light yellow oil (122 mg, 63%). ^{1}H-NMR (400 MHz, $CDCl_3$) δ: 4.44 (q, J = 7.0 Hz, 1H), 4.13 (p, J = 6.7 Hz, 1H), 2.65-2.48 (m, 2H), 2.46-2.36 (m, 1H), 2.17-2.08 (m, 1H), 1.74 (d, J = 6.8 Hz, 3H). ^{13}C-NMR (101 MHz, $CDCl_3$) δ: 176.3, 82.5, 50.5, 28.6, 25.6, 22.1. IR 2979.6, 2930.9, 1771.8, 1172.1, 1010.7, 983.7, 949.8, 847.7, 734.6, 626.1 cm^{-1}. HRMS (ESI$^+$) (m/z) calcd. for $C_6H_{10}BrO_2$ [M+H]$^+$ 192.9859; found 192.9866.

(±)-6-Bromohexahydro-2*H*-cyclopenta[*b*]furan-2-one (6k)

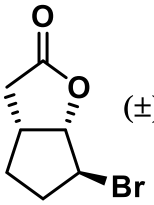

6k (±)

6k (J. Chem. Res. 2010, 34, 167) was purified by flash column chromatography (hexane/ethyl acetate = 10:1 to 2:1) as a yellow oil (144 mg, 70%). ^{1}H-NMR (400 MHz, $CDCl_3$) δ: 5.06 (d, J = 6.2 Hz, 1H), 4.43 (d, J = 4.6 Hz, 1H), 3.19-3.11 (m, 1H), 2.90-2.83 (m, 1H), 2.48-2.38 (m, 1H), 2.36-2.30 (m, 1H), 2.29-2.17 (m, 1H), 2.10-2.04 (m, 1H), 1.62-1.56 (m, 1H). ^{13}C-NMR (101 MHz, $CDCl_3$) δ: 176.5, 90.5, 52.9, 36.1, 36.0, 33.1, 31.4. IR 2967.7, 1772.7, 1346.1, 1225.7, 1152.8, 1009.4, 871.4, 658.2 cm^{-1}. HRMS (ESI$^+$) (m/z) calcd. for $C_7H_{10}BrO_2$ [M+H]$^+$ 204.9859; found 204.9860.

(±)-6-Bromohexahydro-2*H*-3,5-methanocyclopenta[*b*]furan-2-one (6l)

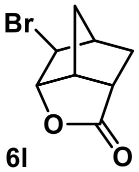

6l (±)

6l (RSC Adv. 2015, 5, 85872) was purified by flash column chromatography (hexane/ethyl acetate = 10:1 to 2:1) as a light yellow oil (187 mg, 86%). ^{1}H-NMR (400 MHz, $CDCl_3$) δ: 4.89 (d, J = 5.1 Hz, 1H), 3.82 (d, J = 2.3 Hz, 1H), 3.21 (t, J = 4.9 Hz, 1H), 2.64 (d, J = 4.1 Hz, 1H), 2.53 (dd, J = 11.5, 4.7 Hz, 1H), 2.29 (d, J = 11.5 Hz, 1H), 2.14-2.07 (m, 1H), 1.79-1.68 (m, 2H). ^{13}C-NMR (100 MHz, $CDCl_3$) δ: 179.3, 87.7, 53.6, 45.9, 45.5, 37.5, 35.7, 33.9. IR 2980.7, 2881.5, 1777.4, 1344.7, 1307.8 1002.3, 938.4, 883.4, 843.4, 670.6 cm^{-1}. HRMS(ESI$^+$) (m/z) calcd. for $C_8H_{10}BrO_2$ [M+H]$^+$ 216.9859; found 216.9862.

12α-Bromo-3β-hydroxyolean-28,13β-olide (6m)

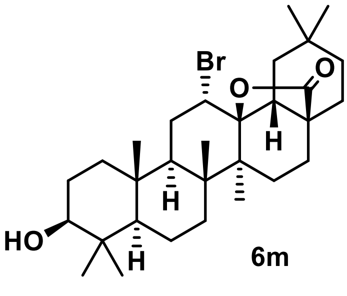

6m

6m (Tetrahedron 2015, 71, 792) was purified by flash column chromatography (hexane/ethyl acetate = 3:1 to 1:2) as a light yellow oil (305 mg, 57%). ^{1}H-NMR (400 MHz, $CDCl_3$) δ: 4.30 (dd, J = 3.9, 2.3 Hz, 1H), 3.25 (dd, J = 11.4, 4.7 Hz, 1H), 2.45-2.27 (m, 2H), 2.16 (td, J = 13.3, 5.6 Hz, 1H), 1.99 (s, 3H), 1.85 (d, J = 15.1 Hz, 1H), 1.75 (dd, J = 12.4, 1.9 Hz, 1H), 1.72-1.60 (m, 5H), 1.59-1.50 (m, 3H), 1.44 (d, J = 0.9 Hz, 4H), 1.42-1.24 (m, 6H), 1.22 (d, J = 0.9 Hz, 3H), 1.12-1.03 (m, 1H), 1.00 (s, 6H), 0.91-0.87 (m, 6H), 0.78 (s, 3H). ^{13}C-NMR (100 MHz, $CDCl_3$) δ: 178.8, 91.6, 78.7, 56.5, 55.2, 52.4, 45.6, 45.5, 43.4, 42.5, 40.0, 38.9, 38.4, 36.6, 34.6, 33.9, 33.3, 31.9, 30.5, 29.2, 28.0, 27.6, 27.2, 23.6, 21.4, 21.1, 19.1, 17.7, 16.9, 15.4. IR 3345.6, 2954.2, 2870.1, 1765.0, 1461.7, 1386.1, 1210,0, 1135.8,

1106.9, 926.3, 643.0 cm^{-1}. HRMS(ESI^+) (m/z) calcd. for $C_{30}H_{48}BrO_3$ $[M+H]^+$ 535.2781; found 535.2787.

8. Catalytic application of $Fe(TPY10)Br_3$ for the sulfide oxidation to sulfoxide.

8.1 Reagent preparation

Substrate **7e** (J. Org. Chem. 2004, 69, 7592), **7f** (J. Org. Chem. 2003, 68, 5890) and **7u** (Anal. Chim. Acta 2013, 800, 71) were prepared according to the published literature, and other reagents can be purchased directly from Sigma-Aldrich Co.

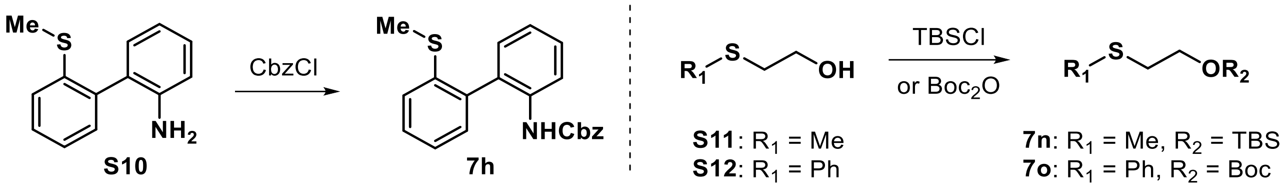

General Procedure: To a stirred solution of substrates **S10** (Angew. Chem., Int. Ed. 2016, 55, 12751), **S11** or **S12** (5 mmol) and base in anhydrous solvent were added protecting reagents (benzyl chloroformate, TBSCl or Boc_2O) at 0°C. After completion of the addition, the reaction mixture was allowed to warm to rt and stirred for 0.5-3 h (detected by TLC). The reaction was quenched by H_2O (100 mL) and dichloromethane (DCM, 100 mL). The organic fractions were collected, and the aqueous phase was extracted with DCM (3 × 30 mL). The combined organic fractions were washed with brine, dried over Na_2SO_4, filtered, and concentrated under reduced pressure. The crude product was purified by flash column chromatography.

2-[(Benzyloxycarbonyl)amino]-2'-methylthiobiphenyl (7h)

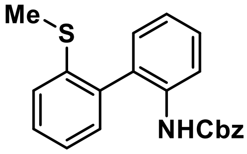

7h

K_2CO_3 (1.38 g, 10 mmol) and benzyl chloroformate (1.07 mL, 7.5 mmol) were added to the solvent of acetone (25 mL), and **7h** was purified by flash column chromatography (ethyl acetate/hexane = 1:10 to 1:2) as a white solid (1.05 g, 60%). M.P. 90-92°C. ^{1}H-NMR (400 MHz, $CDCl_3$) δ: 8.15 (brd, J = 8.2 Hz, 1H), 7.44-7.37 (m, 2H), 7.36-7.32 (m, 5H), 7.30-7.27 (m, 1H), 7.25-7.20 (m, 1H), 7.17-7.14 (m, 3H), 6.42(s, 1H), 5.18-5.10 (m, 2H), 2.35 (s, 3H). ^{13}C-NMR (100 MHz, $CDCl_3$) δ: 153.5, 138.8, 136.2, 135.8, 135.4, 130.5, 130.3, 129.8, 129.1, 128.6, 128.33, 128.28, 125.1, 125.0, 123.5, 120.0, 66.9, 15.2. IR 3409.5, 3031.6, 2916.3,1743.8, 1581.4, 1516.9, 1436.7, 1302.6, 1200.4, 1056.7 cm^{-1}. HRMS (ESI^+) (m/z) calcd. for $C_{21}H_{20}NO_2S$ $[M+H]^+$ 350.1209; found 350.1195.

2-(*tert*-Butyldimethylsilyloxy)ethyl methyl sulfide (7n)

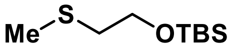

7n

Imidazole (374 mg, 5.5 mmol) and TBSCl (829 mg, 5.5 mmol) were added to the solvent of DCM (25 mL), and **7n** was purified by flash column chromatography (ethyl acetate/hexane = 1:5 to 1:2) as a light-yellow oil (774 mg, 75%), which was used for the following sulfoxidation directly due to its unstability under the normal condition.

2-[(*tert*-Butyloxycarbonyl)oxy]ethyl phenyl sulfide (7o)

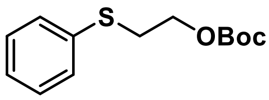

7o

DMAP (61 mg, 0.5 mmol), Et_3N (1.39 mL, 10 mmol) and Boc_2O (1.20 g, 5.5 mmol) were added to the solvent of DCM (25 mL), and **7o** was purified by flash column chromatography (ethyl acetate/hexane = 1:20 to 1:10) as a light-yellow oil (890 mg, 70%). ^{1}H-NMR (400 MHz, $CDCl_3$) δ: 7.41-7.38 (m, 2H), 7.32-7.26 (m, 2H), 7.24-7.18 (m, 1H), 4.22 (t, J = 7.0 Hz, 2H), 3.18 (t, J = 7.2 Hz, 2H), 1.48 (s, 9H). ^{13}C-NMR (100 MHz, $CDCl_3$) δ: 153.2, 135.0, 129.9, 129.1, 126.6, 82.4, 65.1, 32.2, 27.8. IR 2978.7, 1738.1, 1582.3, 1476.3, 1372.3, 1251.5, 1154.5, 1094.8 cm^{-1}. HRMS (ESI$^+$) (m/z) calcd. for $C_{13}H_{19}O_3S$ [M+H]$^+$ 255.1049; found 255.1047.

8.2 Detail information for the sulfoxide synthesis

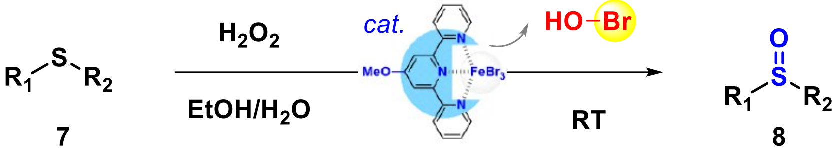

General Procedure IV: To a stirred solution of sulfide **7** (1 mmol) and Fe(TPY10)Br_3 (11.2 mg, 0.02 mmol) in EtOH/H_2O (3/1, 10 mL) was added H_2O_2 (30%, 0.2 mL, 2 mmol) dropwise. After completion of the addition, the reaction mixture was stirred at rt for 1 h. The reaction was quenched by aqueous $Na_2S_2O_3$ solution (0.1 M, 50 mL) and ethyl acetate (50 mL). The organic fractions were collected, and the aqueous phase was extracted with ethyl acetate (2 ×20 mL). The combined organic fractions were washed with brine, dried over Na_2SO_4, filtered, and concentrated under reduced pressure. The resulting residue was purified by flash column chromatography to give compound **8**.

Methyl phenyl sulfoxide (8a)

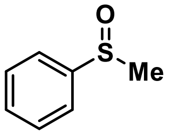

8a

8a was purified by flash column chromatography (ethyl acetate/hexane = 1:2 to 3:1) as a light-yellow oil (130 mg, 93%). ^{1}H-NMR (400 MHz, $CDCl_3$) δ: 7.65-7.62 (m, 2H), 7.52-7.49 (m, 3H), 2.70 (s, 3H). ^{13}C-NMR (100 MHz, $CDCl_3$) δ: 145.8, 131.0, 129.4, 123.5, 44.0. IR 3057.4, 1708.2, 1476.3, 1441.5, 1412.2, 1363.0, 1300.4, 1162.8, 1085.6, 1029.1 cm^{-1}. HRMS (ESI$^+$) (m/z) calcd. for C_7H_9OS [M+H]$^+$ 141.0369; found 141.0368.

2-Fluorophenyl methyl sulfoxide (8b)

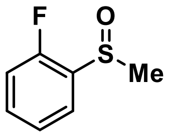

8b

8b was purified by flash column chromatography (ethyl acetate/hexane = 1:2 to 3:1) as a light-yellow oil (152 mg, 96%). ^{1}H-NMR (400 MHz, DMSO) δ: 7.79-7.74 (m, 1H), 7.63-7.59 (m, 1H), 7.51-7.47 (m, 1H), 7.40-7.35 (m, 1H), 2.82 (s, 3H). ^{13}C-NMR (100 MHz, DMSO) δ: 158.8 (d, $^1J_{C\text{-}F}$ = 243.7 Hz), 133.7 (d, $^3J_{C\text{-}F}$ = 7.6 Hz), 133.6 (d, $^2J_{C\text{-}F}$ = 16.7 Hz), 126.3 (d, $^3J_{C\text{-}F}$ = 3.3 Hz), 125.6 (d, $^4J_{C\text{-}F}$ = 2.5 Hz), 116.6 (d, $^2J_{C\text{-}F}$ = 20.1 Hz), 42.4 (d, $^4J_{C\text{-}F}$ = 1.4 Hz). ^{19}F-NMR (376 MHz, $CDCl_3$)

δ: -114.76 (dt, $^3J_{F\text{-}H}$ = 10.0 Hz, $^4J_{F\text{-}H}$ = 6.1 Hz). IR 2922.7, 1770.6, 1711.0, 1587.0, 1468.5, 1414.7, 1298.1, 1257.3, 1211.8, 1168.5, 1122.7, 1072.8, 1041.5 cm^{-1}. HRMS (ESI^+) (m/z) calcd. for C_7H_8FOS $[M+H]^+$ 159.0274; found 159.0272.

3-Nitrophenyl methyl sulfoxide (8c)

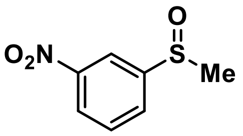

8c

8c was purified by flash column chromatography (ethyl acetate/hexane = 1:2 to 3:1) as a white solid (167 mg, 90%). M.P. 110-112°C. ^{1}H-NMR (400 MHz, $CDCl_3$) δ: 8.48-8.47 (m, 1H), 8.34-8.32 (m, 1H), 8.00-7.97 (m, 1H), 7.77 (t, J = 8.0 Hz, 1H), 2.79 (s, 3H). ^{13}C-NMR (100 MHz, $CDCl_3$) δ: 148.72, 148.67, 130.7, 129.3, 125.7, 119.0, 44.0. IR 2920.5, 1707.3, 1601.1, 1515.9, 1419.9, 1339.6, 1257.4, 1122.7, 1065.7, 1036.8 cm^{-1}. HRMS (ESI^+) (m/z) calcd. for $C_7H_8NO_3S$ $[M+H]^+$ 186.0219; found 186.0222.

4-Methoxyphenyl methyl sulfoxide (8d)

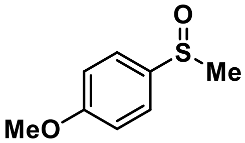

8d

8d was purified by flash column chromatography (ethyl acetate/hexane = 1:2 to 3:1) as a light-yellow oil (162 mg, 95%). ^{1}H-NMR (400 MHz, $CDCl_3$) δ: 7.59 (d, J = 8.8 Hz, 2H), 7.02 (d, J = 8.8 Hz, 2H), 3.84 (s, 3H), 2.68 (s, 3H). ^{13}C-NMR (100 MHz, $CDCl_3$) δ: 162.0, 136.7, 125.5, 114.9, 55.5, 44.0. IR 2987.2, 2953.8, 2838.1, 1647.0, 1589.8, 1492.1, 1461.0, 1299.6, 1247.2, 1171.7, 1084.9, 1008.9 cm^{-1}. HRMS (ESI^+) (m/z) calcd. for $C_8H_{11}O_2S$ $[M+H]^+$ 171.0474; found 171.0477.

Cyclopropyl phenyl sulfoxide (8e)

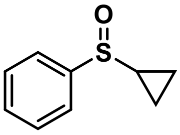

8e

8e was purified by flash column chromatography (ethyl acetate/hexane = 1:2 to 3:1) as a light-yellow oil (161 mg, 97%). ^{1}H-NMR (400 MHz, $CDCl_3$) δ: 7.65 (dd, J = 7.9, 2.4 Hz, 2H), 7.49-7.46(m, 3H), 2.26-2.20 (m, 1H), 1.22-1.17 (m, 1H), 1.02-0.97 (m, 1H), 0.95-0.88 (m, 2H). ^{13}C-NMR (100 MHz, $CDCl_3$) δ: 144.9, 130.9, 129.2, 124.0, 33.8, 3.4, 2.8. IR 3461.0, 3057.6, 3007.6, 1477.0, 1439.4, 1304.0, 1183.4, 1080.2, 1032.0, 876.9, 747.8, 686.1 cm^{-1}. HRMS (ESI^+) (m/z) calcd. for $C_9H_{11}OS$ $[M+H]^+$ 167.0525; found 167.0530.

Allyl phenyl sulfoxide (8f)

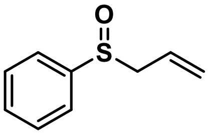

8f

8f was purified by flash column chromatography (ethyl acetate/hexane = 1:2 to 3:1) as a colorless oil (136 mg, 82%). ^{1}H-NMR (400 MHz, $CDCl_3$) δ: 7.60-7.57 (m, 2H), 7.53-7.44 (m, 3H), 5.68-5.57 (m, 1H), 5.32 (d, J = 10.2 Hz, 1H), 5.20 (dd, J = 17.0, 1.4 Hz, 1H), 3.58-3.46 (m, 2H). ^{13}C-NMR (100 MHz, $CDCl_3$) δ: 143.0, 131.1, 129.1,125.3, 124.3, 123.9, 60.9. IR 3455.7, 3059.8, 1724.5, 1634.4, 1584.5, 1476.8, 1436.1, 1300.4, 1214.7, 1084.1, 1035.6, 927.4, 747.5, 690.5 cm^{-1}. HRMS (ESI^+) (m/z) calcd. for $C_9H_{11}OS$ $[M+H]^+$ 167.0525; found 167.0529.

Phenyl propargyl sulfoxide (8g)

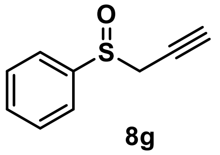

8g

8g was purified by flash column chromatography (ethyl acetate/hexane = 1:2 to 3:1) as a colorless oil (136 mg, 83%). ^{1}H-NMR (400 MHz, $CDCl_3$) δ: 7.72-7.69 (m, 2H), 7.54-7.52 (m, 3H), 3.69-3.58 (m, 2H), 2.34 (t, J = 2.7 Hz, 1H). ^{13}C-NMR (100 MHz, $CDCl_3$) δ: 142.8, 131.8, 129.1, 124.5, 76.4, 72.7, 47.8. IR 3354.2, 3006.8, 2933.9, 2839.1, 1583.6, 1487.8, 1449.4, 1291.3, 1248.4, 1135.7, 1093.3, 1017.4, 815.8, 755.8, 527.0 cm^{-1}. HRMS (ESI^{+}) (m/z) calcd. for C_9H_9OS [M+H]$^{+}$ 165.0369; found 165.0362.

2-[2-[(Benzyloxycarbonyl)amino]phenyl]phenyl methyl sulfoxide (8h)

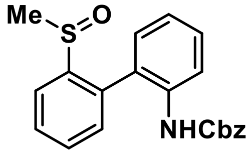

8h

8h was purified by flash column chromatography (ethyl acetate/hexane = 1:2 to 3:1) as a white solid (329 mg, 90%). M.P. 132-134°C. ^{1}H-NMR (400 MHz, $CDCl_3$) δ: 8.16-8.13 (m, 2H), 7.70-7.64 (m, 1H), 7.61-7.54 (m, 1H), 7.46-7.41 (m, 1H), 7.34-7.02 (m, 8H), 6.42 (m, 1H), 5.20-5.03 (m, 2H), 2.44-2.31 (m, 3H). ^{13}C-NMR (100 MHz, $CDCl_3$) δ: 153.3, 153.1, 145.4, 145.3, 135.8, 135.7, 135.20, 135.18, 134.7, 134.5, 131.7, 131.2, 131.0, 130.42, 130.38, 130.3, 130.1, 129.92, 129.91, 129.87, 128.7, 128.58, 128.56, 128.5, 128.4, 128.3, 127.2, 126.8, 125.0, 123.8, 123.7, 120.8, 120.6, 67.3, 67.2, 42.7, 41.2. IR 3174.9, 2959.2, 1947.0, 1710.2, 1582.4, 1539.7, 1442.7, 1370.6, 1298.5, 1215.6, 1122.5, 1042.0 cm^{-1}. HRMS (ESI^{+}) (m/z) calcd. for $C_{21}H_{20}NO_3S$ [M+H]$^{+}$ 366.1158; found 366.1148.

2-Fluoro-pyridin-3-yl methyl sulfoxide (8i)

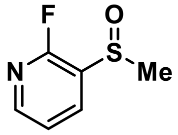

8i

8i was purified by flash column chromatography (ethyl acetate/hexane = 1:2 to ethyl acetate) as a colorless oil (140 mg, 88%). ^{1}H-NMR (400 MHz, $CDCl_3$) δ: 8.32-8.28 (m, 2H), 7.47-7.43 (m, 1H), 2.85 (s, 3H). ^{13}C-NMR (100 MHz, $CDCl_3$) δ: 158.7 (d, $^{1}J_{C\text{-}F}$ = 235.1 Hz), 149.9 (d, $^{3}J_{C\text{-}F}$ = 14.5 Hz), 137.3 (d, $^{5}J_{C\text{-}F}$ = 3.5 Hz), 128.8 (d, $^{2}J_{C\text{-}F}$ = 33.6 Hz), 122.9 (d, $^{4}J_{C\text{-}F}$ = 4.4 Hz), 41.7 (d, $^{4}J_{C\text{-}F}$ = 1.7 Hz). ^{19}F-NMR (376 MHz, $CDCl_3$) δ: -65.90 (d, $^{4}J_{F\text{-}H}$ = 8.9 Hz). IR 3058.5, 1587.4, 1409.6, 1299.3, 1244.4, 1213.1, 1078.6, 1042.0 cm^{-1}. HRMS (ESI^{+}) (m/z) calcd. for C_6H_7FNOS [M+H]$^{+}$ 160.0227; found 160.0220.

3-Methylpyrazin-2-yl methyl sulfoxide (8j)

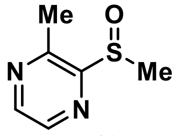

8j

$FeBr_3$ (5.9 mg, 0.02 mmol) was used as the control catalyst according to **General Procedure IV**. **8j** was purified by flash column chromatography (ethyl acetate/hexane = 1:1 to ethyl acetate) as a colorless oil [Fe(TPY10)Br_3: 147 mg, 94%; $FeBr_3$: 7.8 mg, 5%]. ^{1}H-NMR (400 MHz, $CDCl_3$) δ: 8.55 (d, J = 2.4 Hz, 1H), 8.50 (d, J = 2.4 Hz, 1H), 2.85 (s, 3H), 2.75 (s, 3H). ^{13}C-NMR (100 MHz, $CDCl_3$) δ: 156.8, 153.1, 145.9, 142.6, 38.4, 20.7. IR 2999.7, 1646.1, 1520.1, 1417.3,

1376.8, 1296.5, 1202.8, 1157.8, 1031.0 cm^{-1}. HRMS (ESI$^+$) (m/z) calcd. for $C_6H_9N_2OS$ [M+H]$^+$ 157.0430; found 157.0424.

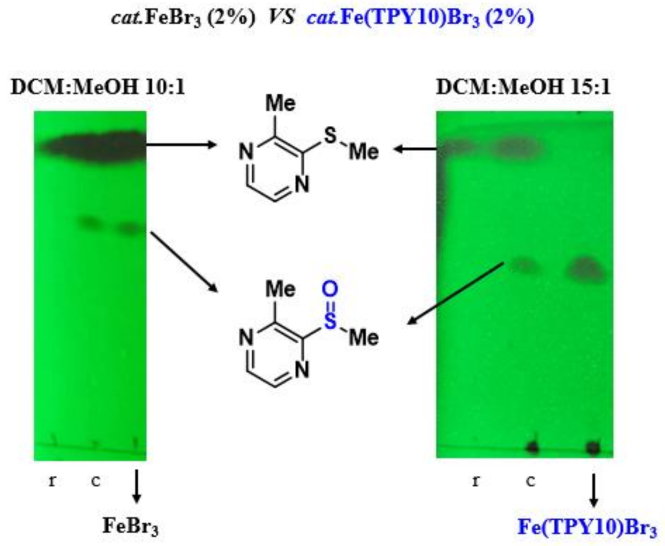

4-Chloro-6-methylpyrimidin-2-yl methyl sulfoxide (8k)

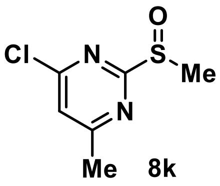

8k

$FeBr_3$ (5.9 mg, 0.02 mmol) was used as the control catalyst according to **General Procedure IV**. **8k** was purified by flash column chromatography (ethyl acetate/hexane = 1:1 to ethyl acetate) as a yellow oil [Fe(TPY10)Br_3: 183 mg, 96%; $FeBr_3$: 38.1 mg, 20%]. ^{1}H-NMR (400 MHz, $CDCl_3$) δ: 7.27 (s, 1H), 2.95 (s, 3H), 2.64 (s, 3H). ^{13}C-NMR (100 MHz, $CDCl_3$) δ: 174.3, 171.1, 162.6, 121.5, 40.3, 24.0. IR 3059.2, 1677.6, 1557.7, 1514.6, 1414.6, 1300.2, 1257.1, 1130.0, 1072.5 cm^{-1}. HRMS (ESI$^+$) (m/z) calcd. for $C_6H_8ClN_2OS$ [M+H]$^+$ 191.0040; found 191.0033.

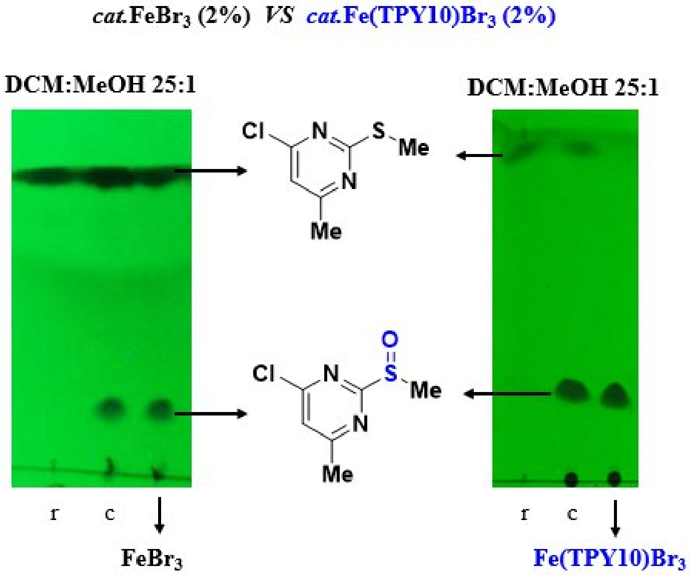

Phenoxathiin 10-oxide (8l)

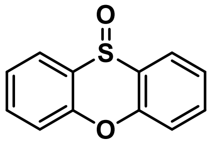

8l

8l was purified by flash column chromatography (ethyl acetate/hexane = 1:2 to 3:1) as a white solid (205 mg, 95%). M.P. 158-160°C. ^{1}H-NMR (400 MHz, $CDCl_3$) δ: 7.93 (dd, *J* = 7.8, 1.6 Hz, 2H), 7.63-7.58 (m, 2H), 7.43-7.40 (m, 2H) 7.38-7.34 (m, 2H). ^{13}C-NMR (100 MHz, $CDCl_3$) δ: 149.5, 133.8, 131.1, 124.9, 123.7, 118.8. IR 1807.2, 1580.9, 1429.5, 1310.3, 1263.3, 1215.3, 1130.2, 1076.3, 1029.8 cm^{-1}. HRMS (ESI$^+$) (m/z) calcd. for $C_{12}H_9O_2S$ [M+H]$^+$ 217.0318; found217.0326.

1,3-benzothiazol-2-yl methyl sulfoxide (8m)

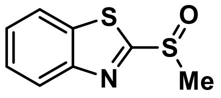

8m

Additional Fe(TPY10)Br_3 (5.6 mg, 0.01 mmol) and H_2O_2 (30%, 0.2 mL, 2 mmol) were added. **8m** was purified by flash column chromatography (ethyl acetate/hexane = 1:2 to 3:1) as a white solid (150 mg, 76%). M.P. 74-76°C. ^{1}H-NMR (400 MHz, $CDCl_3$) δ: 8.07 (d, *J* = 8.3 Hz, 1H), 8.01(d, *J* = 7.3 Hz, 1H), 7.59-7.54 (m, 1H), 7.52-7.47 (m, 1H), 3.08 (s, 3H). ^{13}C-NMR (100 MHz, $CDCl_3$) δ: 178.5, 153.8, 136.0, 127.0, 126.3, 124.0, 122.4, 43.2. IR 2918.6, 2851.8, 1552.2, 1472.6, 1424.2, 1286.6, 1234.2, 1125.1, 1061.3, 1007.5, 960.1, 848.0 cm^{-1}. HRMS (ESI$^+$) (m/z) calcd. for $C_8H_8NOS_2$ [M+H]$^+$ 198.0042; found 198.0039.

2-(*tert*-Butyldimethylsilyloxy)ethyl methyl sulfoxide (8n)

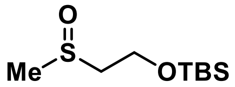

8n

8n was purified by flash column chromatography (ethyl acetate/hexane = 1:2 to 3:1) as a colorless oil (185 mg, 83%). ^{1}H-NMR (400 MHz, $CDCl_3$) δ: 4.06-4.00 (m, 2H), 2.91-2.84 (m, 2H), 2.62 (s, 3H), 0.88 (s, 9H), 0.07 (s, 6H). ^{13}C-NMR (100 MHz, $CDCl_3$) δ: 57.9, 56.2, 39.3, 25.8, 18.2, -5.5. IR 2930.6, 2858.0, 1467.8, 1389.3, 1254.0, 1100.5, 1043.7, 1000.3 cm^{-1}. HRMS (ESI$^+$) (m/z) calcd. for $C_9H_{23}O_2SSi$ [M+H]$^+$ 223.1183; found 223.1187.

2-[(*tert*-Butyloxycarbonyl)oxy]ethyl phenyl sulfoxide (8o)

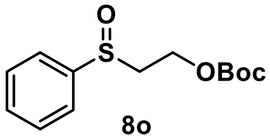

8o

8o was purified by flash column chromatography (ethyl acetate/hexane = 1:2 to 3:1) as a colorless oil (254 mg, 94%). ^{1}H-NMR (400 MHz, DMSO) δ: 7.71-7.68 (m, 2H), 7.61-7.57 (m, 3H), 4.31-4.27 (m, 2H), 3.32-3.05 (m, 2H), 1.41 (s, 9H). ^{13}C-NMR (100 MHz, DMSO) δ: 152.9, 144.3, 131.4, 129.8, 124.4, 82.4, 59.9, 54.9, 27.8. IR 2978.9, 1739.0, 1448.6, 1372.7, 1252.7, 1154.5, 1092.8, 1041.6 cm^{-1}. HRMS (ESI$^+$) (m/z) calcd. for $C_{13}H_{19}O_4S$ [M+H]$^+$ 271.0999; found 271.0989.

Methoxycarbonylmethyl 2-(ethoxycarbonyl)ethyl sulfoxide (8p)

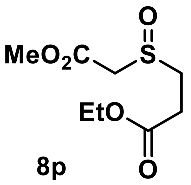

8p

8p was purified by flash column chromatography (ethyl acetate/hexane = 1:2 to 3:1) as a colorless oil (198 mg, 89%). ^{1}H-NMR (400 MHz, $CDCl_3$) δ: 4.15 (q, *J* = 7.0 Hz, 2H), 3.75 (s, 3H), 3.70-3.64 (m, 2H), 3.22-3.00 (m, 2H), 2.81 (t, *J* = 5.6 Hz, 2H), 1.24 (t, *J* = 7.0 Hz, 3H). ^{13}C-NMR (100 MHz, $CDCl_3$) δ: 171.0, 165.4, 61.3, 55.8, 52.9, 47.3, 26.7, 14.1. IR 2973.2, 1725.9, 1432.7, 1380.0, 1247.5, 1182.4, 1014.0, 896.0, 793.0, 582.0 cm^{-1}. HRMS (ESI^{+}) (m/z) calcd. for $C_8H_{15}O_5S$ [M+H]$^{+}$ 223.0635; found 223.0639.

_N_-Fmoc-L-methionine sulfoxide (8q)

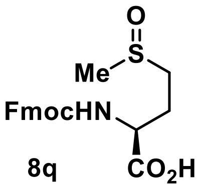

8q

8q (J. Org. Chem. 2021, 86, 14079) was purified by flash column chromatography (ethyl acetate/hexane = 1:1 to ethyl acetate) as a colorless oil (356 mg, 92%). ^{1}H-NMR (400 MHz, DMSO) δ: 7.90-7.87 (m 2H), 7.74-7.69 (m, 2H), 7.43-7.39 (m, 2H), 7.35-7.31 (m, 2H), 4.32-4.30 (m, 2H), 4.25-4.21 (m, 1H), 4.12-4.06 (m, 1H), 2.86-2.62 (m, 2H), 2.54-2.49 (m, 4H), 2.17-1.93 (m, 2H). ^{13}C-NMR (100 MHz, DMSO) δ: 173.7, 173.6, 156.6, 156.5, 144.31, 144.25, 141.2, 128.1, 127.6, 125.7, 120.6, 66.1, 53.8, 53.5, 50.3, 50.1, 47.1, 38.6, 38.3, 24.9, 24.3. IR 3306.0, 3246.7, 2982.7, 2894.9, 2357.5, 1703.4, 1525.4, 1404.7, 1227.1, 1048.2, 996.3, 738.4, 539.7 cm^{-1}. HRMS (ESI^{+}) (m/z) calcd. for $C_{20}H_{22}NO_5S$ [M+H]$^{+}$ 388.1213; found 388.1216.

Tetrahydro-2*H*-thiopyran 1-oxide (8r)

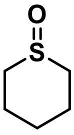

8r

The mixture was diluted by hexane directly and poured onto silica gel for chromatography. **8r** was purified by flash column chromatography (ethyl acetate to ethyl acetate/MeOH = 20:1) as a colorless oil (108 mg, 91%). ^{1}H-NMR (400 MHz, $CDCl_3$) δ: 2.90-2.71 (m, 4H), 2.23-2.20 (m, 2H), 1.66-1.55 (m, 4H). ^{13}C-NMR (100 MHz, $CDCl_3$) δ: 48.9, 24.6, 19.1. HRMS (ESI^{+}) (m/z) calcd. for $C_5H_{11}OS$ [M+H]$^{+}$ 119.0525; found 119.0521.

1,4-Thioxane 4-oxide (8s)

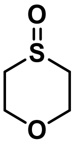

8s

The mixture was diluted by hexane directly and poured onto silica gel for chromatography. **8s** was purified by flash column chromatography (ethyl acetate to ethyl acetate/MeOH = 20:1) as a colorless oil (106 mg, 88%). ^{1}H-NMR (400 MHz, $CDCl_3$) δ: 4.37-4.31 (m, 2H), 3.81-3.76 (m, 2H), 2.93-2.86 (m, 2H), 2.73-2.68 (m, 2H). ^{13}C-NMR (100 MHz, $CDCl_3$) δ: 59.2, 46.4. IR 2924.0, 2866.7, 1713.9, 1650.2, 1460.5, 1389.6, 1269.3, 1213.3, 1152.6, 1095.7, 1057.3 cm^{-1}. HRMS (ESI^{+}) (m/z) calcd. for $C_4H_9O_2S$ [M+H]$^{+}$ 121.0318; found 121.0314.

(*2s,3s*)-2,3-Dihydro-3-hydroxy-2-(4-methoxyphenyl)-1,5-benzothiazepin-4(*5H*)-one 1-oxide (8t)

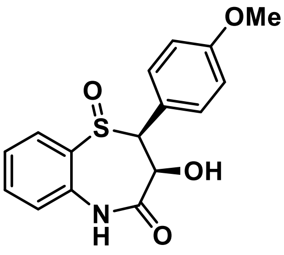

8t

$FeBr_3$ (5.9 mg, 0.02 mmol) was used as the control catalyst according to **General Procedure IV**. **8t** (Org. Biomol. Chem. 2017, 15, 2647) was purified by flash column chromatography (ethyl acetate/hexane = 1:1 to ethyl acetate) as a white solid [Fe(TPY10)Br_3: 257 mg, 81%; $FeBr_3$: 12.7 mg, 4%]. M.P. 222-224°C. ^{1}H-NMR (400 MHz, DMSO) δ: 10.51 (s, 1H), 7.76 (d, J = 7.5 Hz, 1H), 7.64-7.54 (m, 2H), 7.34 (d, J = 7.2 Hz, 2H), 7.23 (d, J = 7.6 Hz, 1H), 7.00 (d, J = 7.2 Hz, 2H), 5.06 (d, J = 5.3 Hz, 1H), 4.64 (d, J = 9.2 Hz, 1H), 4.46 (dd, J = 8.6, 5.3 Hz, 1H), 3.79 (s, 3H). ^{13}C-NMR (100 MHz, DMSO) δ: 171.8, 159.9, 135.5, 134.3, 132.2, 132.0, 127.2, 125.5, 124.8, 123.4, 114.3, 79.1, 65.0, 55.6. IR 3404.5, 3073.6, 2929.1, 2878.5, 1702.0, 1607.2, 1508.7, 1468.3, 1366.3, 1297.6, 1252.3, 1176.3, 1099.5, 1064.7, 1024.2 cm^{-1}. HRMS (ESI$^+$) (m/z) calcd. for $C_{16}H_{16}NO_4S$ [M+H]$^+$ 318.0795; found 318.0785.

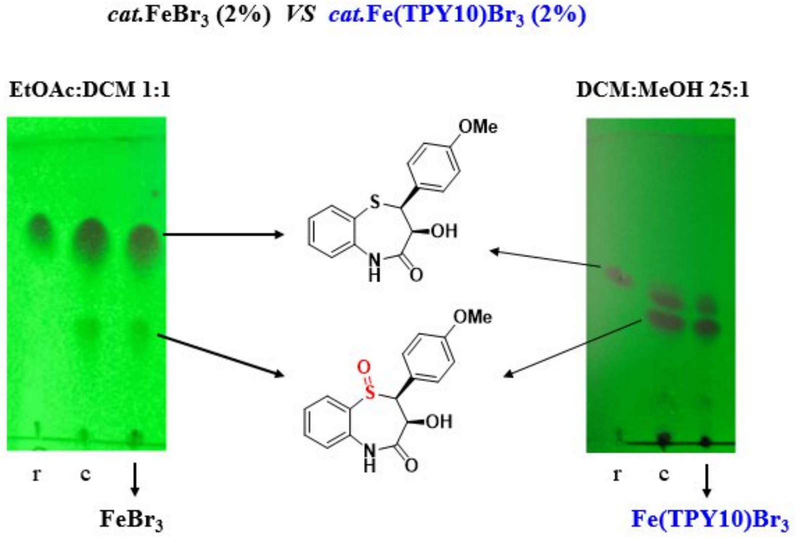

2-(2,6-Diethoxycarbonyl-4,4-difluoro-1,3,5,7-tetramethyl-4-bora-3a,4a-diaza-*s*-indacene-8-yl)phenyl methyl sulfoxide (8u)

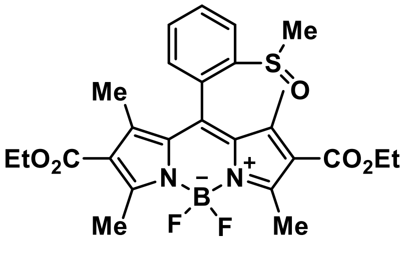

8u

Additional Fe(TPY10)Br_3 (5.6 mg, 0.01 mmol) and H_2O_2 (30%, 0.2 mL, 2 mmol) were needed. **8u** (Anal. Chim. Acta 2013, 800, 71) was purified by flash column chromatography (ethyl acetate/hexane = 1:2 to 3:1) as a dark red solid (398 mg, 75%). M.P. 216-218°C. ^{1}H-NMR (400 MHz, $CDCl_3$) δ: 8.28 (d, J = 8.0 Hz, 1H), 7.87 (t, J = 7.7 Hz, 1H), 7.72 (t, J = 7.5 Hz, 1H), 7.33 (d, J = 7.5 Hz, 1H), 4.30 (q, J = 7.1 Hz, 4H), 2.86 (s, 3H), 2.84 (s, 3H), 2.58 (s, 3H), 1.71 (s, 3H), 1.61 (s, 3H), 1.35-1.30 (m, 6H). ^{13}C-NMR (100 MHz, $CDCl_3$) δ: 164.0, 163.8, 161.9, 159.9, 148.1, 145.4, 144.7, 139.9, 132.3, 131.9, 131.7, 130.8, 128.8, 125.4, 123.6, 122.8, 60.5, 60.4, 42.4, 15.2, 15.1, 14.28, 14.25, 13.8, 13.5. ^{19}F-NMR (376 MHz, $CDCl_3$) δ: -142.51 (q, $^1J_{F\text{-}B}$ = 31.8 Hz), -142.68 (q, $^1J_{F\text{-}B}$ = 31.4 Hz). IR 2992.3, 1697.8, 1470.1, 1398.5, 1310.6, 1004.8, 840.5, 800.8 cm^{-1}. HRMS (ESI$^+$) (m/z) calcd. for $C_{26}H_{30}BF_2N_2O_5S$ [M+H]$^+$ 531.1931; found 531.1917.

9. Catalytic application of Fe(TPY10)Br3 for the thiol oxidation to disulfide.

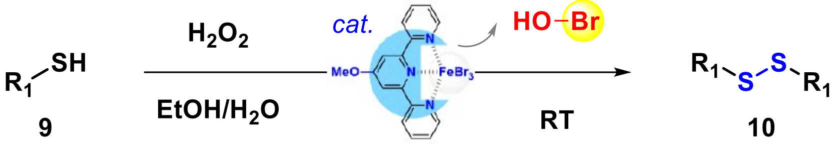

General Procedure V: To a stirred solution of thiol **9** (1 mmol) and Fe(TPY10)Br$_3$ (11.2 mg, 0.02 mmol) in THF (10 mL) was added H_2O_2 (30%, 0.15 mL, 1.5 mmol) dropwise. After completion of the addition, the reaction mixture was stirred at rt for 0.5 h. The reaction was quenched by aqueous $Na_2S_2O_3$ solution (0.1 M, 50 mL) and ethyl acetate (50 mL). The organic fractions were collected, and the aqueous phase was extracted with ethyl acetate (2 ×20 mL). The combined organic fractions were washed with brine, dried over Na $_2$SO$_4$, filtered, and concentrated under reduced pressure. The resulting residue was purified by flash column chromatography to give compound **10**.

Bis(2-aminophenyl) disulfide (10a)

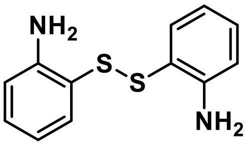

10a

10a was purified by flash column chromatography (ethyl acetate/hexane = 1:15 to 1:5) as a light-yellow solid (117 mg, 94%). M.P. 96-97°C. ^{1}H-NMR (400 MHz, CDCl$_3$) δ: 7.17-7.14 (m, 4H), 6.72 (dd, J = 8.5, 1.3 Hz, 2H), 6.61-6.57 (m, 2H). ^{13}C-NMR (100 MHz, CDCl$_3$) δ: 148.8, 137.0, 131.7, 118.9, 118.4, 115.4. IR 3375.0, 3292.6, 1611.5, 1580.2, 1468.0, 1440.8, 1299.4, 1242.6, 1151.0, 1020.5, 856.2, 543.6, 452.2 cm^{-1}. HRMS (ESI$^+$) (m/z) calcd. for $C_{12}H_{13}N_2S_2$ [M+H]$^+$ 249.0515; found 249.0514.

Bis(3-bromophenyl) disulfide (10b)

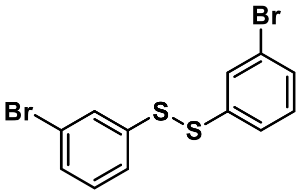

10b

10b was purified by flash column chromatography (ethyl acetate/hexane = 1:15 to 1:5) as a colorless oil (169 mg, 90%). ^{1}H-NMR (400 MHz, CDCl$_3$) δ: 7.64-7.63 (m, 2H), 7.41-7.36 (m, 4H), 7.20-7.16 (m, 2H). ^{13}C-NMR (100 MHz, CDCl$_3$) δ: 138.8, 130.7, 130.6, 130.1, 126.1, 123.3. IR 3051.4, 2922.1, 2852.0, 2738.8, 1665.6, 1560.9, 1485.0, 1452.9, 1394.9, 1318.1, 1288.7, 1256.2, 1194.8, 1163.2, 1067.4, 991.4, 967.7, 867.7, 531.9, 428.0 cm^{-1}. HRMS (ESI$^+$) (m/z) calcd. for $C_{12}H_9Br_2S_2$ [M+H]$^+$ 376.8486; found 376.8483.

Bis(4-chlorophenyl) disulfide (10c)

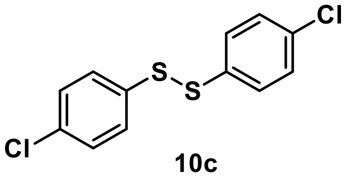

10c

10c was purified by flash column chromatography (ethyl acetate/hexane = 1:15 to 1:5) as a white solid (134 mg, 93%). M.P. 75-76°C. ^{1}H-NMR (400 MHz, CDCl$_3$) δ: 7.42 (d, J = 8.6 Hz, 4H), 7.29 (d, J = 8.6 Hz, 4H). ^{13}C-NMR (100 MHz, CDCl$_3$) δ: 135.3, 133.8, 129.5, 129.4. IR 2920.4, 1564.7, 1439.2, 1248.8, 1109.1, 1022.0, 933.9, 525.8, 485.5 cm^{-1}. GC-MS (m/z = 286.0) and the retrieval result were listed at the end of

SI.

Bis(3,4-dimethoxyphenyl) disulfide (10d)

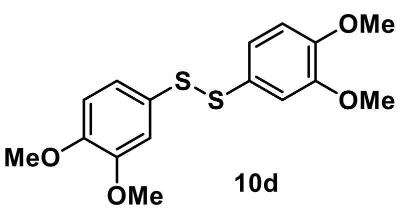

10d

10d was purified by flash column chromatography (ethyl acetate/hexane = 1:15 to 1:5) as a white solid (154 mg, 91%). M.P. 97-98°C. ^{1}H-NMR (400 MHz, $CDCl_3$) δ: 7.06 (dd, *J* = 8.3, 2.1 Hz, 2H), 7.01 (d, *J* = 2.1 Hz, 2H), 6.79 (d, *J* = 8.3 Hz, 2H), 3.86 (s, 6H), 3.82 (s, 6H). ^{13}C-NMR (100 MHz, $CDCl_3$) δ: 149.7, 149.3, 128.8, 124.0, 114.3, 111.5, 56.11, 56.05. IR 2909.1, 2835.4, 1578.4, 1498.3, 1445.1, 1395.3, 1318.2, 1251.7, 1224.4, 1177.3, 1133.8, 1089.0, 1017.4, 841.6, 805.7, 518.4, 456.8 cm^{-1}. HRMS (ESI$^+$) (m/z) calcd. for $C_{16}H_{19}O_4S_2$ [M+H]$^+$ 339.0719; found 339.0717.

Bis(2,6-dimethylphenyl) disulfide (10e)

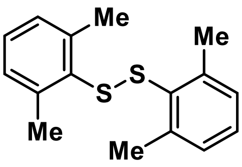

10e

10e was purified by flash column chromatography (ethyl acetate/hexane = 1:15 to 1:5) as a white solid (122 mg, 89%). M.P. 104-105°C. ^{1}H-NMR (400 MHz, $CDCl_3$) δ: 7.13-7.09 (m, 2H), 7.02 (d, *J* = 7.5 Hz, 4H), 2.24 (s, 12H). ^{13}C-NMR (100 MHz, $CDCl_3$) δ: 143.6, 134.9, 129.4, 128.2, 21.6. IR 3051.6, 2915.7, 1571.3, 1452.4, 1372.3, 1160.1, 1031.6, 517.5, 475.7 cm^{-1}. HRMS (ESI$^+$) (m/z) calcd. for $C_{16}H_{19}S_2$ [M+H]$^+$ 275.0923; found 275.0925.

Bis(naphthalene-2-yl) disulfide (10f)

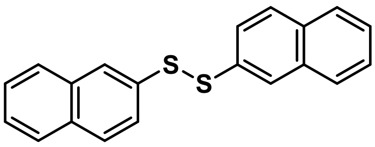

10f

10f was purified by flash column chromatography (ethyl acetate/hexane = 1:15 to 1:5) as a white solid (151 mg, 95%). M.P. 144-145°C. ^{1}H-NMR (400 MHz, $CDCl_3$) δ: 8.00 (d, *J* = 1.9 Hz, 2H), 7.81-7.78 (m, 4H), 7.75-7.73 (m, 2H), 7.65 (dd, *J* = 8.6, 1.9 Hz, 2H), 7.50-7.43 (m, 4H). ^{13}C-NMR (100 MHz, $CDCl_3$) δ: 134.4, 133.6, 132.6, 129.1, 127.9, 127.6, 126.9, 126.7, 126.4, 125.8. IR 3050.4, 2920.5, 2089.4, 1614.8, 1577.1, 1490.3, 1338.9, 1265.8, 1130.9, 952.1, 893.6, 858.8, 809.3, 513.0, 470.6 cm^{-1}. HRMS (ESI$^+$) (m/z) calcd. for $C_{20}H_{15}S_2$ [M+H]$^+$ 319.0610; found 319.0607.

Bis(*n*-hexyl) disulfide (10g)

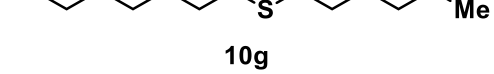

10g

10g was purified by flash column chromatography (ethyl acetate/hexane = 1:15 to 1:5) as a colorless oil (106 mg, 90%). ^{1}H-NMR (400 MHz, $CDCl_3$) δ: 2.69 (t, *J* = 7.4 Hz, 4H), 1.70-1.63 (m, 4H), 1.42-1.25 (m, 12H), 0.90 (t, *J* = 6.3 Hz, 6H). ^{13}C-NMR (100 MHz, $CDCl_3$) δ: 39.3, 31.6, 29.3, 28.3, 22.7, 14.2. IR 2955.1, 2923.9, 2854.6, 1460.7, 1412.7, 1377.8, 1341.9, 1284.3, 1255.7, 1239.0, 1204.2, 1110.9, 1048.0, 986.8, 889.6, 855.0, 808.1, 498.1, 431.0 cm^{-1}. GC-MS (m/z = 234.2) and the retrieval result were listed at the

end of SI.

Bis(2-hydroxyethyl) disulfide (10h)

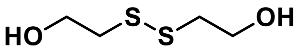

10h

10h was purified by flash column chromatography (ethyl acetate/hexane = 1:15 to 1:5) as a colorless oil (66.3 mg, 86%). ^{1}H-NMR (400 MHz, $CDCl_3$) δ: 3.93 (t, J = 5.8 Hz, 4H), 2.90 (t, J = 5.8 Hz, 4H). ^{13}C-NMR (100 MHz, $CDCl_3$) δ: 60.5, 41.4. IR 3315.2, 2922.1, 2873.2, 1653.0, 1461.4, 1403.9, 1283.5, 1217.5, 1155.9, 1047.4, 1006.6, 938.2, 818.4, 639.5 cm^{-1}. HRMS (ESI$^+$) (m/z) calcd. for $C_4H_{11}O_2S_2$ $[M+H]^+$ 155.0195; found 155.0200.

Bis[2-(methoxycarbonyl)ethyl] disulfide (10i)

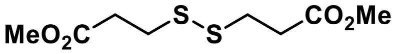

10i

10i was purified by flash column chromatography (ethyl acetate/hexane = 1:15 to 1:5) as a colorless oil (94.1 mg, 79%). ^{1}H-NMR (400 MHz, $CDCl_3$) δ: 3.68 (s, 6H), 2.92 (t, J = 6.6 Hz, 4H), 2.74 (t, J = 7.7 Hz, 4H). ^{13}C-NMR (100 MHz, $CDCl_3$) δ: 172.2, 52.0, 34.0, 33.2. IR 2952.0, 1731.2, 1434.5, 1353.9, 1237.4, 1170.9, 1138.8, 1016.4, 979.6, 890.9, 823.8, 438.8 cm^{-1}. HRMS (ESI$^+$) (m/z) calcd. for $C_8H_{18}NO_4S_2$ $[M+NH_4]^+$ 256.0672; found 256.0682.

Bis(cyclopentyl) disulfide (10j)

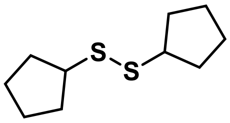

10j

10j was purified by flash column chromatography (ethyl acetate/hexane = 1:15 to 1:5) as a colorless oil (88.0 mg, 87%). ^{1}H-NMR (400 MHz, $CDCl_3$) δ: 3.32-3.26 (m, 2H), 2.00-1.93 (m, 4H), 1.76-1.56 (m, 12H). ^{13}C-NMR (100 MHz, $CDCl_3$) δ: 50.5, 33.3, 24.8. IR 2952.9, 2866.3, 1445.2, 1314.1, 1232.6, 1027.3, 933.0, 898.1 cm^{-1}. GC-MS (m/z = 202.1) and the retrieval result were listed at the end of SI.

Bis(cyclohexyl) disulfide (10k)

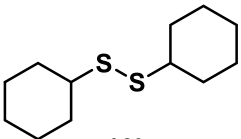

10k

10k was purified by flash column chromatography (ethyl acetate/hexane = 1:15 to 1:5) as a colorless oil (106 mg, 92%). ^{1}H-NMR (400 MHz, $CDCl_3$) δ: 2.71-2.64 (m, 2H), 2.06-2.03 (m, 4H), 1.80-1.76 (m, 4H), 1.63-1.56 (m, 2H), 1.34-1.23 (m, 10H). ^{13}C-NMR (100 MHz, $CDCl_3$) δ: 50.1, 33.0, 26.2, 25.8. IR 2923.8, 2850.0, 1748.0, 1445.4, 1334.6, 1260.9, 1197.3, 1066.5, 996.0, 927.1, 886.5, 854.5, 821.3, 662.1 cm^{-1}. HRMS (ESI$^+$) (m/z) calcd. for $C_{12}H_{23}S_2$ $[M+H]^+$ 231.1236; found 231.1237.

Bis(1-adamantyl) disulfide (10l)

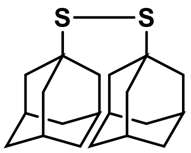

10l

Additional Fe(TPY10)Br_3 (5.6 mg, 0.01 mmol) and H_2O_2 (30%, 0.15 mL, 1.5 mmol) were needed. **10l** was purified by flash column chromatography (ethyl acetate/hexane = 1:15 to 1:5) as a white solid (136 mg, 81%). M.P. 226-227°C. ^{1}H-NMR (400 MHz, $CDCl_3$) δ: 2.07-2.05 (m, 6H), 1.82 (d, *J* = 3.0 Hz, 12H), 1.70-1.63 (m, 12H). ^{13}C-NMR (100 MHz, $CDCl_3$) δ: 47.5, 43.2, 36.3, 30.2. IR 2895.1, 2844.9, 1446.6, 1340.5, 1289.5, 1253.7, 1099.0, 1034.5, 973.6, 808.4 cm^{-1}. HRMS (ESI$^+$) (m/z) calcd. for $C_{20}H_{31}S_2$ [M+H]$^+$ 335.1862; found 335.1873.

_N,N_-Bis(_tert_-butoxy)carbonyl-L-cystine dimethyl ester (10m)

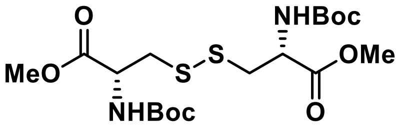

10m

10m was purified by flash column chromatography (ethyl acetate/hexane = 1:15 to 1:5) as a white solid (197 mg, 84%). M.P. 103-104°C. ^{1}H-NMR (400 MHz, $CDCl_3$) δ: 5.40 (d, *J* = 8.2 Hz, 2H), 4.60-4.56 (m, 2H), 3.75 (s, 6H), 3.15 (d, *J* = 5.4 Hz, 4H), 1.43 (s, 18H). ^{13}C-NMR (100 MHz, $CDCl_3$) δ: 171.3, 155.2, 80.4, 52.9, 52.7, 41.4, 28.4. IR 3374.0, 2977.1, 1744.3, 1683.4, 1506.3, 1443.4, 1395.7, 1360.0, 1318.5, 1283.7, 1250.2, 1212.7, 1158.6, 1057.0, 1018.1, 981.8, 921.2, 858.4, 560.7 cm^{-1}. HRMS (ESI$^+$) (m/z) calcd. for $C_{18}H_{33}N_2O_8S_2$ [M+H]$^+$ 469.1673; found 469.1685. $[\alpha]_D^{25}$ = -114 (*c* 1, MeOH).

Bis(pyridin-2-yl) disulfide (10n)

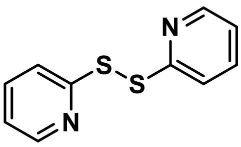

10n

10n was purified by flash column chromatography (ethyl acetate/hexane = 1:2 to 1:1) as a light yellow oil (107 mg, 97%). ^{1}H-NMR (400 MHz, $CDCl_3$) δ: 8.48-8.44 (m, 2H), 7.64-7.57 (m, 4H), 7.15-7.05 (m, 2H). ^{13}C-NMR (100 MHz, $CDCl_3$) δ: 159.0, 149.6, 137.4, 121.1, 119.7. IR 3072.6, 1561.8, 1442.2, 1408.4, 1277.1, 1109.7, 979.8, 751.2, 612.6 cm^{-1}. HRMS (ESI$^+$) (m/z) calcd. for $C_{10}H_9N_2S_2$ [M+H]$^+$ 221.0202; found 221.0205.

10. Gram-scale synthesis for the bromination of aromatic ring (2n) and disulfide synthesis (10b) using _cat._Fe(TPY10)Br_3/H_2O_2 system at rt.

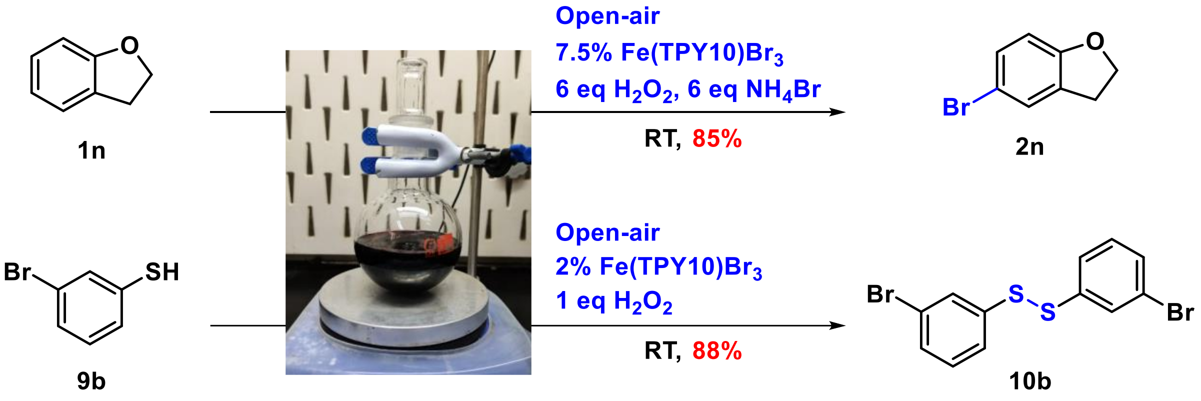

Gram-scale synthesis of 2n: To a stirred solution of **1n** (1.20 g, 10 mmol), NH_4Br (2.94 g, 30 mmol) and catalytic Fe(TPY10)Br_3 (279 mg, 0.5 mmol) in EtOH/H_2O (3/1, 100 mL) was added H_2O_2 (30%, 3 mL,
30

mmol) dropwise. After completion of the addition, the homogenous mixture was stirred vigorously at rt for 2 h followed by the addition of Fe(TPY10)Br_3 (140 mg, 0.25 mmol), NH_4Br (2.94 g, 30 mmol) and H_2O_2 (30%, 3 mL, 30 mmol), and the resulting mixture was further stirred at rt for 1 h. The mixture was quenched by aqueous $Na_2S_2O_3$ (0.2 M, 200 mL) and evaporated under reduced pressure to remove most EtOH, then ethyl acetate (100 mL) was added and mixed. The organic fractions were collected, and the aqueous phase was extracted with ethyl acetate (3 ×50 mL). The combined organic fractions were washed with brine, dried over Na_2SO_4, filtered, and concentrated under reduced pressure. The residue was purified by flash column chromatography (hexane/ethyl acetate = 5:1 to 2:1) to give compound **2n** (1.69 g, 85%).

Gram-scale synthesis of 10b: To a stirred solution of 3-bromothiophenol **9b** (1.89 g, 10 mmol) and Fe(TPY10)Br_3 (112 mg, 0.2 mmol) in THF (100 mL) was added H_2O_2 (30%, 1.5 mL, 15 mmol) dropwise. After completion of the addition, the reaction mixture was stirred at rt for 0.5 h. The mixture was quenched by aqueous $Na_2S_2O_3$ (0.1 M, 200 mL) and evaporated under reduced pressure to remove most THF, then ethyl acetate (100 mL) was added and mixed. The organic fractions were collected, and the aqueous phase was extracted with ethyl acetate (2 × 50 mL). The combined organic fractions were washed with brine, dried over Na_2SO_4, filtered, and concentrated under reduced pressure. The resulting residue was purified by flash column chromatography to give compound **10b** (1.65 g, 88%).

11. Fluorescent probe: Ratio-HOBr synthesis and the detection of in-situ generated HOBr.

11.1 Synthesis of fluorescent probe: Ratio-HOBr.

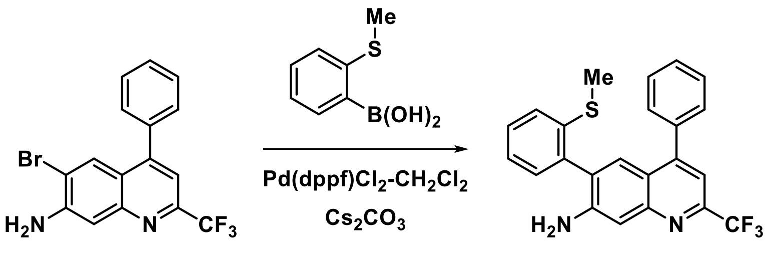

2q Ratio-HOBr

General Procedure: To a stirred solution of brominated quinoline **2q** (95.5 mg, 0.26 mmol) in toluene (3 mL) were added 2-methylthiophenylboronic acid (87.4 mg, 0.52 mmol), Pd(dppf)Cl_2-CH_2Cl_2 (21.2 mg, 0.026 mmol), EtOH (0.05 mL) and 4 M Cs_2CO_3 (0.9 mL) under nitrogen. After completion of the addition, the homogenous mixture was stirred vigorously at 90°C for 12 h. Then, the reaction was quenched by H_2O (50 mL) and ethyl acetate (50 mL). The organic fractions were collected, and the aqueous phase was extracted with ethyl acetate (2 × 20 mL). The combined organic fractions were washed with H_2O, dried over Na_2SO_4, filtered, and concentrated under reduced pressure.

6-(2-Methylthiophenyl)-4-phenyl-2-trifluoromethyl-7-aminoquinoline (Ratio-HOBr)

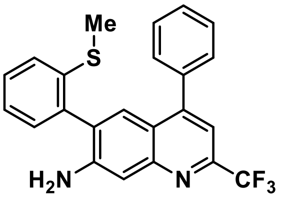

Ratio-HOBr

Ratio-HOBr(*69*) was purified by flash column chromatography (ethyl acetate/hexane = 1:3 to 1:1) as a light-yellow solid (66 mg, 62%). M.P. 126-127°C. ^{1}H-NMR (400 MHz, $CDCl_3$) δ: 7.99 (dd, J = 9.1, 2.1 Hz, 1H), 7.95-7.89 (m, 3H), 7.48-7.40 (m, 2H), 7.39-7.33 (m, 3H), 7.31 (td, J = 6.6, 1.8 Hz, 1H), 7.27 (d, J = 1.6 Hz, 1H), 7.16 (d, J = 9.1 Hz, 1H), 3.91 (brs, 2H), 2.31 (s, 3H). ^{13}C-NMR (101 MHz, $CDCl_3$) δ: 155.4, 148.8, 145.4, 139.4, 138.7, 134.9, 134.2 (q, J = 30.9 Hz), 131.5, 129.5, 128.6, 128.3, 127.2, 125.5, 125.2, 124.5, 122.6 (q, J = 272.9 Hz), 119.7, 119.6, 115.5, 111.3 (q, J = 5.2 Hz), 15.7. IR 3488.7, 3388.8, 2971.8, 2920.8, 1593.2, 1436.0, 1354.8, 1251.5, 1115.0, 743.2, 688.3, 474.7 cm^{-1}. HRMS (ESI^{+}) (m/z) calcd. for $C_{23}H_{18}F_3N_2S$ $[M+H]^+$ 411.1137; found 411.1143.

11.2 Fluorescent probe: Ratio-HOBr for the detection of in-situ generated HOBr.

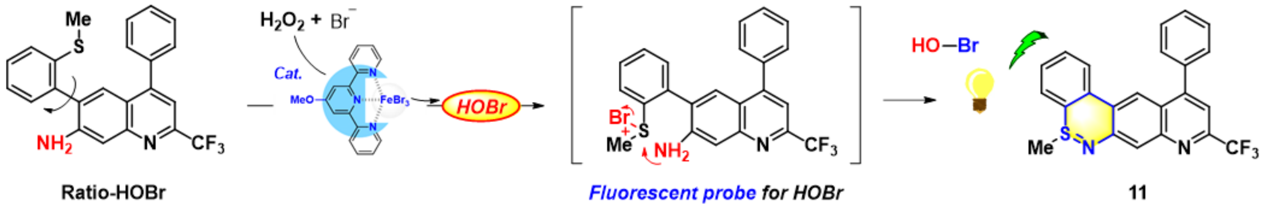

Emission wavelength: 505 nm

Emission wavelength: 545 nm

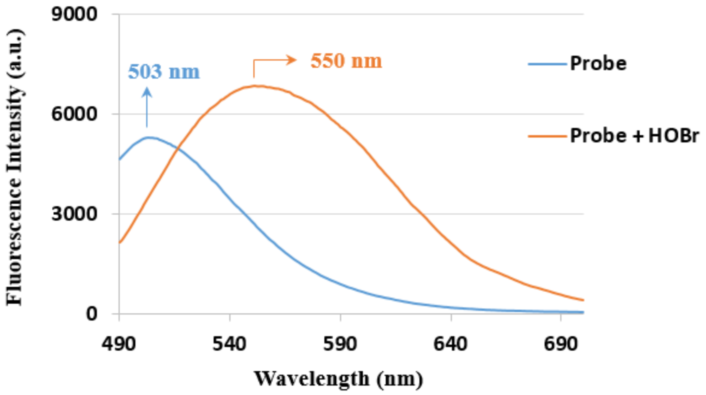

To a stirred solution of fluorescent probe **Ratio-HOBr**(*69*) (20.5 mg, 0.05 mmol) in EtOH/H_2O (3/1, 1 mL) were added Fe(TPY10)Br_3 (1.7 mg, 0.003 mmol) and H_2O_2 (30%, 0.2 mL, 0.2 mmol). After completion of the addition, the reaction mixture was stirred at rt for 1 h. The reaction was quenched by aqueous $Na_2S_2O_3$ solution (0.1 M, 20 mL) and ethyl acetate (20 mL). The organic fractions were collected, and the aqueous phase was extracted with ethyl acetate (2 × 10 mL). The combined organic fractions were washed with H_2O, dried over Na_2SO_4, filtered, and concentrated under reduced pressure. Compound **11** was purified by flash column chromatography (CH_2Cl_2/MeOH = 30:1 to 10:1) as a light-yellow oil (17.6 mg, 86%). ^{1}H-NMR (400 MHz, $CDCl_3$) δ: 9.08 (dd, J = 8.2, 1.2 Hz, 1H), 8.30-8.20 (m, 2H), 8.05 (s, 1H), 7.99 (dq, J = 9.3, 2.2 Hz, 1H), 7.66 (td, J = 6.7, 2.0 Hz, 1H), 7.58-7.46 (m, 6H), 2.37 (s, 3H). ^{13}C-NMR (100 MHz, $CDCl_3$) δ: 155.1, 154.3, 148.4,

138.9, 134.7(q, J = 31.0 Hz), 131.5, 131.4, 131.0, 129.7, 129.3, 129.0, 127.6, 127.3, 124.5, 124.4, 121.3, 119.8(q, J = 274.9 Hz).117.2, 116.6, 111.6(q, J = 5.3 Hz), 28.6. IR 3056.0, 2922.4, 2856.4, 1581.6, 1359.0, 1253.6, 1128.5, 873.6, 763.1, 743.5, 692.4 cm^{-1}. HRMS (ESI^+) (m/z) calcd. for $C_{23}H_{16}F_3N_2S$ $[M+H]^+$ 409.0981; found 409.0984.

The emission spectra of fluorescent probe **Ratio-HOBr** [50 µM in (PBS: 10 mM)/EtOH: 3/2] and **11** [500 µM in (PBS: 10 mM)/EtOH: 3/2] were got under the excitation wavelength of 460 nm, furnishing the emission wavelength of 503 nm (light blue curve) and 550 nm (orange curve) respectively, which were almost identical to the reported values: 505 nm and 545 nm in the literature(*69*).

12. Control experiments for sulfoxidation and the confirmation of HO• and HOO• radical.

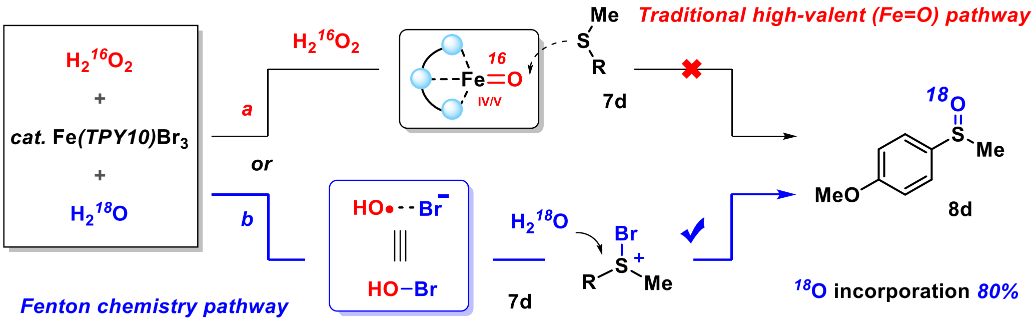

12.1 Isotope labeling experiment:

To a stirred solution of compound **7d** (30.8 mg, 0.2 mmol) and Fe(TPY10)Br_3 (2.2 mg, 0.004 mmol) in EtOH (1.5 mL) and $H_2{}^{18}O$ (0.5 mL) were added 30% H_2O_2 (40 µL, 0.4 mmol). After completion of the addition, the resulting mixture was allowed to stir at rt for 1 h. Then the mixture was quenched by aqueous $Na_2S_2O_3$ solution (0.1 M, 10 mL) and ethyl acetate (10 mL). The organic fractions were collected, and the aqueous phase was extracted with ethyl acetate (3 ×5 mL). The combined organic fractions were washed with brine, dried over Na_2SO_4, filtered, and concentrated under reduced pressure. The residue was purified by flash column chromatography (ethyl acetate/hexane = 1:2 to 2:1) to give compound **8d** (32.0 mg, 93%). HRMS (ESI^+) $C_8H_{11}{}^{18}OS$ $[M+H]^+$ 173.0516 for **^{18}O-8d**, while $C_8H_{11}{}^{16}OS$ $[M+H]^+$ 171.0468 for **^{16}O-8d**.

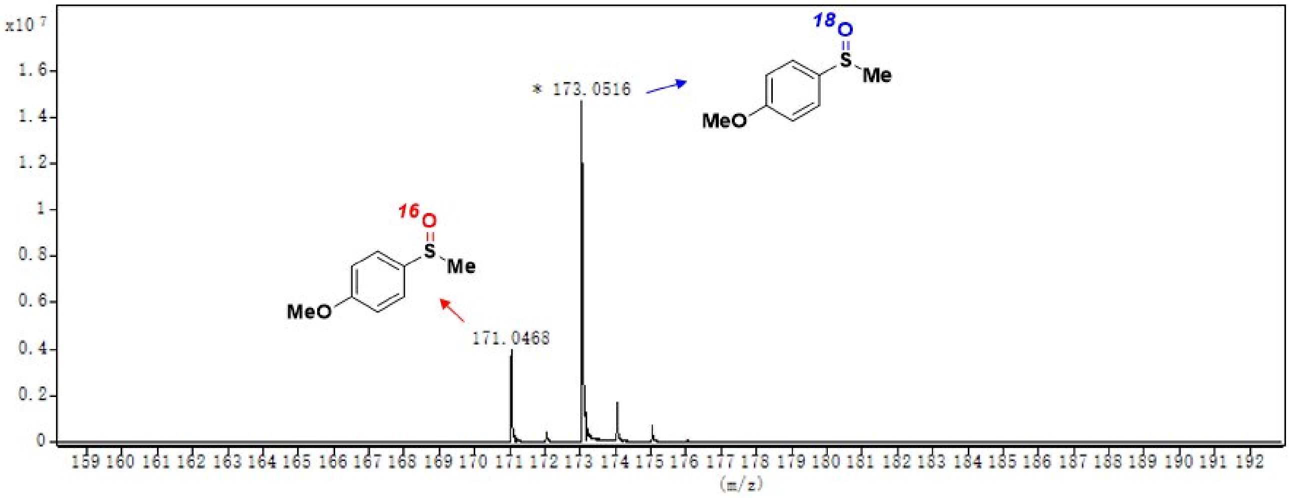

12.2 HO• quenching experiment for sulfoxidation and aromatic bromination by the addition of ABTS [2,2'-azino-bis(3-ethylbenzothiazoline-6-sulfonic acid) diammonium salt]:

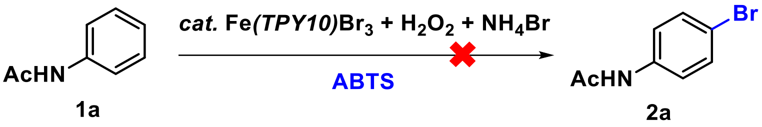

To a stirred solution of aromatic ring **1a** (13.5 mg, 0.1 mmol), NH_4Br (29.4 mg, 0.3 mmol), Fe(TPY10)Br_3 (5.6 mg, 0.01 mmol) and ABTS (165 mg, 0.3 mmol) in EtOH/H_2O (3/1, 1.5 mL) were added H_2O_2 (30%, 30 µL, 0.3 mmol) dropwise, and the resulting solution was stirred at rt for 1.5 h. Then, another NH_4Br (29.4 mg, 0.3 mmol) and H_2O_2 (30%, 30 µL, 0.3 mmol) were added, and the resulting mixture was continuously stirred at rt for 1 h (no product in TLC).

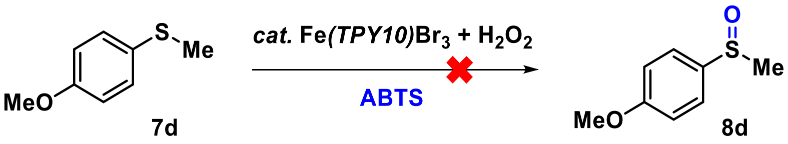

To a stirred solution of sulfide **7d** (15.4 mg, 0.1 mmol) and Fe(TPY10)Br_3 (1.1 mg, 0.002 mmol) and ABTS (110 mg, 0.2 mmol) in EtOH/H_2O (3/1, 1 mL) was added H_2O_2 (30%, 20 µL, 0.2 mmol) dropwise. After completion of the addition, the reaction mixture was stirred at rt for 1 h (TLC showed no product).

12.3 EPR spectrum for Fe(TPY10)Br_3/H_2O_2 system in the presence of DMPO:

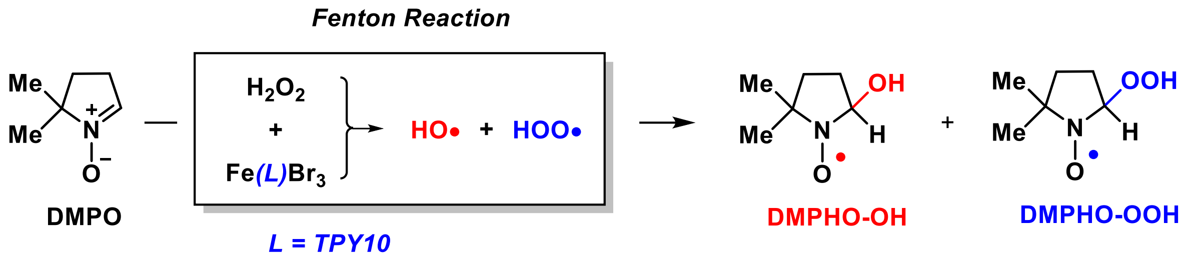

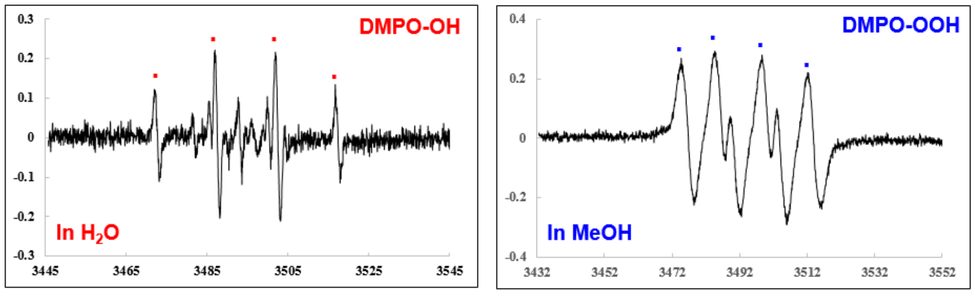

For the HO• radical detection: The above spectra were obtained with a solution containing DMPO (40 mM), H_2O_2 (10 mM) and Fe(TPY10)Br_3 (1 mM) in distilled water. Notably, the EPR spectrum need to be recorded immediately after the addition of H_2O_2 to a solution of Fe(TPY10)Br_3 and DMPO in distilled water.

For the HOO• radical detection: The above spectra were obtained with a solution containing DMPO (40 mM), H_2O_2 (5 mM) and Fe(TYP10)Br_3 (0.2 mM) in MeOH. Notably, the EPR spectrum need to be recorded immediately after the addition of H_2O_2 to a solution of Fe(TPY10)Br_3 and DMPO in MeOH.

Another characteristic Fenton oxidant: hydroperoxyl radical HOO• was further confirmed in the solvent of MeOH with the generation of DMPO-OOH adduct (characteristic 1:1:1:1 peeks) and specific hyperfine coupling constant: $\alpha_N = 13.3$ G, $\alpha^{\beta}_H = 10.4$ G (ACS Omega 2021, 6, 8119; Int. J. Radiat. Appl. Instrum., Part C 1991, 37, 657; J. Phys. Chem. 1978, 82, 1397), fully illustrating the presence of Fenton reaction for our system. Notably, the lifetime of HOO• in aqueous solution appears to be minimal, and DMPO has a very low efficacy to trap hydroperoxyl radical HOO• in aqueous solution. Therefore, efficient spin trapping of superoxide with DMPO was usually carried out in nonaqueous solution (e.g., MeOH) instead of water, generating a longer half-life for the detectable DMPO-OOH adduct (Int. J. Radiat. Appl. Instrum., Part C 1991, 37, 657; J. Phys. Chem. 1978, 82, 1397).

13. Control experiments for the bromination of MCD with different iron catalysts.

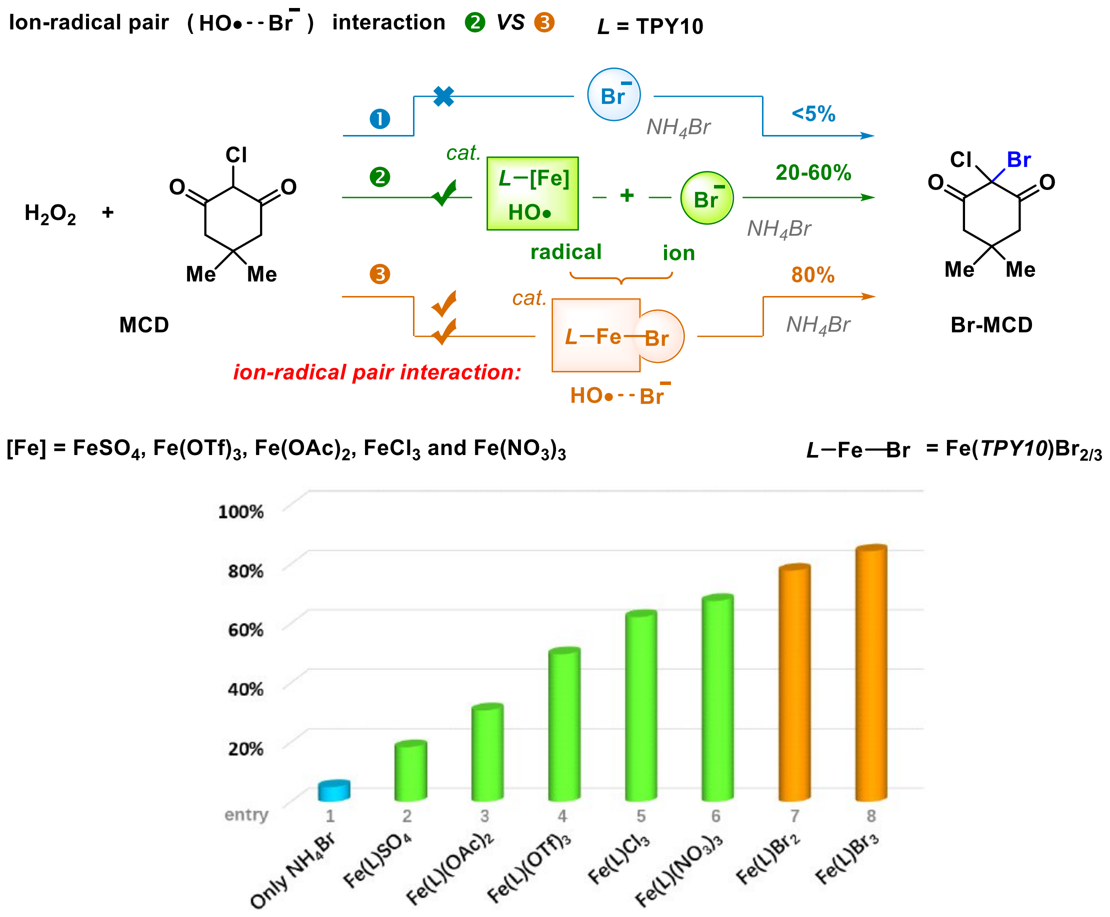

13.1 Iron catalyst screening procedure.

Control experiments was carried out according to the above screening procedure discussed in 2.2: To a stirred solution of TPY10 (52.7 mg, 0.2 mmol) in acetonitrile (MeCN, 4 mL) or H_2O (4 mL) was added different iron salt (0.2 mmol). After completion of the addition, the mixture was allowed to stir overnight from rt to 60°C under nitrogen, accelerating a thorough coordination (detected by TLC). Subsequently, the complex was concentrated and dried under vacuum to furnish a crude iron catalyst with almost quantitative yield (~100%),

for which 5.5 μmol of crude iron catalyst was taken out and transferred to a flask, followed by the addition of MeCN/H_2O (3/1, 40 mL), 2-chloro-5,5-dimethyl-1,3-dimedone (MCD, 19 mg, 0.11 mmol), NH_4Br (54 mg, 0.55 mmol) and H_2O_2 (30%, 55 μL, 0.55 mmol). After completion of the addition, the homogenous mixture was stirred vigorously at rt for 0.5 h. Then, 0.2 mL of the above mixture was taken out, diluted by MeCN/H_2O (3/1, 3.8 mL) and analyzed by UV-Vis spectroscopy immediately. Iron salts: $Fe(OTf)_3$, $FeBr_3$, $FeBr_2$, $FeCl_3$ and $Fe(NO_3)_3$ were coordinated with TPY10 in the solvent of MeCN, while $FeSO_4$ and $Fe(OAc)_2$ were coordinated with TPY10 in the solvent of H_2O.

Notably, the interference of different iron catalysts for UV-Vis spectrum was excluded: UV-Vis absorptions of iron complex [crude iron catalyst (6.88 μM) in MeCN/H_2O (3/1)] were subtracted simultaneously for all the

spectra provided in this paper to suppress the interference of different iron catalyst, and 6.88 μM was identical to the final concentration of diluted iron catalyst following the above procedure.

Table S3. Absorbances at λ_{max} = 293 nm and the conversion yields for the MCD bromination using different iron catalysts

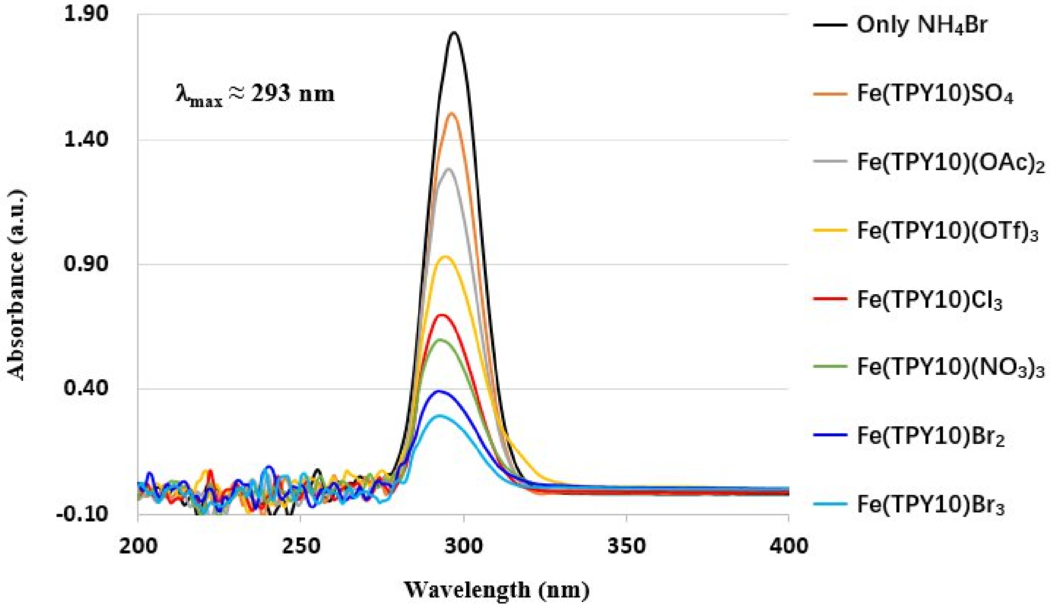

Conversion = (1.85 - Absorbance)/1.85 × 100%

Catalyst	Absorbance	Conversion	Catalyst	Absorbance	Conversion
$Fe(TPY10)Br_3$	0.29	84.3%	$Fe(TPY10)(OTf)_3$	0.93	49.7%
$Fe(TPY10)Br_2$	0.41	77.8%	$Fe(TPY10)(OAc)_2$	1.28	30.8%
$Fe(TPY10)(NO_3)_3$	0.60	67.6%	$Fe(TPY10)SO_4$	1.51	18.4%
$Fe(TPY10)Cl_3$	0.70	62.2%	Only NH_4Br	1.83	1.08%

Absorbances at λ_{max} = 293 nm were given as above, and the conversion yield was calculated: (1.85 - Absorbance)/1.85 × 100%, for which 1.85 was the absorbance at 293 nm without the addition of any catalyst, NH_4Br and H_2O_2 (see above 2.2). Thus, the greater loss of absorbance at 293 nm the higher conversion yield assessed.

13.2 Characterization of different iron catalysts.

All the iron catalysts listed below were synthesized according to the above **control experiments procedure for MCD bromination** using TPY10 (52.7 mg, 0.2 mmol) and different iron salt (0.2 mmol) in the solvent of MeCN (4 mL). Notably, crude iron catalyst without further purification was efficient for the iron salt screening just as reported in the above MCD bromination, but the crude iron coordination was not suitable for further

detail characterization. To furnish neat NMR spectra, iron catalysts were recrystallized from MeCN to eliminate the strong paramagnetism of remaining iron salt. Even though, catalysts **Fe(TPY10)(OTf)$_3$**, **Fe(TPY10)(OAc)$_2$**, **Fe(TPY10)SO$_4$** and **Fe(TPY10)(NO$_3$)$_3$** cannot be locked by ^{1}H-NMR, and all their ^{13}C-NMR cannot be locked owing to the high concentration of iron complex. HRMS, IR and melting point were also carried out for the iron catalysts, and the coordination TLC for iron catalyst **[Fe(TPY10)(OTf)$_3$]** was given below as an example.

Fe[4'-(methoxy)-2,2':6',2"-terpyridine](OTf)$_3$ [Fe(TPY10)(OTf)$_3$]

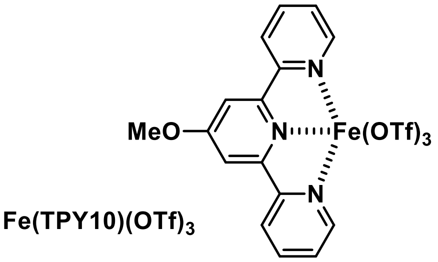

[Fe(TPY10)(OTf)$_3$] was recrystallized from MeCN as a purple solid (133 mg, 87%). M.P. 218-220°C. IR 3084.0, 1600.5, 1565.7, 1485.4, 1442.4, 1370.4, 1277.2, 1253.7, 1218.8, 1161.0, 1024.0, 844.4, 788.9, 633.0 cm^{-1}. HRMS(ESI^{+}) (m/z) calcd. for $C_{18}H_{13}F_6FeN_3O_7S_2$ [M-OTf]$^{+}$ 616.9443; found 616.9445. Elemental analysis (%) calcd. for $C_{19}H_{13}F_9FeN_3O_{10}S_3$: C, 29.78; H, 1.71; N, 5.48; found: C, 29.97; H, 1.64; N, 5.29. μ_{eff} = 4.32 μ_B (D_2O, 300K, Evans method).

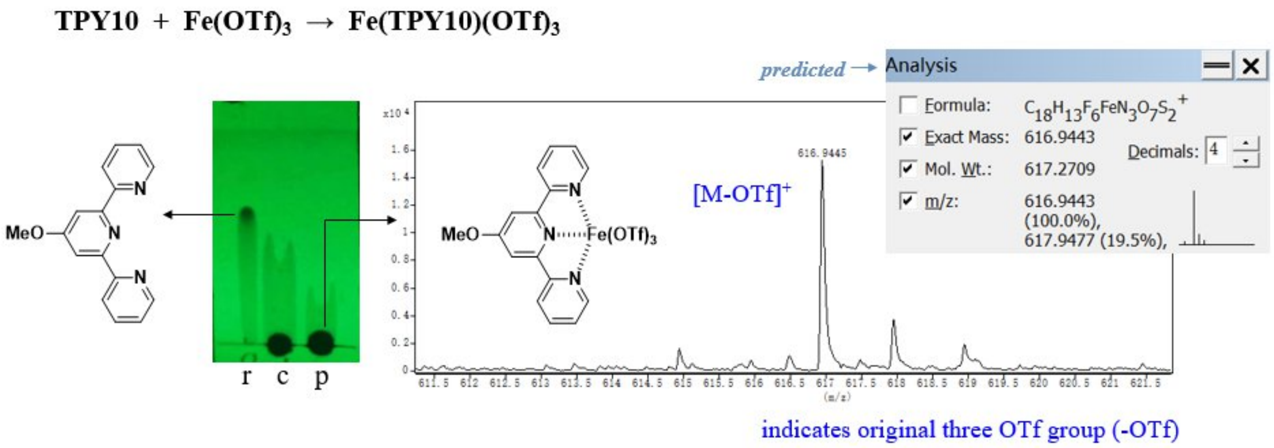

Fe[4'-(methoxy)-2,2':6',2"-terpyridine]Br$_2$ [Fe(TPY10)Br$_2$]

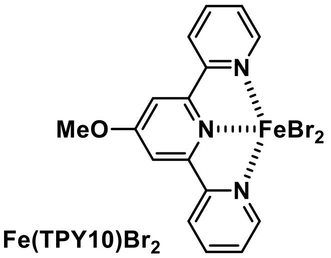

[Fe(TPY10)Br$_2$] was recrystallized from MeCN as a black solid (87.2 mg, 91%). M.P. > 350°C. ^{1}H-NMR (400 MHz, DMSO) δ: 8.94 (s, 2H), 8.85 (d, J = 8.0 Hz, 2H), 7.98 (t, J = 7.7 Hz, 2H), 7.20 (dd, J = 16.2, 6.2 Hz, 4H), 4.38 (s, 3H). IR 3370.2, 3056.7, 1606.7, 1558.2, 1473.8, 1437.4, 1405.2, 1364.4, 1221.4, 1184.4, 1155.8, 1014.7, 822.9, 790.7, 753.1 cm^{-1}. HRMS (ESI^{+}) (m/z) calcd. for $C_{16}H_{13}BrFeN_3O$ [M-Br]$^{+}$ 397.9586; found 397.9590. Elemental analysis (%) calcd. for $C_{16}H_{13}Br_2FeN_3O$: C, 40.12; H, 2.74; N, 8.77; found: C, 40.22; H, 2.71; N, 8.83. μ_{eff} = 2.60 μ_B (D_2O, 300K, Evans method).

Fe[4'-(methoxy)-2,2':6',2"-terpyridine]Cl$_3$ [Fe(TPY10)Cl$_3$]

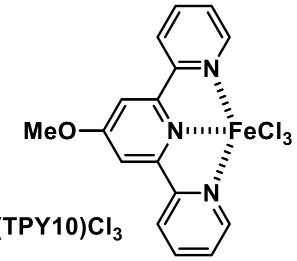

Fe(TPY10)Cl$_3$

[Fe(TPY10)Cl$_3$] was purified by wash with ethyl acetate as a brown solid. (73.2 mg, 86%). M.P. > 350°C. ^{1}H-NMR (400 MHz, DMSO) δ: 8.72 (brs, 2H), 8.62 (brs, 2H), 7.98 (brs, 4H), 7.50 (brs, 2H), 4.01 (s, 3H). IR 3368.9, 3069.4, 1602.6, 1562.4, 1480.9, 1439.2, 1367.8, 1305.8, 1229.2, 1160.4, 1056.8, 1026.7, 840.2, 796.4, 749.9 cm^{-1}. HRMS (ESI$^+$) (m/z) calcd. for $C_{16}H_{13}Cl_2FeN_3O$ [M-Cl]$^+$ 388.9780; found 388.9779. Elemental analysis (%) calcd. for $C_{16}H_{13}Cl_3FeN_3O$: C, 45.17; H, 3.08; N, 9.88; found: C, 45.34; H, 3.11; N, 9.97. μ_{eff} = 5.79 μ_B (D_2O, 300K, Evans method).

Fe[4'-(methoxy)-2,2':6',2"-terpyridine](OAc)$_2$ [Fe(TPY10)(OAc)$_2$]

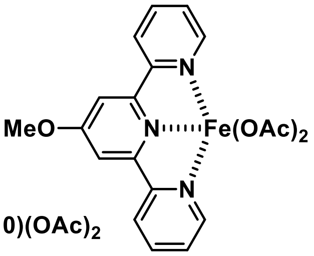

Fe(TPY10)(OAc)$_2$

H_2O was used as the solvent for coordination. **[Fe(TPY10)(OAc)$_2$]** was purified by wash with ethyl acetate as a purple solid (73.5 mg, 84%). M.P. 198-200°C. IR 3410.2, 3076.2, 1604.6, 1542.2, 1483.8, 1414.5, 1369.7, 1227.9, 1164.5, 1026.6, 795.2, 650.5, 618.9 cm^{-1}. HRMS (ESI$^+$) (m/z) calcd. for $C_{18}H_{16}FeN_3O_3$ [M-OAc]$^+$ 378.0536; found 378.0540. Elemental analysis (%) calcd. for $C_{20}H_{19}FeN_3O_5$: C, 54.94; H, 4.38; N, 9.61; found: C, 55.12; H, 4.29; N, 9.72. μ_{eff} = 1.96 μ_B (D_2O, 300K, Evans method).

Fe[4'-(methoxy)-2,2':6',2"-terpyridine]SO$_4$ [Fe(TPY10)SO$_4$]

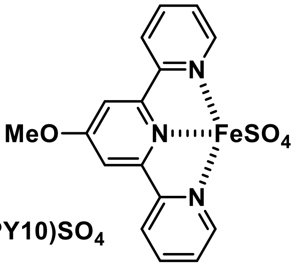

Fe(TPY10)SO$_4$

H_2O was used as the solvent for coordination. **[Fe(TPY10)SO$_4$]** was purified by wash with ethyl acetate as a purple solid (78.9 mg, 95%). M.P. > 350°C. IR 3167.9, 3063.5, 2116.6, 1940.4, 1651.0, 1607.5, 1551.8, 1471.1, 1404.4, 1297.5, 1217.4, 1057.0, 791.7, 752.5, 610.8 cm^{-1} HRMS (ESI$^+$) (m/z) calcd. for $C_{16}H_{13}FeN_3O$ [M-SO$_4$]$^{2+}$ 159.5199; found 159.5199. Elemental analysis (%) calcd. for $C_{16}H_{13}FeN_3O_5S$: C, 46.29; H, 3.16; N, 10.12; found: C, 46.33; H, 3.25; N, 10.27. μ_{eff} = 4.19 μ_B (D_2O, 300K, Evans method).

Fe[4'-(methoxy)-2,2':6',2"-terpyridine](NO$_3$)$_3$ [Fe(TPY10)(NO$_3$)$_3$]

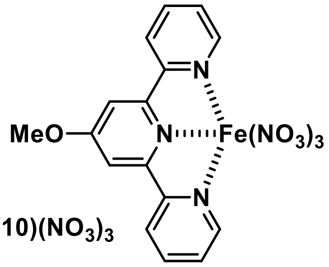

Fe(TPY10)(NO$_3$)$_3$

[Fe(TPY10)(NO$_3$)$_3$] was purified by wash with ethyl acetate as a brown solid (94.0 mg, 93%). M.P. > 350°C. IR 3070.1, 1604.8, 1567.1, 1484.8, 1438.9, 1370.0, 1286.2, 1229.3, 1160.1, 1059.8, 1020.0, 847.8, 791.9, 724.3 cm^{-1}. HRMS (ESI$^+$) (m/z) calcd. for $C_{16}H_{13}FeN_5O_7$ [M-NO$_3$]$^+$ 443.0159; found 443.0151. Elemental analysis (%) calcd. for $C_{16}H_{13}FeN_6O_{10}$: C, 38.04; H, 2.59; N, 16.64; found: C, 38.21; H, 2.47; N, 16.58. μ_{eff} = 3.04 μ_B (D_2O, 300K, Evans method).

14. Cyclic voltammetry (CV) determination for the Fe(TPY10)Br_3 complex.

Cyclic voltammetry (CV) was measured by a CHI 660e electrochemical workstation (Shanghai Chen Hua Instrument Co., Ltd.) in EtOH/H_2O (v/v, 3/1) containing 0.10 M Bu_4NPF_6 ($TBAPF_6$) as a supporting electrolyte. A conventional three-electrode cell was used with glassy carbon as a working electrode, platinum wire as a counter electrode and saturated calomel electrode as a reference electrode. CV of iron complex [Fe(TPY10)Br_3] (1.0 mM) was listed as below with the scan rate of 100 mV/s.

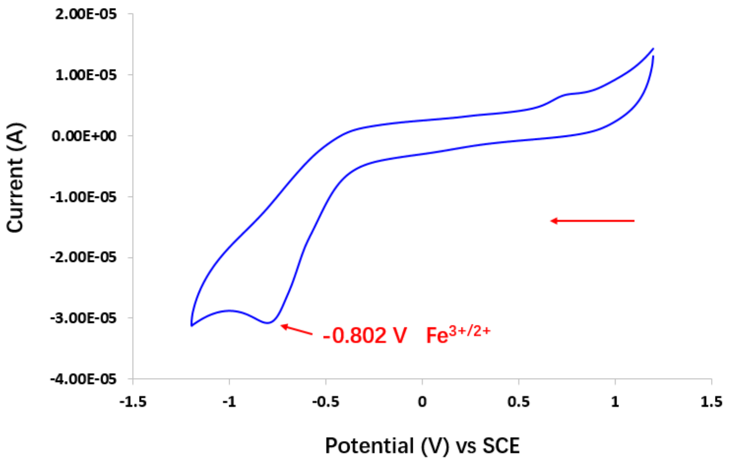

First scan from high to low voltage (+1.2 V → -1.2 V) followed by the reverse form, for which Fe^{3+} was reduced to Fe^{2+} first with the reduction peak at **-0.802 V vs SCE** (Fe^{3+}→Fe^{2+}). However, no observable return oxidation peak E_{pa} was detected (irreversible wave form), which is not possible for the formal one-electron reduction potential determination (Fe^{3+}/Fe^{2+}) of [Fe(TPY10)Br_3]. Considering the relative low reduction peak (**-0.802 V vs SCE**) for Fe^{3+}/Fe^{2+} versus high oxidation peak for sulfide oxidation (+1.0 V to +1.5 V vs SCE for S → S=O/S-S) (Eur. J. Org. Chem. 2021, 2021, 3743; Electrochem. Commun. 2019, 109, 106583) and bromide oxidation (+0.85 V vs SCE for Br^0/Br^{-1}) (Water 2020, 12, 102), we speculate that this Fe^{3+}/Fe^{2+} [Fe(TPY10)Br_3] reduction potential may not affect the oxidation of sulfide or bromide. Whereas, hydroxyl radical HO· was a very strong oxidant (potential: 2.80 V) (Water 2020, 12, 102), which is more competitive for the efficient oxidation of bromide (Br^- → Br^+) as indicated in previous literatures (J. Phys. Chem. 1972, 76, 312; J. Phys. Chem. A 2013, 117, 877).

15. Mass spectrum detection and analysis for the catalytic process of [Fe(TPY10)Br_3].

To a stirred solution of *N*-phenylacetamide (135 mg, 1 mmol), NH_4Br (294 mg, 3 mmol) and Fe(TPY10)Br_3 (55.8 mg, 0.1 mmol) in EtOH/H_2O (3/1, 10 mL) was added H_2O_2 (30%, 0.3 mL, 3 mmol) dropwise. After completion of the addition, the homogenous mixture was stirred vigorously, and the mixture was injected directly into readily available mass spectrometer for our laboratory use (TSQ Quantum Access MAX triple quadrupole, Thermo Fisher Scientific). The representative spectra, proposed structure and corresponding

quasimolecular ion are all listed below for the assisting explanation of intermediates shown in Fig. 6E in the Manuscript.

Mass spectrum for intermediate **II** (Fig. 6E in the Manuscript) with the loss of its corresponding bromide (-Br^-) ion and intermediate **VIII** (Fig. 6E in the Manuscript) with its quasimolecular ion for H^+ addition, for which both are detected at 353.2.

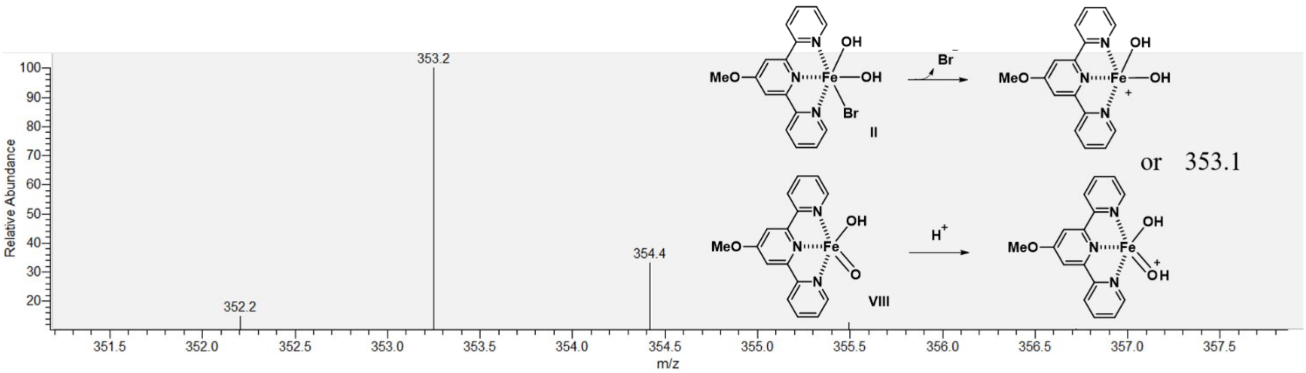

Mass spectrum for intermediate **IV** (Fig. 6E in the Manuscript) and its corresponding quasimolecular ion with H^+ addition (387.1).

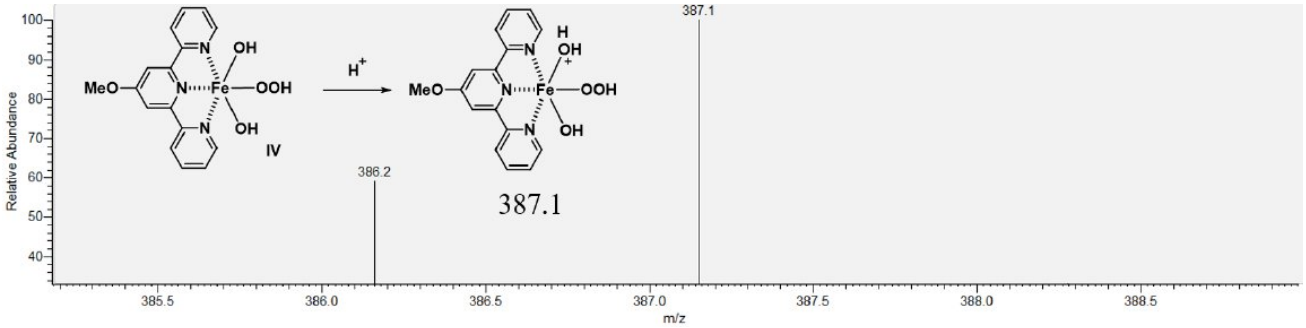

Mass spectrum for intermediates **III** (Fig. 6E in the Manuscript) with the loss of its corresponding bromide (-Br^-) ion: 369.2 and intermediate **V** with its quasimolecular ion for H^+ addition: 371.0.

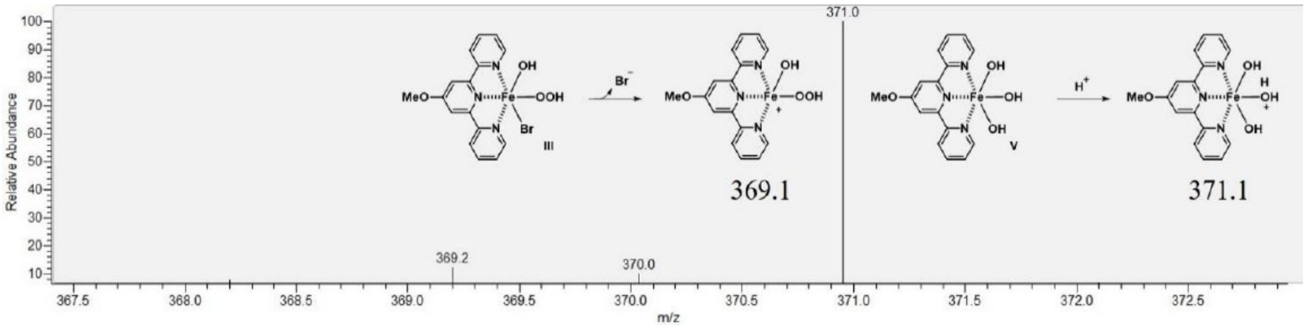

16. Hammett analysis of the electrophilic bromination reaction.

To a stirred solution of aromatic ring **1** (1 mmol), NH_4Br (588 mg, 6 mmol) and Fe(TPY10)Br_3 (55.8 mg, 0.1 mmol) in EtOH/H_2O (3/1, 10 mL) was added H_2O_2 (30%, 0.6 mL, 6 mmol) in one potion. Aliquots of the reaction mixture (100 μL) were taken via syringe at 1, 5, and 10 s (for **1b** and **1e**) or 1, 5, and 10 min (for **1a**

and **1s**) periodically, quenched by $Na_2S_2O_3$ solution (12.7 mM in EtOH/H_2O 3/1, 10 mL) and analyzed by HPLC method (Food Chem. 2011, 128, 223) immediately to determine the crude yield for **2** throughout the process.

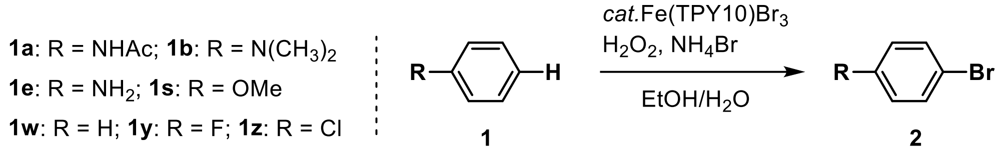

Reactions were performed in triplicate and the average yields with kinetic data are listed below. Notably, no reaction occurred for R = H: **1w**, R = F: **1y** and R = Cl: **1z**, even with overnight stirring.

Table S4. Kinetic data for the bromination of different substrates

**1b**: R = $N(CH_3)_2$		**1e**: R = NH_2		**1s**: R = OMe		**1a**: R = NHAc	
Time (min)	**2b** (%)	Time (min)	**2e** (%)	Time (min)	**2s** (%)	Time (min)	**2a** (%)
0	0	0	0	0	0	0	0
0.0167	6.4	0.0167	4.5	1	9.2	1	7.3
0.0833	18.8	0.0833	14.7	5	28.6	5	24.5
0.1667	29.9	0.1667	22.3	10	45.4	10	38.1

The crude yield of product **2** was then plotted against time to get the rate constant (J. Am. Chem. Soc. 2017, 139, 2484) k_{obs} for each substituent (R = $N(CH_3)_2$, NH_2, OMe or NHAc) as described below.

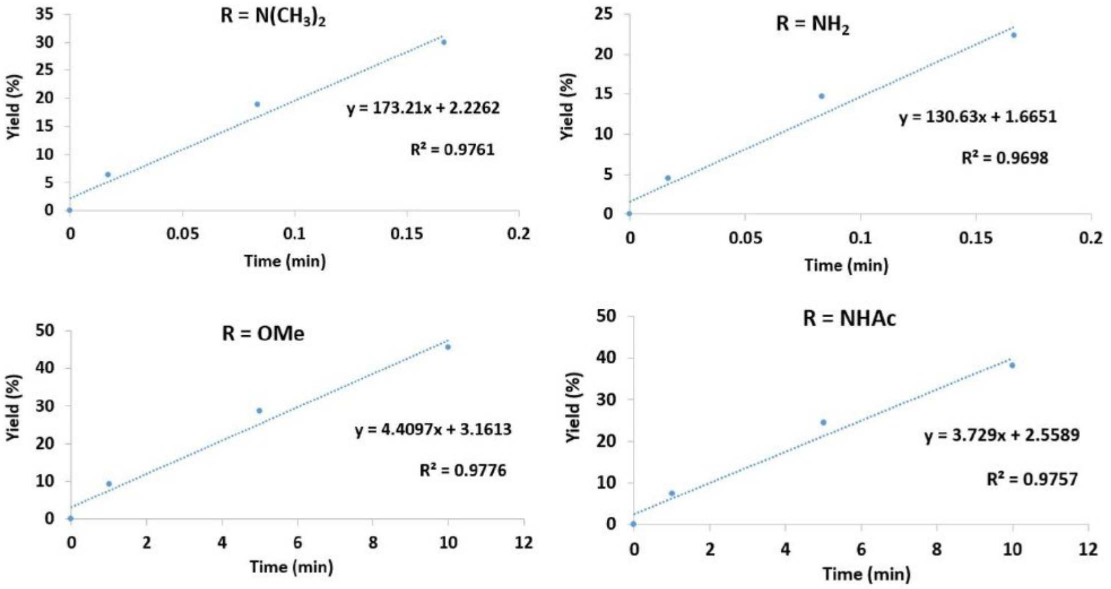

Figure S5. Plots of yields for different substrates vs time

The observed reaction rate constants (k_{obs}) were derived from the slopes of aforementioned fitted lines: k_{obs} = 173.2 min^{-1} for R = $N(CH_3)_2$: **2b**, k_{obs} = 130.6 min^{-1} for R = NH_2: **2e**, k_{obs} = 4.410 min^{-1} for R = OMe: **2s**, and

k_{obs} = 3.729 min^{-1} for R = NHAc: **2a**. Observed rate constants k_{obs} for catalytic bromination with different substituents and their Hammett constants σ_p for Hammett plot analysis are listed below.

Table S5. Tabular data used to construct the Hammett plot

Substrate	σ_p	k_{obs} (min^{-1})	Log k_{obs}
R = $N(CH_3)_2$	-0.83	173.2	2.24
R = NH_2	-0.66	130.6	2.12
R = OMe	-0.27	4.410	0.640
R = NHAc	-0.15	3.729	0.572
R = H	0	No Reaction	
R = F	0.06	No Reaction	
R = Cl	0.23	No Reaction	

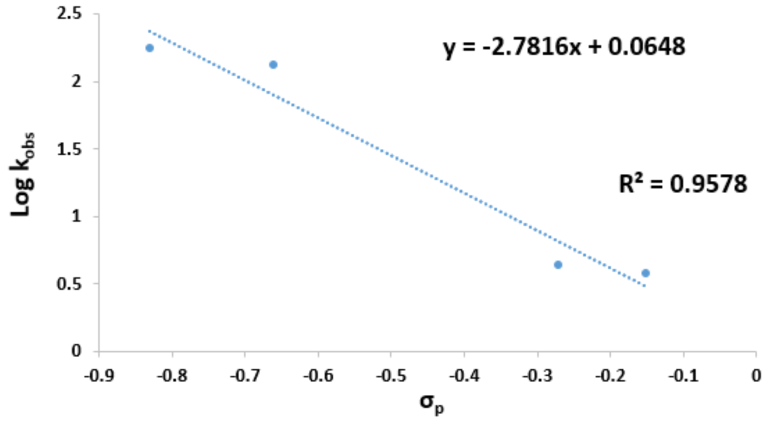

Figure S6. Hammett plots for the bromination of different substrates

No reaction occurred for the electron-withdrawing substituent (e.g., R = F: σ_p = 0.06 or R = Cl: σ_p = 0.23) and neutral ones (H: σ_p = 0) even with overnight stirring. Therefore, the rate constant k_{obs} for R = H (i.e., k_H) and its ratio with other substituents (i.e., k_X/k_H) cannot be furnished, and the Log k_{obs} was plotted against Hammett constant (Chem. Rev. 1991, 91, 165) σ_p directly, which is similar to previous literature (J. Am. Chem. Soc. 2011, 133, 8317). We found that the more electron-donating effect of substituent ($N(CH_3)_2$ > NH_2 > OMe > NHAc), the faster rate constant Log k_{obs} will be furnished (2.24 > 2.12 > 0.640 > 0.572). Namely, log k_{obs} showed a linear dependence on substitute constants σ_p, and the Hammett slope ρ is calculated to be -2.78. Negative value illustrates the formation of cationic transition state: Ar^+-Br as rate-limiting step and the electrophilic nature of in-situ generated bromine species (Chem. Rev. 1991, 91, 165) (i.e., HOBr). Aromatic ring with EDG (e.g., R = $N(CH_3)_2$, R = NH_2, R = OMe, R = NHAc) reacted much faster than those with EWG (e.g., R = F/Cl/H), which is consistent with the negative value of Hammett slope (ρ = –2.78).

17. Characterization of iron complex after catalysis using EPR and Mössbauer spectrum.

To a stirred solution of *N*-phenylacetamide (270 mg, 2 mmol), NH_4Br (588 mg, 6 mmol) and Fe(TPY10)Br_3 (112 mg, 0.2 mmol) in EtOH/H_2O (3/1, 20 mL) was added H_2O_2 (30%, 0.6 mL, 6 mmol) dropwise. After completion of the addition, the reaction mixture was allowed to stir at room temperature (rt) for 1.5 h. Then, additional NH_4Br (588 mg, 6 mmol) and H_2O_2 (30%, 0.6 mL, 6 mmol) were added, and the resulting mixture was continuously stirred at rt for another 1 h. The resulting mixture was concentrated under reduced pressure, followed by gradient elution (hexane/ethyl acetate, ethyl acetate and MeOH system) through a thin layer of silica gel to remove the bromide source (i.e., **2a**: *N*-(4-bromophenyl)acetamide and remaining NH_4Br). The obtained crude high-polar iron complex mixture was detected by EPR and Mössbauer spectrum immediately.

The final oxidation state of non-heme iron complex Fe(TPY10)Br_3 after catalysis was recorded by EPR spectrum at 110 K, and the microwave frequency was kept at 9.3276 GHz.

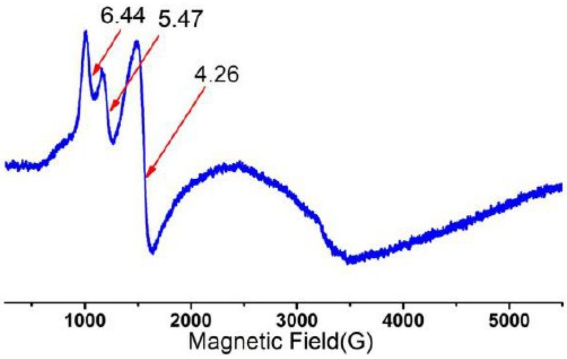

The EPR spectrum exhibit resonance signals with effective *g* values at g = 4.26, g = 5.47 and g = 6.44, illustrating the final oxidation state of iron complex after catalysis to be Fe(III), particularly for g = 4.26, which is an evident signal for the presence of high-spin Fe(III) according to previous literatures(*72-74*).

The final oxidation state of non-heme iron complex Fe(TPY10)Br_3 after catalysis was further detected by Zero-field ^{57}Fe Mössbauer spectrum at 80K. ^{57}Fe Mössbauer spectrum was recorded on a conventional spectrometer with alternating constant acceleration of the γ-source (^{57}Co/Rh, 0.925 GBq). The sample temperature was maintained constant in a liquid nitrogen Mössbauer cryostat (80 K) produced by Cryo Industries of American Inc. Isomer shifts are quoted relative to iron metal at 300 K. The zero-field spectra were simulated with Lorentzian doublets with the program *mf.SL* developed by Dr. Eckhard Bill at the MPI CEC. The data (dots) and best fit (solid line) are listed below.

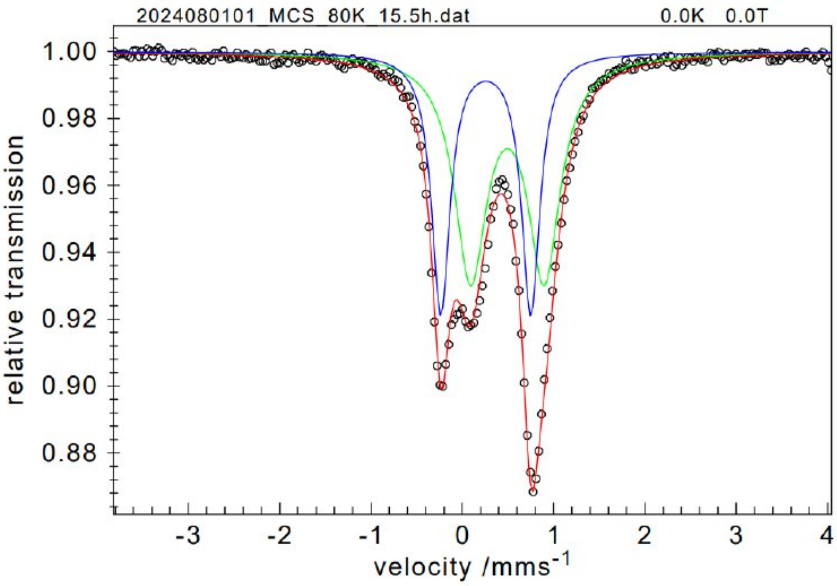

Final iron complex	δ (mm/s)	ΔE_Q (mm/s)	Γ (mm/s)
Sub-1 (green line)	0.49	0.80	0.43
Sub-2 (blue line)	0.26	0.98	0.24

According to the above data of Mössbauer and EPR spectra, we speculate that two high-spin iron(III) species of different rhombicity exist at the end of catalysis, which are geometrically different referring to previous literatures(*74-76*).

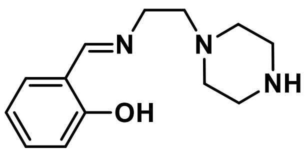

L14

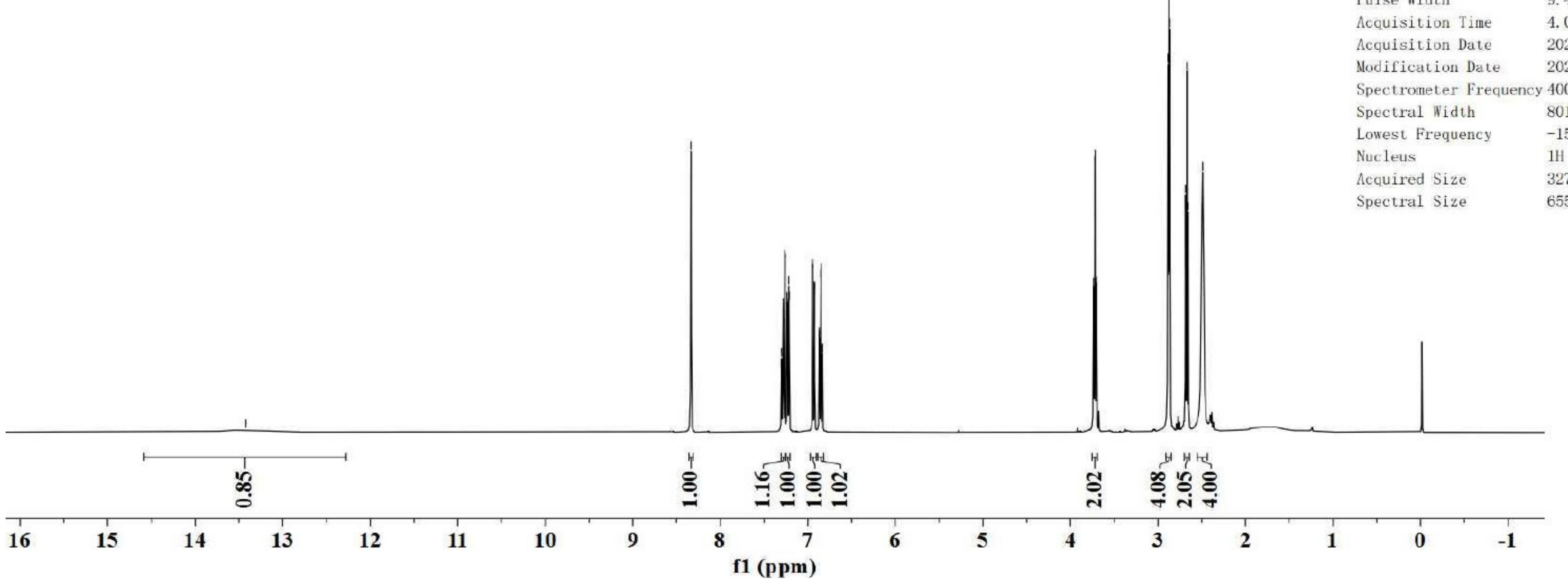

Parameter	Value
Title	DHL-B-53.1.fid
Origin	Bruker BioSpin GmbH
Owner	nmrsu
Instrument	spect
Solvent	CDCl3
Temperature	298.0
Pulse Sequence	zg30
Experiment	1D
Number of Scans	16
Receiver Gain	53.4
Relaxation Delay	1.0000
Pulse Width	9.4300
Acquisition Time	4.0894
Acquisition Date	2023-05-05T17:30:30
Modification Date	2023-05-05T17:30:32
Spectrometer Frequency	400.15
Spectral Width	8012.8
Lowest Frequency	-1546.0
Nucleus	1H
Acquired Size	32768
Spectral Size	65536

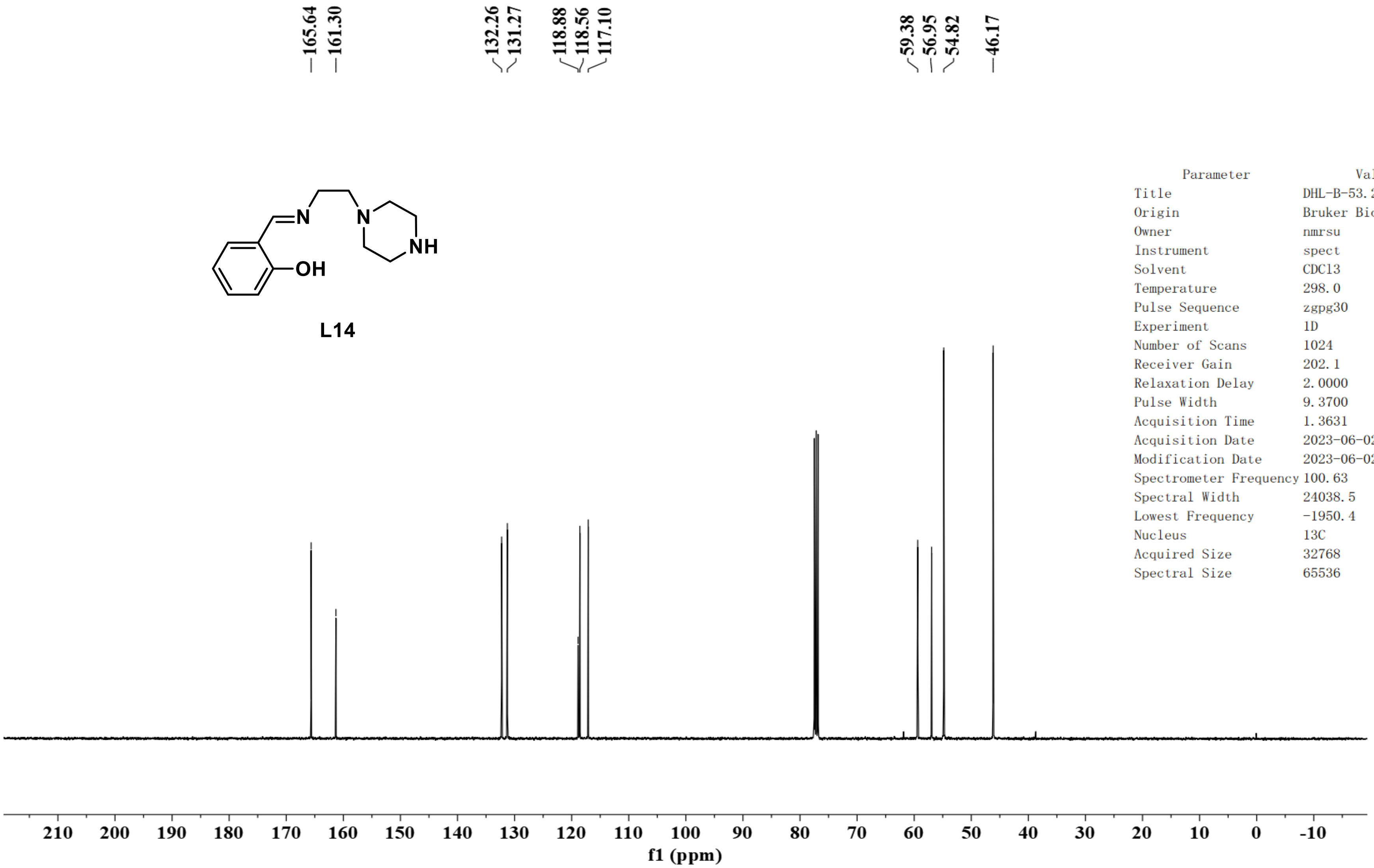

Parameter	Value
Title	DHL-B-53.2.fid
Origin	Bruker BioSpin GmbH
Owner	nmrsu
Instrument	spect
Solvent	CDCl3
Temperature	298.0
Pulse Sequence	zgpg30
Experiment	1D
Number of Scans	1024
Receiver Gain	202.1
Relaxation Delay	2.0000
Pulse Width	9.3700
Acquisition Time	1.3631
Acquisition Date	2023-06-02T01:04:06
Modification Date	2023-06-02T01:04:08
Spectrometer Frequency	100.63
Spectral Width	24038.5
Lowest Frequency	-1950.4
Nucleus	13C
Acquired Size	32768
Spectral Size	65536

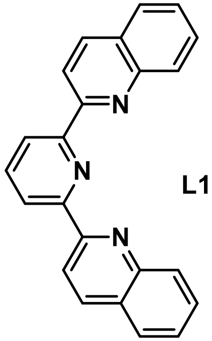

L16

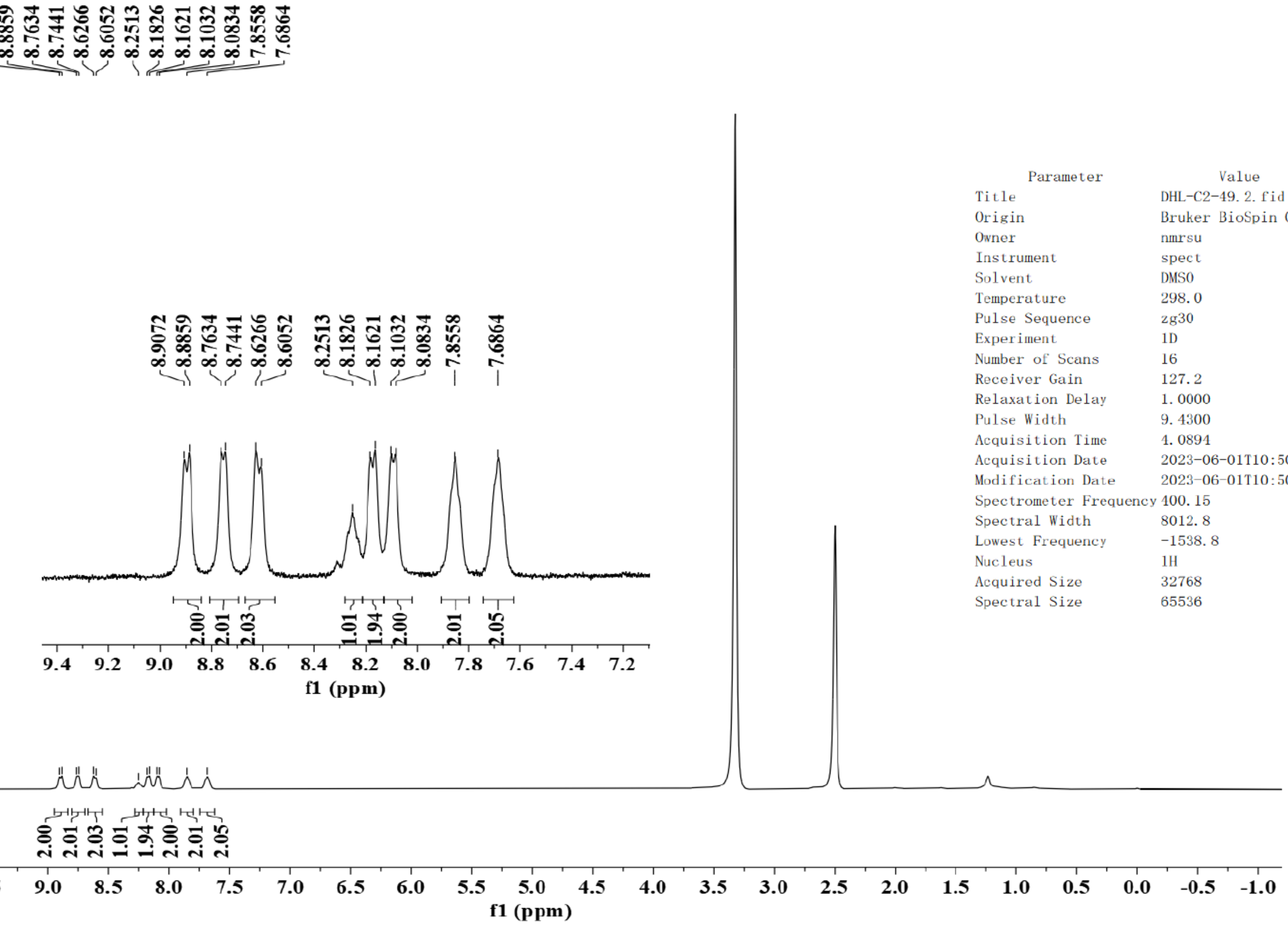

Parameter	Value
Title	DHL-C2-49.2.fid
Origin	Bruker BioSpin GmbH
Owner	nmrsu
Instrument	spect
Solvent	DMSO
Temperature	298.0
Pulse Sequence	zg30
Experiment	1D
Number of Scans	16
Receiver Gain	127.2
Relaxation Delay	1.0000
Pulse Width	9.4300
Acquisition Time	4.0894
Acquisition Date	2023-06-01T10:50:30
Modification Date	2023-06-01T10:50:32
Spectrometer Frequency	400.15
Spectral Width	8012.8
Lowest Frequency	-1538.8
Nucleus	1H
Acquired Size	32768
Spectral Size	65536

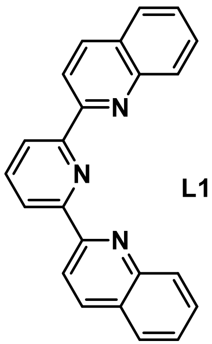

L16

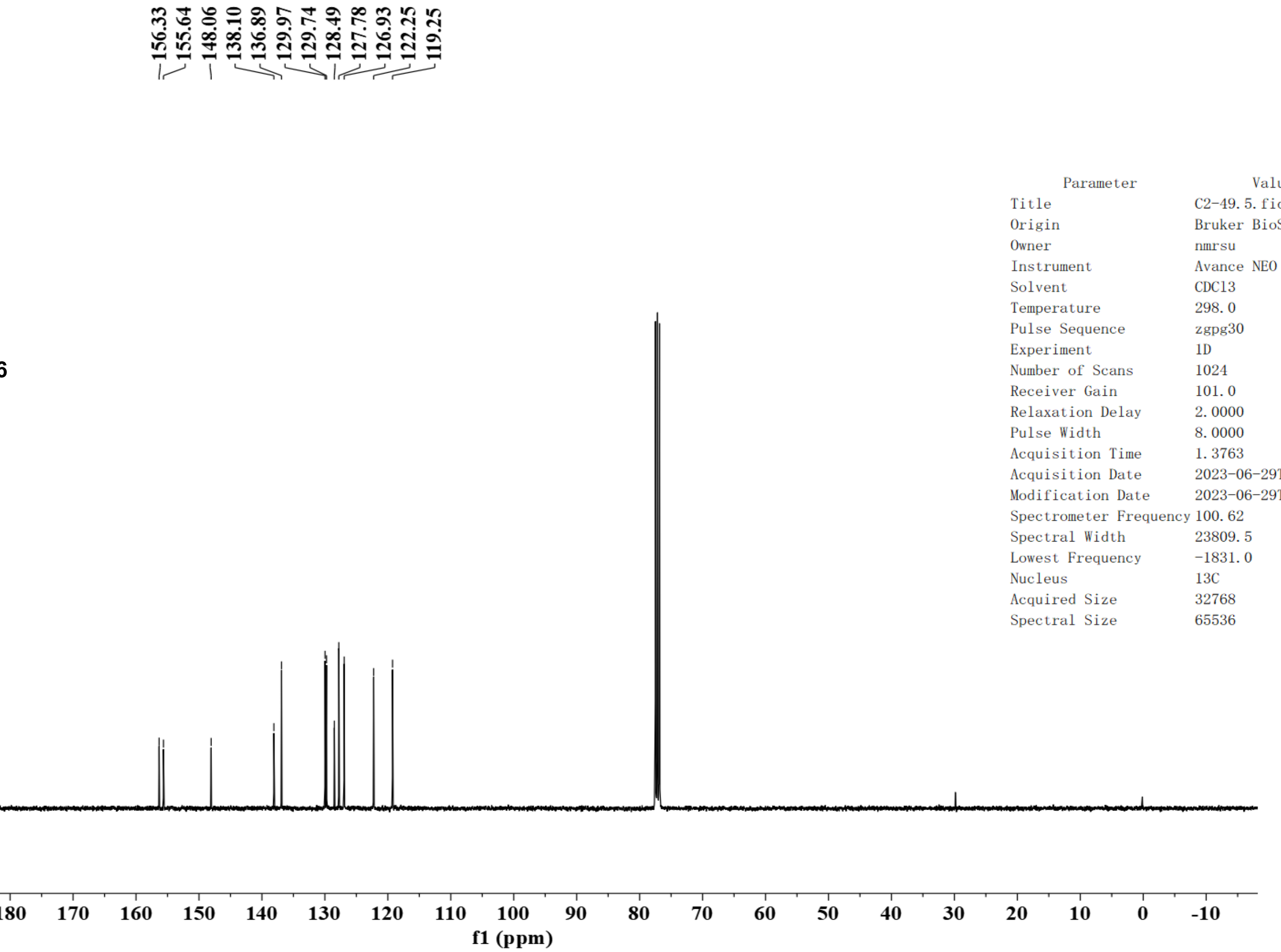

Parameter	Value
Title	C2-49.5.fid
Origin	Bruker BioSpin GmbH
Owner	nmrsu
Instrument	Avance NEO
Solvent	CDCl3
Temperature	298.0
Pulse Sequence	zgpg30
Experiment	1D
Number of Scans	1024
Receiver Gain	101.0
Relaxation Delay	2.0000
Pulse Width	8.0000
Acquisition Time	1.3763
Acquisition Date	2023-06-29T19:50:56
Modification Date	2023-06-29T19:50:51
Spectrometer Frequency	100.62
Spectral Width	23809.5
Lowest Frequency	-1831.0
Nucleus	13C
Acquired Size	32768
Spectral Size	65536

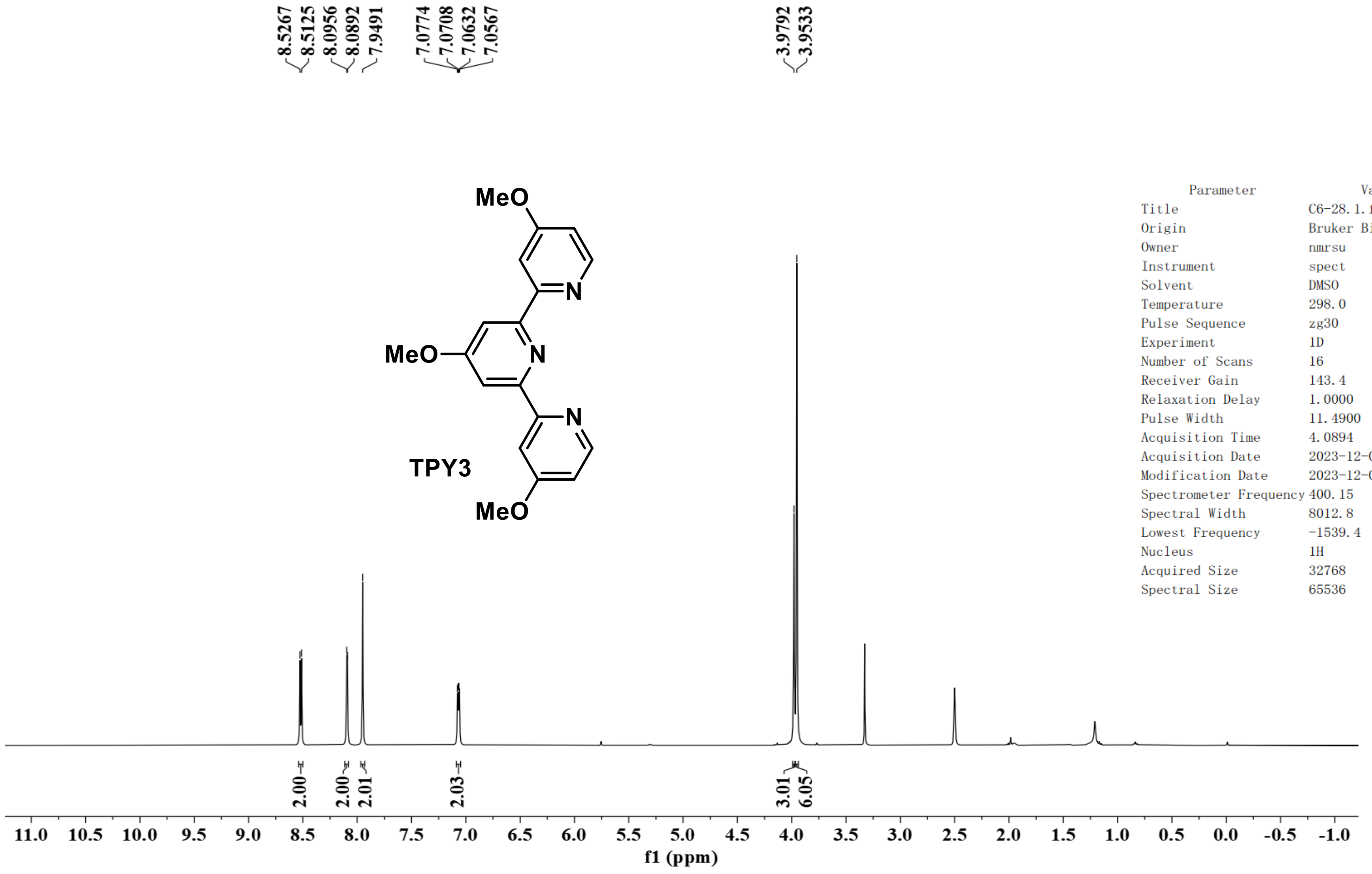

Parameter	Value
Title	C6-28.1.fid
Origin	Bruker BioSpin GmbH
Owner	nmrsu
Instrument	spect
Solvent	DMSO
Temperature	298.0
Pulse Sequence	zg30
Experiment	1D
Number of Scans	16
Receiver Gain	143.4
Relaxation Delay	1.0000
Pulse Width	11.4900
Acquisition Time	4.0894
Acquisition Date	2023-12-07T17:11:59
Modification Date	2023-12-08T21:15:57
Spectrometer Frequency	400.15
Spectral Width	8012.8
Lowest Frequency	-1539.4
Nucleus	1H
Acquired Size	32768
Spectral Size	65536

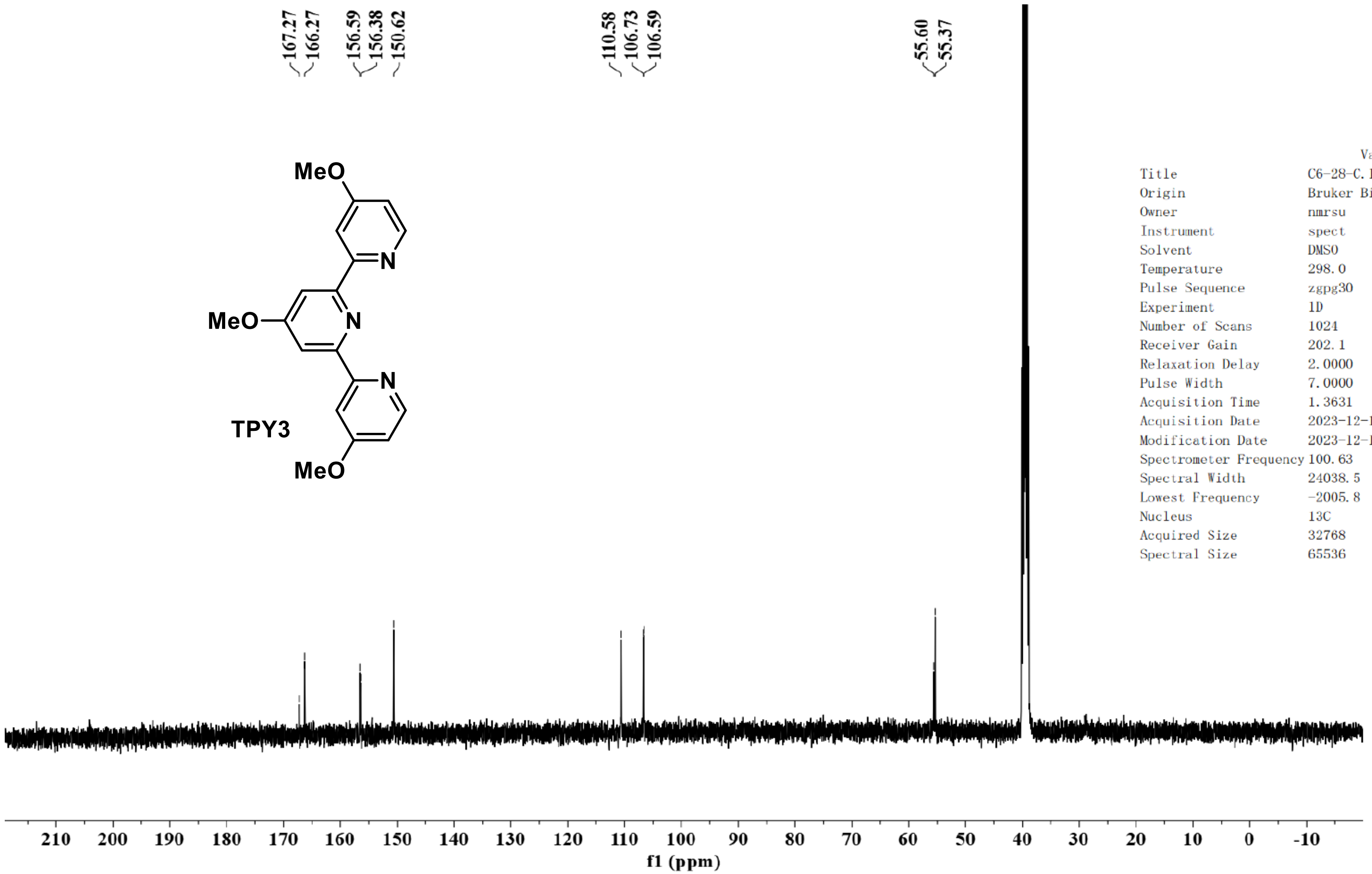

	Value
Title	C6-28-C.1.fid
Origin	Bruker BioSpin GmbH
Owner	nmrsu
Instrument	spect
Solvent	DMSO
Temperature	298.0
Pulse Sequence	zgpg30
Experiment	1D
Number of Scans	1024
Receiver Gain	202.1
Relaxation Delay	2.0000
Pulse Width	7.0000
Acquisition Time	1.3631
Acquisition Date	2023-12-11T23:39:24
Modification Date	2023-12-11T23:39:24
Spectrometer Frequency	100.63
Spectral Width	24038.5
Lowest Frequency	-2005.8
Nucleus	13C
Acquired Size	32768
Spectral Size	65536

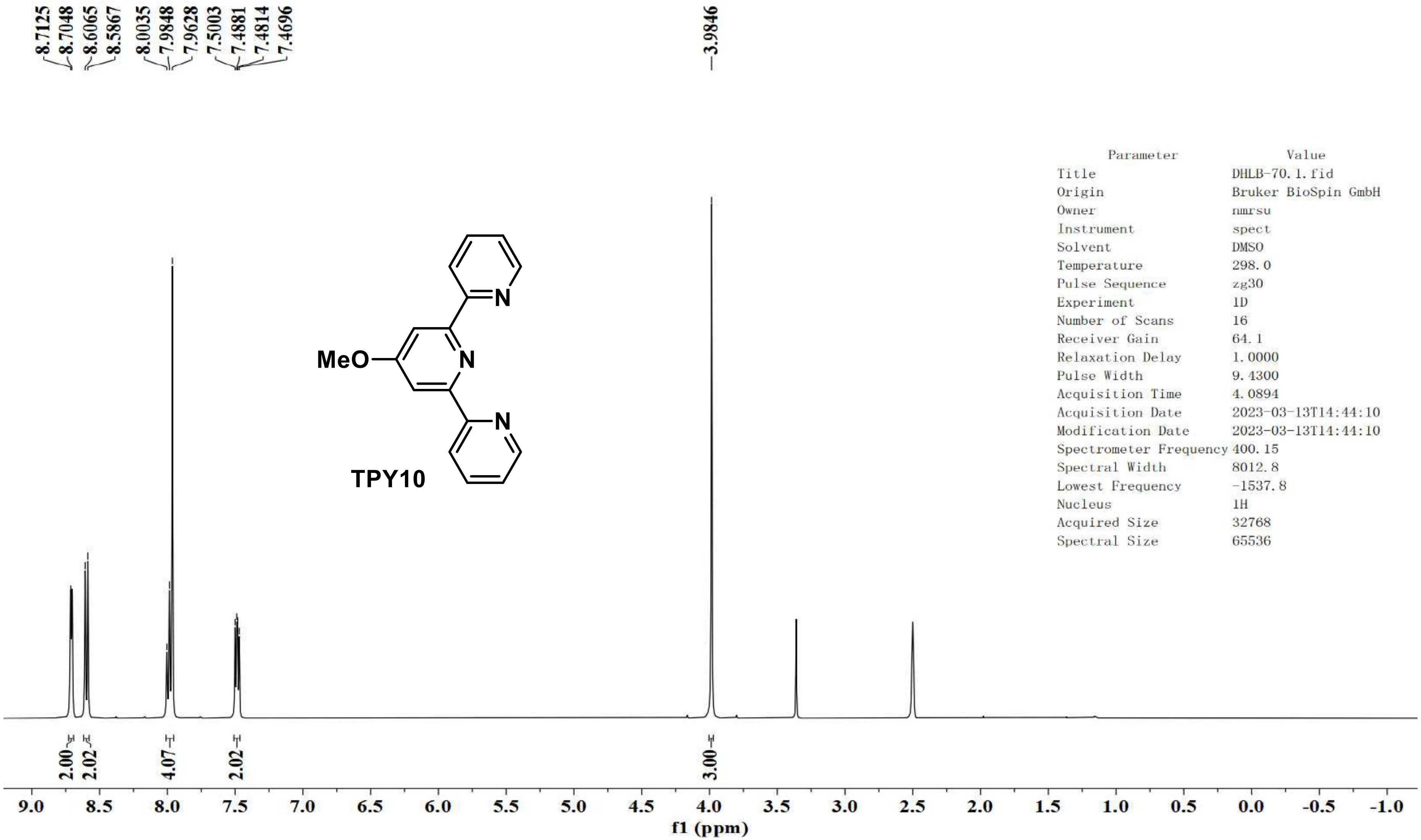

Parameter	Value
Title	DHLB-70.1.fid
Origin	Bruker BioSpin GmbH
Owner	nmrsu
Instrument	spect
Solvent	DMSO
Temperature	298.0
Pulse Sequence	zg30
Experiment	1D
Number of Scans	16
Receiver Gain	64.1
Relaxation Delay	1.0000
Pulse Width	9.4300
Acquisition Time	4.0894
Acquisition Date	2023-03-13T14:44:10
Modification Date	2023-03-13T14:44:10
Spectrometer Frequency	400.15
Spectral Width	8012.8
Lowest Frequency	-1537.8
Nucleus	1H
Acquired Size	32768
Spectral Size	65536

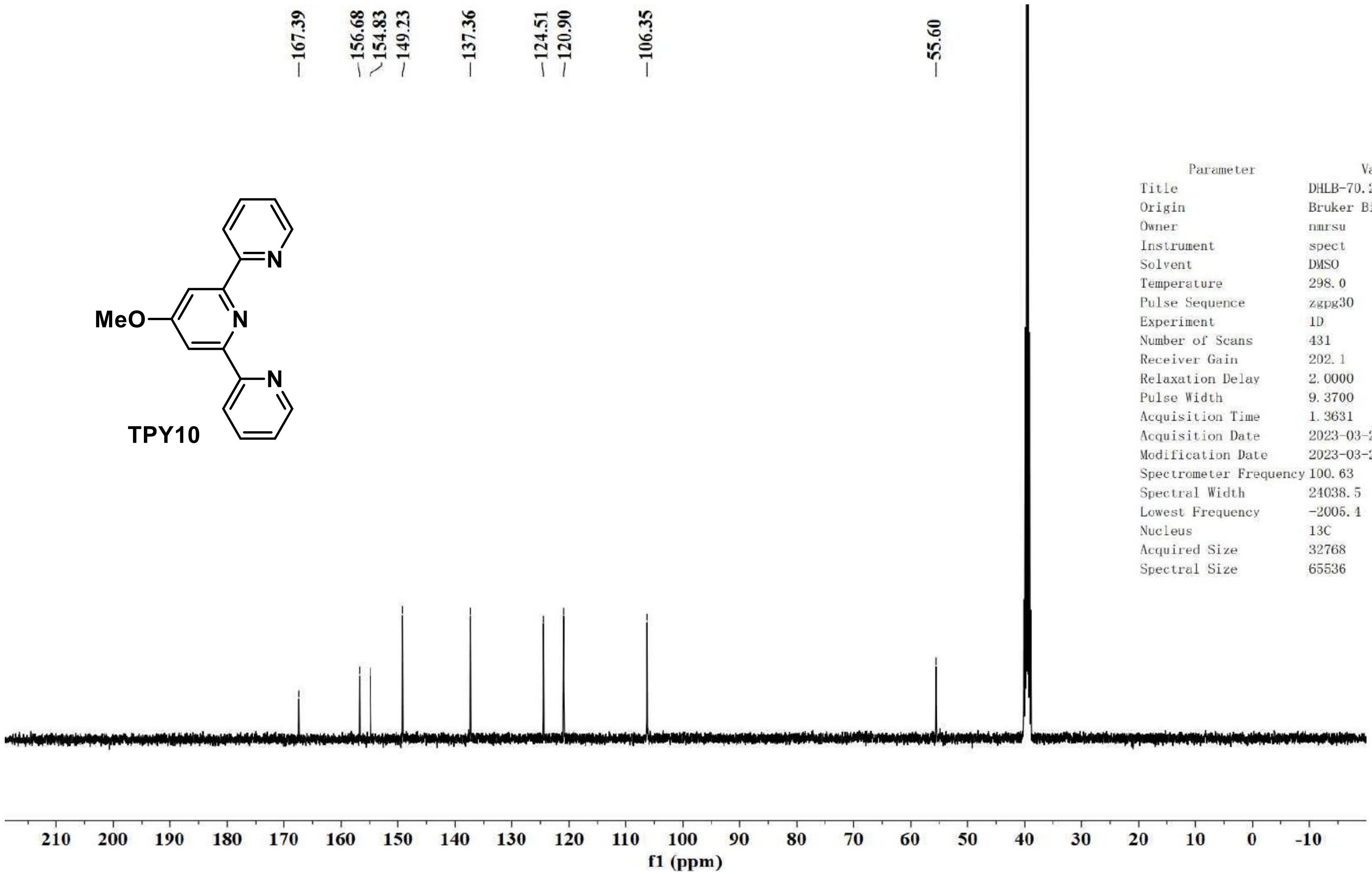

TPY10

Parameter	Value
Title	DHLB-70.2.fid
Origin	Bruker BioSpin GmbH
Owner	nmrsu
Instrument	spect
Solvent	DMSO
Temperature	298.0
Pulse Sequence	zgpg30
Experiment	1D
Number of Scans	431
Receiver Gain	202.1
Relaxation Delay	2.0000
Pulse Width	9.3700
Acquisition Time	1.3631
Acquisition Date	2023-03-23T16:56:37
Modification Date	2023-03-23T16:56:40
Spectrometer Frequency	100.63
Spectral Width	24038.5
Lowest Frequency	-2005.4
Nucleus	13C
Acquired Size	32768
Spectral Size	65536

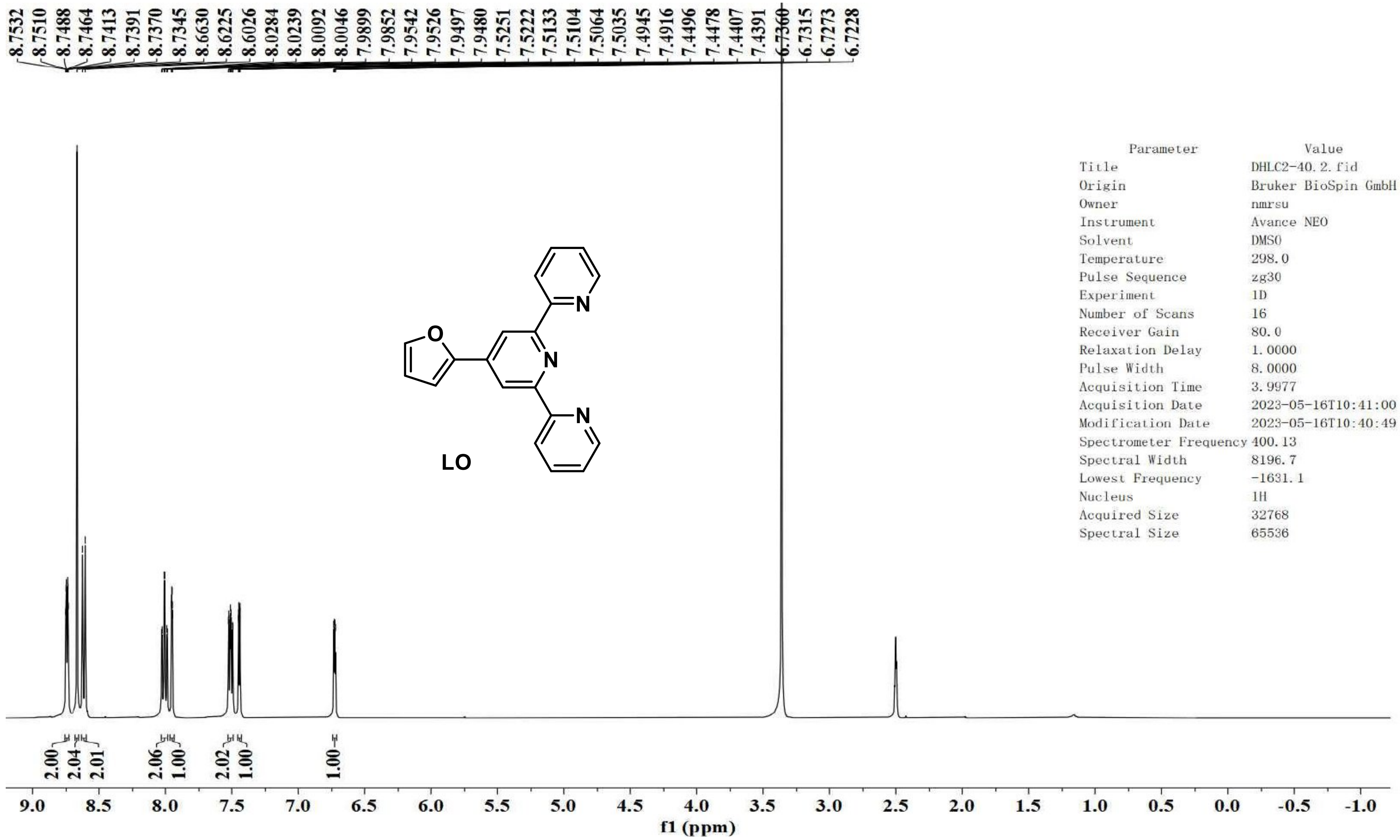

Parameter	Value
Title	DHLC2-40.2.fid
Origin	Bruker BioSpin GmbH
Owner	nmrsu
Instrument	Avance NEO
Solvent	DMSO
Temperature	298.0
Pulse Sequence	zg30
Experiment	1D
Number of Scans	16
Receiver Gain	80.0
Relaxation Delay	1.0000
Pulse Width	8.0000
Acquisition Time	3.9977
Acquisition Date	2023-05-16T10:41:00
Modification Date	2023-05-16T10:40:49
Spectrometer Frequency	400.13
Spectral Width	8196.7
Lowest Frequency	-1631.1
Nucleus	1H
Acquired Size	32768
Spectral Size	65536

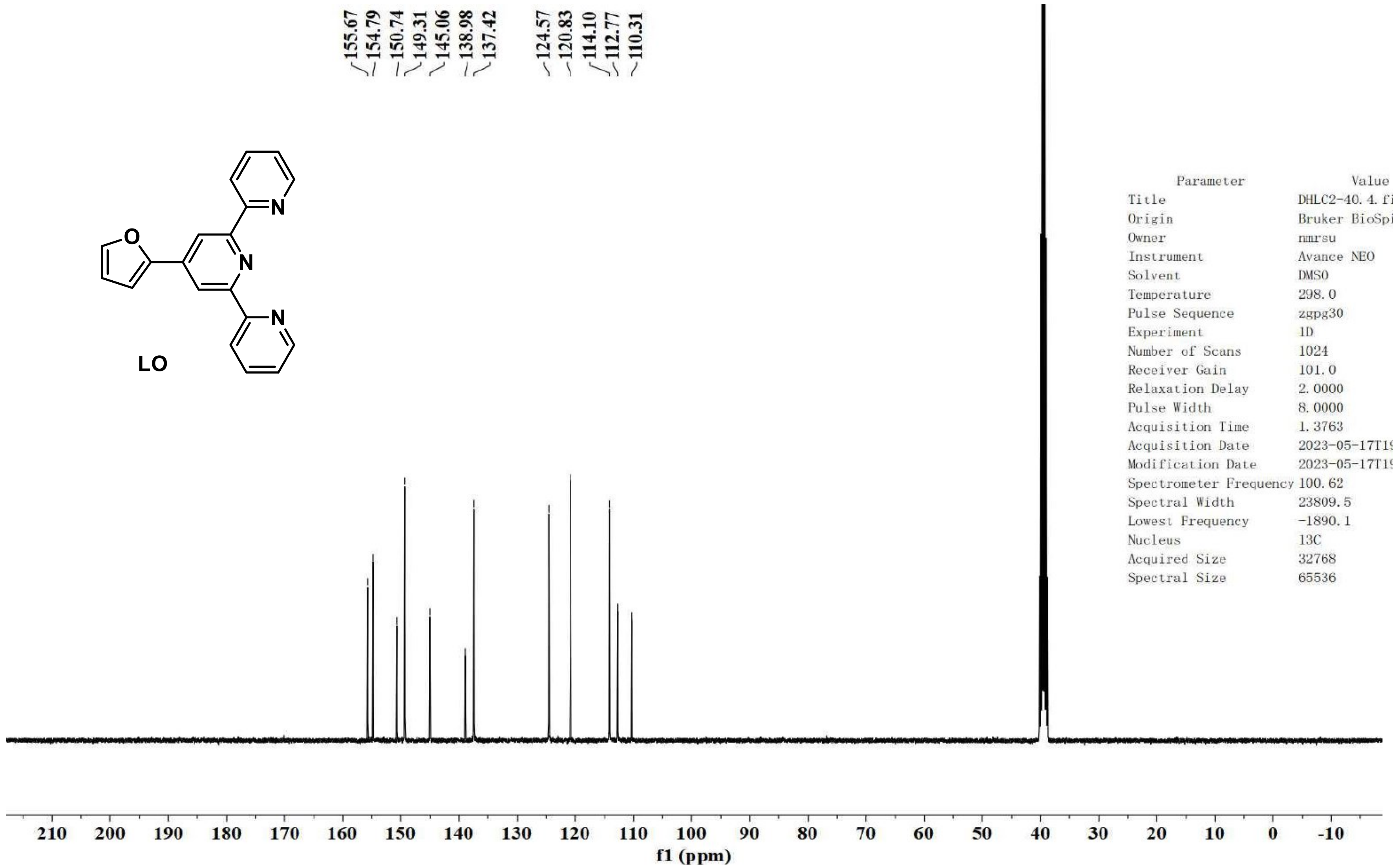

Parameter	Value
Title	DHLC2-40.4.fid
Origin	Bruker BioSpin GmbH
Owner	nmrsu
Instrument	Avance NEO
Solvent	DMSO
Temperature	298.0
Pulse Sequence	zgpg30
Experiment	1D
Number of Scans	1024
Receiver Gain	101.0
Relaxation Delay	2.0000
Pulse Width	8.0000
Acquisition Time	1.3763
Acquisition Date	2023-05-17T19:11:50
Modification Date	2023-05-17T19:11:42
Spectrometer Frequency	100.62
Spectral Width	23809.5
Lowest Frequency	-1890.1
Nucleus	13C
Acquired Size	32768
Spectral Size	65536

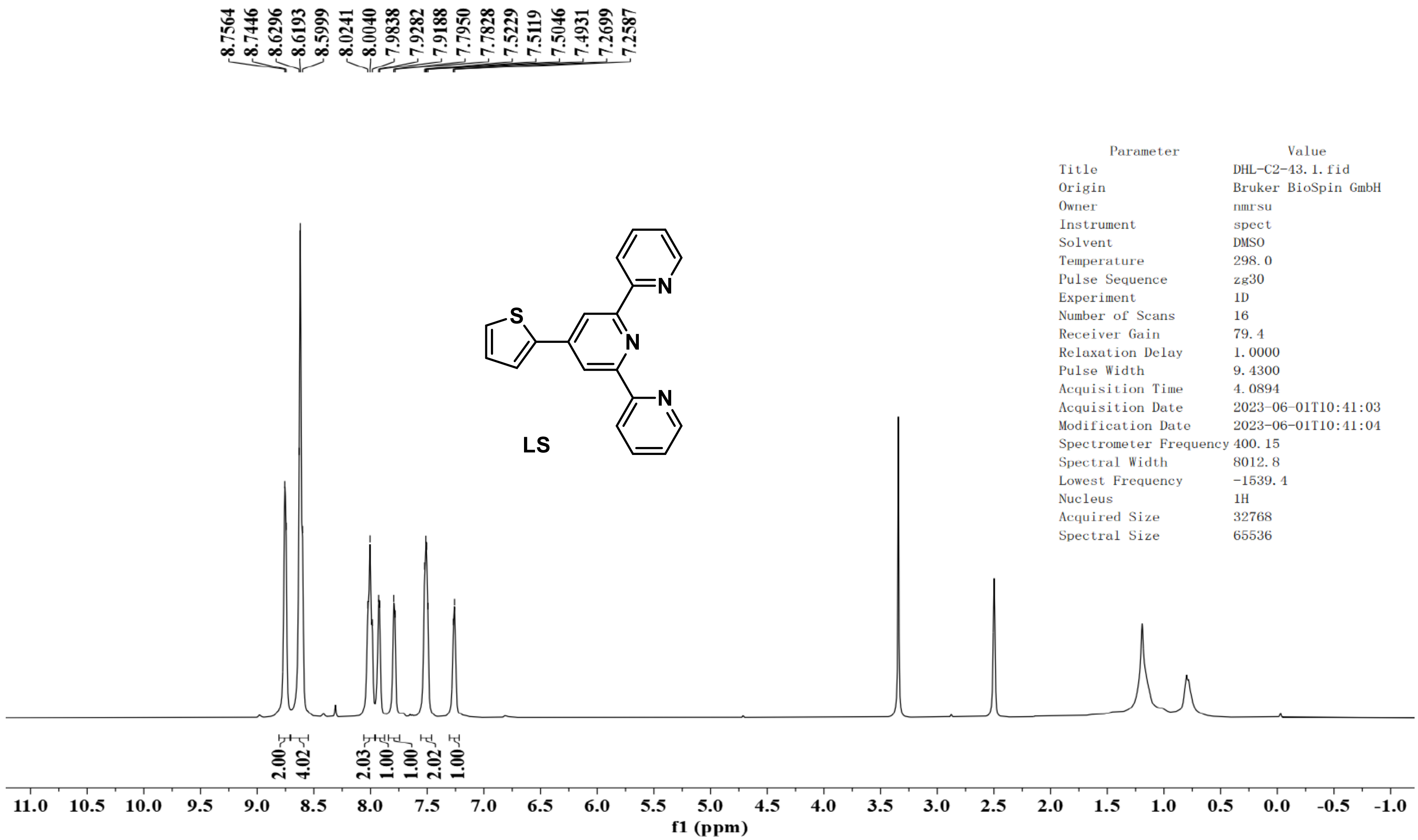
8.7564
8.7446
8.6296
8.6193
8.5999
8.0241
8.0040
7.9838
7.9282
7.9188
7.7950
7.7828
7.5229
7.5119
7.5046
7.4931
7.2699
7.2587
Parameter Value
Title DHL-C2-43.1.fid
Origin Bruker BioSpin GmbH
Owner nmrsu
Instrument spect
Solvent DMSO
Temperature 298.0
Pulse Sequence zg30
Experiment 1D
Number of Scans 16
Receiver Gain 79.4
Relaxation Delay 1.0000
Pulse Width 9.4300
Acquisition Time 4.0894
Acquisition Date 2023-06-01T10:41:03
Modification Date 2023-06-01T10:41:04
Spectrometer Frequency 400.15
Spectral Width 8012.8
Lowest Frequency -1539.4
Nucleus 1H
Acquired Size 32768
Spectral Size 65536
S
N
N
N
LS
2.00
4.02
2.03
1.00
1.00
2.02
1.00
11.0 10.5 10.0 9.5 9.0 8.5 8.0 7.5 7.0 6.5 6.0 5.5 5.0 4.5 4.0 3.5 3.0 2.5 2.0 1.5 1.0 0.5 0.0 -0.5 -1.0
f1 (ppm)

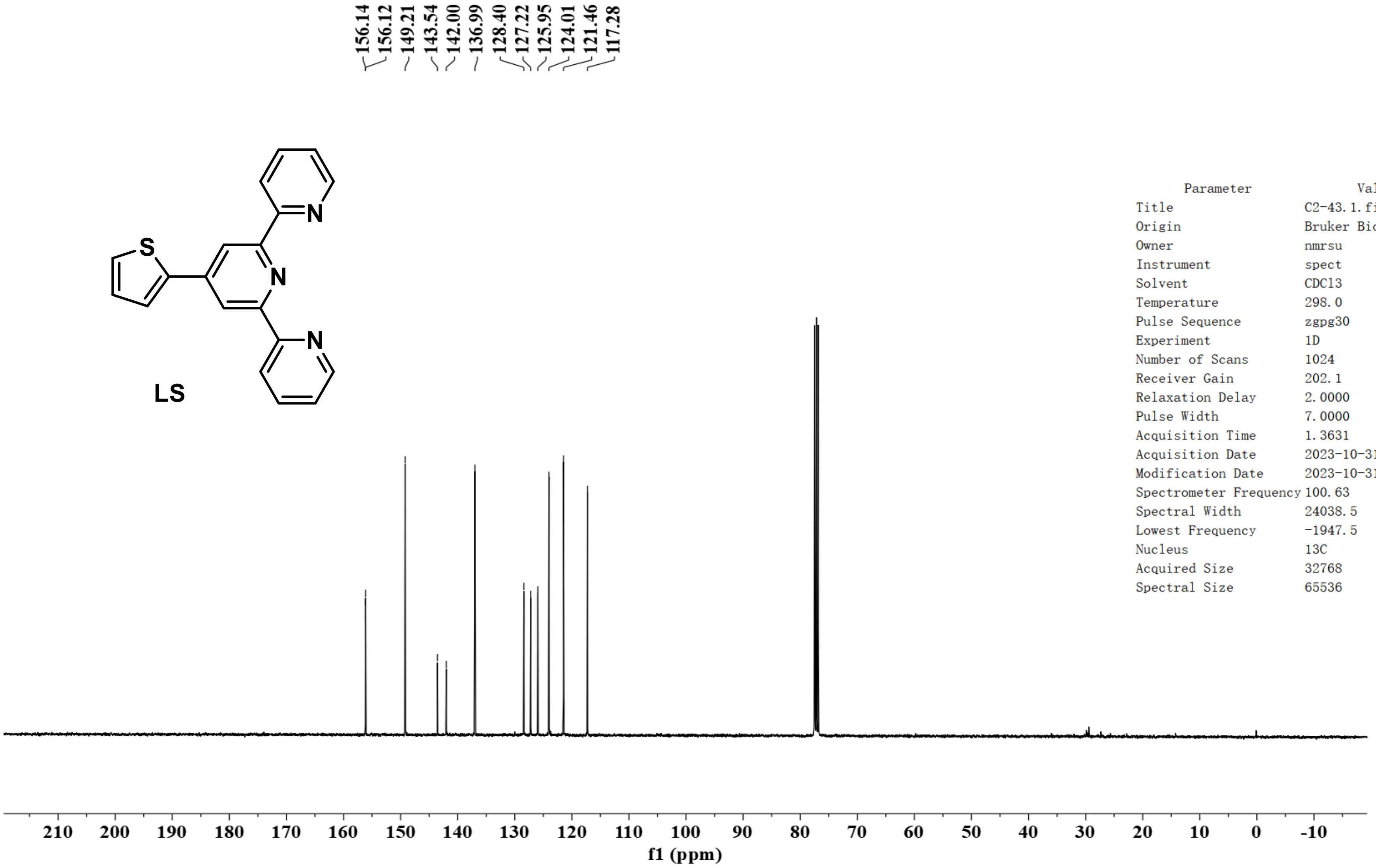

Parameter	Value
Title	C2-43.1.fid
Origin	Bruker BioSpin GmbH
Owner	nmrsu
Instrument	spect
Solvent	CDCl3
Temperature	298.0
Pulse Sequence	zgpg30
Experiment	1D
Number of Scans	1024
Receiver Gain	202.1
Relaxation Delay	2.0000
Pulse Width	7.0000
Acquisition Time	1.3631
Acquisition Date	2023-10-31T00:07:12
Modification Date	2023-10-31T00:07:12
Spectrometer Frequency	100.63
Spectral Width	24038.5
Lowest Frequency	-1947.5
Nucleus	13C
Acquired Size	32768
Spectral Size	65536

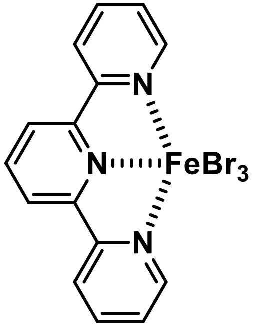

Fe(TPY1)Br_3

Parameter	Value
Title	H-5.1.fid
Origin	Bruker BioSpin GmbH
Owner	nmrsu
Instrument	spect
Solvent	DMSO
Temperature	298.0
Pulse Sequence	zg30
Experiment	1D
Number of Scans	16
Receiver Gain	98.2
Relaxation Delay	1.0000
Pulse Width	9.7800
Acquisition Time	4.0894
Acquisition Date	2022-04-20T20:43:42
Modification Date	2022-04-20T20:43:44
Spectrometer Frequency	400.15
Spectral Width	8012.8
Lowest Frequency	-3441.1
Nucleus	1H
Acquired Size	32768
Spectral Size	65536

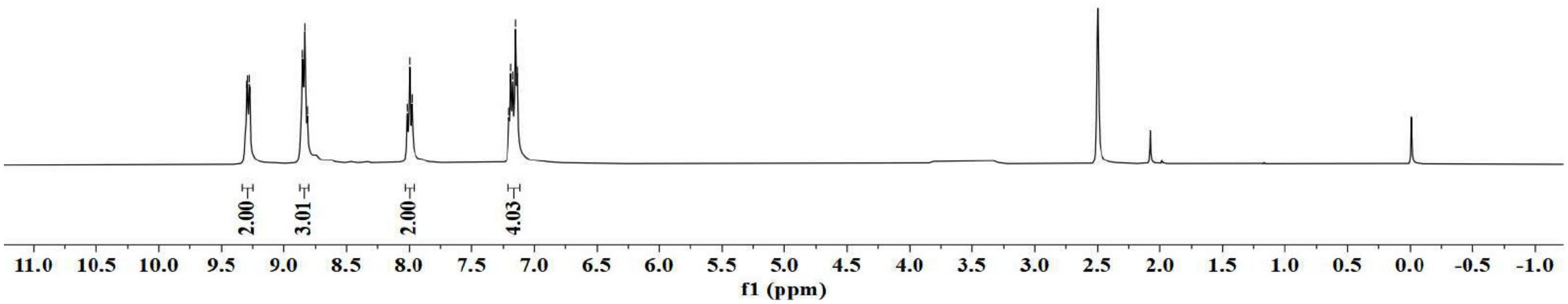

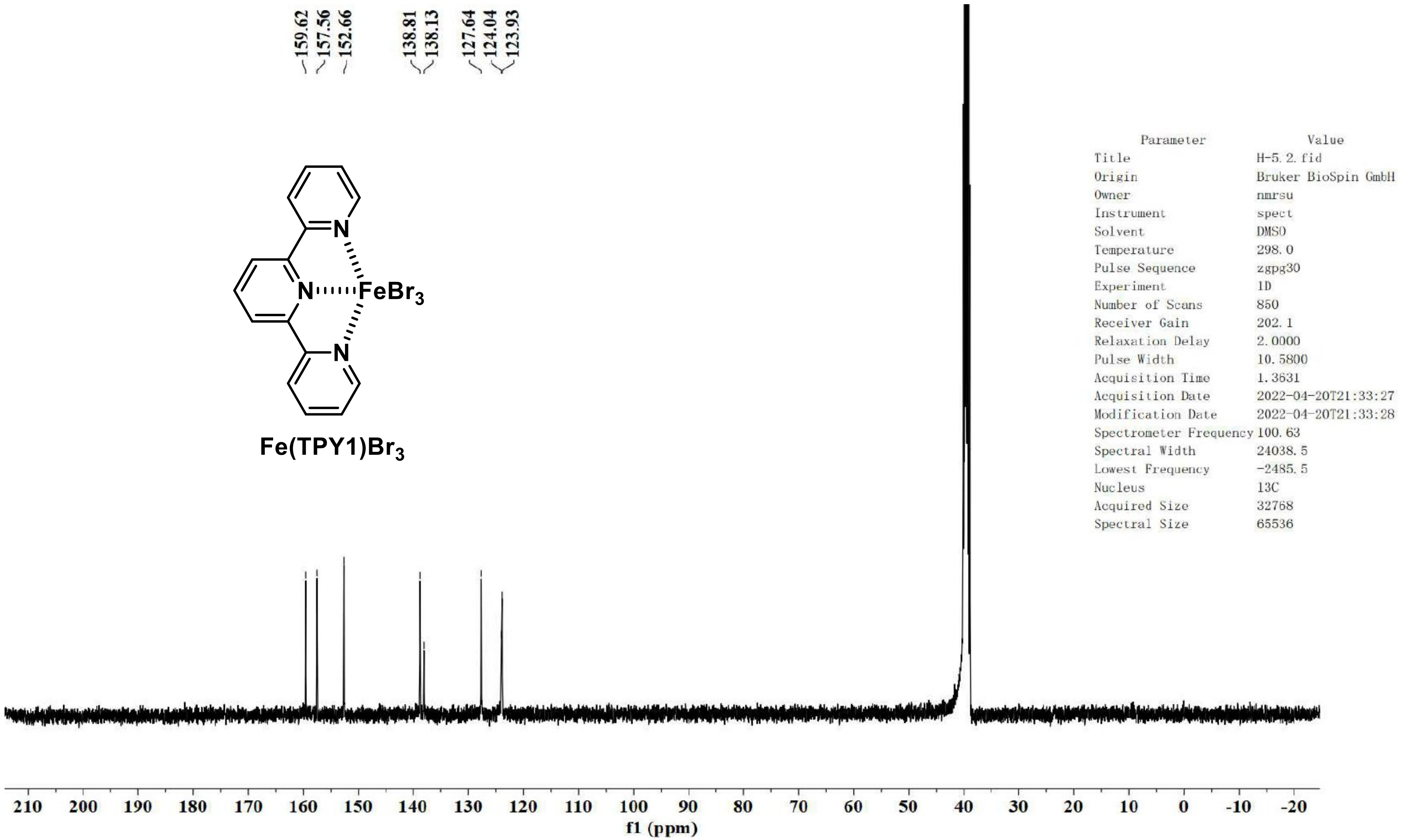
159.62
157.56
152.66
138.81
138.13
127.64
124.04
123.93
N
FeBr3
Fe(TPY1)Br3
Parameter Value
Title H-5.2.fid
Origin Bruker BioSpin GmbH
Owner nmrsu
Instrument spect
Solvent DMSO
Temperature 298.0
Pulse Sequence zgpg30
Experiment 1D
Number of Scans 850
Receiver Gain 202.1
Relaxation Delay 2.0000
Pulse Width 10.5800
Acquisition Time 1.3631
Acquisition Date 2022-04-20T21:33:27
Modification Date 2022-04-20T21:33:28
Spectrometer Frequency 100.63
Spectral Width 24038.5
Lowest Frequency -2485.5
Nucleus 13C
Acquired Size 32768
Spectral Size 65536
210 200 190 180 170 160 150 140 130 120 110 100 90 80 70 60 50 40 30 20 10 0 -10 -20
f1 (ppm)

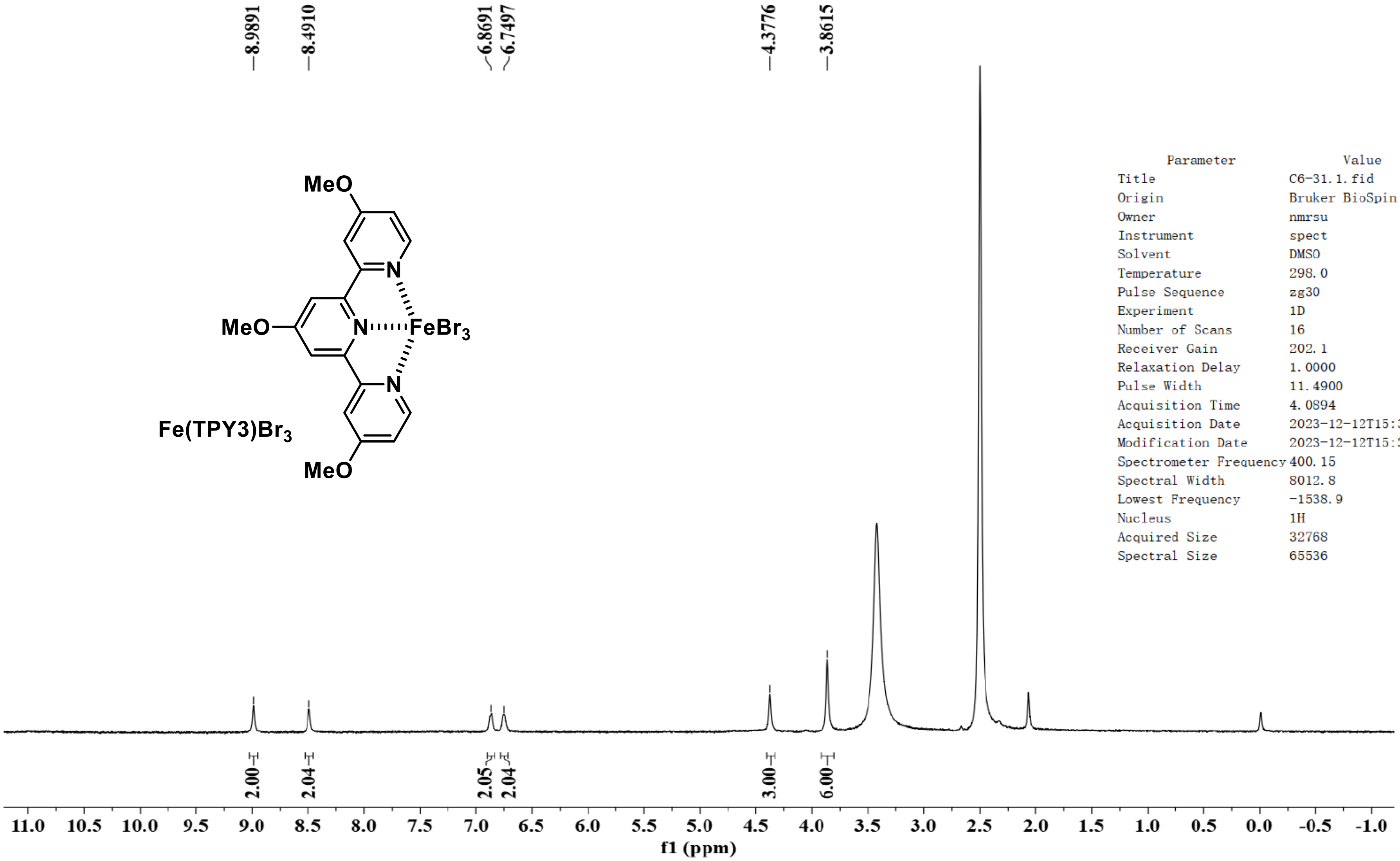

Parameter	Value
Title	C6-31.1.fid
Origin	Bruker BioSpin GmbH
Owner	nmrsu
Instrument	spect
Solvent	DMSO
Temperature	298.0
Pulse Sequence	zg30
Experiment	1D
Number of Scans	16
Receiver Gain	202.1
Relaxation Delay	1.0000
Pulse Width	11.4900
Acquisition Time	4.0894
Acquisition Date	2023-12-12T15:32:58
Modification Date	2023-12-12T15:32:58
Spectrometer Frequency	400.15
Spectral Width	8012.8
Lowest Frequency	-1538.9
Nucleus	1H
Acquired Size	32768
Spectral Size	65536

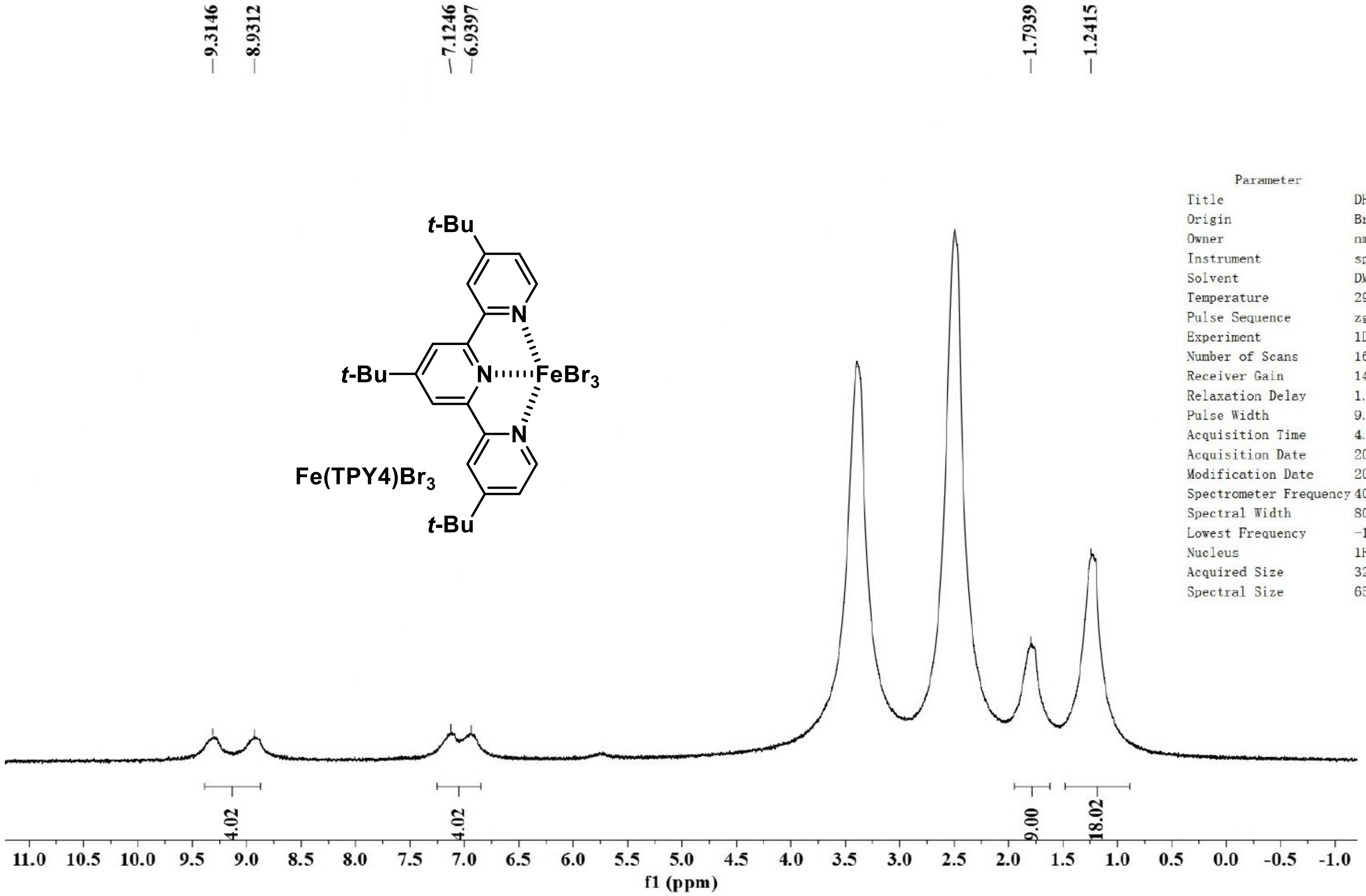

Parameter	Value
Title	DHLB-43.1.fid
Origin	Bruker BioSpin GmbH
Owner	nmrsu
Instrument	spect
Solvent	DMSO
Temperature	298.1
Pulse Sequence	zg30
Experiment	1D
Number of Scans	16
Receiver Gain	143.4
Relaxation Delay	1.0000
Pulse Width	9.4300
Acquisition Time	4.0894
Acquisition Date	2023-03-07T16:59:07
Modification Date	2023-03-07T16:59:08
Spectrometer Frequency	400.15
Spectral Width	8012.8
Lowest Frequency	-1540.0
Nucleus	1H
Acquired Size	32768
Spectral Size	65536

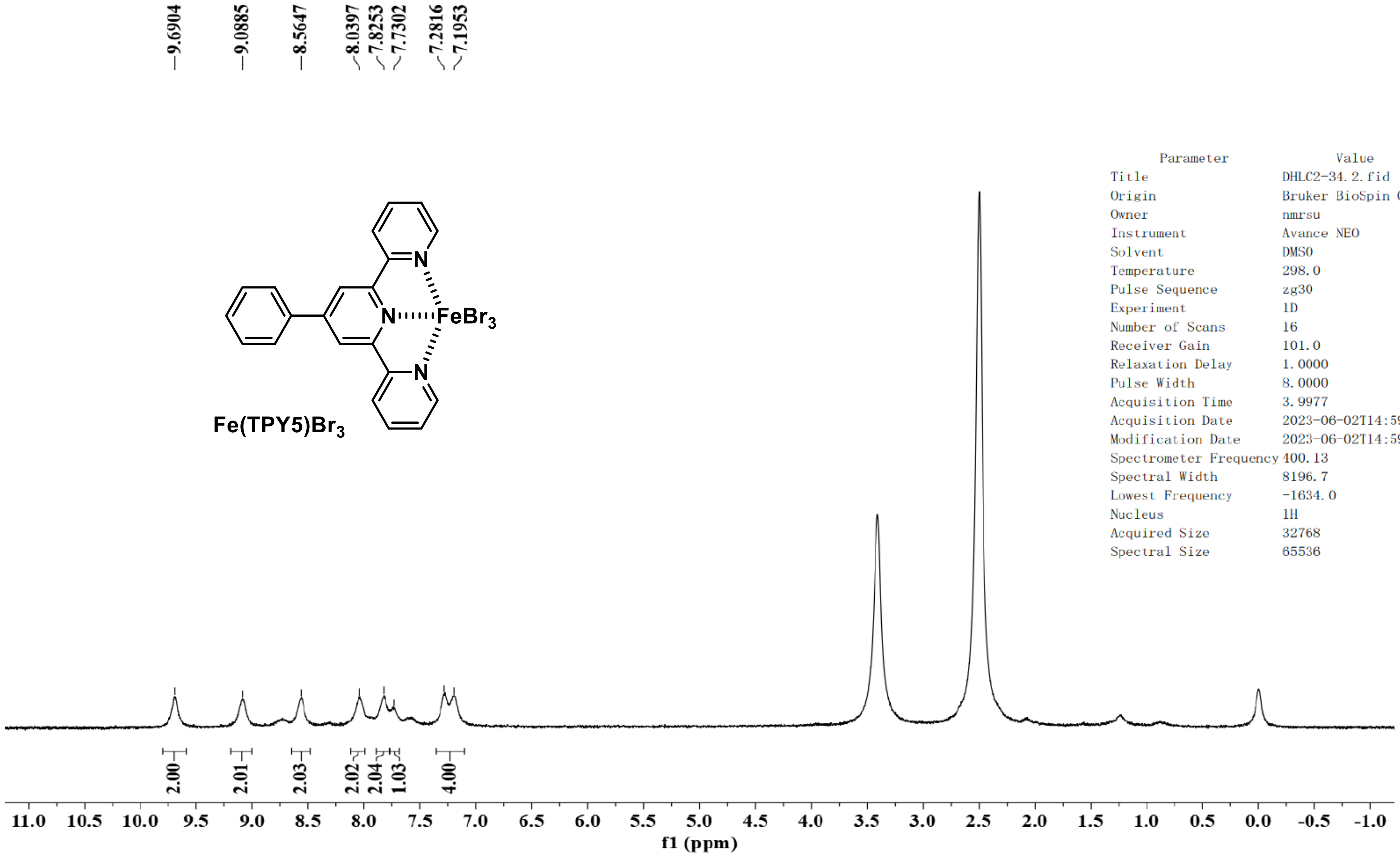

Parameter	Value
Title	DHLC2-34.2.fid
Origin	Bruker BioSpin GmbH
Owner	nmrsu
Instrument	Avance NEO
Solvent	DMSO
Temperature	298.0
Pulse Sequence	zg30
Experiment	1D
Number of Scans	16
Receiver Gain	101.0
Relaxation Delay	1.0000
Pulse Width	8.0000
Acquisition Time	3.9977
Acquisition Date	2023-06-02T14:59:43
Modification Date	2023-06-02T14:59:54
Spectrometer Frequency	400.13
Spectral Width	8196.7
Lowest Frequency	-1634.0
Nucleus	1H
Acquired Size	32768
Spectral Size	65536

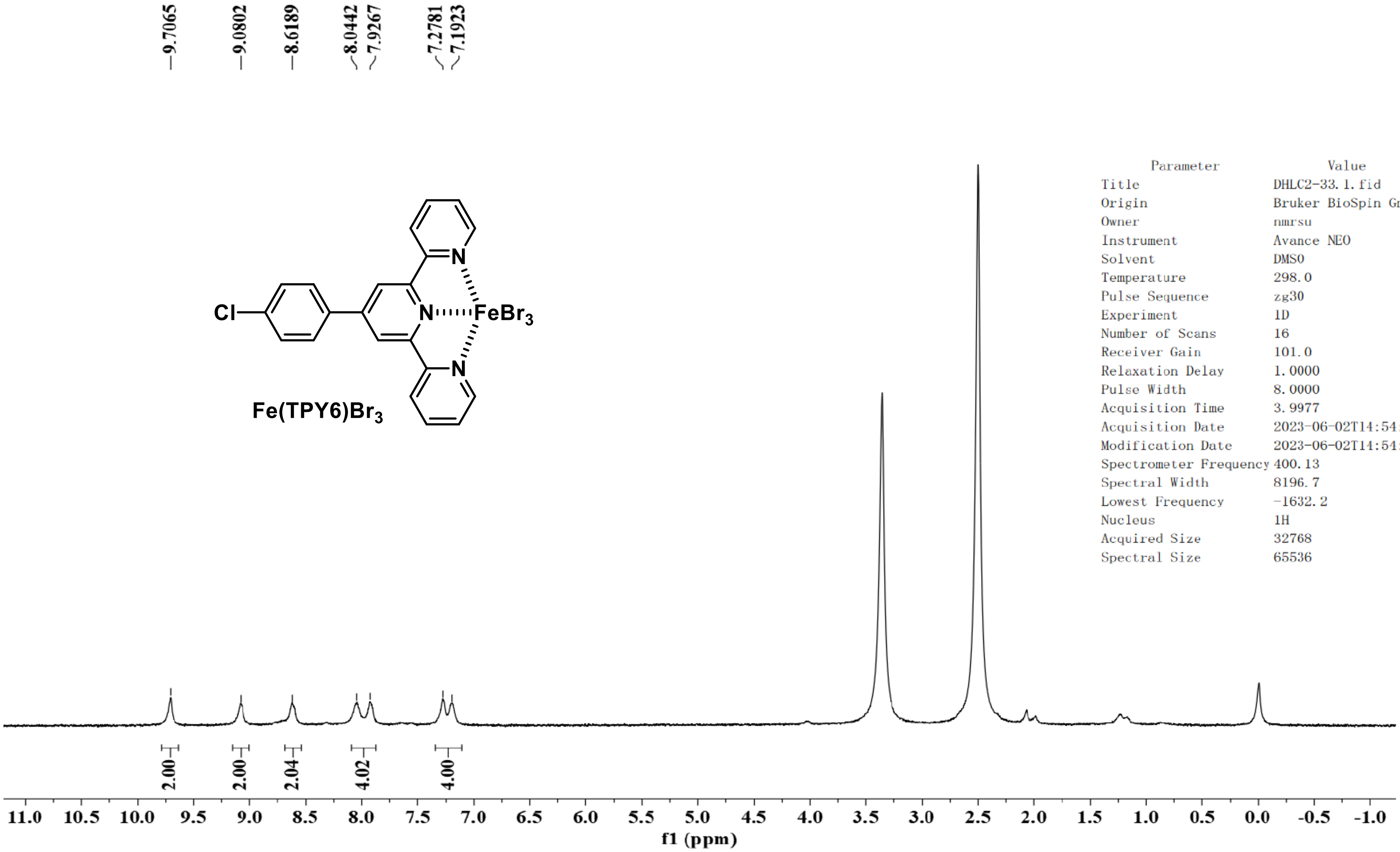

Parameter	Value
Title	DHLC2-33.1.fid
Origin	Bruker BioSpin GmbH
Owner	nmrsu
Instrument	Avance NEO
Solvent	DMSO
Temperature	298.0
Pulse Sequence	zg30
Experiment	1D
Number of Scans	16
Receiver Gain	101.0
Relaxation Delay	1.0000
Pulse Width	8.0000
Acquisition Time	3.9977
Acquisition Date	2023-06-02T14:54:04
Modification Date	2023-06-02T14:54:14
Spectrometer Frequency	400.13
Spectral Width	8196.7
Lowest Frequency	-1632.2
Nucleus	1H
Acquired Size	32768
Spectral Size	65536

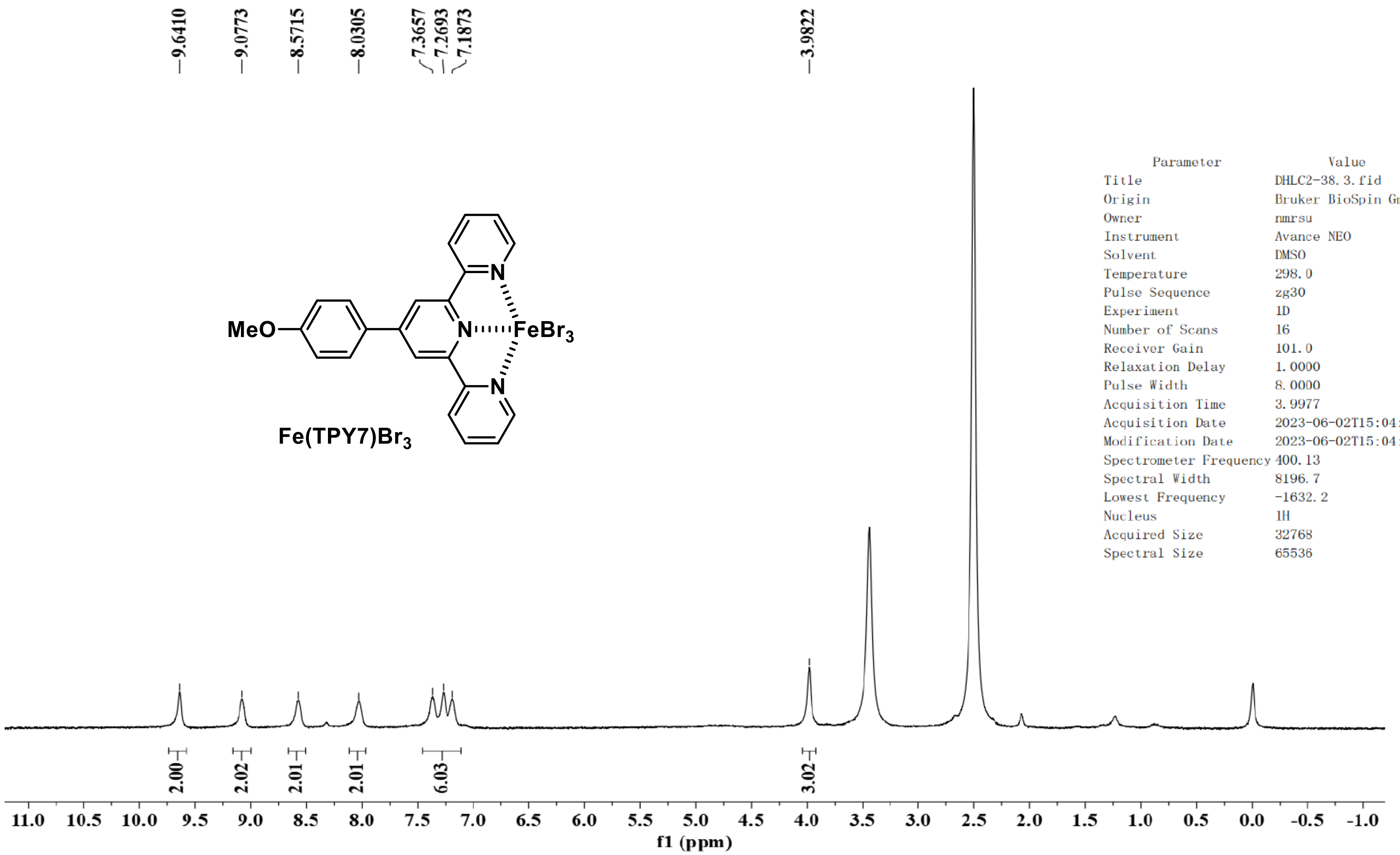

9.6410
9.0773
8.5715
8.0305
7.3657
7.2693
7.1873
3.9822
MeO
N
N
N
FeBr3
Fe(TPY7)Br3
Parameter Value
Title DHLC2-38.3.fid
Origin Bruker BioSpin GmbH
Owner nmrsu
Instrument Avance NEO
Solvent DMSO
Temperature 298.0
Pulse Sequence zg30
Experiment 1D
Number of Scans 16
Receiver Gain 101.0
Relaxation Delay 1.0000
Pulse Width 8.0000
Acquisition Time 3.9977
Acquisition Date 2023-06-02T15:04:19
Modification Date 2023-06-02T15:04:29
Spectrometer Frequency 400.13
Spectral Width 8196.7
Lowest Frequency -1632.2
Nucleus 1H
Acquired Size 32768
Spectral Size 65536
2.00
2.02
2.01
2.01
6.03
3.02
11.0 10.5 10.0 9.5 9.0 8.5 8.0 7.5 7.0 6.5 6.0 5.5 5.0 4.5 4.0 3.5 3.0 2.5 2.0 1.5 1.0 0.5 0.0 -0.5 -1.0
f1 (ppm)

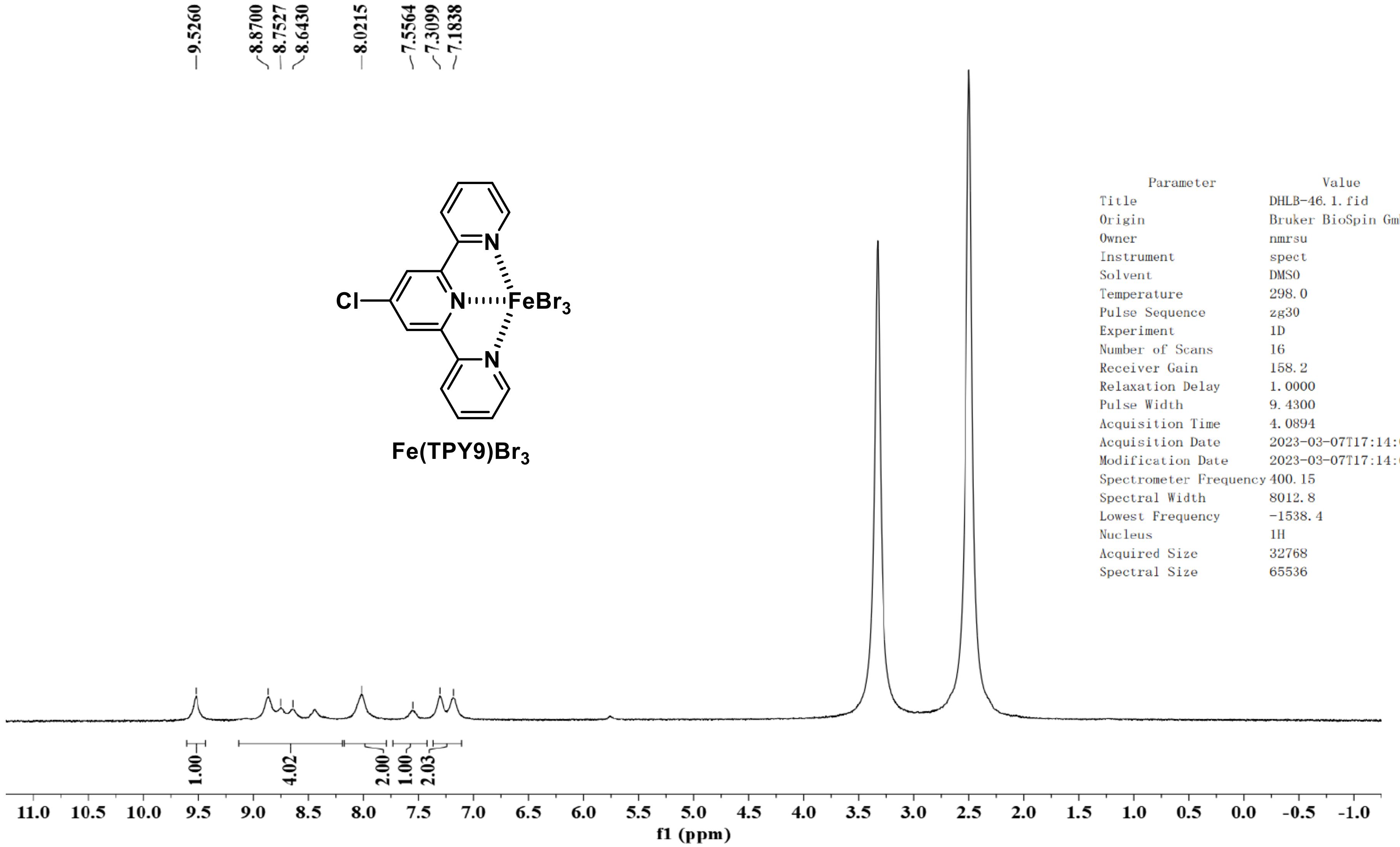

Fe(TPY9)Br_3

Parameter	Value
Title	DHLB-46.1.fid
Origin	Bruker BioSpin GmbH
Owner	nmrsu
Instrument	spect
Solvent	DMSO
Temperature	298.0
Pulse Sequence	zg30
Experiment	1D
Number of Scans	16
Receiver Gain	158.2
Relaxation Delay	1.0000
Pulse Width	9.4300
Acquisition Time	4.0894
Acquisition Date	2023-03-07T17:14:04
Modification Date	2023-03-07T17:14:06
Spectrometer Frequency	400.15
Spectral Width	8012.8
Lowest Frequency	-1538.4
Nucleus	1H
Acquired Size	32768
Spectral Size	65536

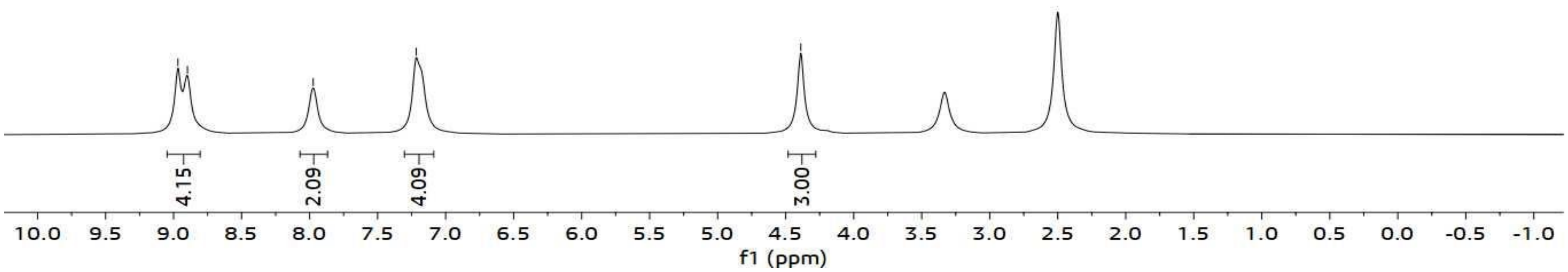

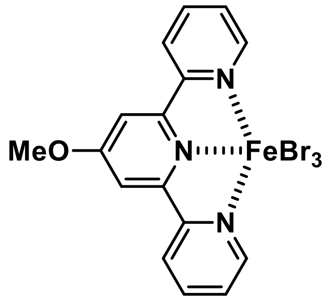

Fe(TPY10)Br_3

Title	PHL-C71.1.fid
Origin	Bruker BioSpin GmbH
Owner	nmrsu
Spectrometer	spect
Solvent	DMSO
Temperature	298.0
Pulse Sequence	zg30
Experiment	1D
Number of Scans	16
Receiver Gain	31
Relaxation Delay	1.0000
Pulse Width	9.7800
Acquisition Time	4.0894
Acquisition Date	2023-01-06T09:51:49
Modification Date	2023-01-06T09:51:50
Spectrometer Frequency	400.15
Spectral Width	8012.8
Lowest Frequency	-1534.2
Nucleus	1H
Acquired Size	32768
Spectral Size	65536

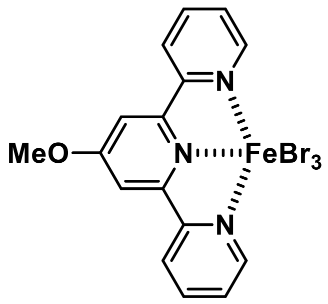

Fe(TPY10)Br_3

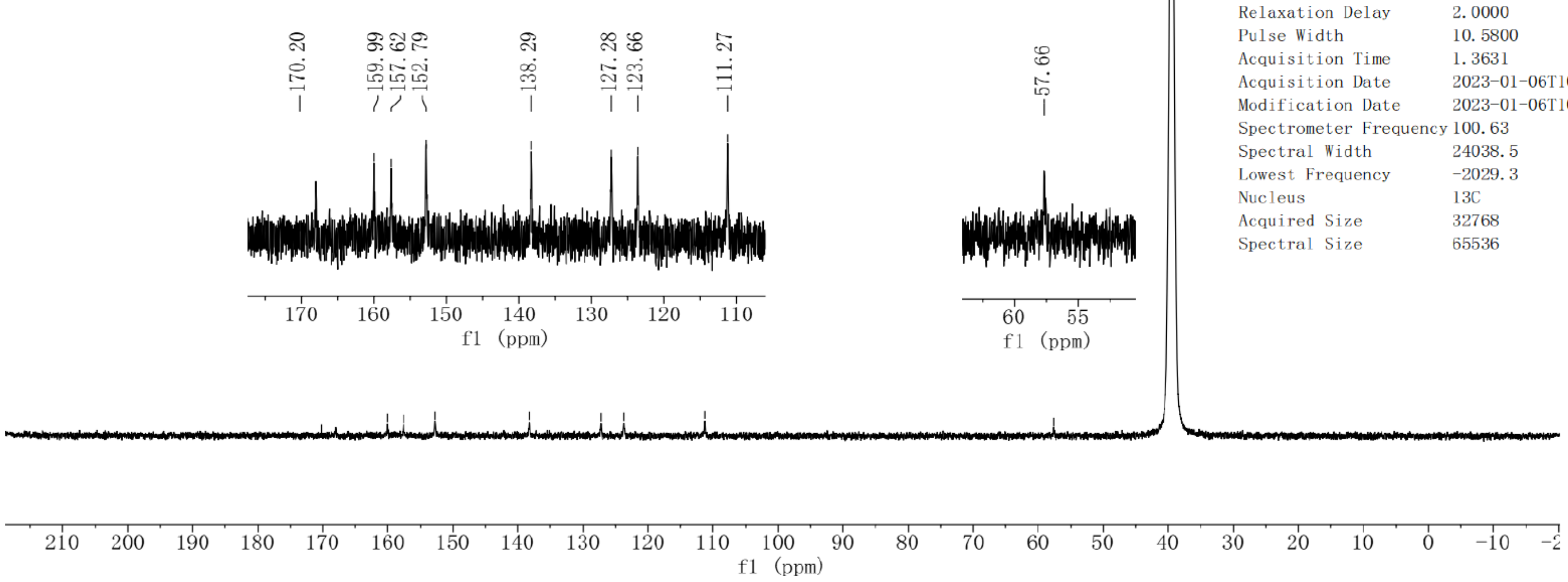

Title	PHL-C71.2.fid
Origin	Bruker BioSpin GmbH
Owner	nmrsu
Spectrometer	spect
Solvent	DMSO
Temperature	298.0
Pulse Sequence	zgpg30
Experiment	1D
Number of Scans	1024
Receiver Gain	202
Relaxation Delay	2.0000
Pulse Width	10.5800
Acquisition Time	1.3631
Acquisition Date	2023-01-06T10:50:38
Modification Date	2023-01-06T10:50:40
Spectrometer Frequency	100.63
Spectral Width	24038.5
Lowest Frequency	-2029.3
Nucleus	13C
Acquired Size	32768
Spectral Size	65536

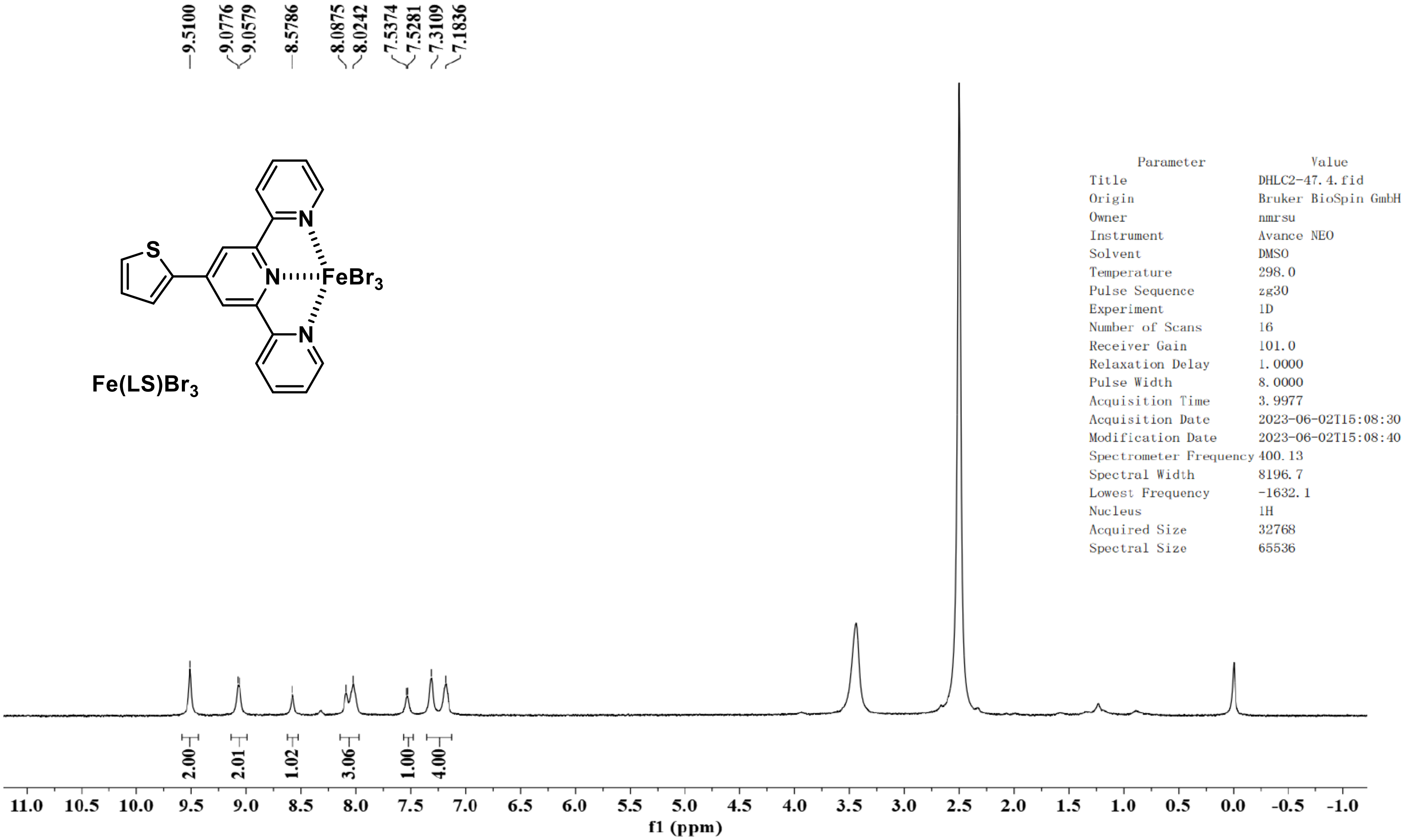

9.5100
9.0776
9.0579
8.5786
8.0875
8.0242
7.5374
7.5281
7.3109
7.1836
S
N
N
N
FeBr3
Fe(LS)Br3
Parameter Value
Title DHLC2-47.4.fid
Origin Bruker BioSpin GmbH
Owner nmrsu
Instrument Avance NEO
Solvent DMSO
Temperature 298.0
Pulse Sequence zg30
Experiment 1D
Number of Scans 16
Receiver Gain 101.0
Relaxation Delay 1.0000
Pulse Width 8.0000
Acquisition Time 3.9977
Acquisition Date 2023-06-02T15:08:30
Modification Date 2023-06-02T15:08:40
Spectrometer Frequency 400.13
Spectral Width 8196.7
Lowest Frequency -1632.1
Nucleus 1H
Acquired Size 32768
Spectral Size 65536
2.00
2.01
1.02
3.06
1.00
4.00
11.0 10.5 10.0 9.5 9.0 8.5 8.0 7.5 7.0 6.5 6.0 5.5 5.0 4.5 4.0 3.5 3.0 2.5 2.0 1.5 1.0 0.5 0.0 -0.5 -1.0
f1 (ppm)

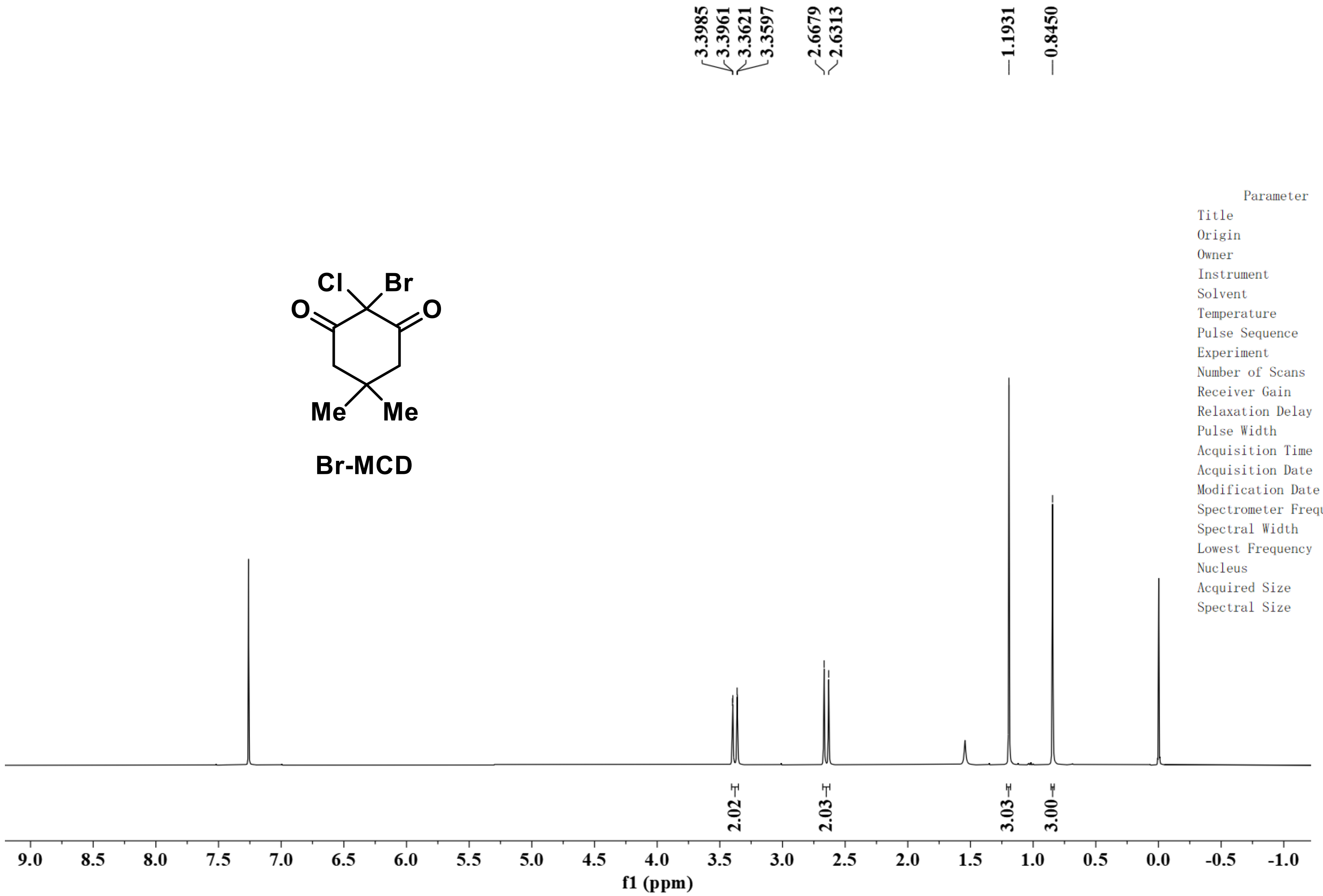

Br-MCD

Parameter	Value
Title	C6-20.1.fid
Origin	Bruker BioSpin GmbH
Owner	nmrsu
Instrument	spect
Solvent	CDCl3
Temperature	298.0
Pulse Sequence	zg30
Experiment	1D
Number of Scans	16
Receiver Gain	202.1
Relaxation Delay	1.0000
Pulse Width	11.4900
Acquisition Time	4.0894
Acquisition Date	2023-11-21T15:32:13
Modification Date	2023-11-21T15:32:12
Spectrometer Frequency	400.15
Spectral Width	8012.8
Lowest Frequency	-1545.7
Nucleus	1H
Acquired Size	32768
Spectral Size	65536

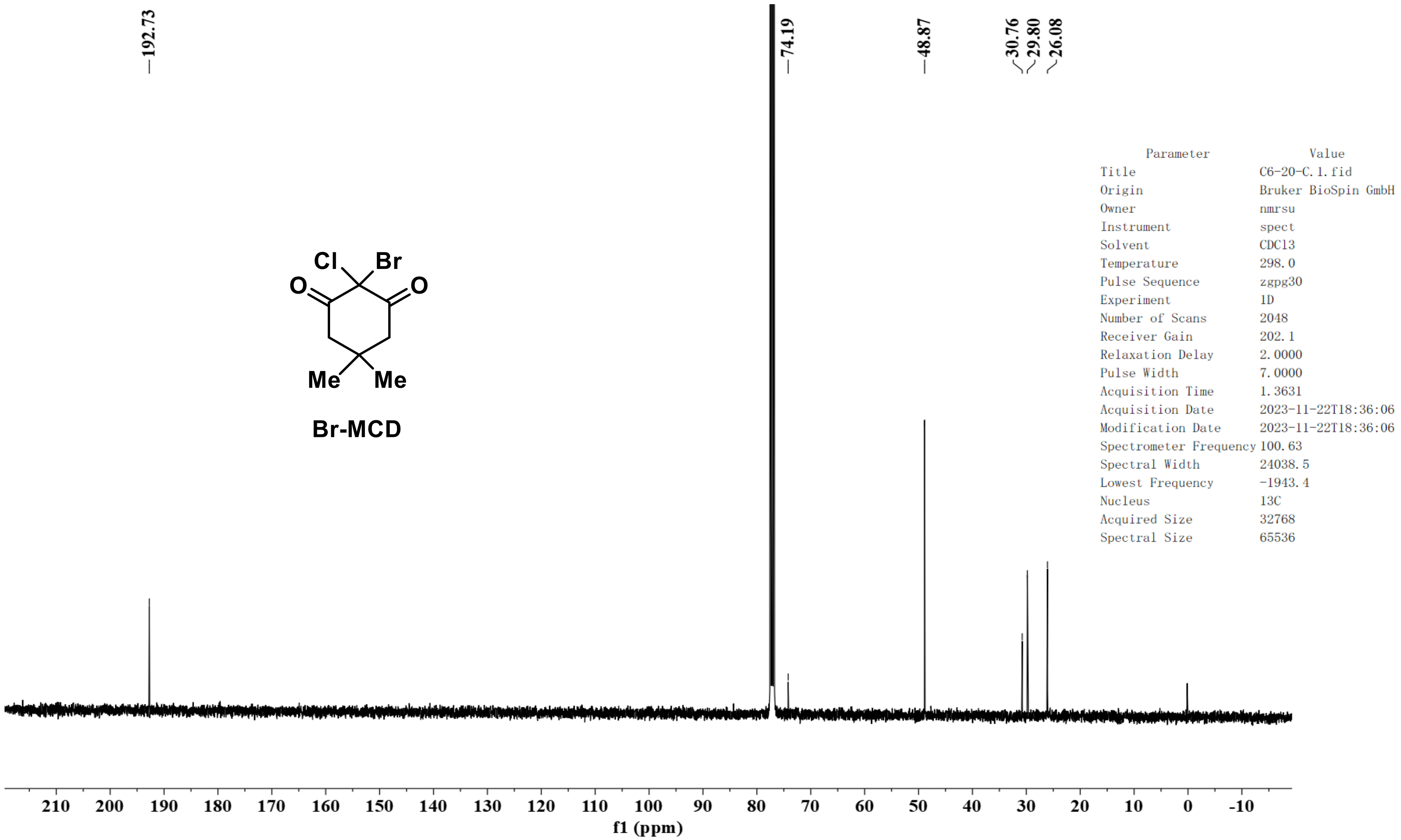
192.73
74.19
48.87
30.76
29.80
26.08
Cl
Br
O
O
Me
Me
Br-MCD
Parameter Value
Title C6-20-C.1.fid
Origin Bruker BioSpin GmbH
Owner nmrsu
Instrument spect
Solvent CDCl3
Temperature 298.0
Pulse Sequence zgpg30
Experiment 1D
Number of Scans 2048
Receiver Gain 202.1
Relaxation Delay 2.0000
Pulse Width 7.0000
Acquisition Time 1.3631
Acquisition Date 2023-11-22T18:36:06
Modification Date 2023-11-22T18:36:06
Spectrometer Frequency 100.63
Spectral Width 24038.5
Lowest Frequency -1943.4
Nucleus 13C
Acquired Size 32768
Spectral Size 65536
210 200 190 180 170 160 150 140 130 120 110 100 90 80 70 60 50 40 30 20 10 0 -10
f1 (ppm)

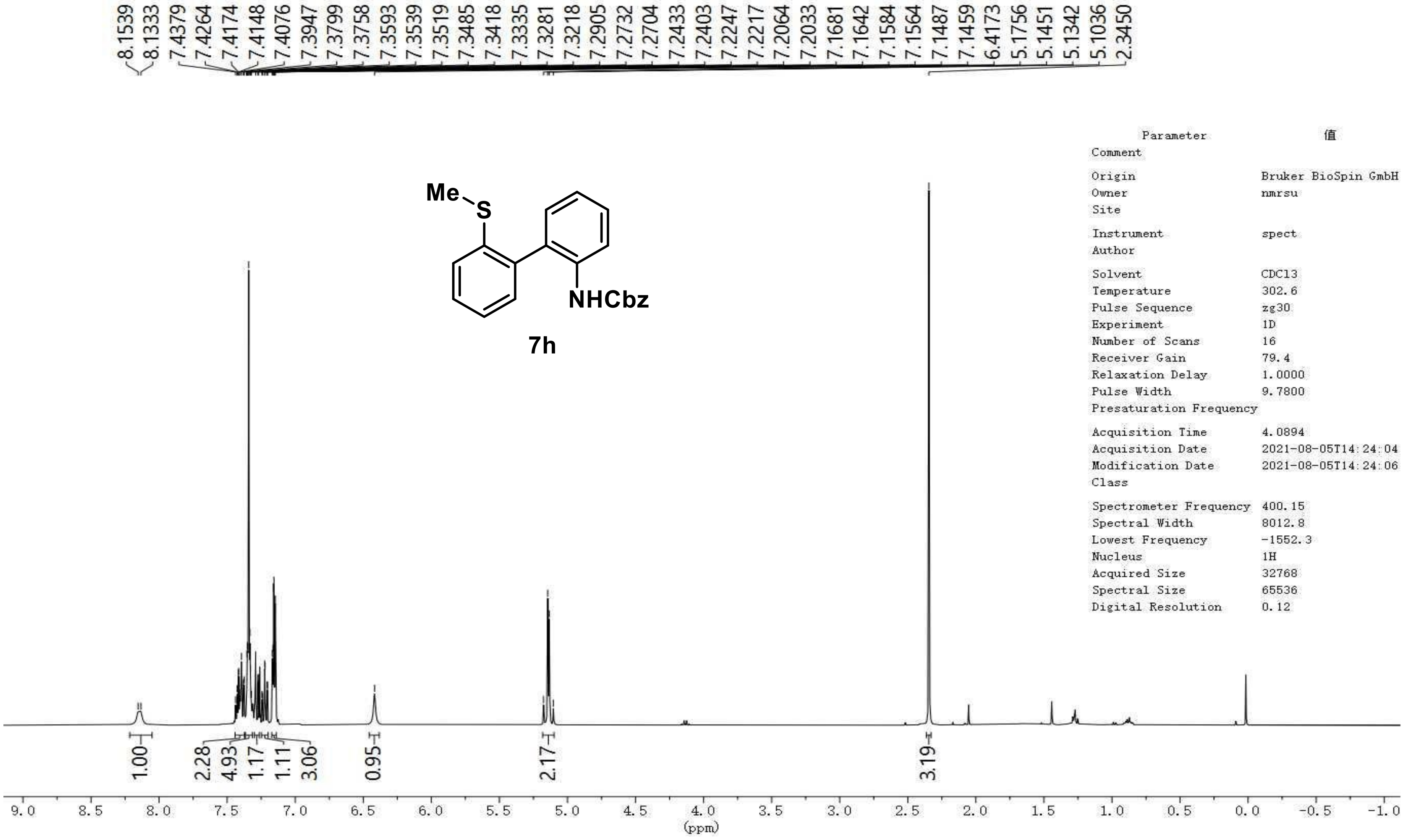
8.1539
8.1333
7.4379
7.4264
7.4174
7.4148
7.4076
7.3947
7.3799
7.3758
7.3593
7.3539
7.3519
7.3485
7.3418
7.3335
7.3281
7.3218
7.2905
7.2732
7.2704
7.2433
7.2403
7.2247
7.2217
7.2064
7.2033
7.1681
7.1642
7.1584
7.1564
7.1487
7.1459
6.4173
5.1756
5.1451
5.1342
5.1036
2.3450
Me
S
NHCbz
7h
Parameter 值
Comment
Origin Bruker BioSpin GmbH
Owner nmrsu
Site
Instrument spect
Author
Solvent CDCl3
Temperature 302.6
Pulse Sequence zg30
Experiment 1D
Number of Scans 16
Receiver Gain 79.4
Relaxation Delay 1.0000
Pulse Width 9.7800
Presaturation Frequency
Acquisition Time 4.0894
Acquisition Date 2021-08-05T14:24:04
Modification Date 2021-08-05T14:24:06
Class
Spectrometer Frequency 400.15
Spectral Width 8012.8
Lowest Frequency -1552.3
Nucleus 1H
Acquired Size 32768
Spectral Size 65536
Digital Resolution 0.12
1.00
2.28
4.93
1.17
1.11
3.06
0.95
2.17
3.19
9.0 8.5 8.0 7.5 7.0 6.5 6.0 5.5 5.0 4.5 4.0 3.5 3.0 2.5 2.0 1.5 1.0 0.5 0.0 -0.5 -1.0
(ppm)

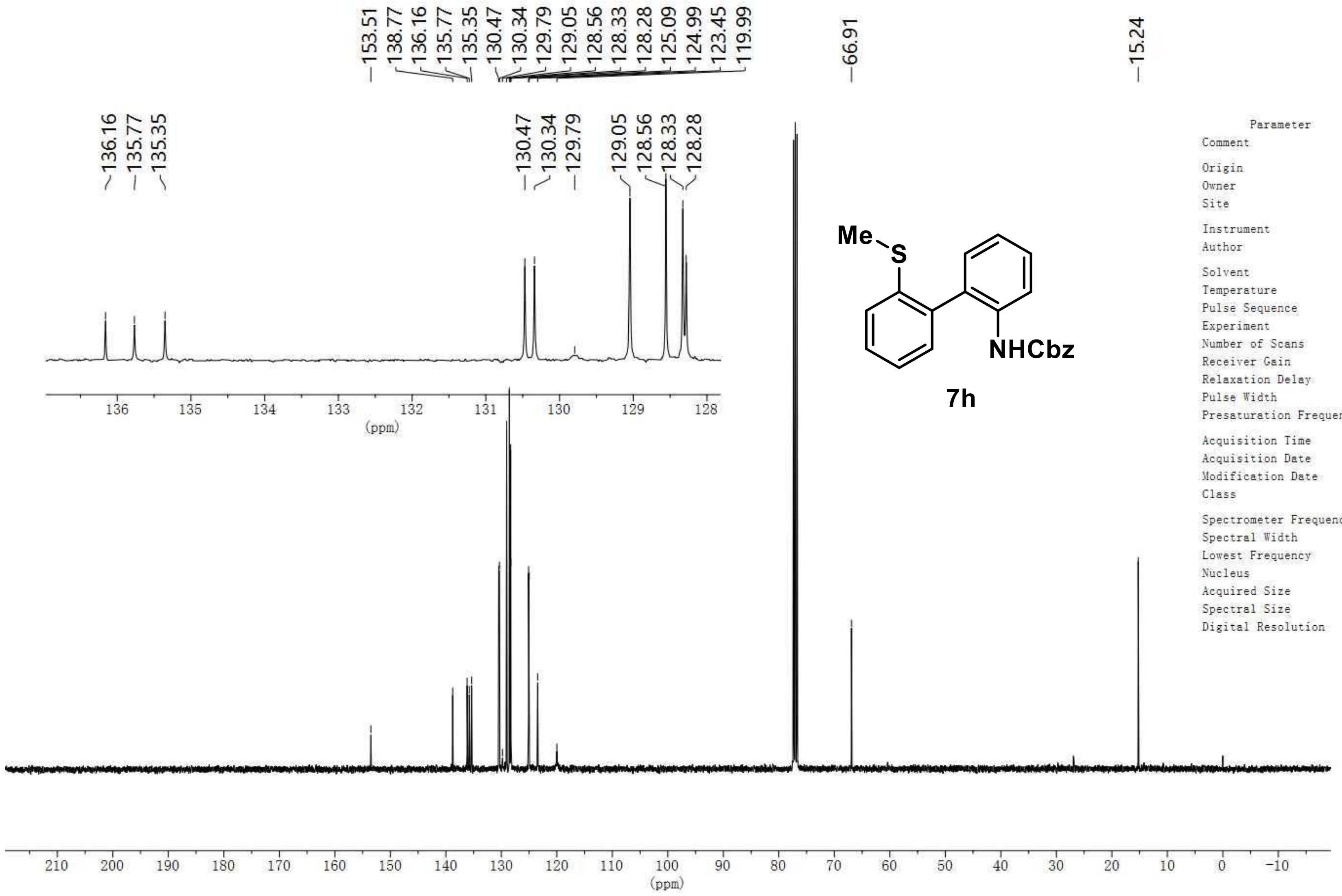

Parameter	值
Comment	
Origin	Bruker BioSpin GmbH
Owner	nmrsu
Site	
Instrument	spect
Author	
Solvent	CDCl3
Temperature	303.3
Pulse Sequence	zgpg30
Experiment	1D
Number of Scans	700
Receiver Gain	202.1
Relaxation Delay	2.0000
Pulse Width	10.5800
Presaturation Frequency	
Acquisition Time	1.3631
Acquisition Date	2021-08-05T15:05:07
Modification Date	2021-08-05T15:05:08
Class	
Spectrometer Frequency	100.63
Spectral Width	24038.5
Lowest Frequency	-1957.5
Nucleus	13C
Acquired Size	32768
Spectral Size	65536
Digital Resolution	0.37

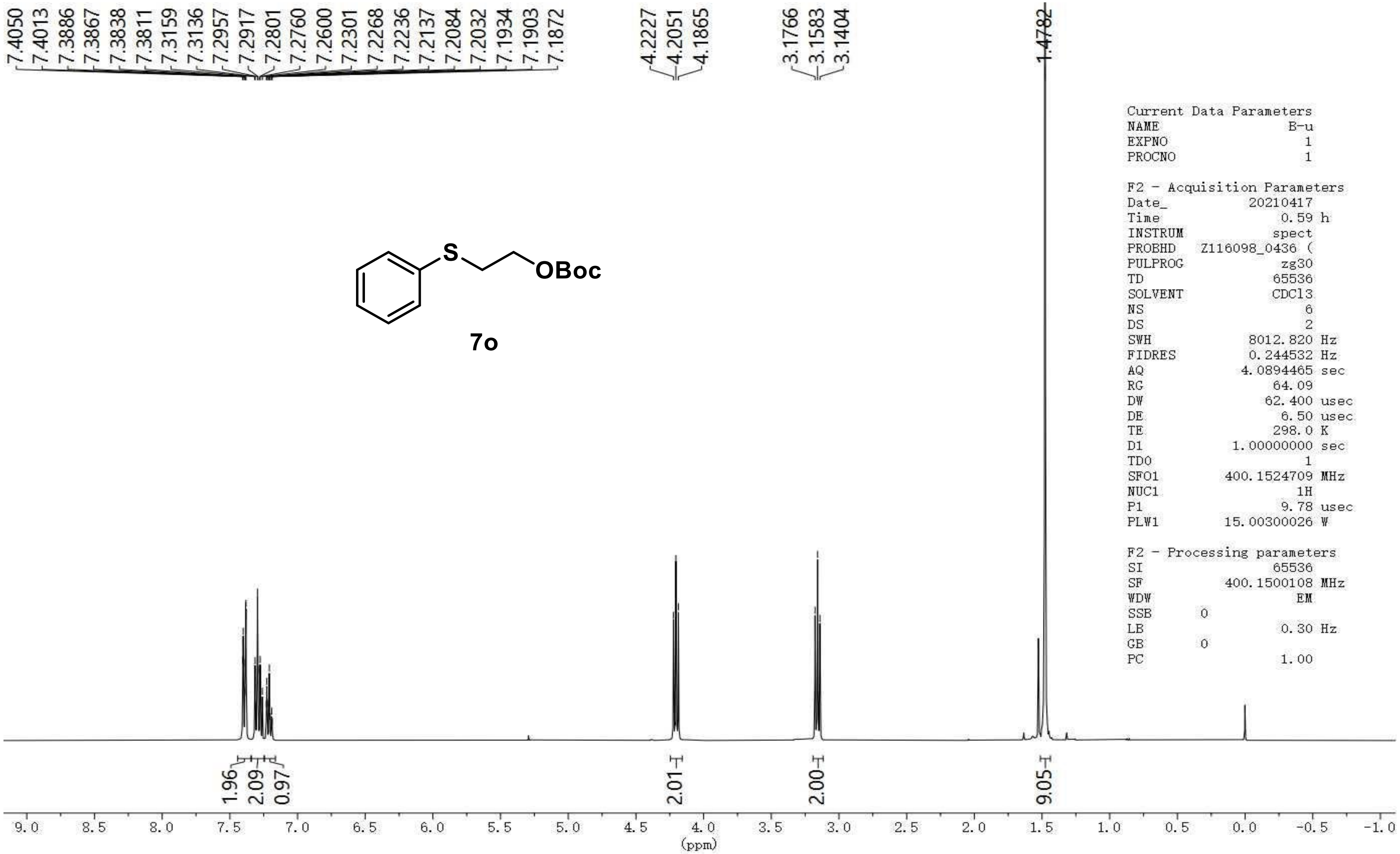
7.4050
7.4013
7.3886
7.3867
7.3838
7.3811
7.3159
7.3136
7.2957
7.2917
7.2801
7.2760
7.2600
7.2301
7.2268
7.2236
7.2137
7.2084
7.2032
7.1934
7.1903
7.1872
4.2227
4.2051
4.1865
3.1766
3.1583
3.1404
1.4782
S
OBoc
7o
Current Data Parameters
NAME B-u
EXPNO 1
PROCNO 1
F2 - Acquisition Parameters
Date_ 20210417
Time 0.59 h
INSTRUM spect
PROBHD Z116098_0436 (
PULPROG zg30
TD 65536
SOLVENT CDCl3
NS 6
DS 2
SWH 8012.820 Hz
FIDRES 0.244532 Hz
AQ 4.0894465 sec
RG 64.09
DW 62.400 usec
DE 6.50 usec
TE 298.0 K
D1 1.00000000 sec
TD0 1
SFO1 400.1524709 MHz
NUC1 1H
P1 9.78 usec
PLW1 15.00300026 W
F2 - Processing parameters
SI 65536
SF 400.1500108 MHz
WDW EM
SSB 0
LB 0.30 Hz
GB 0
PC 1.00
1.96
2.09
0.97
2.01
2.00
9.05
9.0 8.5 8.0 7.5 7.0 6.5 6.0 5.5 5.0 4.5 4.0 3.5 3.0 2.5 2.0 1.5 1.0 0.5 0.0 -0.5 -1.0
(ppm)

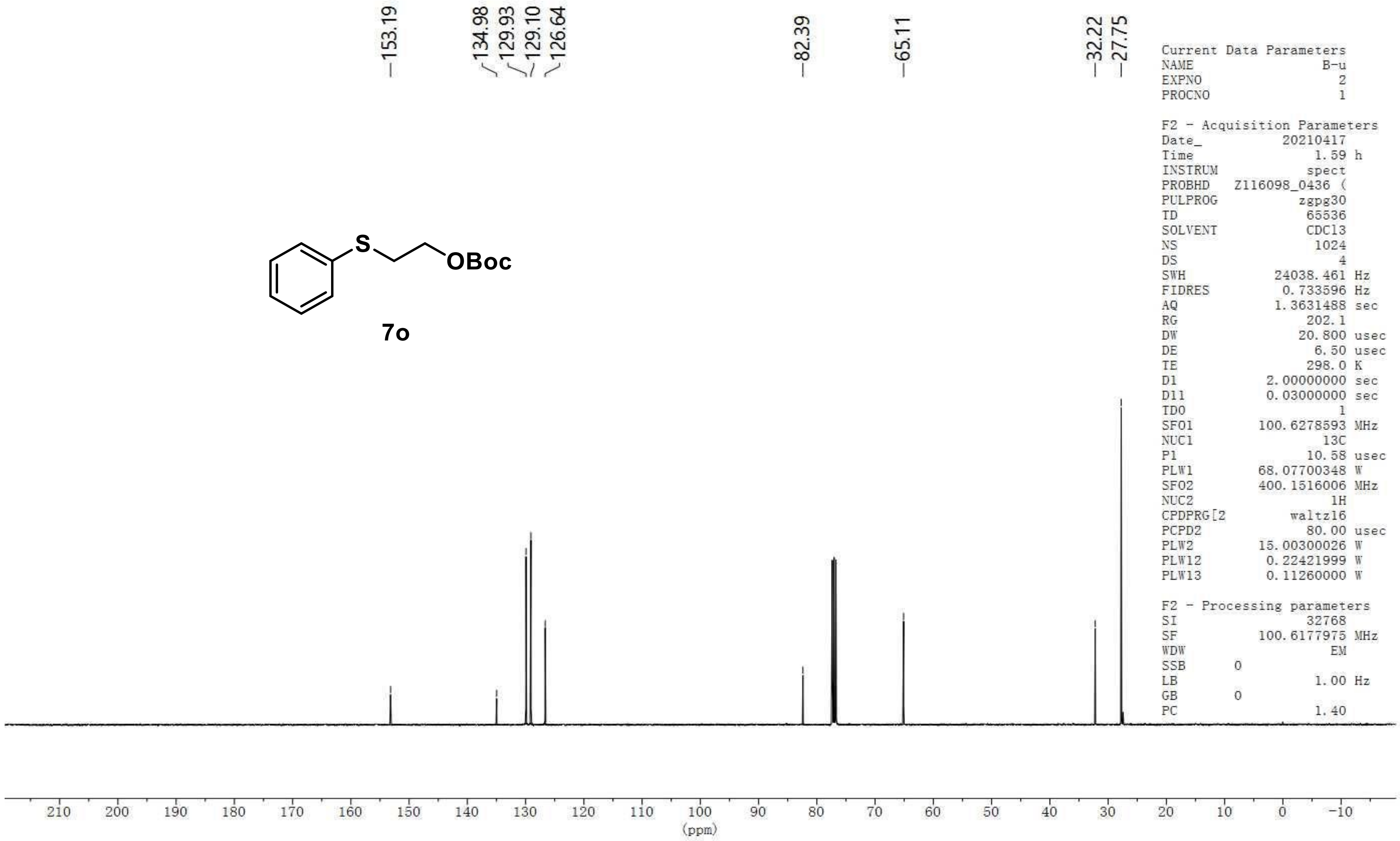
153.19
134.98
129.93
129.10
126.64
82.39
65.11
32.22
27.75
OBoc
S
7o
Current Data Parameters
NAME B-u
EXPNO 2
PROCNO 1
F2 - Acquisition Parameters
Date_ 20210417
Time 1.59 h
INSTRUM spect
PROBHD Z116098_0436 (
PULPROG zgpg30
TD 65536
SOLVENT CDCl3
NS 1024
DS 4
SWH 24038.461 Hz
FIDRES 0.733596 Hz
AQ 1.3631488 sec
RG 202.1
DW 20.800 usec
DE 6.50 usec
TE 298.0 K
D1 2.00000000 sec
D11 0.03000000 sec
TD0 1
SFO1 100.6278593 MHz
NUC1 13C
P1 10.58 usec
PLW1 68.07700348 W
SFO2 400.1516006 MHz
NUC2 1H
CPDPRG[2 waltz16
PCPD2 80.00 usec
PLW2 15.00300026 W
PLW12 0.22421999 W
PLW13 0.11260000 W
F2 - Processing parameters
SI 32768
SF 100.6177975 MHz
WDW EM
SSB 0
LB 1.00 Hz
GB 0
PC 1.40
210 200 190 180 170 160 150 140 130 120 110 100 90 80 70 60 50 40 30 20 10 0 -10
(ppm)

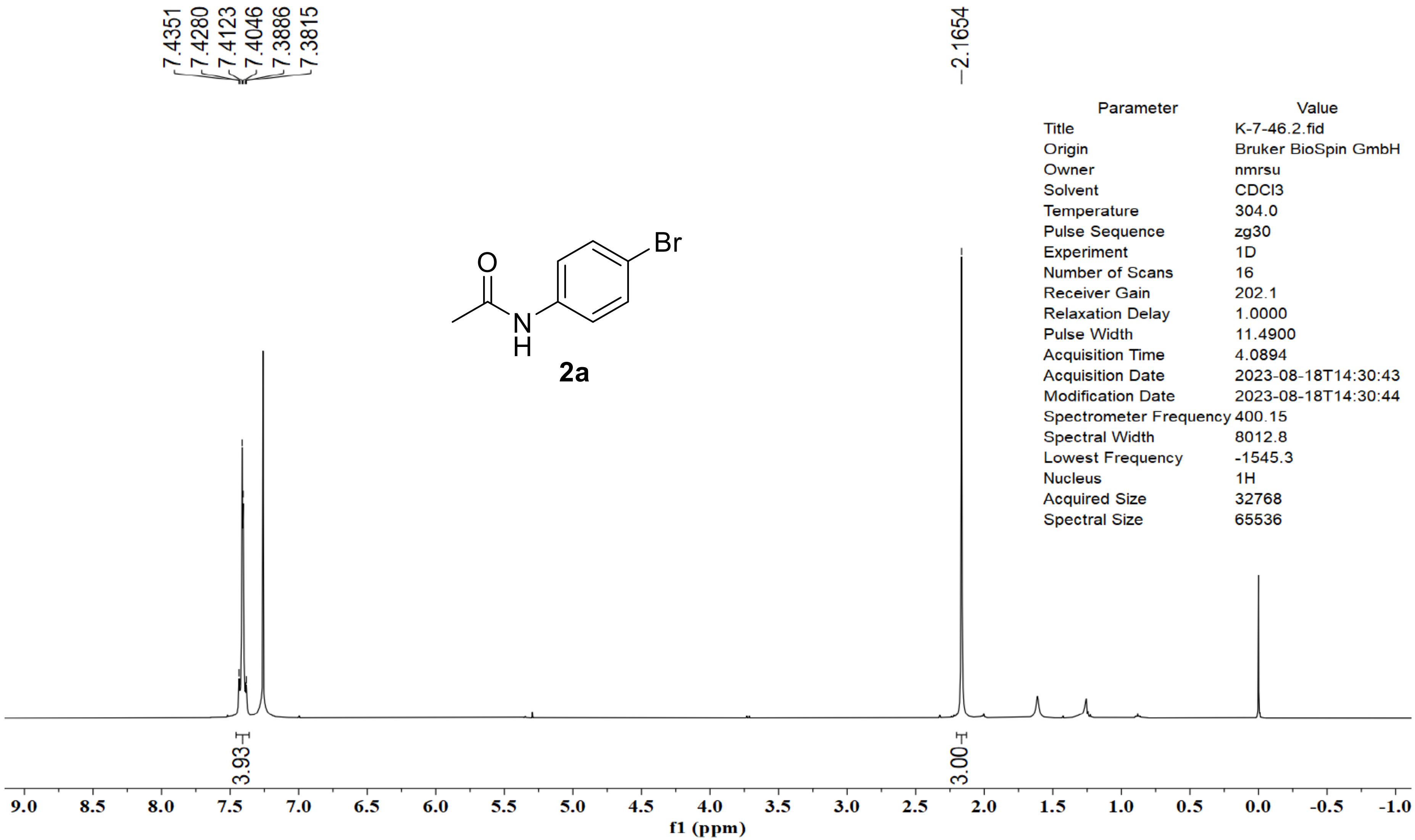

Parameter	Value
Title	K-7-46.2.fid
Origin	Bruker BioSpin GmbH
Owner	nmrsu
Solvent	CDCl3
Temperature	304.0
Pulse Sequence	zg30
Experiment	1D
Number of Scans	16
Receiver Gain	202.1
Relaxation Delay	1.0000
Pulse Width	11.4900
Acquisition Time	4.0894
Acquisition Date	2023-08-18T14:30:43
Modification Date	2023-08-18T14:30:44
Spectrometer Frequency	400.15
Spectral Width	8012.8
Lowest Frequency	-1545.3
Nucleus	1H
Acquired Size	32768
Spectral Size	65536

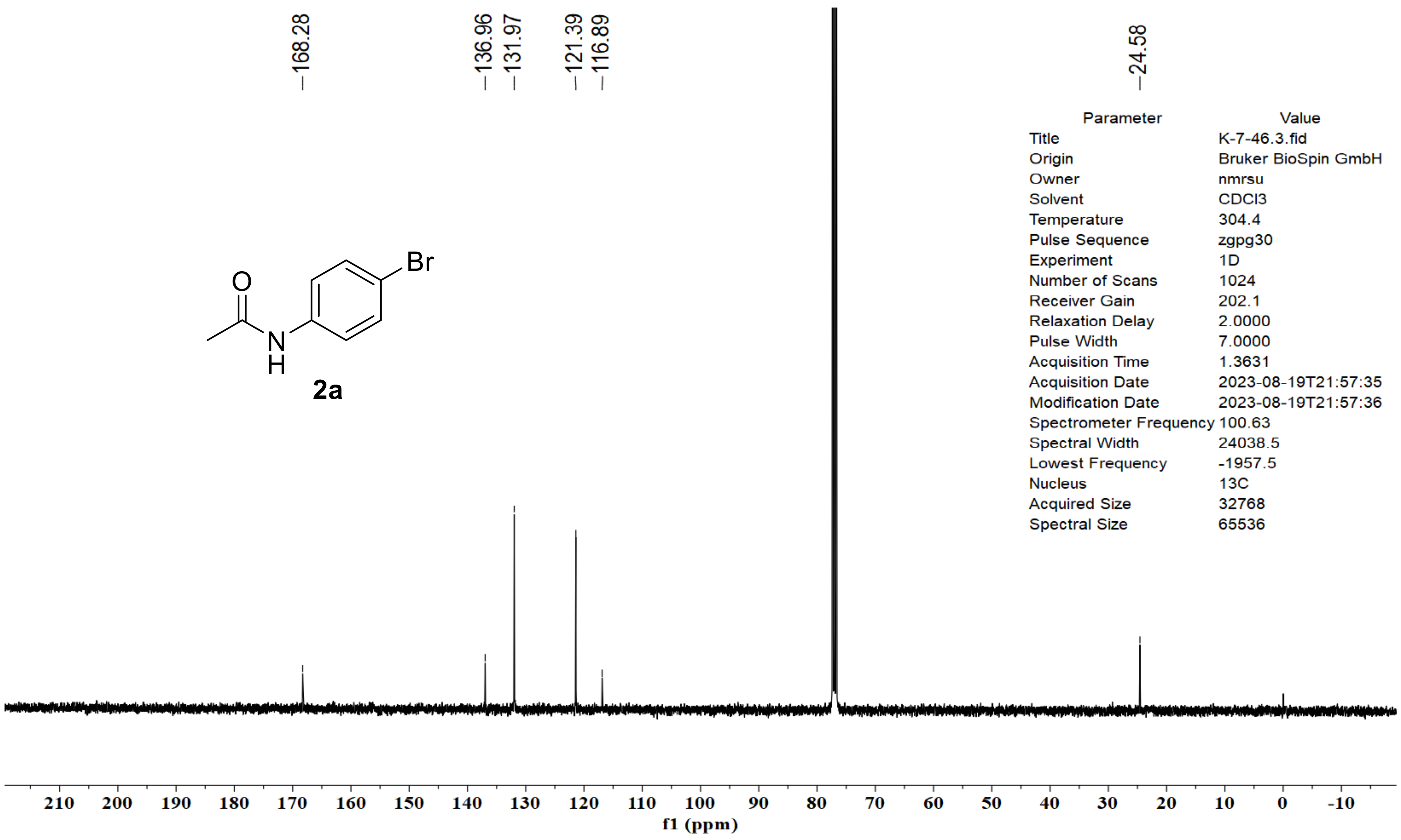
168.28
136.96
131.97
121.39
116.89
24.58
O
N
H
Br
2a
Parameter Value
Title K-7-46.3.fid
Origin Bruker BioSpin GmbH
Owner nmrsu
Solvent CDCl3
Temperature 304.4
Pulse Sequence zgpg30
Experiment 1D
Number of Scans 1024
Receiver Gain 202.1
Relaxation Delay 2.0000
Pulse Width 7.0000
Acquisition Time 1.3631
Acquisition Date 2023-08-19T21:57:35
Modification Date 2023-08-19T21:57:36
Spectrometer Frequency 100.63
Spectral Width 24038.5
Lowest Frequency -1957.5
Nucleus 13C
Acquired Size 32768
Spectral Size 65536
210 200 190 180 170 160 150 140 130 120 110 100 90 80 70 60 50 40 30 20 10 0 -10
f1 (ppm)

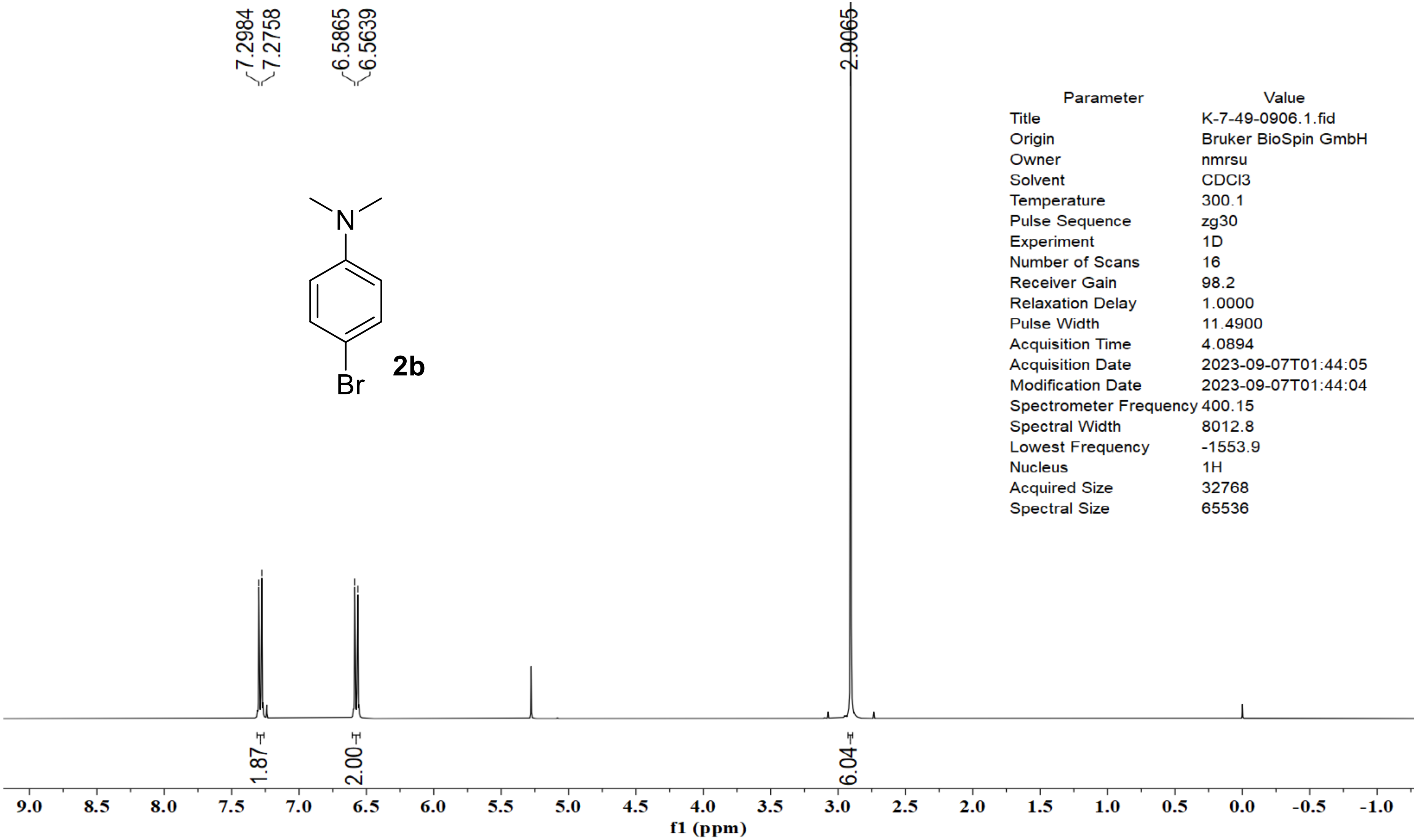

2b

Parameter	Value
Title	K-7-49-0906.1.fid
Origin	Bruker BioSpin GmbH
Owner	nmrsu
Solvent	CDCl3
Temperature	300.1
Pulse Sequence	zg30
Experiment	1D
Number of Scans	16
Receiver Gain	98.2
Relaxation Delay	1.0000
Pulse Width	11.4900
Acquisition Time	4.0894
Acquisition Date	2023-09-07T01:44:05
Modification Date	2023-09-07T01:44:04
Spectrometer Frequency	400.15
Spectral Width	8012.8
Lowest Frequency	-1553.9
Nucleus	1H
Acquired Size	32768
Spectral Size	65536

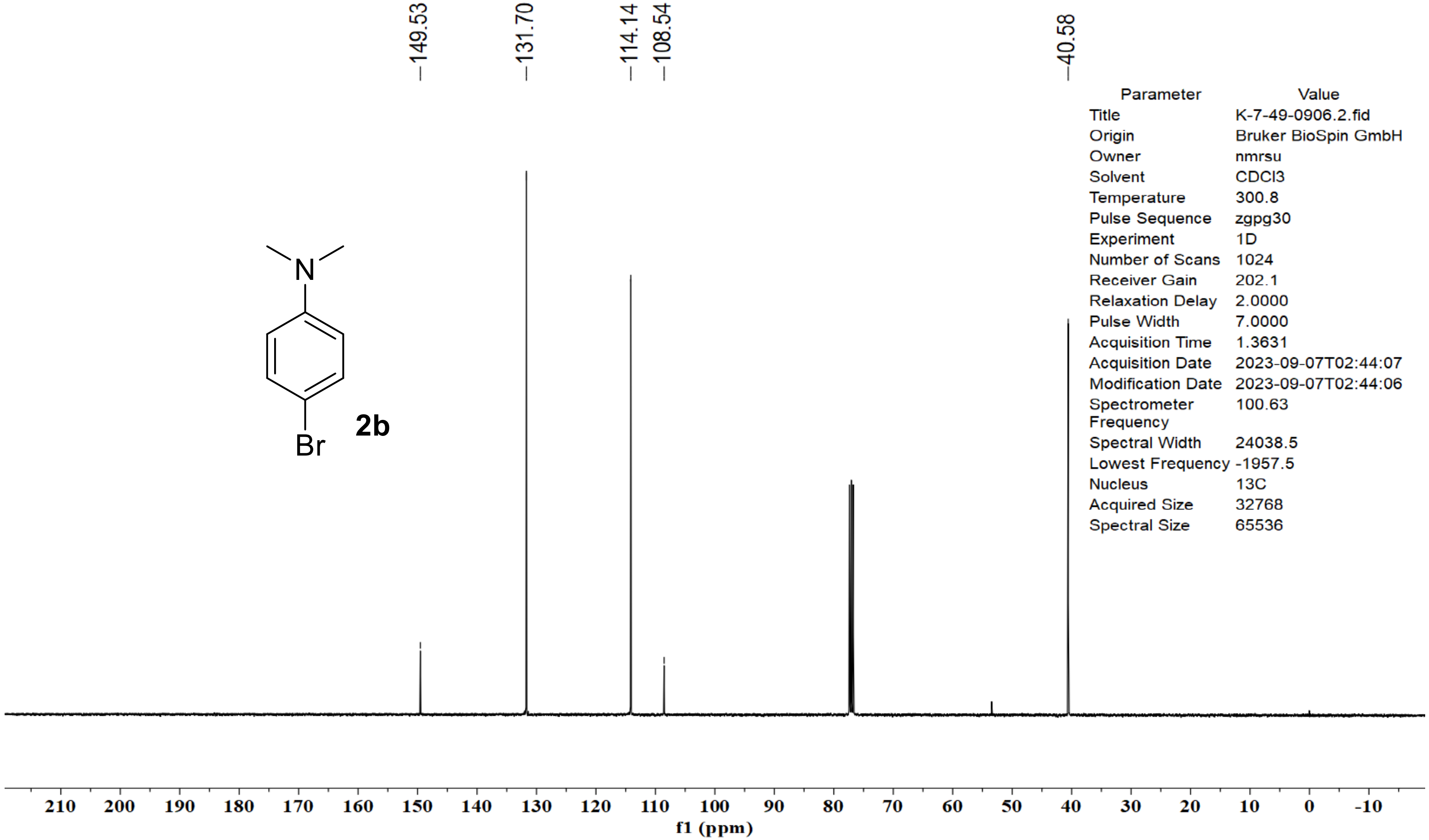

Parameter	Value
Title	K-7-49-0906.2.fid
Origin	Bruker BioSpin GmbH
Owner	nmrsu
Solvent	CDCl3
Temperature	300.8
Pulse Sequence	zgpg30
Experiment	1D
Number of Scans	1024
Receiver Gain	202.1
Relaxation Delay	2.0000
Pulse Width	7.0000
Acquisition Time	1.3631
Acquisition Date	2023-09-07T02:44:07
Modification Date	2023-09-07T02:44:06
Spectrometer Frequency	100.63
Spectral Width	24038.5
Lowest Frequency	-1957.5
Nucleus	13C
Acquired Size	32768
Spectral Size	65536

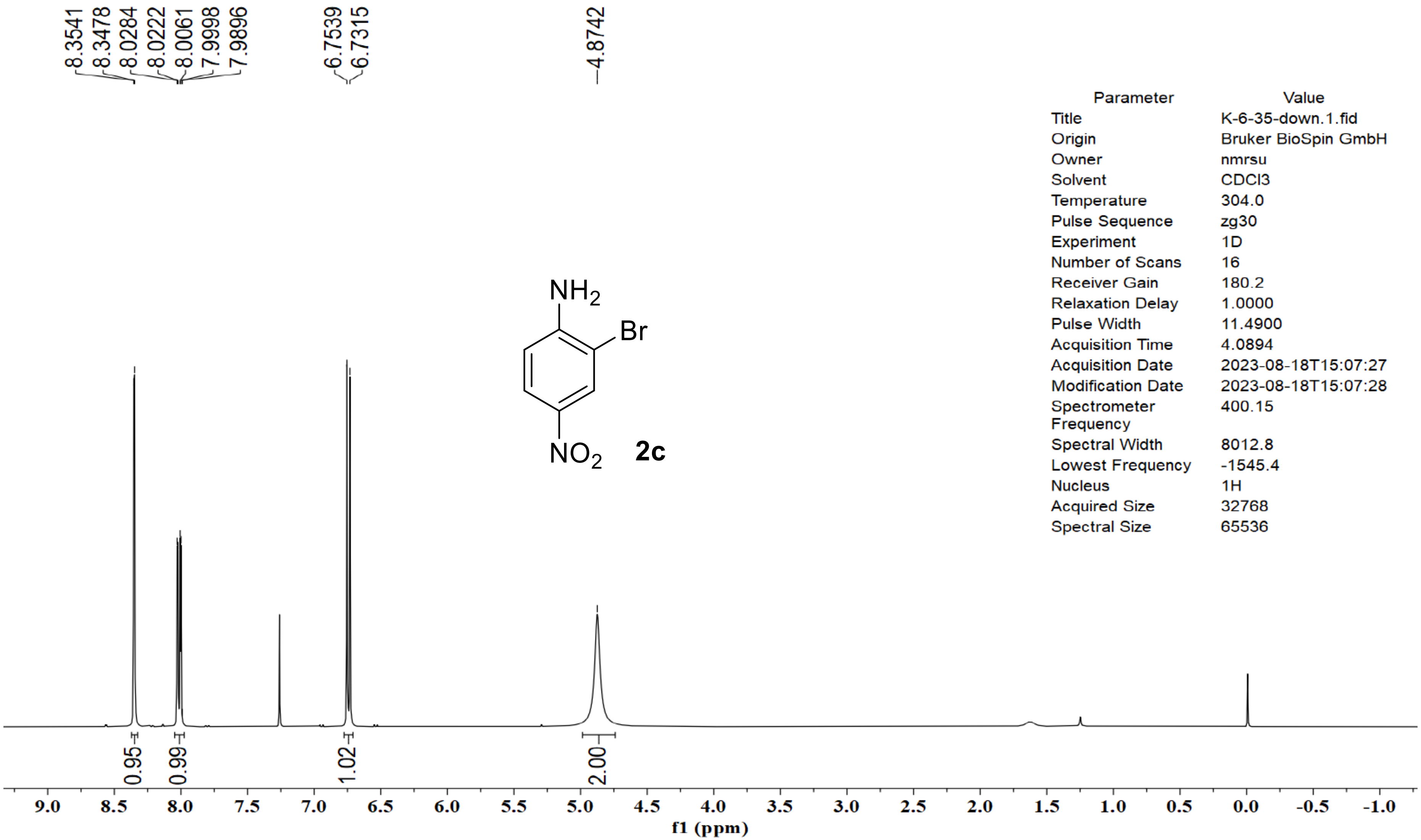

Parameter	Value
Title	K-6-35-down.1.fid
Origin	Bruker BioSpin GmbH
Owner	nmrsu
Solvent	CDCl3
Temperature	304.0
Pulse Sequence	zg30
Experiment	1D
Number of Scans	16
Receiver Gain	180.2
Relaxation Delay	1.0000
Pulse Width	11.4900
Acquisition Time	4.0894
Acquisition Date	2023-08-18T15:07:27
Modification Date	2023-08-18T15:07:28
Spectrometer Frequency	400.15
Spectral Width	8012.8
Lowest Frequency	-1545.4
Nucleus	1H
Acquired Size	32768
Spectral Size	65536

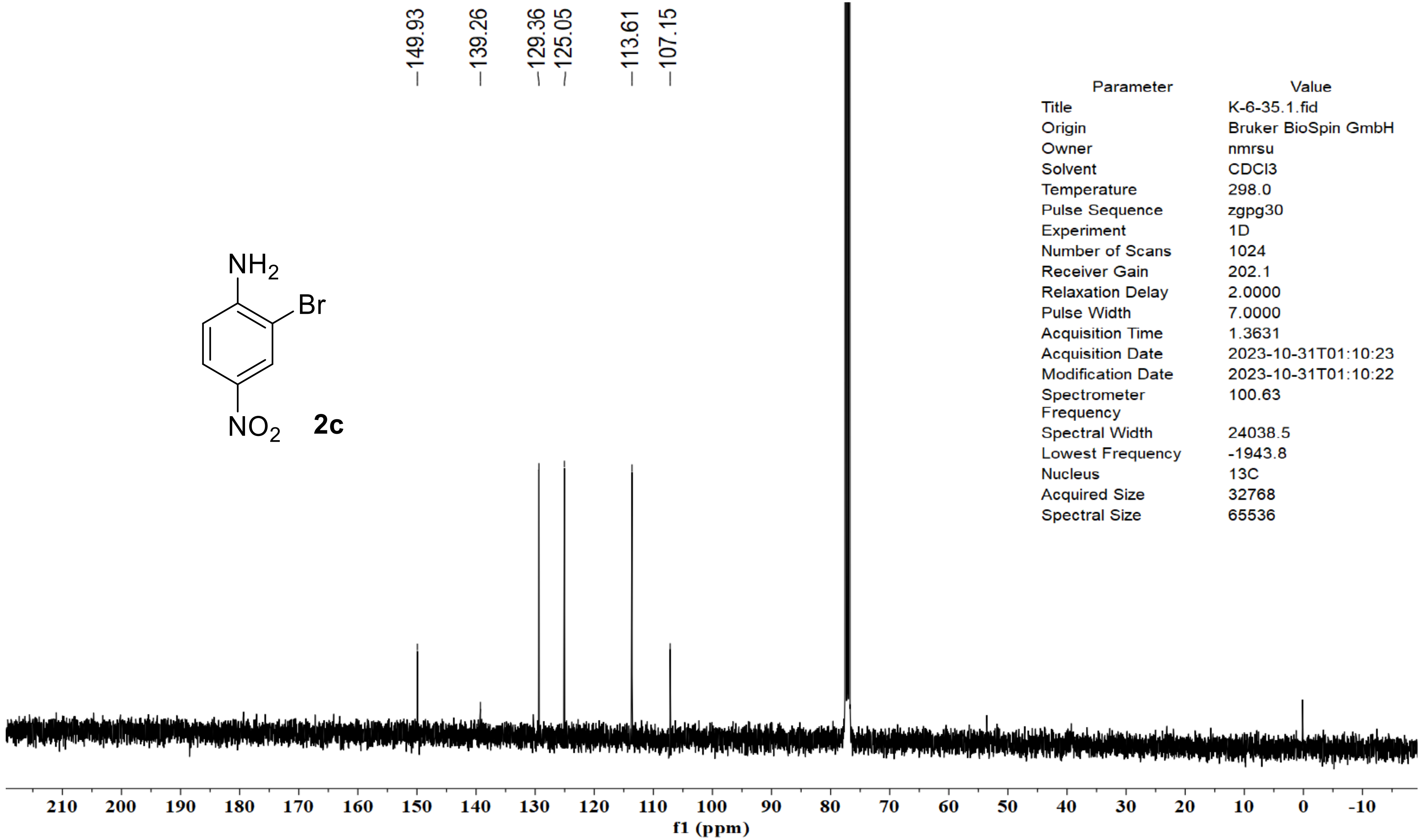

2c

Parameter	Value
Title	K-6-35.1.fid
Origin	Bruker BioSpin GmbH
Owner	nmrsu
Solvent	CDCl3
Temperature	298.0
Pulse Sequence	zgpg30
Experiment	1D
Number of Scans	1024
Receiver Gain	202.1
Relaxation Delay	2.0000
Pulse Width	7.0000
Acquisition Time	1.3631
Acquisition Date	2023-10-31T01:10:23
Modification Date	2023-10-31T01:10:22
Spectrometer Frequency	100.63
Spectral Width	24038.5
Lowest Frequency	-1943.8
Nucleus	13C
Acquired Size	32768
Spectral Size	65536

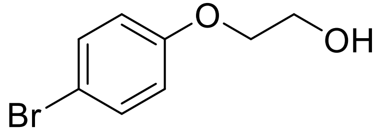

2d

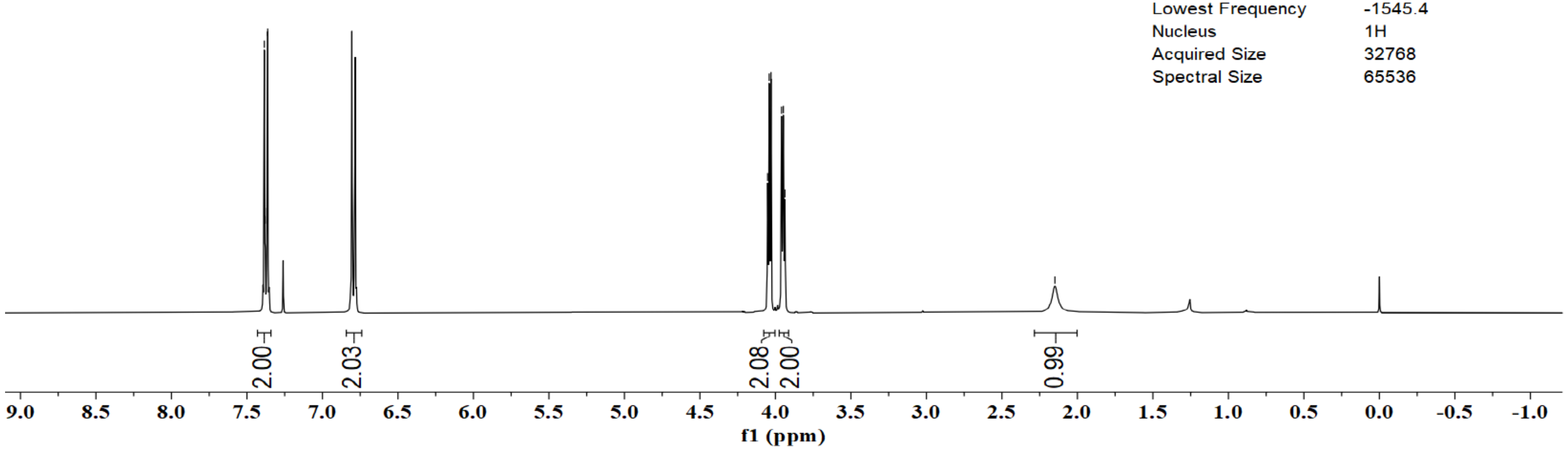

Parameter	Value
Title	K-6-49.1.fid
Origin	Bruker BioSpin GmbH
Owner	nmrsu
Solvent	CDCl3
Temperature	300.2
Pulse Sequence	zg30
Experiment	1D
Number of Scans	16
Receiver Gain	143.4
Relaxation Delay	1.0000
Pulse Width	11.4900
Acquisition Time	4.0894
Acquisition Date	2023-08-29T15:39:07
Modification Date	2023-08-29T15:39:08
Spectrometer Frequency	400.15
Spectral Width	8012.8
Lowest Frequency	-1545.4
Nucleus	1H
Acquired Size	32768
Spectral Size	65536

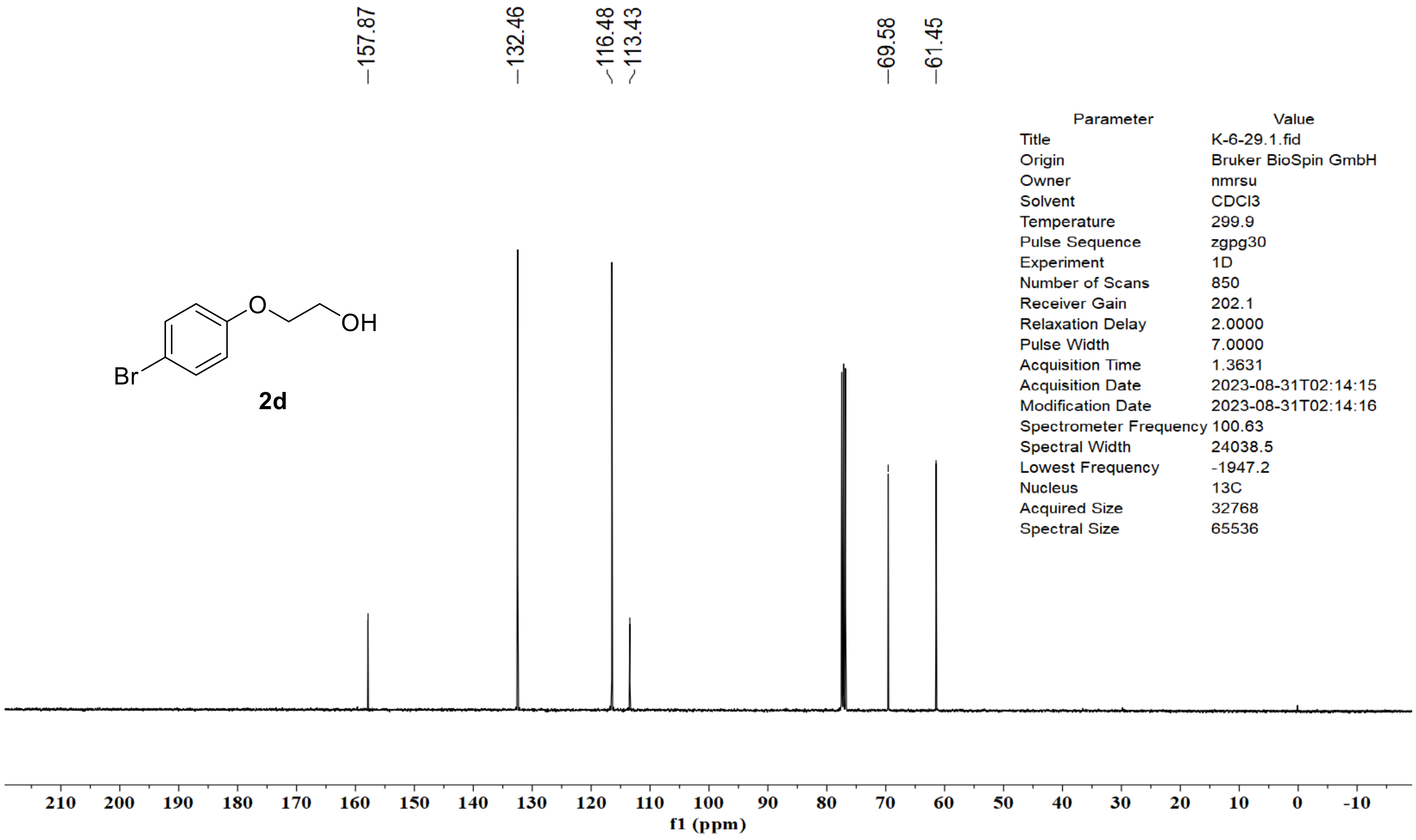

Parameter	Value
Title	K-6-29.1.fid
Origin	Bruker BioSpin GmbH
Owner	nmrsu
Solvent	CDCl3
Temperature	299.9
Pulse Sequence	zgpg30
Experiment	1D
Number of Scans	850
Receiver Gain	202.1
Relaxation Delay	2.0000
Pulse Width	7.0000
Acquisition Time	1.3631
Acquisition Date	2023-08-31T02:14:15
Modification Date	2023-08-31T02:14:16
Spectrometer Frequency	100.63
Spectral Width	24038.5
Lowest Frequency	-1947.2
Nucleus	13C
Acquired Size	32768
Spectral Size	65536

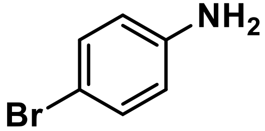

2e

Parameter	Value
Title	K-8-20t.4.fid
Origin	Bruker BioSpin GmbH
Owner	nmrsu
Solvent	CDCl3
Temperature	298.0
Pulse Sequence	zg30
Experiment	1D
Number of Scans	16
Receiver Gain	91.8
Relaxation Delay	1.0000
Pulse Width	8.0000
Acquisition Time	3.9977
Acquisition Date	2024-01-29T18:15:40
Modification Date	2024-01-29T18:16:19
Spectrometer Frequency	400.13
Spectral Width	8196.7
Lowest Frequency	-1627.6
Nucleus	1H
Acquired Size	32768
Spectral Size	65536

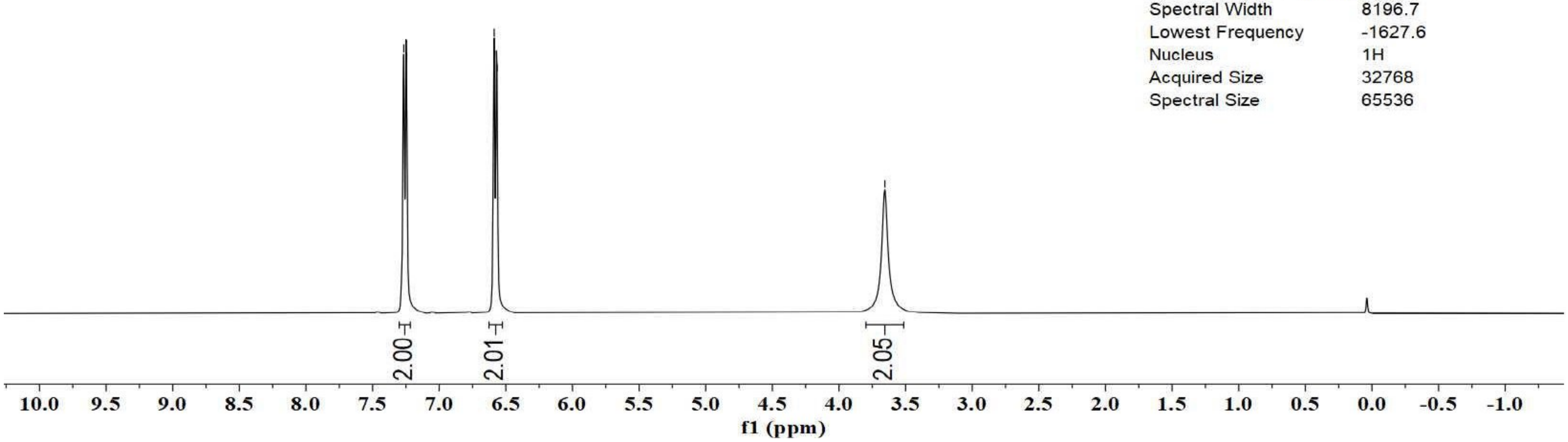

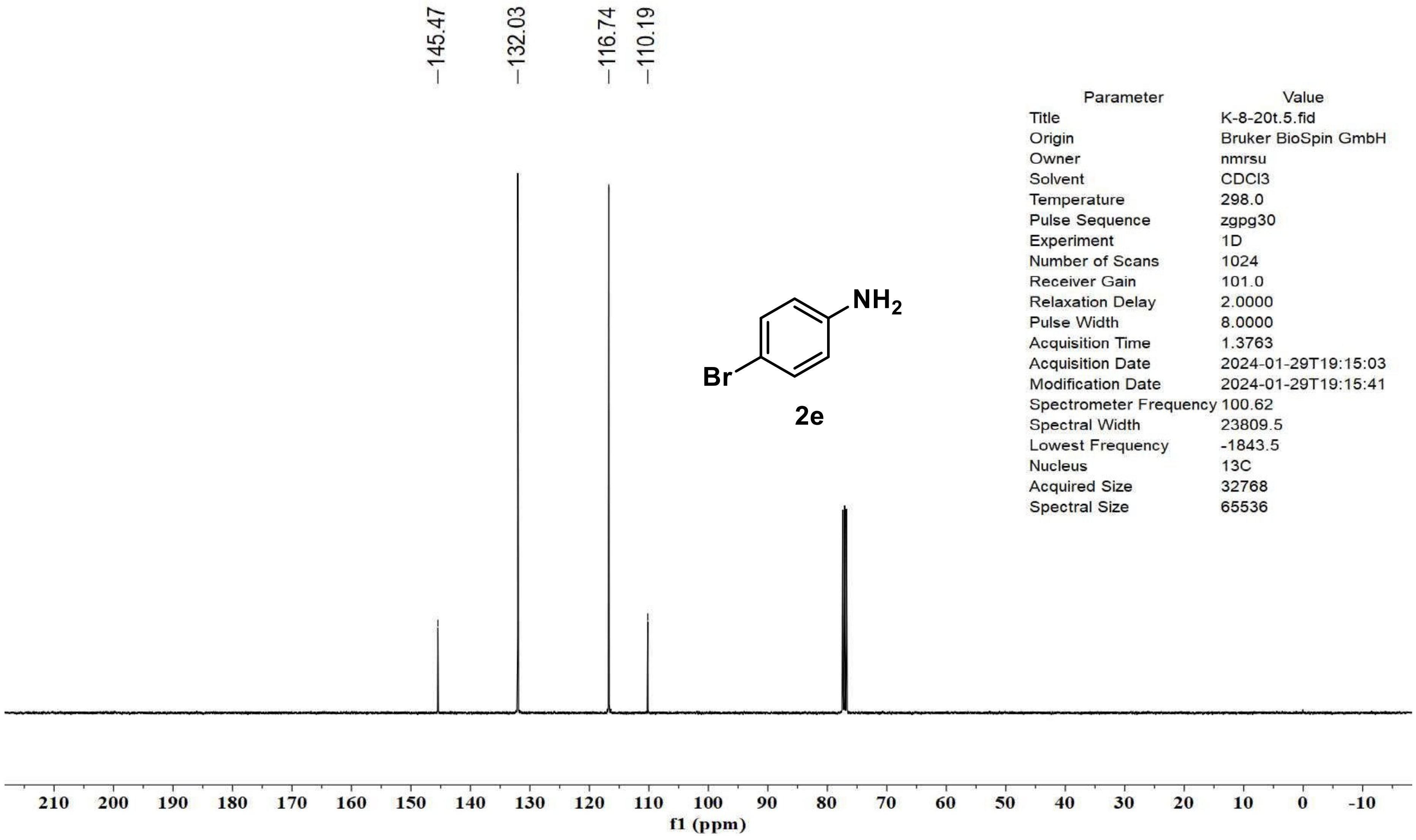

Parameter	Value
Title	K-8-20t.5.fid
Origin	Bruker BioSpin GmbH
Owner	nmrsu
Solvent	CDCl3
Temperature	298.0
Pulse Sequence	zgpg30
Experiment	1D
Number of Scans	1024
Receiver Gain	101.0
Relaxation Delay	2.0000
Pulse Width	8.0000
Acquisition Time	1.3763
Acquisition Date	2024-01-29T19:15:03
Modification Date	2024-01-29T19:15:41
Spectrometer Frequency	100.62
Spectral Width	23809.5
Lowest Frequency	-1843.5
Nucleus	13C
Acquired Size	32768
Spectral Size	65536

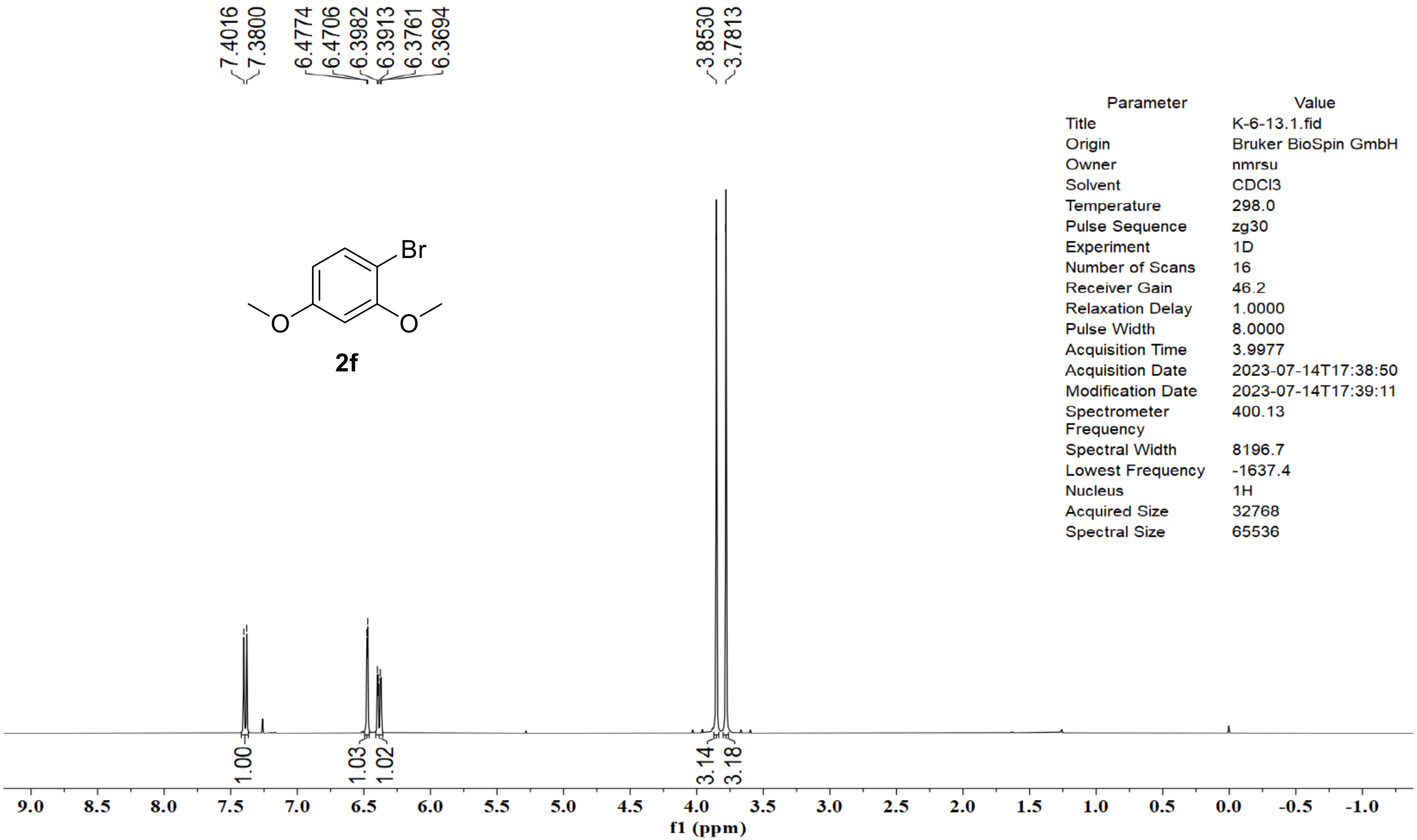

Parameter	Value
Title	K-6-13.1.fid
Origin	Bruker BioSpin GmbH
Owner	nmrsu
Solvent	CDCl3
Temperature	298.0
Pulse Sequence	zg30
Experiment	1D
Number of Scans	16
Receiver Gain	46.2
Relaxation Delay	1.0000
Pulse Width	8.0000
Acquisition Time	3.9977
Acquisition Date	2023-07-14T17:38:50
Modification Date	2023-07-14T17:39:11
Spectrometer Frequency	400.13
Spectral Width	8196.7
Lowest Frequency	-1637.4
Nucleus	1H
Acquired Size	32768
Spectral Size	65536

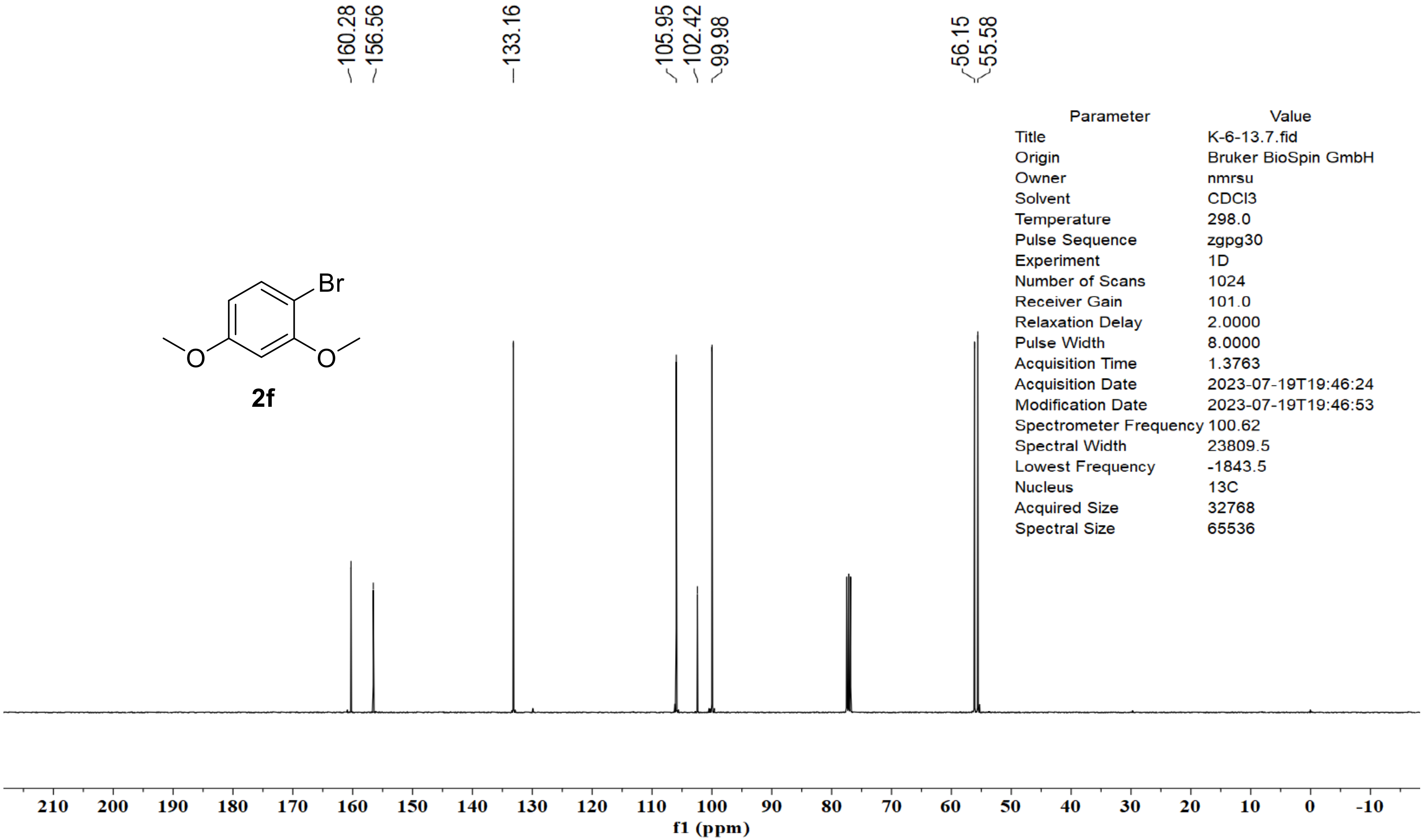

Parameter	Value
Title	K-6-13.7.fid
Origin	Bruker BioSpin GmbH
Owner	nmrsu
Solvent	CDCl3
Temperature	298.0
Pulse Sequence	zgpg30
Experiment	1D
Number of Scans	1024
Receiver Gain	101.0
Relaxation Delay	2.0000
Pulse Width	8.0000
Acquisition Time	1.3763
Acquisition Date	2023-07-19T19:46:24
Modification Date	2023-07-19T19:46:53
Spectrometer Frequency	100.62
Spectral Width	23809.5
Lowest Frequency	-1843.5
Nucleus	13C
Acquired Size	32768
Spectral Size	65536

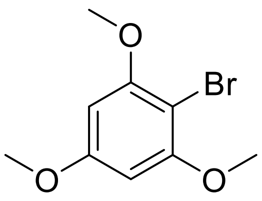

2g

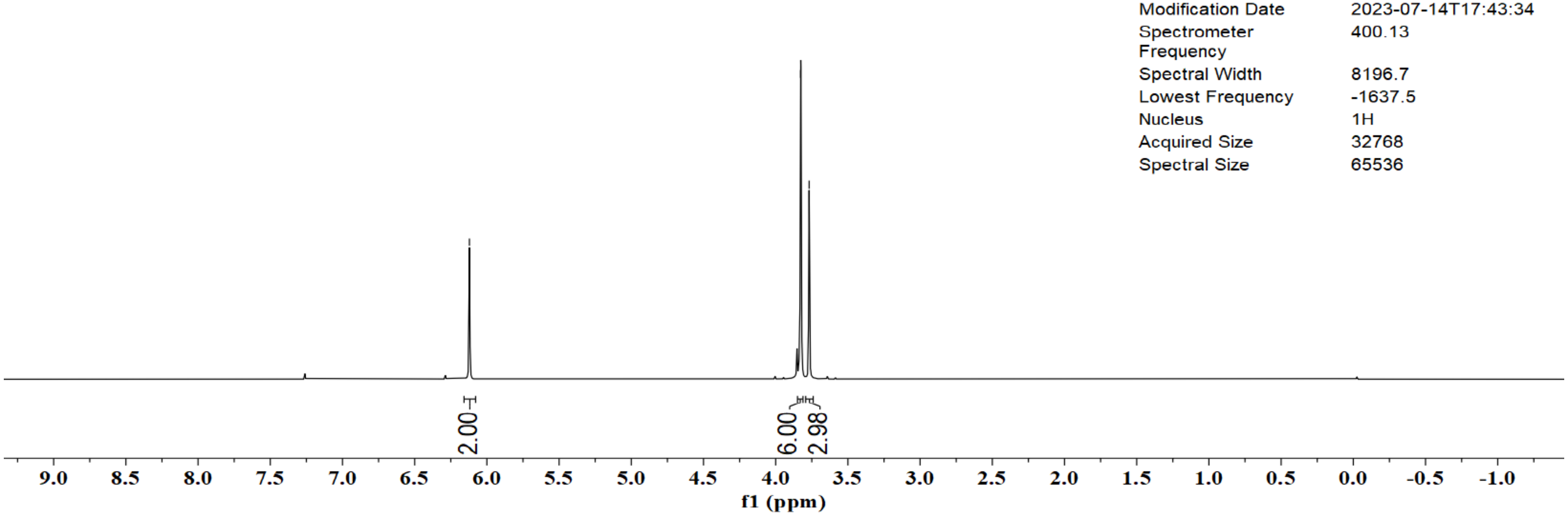

Parameter	Value
Title	K-6-16.2.fid
Origin	Bruker BioSpin GmbH
Owner	nmrsu
Solvent	CDCl3
Temperature	298.0
Pulse Sequence	zg30
Experiment	1D
Number of Scans	16
Receiver Gain	32.0
Relaxation Delay	1.0000
Pulse Width	8.0000
Acquisition Time	3.9977
Acquisition Date	2023-07-14T17:43:13
Modification Date	2023-07-14T17:43:34
Spectrometer Frequency	400.13
Spectral Width	8196.7
Lowest Frequency	-1637.5
Nucleus	1H
Acquired Size	32768
Spectral Size	65536

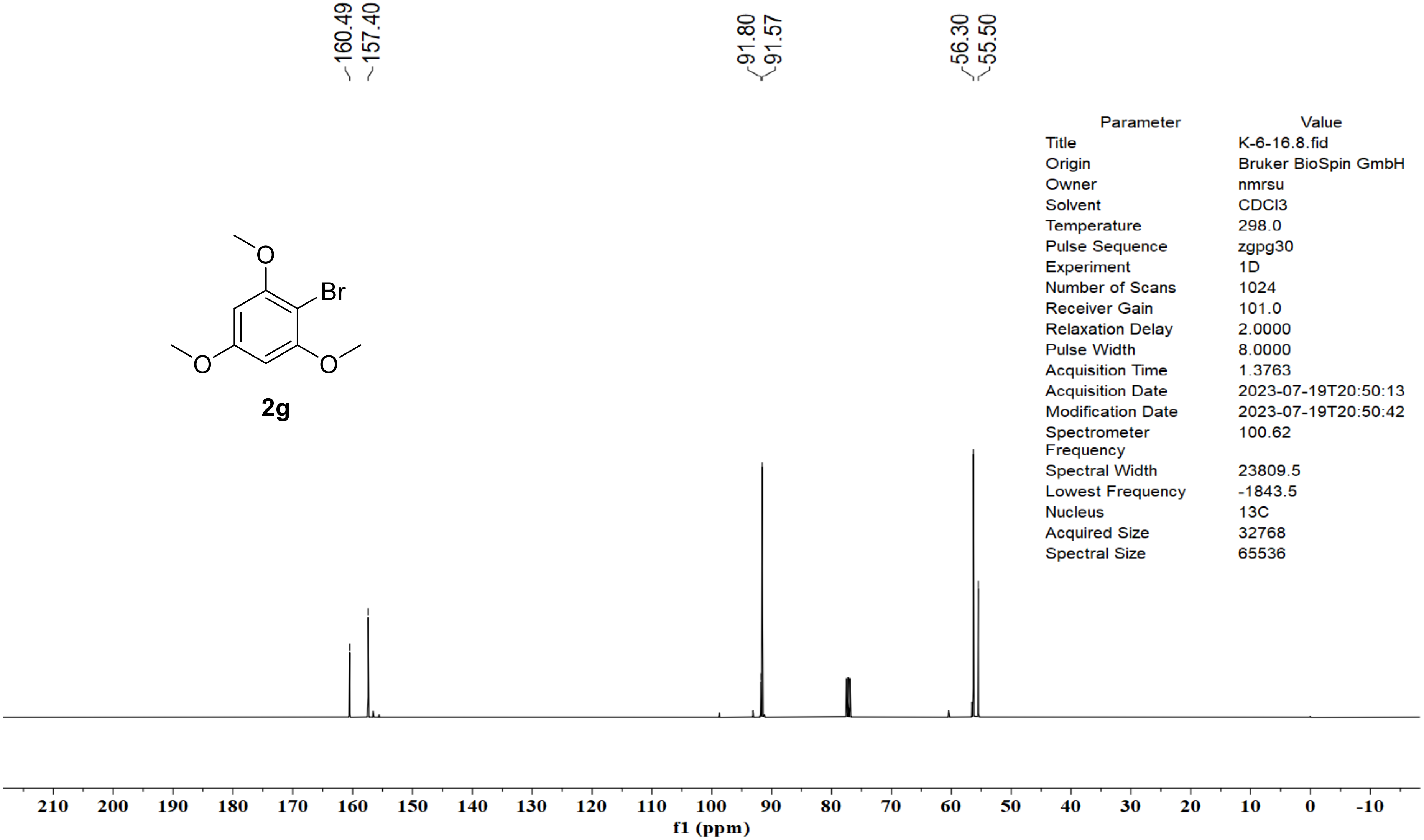

2g

Parameter	Value
Title	K-6-16.8.fid
Origin	Bruker BioSpin GmbH
Owner	nmrsu
Solvent	CDCl3
Temperature	298.0
Pulse Sequence	zgpg30
Experiment	1D
Number of Scans	1024
Receiver Gain	101.0
Relaxation Delay	2.0000
Pulse Width	8.0000
Acquisition Time	1.3763
Acquisition Date	2023-07-19T20:50:13
Modification Date	2023-07-19T20:50:42
Spectrometer Frequency	100.62
Spectral Width	23809.5
Lowest Frequency	-1843.5
Nucleus	13C
Acquired Size	32768
Spectral Size	65536

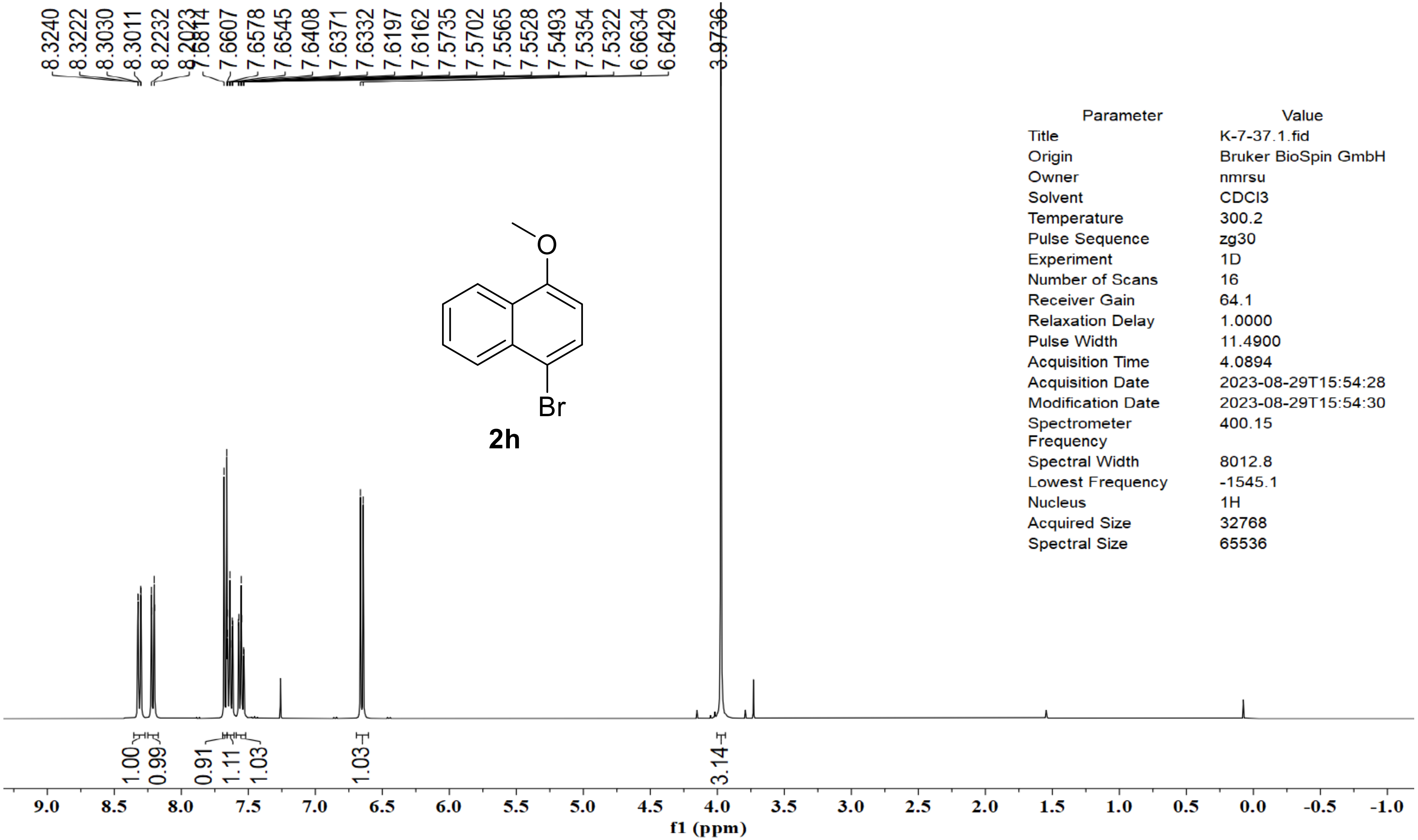

2h

Parameter	Value
Title	K-7-37.1.fid
Origin	Bruker BioSpin GmbH
Owner	nmrsu
Solvent	CDCl3
Temperature	300.2
Pulse Sequence	zg30
Experiment	1D
Number of Scans	16
Receiver Gain	64.1
Relaxation Delay	1.0000
Pulse Width	11.4900
Acquisition Time	4.0894
Acquisition Date	2023-08-29T15:54:28
Modification Date	2023-08-29T15:54:30
Spectrometer Frequency	400.15
Spectral Width	8012.8
Lowest Frequency	-1545.1
Nucleus	1H
Acquired Size	32768
Spectral Size	65536

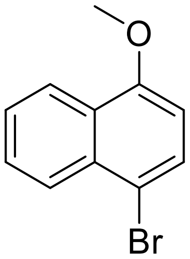

2h

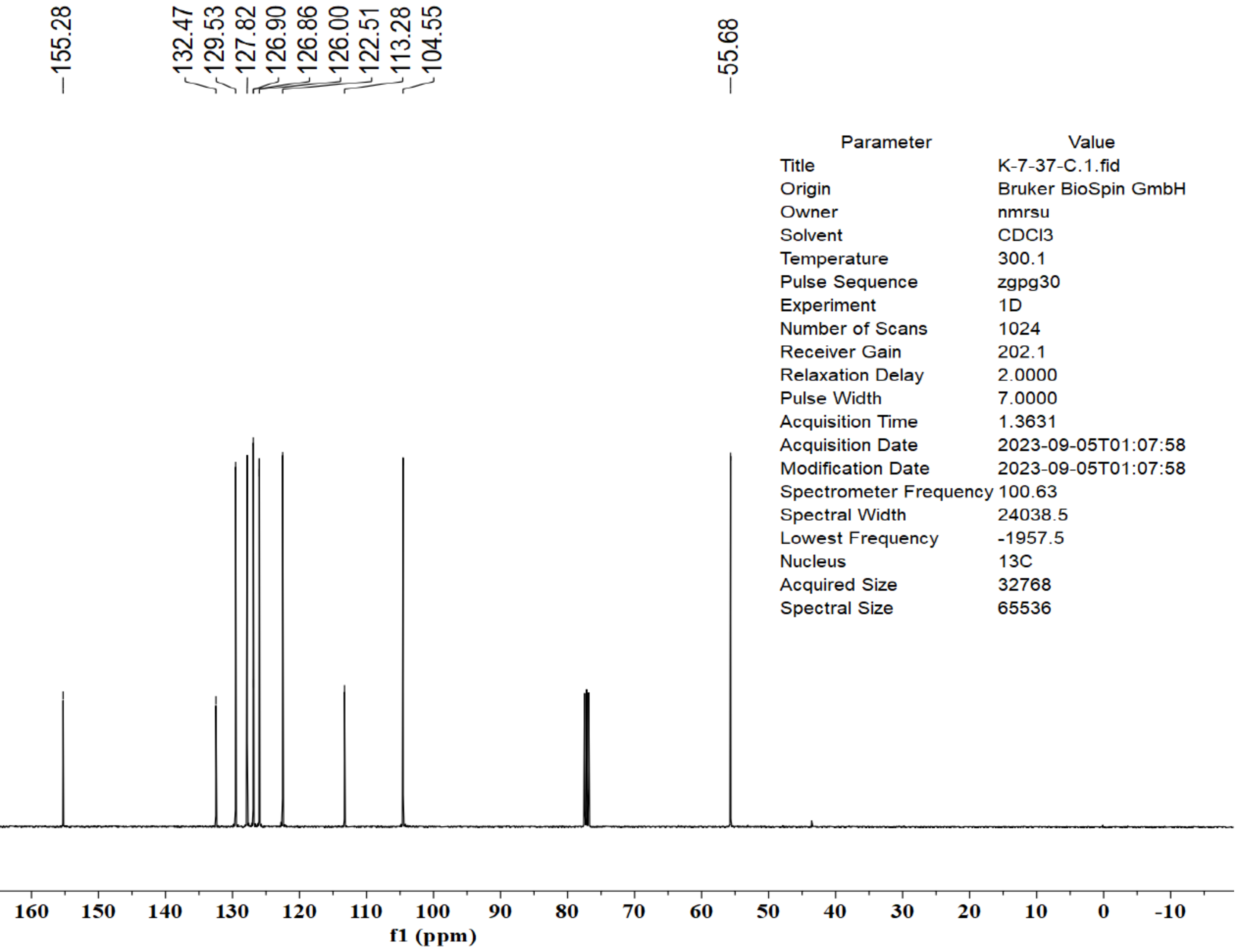

Parameter	Value
Title	K-7-37-C.1.fid
Origin	Bruker BioSpin GmbH
Owner	nmrsu
Solvent	CDCl3
Temperature	300.1
Pulse Sequence	zgpg30
Experiment	1D
Number of Scans	1024
Receiver Gain	202.1
Relaxation Delay	2.0000
Pulse Width	7.0000
Acquisition Time	1.3631
Acquisition Date	2023-09-05T01:07:58
Modification Date	2023-09-05T01:07:58
Spectrometer Frequency	100.63
Spectral Width	24038.5
Lowest Frequency	-1957.5
Nucleus	13C
Acquired Size	32768
Spectral Size	65536

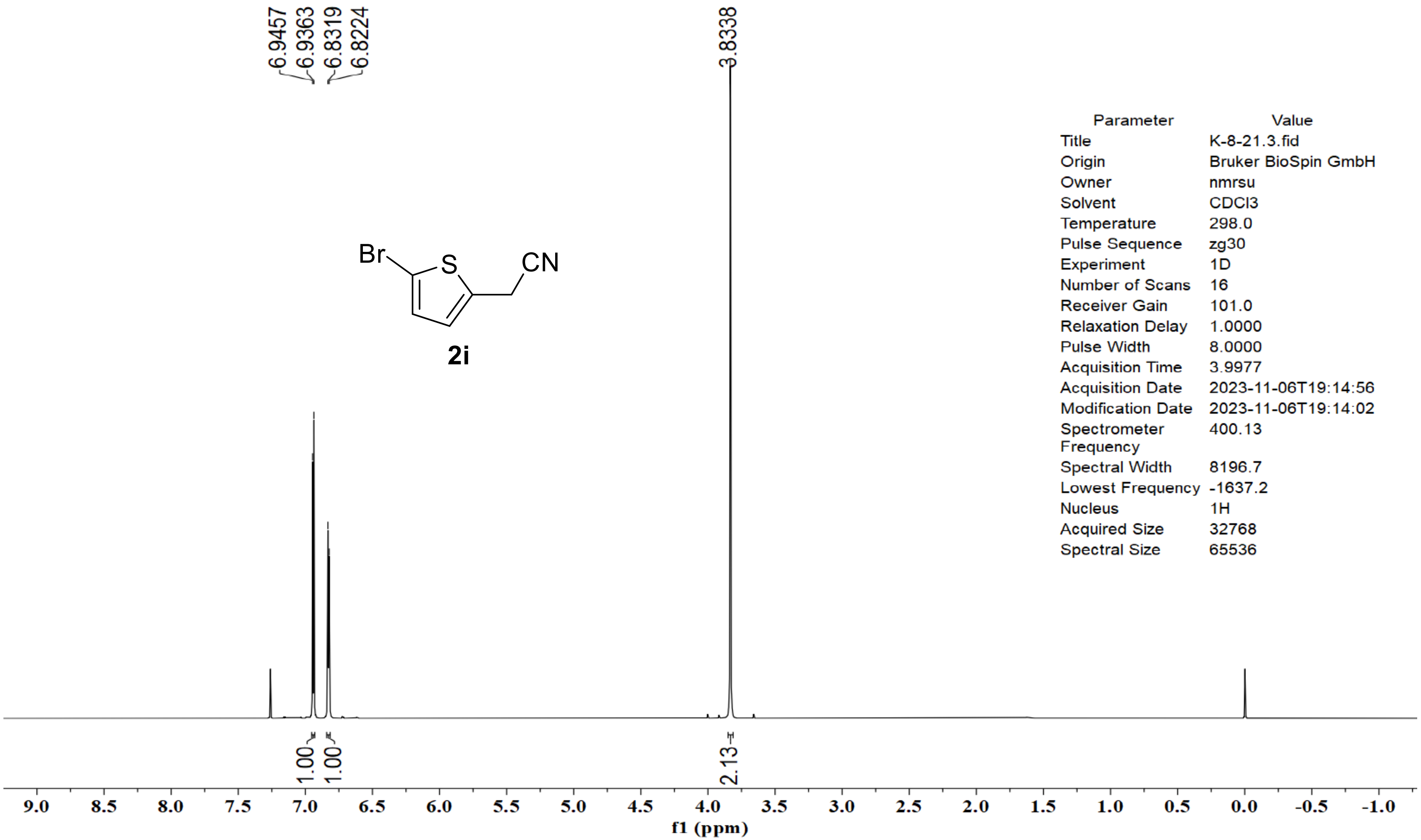

Parameter	Value
Title	K-8-21.3.fid
Origin	Bruker BioSpin GmbH
Owner	nmrsu
Solvent	CDCl3
Temperature	298.0
Pulse Sequence	zg30
Experiment	1D
Number of Scans	16
Receiver Gain	101.0
Relaxation Delay	1.0000
Pulse Width	8.0000
Acquisition Time	3.9977
Acquisition Date	2023-11-06T19:14:56
Modification Date	2023-11-06T19:14:02
Spectrometer Frequency	400.13
Spectral Width	8196.7
Lowest Frequency	-1637.2
Nucleus	1H
Acquired Size	32768
Spectral Size	65536

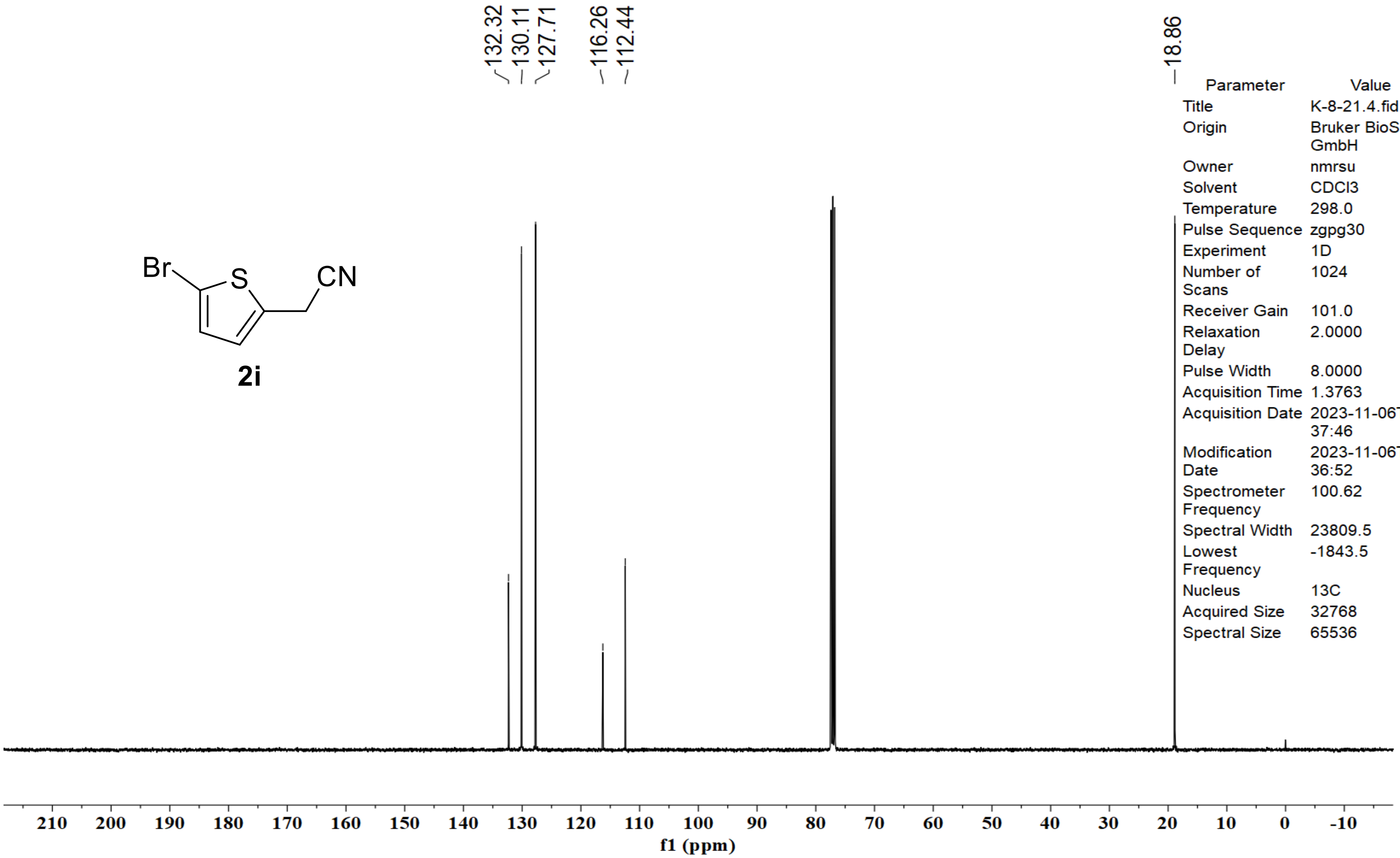

Parameter	Value
Title	K-8-21.4.fid
Origin	Bruker BioSpin GmbH
Owner	nmrsu
Solvent	CDCl3
Temperature	298.0
Pulse Sequence	zgpg30
Experiment	1D
Number of Scans	1024
Receiver Gain	101.0
Relaxation Delay	2.0000
Pulse Width	8.0000
Acquisition Time	1.3763
Acquisition Date	2023-11-06T20:37:46
Modification Date	2023-11-06T20:36:52
Spectrometer Frequency	100.62
Spectral Width	23809.5
Lowest Frequency	-1843.5
Nucleus	13C
Acquired Size	32768
Spectral Size	65536

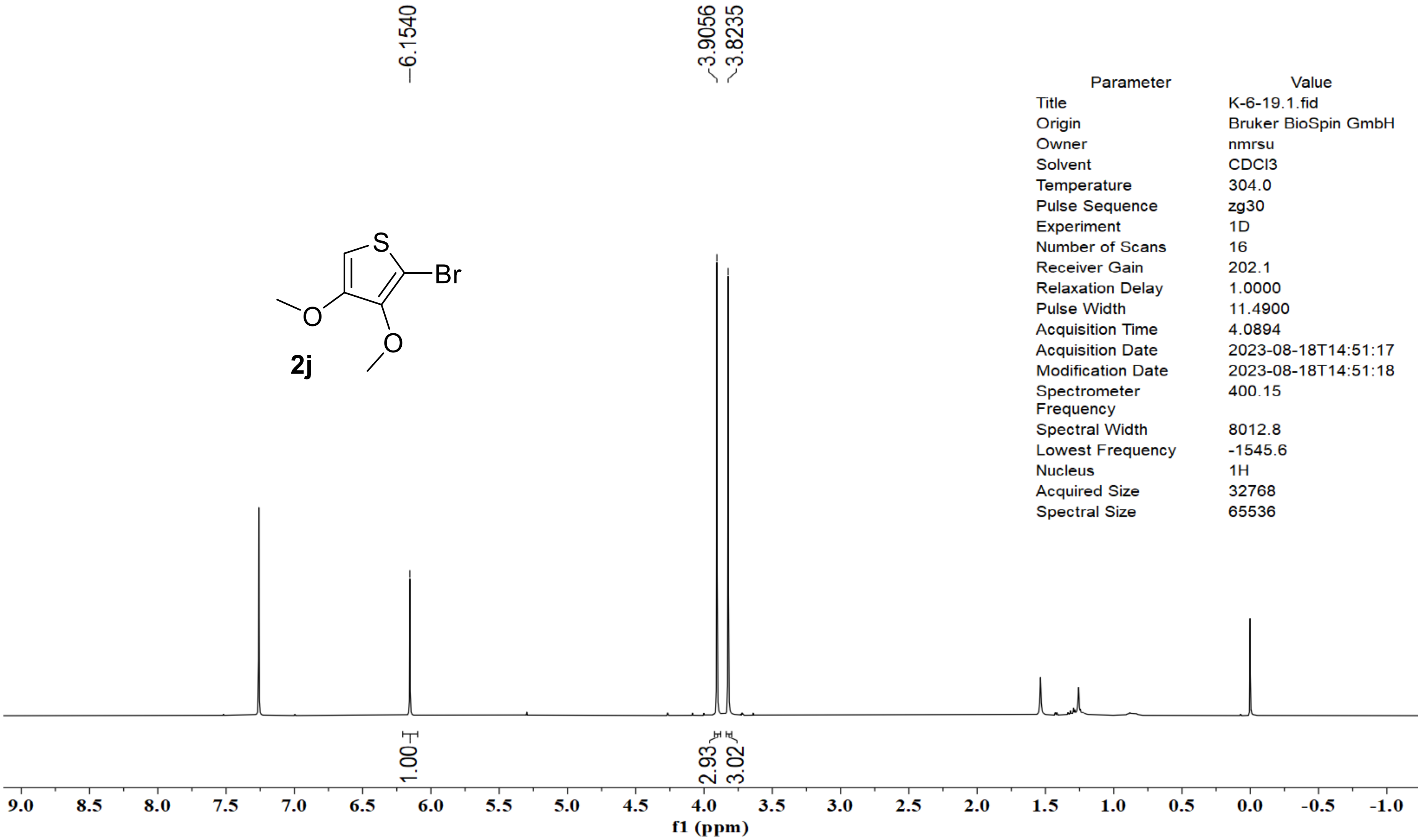

2j

Parameter	Value
Title	K-6-19.1.fid
Origin	Bruker BioSpin GmbH
Owner	nmrsu
Solvent	CDCl3
Temperature	304.0
Pulse Sequence	zg30
Experiment	1D
Number of Scans	16
Receiver Gain	202.1
Relaxation Delay	1.0000
Pulse Width	11.4900
Acquisition Time	4.0894
Acquisition Date	2023-08-18T14:51:17
Modification Date	2023-08-18T14:51:18
Spectrometer Frequency	400.15
Spectral Width	8012.8
Lowest Frequency	-1545.6
Nucleus	1H
Acquired Size	32768
Spectral Size	65536

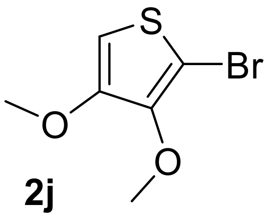

2j

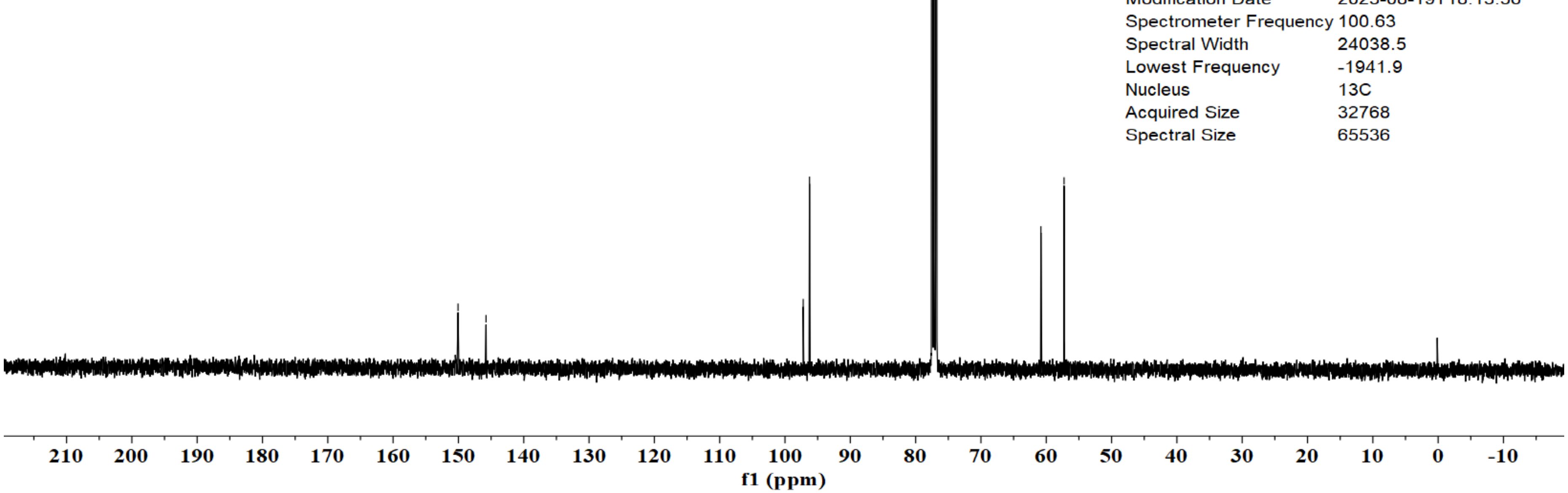

Parameter	Value
Title	K-6-19.2.fid
Origin	Bruker BioSpin GmbH
Owner	nmrsu
Solvent	CDCl3
Temperature	304.3
Pulse Sequence	zgpg30
Experiment	1D
Number of Scans	1024
Receiver Gain	202.1
Relaxation Delay	2.0000
Pulse Width	7.0000
Acquisition Time	1.3631
Acquisition Date	2023-08-19T18:13:55
Modification Date	2023-08-19T18:13:56
Spectrometer Frequency	100.63
Spectral Width	24038.5
Lowest Frequency	-1941.9
Nucleus	13C
Acquired Size	32768
Spectral Size	65536

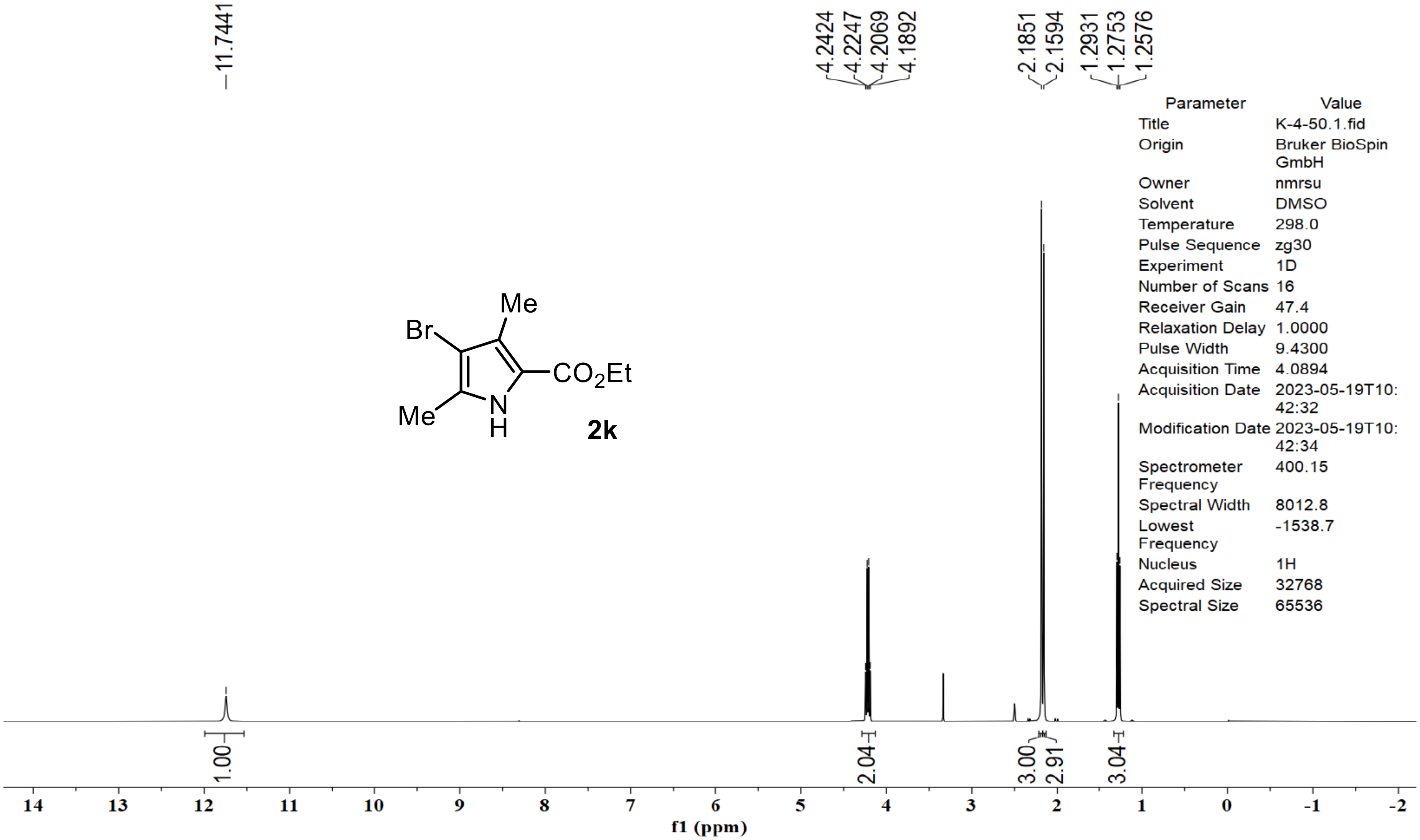

Parameter	Value
Title	K-4-50.1.fid
Origin	Bruker BioSpin GmbH
Owner	nmrsu
Solvent	DMSO
Temperature	298.0
Pulse Sequence	zg30
Experiment	1D
Number of Scans	16
Receiver Gain	47.4
Relaxation Delay	1.0000
Pulse Width	9.4300
Acquisition Time	4.0894
Acquisition Date	2023-05-19T10:42:32
Modification Date	2023-05-19T10:42:34
Spectrometer Frequency	400.15
Spectral Width	8012.8
Lowest Frequency	-1538.7
Nucleus	1H
Acquired Size	32768
Spectral Size	65536

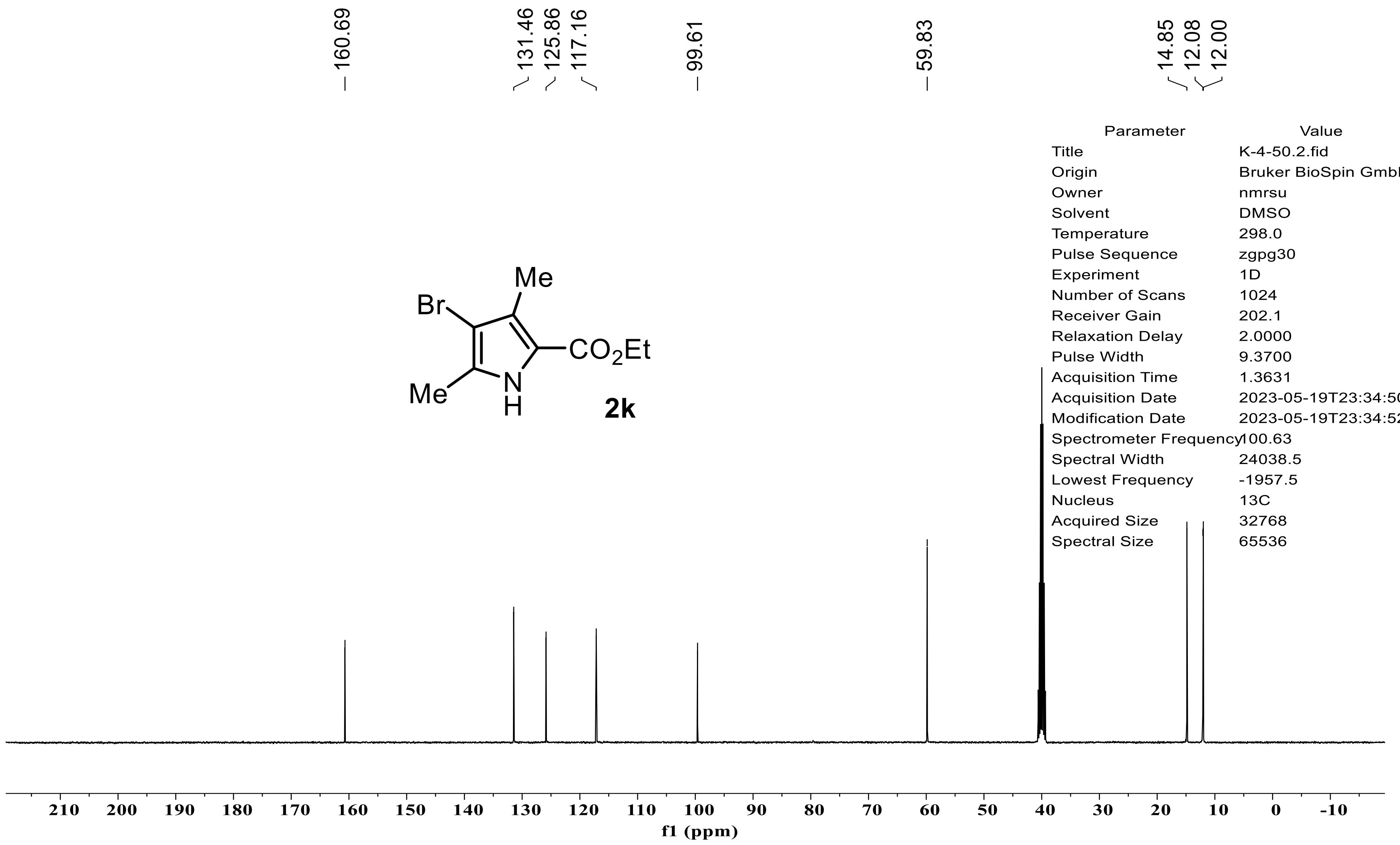

Parameter	Value
Title	K-4-50.2.fid
Origin	Bruker BioSpin GmbH
Owner	nmrsu
Solvent	DMSO
Temperature	298.0
Pulse Sequence	zgpg30
Experiment	1D
Number of Scans	1024
Receiver Gain	202.1
Relaxation Delay	2.0000
Pulse Width	9.3700
Acquisition Time	1.3631
Acquisition Date	2023-05-19T23:34:50
Modification Date	2023-05-19T23:34:52
Spectrometer Frequency	100.63
Spectral Width	24038.5
Lowest Frequency	-1957.5
Nucleus	13C
Acquired Size	32768
Spectral Size	65536

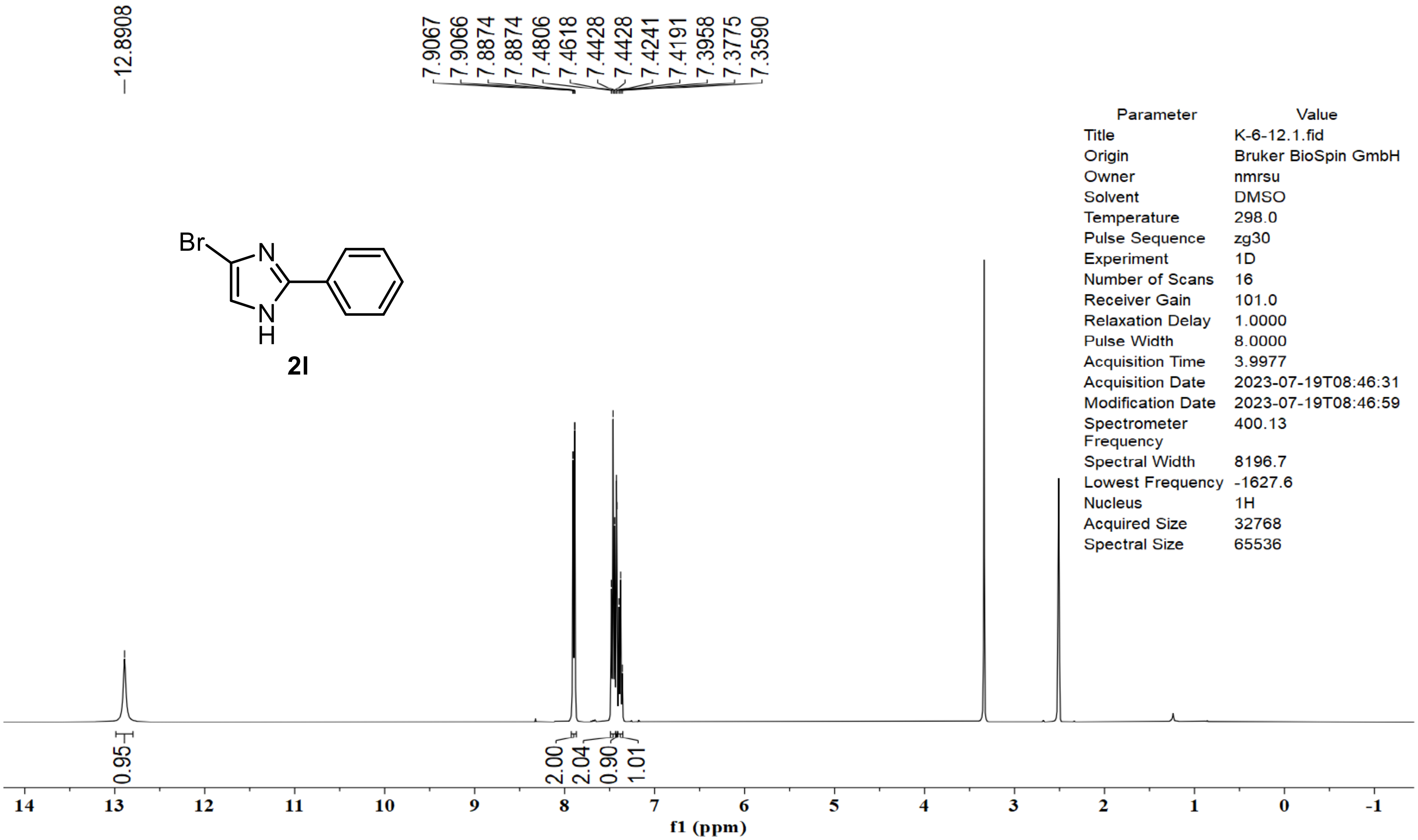

2l

Parameter	Value
Title	K-6-12.1.fid
Origin	Bruker BioSpin GmbH
Owner	nmrsu
Solvent	DMSO
Temperature	298.0
Pulse Sequence	zg30
Experiment	1D
Number of Scans	16
Receiver Gain	101.0
Relaxation Delay	1.0000
Pulse Width	8.0000
Acquisition Time	3.9977
Acquisition Date	2023-07-19T08:46:31
Modification Date	2023-07-19T08:46:59
Spectrometer Frequency	400.13
Spectral Width	8196.7
Lowest Frequency	-1627.6
Nucleus	1H
Acquired Size	32768
Spectral Size	65536

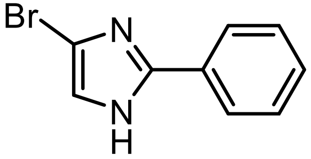

2l

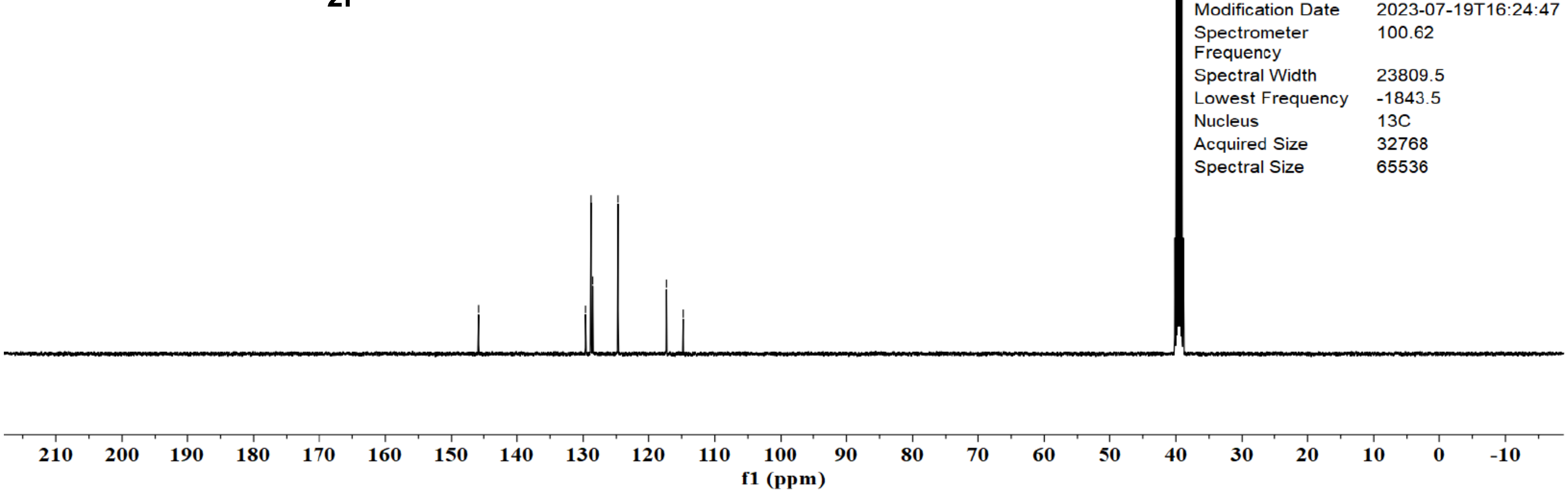

Parameter	Value
Title	K-6-12.6.fid
Origin	Bruker BioSpin GmbH
Owner	nmrsu
Solvent	DMSO
Temperature	298.0
Pulse Sequence	zgpg30
Experiment	1D
Number of Scans	1024
Receiver Gain	101.0
Relaxation Delay	2.0000
Pulse Width	8.0000
Acquisition Time	1.3763
Acquisition Date	2023-07-19T16:24:18
Modification Date	2023-07-19T16:24:47
Spectrometer Frequency	100.62
Spectral Width	23809.5
Lowest Frequency	-1843.5
Nucleus	13C
Acquired Size	32768
Spectral Size	65536

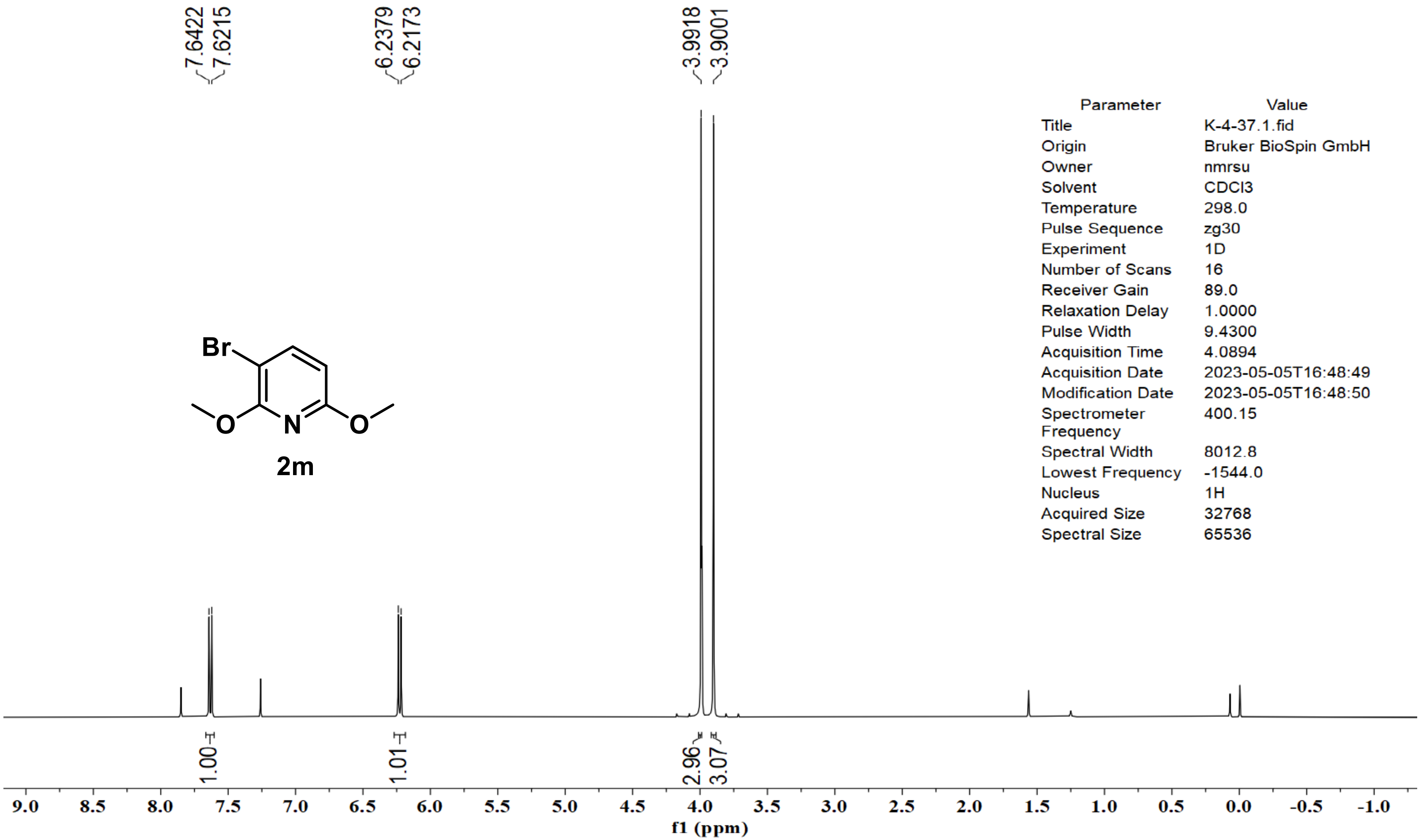

Parameter	Value
Title	K-4-37.1.fid
Origin	Bruker BioSpin GmbH
Owner	nmrsu
Solvent	CDCl3
Temperature	298.0
Pulse Sequence	zg30
Experiment	1D
Number of Scans	16
Receiver Gain	89.0
Relaxation Delay	1.0000
Pulse Width	9.4300
Acquisition Time	4.0894
Acquisition Date	2023-05-05T16:48:49
Modification Date	2023-05-05T16:48:50
Spectrometer Frequency	400.15
Spectral Width	8012.8
Lowest Frequency	-1544.0
Nucleus	1H
Acquired Size	32768
Spectral Size	65536

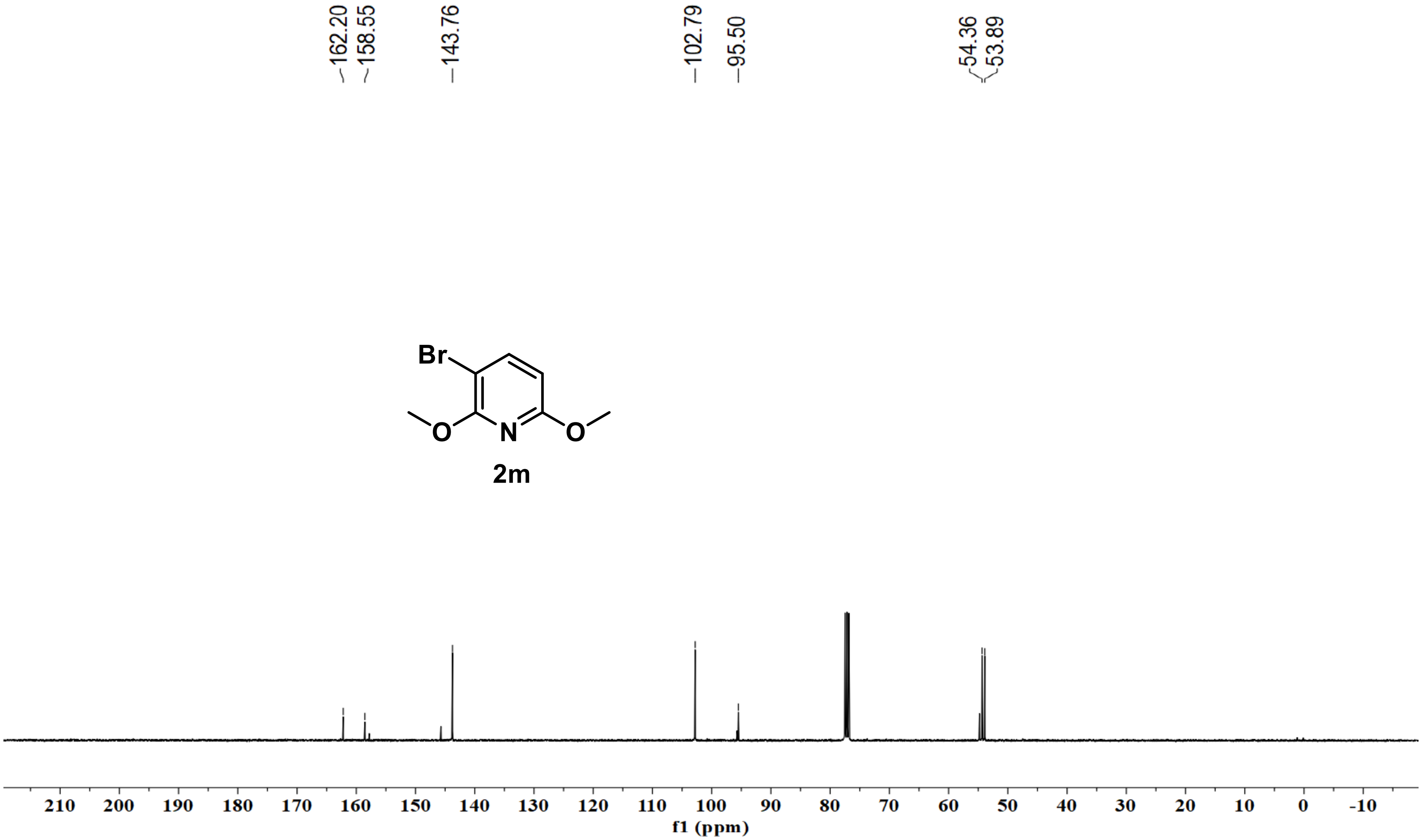
162.20
158.55
143.76
102.79
95.50
54.36
53.89
Br
O
N
O
2m
210 200 190 180 170 160 150 140 130 120 110 100 90 80 70 60 50 40 30 20 10 0 -10
f1 (ppm)

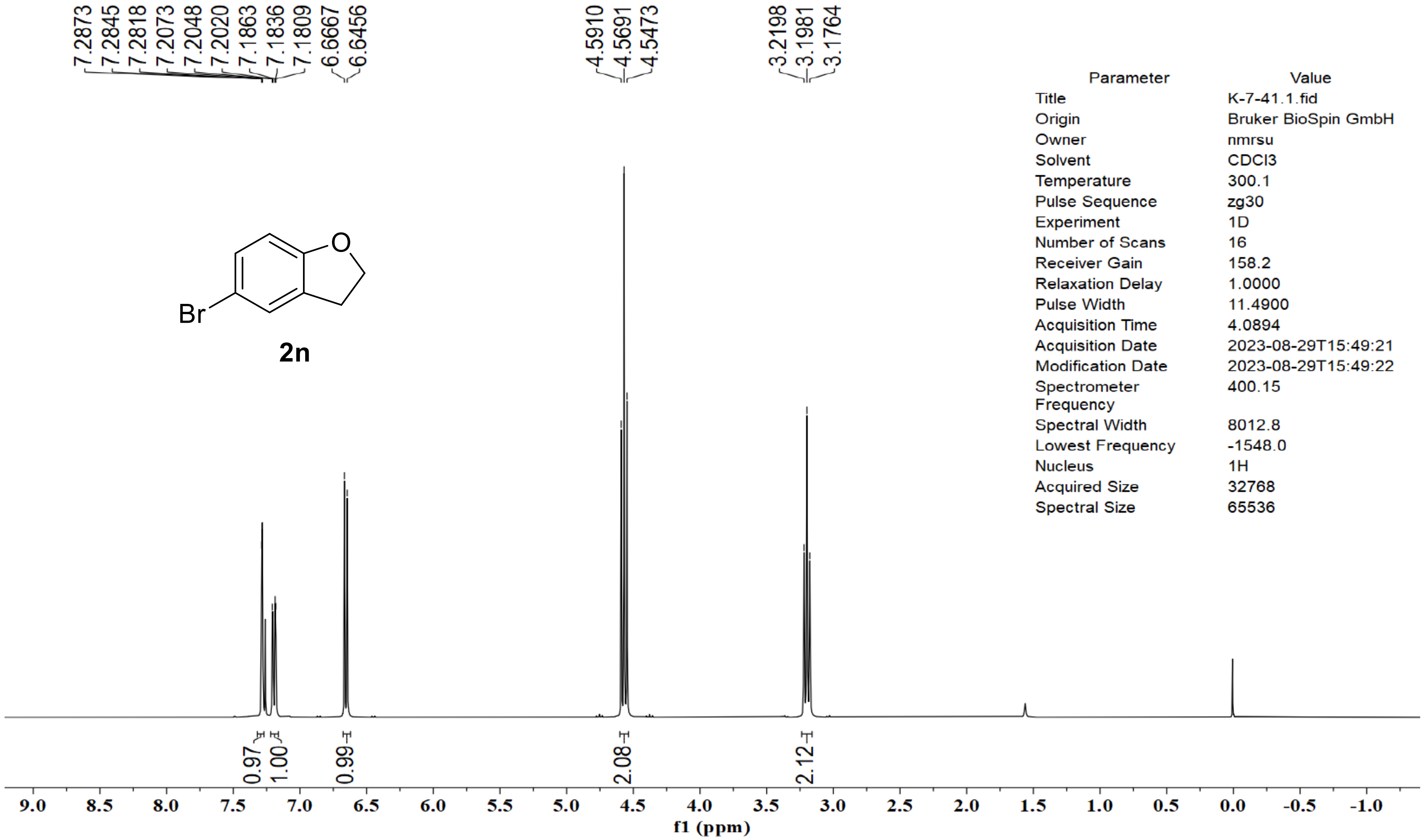

2n

Parameter	Value
Title	K-7-41.1.fid
Origin	Bruker BioSpin GmbH
Owner	nmrsu
Solvent	CDCl3
Temperature	300.1
Pulse Sequence	zg30
Experiment	1D
Number of Scans	16
Receiver Gain	158.2
Relaxation Delay	1.0000
Pulse Width	11.4900
Acquisition Time	4.0894
Acquisition Date	2023-08-29T15:49:21
Modification Date	2023-08-29T15:49:22
Spectrometer Frequency	400.15
Spectral Width	8012.8
Lowest Frequency	-1548.0
Nucleus	1H
Acquired Size	32768
Spectral Size	65536

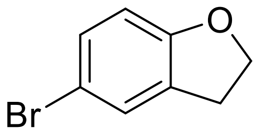

2n

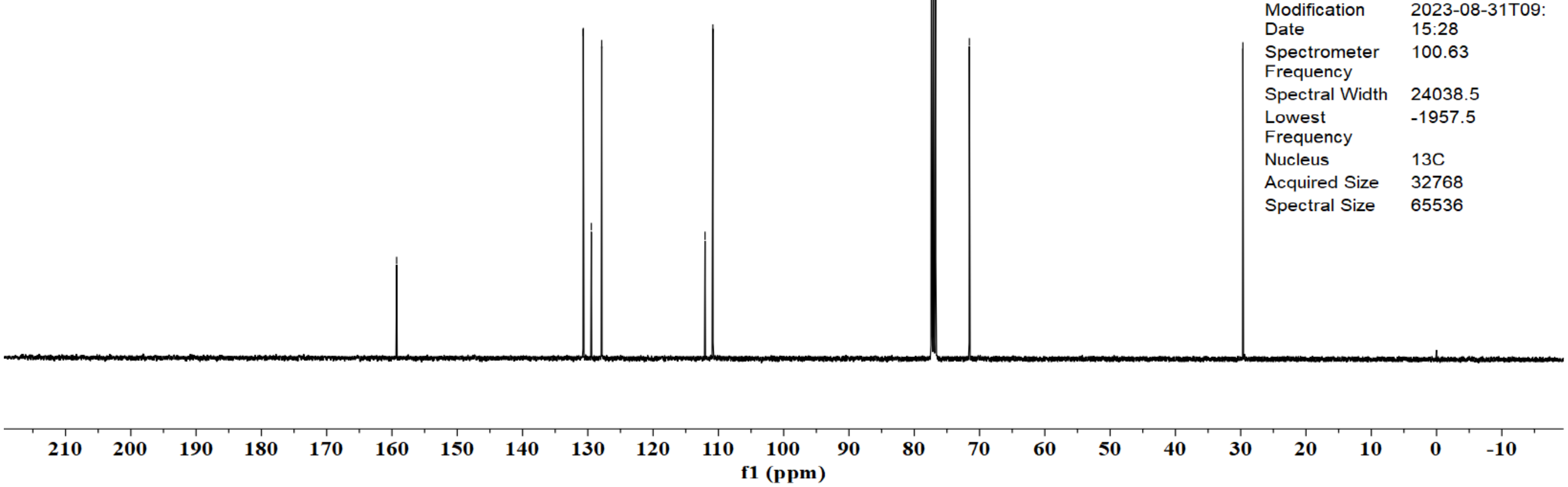

Parameter	Value
Title	K-7-41.2.fid
Origin	Bruker BioSpin GmbH
Owner	nmrsu
Solvent	CDCl3
Temperature	300.0
Pulse Sequence	zgpg30
Experiment	1D
Number of Scans	550
Receiver Gain	202.1
Relaxation Delay	2.0000
Pulse Width	7.0000
Acquisition Time	1.3631
Acquisition Date	2023-08-31T09:15:27
Modification Date	2023-08-31T09:15:28
Spectrometer Frequency	100.63
Spectral Width	24038.5
Lowest Frequency	-1957.5
Nucleus	13C
Acquired Size	32768
Spectral Size	65536

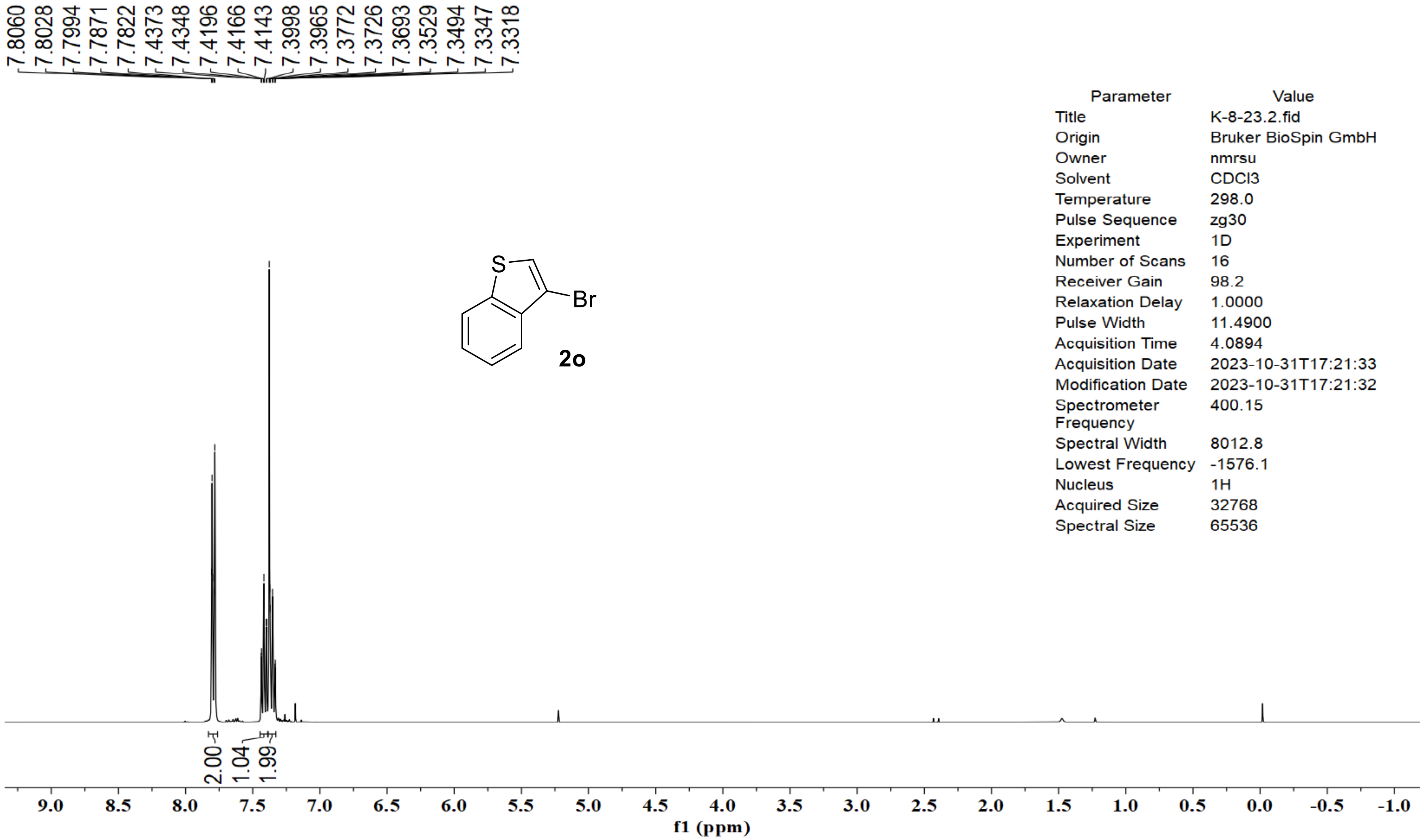

Parameter	Value
Title	K-8-23.2.fid
Origin	Bruker BioSpin GmbH
Owner	nmrsu
Solvent	CDCl3
Temperature	298.0
Pulse Sequence	zg30
Experiment	1D
Number of Scans	16
Receiver Gain	98.2
Relaxation Delay	1.0000
Pulse Width	11.4900
Acquisition Time	4.0894
Acquisition Date	2023-10-31T17:21:33
Modification Date	2023-10-31T17:21:32
Spectrometer Frequency	400.15
Spectral Width	8012.8
Lowest Frequency	-1576.1
Nucleus	1H
Acquired Size	32768
Spectral Size	65536

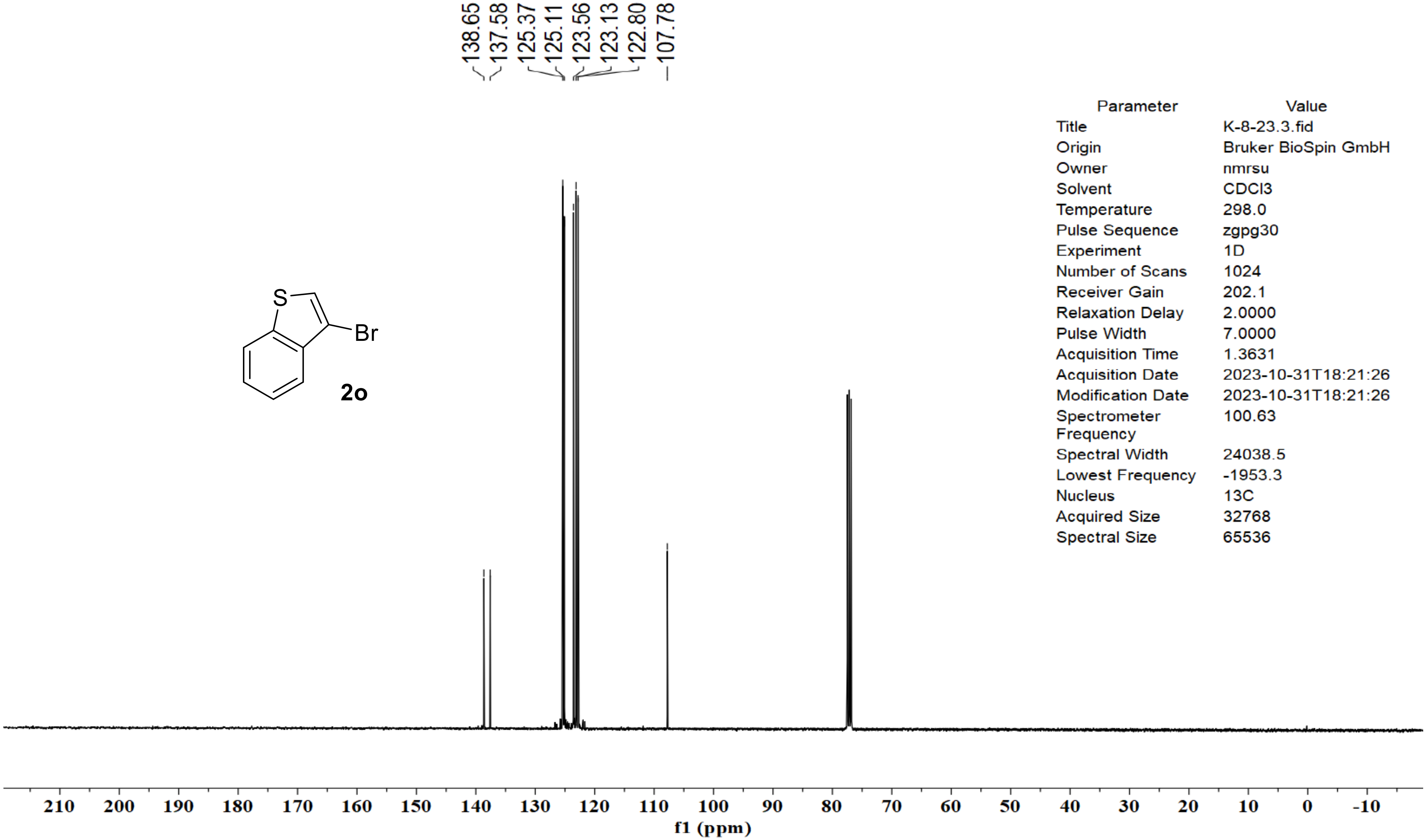

2o

Parameter	Value
Title	K-8-23.3.fid
Origin	Bruker BioSpin GmbH
Owner	nmrsu
Solvent	CDCl3
Temperature	298.0
Pulse Sequence	zgpg30
Experiment	1D
Number of Scans	1024
Receiver Gain	202.1
Relaxation Delay	2.0000
Pulse Width	7.0000
Acquisition Time	1.3631
Acquisition Date	2023-10-31T18:21:26
Modification Date	2023-10-31T18:21:26
Spectrometer Frequency	100.63
Spectral Width	24038.5
Lowest Frequency	-1953.3
Nucleus	13C
Acquired Size	32768
Spectral Size	65536

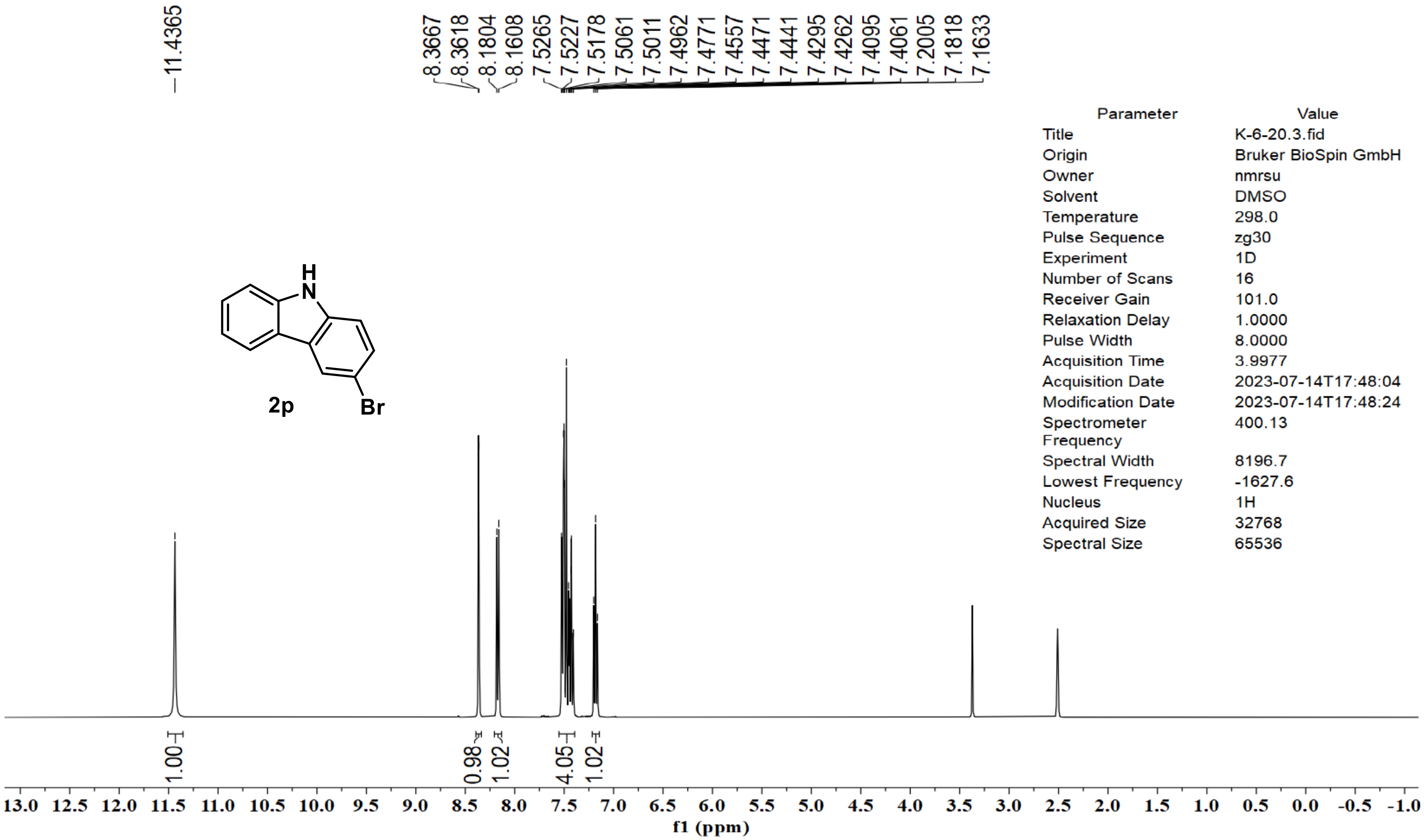

Parameter	Value
Title	K-6-20.3.fid
Origin	Bruker BioSpin GmbH
Owner	nmrsu
Solvent	DMSO
Temperature	298.0
Pulse Sequence	zg30
Experiment	1D
Number of Scans	16
Receiver Gain	101.0
Relaxation Delay	1.0000
Pulse Width	8.0000
Acquisition Time	3.9977
Acquisition Date	2023-07-14T17:48:04
Modification Date	2023-07-14T17:48:24
Spectrometer Frequency	400.13
Spectral Width	8196.7
Lowest Frequency	-1627.6
Nucleus	1H
Acquired Size	32768
Spectral Size	65536

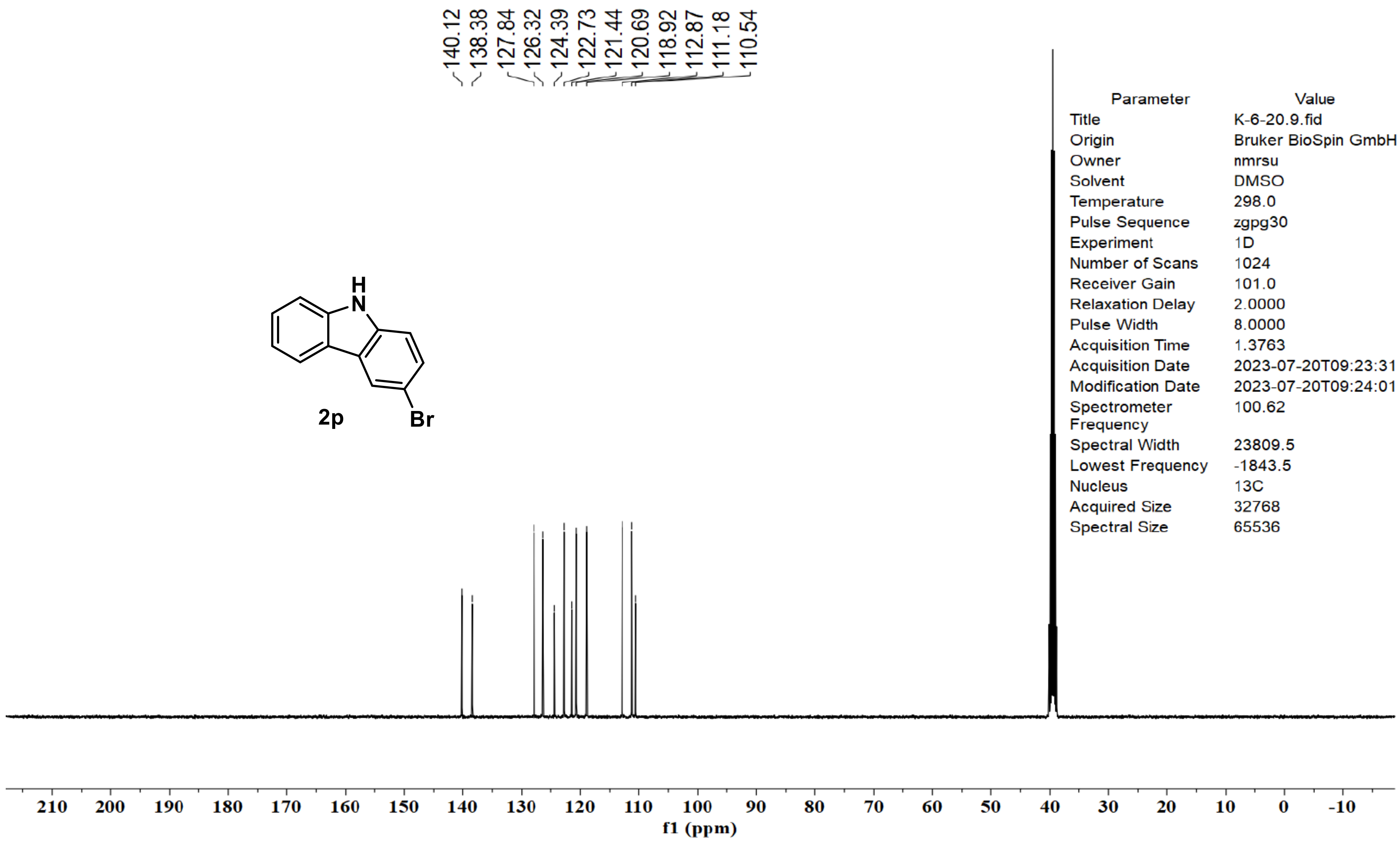

Parameter	Value
Title	K-6-20.9.fid
Origin	Bruker BioSpin GmbH
Owner	nmrsu
Solvent	DMSO
Temperature	298.0
Pulse Sequence	zgpg30
Experiment	1D
Number of Scans	1024
Receiver Gain	101.0
Relaxation Delay	2.0000
Pulse Width	8.0000
Acquisition Time	1.3763
Acquisition Date	2023-07-20T09:23:31
Modification Date	2023-07-20T09:24:01
Spectrometer Frequency	100.62
Spectral Width	23809.5
Lowest Frequency	-1843.5
Nucleus	13C
Acquired Size	32768
Spectral Size	65536

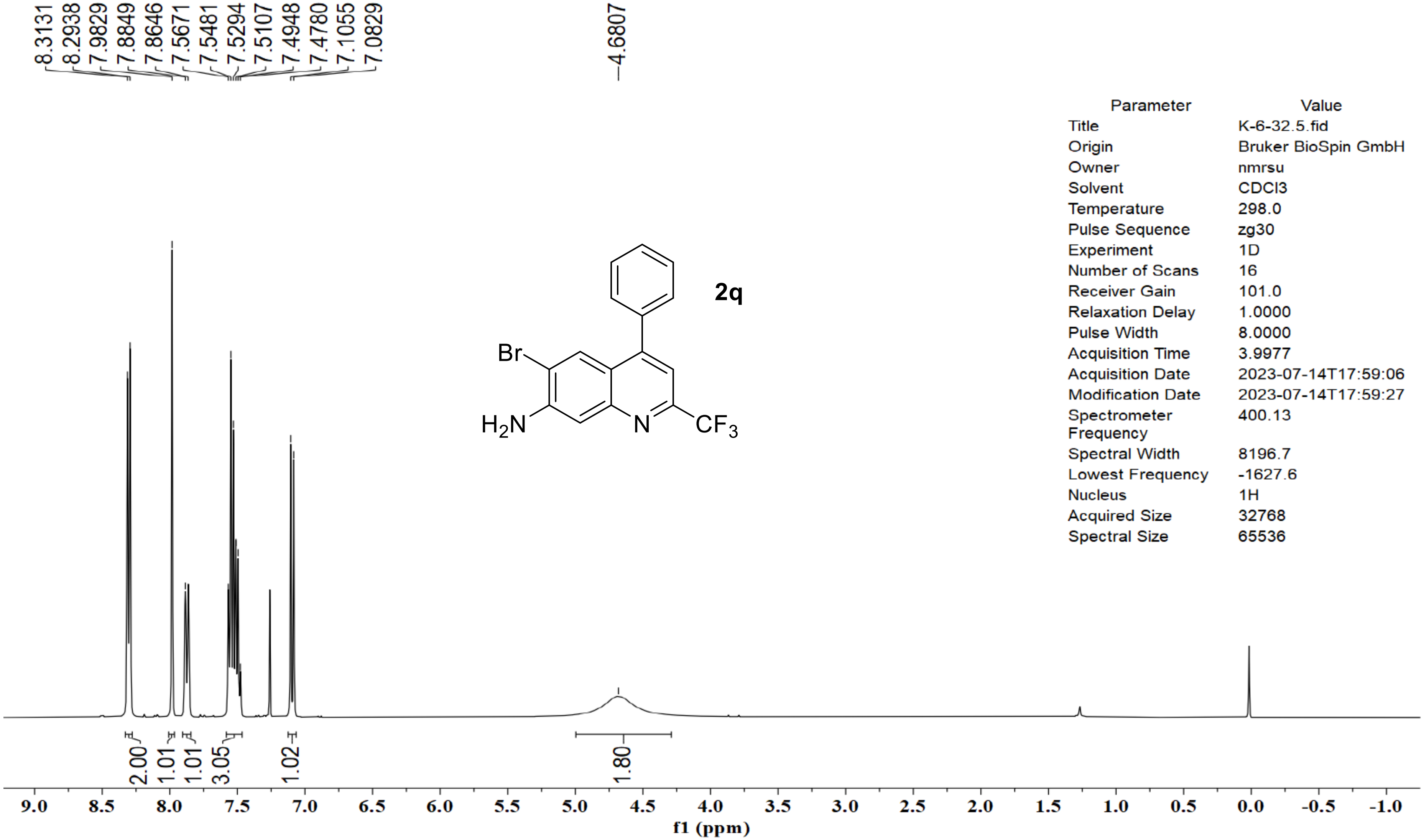

Parameter	Value
Title	K-6-32.5.fid
Origin	Bruker BioSpin GmbH
Owner	nmrsu
Solvent	CDCl3
Temperature	298.0
Pulse Sequence	zg30
Experiment	1D
Number of Scans	16
Receiver Gain	101.0
Relaxation Delay	1.0000
Pulse Width	8.0000
Acquisition Time	3.9977
Acquisition Date	2023-07-14T17:59:06
Modification Date	2023-07-14T17:59:27
Spectrometer Frequency	400.13
Spectral Width	8196.7
Lowest Frequency	-1627.6
Nucleus	1H
Acquired Size	32768
Spectral Size	65536

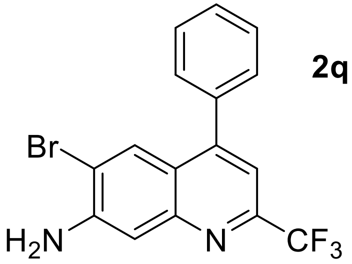

2q

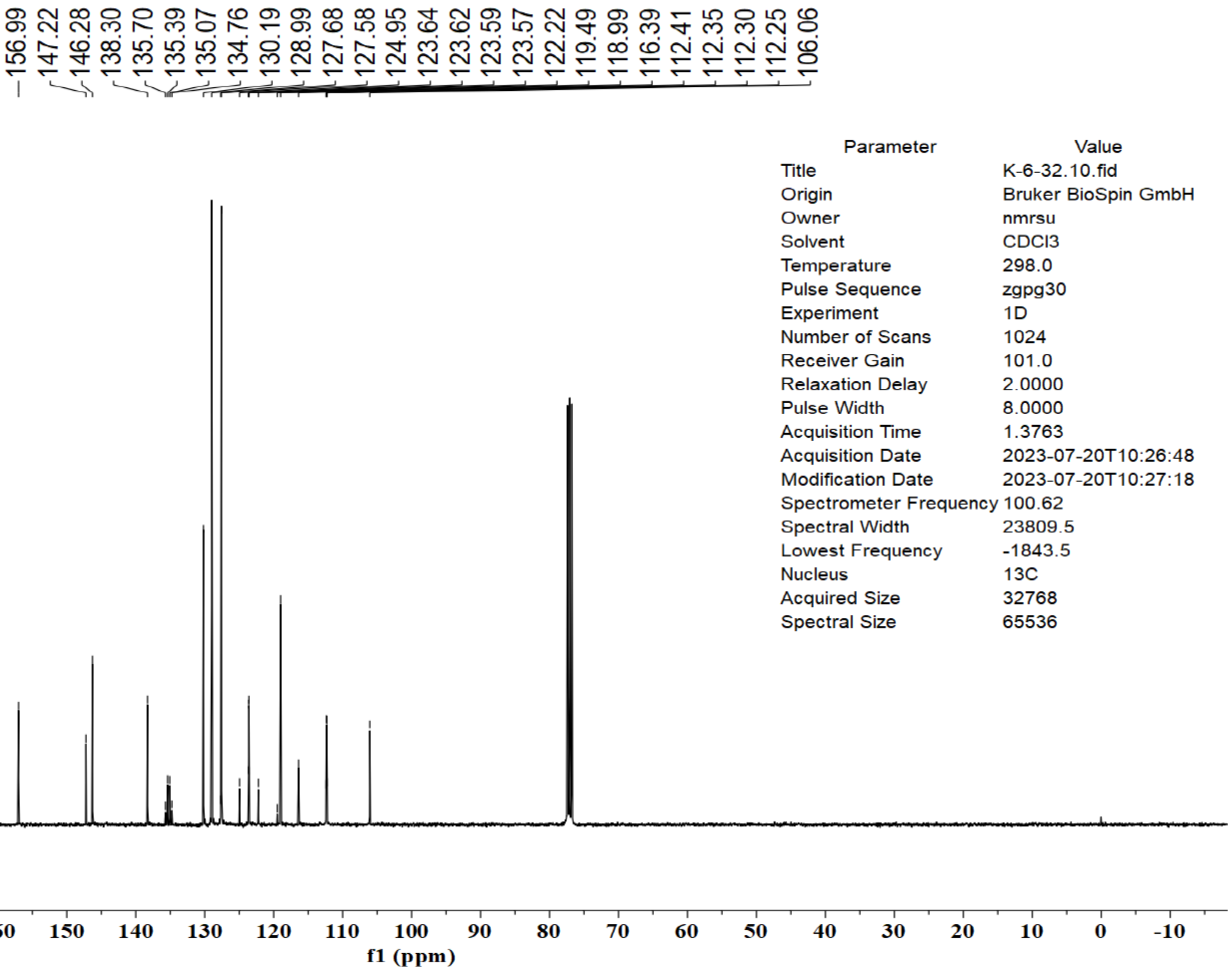

Parameter	Value
Title	K-6-32.10.fid
Origin	Bruker BioSpin GmbH
Owner	nmrsu
Solvent	CDCl3
Temperature	298.0
Pulse Sequence	zgpg30
Experiment	1D
Number of Scans	1024
Receiver Gain	101.0
Relaxation Delay	2.0000
Pulse Width	8.0000
Acquisition Time	1.3763
Acquisition Date	2023-07-20T10:26:48
Modification Date	2023-07-20T10:27:18
Spectrometer Frequency	100.62
Spectral Width	23809.5
Lowest Frequency	-1843.5
Nucleus	13C
Acquired Size	32768
Spectral Size	65536

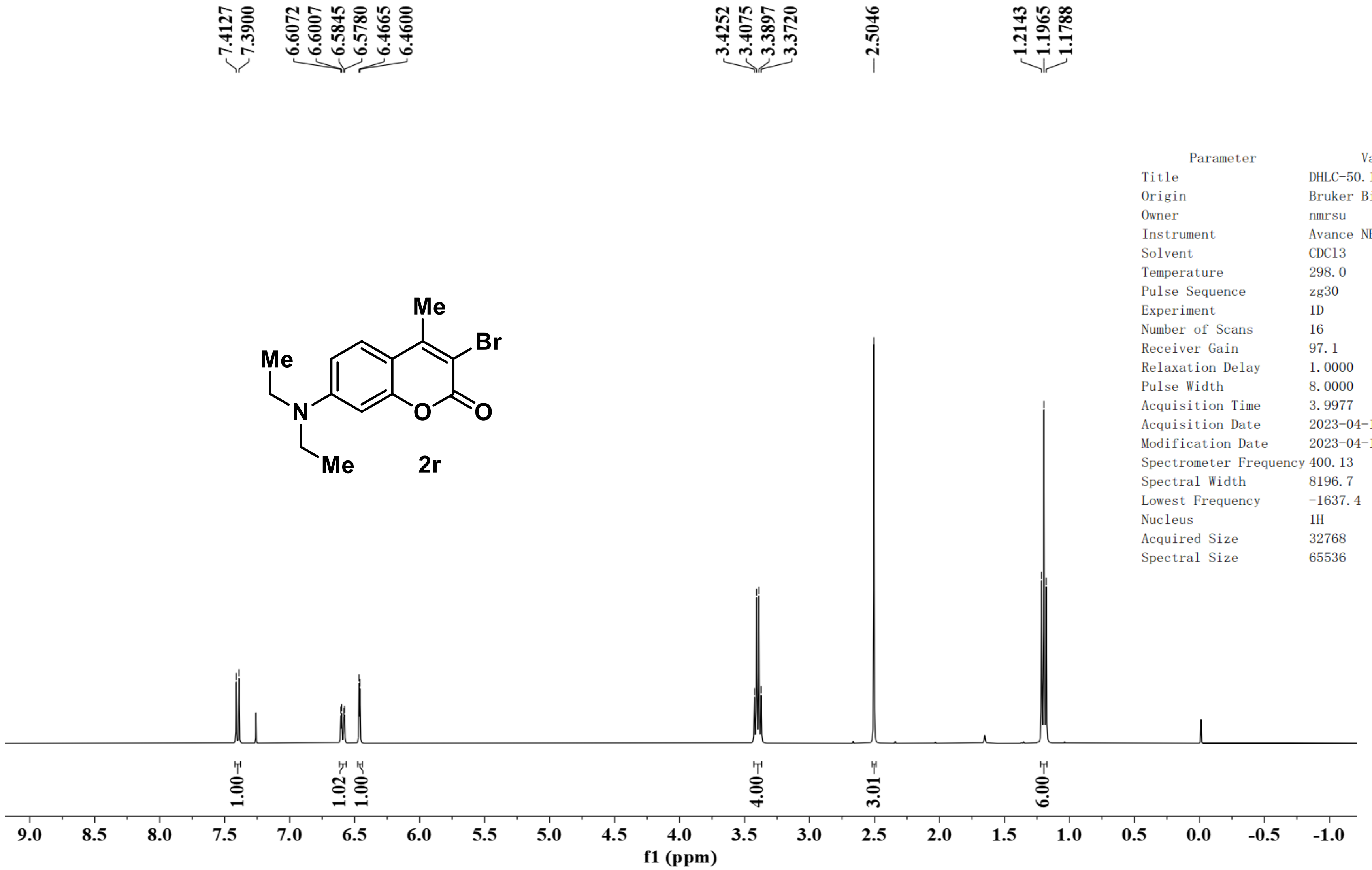

2r

Parameter	Value
Title	DHLC-50.1.fid
Origin	Bruker BioSpin GmbH
Owner	nmrsu
Instrument	Avance NEO
Solvent	CDCl3
Temperature	298.0
Pulse Sequence	zg30
Experiment	1D
Number of Scans	16
Receiver Gain	97.1
Relaxation Delay	1.0000
Pulse Width	8.0000
Acquisition Time	3.9977
Acquisition Date	2023-04-18T20:35:05
Modification Date	2023-04-18T20:35:11
Spectrometer Frequency	400.13
Spectral Width	8196.7
Lowest Frequency	-1637.4
Nucleus	1H
Acquired Size	32768
Spectral Size	65536

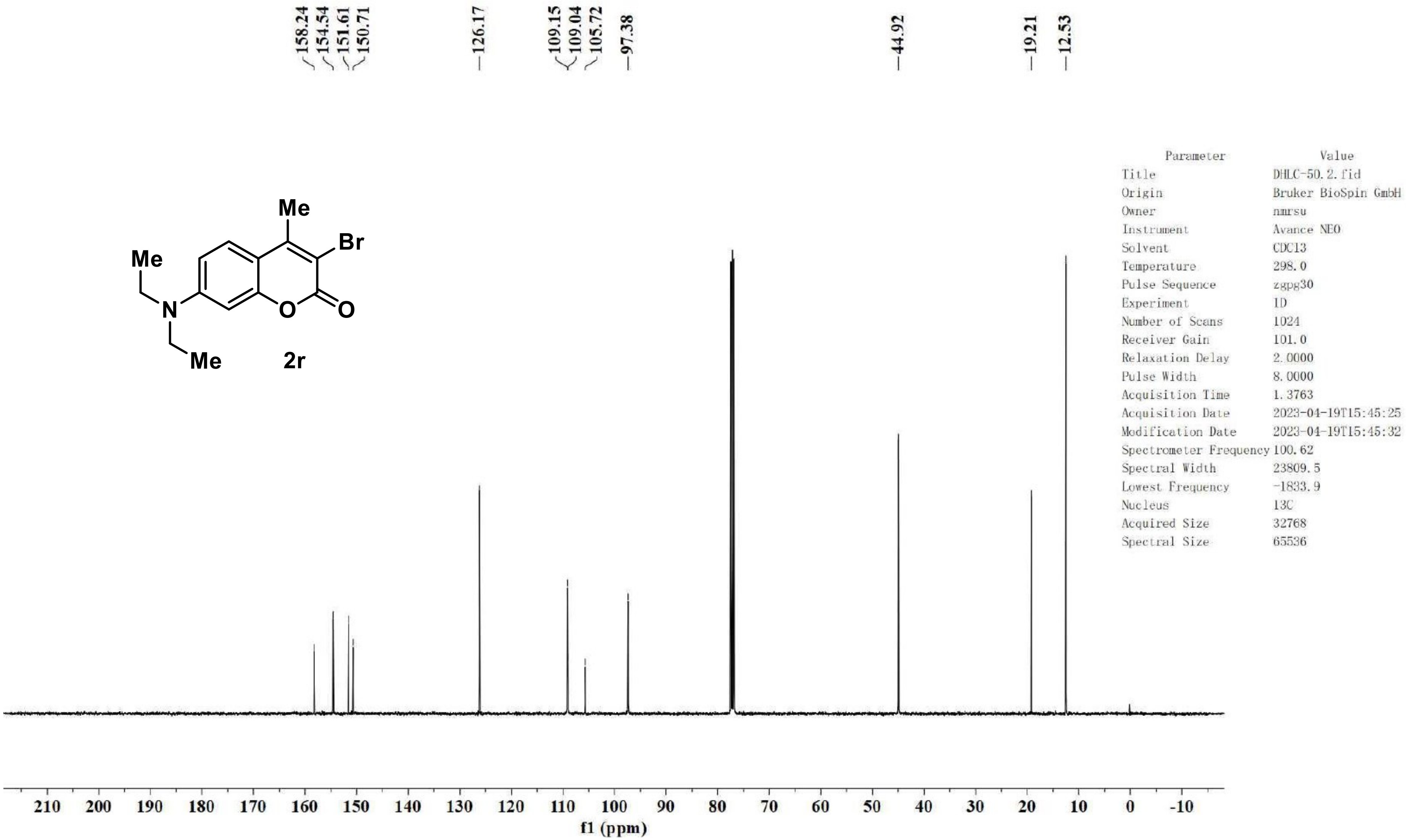
158.24
154.54
151.61
150.71
126.17
109.15
109.04
105.72
97.38
44.92
19.21
12.53
Me
Br
Me
N
O
O
Me
2r
Parameter Value
Title DHLC-50.2.fid
Origin Bruker BioSpin GmbH
Owner nmrsu
Instrument Avance NEO
Solvent CDCl3
Temperature 298.0
Pulse Sequence zgpg30
Experiment 1D
Number of Scans 1024
Receiver Gain 101.0
Relaxation Delay 2.0000
Pulse Width 8.0000
Acquisition Time 1.3763
Acquisition Date 2023-04-19T15:45:25
Modification Date 2023-04-19T15:45:32
Spectrometer Frequency 100.62
Spectral Width 23809.5
Lowest Frequency -1833.9
Nucleus 13C
Acquired Size 32768
Spectral Size 65536
210 200 190 180 170 160 150 140 130 120 110 100 90 80 70 60 50 40 30 20 10 0 -10
f1 (ppm)

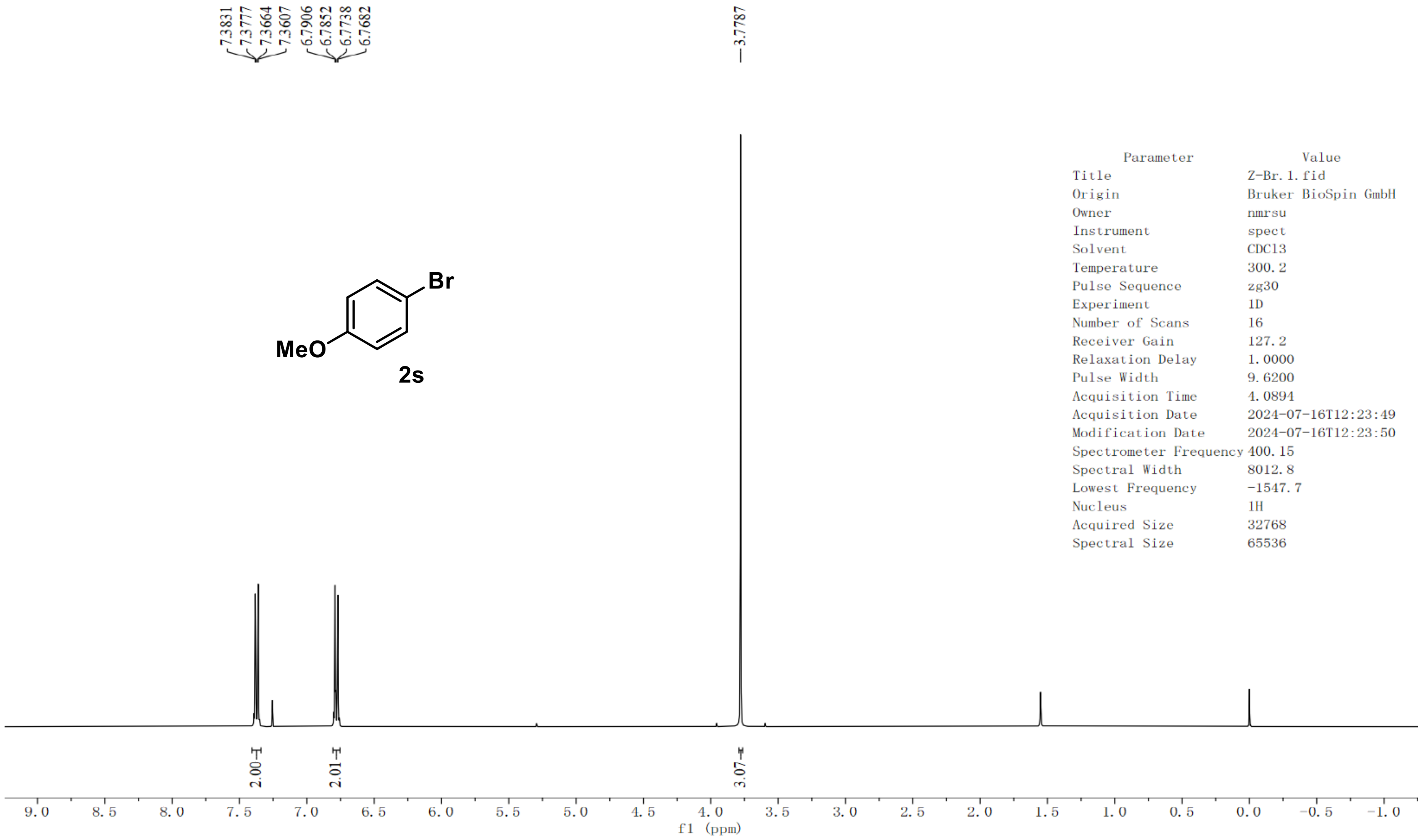
7.3831
7.3777
7.3664
7.3607
6.7906
6.7852
6.7738
6.7682
3.7787
Br
MeO
2s
Parameter Value
Title Z-Br.1.fid
Origin Bruker BioSpin GmbH
Owner nmrsu
Instrument spect
Solvent CDCl3
Temperature 300.2
Pulse Sequence zg30
Experiment 1D
Number of Scans 16
Receiver Gain 127.2
Relaxation Delay 1.0000
Pulse Width 9.6200
Acquisition Time 4.0894
Acquisition Date 2024-07-16T12:23:49
Modification Date 2024-07-16T12:23:50
Spectrometer Frequency 400.15
Spectral Width 8012.8
Lowest Frequency -1547.7
Nucleus 1H
Acquired Size 32768
Spectral Size 65536
2.00
2.01
3.07
9.0 8.5 8.0 7.5 7.0 6.5 6.0 5.5 5.0 4.5 4.0 3.5 3.0 2.5 2.0 1.5 1.0 0.5 0.0 -0.5 -1.0
f1 (ppm)

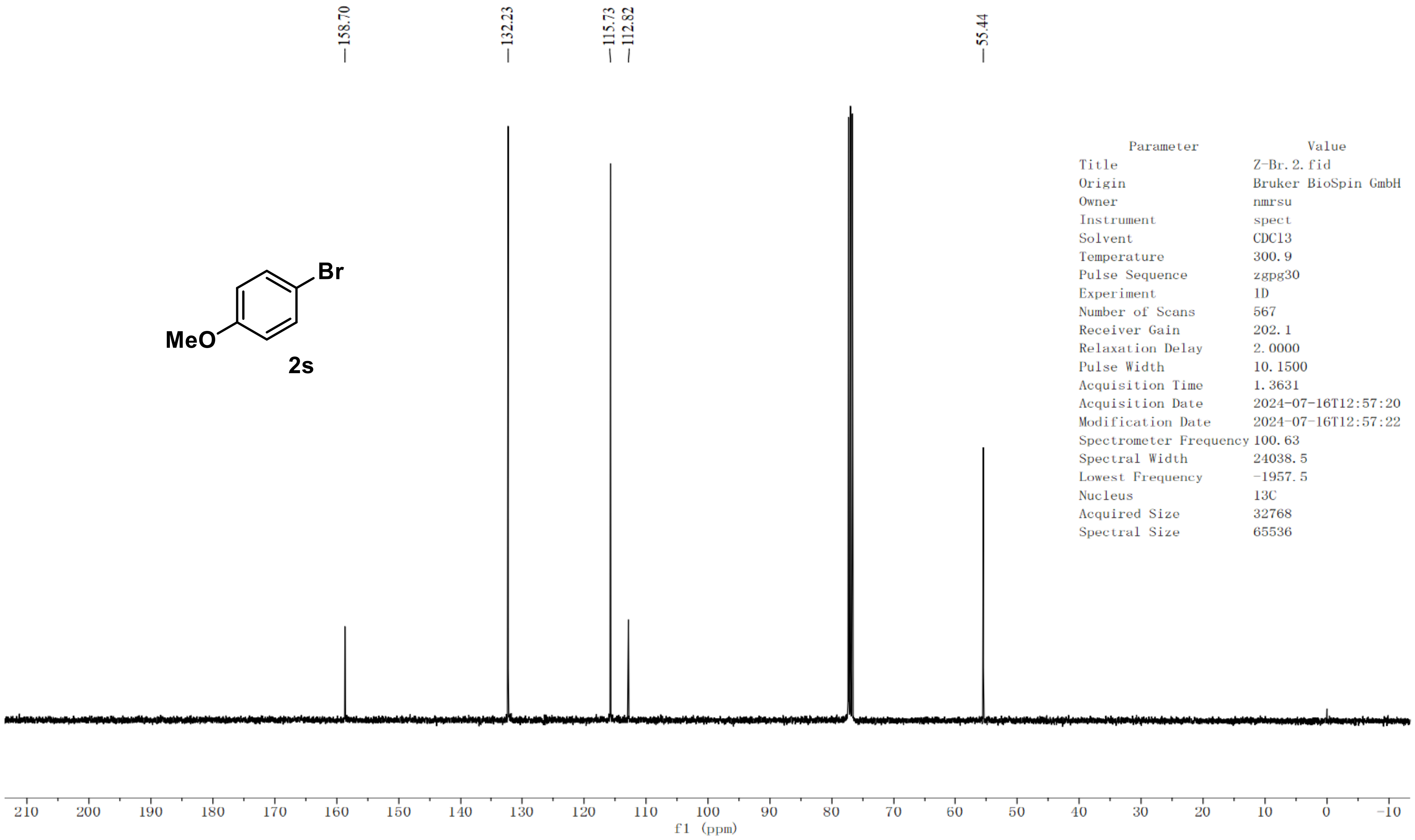

Parameter	Value
Title	Z-Br.2.fid
Origin	Bruker BioSpin GmbH
Owner	nmrsu
Instrument	spect
Solvent	CDCl3
Temperature	300.9
Pulse Sequence	zgpg30
Experiment	1D
Number of Scans	567
Receiver Gain	202.1
Relaxation Delay	2.0000
Pulse Width	10.1500
Acquisition Time	1.3631
Acquisition Date	2024-07-16T12:57:20
Modification Date	2024-07-16T12:57:22
Spectrometer Frequency	100.63
Spectral Width	24038.5
Lowest Frequency	-1957.5
Nucleus	13C
Acquired Size	32768
Spectral Size	65536

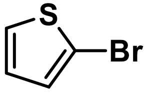

2t

Parameter	Value
Title	ZXH-14.1.fid
Origin	Bruker BioSpin GmbH
Owner	nmrsu
Instrument	spect
Solvent	CDCl3
Temperature	303.1
Pulse Sequence	zg30
Experiment	1D
Number of Scans	16
Receiver Gain	158.2
Relaxation Delay	1.0000
Pulse Width	9.6200
Acquisition Time	4.0894
Acquisition Date	2024-07-22T13:30:43
Modification Date	2024-07-22T13:30:44
Spectrometer Frequency	400.15
Spectral Width	8012.8
Lowest Frequency	-1545.3
Nucleus	1H
Acquired Size	32768
Spectral Size	65536

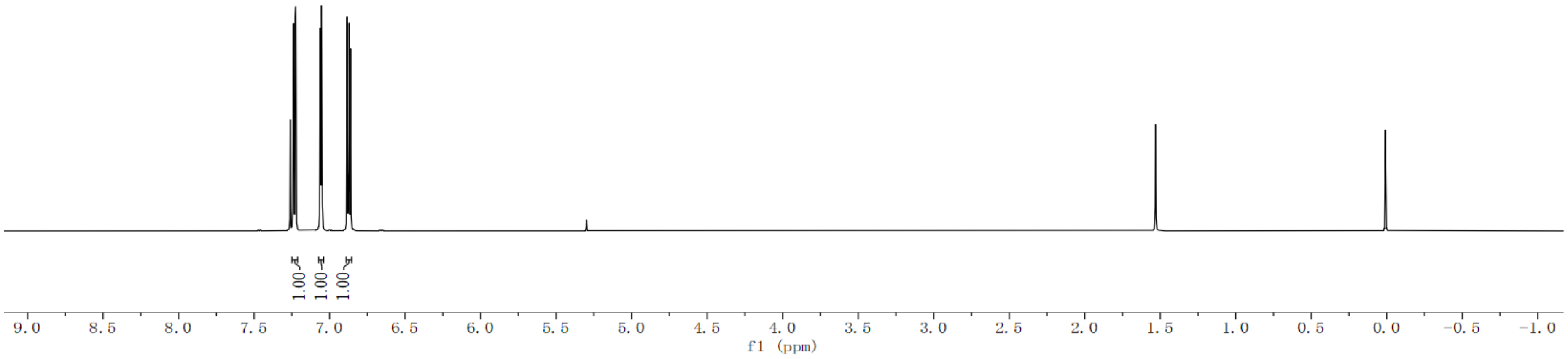

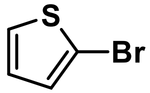

2t

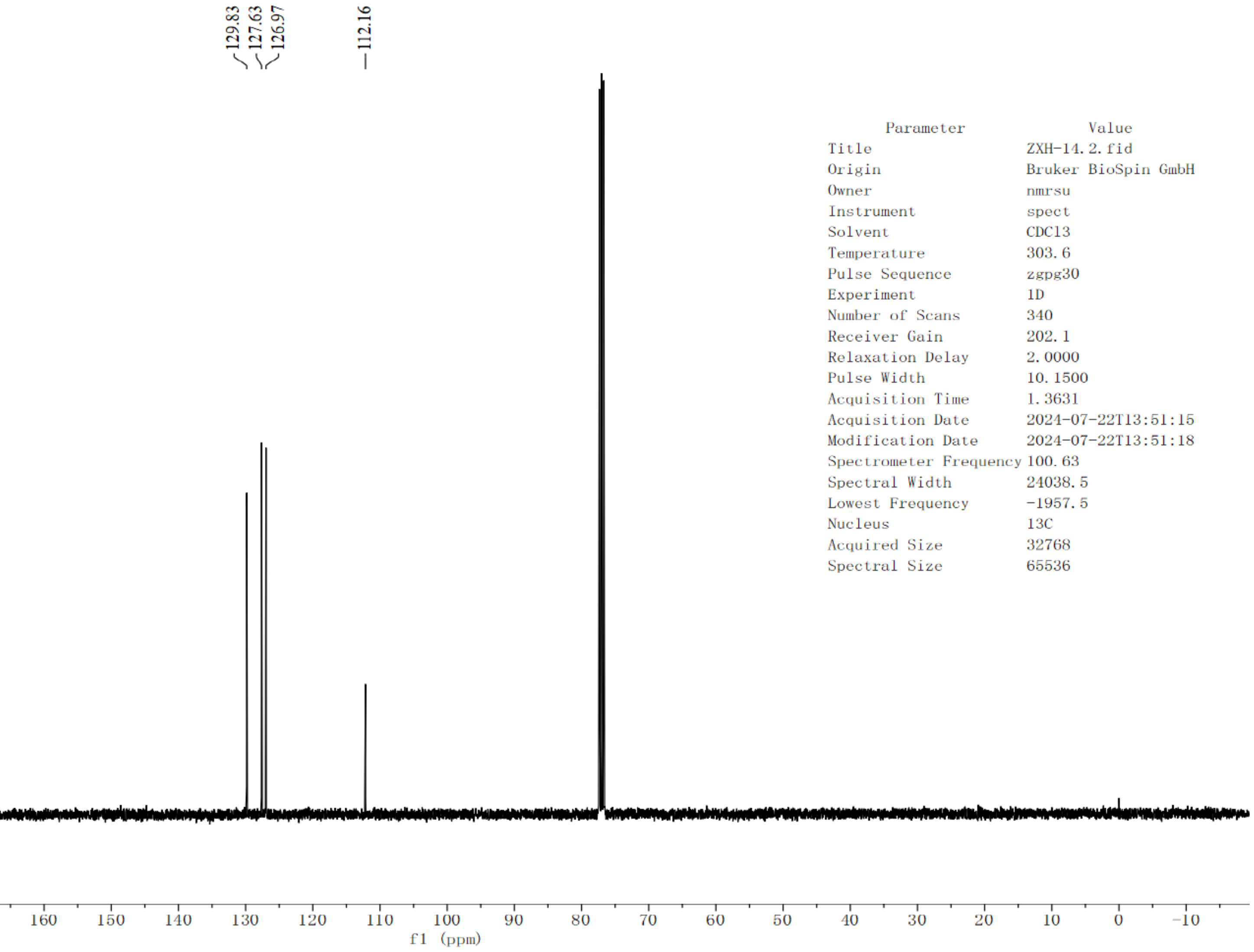

Parameter	Value
Title	ZXH-14.2.fid
Origin	Bruker BioSpin GmbH
Owner	nmrsu
Instrument	spect
Solvent	CDCl3
Temperature	303.6
Pulse Sequence	zgpg30
Experiment	1D
Number of Scans	340
Receiver Gain	202.1
Relaxation Delay	2.0000
Pulse Width	10.1500
Acquisition Time	1.3631
Acquisition Date	2024-07-22T13:51:15
Modification Date	2024-07-22T13:51:18
Spectrometer Frequency	100.63
Spectral Width	24038.5
Lowest Frequency	-1957.5
Nucleus	13C
Acquired Size	32768
Spectral Size	65536

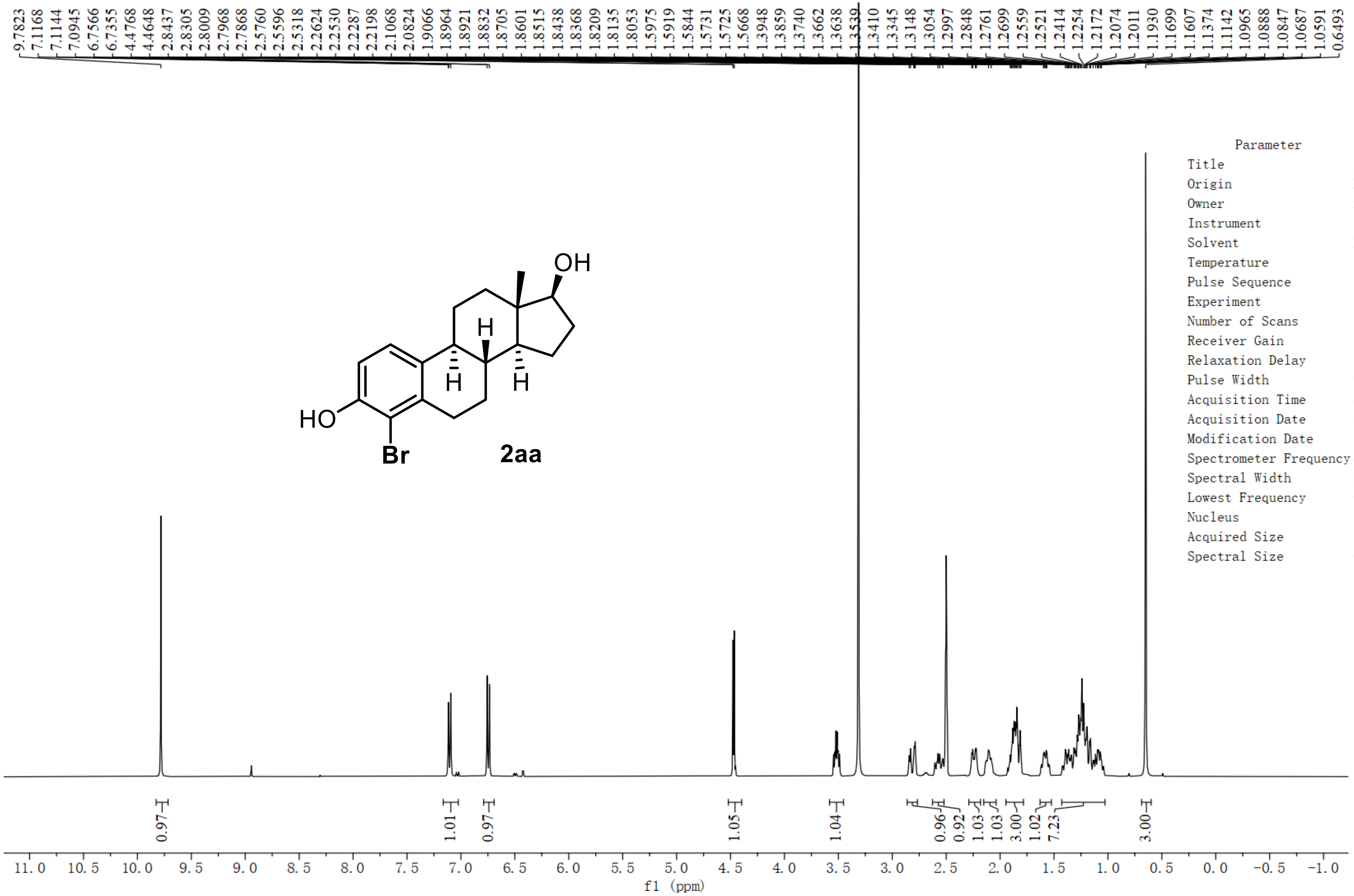

Parameter	Value
Title	ZXH-16.1.fid
Origin	Bruker BioSpin GmbH
Owner	nmrsu
Instrument	spect
Solvent	DMSO
Temperature	303.0
Pulse Sequence	zg30
Experiment	1D
Number of Scans	16
Receiver Gain	112.0
Relaxation Delay	1.0000
Pulse Width	9.6200
Acquisition Time	4.0894
Acquisition Date	2024-07-22T17:12:21
Modification Date	2024-07-22T17:12:22
Spectrometer Frequency	400.15
Spectral Width	8012.8
Lowest Frequency	-1538.3
Nucleus	1H
Acquired Size	32768
Spectral Size	65536

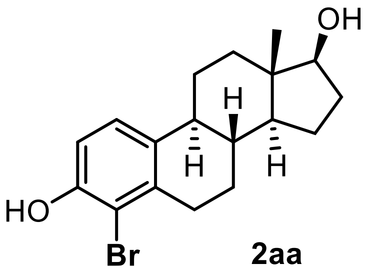

2aa

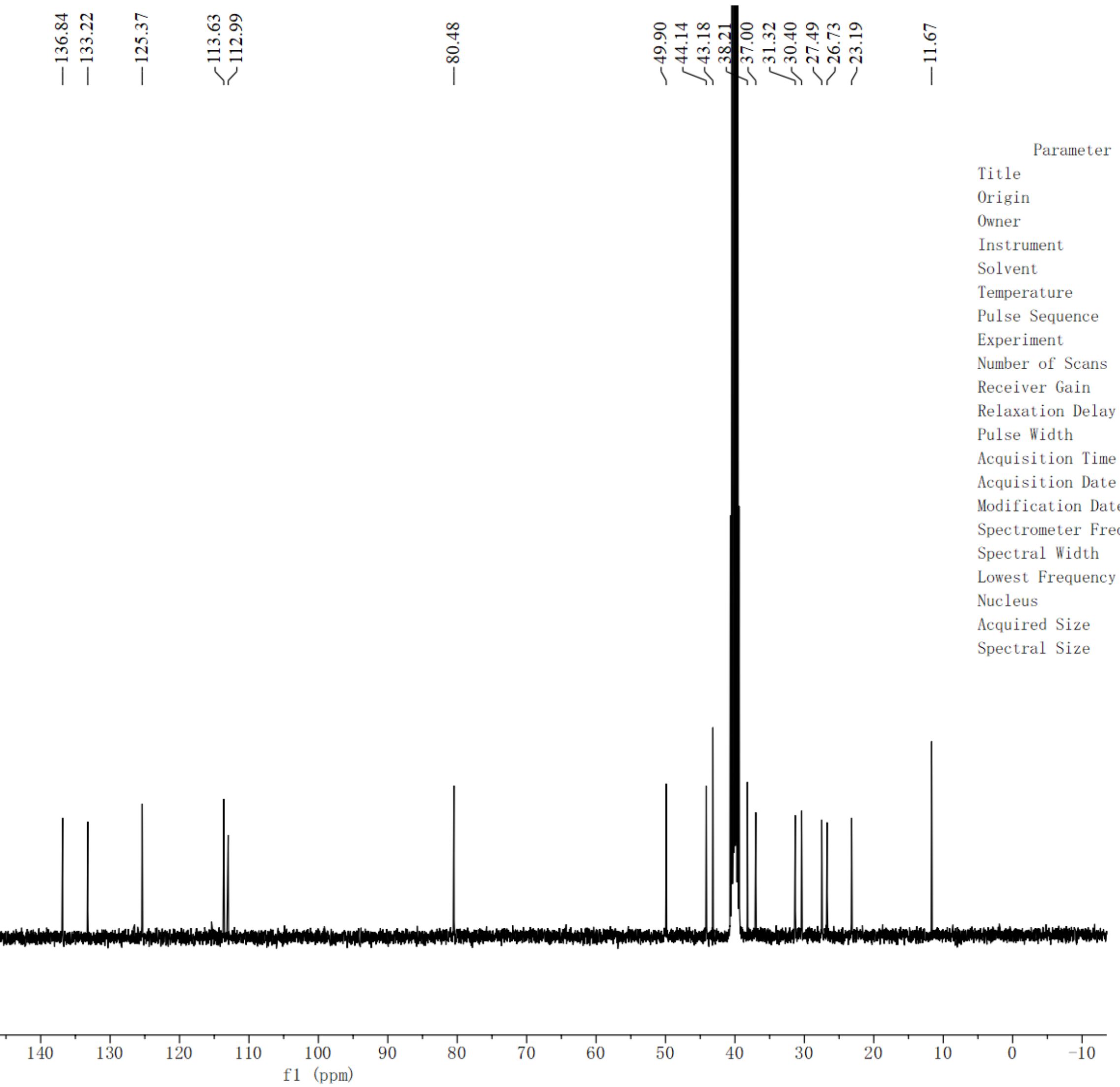

Parameter	Value
Title	ZXH-16.2.fid
Origin	Bruker BioSpin GmbH
Owner	nmrsu
Instrument	spect
Solvent	DMSO
Temperature	304.5
Pulse Sequence	zgpg30
Experiment	1D
Number of Scans	1024
Receiver Gain	202.1
Relaxation Delay	2.0000
Pulse Width	10.1500
Acquisition Time	1.3631
Acquisition Date	2024-07-23T16:14:58
Modification Date	2024-07-23T16:15:00
Spectrometer Frequency	100.63
Spectral Width	24038.5
Lowest Frequency	-1957.5
Nucleus	13C
Acquired Size	32768
Spectral Size	65536

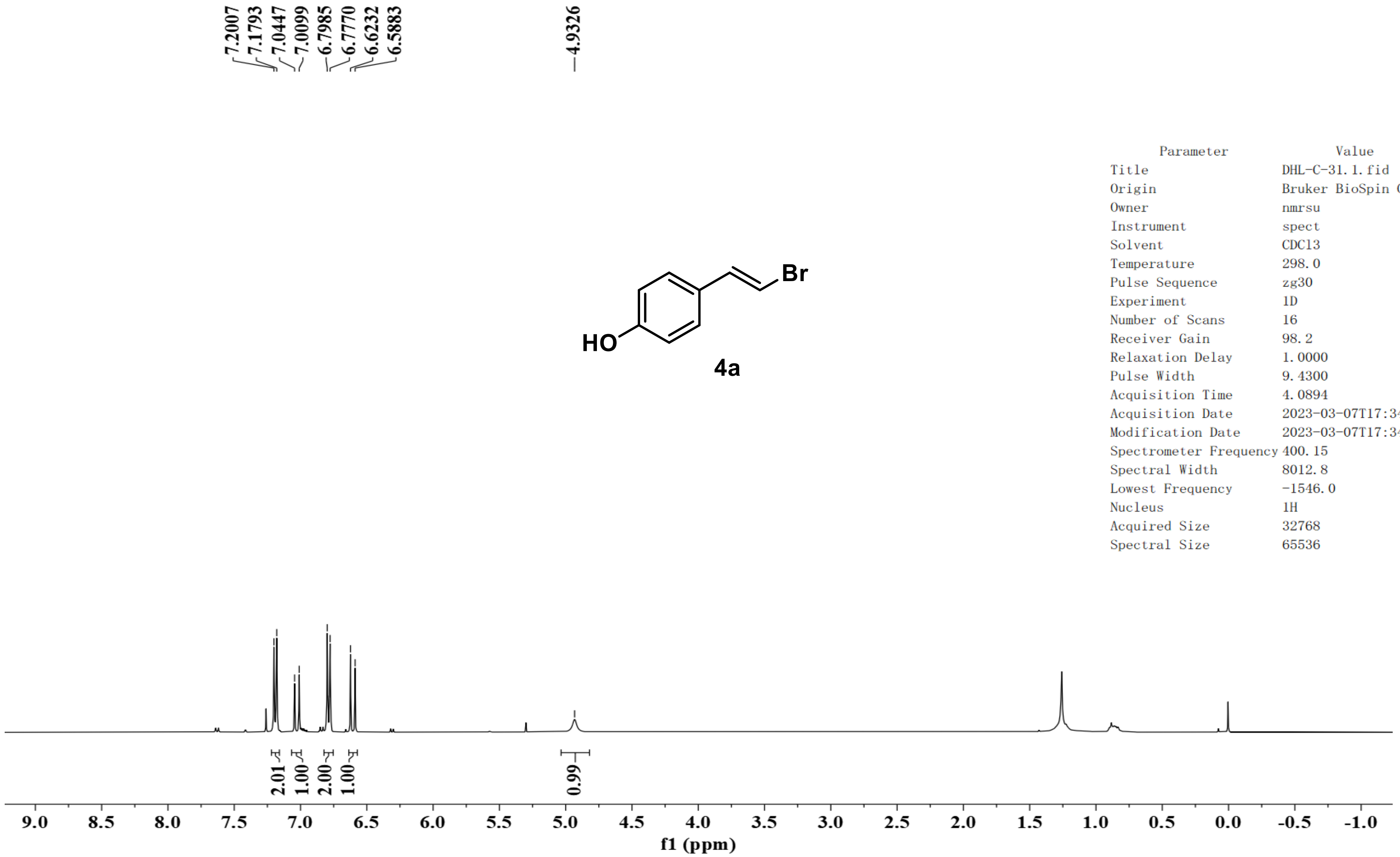

Parameter	Value
Title	DHL-C-31.1.fid
Origin	Bruker BioSpin GmbH
Owner	nmrsu
Instrument	spect
Solvent	CDCl3
Temperature	298.0
Pulse Sequence	zg30
Experiment	1D
Number of Scans	16
Receiver Gain	98.2
Relaxation Delay	1.0000
Pulse Width	9.4300
Acquisition Time	4.0894
Acquisition Date	2023-03-07T17:34:52
Modification Date	2023-03-07T17:34:54
Spectrometer Frequency	400.15
Spectral Width	8012.8
Lowest Frequency	-1546.0
Nucleus	1H
Acquired Size	32768
Spectral Size	65536

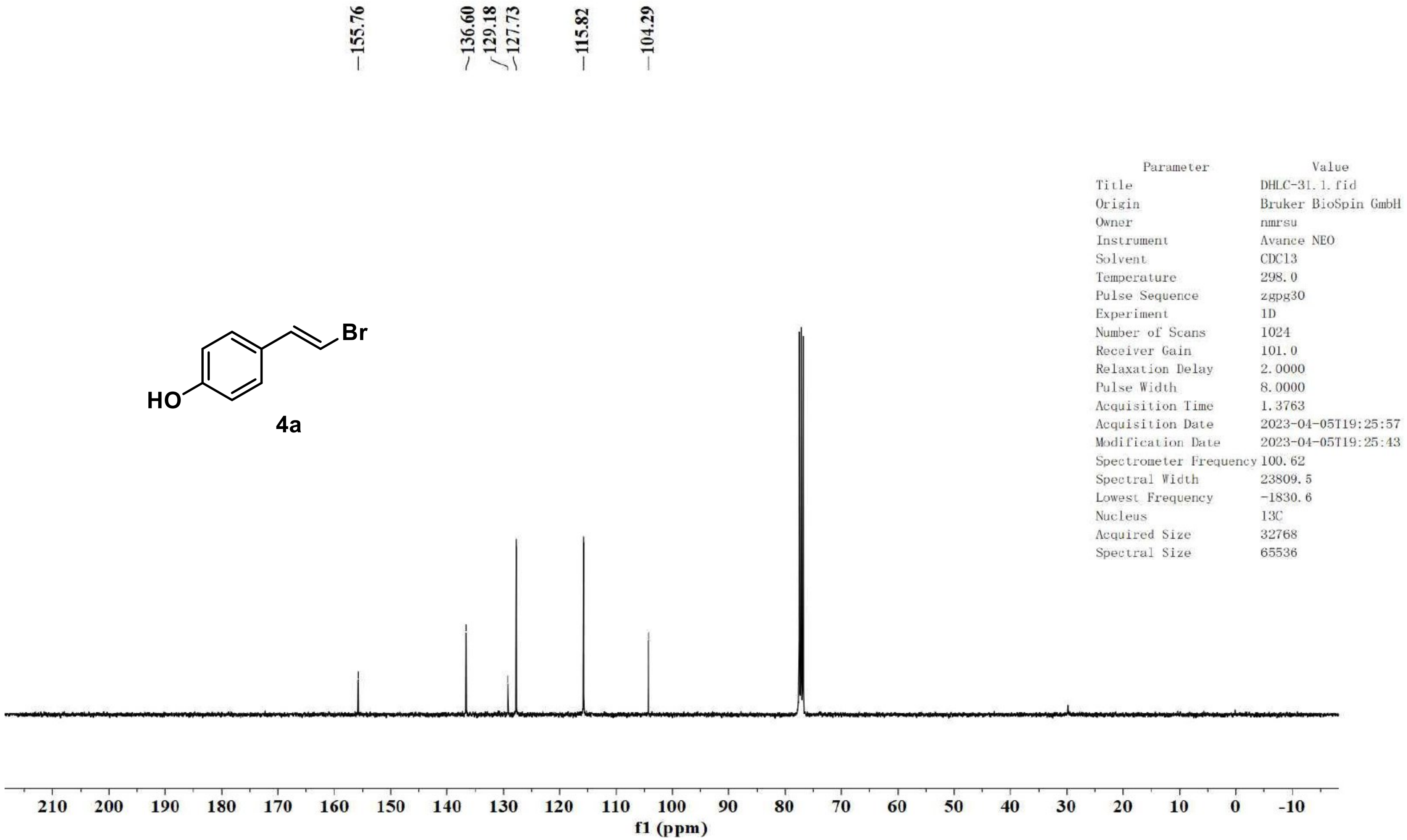
155.76
136.60
129.18
127.73
115.82
104.29
HO
Br
4a
Parameter Value
Title DHLC-31.1.fid
Origin Bruker BioSpin GmbH
Owner nmrsu
Instrument Avance NEO
Solvent CDCl3
Temperature 298.0
Pulse Sequence zgpg30
Experiment 1D
Number of Scans 1024
Receiver Gain 101.0
Relaxation Delay 2.0000
Pulse Width 8.0000
Acquisition Time 1.3763
Acquisition Date 2023-04-05T19:25:57
Modification Date 2023-04-05T19:25:43
Spectrometer Frequency 100.62
Spectral Width 23809.5
Lowest Frequency -1830.6
Nucleus 13C
Acquired Size 32768
Spectral Size 65536
210 200 190 180 170 160 150 140 130 120 110 100 90 80 70 60 50 40 30 20 10 0 -10
f1 (ppm)

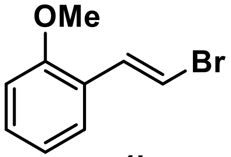

4b

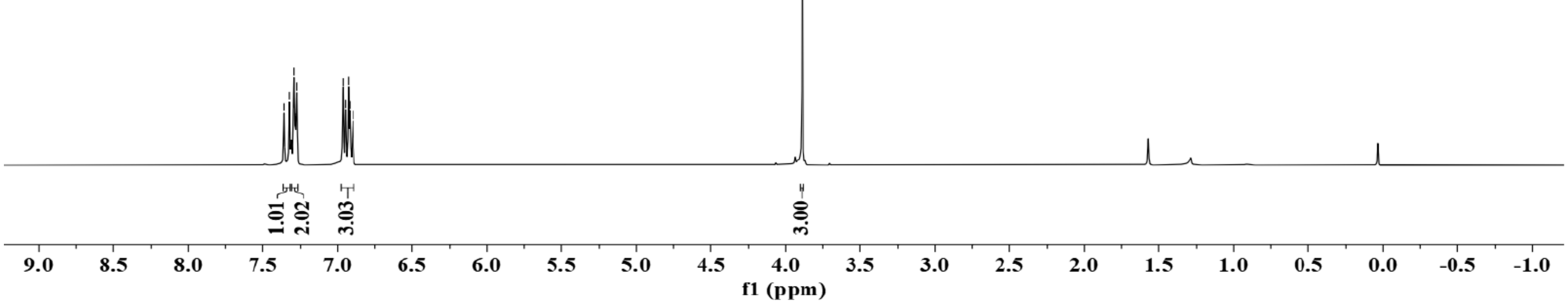

Parameter	Value
Title	C3-36.5.fid
Origin	Bruker BioSpin GmbH
Owner	nmrsu
Instrument	Avance NEO
Solvent	CDCl3
Temperature	298.0
Pulse Sequence	zg30
Experiment	1D
Number of Scans	16
Receiver Gain	101.0
Relaxation Delay	1.0000
Pulse Width	8.0000
Acquisition Time	3.9977
Acquisition Date	2023-07-13T12:46:53
Modification Date	2023-07-13T12:47:12
Spectrometer Frequency	400.13
Spectral Width	8196.7
Lowest Frequency	-1627.6
Nucleus	1H
Acquired Size	32768
Spectral Size	65536

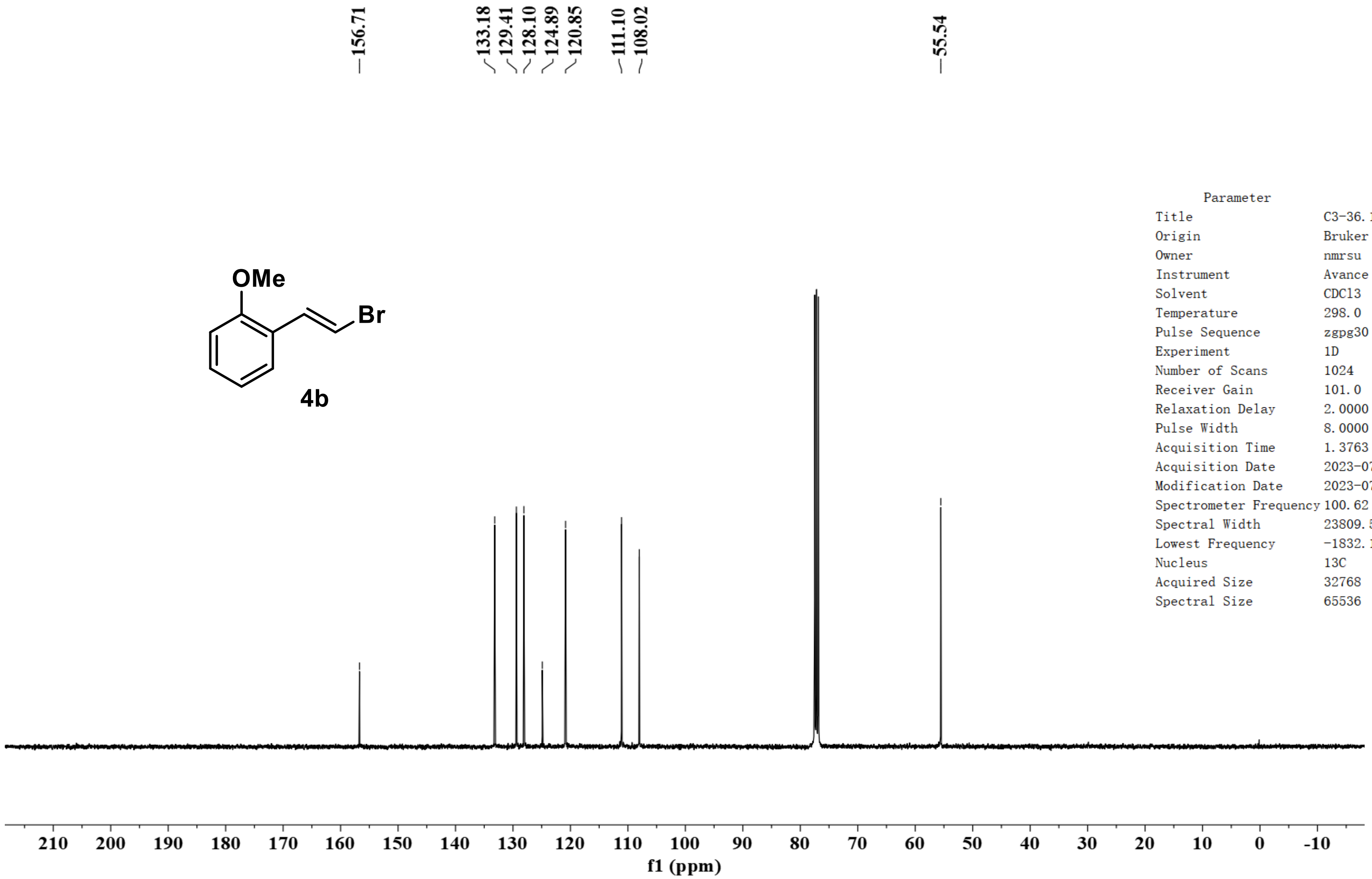

Parameter	Value
Title	C3-36.10.fid
Origin	Bruker BioSpin GmbH
Owner	nmrsu
Instrument	Avance NEO
Solvent	CDCl3
Temperature	298.0
Pulse Sequence	zgpg30
Experiment	1D
Number of Scans	1024
Receiver Gain	101.0
Relaxation Delay	2.0000
Pulse Width	8.0000
Acquisition Time	1.3763
Acquisition Date	2023-07-15T18:55:59
Modification Date	2023-07-15T18:56:21
Spectrometer Frequency	100.62
Spectral Width	23809.5
Lowest Frequency	-1832.1
Nucleus	13C
Acquired Size	32768
Spectral Size	65536

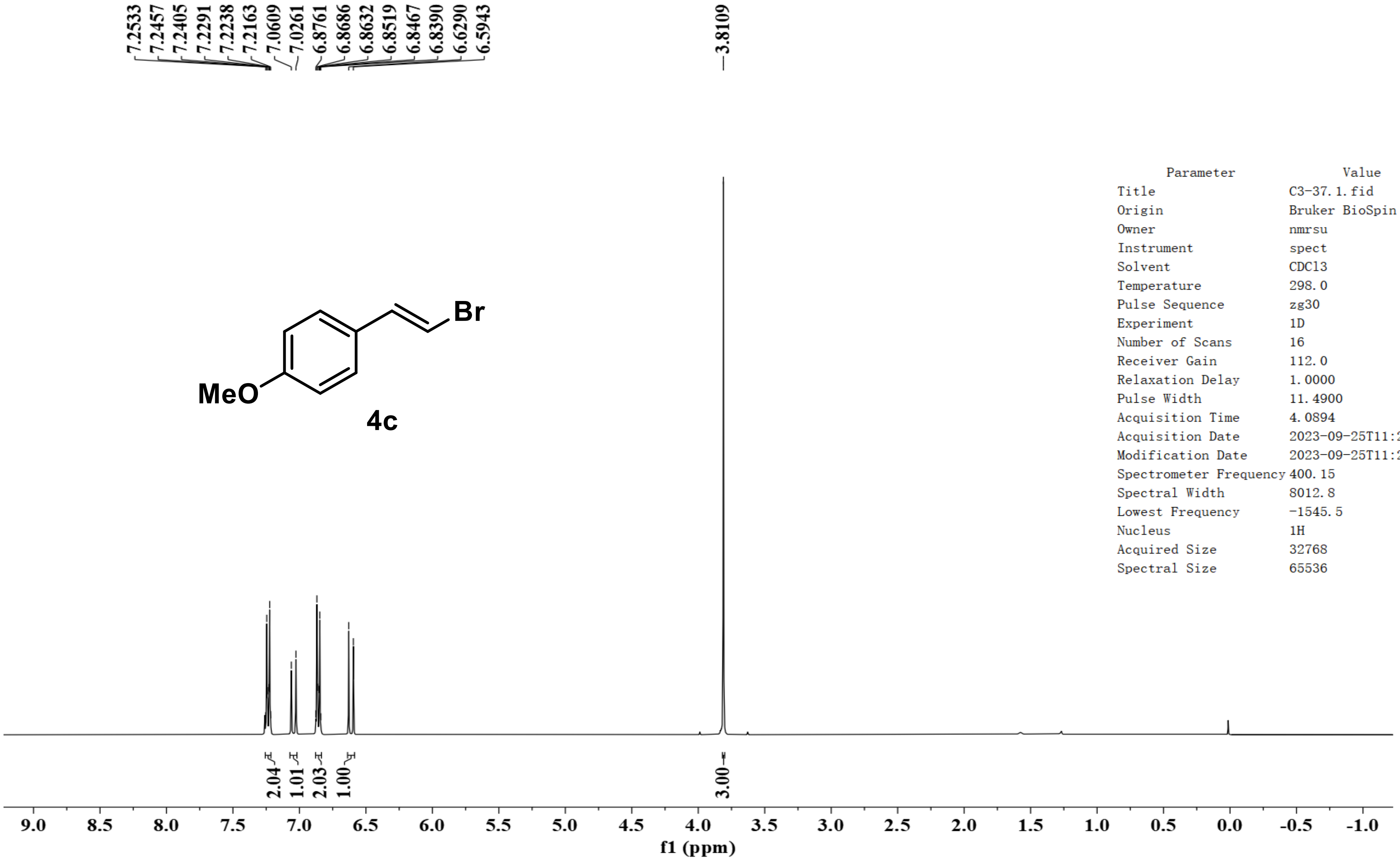

Parameter	Value
Title	C3-37.1.fid
Origin	Bruker BioSpin GmbH
Owner	nmrsu
Instrument	spect
Solvent	CDCl3
Temperature	298.0
Pulse Sequence	zg30
Experiment	1D
Number of Scans	16
Receiver Gain	112.0
Relaxation Delay	1.0000
Pulse Width	11.4900
Acquisition Time	4.0894
Acquisition Date	2023-09-25T11:23:19
Modification Date	2023-09-25T11:23:20
Spectrometer Frequency	400.15
Spectral Width	8012.8
Lowest Frequency	-1545.5
Nucleus	1H
Acquired Size	32768
Spectral Size	65536

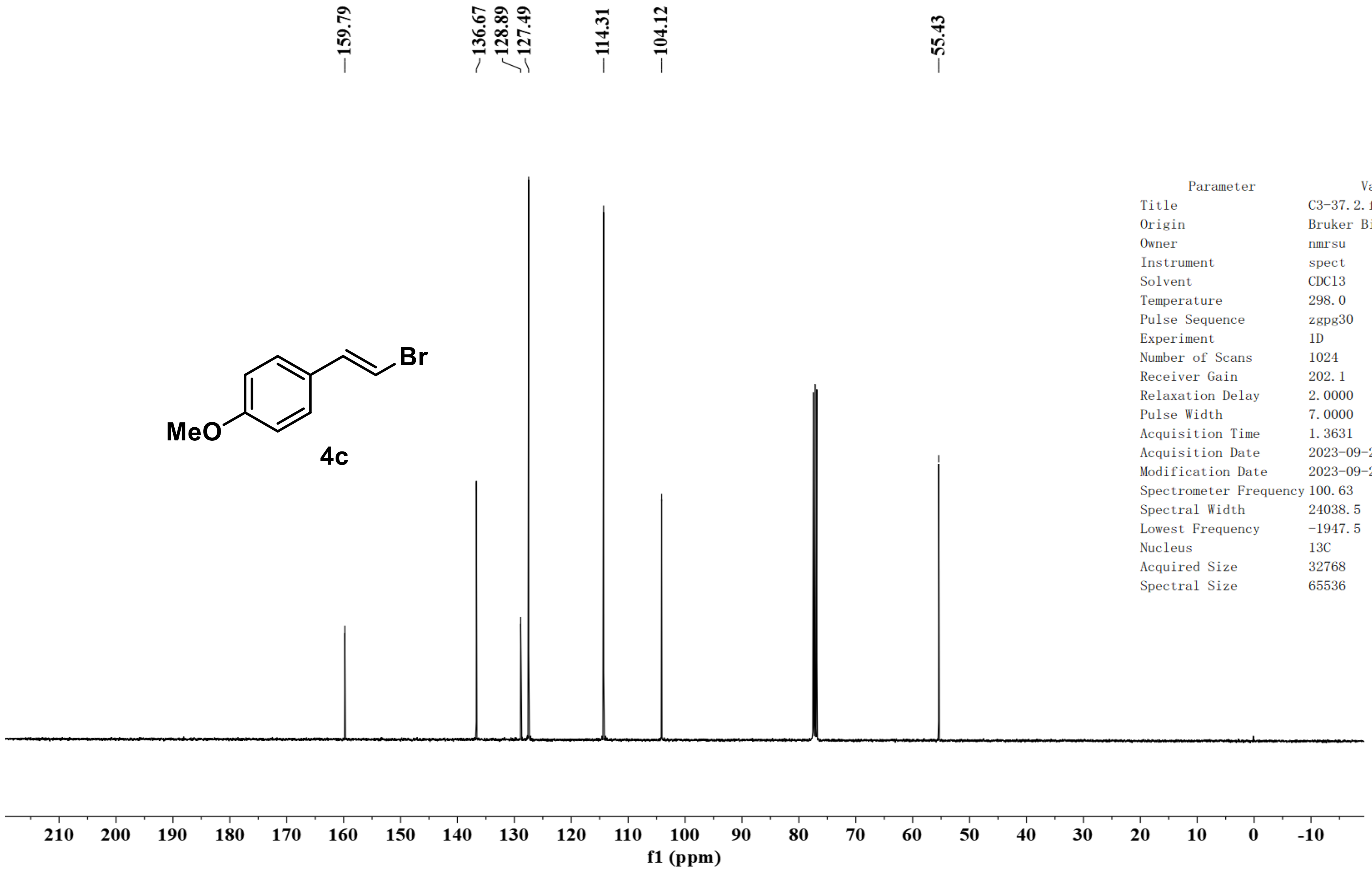

Parameter	Value
Title	C3-37.2.fid
Origin	Bruker BioSpin GmbH
Owner	nmrsu
Instrument	spect
Solvent	CDCl3
Temperature	298.0
Pulse Sequence	zgpg30
Experiment	1D
Number of Scans	1024
Receiver Gain	202.1
Relaxation Delay	2.0000
Pulse Width	7.0000
Acquisition Time	1.3631
Acquisition Date	2023-09-26T00:09:28
Modification Date	2023-09-26T00:09:30
Spectrometer Frequency	100.63
Spectral Width	24038.5
Lowest Frequency	-1947.5
Nucleus	13C
Acquired Size	32768
Spectral Size	65536

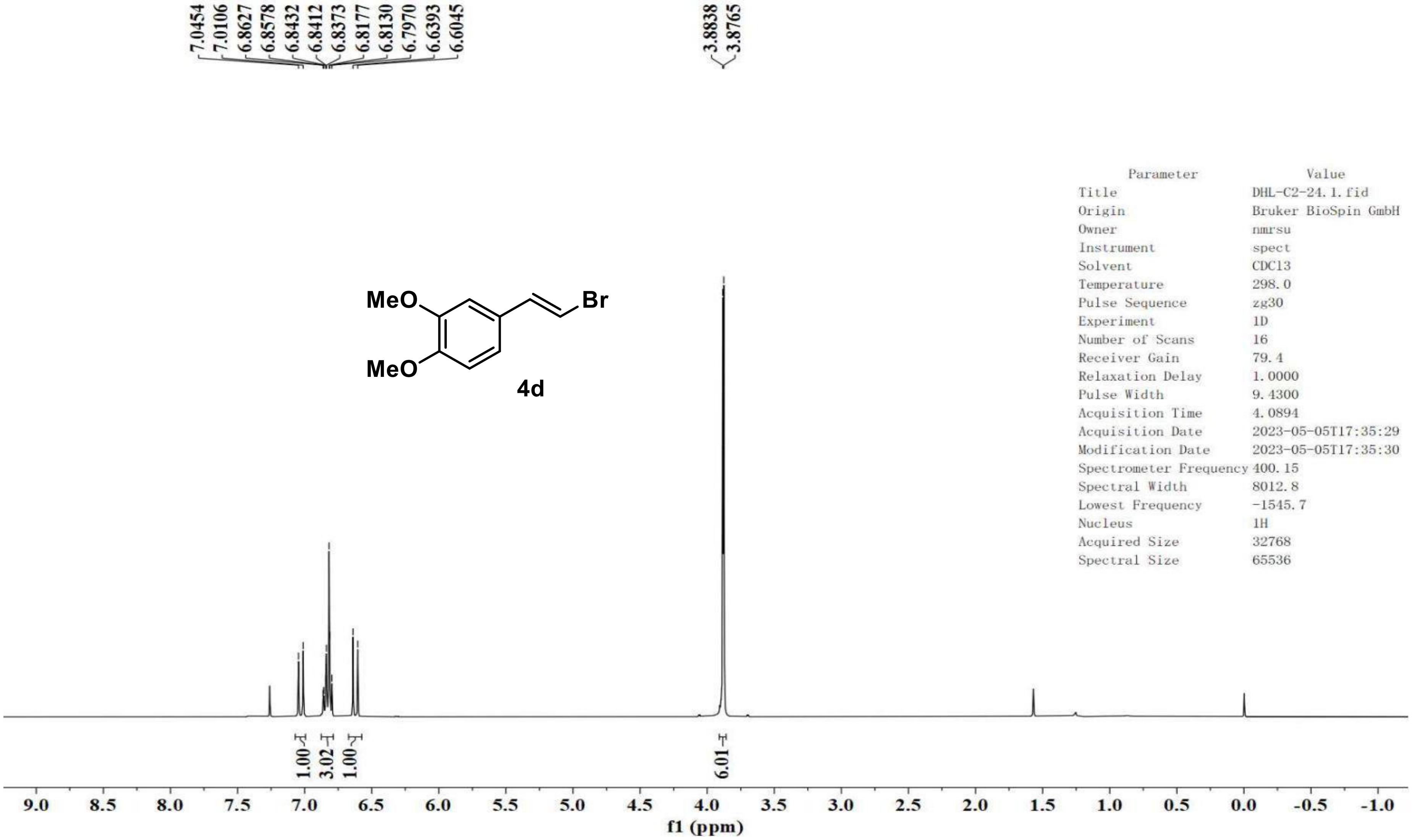

Parameter	Value
Title	DHL-C2-24.1.fid
Origin	Bruker BioSpin GmbH
Owner	nmrsu
Instrument	spect
Solvent	CDCl3
Temperature	298.0
Pulse Sequence	zg30
Experiment	1D
Number of Scans	16
Receiver Gain	79.4
Relaxation Delay	1.0000
Pulse Width	9.4300
Acquisition Time	4.0894
Acquisition Date	2023-05-05T17:35:29
Modification Date	2023-05-05T17:35:30
Spectrometer Frequency	400.15
Spectral Width	8012.8
Lowest Frequency	-1545.7
Nucleus	1H
Acquired Size	32768
Spectral Size	65536

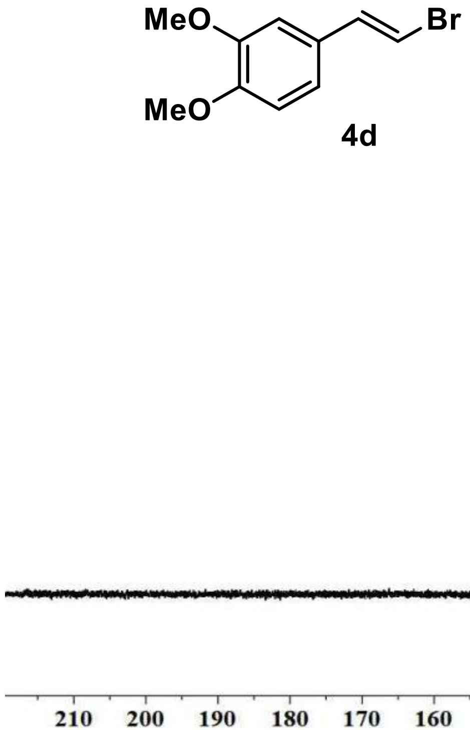

4d

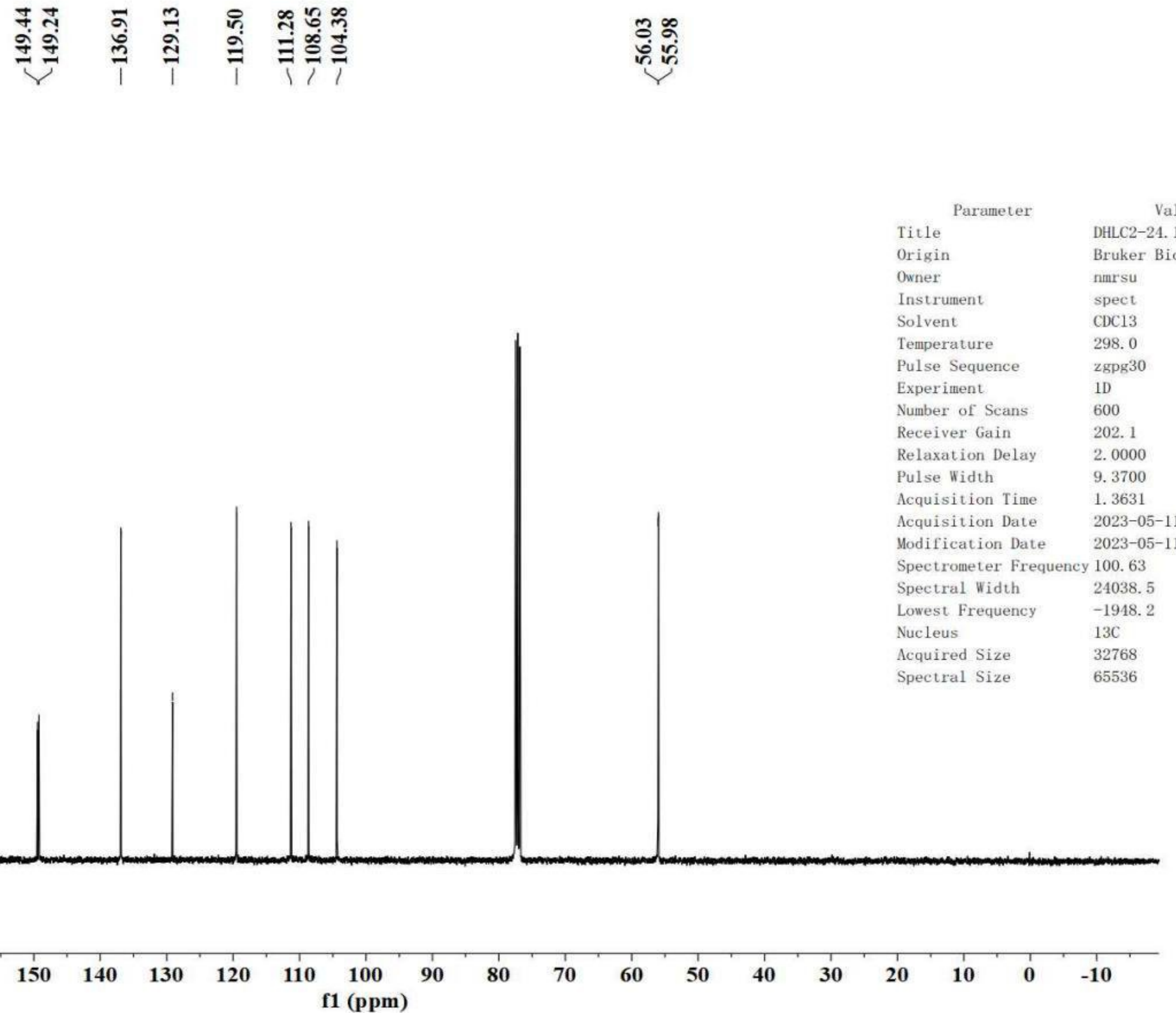

Parameter	Value
Title	DHLC2-24.1.fid
Origin	Bruker BioSpin GmbH
Owner	nmrsu
Instrument	spect
Solvent	CDCl3
Temperature	298.0
Pulse Sequence	zgpg30
Experiment	1D
Number of Scans	600
Receiver Gain	202.1
Relaxation Delay	2.0000
Pulse Width	9.3700
Acquisition Time	1.3631
Acquisition Date	2023-05-11T22:58:18
Modification Date	2023-05-11T22:58:20
Spectrometer Frequency	100.63
Spectral Width	24038.5
Lowest Frequency	-1948.2
Nucleus	13C
Acquired Size	32768
Spectral Size	65536

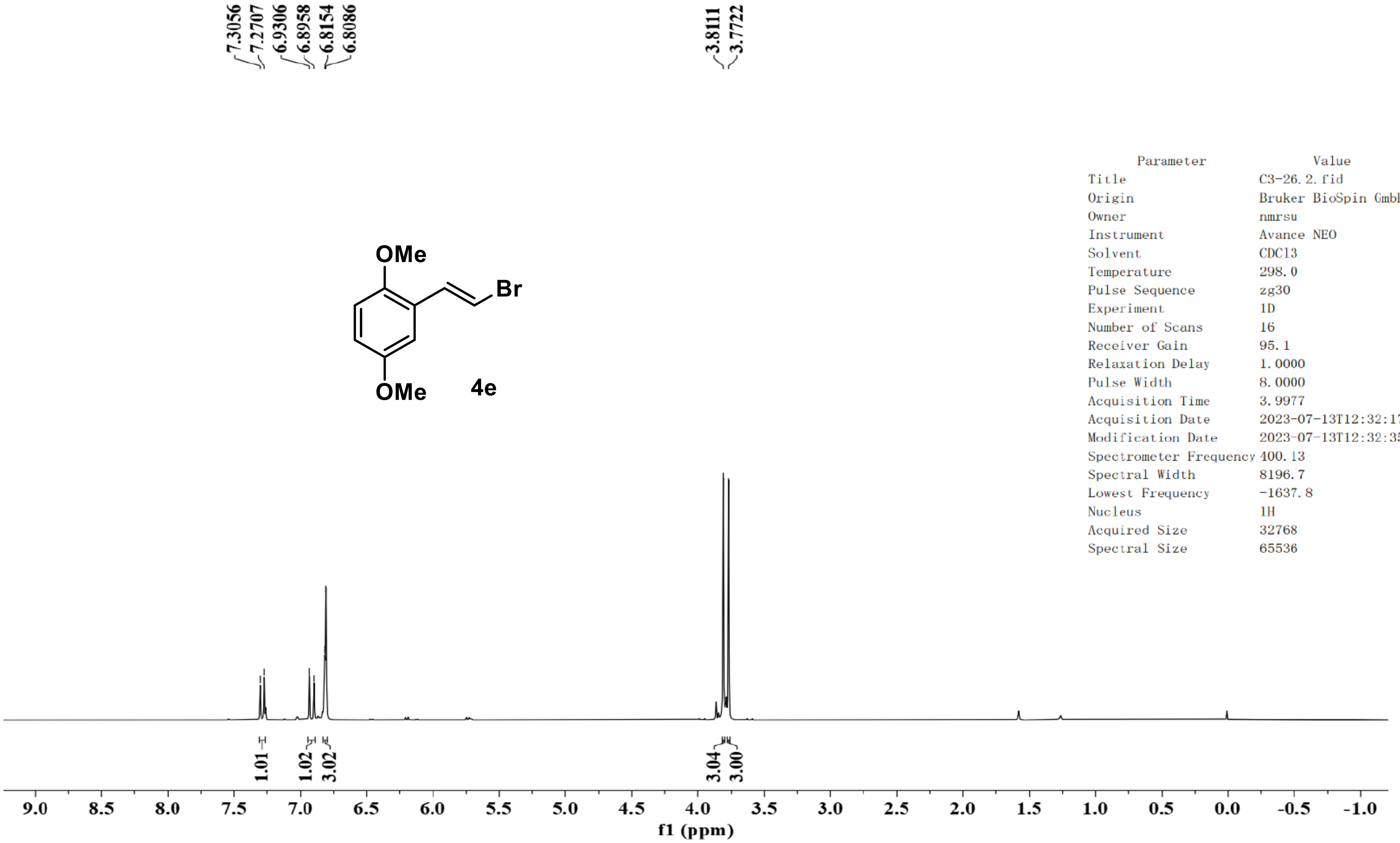

Parameter	Value
Title	C3-26.2.fid
Origin	Bruker BioSpin GmbH
Owner	nmrsu
Instrument	Avance NEO
Solvent	CDCl3
Temperature	298.0
Pulse Sequence	zg30
Experiment	1D
Number of Scans	16
Receiver Gain	95.1
Relaxation Delay	1.0000
Pulse Width	8.0000
Acquisition Time	3.9977
Acquisition Date	2023-07-13T12:32:17
Modification Date	2023-07-13T12:32:35
Spectrometer Frequency	400.13
Spectral Width	8196.7
Lowest Frequency	-1637.8
Nucleus	1H
Acquired Size	32768
Spectral Size	65536

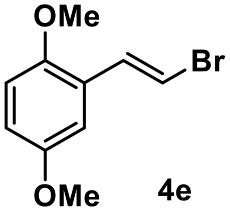

4e

Parameter	Value
Title	C3-26.8.fid
Origin	Bruker BioSpin GmbH
Owner	nmrsu
Instrument	Avance NEO
Solvent	CDCl3
Temperature	298.0
Pulse Sequence	zgpg30
Experiment	1D
Number of Scans	1024
Receiver Gain	101.0
Relaxation Delay	2.0000
Pulse Width	8.0000
Acquisition Time	1.3763
Acquisition Date	2023-07-15T16:41:48
Modification Date	2023-07-15T16:42:11
Spectrometer Frequency	100.62
Spectral Width	23809.5
Lowest Frequency	-1833.6
Nucleus	13C
Acquired Size	32768
Spectral Size	65536

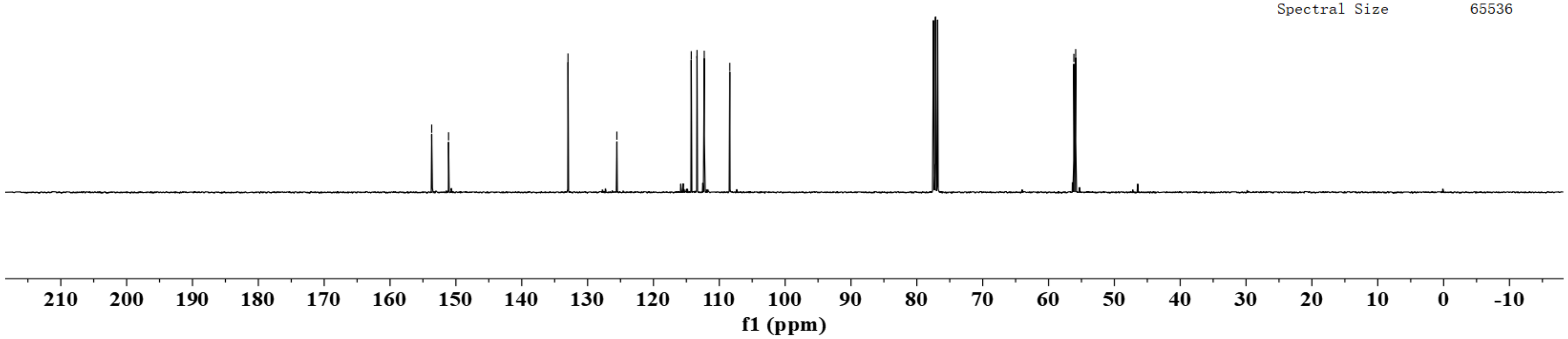

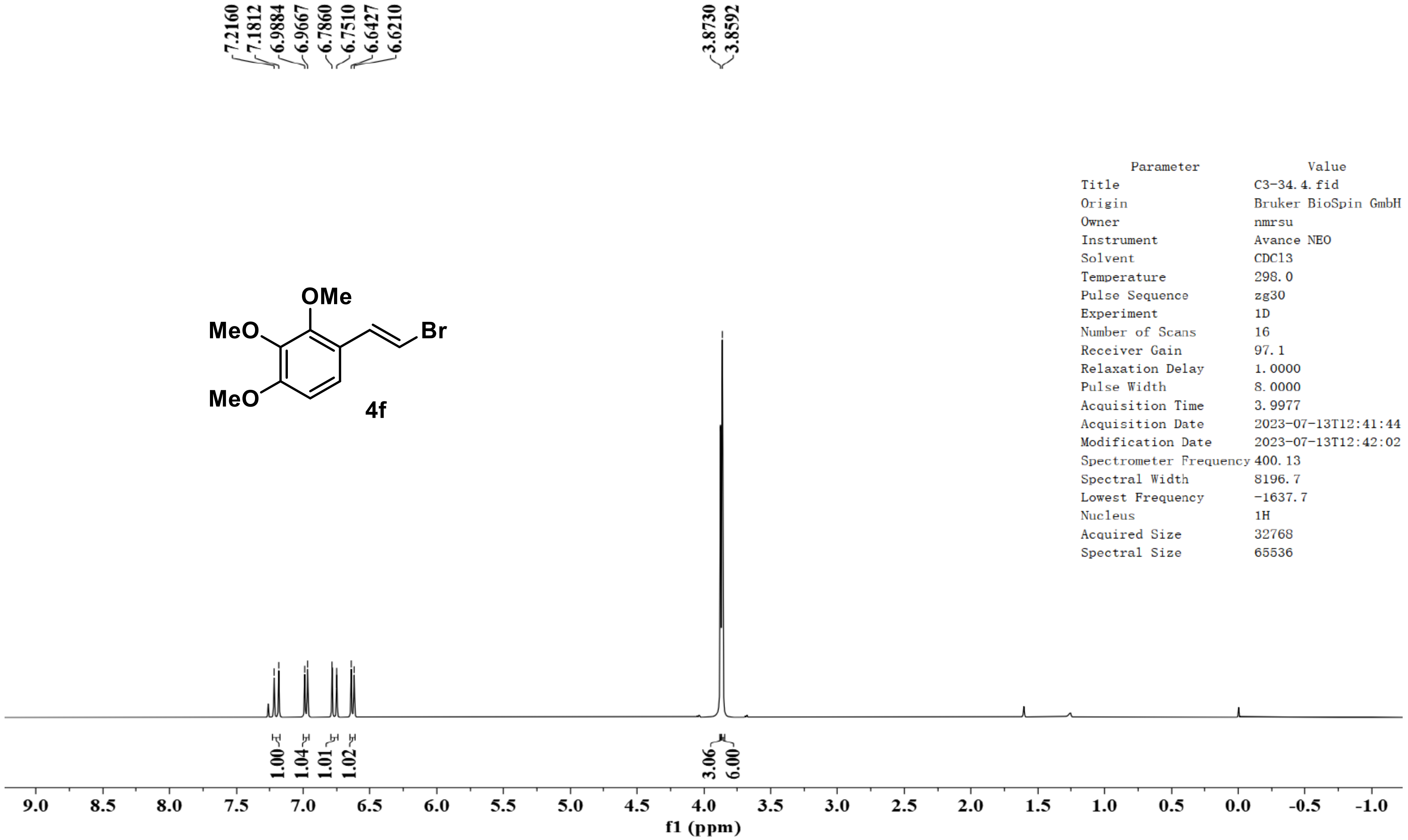

4f

Parameter	Value
Title	C3-34.4.fid
Origin	Bruker BioSpin GmbH
Owner	nmrsu
Instrument	Avance NEO
Solvent	CDCl3
Temperature	298.0
Pulse Sequence	zg30
Experiment	1D
Number of Scans	16
Receiver Gain	97.1
Relaxation Delay	1.0000
Pulse Width	8.0000
Acquisition Time	3.9977
Acquisition Date	2023-07-13T12:41:44
Modification Date	2023-07-13T12:42:02
Spectrometer Frequency	400.13
Spectral Width	8196.7
Lowest Frequency	-1637.7
Nucleus	1H
Acquired Size	32768
Spectral Size	65536

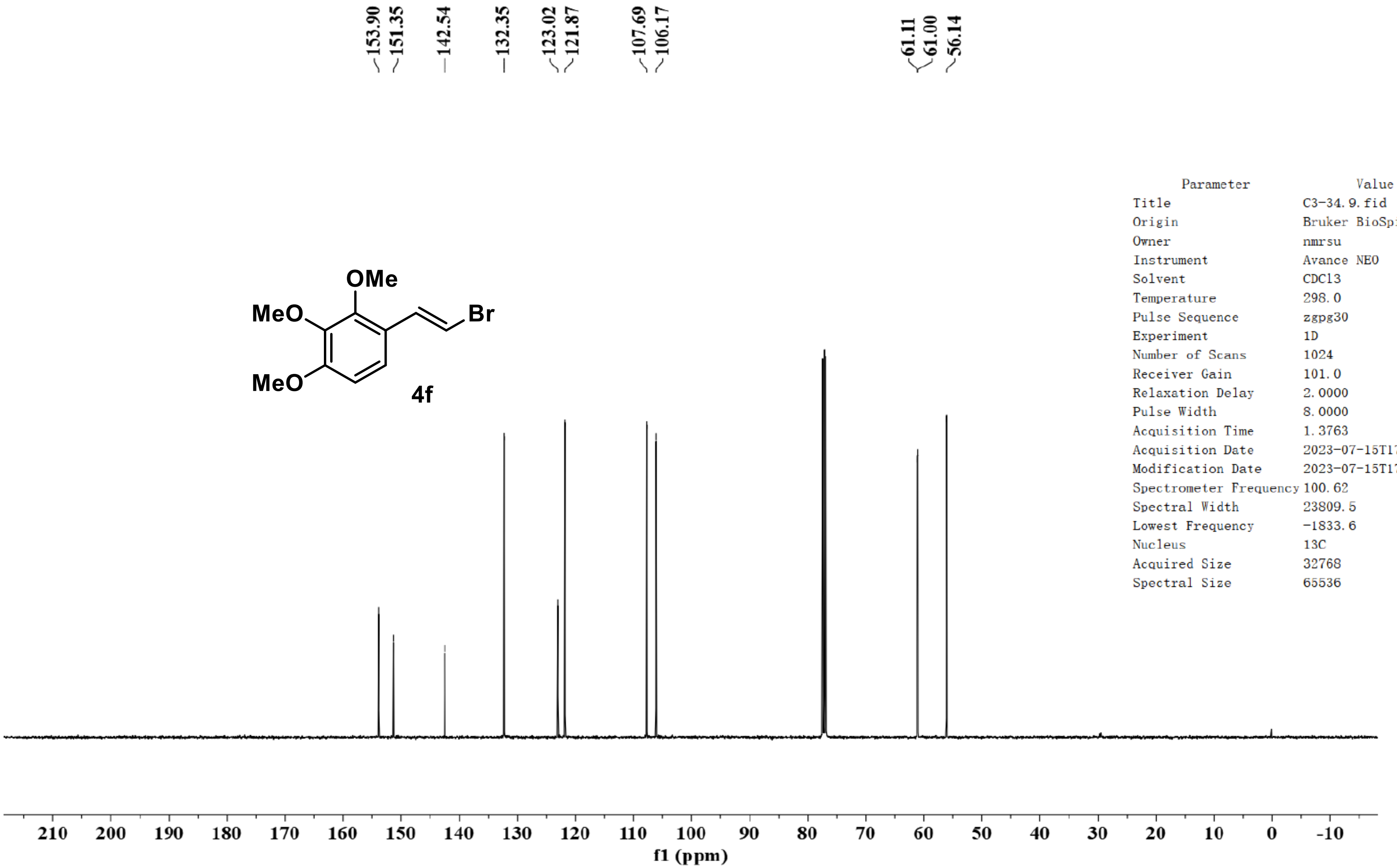

Parameter	Value
Title	C3-34.9.fid
Origin	Bruker BioSpin GmbH
Owner	nmrsu
Instrument	Avance NEO
Solvent	CDCl3
Temperature	298.0
Pulse Sequence	zgpg30
Experiment	1D
Number of Scans	1024
Receiver Gain	101.0
Relaxation Delay	2.0000
Pulse Width	8.0000
Acquisition Time	1.3763
Acquisition Date	2023-07-15T17:52:21
Modification Date	2023-07-15T17:52:43
Spectrometer Frequency	100.62
Spectral Width	23809.5
Lowest Frequency	-1833.6
Nucleus	13C
Acquired Size	32768
Spectral Size	65536

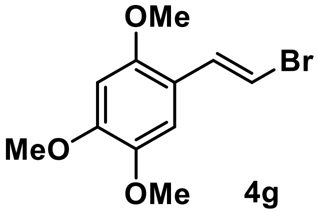

4g

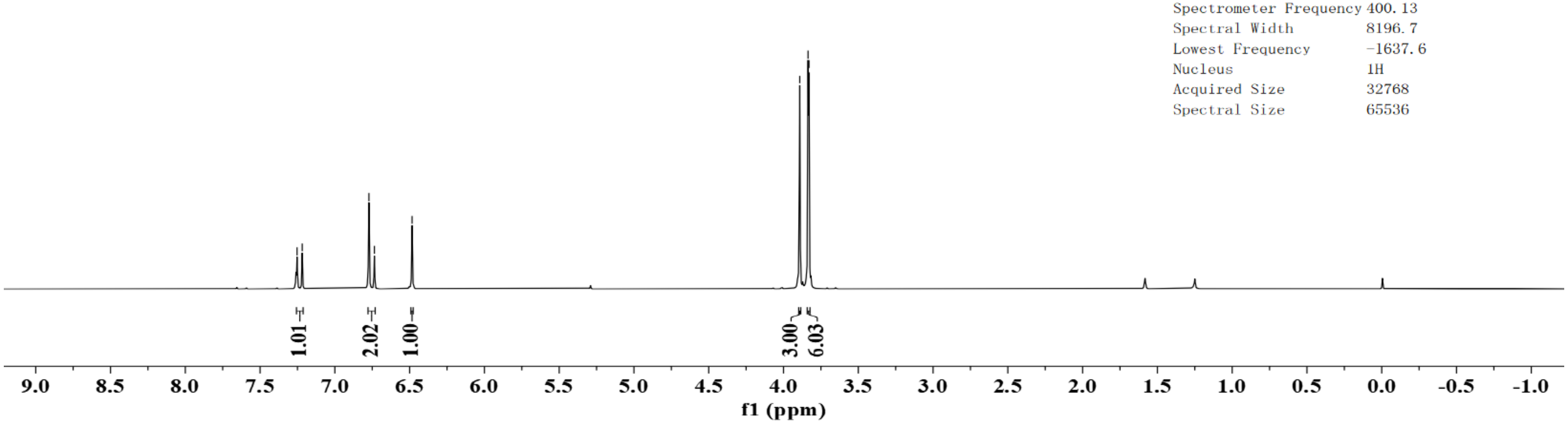

Parameter	Value
Title	C3-33.4.fid
Origin	Bruker BioSpin GmbH
Owner	nmrsu
Instrument	Avance NEO
Solvent	CDCl3
Temperature	298.0
Pulse Sequence	zg30
Experiment	1D
Number of Scans	16
Receiver Gain	101.0
Relaxation Delay	1.0000
Pulse Width	8.0000
Acquisition Time	3.9977
Acquisition Date	2023-06-28T20:15:22
Modification Date	2023-06-28T20:15:16
Spectrometer Frequency	400.13
Spectral Width	8196.7
Lowest Frequency	-1637.6
Nucleus	1H
Acquired Size	32768
Spectral Size	65536

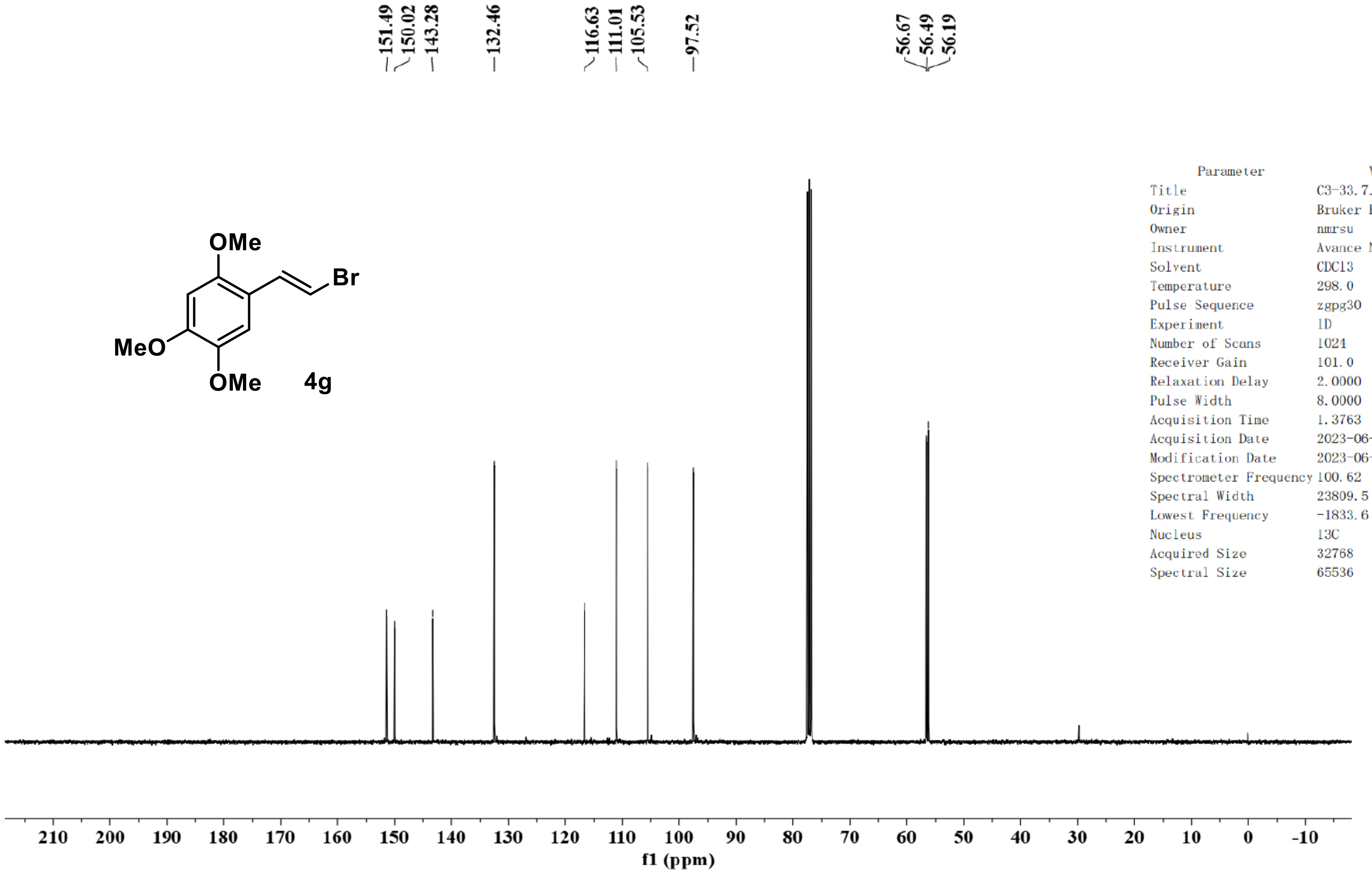

Parameter	Value
Title	C3-33.7.fid
Origin	Bruker BioSpin GmbH
Owner	nmrsu
Instrument	Avance NEO
Solvent	CDCl3
Temperature	298.0
Pulse Sequence	zgpg30
Experiment	1D
Number of Scans	1024
Receiver Gain	101.0
Relaxation Delay	2.0000
Pulse Width	8.0000
Acquisition Time	1.3763
Acquisition Date	2023-06-30T11:22:14
Modification Date	2023-06-30T11:22:11
Spectrometer Frequency	100.62
Spectral Width	23809.5
Lowest Frequency	-1833.6
Nucleus	13C
Acquired Size	32768
Spectral Size	65536

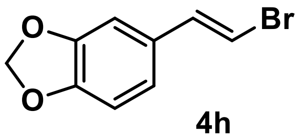

4h

Parameter	Value
Title	C3-24.1.fid
Origin	Bruker BioSpin GmbH
Owner	nmrsu
Instrument	Avance NEO
Solvent	CDCl3
Temperature	298.0
Pulse Sequence	zg30
Experiment	1D
Number of Scans	16
Receiver Gain	101.0
Relaxation Delay	1.0000
Pulse Width	8.0000
Acquisition Time	3.9977
Acquisition Date	2023-07-13T12:27:19
Modification Date	2023-07-13T12:27:37
Spectrometer Frequency	400.13
Spectral Width	8196.7
Lowest Frequency	-1637.7
Nucleus	1H
Acquired Size	32768
Spectral Size	65536

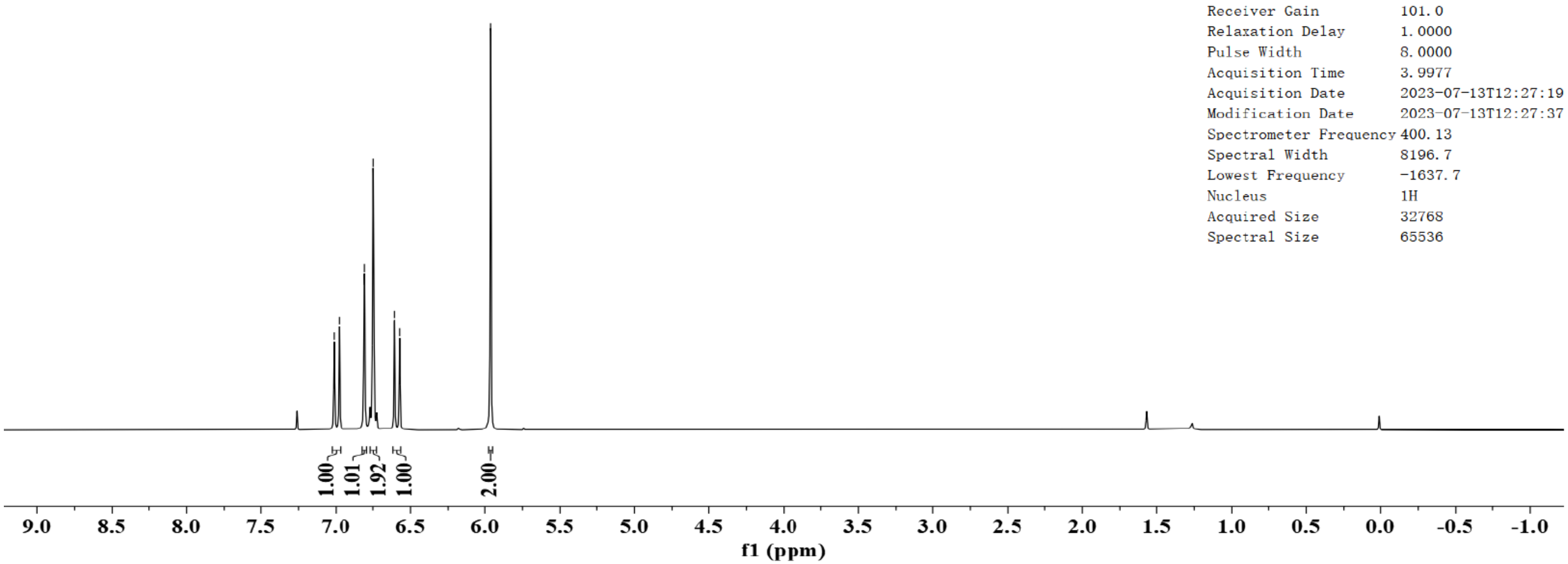

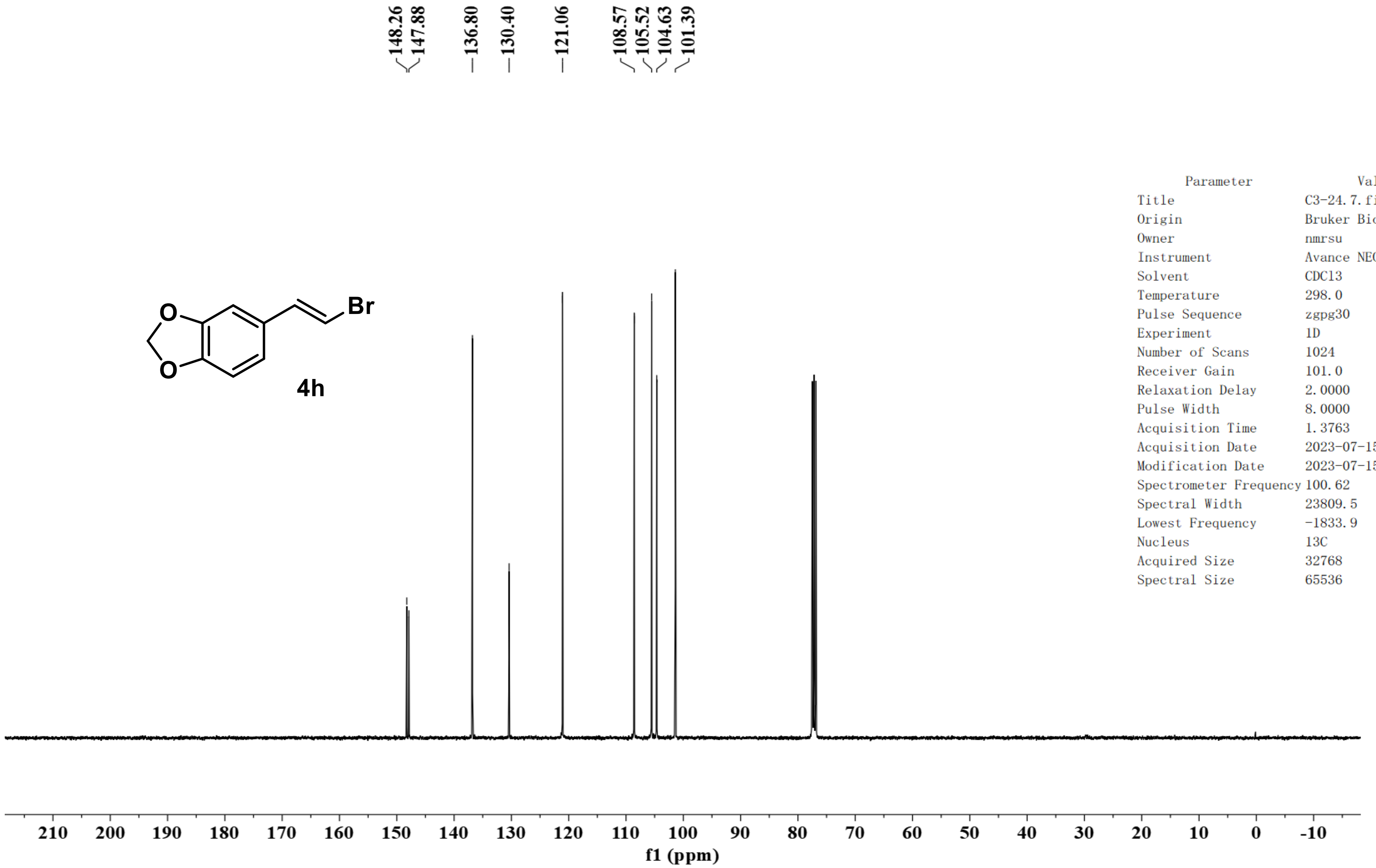

Parameter	Value
Title	C3-24.7.fid
Origin	Bruker BioSpin GmbH
Owner	nmrsu
Instrument	Avance NEO
Solvent	CDCl3
Temperature	298.0
Pulse Sequence	zgpg30
Experiment	1D
Number of Scans	1024
Receiver Gain	101.0
Relaxation Delay	2.0000
Pulse Width	8.0000
Acquisition Time	1.3763
Acquisition Date	2023-07-15T15:38:28
Modification Date	2023-07-15T15:38:50
Spectrometer Frequency	100.62
Spectral Width	23809.5
Lowest Frequency	-1833.9
Nucleus	13C
Acquired Size	32768
Spectral Size	65536

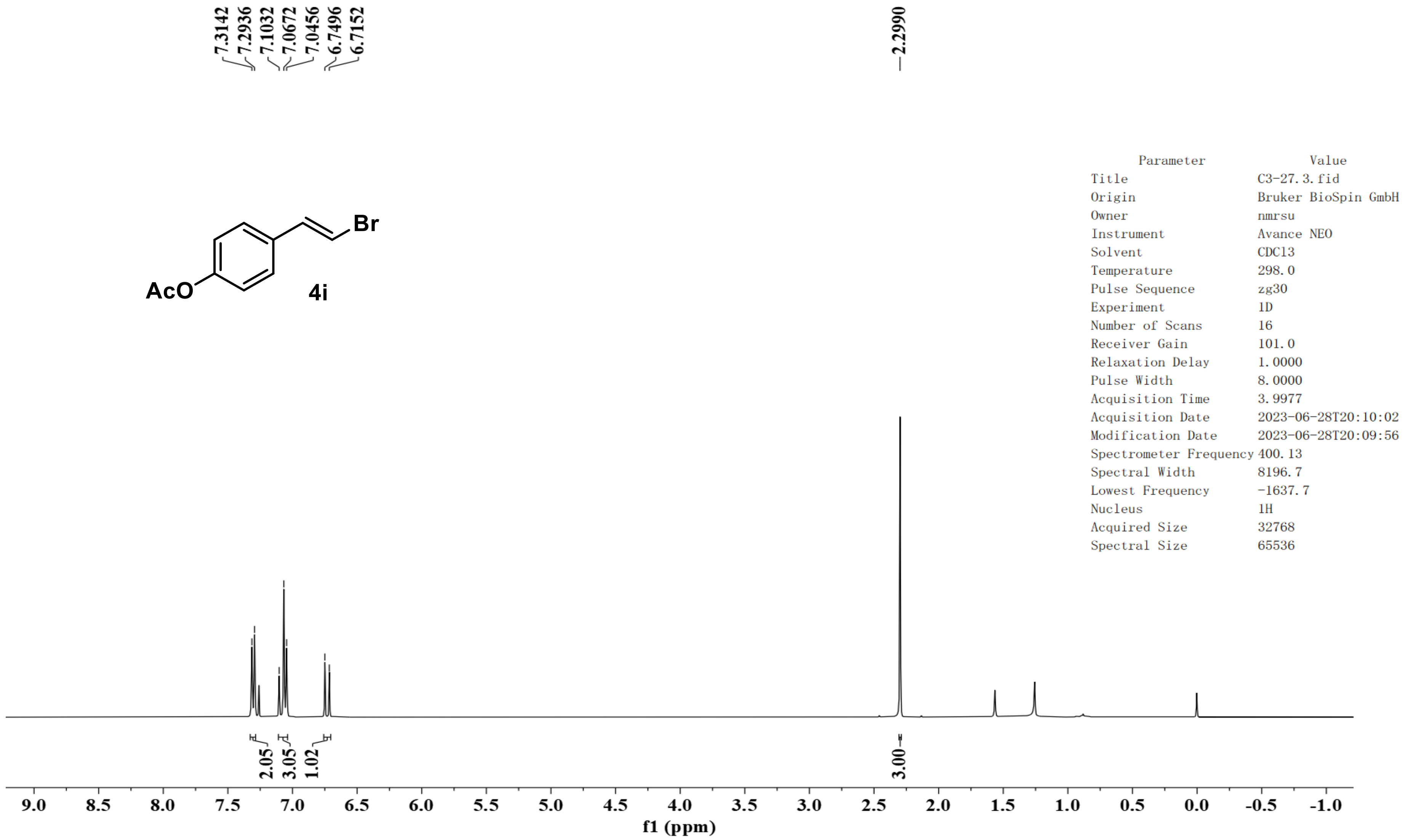

7.3142
7.2936
7.1032
7.0672
7.0456
6.7496
6.7152
2.2990
Br
AcO
4i
Parameter Value
Title C3-27.3.fid
Origin Bruker BioSpin GmbH
Owner nmrsu
Instrument Avance NEO
Solvent CDCl3
Temperature 298.0
Pulse Sequence zg30
Experiment 1D
Number of Scans 16
Receiver Gain 101.0
Relaxation Delay 1.0000
Pulse Width 8.0000
Acquisition Time 3.9977
Acquisition Date 2023-06-28T20:10:02
Modification Date 2023-06-28T20:09:56
Spectrometer Frequency 400.13
Spectral Width 8196.7
Lowest Frequency -1637.7
Nucleus 1H
Acquired Size 32768
Spectral Size 65536
2.05
3.05
1.02
3.00
9.0 8.5 8.0 7.5 7.0 6.5 6.0 5.5 5.0 4.5 4.0 3.5 3.0 2.5 2.0 1.5 1.0 0.5 0.0 -0.5 -1.0
f1 (ppm)

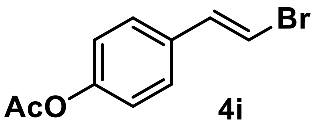

4i

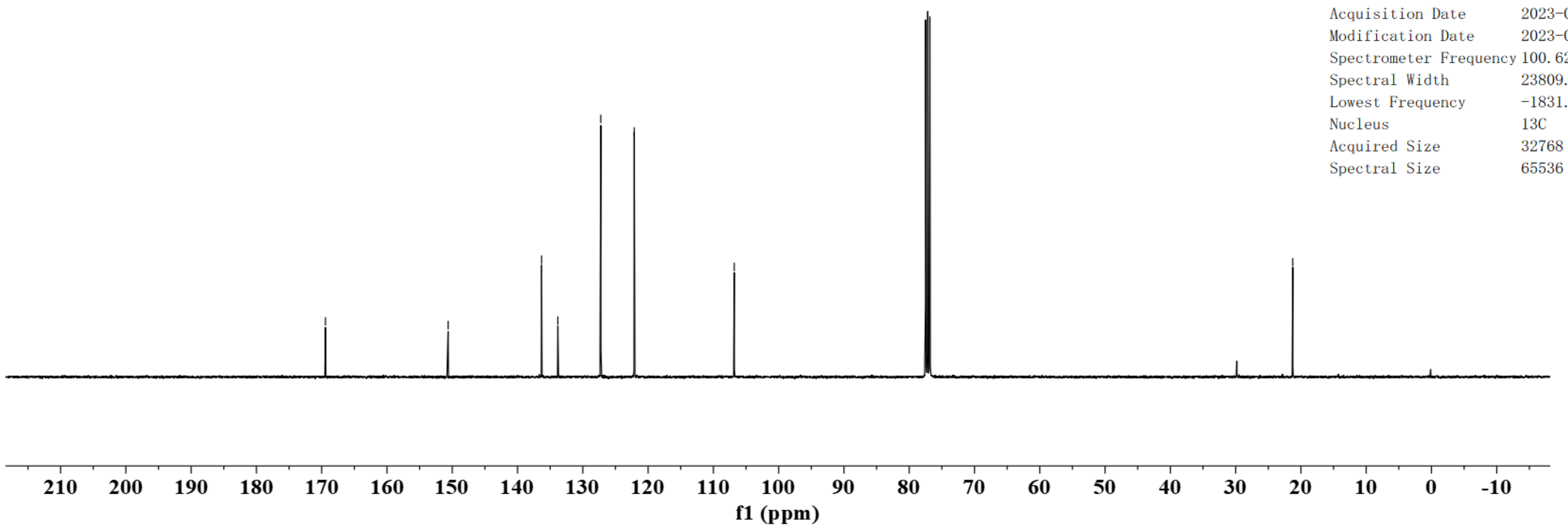

Parameter	Value
Title	C3-27.6.fid
Origin	Bruker BioSpin GmbH
Owner	nmrsu
Instrument	Avance NEO
Solvent	CDCl3
Temperature	298.0
Pulse Sequence	zgpg30
Experiment	1D
Number of Scans	1024
Receiver Gain	101.0
Relaxation Delay	2.0000
Pulse Width	8.0000
Acquisition Time	1.3763
Acquisition Date	2023-06-30T10:18:17
Modification Date	2023-06-30T10:18:14
Spectrometer Frequency	100.62
Spectral Width	23809.5
Lowest Frequency	-1831.7
Nucleus	13C
Acquired Size	32768
Spectral Size	65536

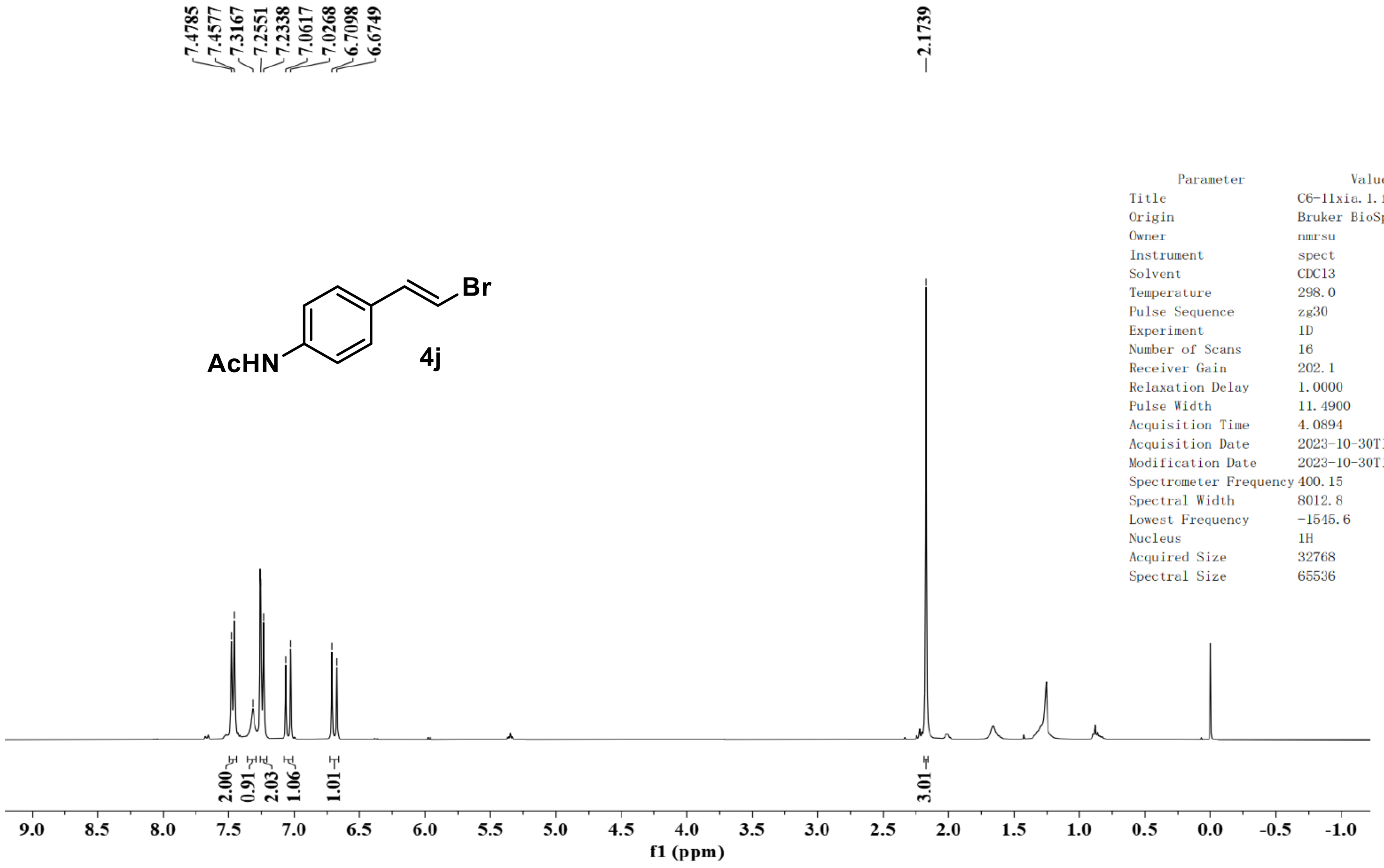

Parameter	Value
Title	C6-11xia.1.fid
Origin	Bruker BioSpin GmbH
Owner	nmrsu
Instrument	spect
Solvent	CDCl3
Temperature	298.0
Pulse Sequence	zg30
Experiment	1D
Number of Scans	16
Receiver Gain	202.1
Relaxation Delay	1.0000
Pulse Width	11.4900
Acquisition Time	4.0894
Acquisition Date	2023-10-30T15:19:45
Modification Date	2023-10-30T15:19:44
Spectrometer Frequency	400.15
Spectral Width	8012.8
Lowest Frequency	-1545.6
Nucleus	1H
Acquired Size	32768
Spectral Size	65536

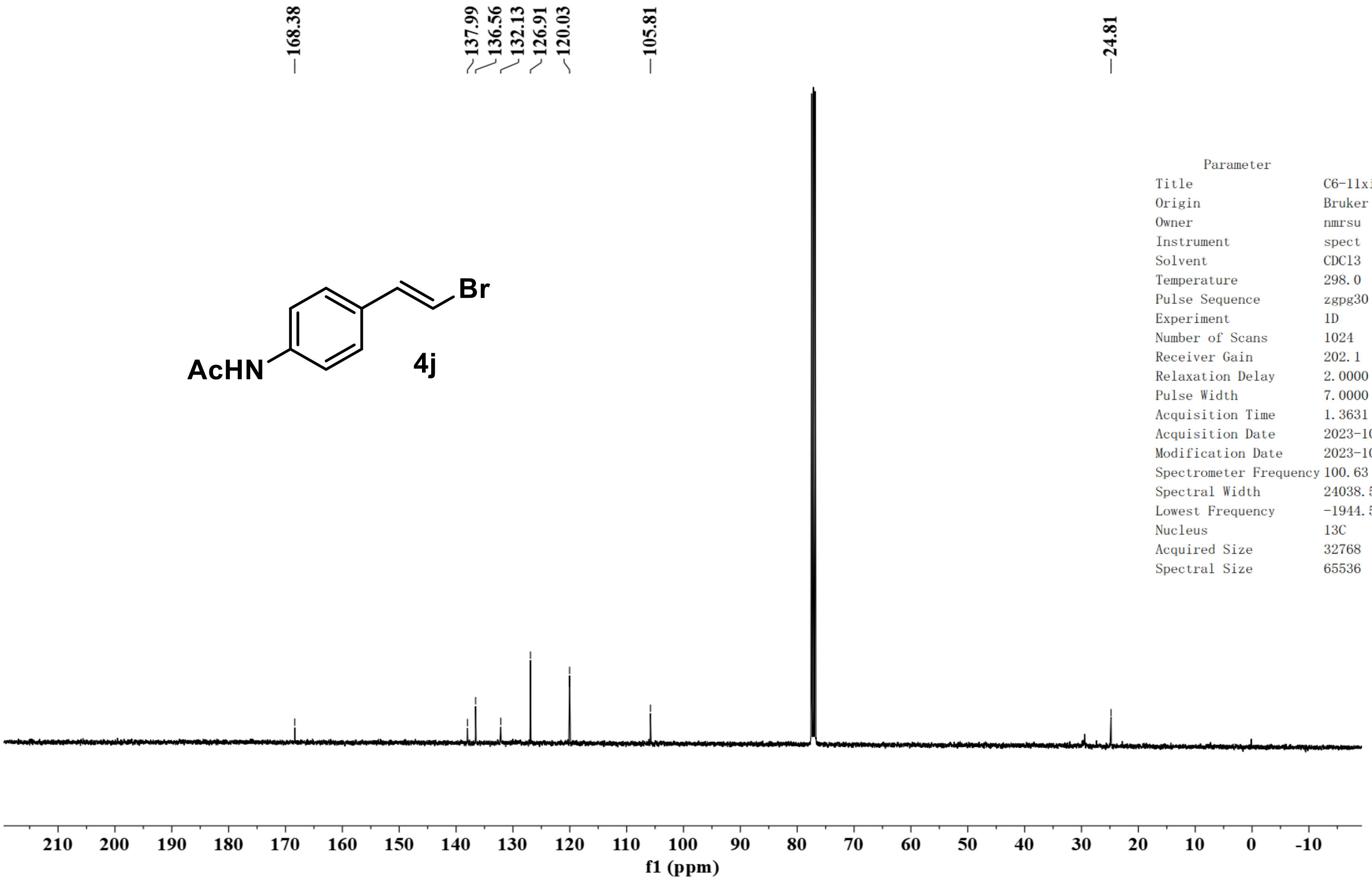

Parameter	Value
Title	C6-11xia-C.1.fid
Origin	Bruker BioSpin GmbH
Owner	nmrsu
Instrument	spect
Solvent	CDCl3
Temperature	298.0
Pulse Sequence	zgpg30
Experiment	1D
Number of Scans	1024
Receiver Gain	202.1
Relaxation Delay	2.0000
Pulse Width	7.0000
Acquisition Time	1.3631
Acquisition Date	2023-10-31T19:49:35
Modification Date	2023-10-31T19:49:34
Spectrometer Frequency	100.63
Spectral Width	24038.5
Lowest Frequency	-1944.5
Nucleus	13C
Acquired Size	32768
Spectral Size	65536

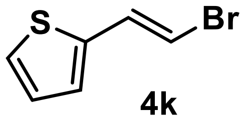

4k

Parameter	Value
Title	C3-29.1.fid
Origin	Bruker BioSpin GmbH
Owner	nmrsu
Instrument	spect
Solvent	CDCl3
Temperature	298.0
Pulse Sequence	zg30
Experiment	1D
Number of Scans	16
Receiver Gain	180.2
Relaxation Delay	1.0000
Pulse Width	11.4900
Acquisition Time	4.0894
Acquisition Date	2023-10-21T14:56:15
Modification Date	2023-10-21T14:56:16
Spectrometer Frequency	400.15
Spectral Width	8012.8
Lowest Frequency	-1545.5
Nucleus	1H
Acquired Size	32768
Spectral Size	65536

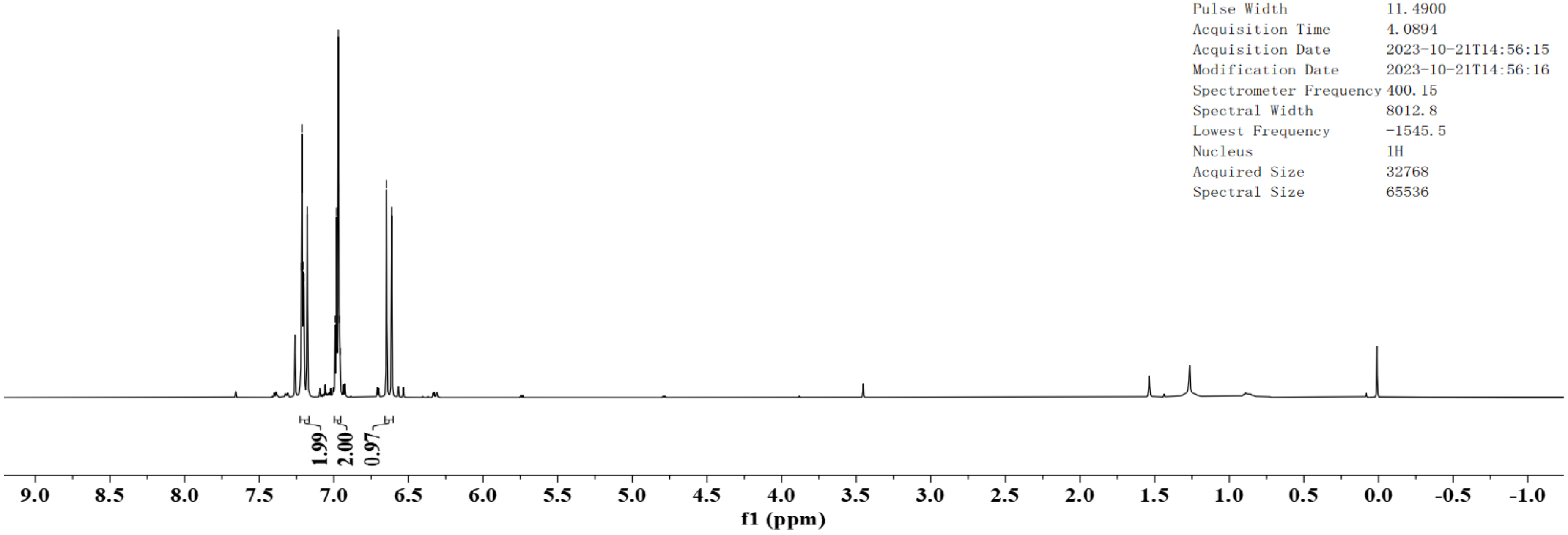

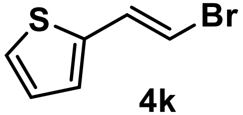

4k

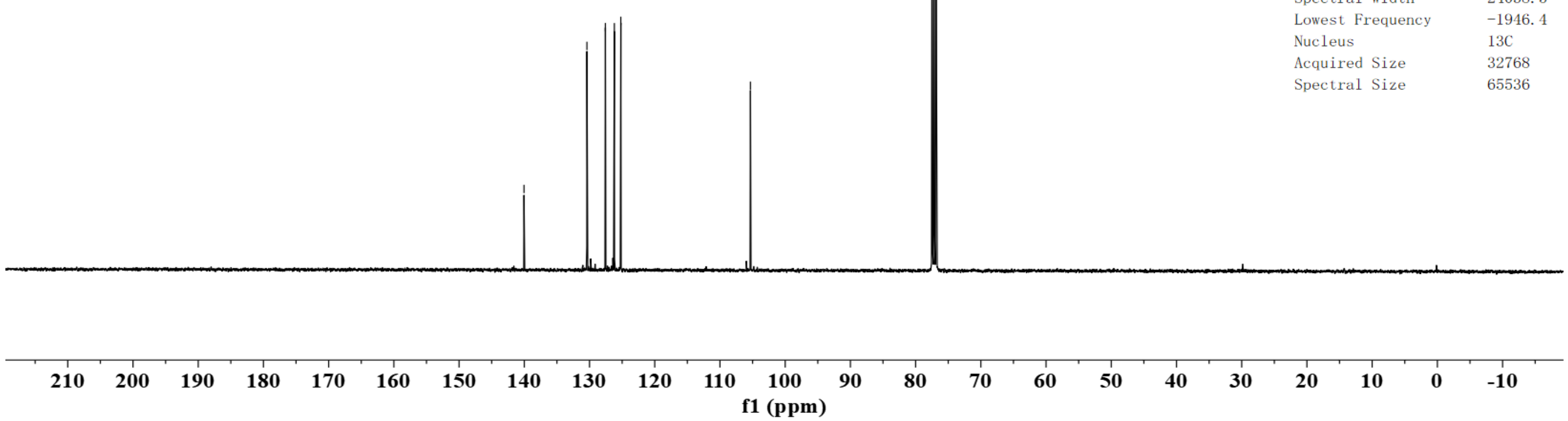

Parameter	Value
Title	C3-29-C.1.fid
Origin	Bruker BioSpin GmbH
Owner	nmrsu
Instrument	spect
Solvent	CDCl3
Temperature	298.0
Pulse Sequence	zgpg30
Experiment	1D
Number of Scans	1024
Receiver Gain	202.1
Relaxation Delay	2.0000
Pulse Width	7.0000
Acquisition Time	1.3631
Acquisition Date	2023-10-25T01:55:25
Modification Date	2023-10-25T01:55:24
Spectrometer Frequency	100.63
Spectral Width	24038.5
Lowest Frequency	-1946.4
Nucleus	13C
Acquired Size	32768
Spectral Size	65536

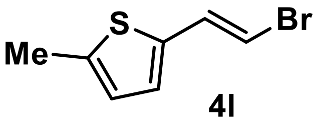

4l

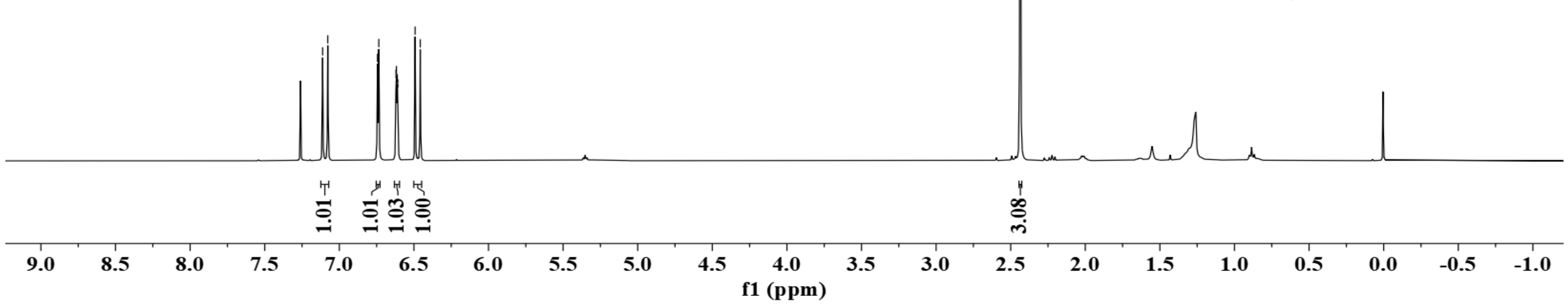

Parameter	Value
Title	C6-10.1.fid
Origin	Bruker BioSpin GmbH
Owner	nmrsu
Instrument	spect
Solvent	CDCl3
Temperature	298.0
Pulse Sequence	zg30
Experiment	1D
Number of Scans	16
Receiver Gain	202.1
Relaxation Delay	1.0000
Pulse Width	11.4900
Acquisition Time	4.0894
Acquisition Date	2023-10-30T15:13:52
Modification Date	2023-10-30T15:13:52
Spectrometer Frequency	400.15
Spectral Width	8012.8
Lowest Frequency	-1545.4
Nucleus	1H
Acquired Size	32768
Spectral Size	65536

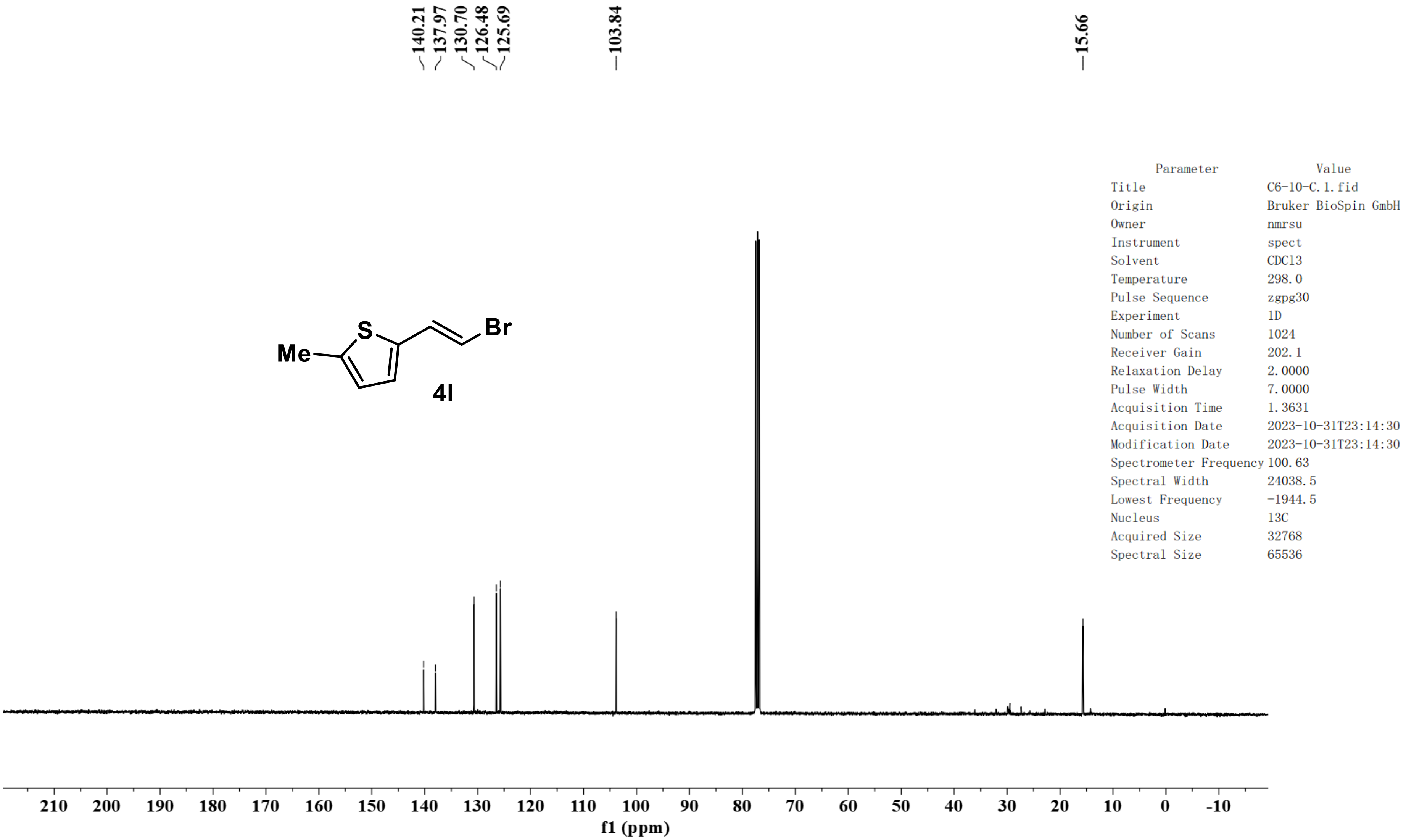

140.21
137.97
130.70
126.48
125.69
103.84
15.66
S
Br
Me
4l
Parameter Value
Title C6-10-C.1.fid
Origin Bruker BioSpin GmbH
Owner nmrsu
Instrument spect
Solvent CDCl3
Temperature 298.0
Pulse Sequence zgpg30
Experiment 1D
Number of Scans 1024
Receiver Gain 202.1
Relaxation Delay 2.0000
Pulse Width 7.0000
Acquisition Time 1.3631
Acquisition Date 2023-10-31T23:14:30
Modification Date 2023-10-31T23:14:30
Spectrometer Frequency 100.63
Spectral Width 24038.5
Lowest Frequency -1944.5
Nucleus 13C
Acquired Size 32768
Spectral Size 65536
210 200 190 180 170 160 150 140 130 120 110 100 90 80 70 60 50 40 30 20 10 0 -10
f1 (ppm)

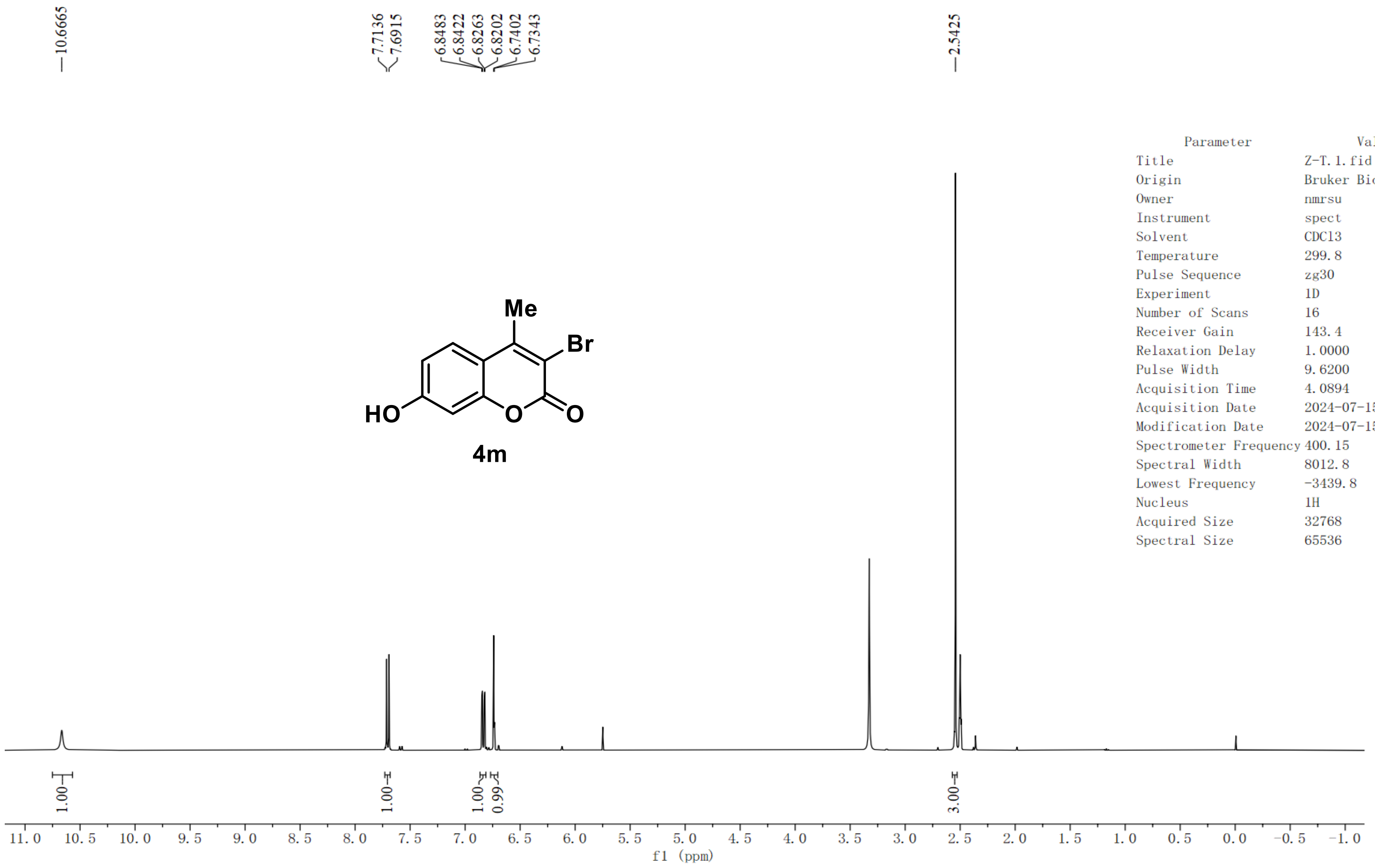

Parameter	Value
Title	Z-T.1.fid
Origin	Bruker BioSpin GmbH
Owner	nmrsu
Instrument	spect
Solvent	CDCl3
Temperature	299.8
Pulse Sequence	zg30
Experiment	1D
Number of Scans	16
Receiver Gain	143.4
Relaxation Delay	1.0000
Pulse Width	9.6200
Acquisition Time	4.0894
Acquisition Date	2024-07-15T12:26:48
Modification Date	2024-07-15T12:26:50
Spectrometer Frequency	400.15
Spectral Width	8012.8
Lowest Frequency	-3439.8
Nucleus	1H
Acquired Size	32768
Spectral Size	65536

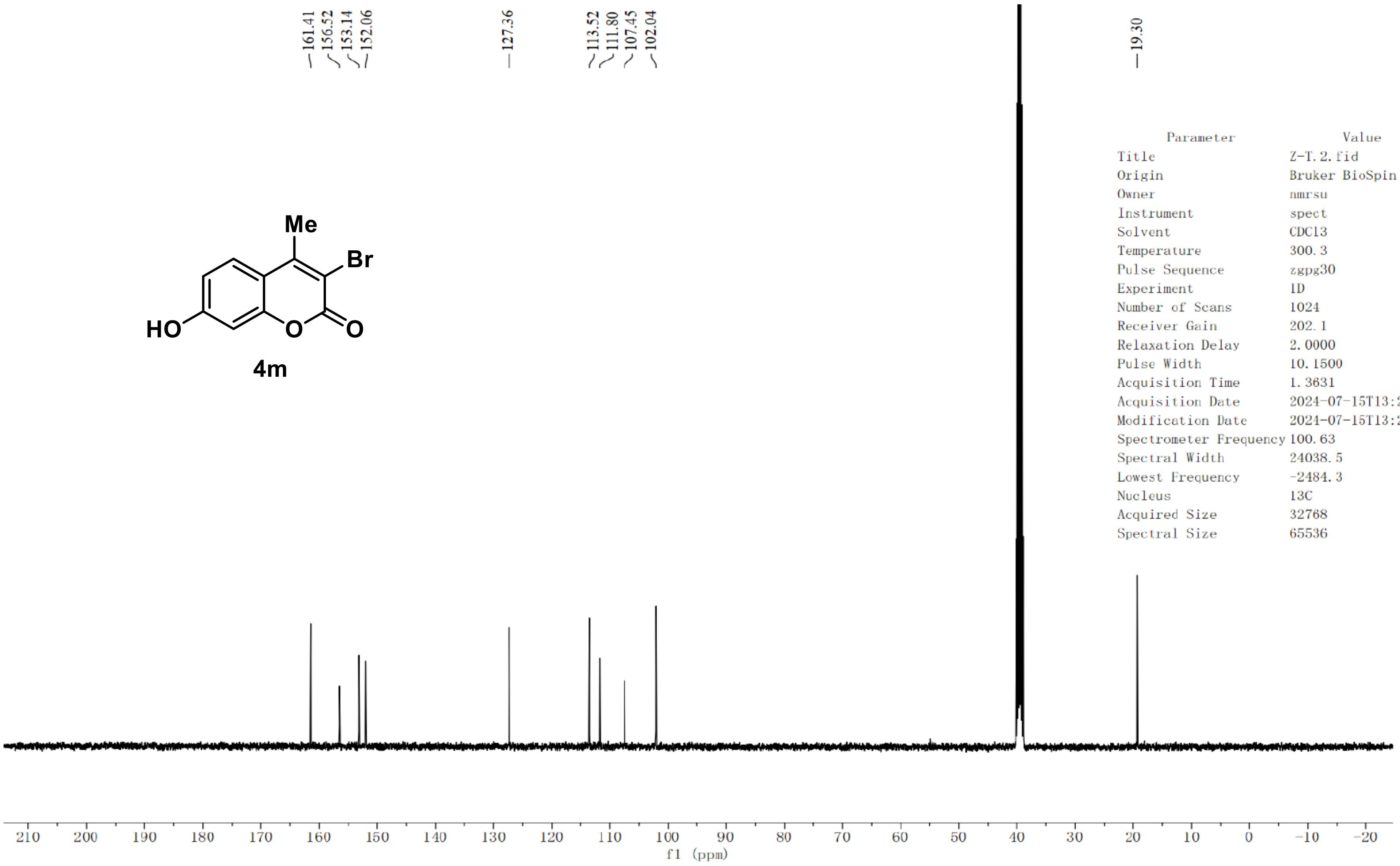

Parameter	Value
Title	Z-T.2.fid
Origin	Bruker BioSpin GmbH
Owner	nmrsu
Instrument	spect
Solvent	CDCl3
Temperature	300.3
Pulse Sequence	zgpg30
Experiment	1D
Number of Scans	1024
Receiver Gain	202.1
Relaxation Delay	2.0000
Pulse Width	10.1500
Acquisition Time	1.3631
Acquisition Date	2024-07-15T13:26:29
Modification Date	2024-07-15T13:26:30
Spectrometer Frequency	100.63
Spectral Width	24038.5
Lowest Frequency	-2484.3
Nucleus	13C
Acquired Size	32768
Spectral Size	65536

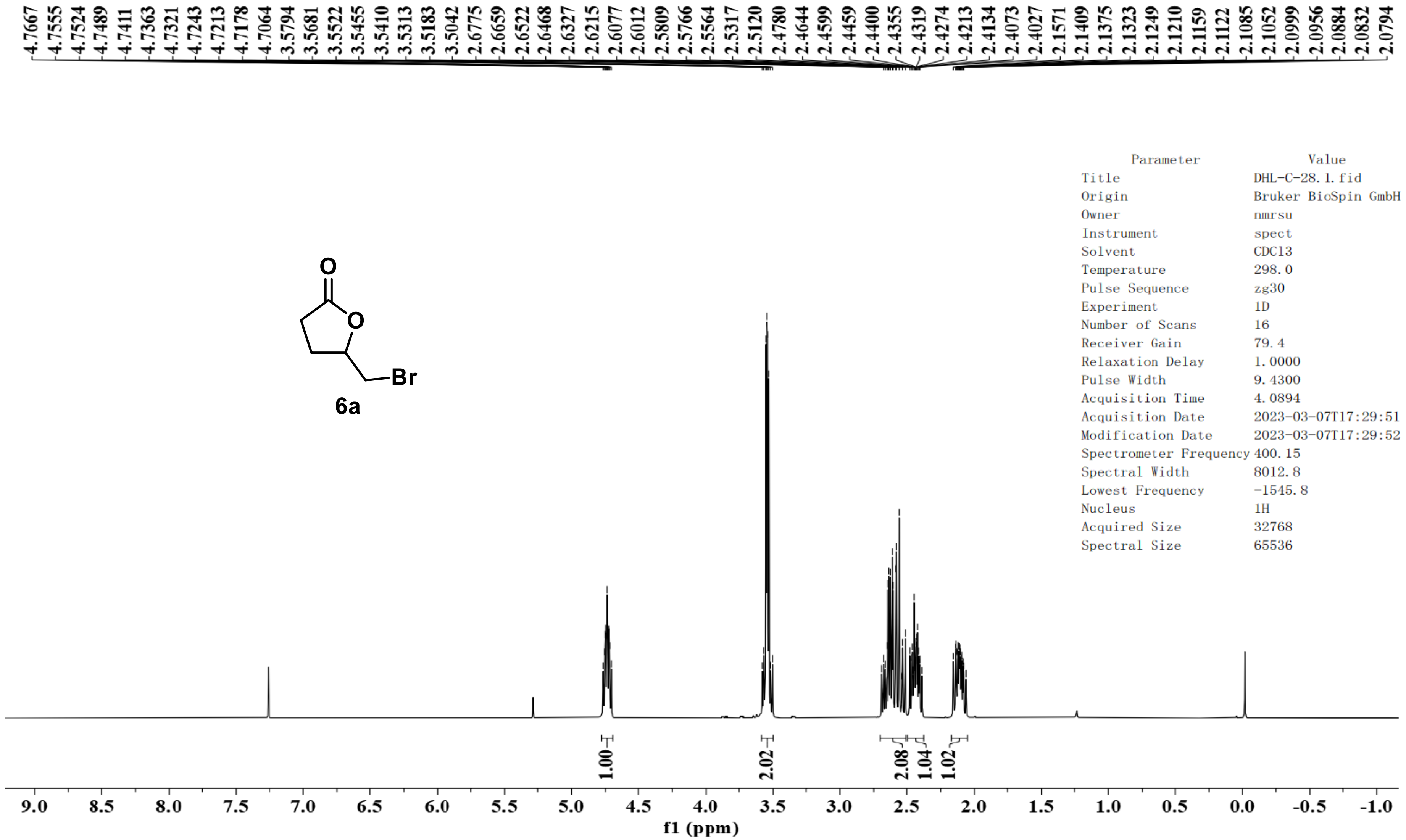

4.7667 4.7555 4.7524 4.7489 4.7411 4.7363 4.7321 4.7243 4.7213 4.7178 4.7064 3.5794 3.5681 3.5522 3.5455 3.5410 3.5313 3.5183 3.5042 2.6775 2.6659 2.6522 2.6468 2.6327 2.6215 2.6077 2.6012 2.5809 2.5766 2.5564 2.5317 2.5120 2.4780 2.4644 2.4599 2.4459 2.4400 2.4355 2.4319 2.4274 2.4213 2.4134 2.4073 2.4027 2.1571 2.1409 2.1375 2.1323 2.1249 2.1210 2.1159 2.1122 2.1085 2.1052 2.0999 2.0956 2.0884 2.0832 2.0794
O
O
Br
6a
Parameter Value
Title DHL-C-28.1.fid
Origin Bruker BioSpin GmbH
Owner nmrsu
Instrument spect
Solvent CDCl3
Temperature 298.0
Pulse Sequence zg30
Experiment 1D
Number of Scans 16
Receiver Gain 79.4
Relaxation Delay 1.0000
Pulse Width 9.4300
Acquisition Time 4.0894
Acquisition Date 2023-03-07T17:29:51
Modification Date 2023-03-07T17:29:52
Spectrometer Frequency 400.15
Spectral Width 8012.8
Lowest Frequency -1545.8
Nucleus 1H
Acquired Size 32768
Spectral Size 65536
1.00
2.02
2.08
1.04
1.02
9.0 8.5 8.0 7.5 7.0 6.5 6.0 5.5 5.0 4.5 4.0 3.5 3.0 2.5 2.0 1.5 1.0 0.5 0.0 -0.5 -1.0
f1 (ppm)

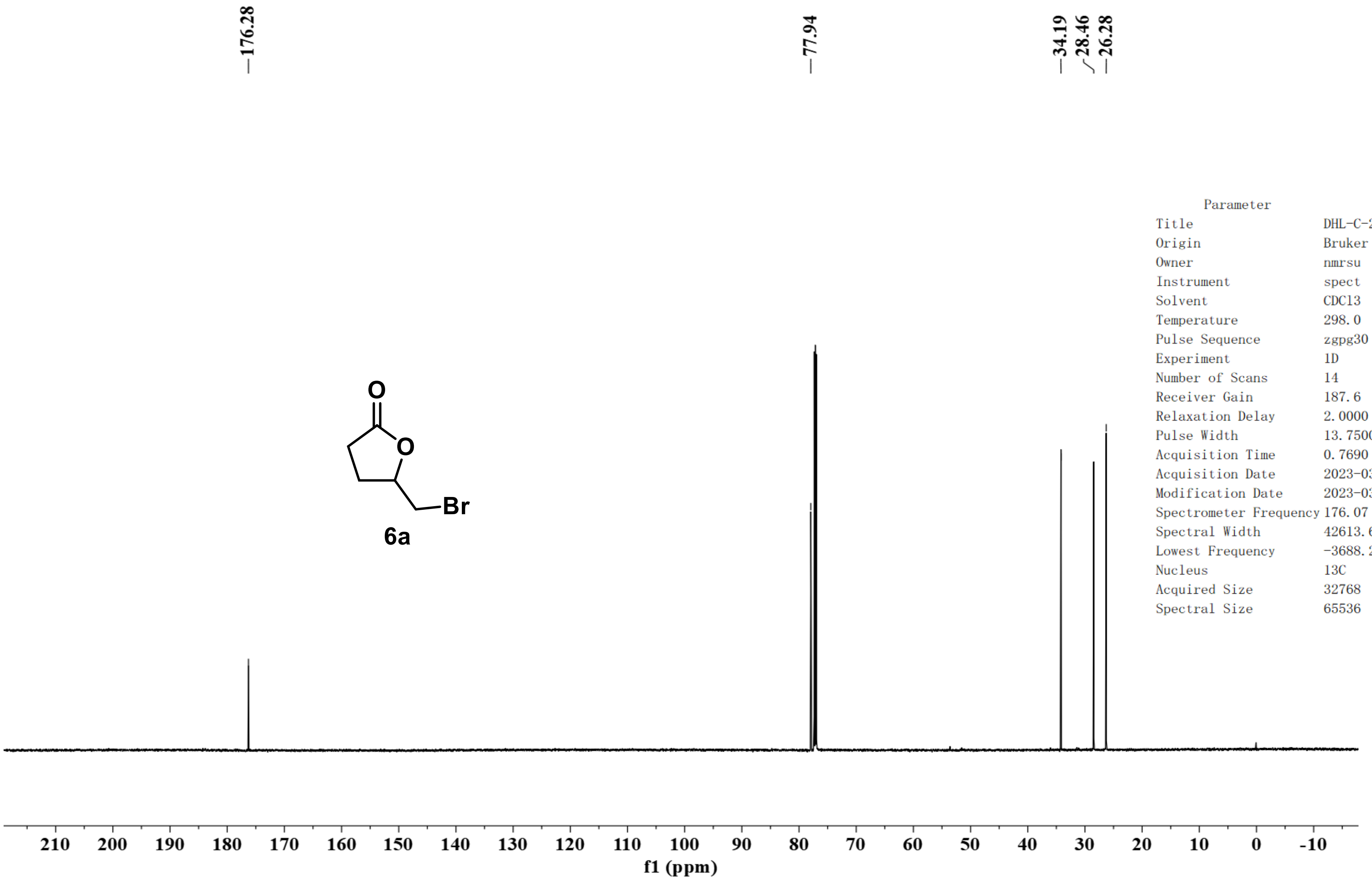

Parameter	Value
Title	DHL-C-27.2.fid
Origin	Bruker BioSpin GmbH
Owner	nmrsu
Instrument	spect
Solvent	CDCl3
Temperature	298.0
Pulse Sequence	zgpg30
Experiment	1D
Number of Scans	14
Receiver Gain	187.6
Relaxation Delay	2.0000
Pulse Width	13.7500
Acquisition Time	0.7690
Acquisition Date	2023-03-09T14:43:36
Modification Date	2023-03-09T14:43:38
Spectrometer Frequency	176.07
Spectral Width	42613.6
Lowest Frequency	-3688.2
Nucleus	13C
Acquired Size	32768
Spectral Size	65536

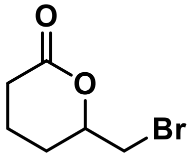

6b

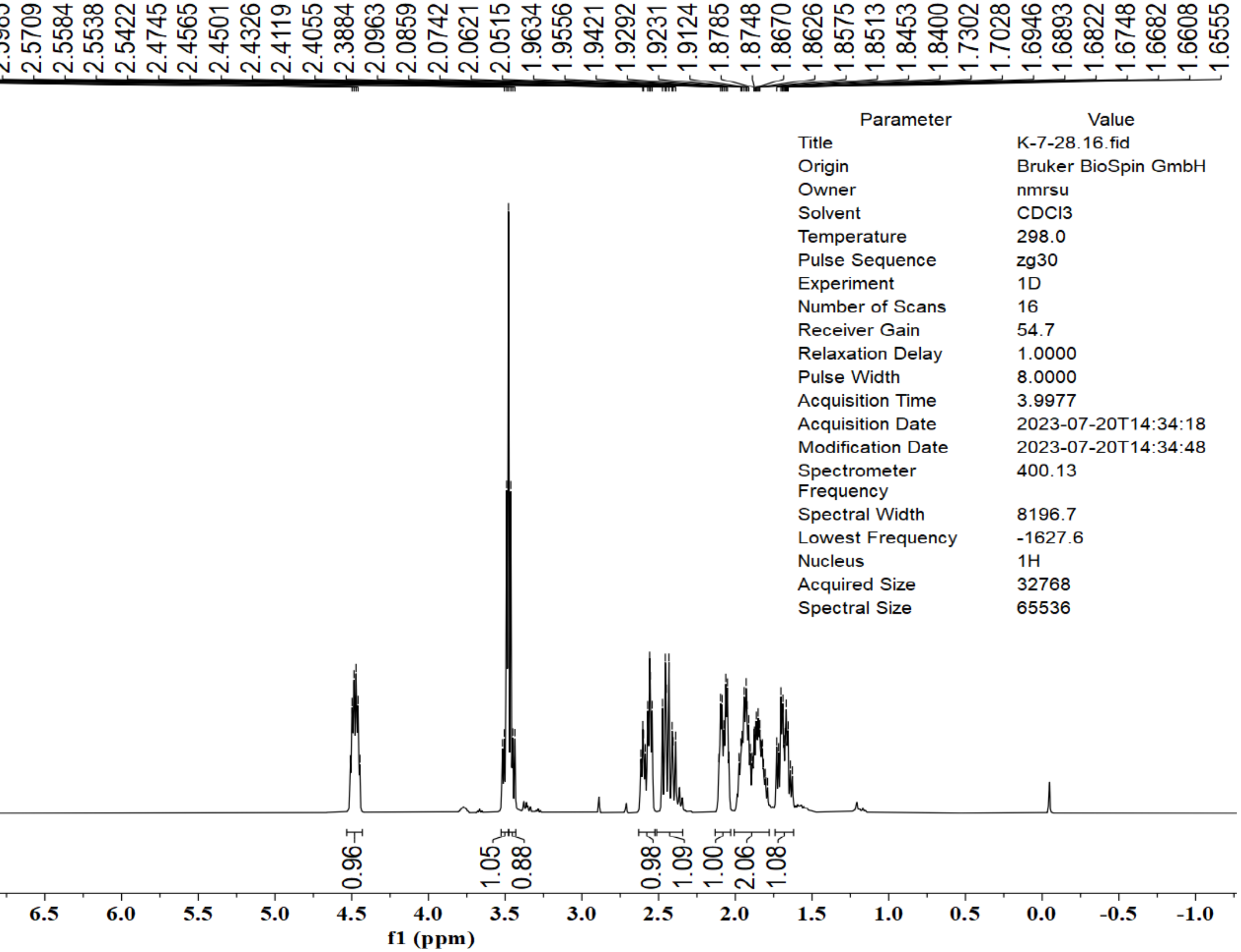

Parameter	Value
Title	K-7-28.16.fid
Origin	Bruker BioSpin GmbH
Owner	nmrsu
Solvent	CDCl3
Temperature	298.0
Pulse Sequence	zg30
Experiment	1D
Number of Scans	16
Receiver Gain	54.7
Relaxation Delay	1.0000
Pulse Width	8.0000
Acquisition Time	3.9977
Acquisition Date	2023-07-20T14:34:18
Modification Date	2023-07-20T14:34:48
Spectrometer Frequency	400.13
Spectral Width	8196.7
Lowest Frequency	-1627.6
Nucleus	1H
Acquired Size	32768
Spectral Size	65536

Parameter	Value
Title	K-7-28.21.fid
Origin	Bruker BioSpin GmbH
Owner	nmrsu
Solvent	CDCl3
Temperature	298.0
Pulse Sequence	zgpg30
Experiment	1D
Number of Scans	1024
Receiver Gain	101.0
Relaxation Delay	2.0000
Pulse Width	8.0000
Acquisition Time	1.3763
Acquisition Date	2023-07-21T20:18:23
Modification Date	2023-07-21T20:18:55
Spectrometer Frequency	100.62
Spectral Width	23809.5
Lowest Frequency	-1843.5
Nucleus	13C
Acquired Size	32768
Spectral Size	65536

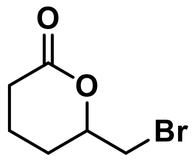

6b

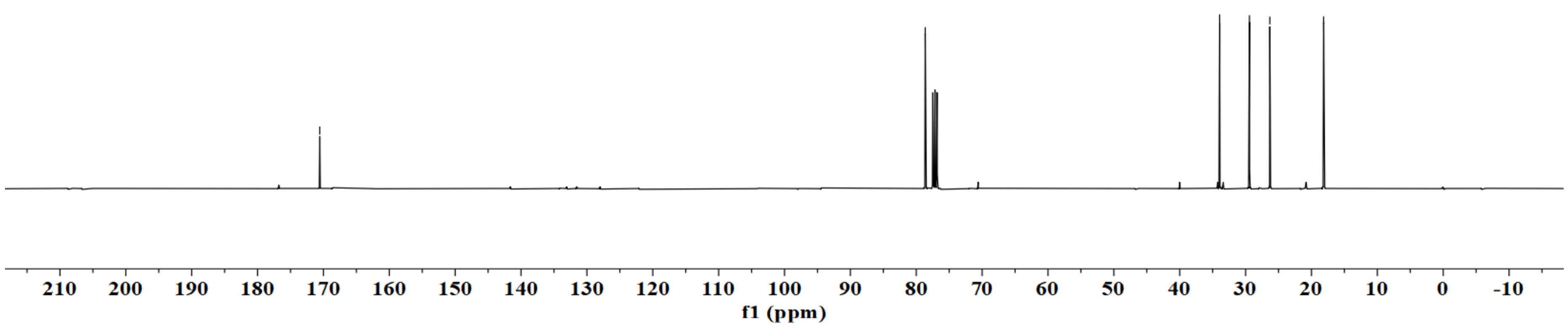

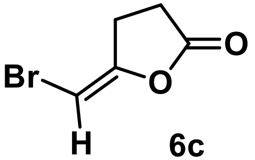

6c

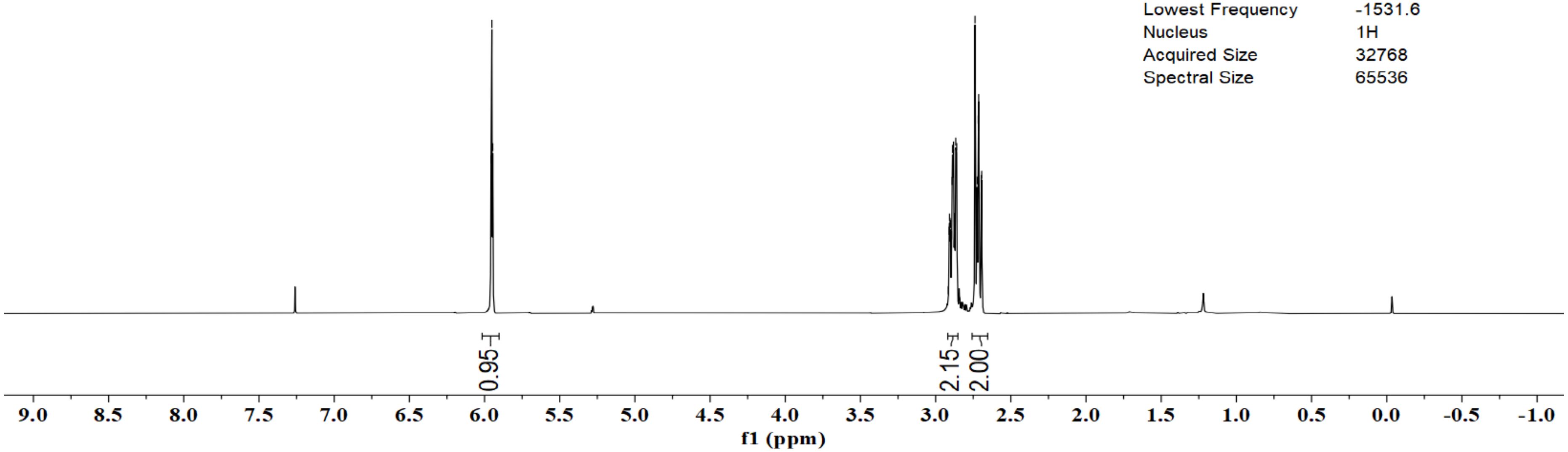

Parameter	Value
Title	K-4-48.1.fid
Origin	Bruker BioSpin GmbH
Owner	nmrsu
Solvent	CDCl3
Temperature	298.0
Pulse Sequence	zg30
Experiment	1D
Number of Scans	16
Receiver Gain	30.5
Relaxation Delay	1.0000
Pulse Width	9.4300
Acquisition Time	4.0894
Acquisition Date	2023-05-11T10:53:17
Modification Date	2023-05-11T10:53:18
Spectrometer Frequency	400.15
Spectral Width	8012.8
Lowest Frequency	-1531.6
Nucleus	1H
Acquired Size	32768
Spectral Size	65536

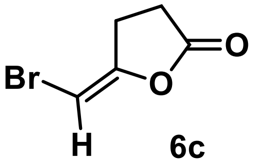

6c

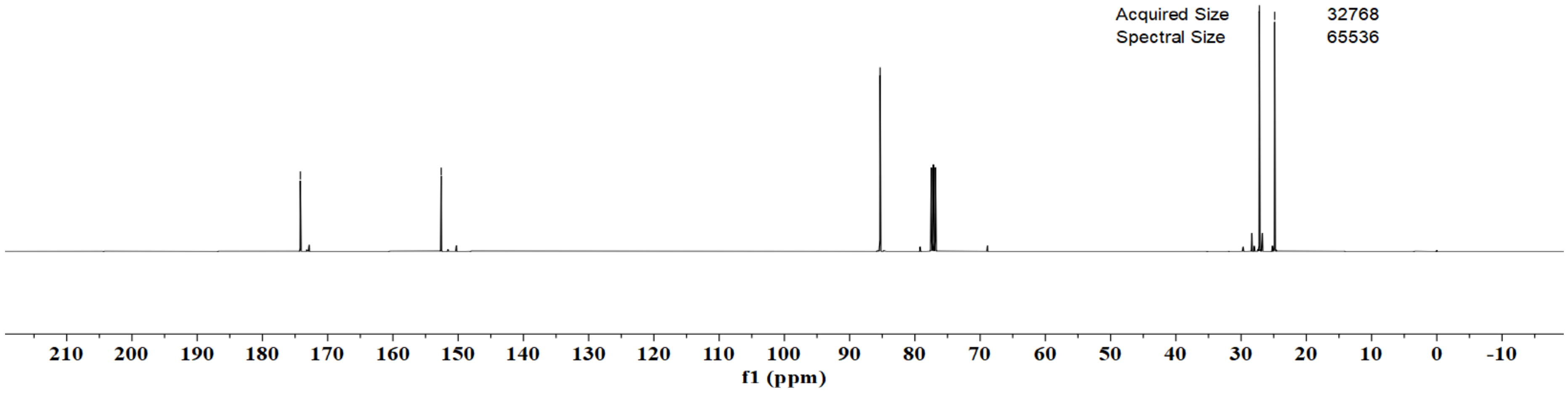

Parameter	Value
Title	K-4-48.2.fid
Origin	Bruker BioSpin GmbH
Owner	nmrsu
Solvent	CDCl3
Temperature	298.0
Pulse Sequence	zgpg30
Experiment	1D
Number of Scans	1024
Receiver Gain	202.1
Relaxation Delay	2.0000
Pulse Width	9.3700
Acquisition Time	1.3631
Acquisition Date	2023-05-15T22:29:03
Modification Date	2023-05-15T22:29:04
Spectrometer Frequency	100.63
Spectral Width	24038.5
Lowest Frequency	-1957.5
Nucleus	13C
Acquired Size	32768
Spectral Size	65536

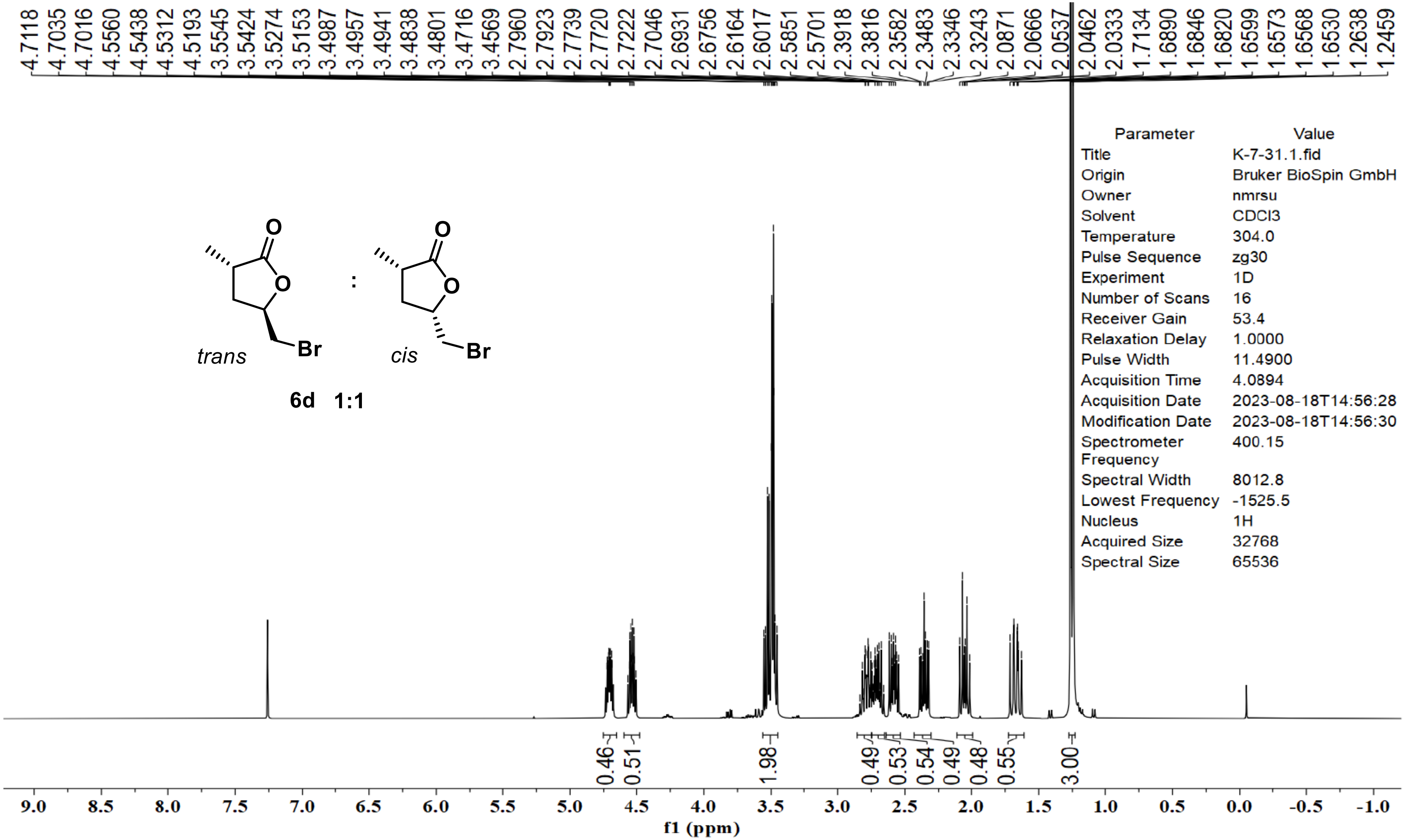
4.7118
4.7035
4.7016
4.5560
4.5438
4.5312
4.5193
3.5545
3.5424
3.5274
3.5153
3.4987
3.4957
3.4941
3.4838
3.4801
3.4716
3.4569
2.7960
2.7923
2.7739
2.7720
2.7222
2.7046
2.6931
2.6756
2.6164
2.6017
2.5851
2.5701
2.3918
2.3816
2.3582
2.3483
2.3346
2.3243
2.0871
2.0666
2.0537
2.0462
2.0333
1.7134
1.6890
1.6846
1.6820
1.6599
1.6573
1.6568
1.6530
1.2638
1.2459
Parameter Value
Title K-7-31.1.fid
Origin Bruker BioSpin GmbH
Owner nmrsu
Solvent CDCl3
Temperature 304.0
Pulse Sequence zg30
Experiment 1D
Number of Scans 16
Receiver Gain 53.4
Relaxation Delay 1.0000
Pulse Width 11.4900
Acquisition Time 4.0894
Acquisition Date 2023-08-18T14:56:28
Modification Date 2023-08-18T14:56:30
Spectrometer Frequency 400.15
Spectral Width 8012.8
Lowest Frequency -1525.5
Nucleus 1H
Acquired Size 32768
Spectral Size 65536
O
O
Br
trans
:
O
O
Br
cis
6d 1:1
0.46
0.51
1.98
0.49
0.53
0.54
0.49
0.48
0.55
3.00
9.0 8.5 8.0 7.5 7.0 6.5 6.0 5.5 5.0 4.5 4.0 3.5 3.0 2.5 2.0 1.5 1.0 0.5 0.0 -0.5 -1.0
f1 (ppm)

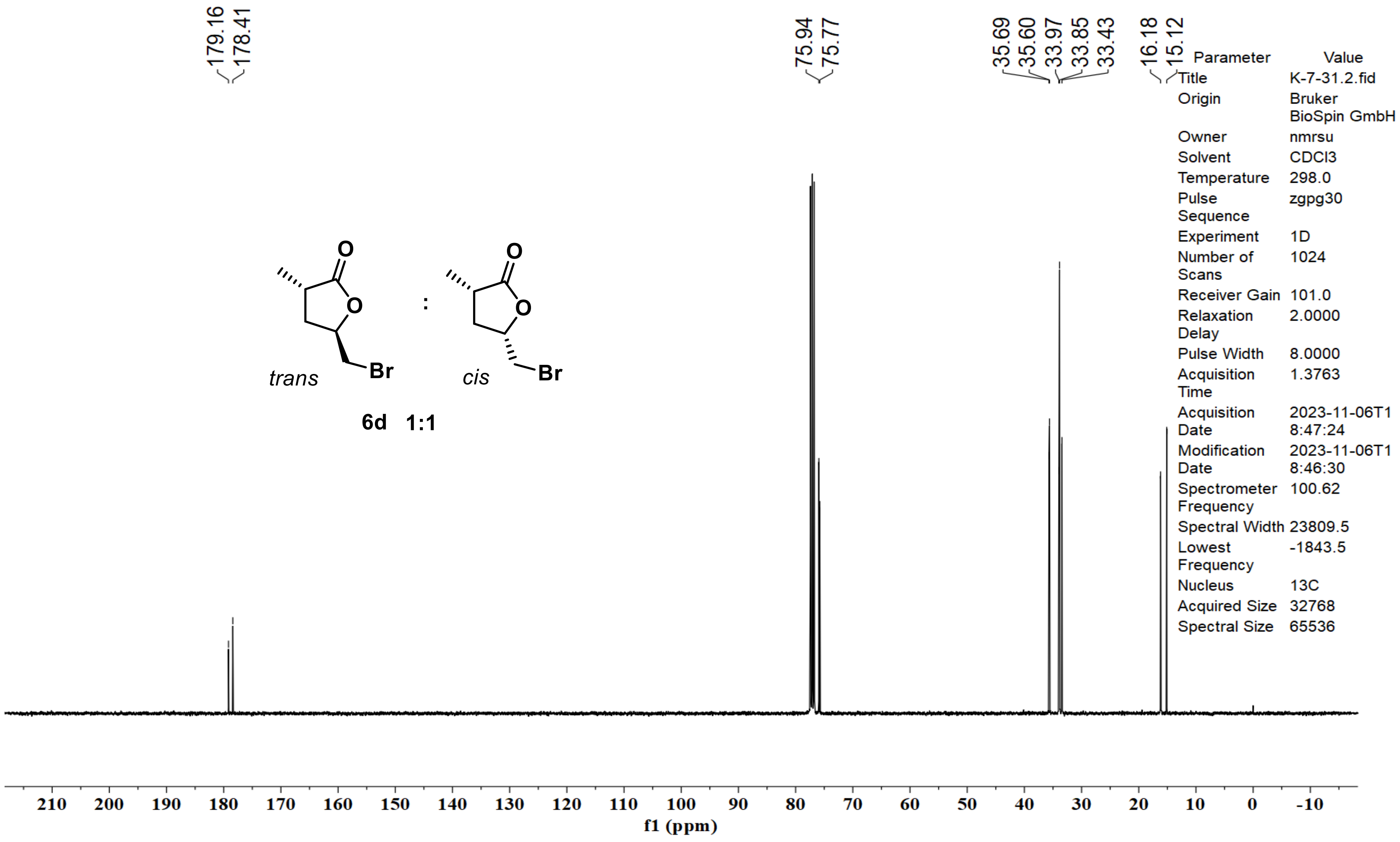
179.16
178.41
75.94
75.77
35.69
35.60
33.97
33.85
33.43
16.18
15.12
trans
cis
6d 1:1
Parameter Value
Title K-7-31.2.fid
Origin Bruker BioSpin GmbH
Owner nmrsu
Solvent CDCl3
Temperature 298.0
Pulse Sequence zgpg30
Experiment 1D
Number of Scans 1024
Receiver Gain 101.0
Relaxation Delay 2.0000
Pulse Width 8.0000
Acquisition Time 1.3763
Acquisition Date 2023-11-06T18:47:24
Modification Date 2023-11-06T18:46:30
Spectrometer Frequency 100.62
Spectral Width 23809.5
Lowest Frequency -1843.5
Nucleus 13C
Acquired Size 32768
Spectral Size 65536
210 200 190 180 170 160 150 140 130 120 110 100 90 80 70 60 50 40 30 20 10 0 -10
f1 (ppm)

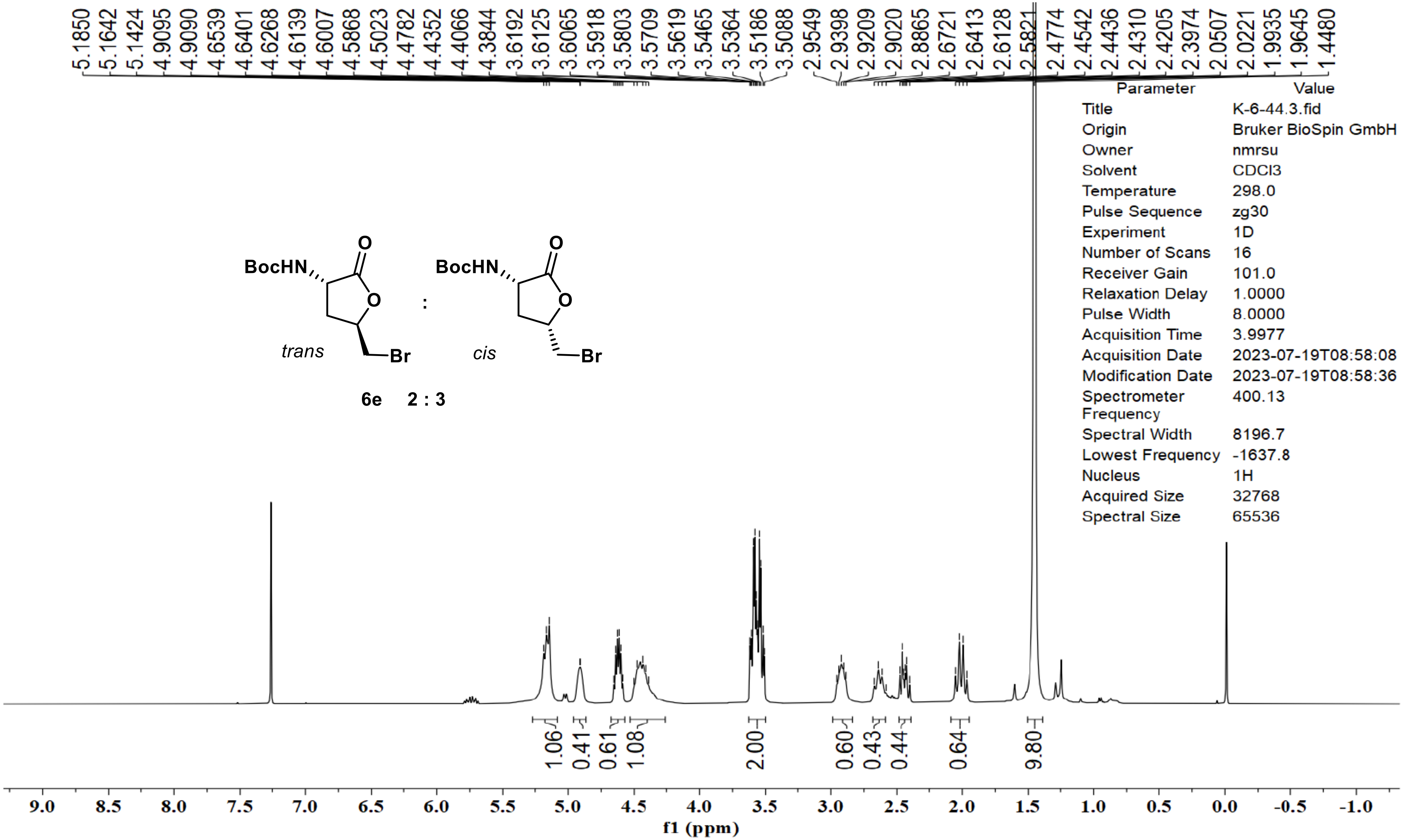

Parameter	Value
Title	K-6-44.3.fid
Origin	Bruker BioSpin GmbH
Owner	nmrsu
Solvent	CDCl3
Temperature	298.0
Pulse Sequence	zg30
Experiment	1D
Number of Scans	16
Receiver Gain	101.0
Relaxation Delay	1.0000
Pulse Width	8.0000
Acquisition Time	3.9977
Acquisition Date	2023-07-19T08:58:08
Modification Date	2023-07-19T08:58:36
Spectrometer Frequency	400.13
Spectral Width	8196.7
Lowest Frequency	-1637.8
Nucleus	1H
Acquired Size	32768
Spectral Size	65536

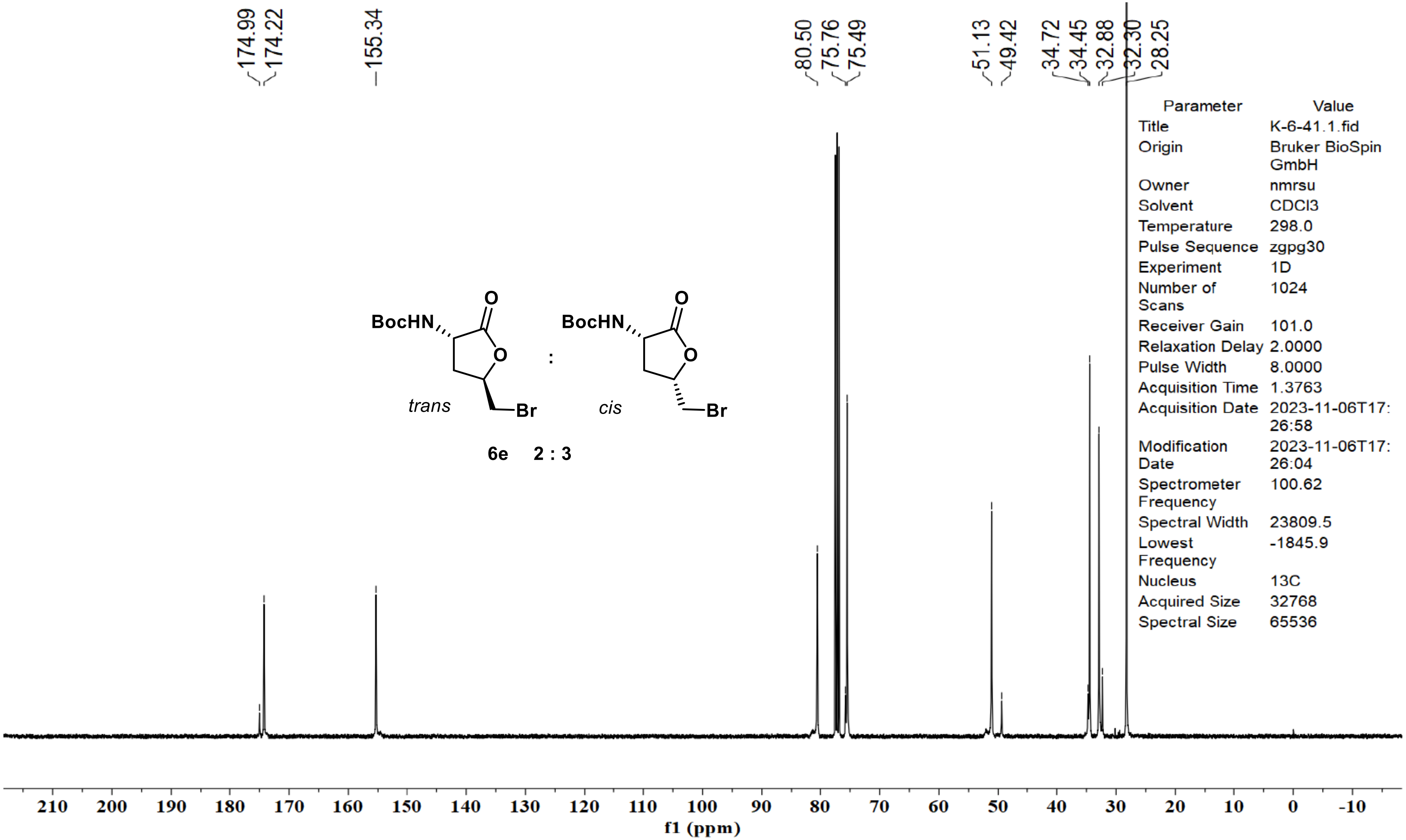

Parameter	Value
Title	K-6-41.1.fid
Origin	Bruker BioSpin GmbH
Owner	nmrsu
Solvent	CDCl3
Temperature	298.0
Pulse Sequence	zgpg30
Experiment	1D
Number of Scans	1024
Receiver Gain	101.0
Relaxation Delay	2.0000
Pulse Width	8.0000
Acquisition Time	1.3763
Acquisition Date	2023-11-06T17:26:58
Modification Date	2023-11-06T17:26:04
Spectrometer Frequency	100.62
Spectral Width	23809.5
Lowest Frequency	-1845.9
Nucleus	13C
Acquired Size	32768
Spectral Size	65536

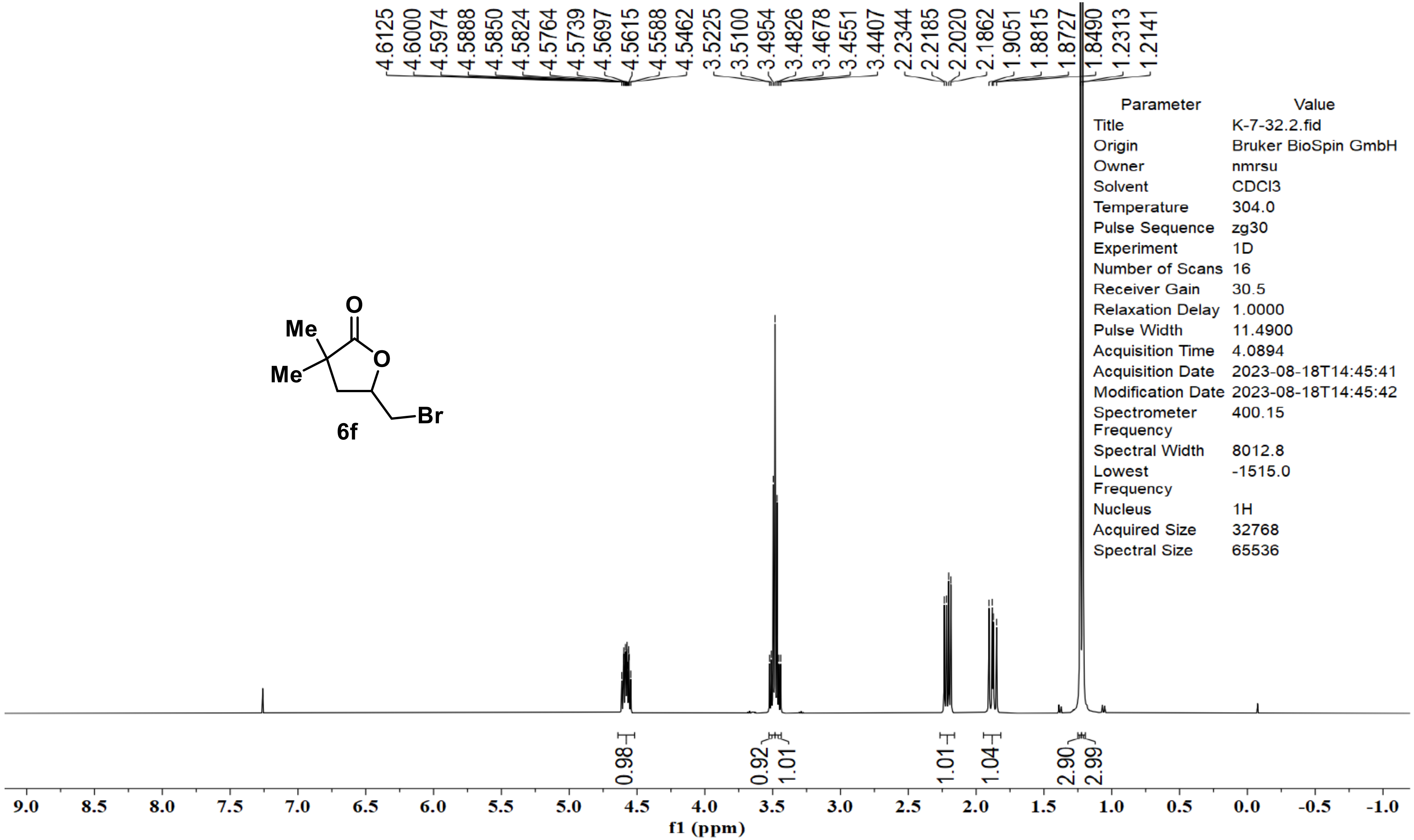
4.6125
4.6000
4.5974
4.5888
4.5850
4.5824
4.5764
4.5739
4.5697
4.5615
4.5588
4.5462
3.5225
3.5100
3.4954
3.4826
3.4678
3.4551
3.4407
2.2344
2.2185
2.2020
2.1862
1.9051
1.8815
1.8727
1.8490
1.2313
1.2141
Parameter Value
Title K-7-32.2.fid
Origin Bruker BioSpin GmbH
Owner nmrsu
Solvent CDCl3
Temperature 304.0
Pulse Sequence zg30
Experiment 1D
Number of Scans 16
Receiver Gain 30.5
Relaxation Delay 1.0000
Pulse Width 11.4900
Acquisition Time 4.0894
Acquisition Date 2023-08-18T14:45:41
Modification Date 2023-08-18T14:45:42
Spectrometer Frequency 400.15
Spectral Width 8012.8
Lowest Frequency -1515.0
Nucleus 1H
Acquired Size 32768
Spectral Size 65536
O
Me
O
Me
Br
6f
0.98
0.92
1.01
1.01
1.04
2.90
2.99
9.0 8.5 8.0 7.5 7.0 6.5 6.0 5.5 5.0 4.5 4.0 3.5 3.0 2.5 2.0 1.5 1.0 0.5 0.0 -0.5 -1.0
f1 (ppm)

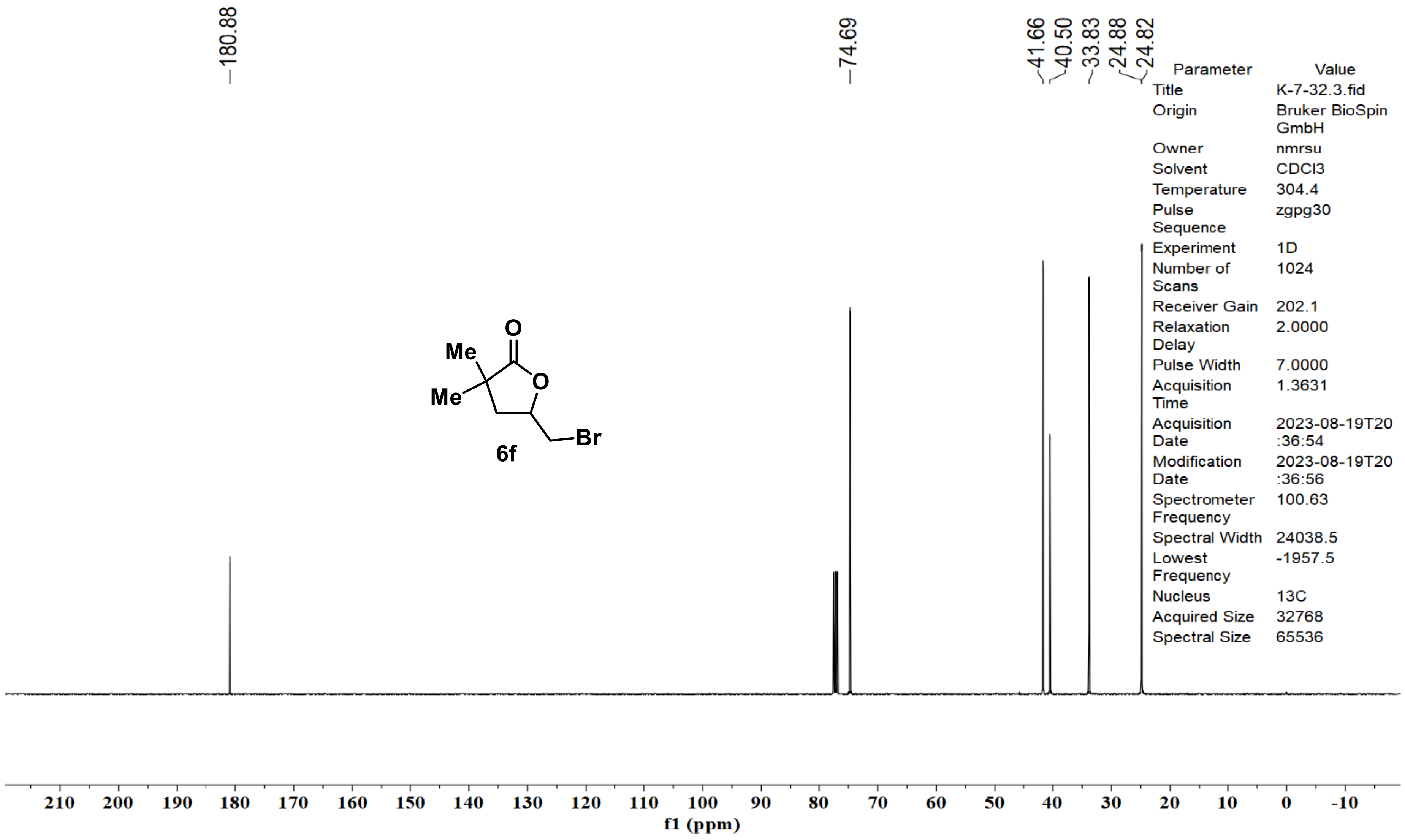

180.88
74.69
41.66
40.50
33.83
24.88
24.82
Me
Me
O
O
Br
6f
Parameter Value
Title K-7-32.3.fid
Origin Bruker BioSpin GmbH
Owner nmrsu
Solvent CDCl3
Temperature 304.4
Pulse Sequence zgpg30
Experiment 1D
Number of Scans 1024
Receiver Gain 202.1
Relaxation Delay 2.0000
Pulse Width 7.0000
Acquisition Time 1.3631
Acquisition Date 2023-08-19T20:36:54
Modification Date 2023-08-19T20:36:56
Spectrometer Frequency 100.63
Spectral Width 24038.5
Lowest Frequency -1957.5
Nucleus 13C
Acquired Size 32768
Spectral Size 65536
210 200 190 180 170 160 150 140 130 120 110 100 90 80 70 60 50 40 30 20 10 0 -10
f1 (ppm)

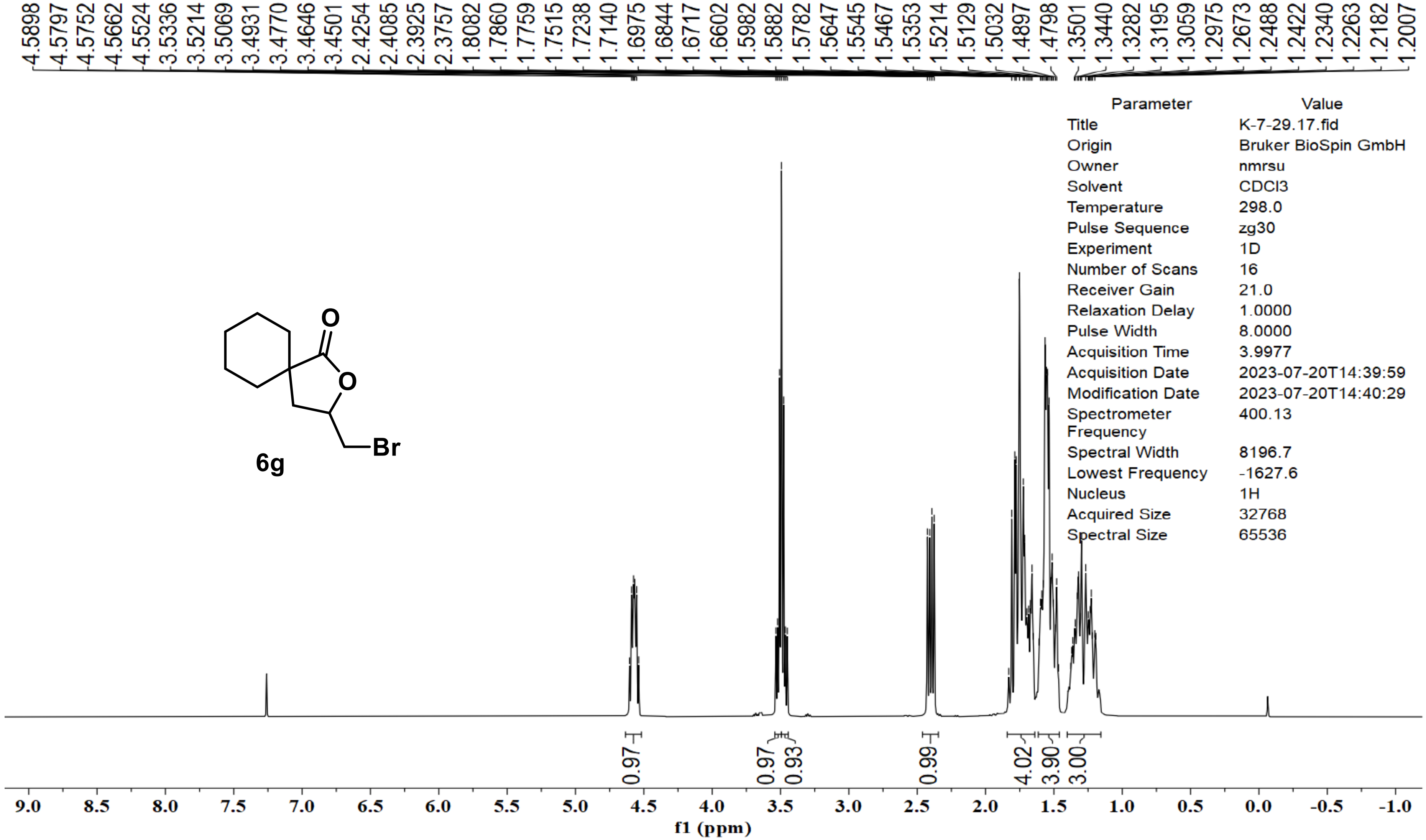

Parameter	Value
Title	K-7-29.17.fid
Origin	Bruker BioSpin GmbH
Owner	nmrsu
Solvent	CDCl3
Temperature	298.0
Pulse Sequence	zg30
Experiment	1D
Number of Scans	16
Receiver Gain	21.0
Relaxation Delay	1.0000
Pulse Width	8.0000
Acquisition Time	3.9977
Acquisition Date	2023-07-20T14:39:59
Modification Date	2023-07-20T14:40:29
Spectrometer Frequency	400.13
Spectral Width	8196.7
Lowest Frequency	-1627.6
Nucleus	1H
Acquired Size	32768
Spectral Size	65536

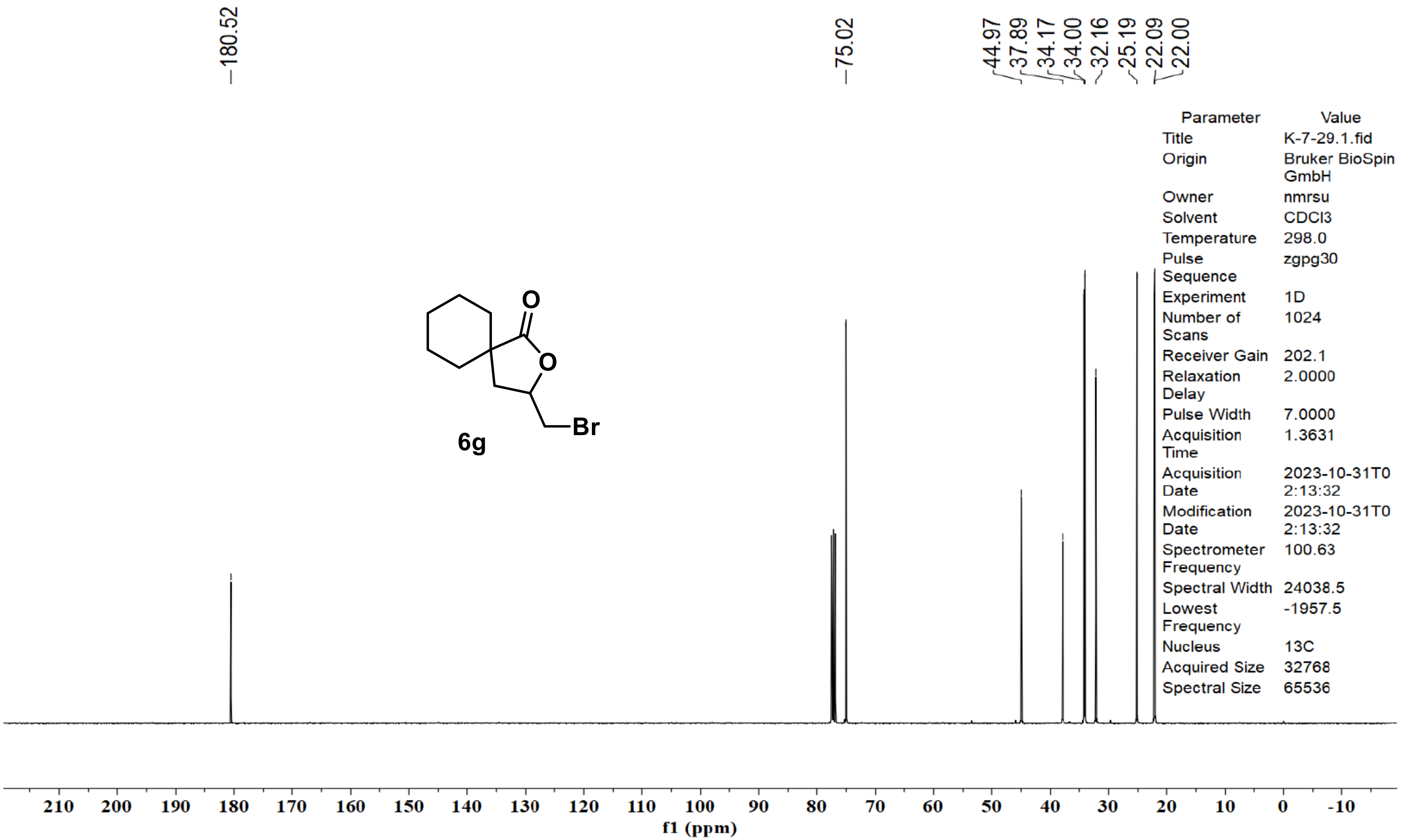

180.52
75.02
44.97
37.89
34.17
34.00
32.16
25.19
22.09
22.00
O
O
Br
6g
Parameter Value
Title K-7-29.1.fid
Origin Bruker BioSpin GmbH
Owner nmrsu
Solvent CDCl3
Temperature 298.0
Pulse Sequence zgpg30
Experiment 1D
Number of Scans 1024
Receiver Gain 202.1
Relaxation Delay 2.0000
Pulse Width 7.0000
Acquisition Time 1.3631
Acquisition Date 2023-10-31T02:13:32
Modification Date 2023-10-31T02:13:32
Spectrometer Frequency 100.63
Spectral Width 24038.5
Lowest Frequency -1957.5
Nucleus 13C
Acquired Size 32768
Spectral Size 65536
210 200 190 180 170 160 150 140 130 120 110 100 90 80 70 60 50 40 30 20 10 0 -10
f1 (ppm)

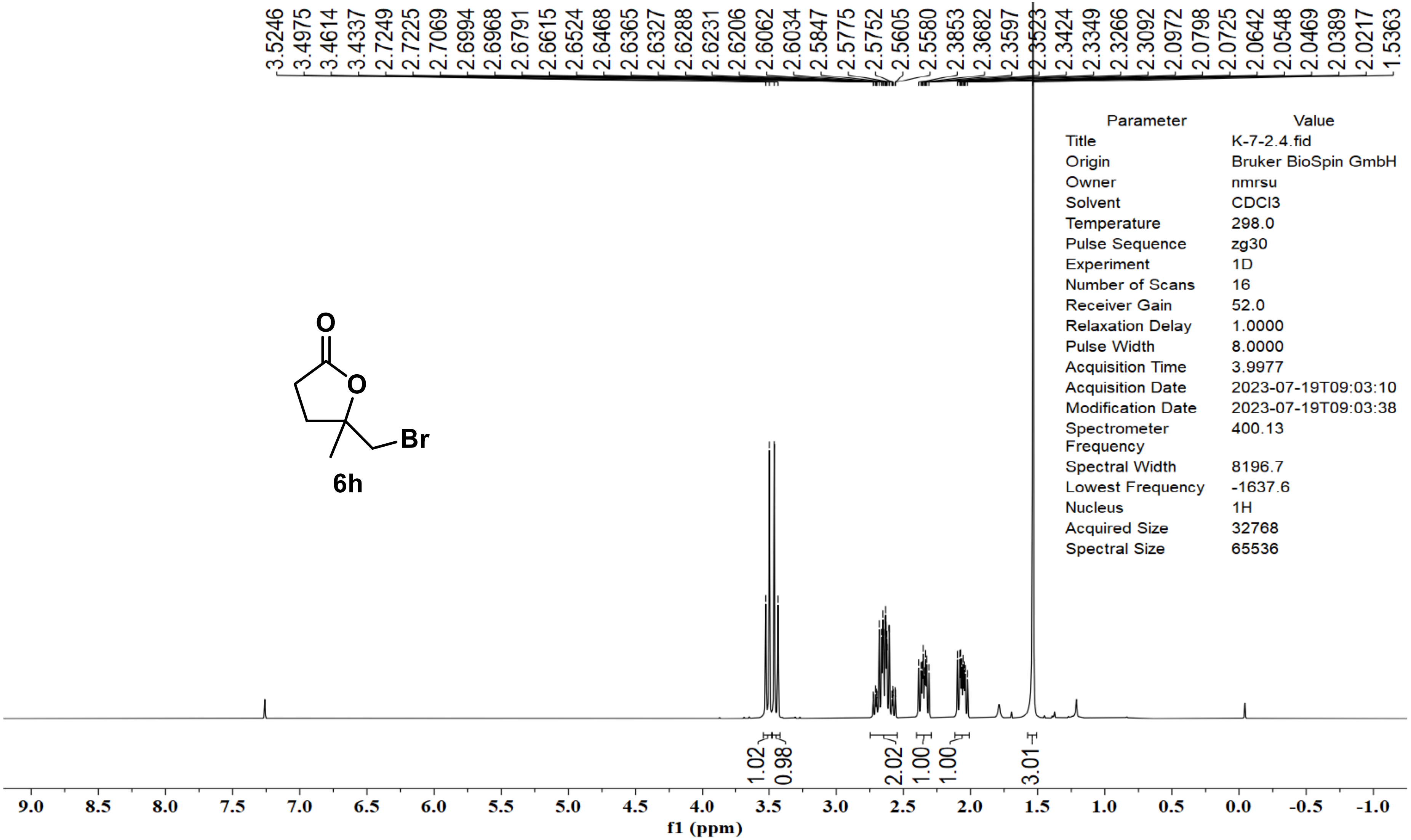

Parameter	Value
Title	K-7-2.4.fid
Origin	Bruker BioSpin GmbH
Owner	nmrsu
Solvent	CDCl3
Temperature	298.0
Pulse Sequence	zg30
Experiment	1D
Number of Scans	16
Receiver Gain	52.0
Relaxation Delay	1.0000
Pulse Width	8.0000
Acquisition Time	3.9977
Acquisition Date	2023-07-19T09:03:10
Modification Date	2023-07-19T09:03:38
Spectrometer Frequency	400.13
Spectral Width	8196.7
Lowest Frequency	-1637.6
Nucleus	1H
Acquired Size	32768
Spectral Size	65536

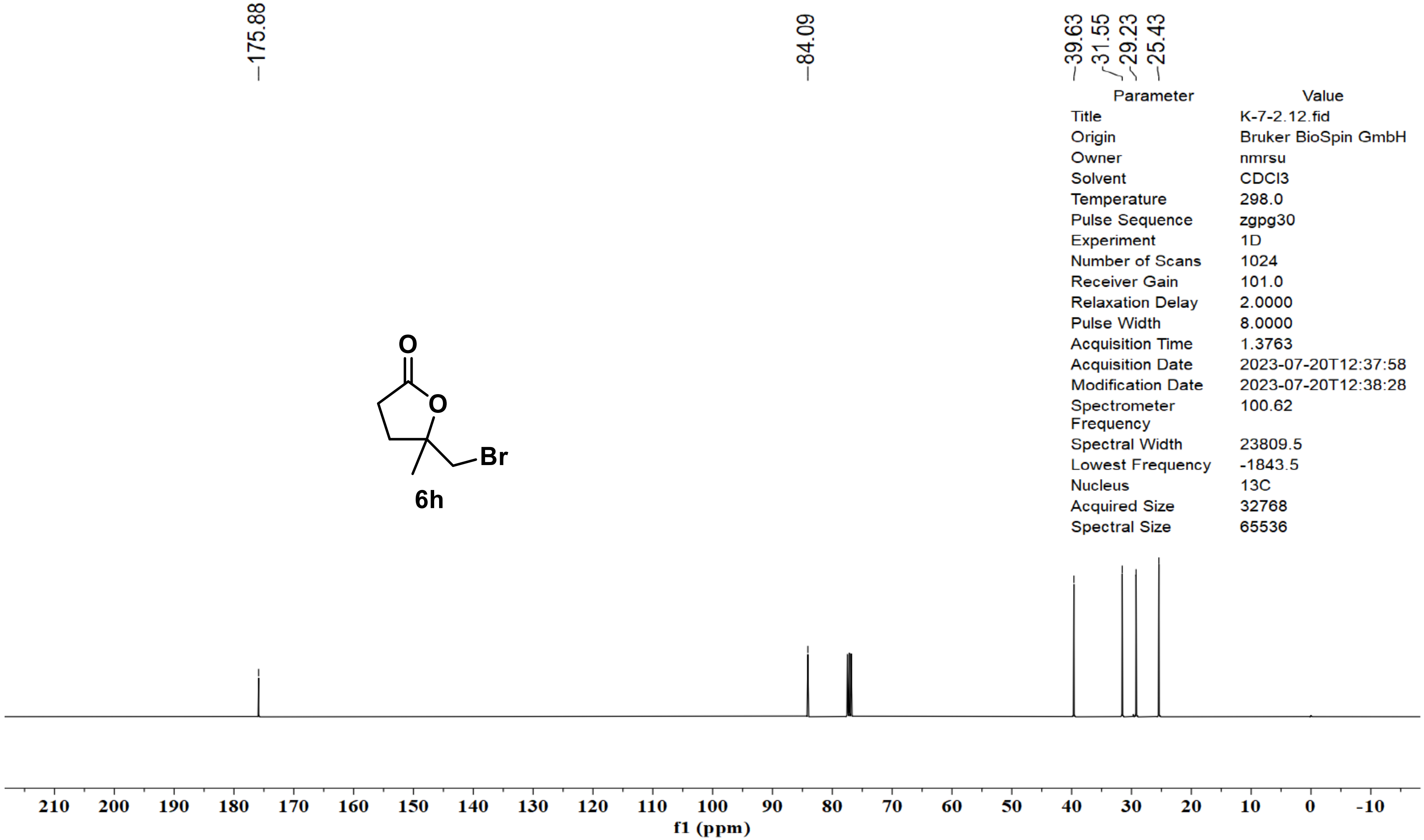

175.88
84.09
39.63
31.55
29.23
25.43
Parameter | Value
Title | K-7-2.12.fid
Origin | Bruker BioSpin GmbH
Owner | nmrsu
Solvent | CDCl3
Temperature | 298.0
Pulse Sequence | zgpg30
Experiment | 1D
Number of Scans | 1024
Receiver Gain | 101.0
Relaxation Delay | 2.0000
Pulse Width | 8.0000
Acquisition Time | 1.3763
Acquisition Date | 2023-07-20T12:37:58
Modification Date | 2023-07-20T12:38:28
Spectrometer Frequency | 100.62
Spectral Width | 23809.5
Lowest Frequency | -1843.5
Nucleus | 13C
Acquired Size | 32768
Spectral Size | 65536
210 200 190 180 170 160 150 140 130 120 110 100 90 80 70 60 50 40 30 20 10 0 -10
f1 (ppm)

6h

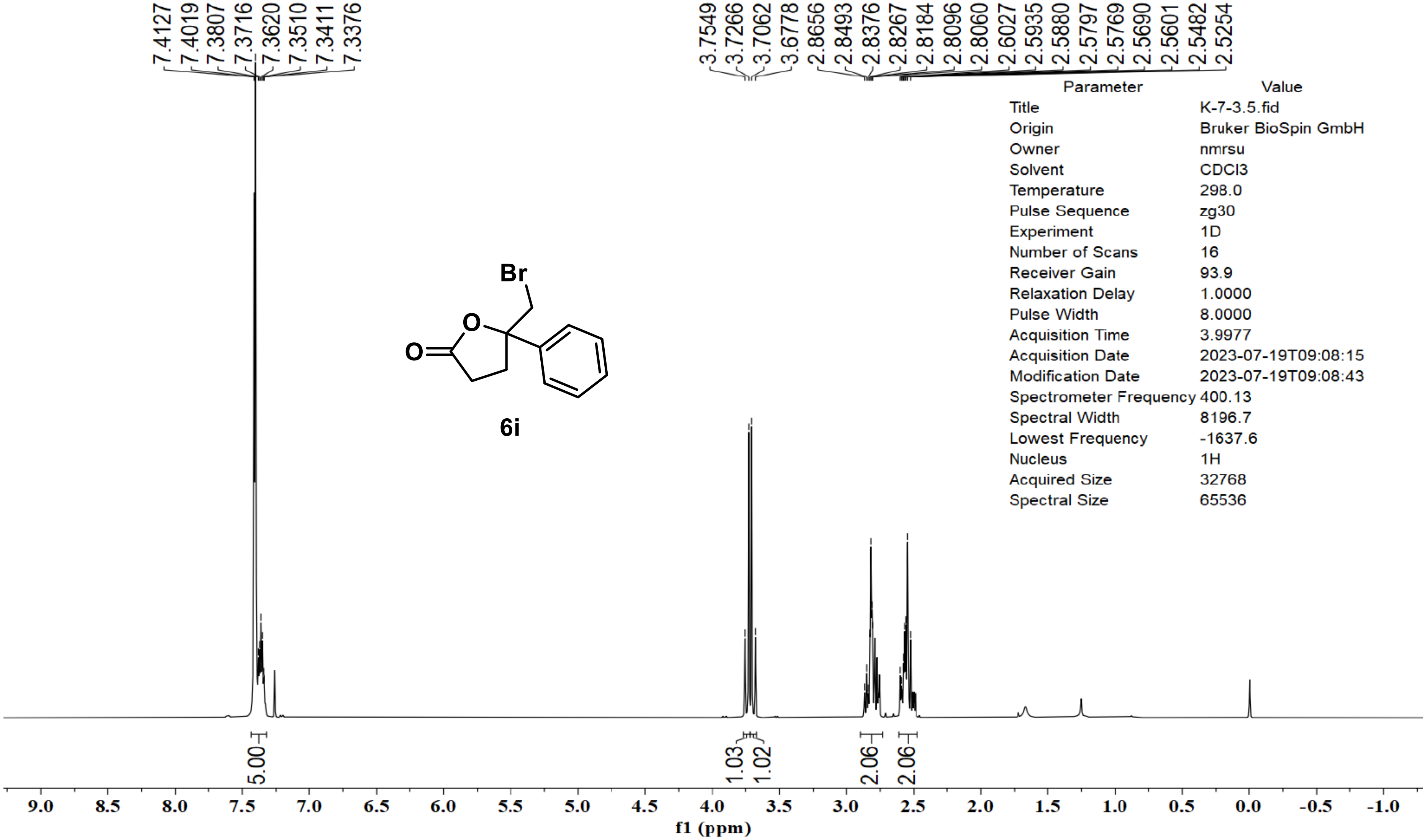

Parameter	Value
Title	K-7-3.5.fid
Origin	Bruker BioSpin GmbH
Owner	nmrsu
Solvent	CDCl3
Temperature	298.0
Pulse Sequence	zg30
Experiment	1D
Number of Scans	16
Receiver Gain	93.9
Relaxation Delay	1.0000
Pulse Width	8.0000
Acquisition Time	3.9977
Acquisition Date	2023-07-19T09:08:15
Modification Date	2023-07-19T09:08:43
Spectrometer Frequency	400.13
Spectral Width	8196.7
Lowest Frequency	-1637.6
Nucleus	1H
Acquired Size	32768
Spectral Size	65536

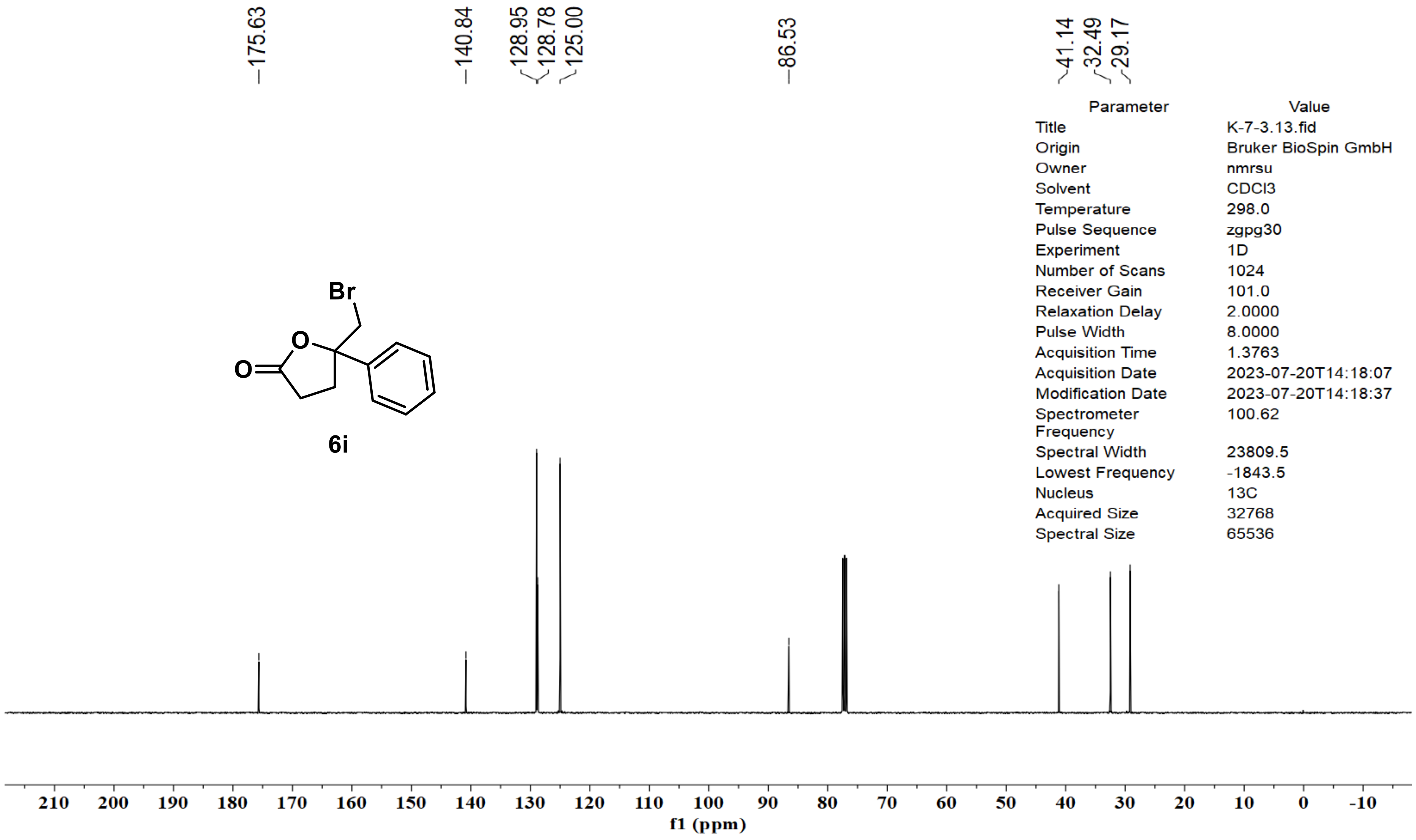

6i

Parameter	Value
Title	K-7-3.13.fid
Origin	Bruker BioSpin GmbH
Owner	nmrsu
Solvent	CDCl3
Temperature	298.0
Pulse Sequence	zgpg30
Experiment	1D
Number of Scans	1024
Receiver Gain	101.0
Relaxation Delay	2.0000
Pulse Width	8.0000
Acquisition Time	1.3763
Acquisition Date	2023-07-20T14:18:07
Modification Date	2023-07-20T14:18:37
Spectrometer Frequency	100.62
Spectral Width	23809.5
Lowest Frequency	-1843.5
Nucleus	13C
Acquired Size	32768
Spectral Size	65536

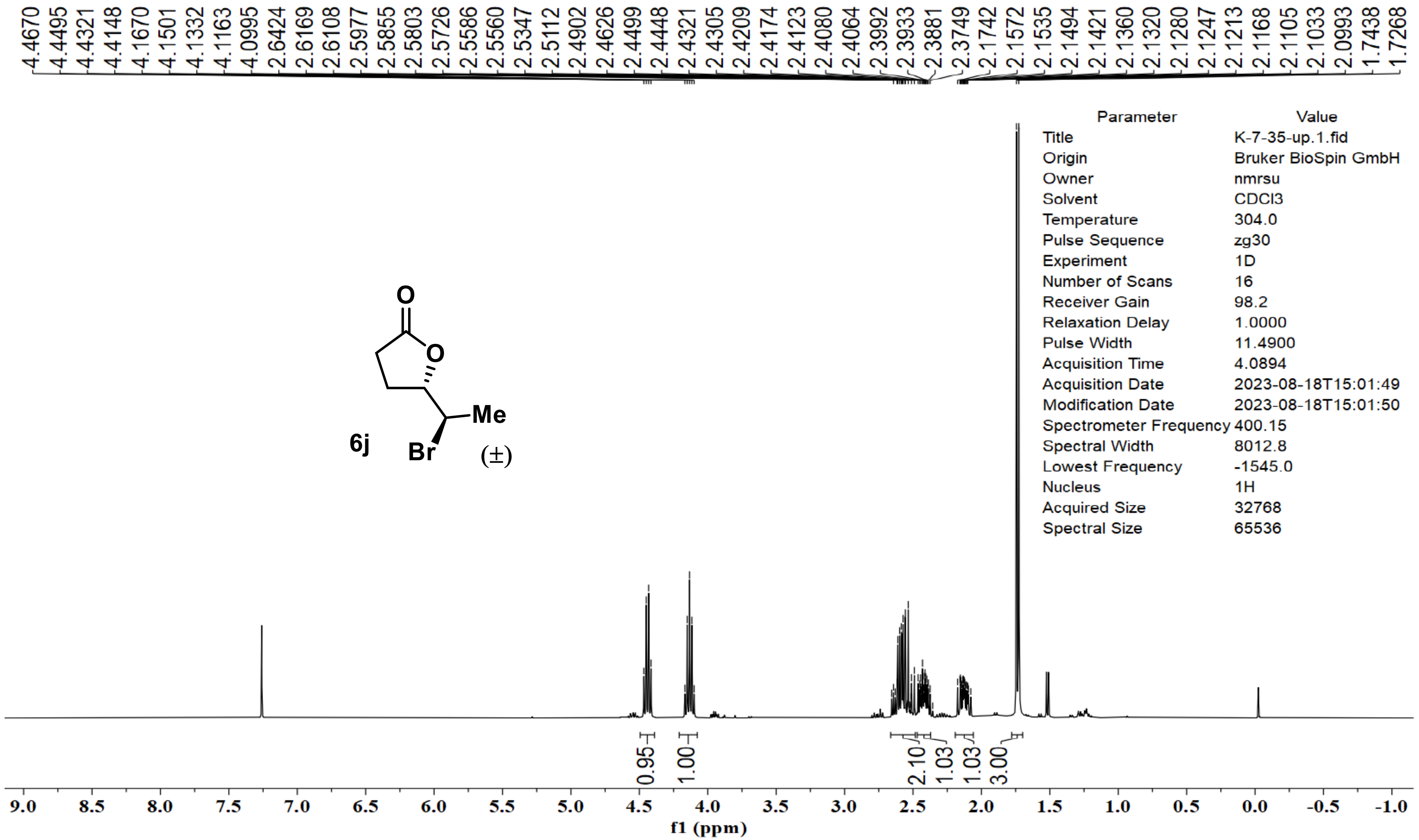
4.4670
4.4495
4.4321
4.4148
4.1670
4.1501
4.1332
4.1163
4.0995
2.6424
2.6169
2.6108
2.5977
2.5855
2.5803
2.5726
2.5586
2.5560
2.5347
2.5112
2.4902
2.4626
2.4499
2.4448
2.4321
2.4305
2.4209
2.4174
2.4123
2.4080
2.4064
2.3992
2.3933
2.3881
2.3749
2.1742
2.1572
2.1535
2.1494
2.1421
2.1360
2.1320
2.1280
2.1247
2.1213
2.1168
2.1105
2.1033
2.0993
1.7438
1.7268
O
O
Me
6j
Br
(±)
Parameter
Value
Title
K-7-35-up.1.fid
Origin
Bruker BioSpin GmbH
Owner
nmrsu
Solvent
CDCl3
Temperature
304.0
Pulse Sequence
zg30
Experiment
1D
Number of Scans
16
Receiver Gain
98.2
Relaxation Delay
1.0000
Pulse Width
11.4900
Acquisition Time
4.0894
Acquisition Date
2023-08-18T15:01:49
Modification Date
2023-08-18T15:01:50
Spectrometer Frequency
400.15
Spectral Width
8012.8
Lowest Frequency
-1545.0
Nucleus
1H
Acquired Size
32768
Spectral Size
65536
0.95
1.00
2.10
1.03
1.03
3.00
9.0
8.5
8.0
7.5
7.0
6.5
6.0
5.5
5.0
4.5
4.0
3.5
3.0
2.5
2.0
1.5
1.0
0.5
0.0
-0.5
-1.0
f1 (ppm)

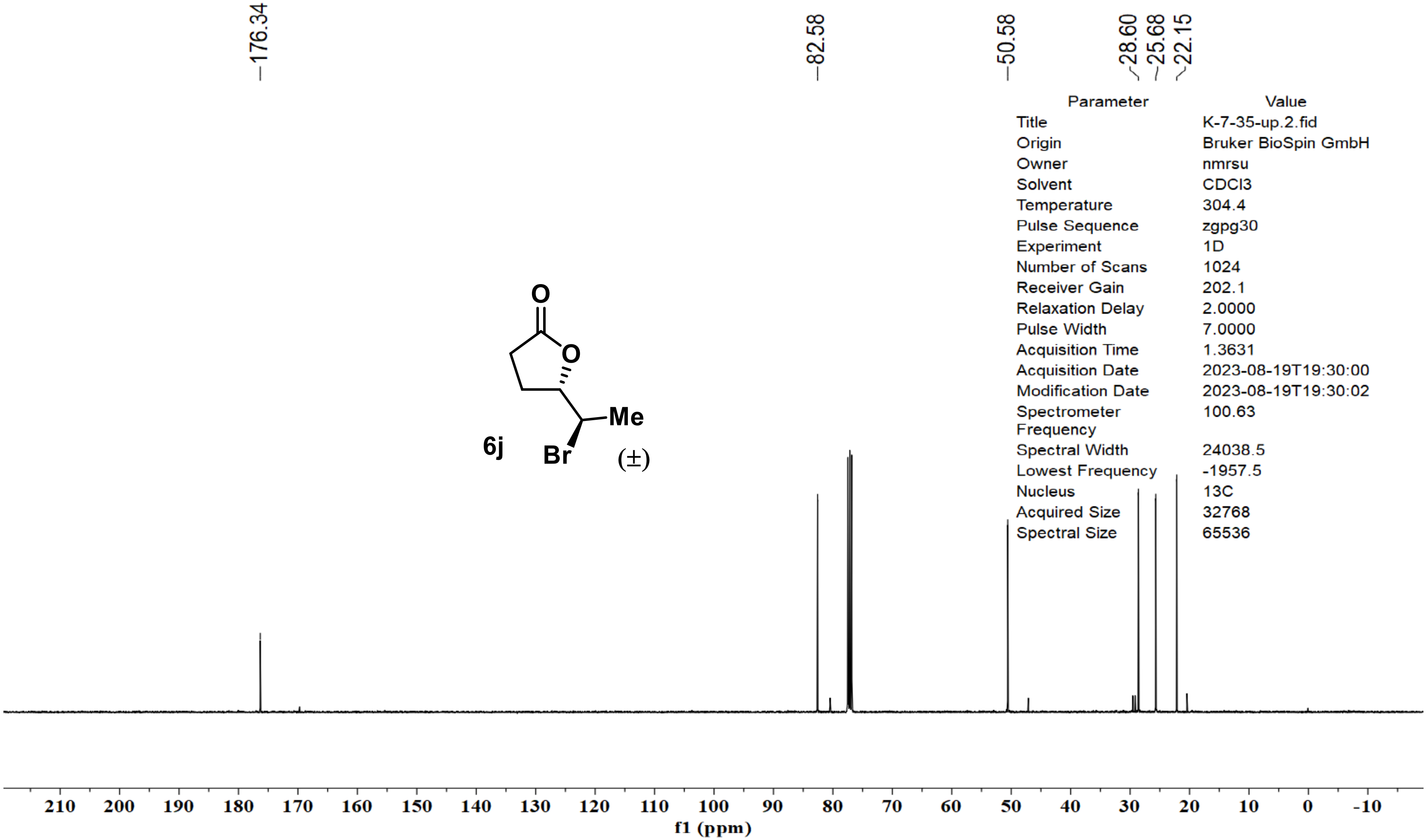

Parameter	Value
Title	K-7-35-up.2.fid
Origin	Bruker BioSpin GmbH
Owner	nmrsu
Solvent	CDCl3
Temperature	304.4
Pulse Sequence	zgpg30
Experiment	1D
Number of Scans	1024
Receiver Gain	202.1
Relaxation Delay	2.0000
Pulse Width	7.0000
Acquisition Time	1.3631
Acquisition Date	2023-08-19T19:30:00
Modification Date	2023-08-19T19:30:02
Spectrometer Frequency	100.63
Spectral Width	24038.5
Lowest Frequency	-1957.5
Nucleus	13C
Acquired Size	32768
Spectral Size	65536

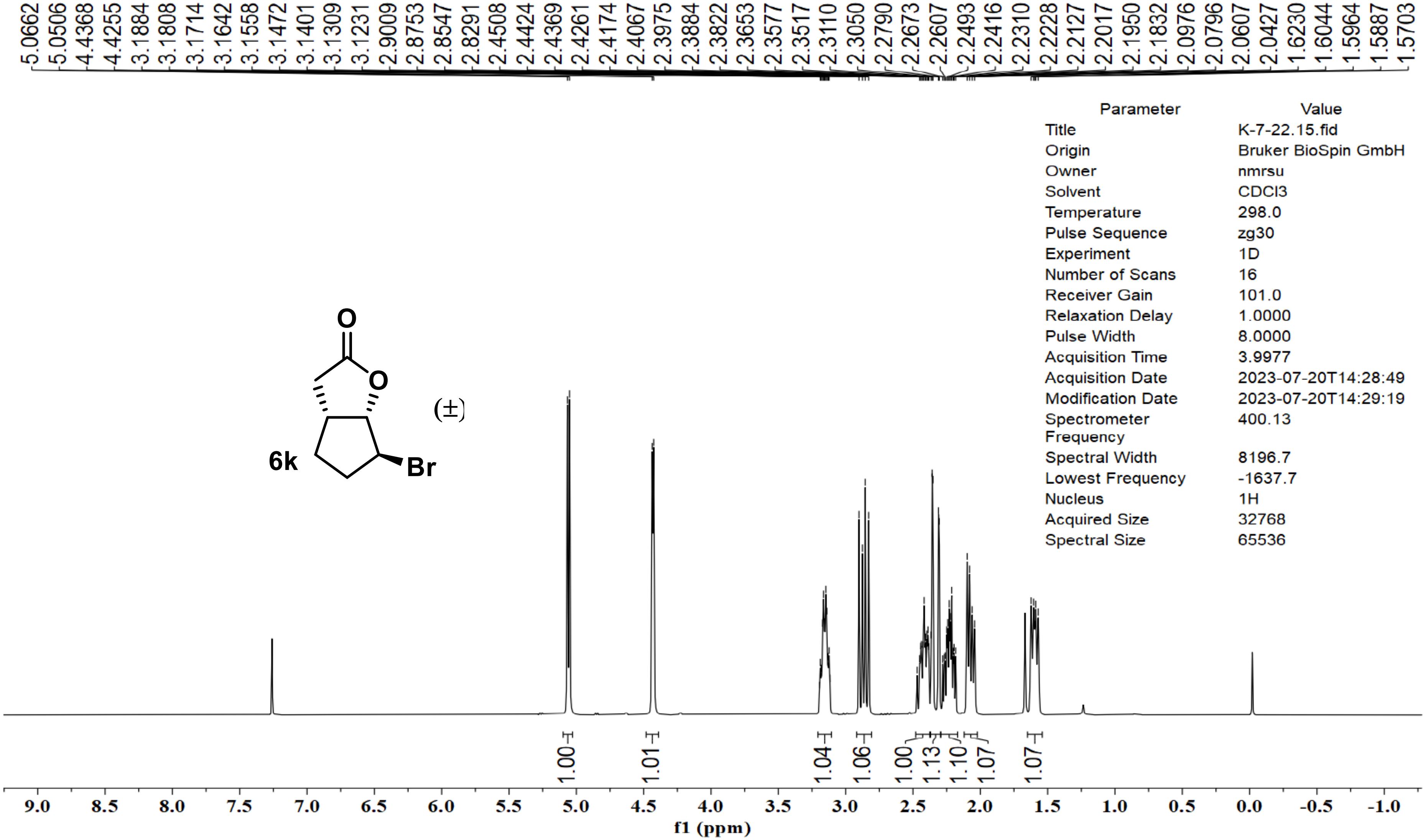
5.0662
5.0506
4.4368
4.4255
3.1884
3.1808
3.1714
3.1642
3.1558
3.1472
3.1401
3.1309
3.1231
2.9009
2.8753
2.8547
2.8291
2.4508
2.4424
2.4369
2.4261
2.4174
2.4067
2.3975
2.3884
2.3822
2.3653
2.3577
2.3517
2.3110
2.3050
2.2790
2.2673
2.2607
2.2493
2.2416
2.2310
2.2228
2.2127
2.2017
2.1950
2.1832
2.0976
2.0796
2.0607
2.0427
1.6230
1.6044
1.5964
1.5887
1.5703
Parameter
Value
Title
K-7-22.15.fid
Origin
Bruker BioSpin GmbH
Owner
nmrsu
Solvent
CDCl3
Temperature
298.0
Pulse Sequence
zg30
Experiment
1D
Number of Scans
16
Receiver Gain
101.0
Relaxation Delay
1.0000
Pulse Width
8.0000
Acquisition Time
3.9977
Acquisition Date
2023-07-20T14:28:49
Modification Date
2023-07-20T14:29:19
Spectrometer Frequency
400.13
Spectral Width
8196.7
Lowest Frequency
-1637.7
Nucleus
1H
Acquired Size
32768
Spectral Size
65536
O
O
(±)
6k
Br
1.00
1.01
1.04
1.06
1.00
1.13
1.10
1.07
1.07
9.0
8.5
8.0
7.5
7.0
6.5
6.0
5.5
5.0
4.5
4.0
3.5
3.0
2.5
2.0
1.5
1.0
0.5
0.0
-0.5
-1.0
f1 (ppm)

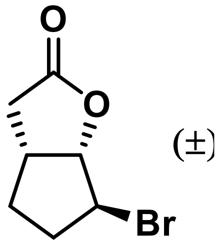

6k

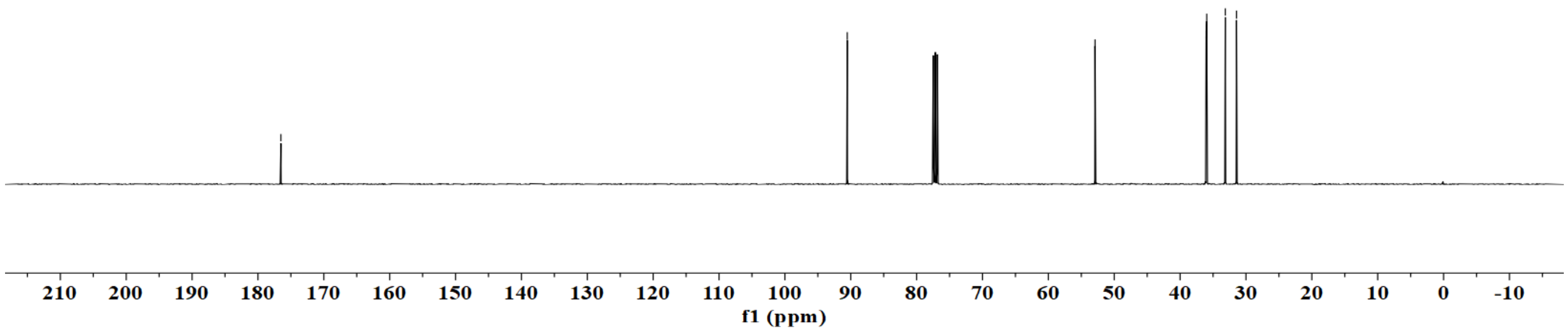

Parameter	Value
Title	K-7-22.20.fid
Origin	Bruker BioSpin GmbH
Owner	nmrsu
Solvent	CDCl3
Temperature	298.0
Pulse Sequence	zgpg30
Experiment	1D
Number of Scans	1024
Receiver Gain	101.0
Relaxation Delay	2.0000
Pulse Width	8.0000
Acquisition Time	1.3763
Acquisition Date	2023-07-21T19:15:16
Modification Date	2023-07-21T19:15:48
Spectrometer Frequency	100.62
Spectral Width	23809.5
Lowest Frequency	-1843.5
Nucleus	13C
Acquired Size	32768
Spectral Size	65536

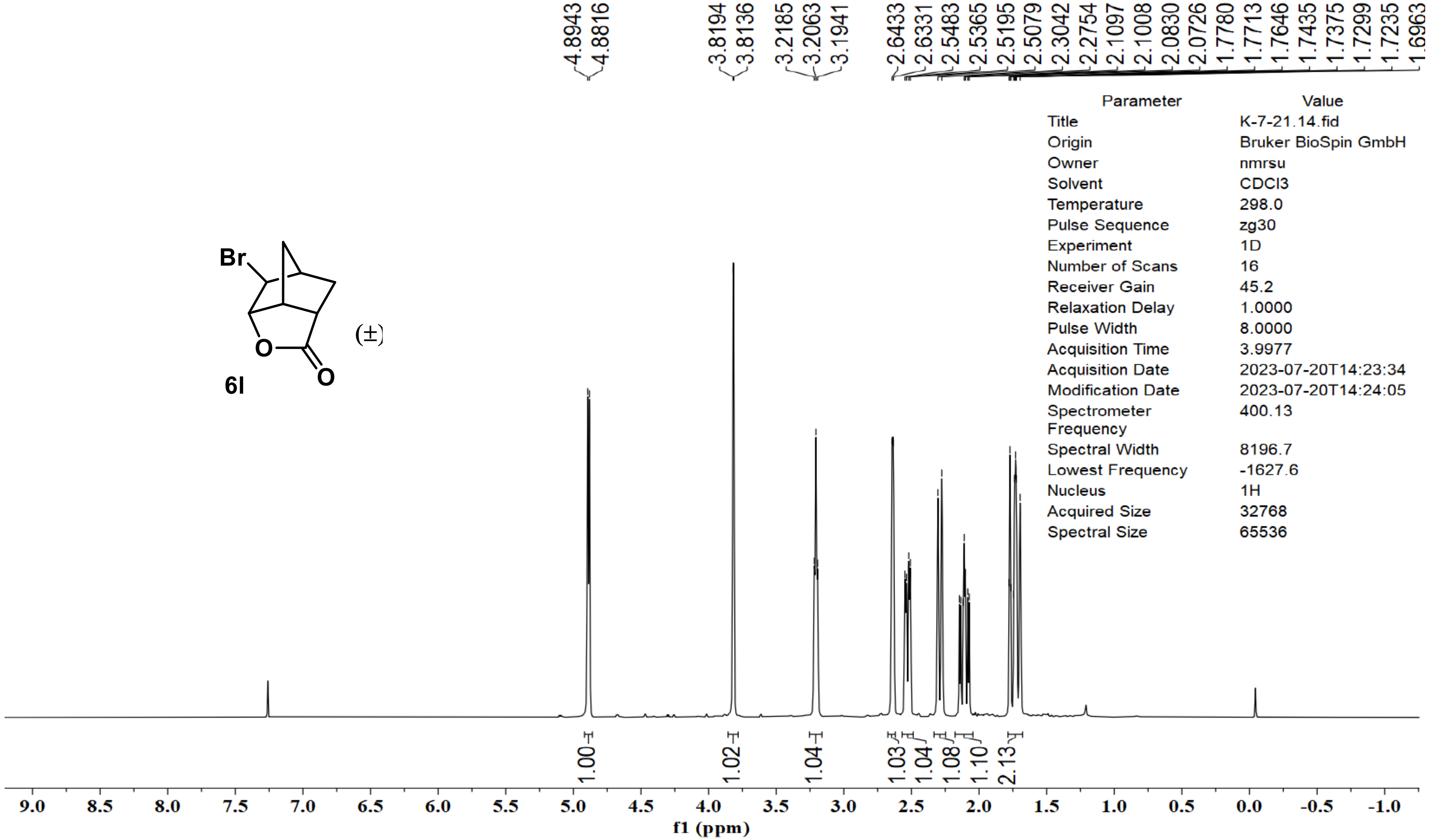

Parameter	Value
Title	K-7-21.14.fid
Origin	Bruker BioSpin GmbH
Owner	nmrsu
Solvent	CDCl3
Temperature	298.0
Pulse Sequence	zg30
Experiment	1D
Number of Scans	16
Receiver Gain	45.2
Relaxation Delay	1.0000
Pulse Width	8.0000
Acquisition Time	3.9977
Acquisition Date	2023-07-20T14:23:34
Modification Date	2023-07-20T14:24:05
Spectrometer Frequency	400.13
Spectral Width	8196.7
Lowest Frequency	-1627.6
Nucleus	1H
Acquired Size	32768
Spectral Size	65536

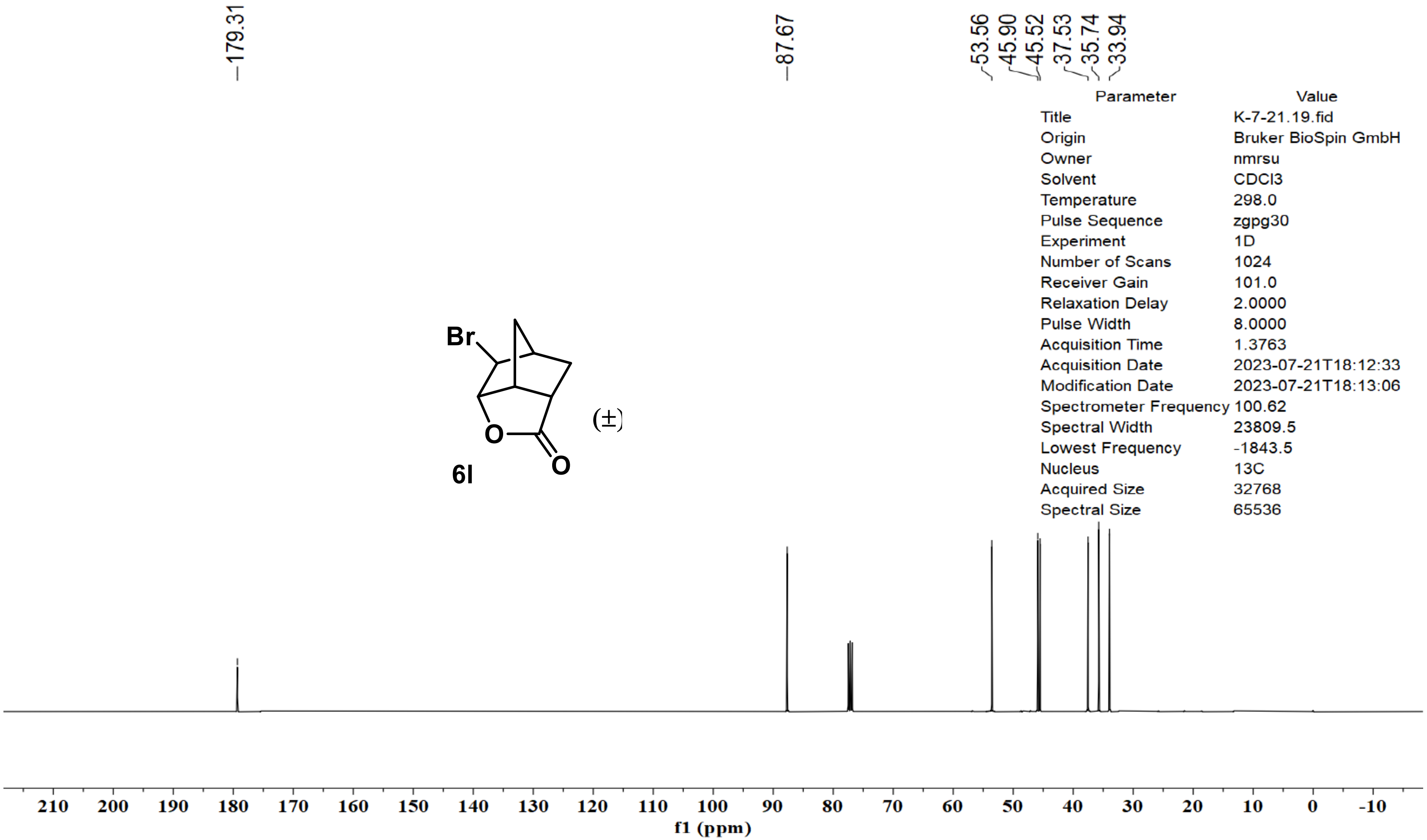

Parameter	Value
Title	K-7-21.19.fid
Origin	Bruker BioSpin GmbH
Owner	nmrsu
Solvent	CDCl3
Temperature	298.0
Pulse Sequence	zgpg30
Experiment	1D
Number of Scans	1024
Receiver Gain	101.0
Relaxation Delay	2.0000
Pulse Width	8.0000
Acquisition Time	1.3763
Acquisition Date	2023-07-21T18:12:33
Modification Date	2023-07-21T18:13:06
Spectrometer Frequency	100.62
Spectral Width	23809.5
Lowest Frequency	-1843.5
Nucleus	13C
Acquired Size	32768
Spectral Size	65536

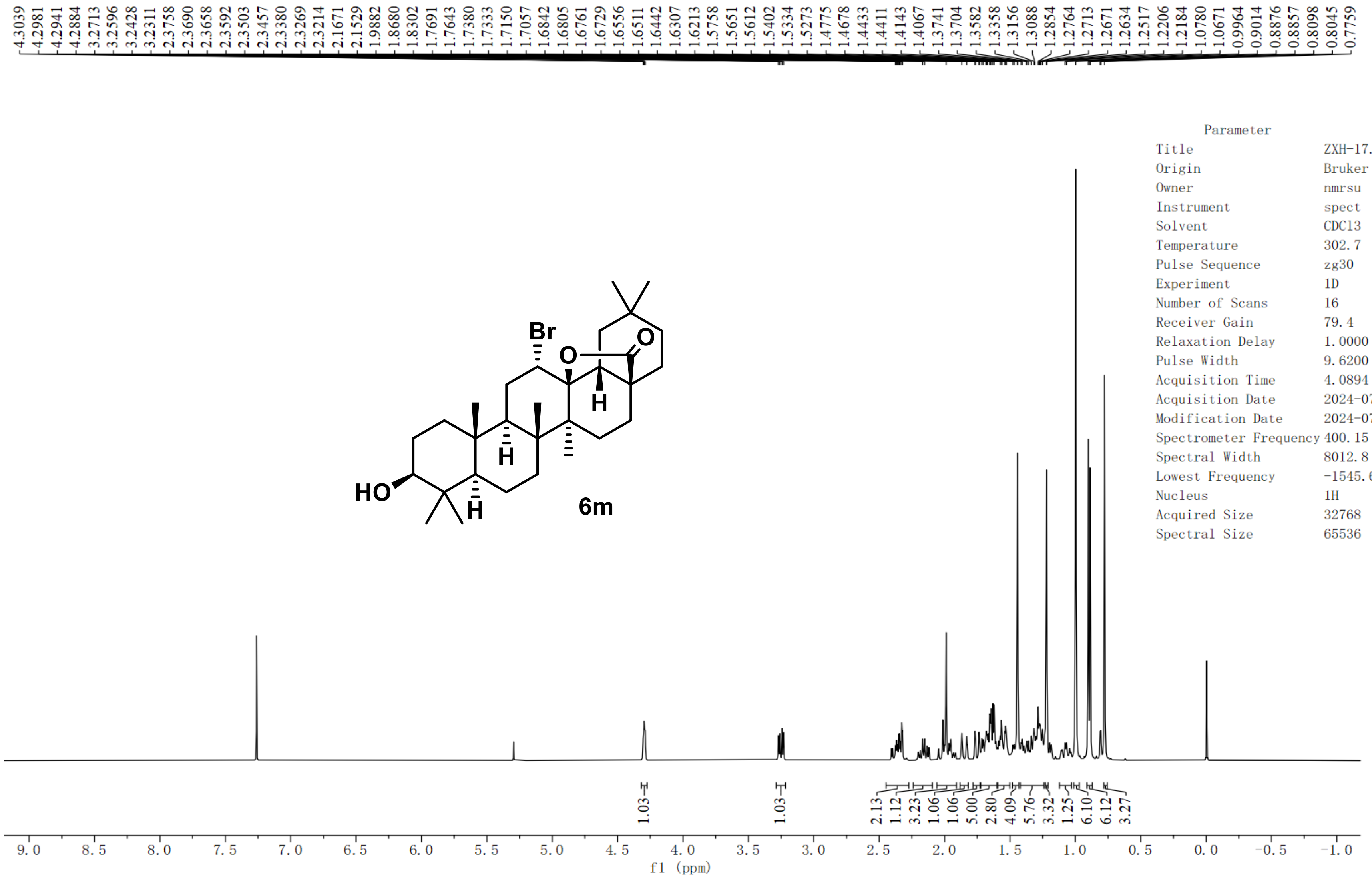

Parameter	Value
Title	ZXH-17.1.fid
Origin	Bruker BioSpin GmbH
Owner	nmrsu
Instrument	spect
Solvent	CDCl3
Temperature	302.7
Pulse Sequence	zg30
Experiment	1D
Number of Scans	16
Receiver Gain	79.4
Relaxation Delay	1.0000
Pulse Width	9.6200
Acquisition Time	4.0894
Acquisition Date	2024-07-22T12:26:06
Modification Date	2024-07-22T12:26:08
Spectrometer Frequency	400.15
Spectral Width	8012.8
Lowest Frequency	-1545.6
Nucleus	1H
Acquired Size	32768
Spectral Size	65536

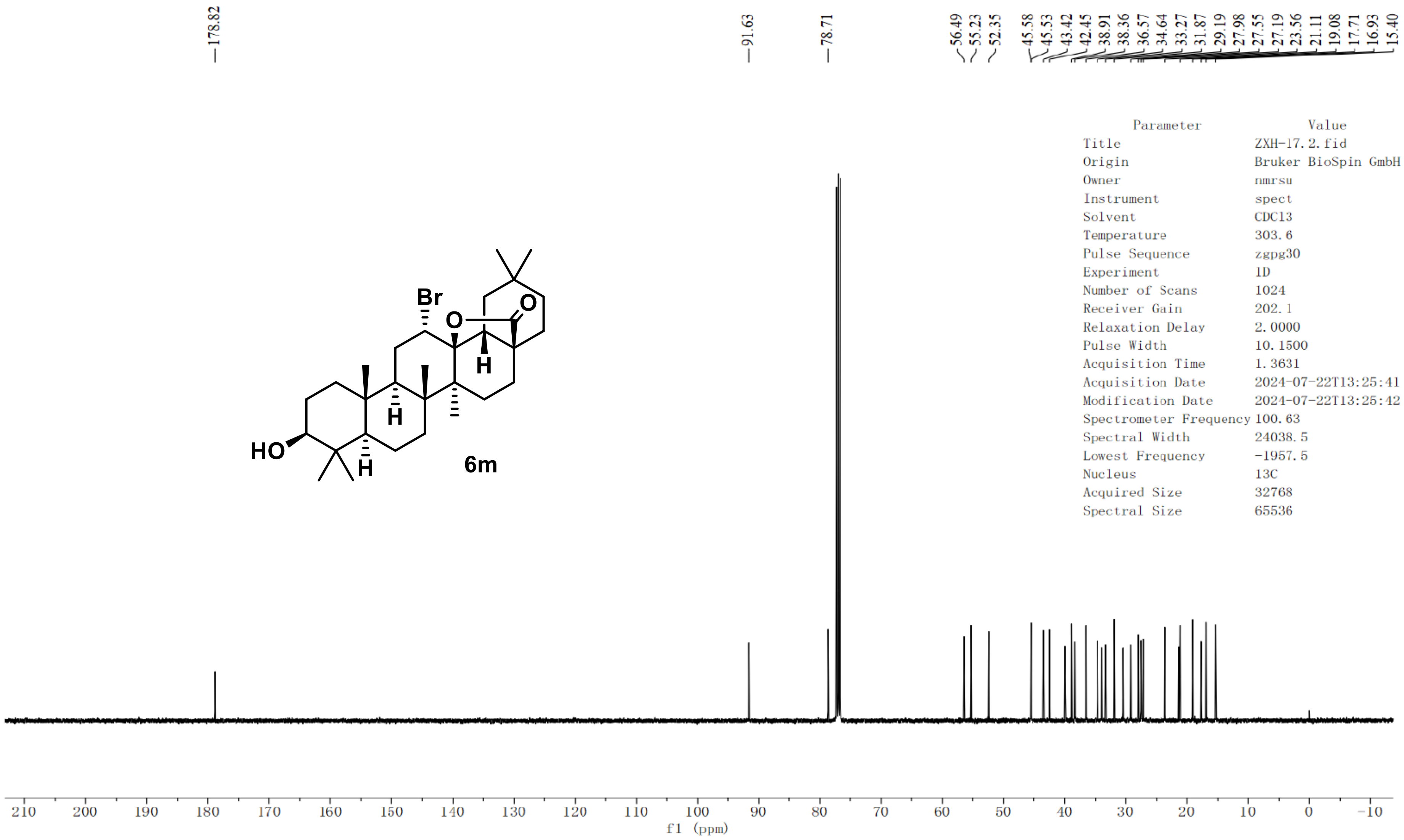

6m

Parameter	Value
Title	ZXH-17.2.fid
Origin	Bruker BioSpin GmbH
Owner	nmrsu
Instrument	spect
Solvent	CDCl3
Temperature	303.6
Pulse Sequence	zgpg30
Experiment	1D
Number of Scans	1024
Receiver Gain	202.1
Relaxation Delay	2.0000
Pulse Width	10.1500
Acquisition Time	1.3631
Acquisition Date	2024-07-22T13:25:41
Modification Date	2024-07-22T13:25:42
Spectrometer Frequency	100.63
Spectral Width	24038.5
Lowest Frequency	-1957.5
Nucleus	13C
Acquired Size	32768
Spectral Size	65536

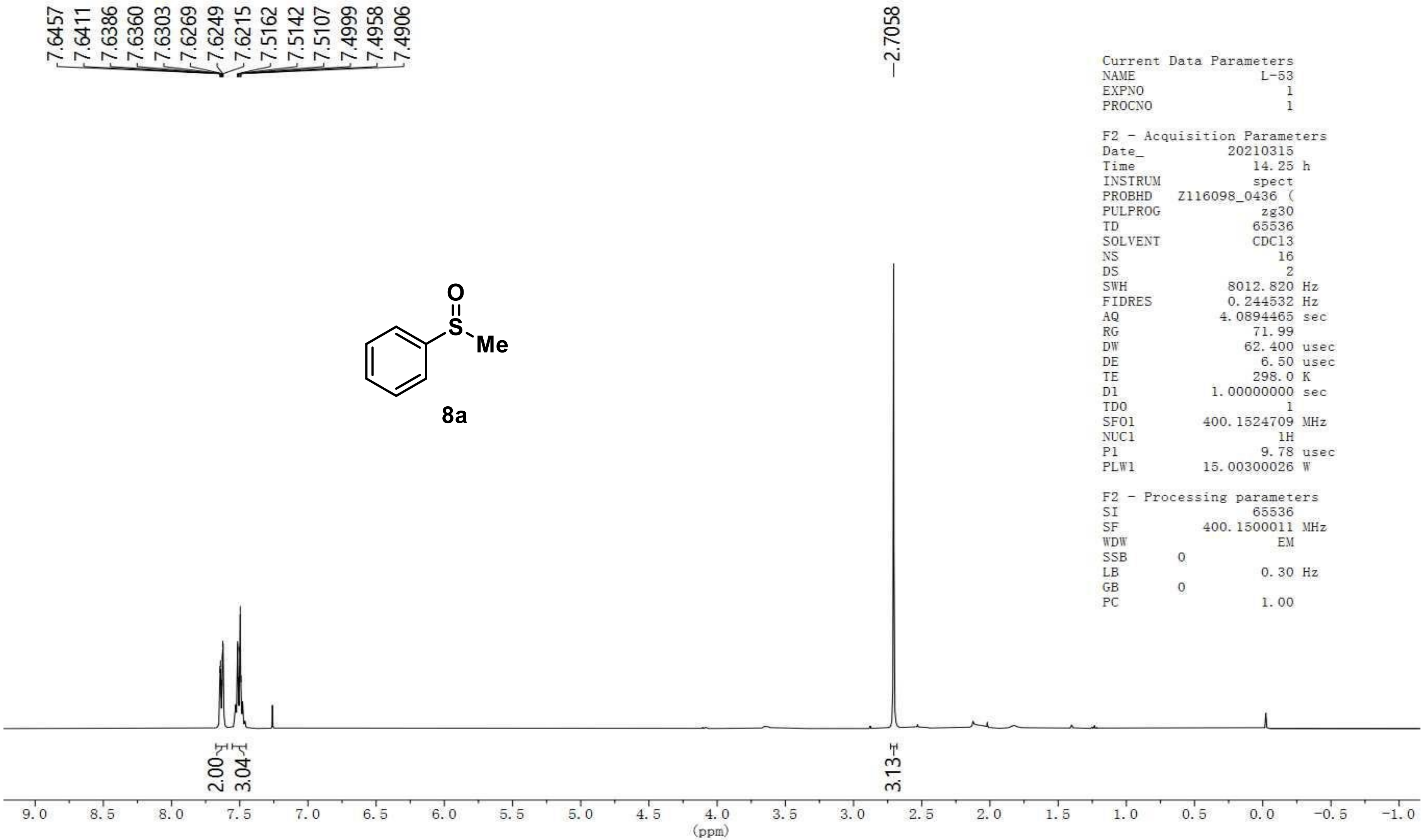

7.6457
7.6411
7.6386
7.6360
7.6303
7.6269
7.6249
7.6215
7.5162
7.5142
7.5107
7.4999
7.4958
7.4906
2.7058
O
S
Me
8a
Current Data Parameters
NAME L-53
EXPNO 1
PROCNO 1
F2 - Acquisition Parameters
Date_ 20210315
Time 14.25 h
INSTRUM spect
PROBHD Z116098_0436 (
PULPROG zg30
TD 65536
SOLVENT CDCl3
NS 16
DS 2
SWH 8012.820 Hz
FIDRES 0.244532 Hz
AQ 4.0894465 sec
RG 71.99
DW 62.400 usec
DE 6.50 usec
TE 298.0 K
D1 1.00000000 sec
TD0 1
SFO1 400.1524709 MHz
NUC1 1H
P1 9.78 usec
PLW1 15.00300026 W
F2 - Processing parameters
SI 65536
SF 400.1500011 MHz
WDW EM
SSB 0
LB 0.30 Hz
GB 0
PC 1.00
2.00
3.04
3.13
9.0 8.5 8.0 7.5 7.0 6.5 6.0 5.5 5.0 4.5 4.0 3.5 3.0 2.5 2.0 1.5 1.0 0.5 0.0 -0.5 -1.0
(ppm)

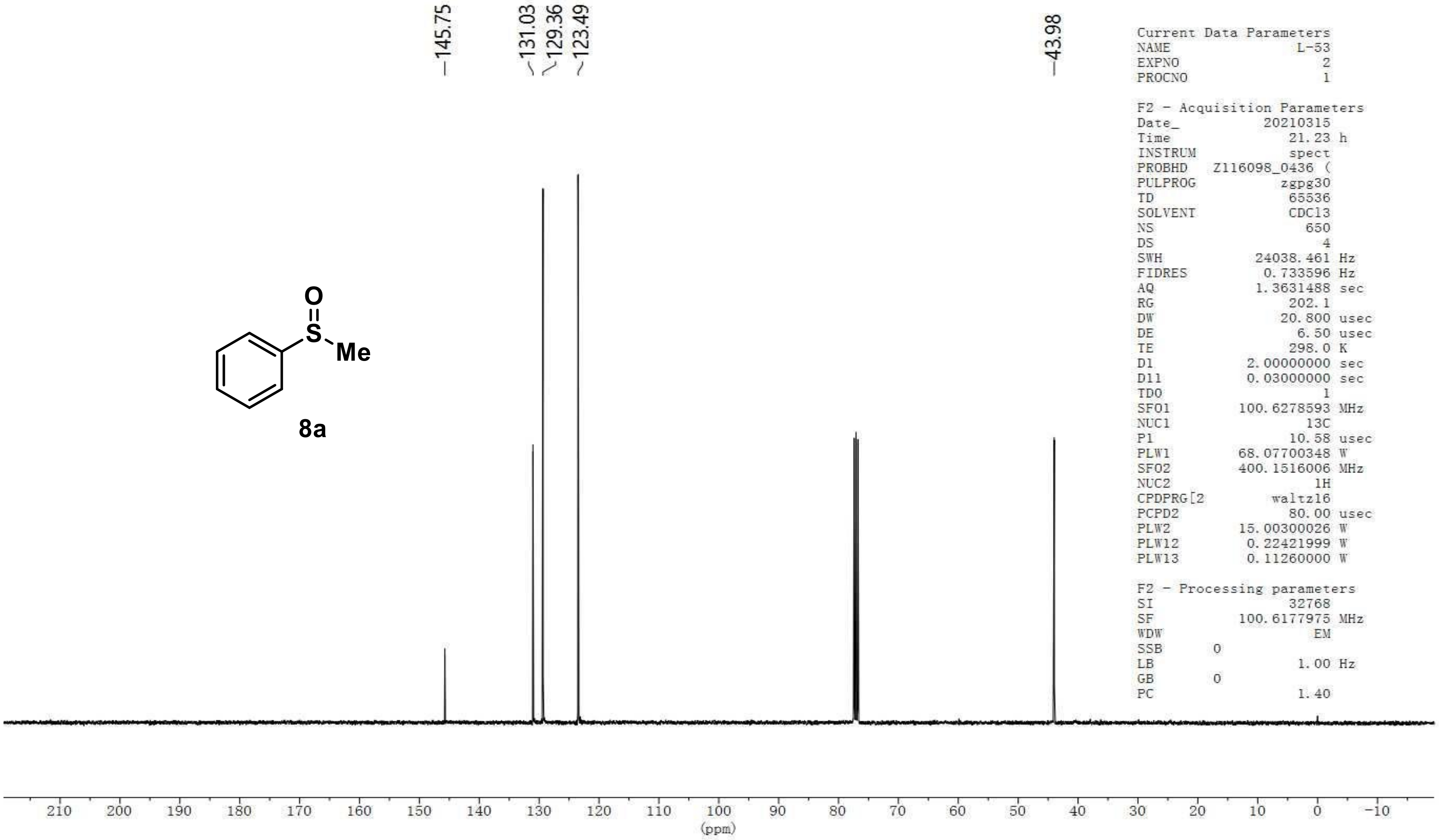
8a
145.75
131.03
129.36
123.49
43.98
Current Data Parameters
NAME L-53
EXPNO 2
PROCNO 1
F2 - Acquisition Parameters
Date_ 20210315
Time 21.23 h
INSTRUM spect
PROBHD Z116098_0436 (
PULPROG zgpg30
TD 65536
SOLVENT CDCl3
NS 650
DS 4
SWH 24038.461 Hz
FIDRES 0.733596 Hz
AQ 1.3631488 sec
RG 202.1
DW 20.800 usec
DE 6.50 usec
TE 298.0 K
D1 2.00000000 sec
D11 0.03000000 sec
TD0 1
SFO1 100.6278593 MHz
NUC1 13C
P1 10.58 usec
PLW1 68.07700348 W
SFO2 400.1516006 MHz
NUC2 1H
CPDPRG[2 waltz16
PCPD2 80.00 usec
PLW2 15.00300026 W
PLW12 0.22421999 W
PLW13 0.11260000 W
F2 - Processing parameters
SI 32768
SF 100.6177975 MHz
WDW EM
SSB 0
LB 1.00 Hz
GB 0
PC 1.40
210 200 190 180 170 160 150 140 130 120 110 100 90 80 70 60 50 40 30 20 10 0 -10
(ppm)

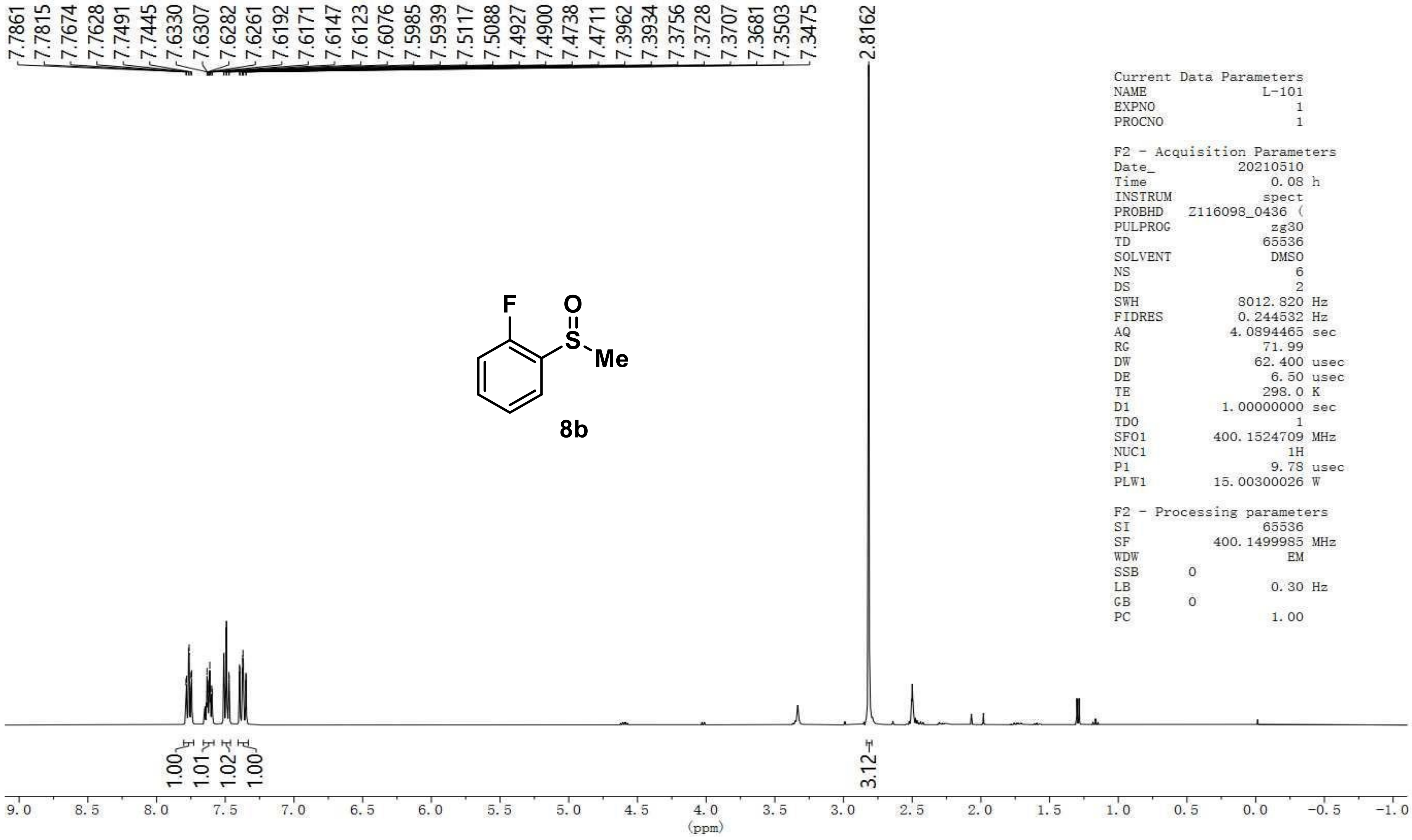

7.7861
7.7815
7.7674
7.7628
7.7491
7.7445
7.6330
7.6307
7.6282
7.6261
7.6192
7.6171
7.6147
7.6123
7.6076
7.5985
7.5939
7.5117
7.5088
7.4927
7.4900
7.4738
7.4711
7.3962
7.3934
7.3756
7.3728
7.3707
7.3681
7.3503
7.3475
2.8162
F
O
S
Me
8b
Current Data Parameters
NAME L-101
EXPNO 1
PROCNO 1
F2 - Acquisition Parameters
Date_ 20210510
Time 0.08 h
INSTRUM spect
PROBHD Z116098_0436 (
PULPROG zg30
TD 65536
SOLVENT DMSO
NS 6
DS 2
SWH 8012.820 Hz
FIDRES 0.244532 Hz
AQ 4.0894465 sec
RG 71.99
DW 62.400 usec
DE 6.50 usec
TE 298.0 K
D1 1.00000000 sec
TD0 1
SFO1 400.1524709 MHz
NUC1 1H
P1 9.78 usec
PLW1 15.00300026 W
F2 - Processing parameters
SI 65536
SF 400.1499985 MHz
WDW EM
SSB 0
LB 0.30 Hz
GB 0
PC 1.00
1.00
1.01
1.02
1.00
3.12
9.0 8.5 8.0 7.5 7.0 6.5 6.0 5.5 5.0 4.5 4.0 3.5 3.0 2.5 2.0 1.5 1.0 0.5 0.0 -0.5 -1.0
(ppm)

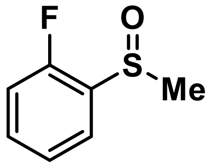

8b

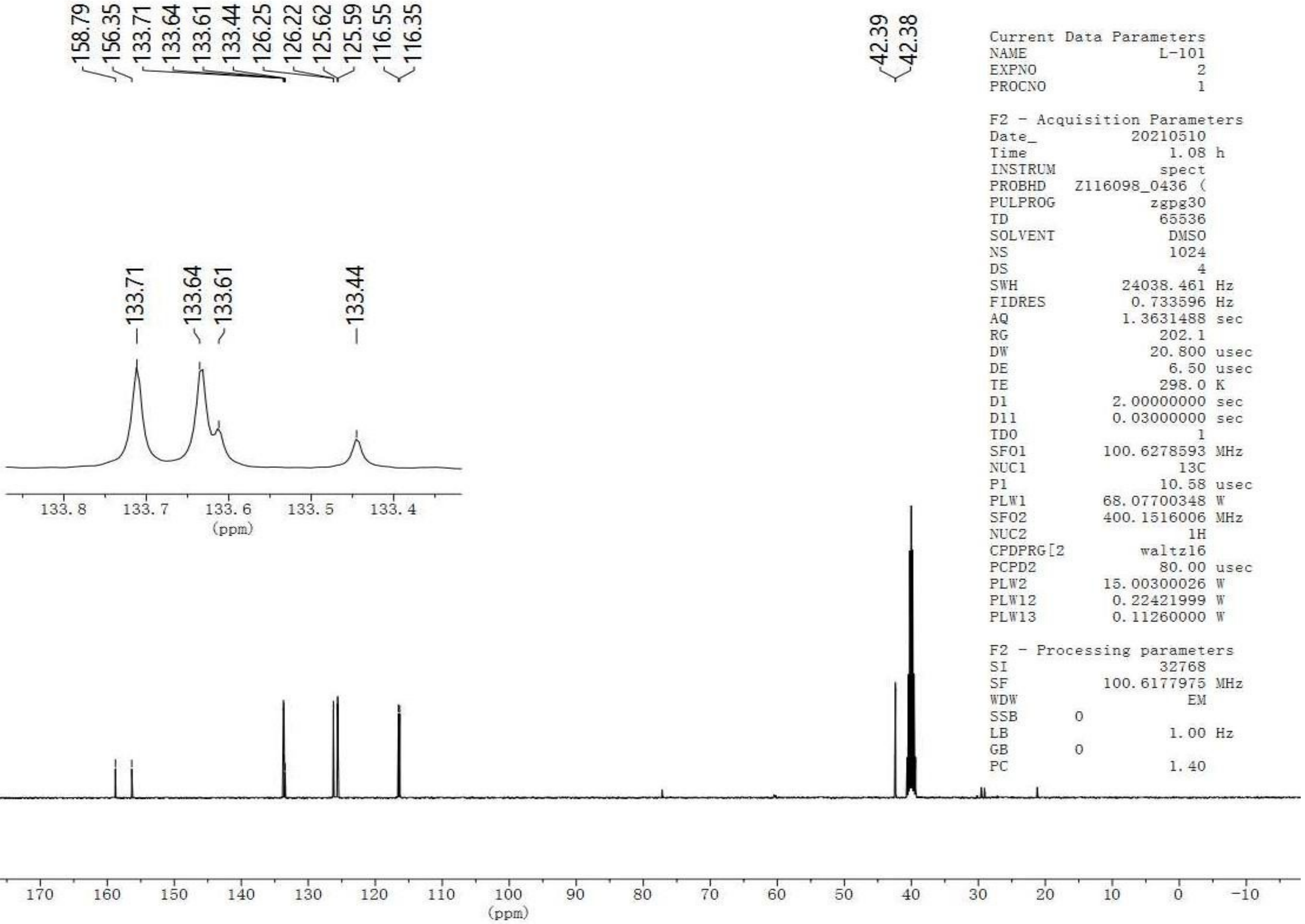

Current Data Parameters
NAME L-101
EXPNO 2
PROCNO 1

F2 - Acquisition Parameters
Date_ 20210510
Time 1.08 h
INSTRUM spect
PROBHD Z116098_0436 (
PULPROG zgpg30
TD 65536
SOLVENT DMSO
NS 1024
DS 4
SWH 24038.461 Hz
FIDRES 0.733596 Hz
AQ 1.3631488 sec
RG 202.1
DW 20.800 usec
DE 6.50 usec
TE 298.0 K
D1 2.00000000 sec
D11 0.03000000 sec
TD0 1
SFO1 100.6278593 MHz
NUC1 13C
P1 10.58 usec
PLW1 68.07700348 W
SFO2 400.1516006 MHz
NUC2 1H
CPDPRG[2 waltz16
PCPD2 80.00 usec
PLW2 15.00300026 W
PLW12 0.22421999 W
PLW13 0.11260000 W

F2 - Processing parameters
SI 32768
SF 100.6177975 MHz
WDW EM
SSB 0
LB 1.00 Hz
GB 0
PC 1.40

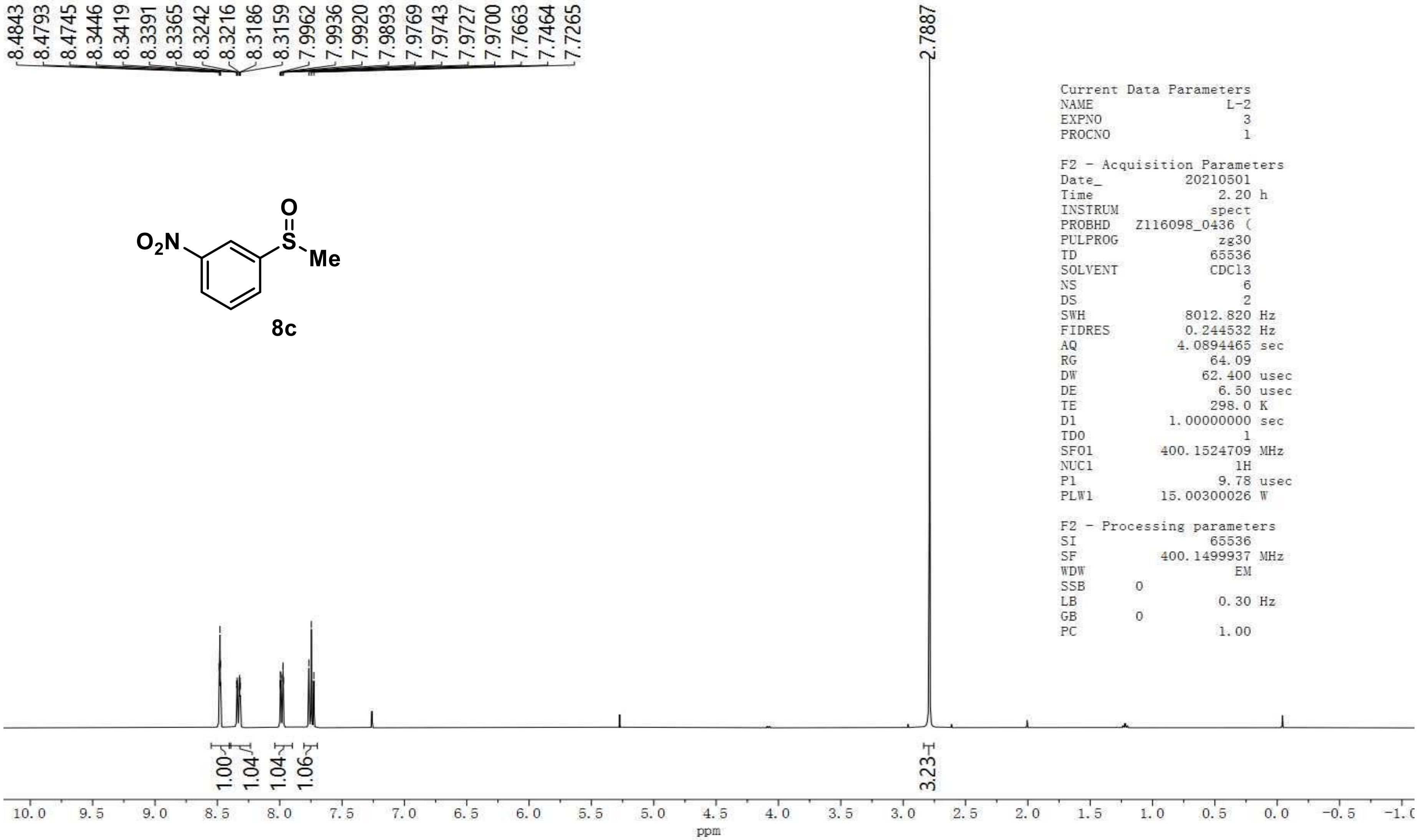
8.4843
8.4793
8.4745
8.3446
8.3419
8.3391
8.3365
8.3242
8.3216
8.3186
8.3159
7.9962
7.9936
7.9920
7.9893
7.9769
7.9743
7.9727
7.9700
7.7663
7.7464
7.7265
2.7887
O
O2N
S
Me
8c
Current Data Parameters
NAME L-2
EXPNO 3
PROCNO 1
F2 - Acquisition Parameters
Date_ 20210501
Time 2.20 h
INSTRUM spect
PROBHD Z116098_0436 (
PULPROG zg30
TD 65536
SOLVENT CDCl3
NS 6
DS 2
SWH 8012.820 Hz
FIDRES 0.244532 Hz
AQ 4.0894465 sec
RG 64.09
DW 62.400 usec
DE 6.50 usec
TE 298.0 K
D1 1.00000000 sec
TD0 1
SFO1 400.1524709 MHz
NUC1 1H
P1 9.78 usec
PLW1 15.00300026 W
F2 - Processing parameters
SI 65536
SF 400.1499937 MHz
WDW EM
SSB 0
LB 0.30 Hz
GB 0
PC 1.00
1.00
1.04
1.04
1.06
3.23
10.0
9.5
9.0
8.5
8.0
7.5
7.0
6.5
6.0
5.5
5.0
4.5
4.0
3.5
3.0
2.5
2.0
1.5
1.0
0.5
0.0
-0.5
-1.0
ppm

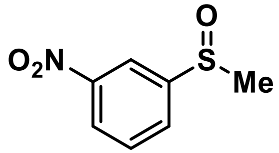
O
S
Me
O2N

8c

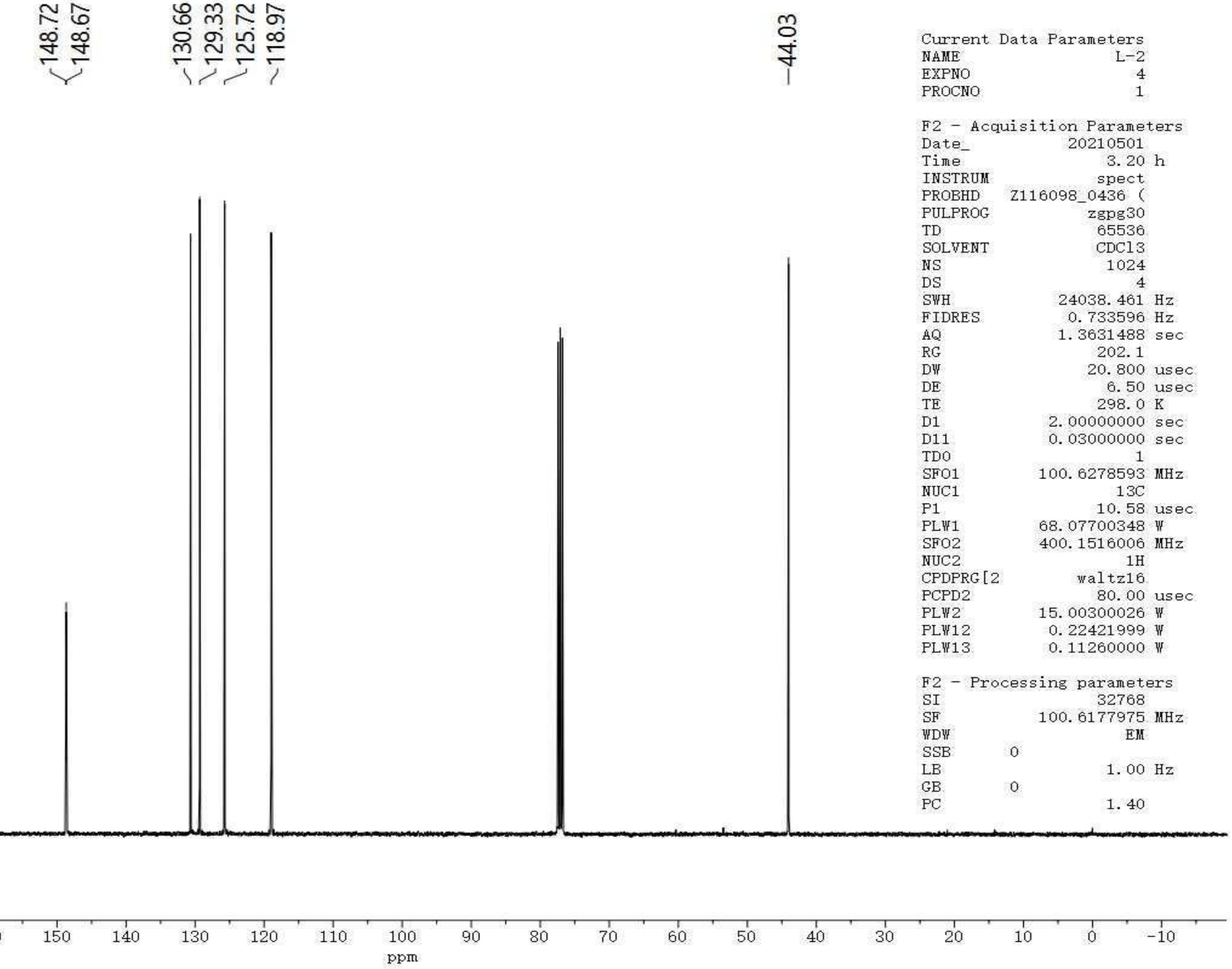
148.72
148.67
130.66
129.33
125.72
118.97
44.03
Current Data Parameters
NAME L-2
EXPNO 4
PROCNO 1
F2 - Acquisition Parameters
Date_ 20210501
Time 3.20 h
INSTRUM spect
PROBHD Z116098_0436 (
PULPROG zgpg30
TD 65536
SOLVENT CDCl3
NS 1024
DS 4
SWH 24038.461 Hz
FIDRES 0.733596 Hz
AQ 1.3631488 sec
RG 202.1
DW 20.800 usec
DE 6.50 usec
TE 298.0 K
D1 2.00000000 sec
D11 0.03000000 sec
TD0 1
SFO1 100.6278593 MHz
NUC1 13C
P1 10.58 usec
PLW1 68.07700348 W
SFO2 400.1516006 MHz
NUC2 1H
CPDPRG[2 waltz16
PCPD2 80.00 usec
PLW2 15.00300026 W
PLW12 0.22421999 W
PLW13 0.11260000 W
F2 - Processing parameters
SI 32768
SF 100.6177975 MHz
WDW EM
SSB 0
LB 1.00 Hz
GB 0
PC 1.40
210 200 190 180 170 160 150 140 130 120 110 100 90 80 70 60 50 40 30 20 10 0 -10
ppm

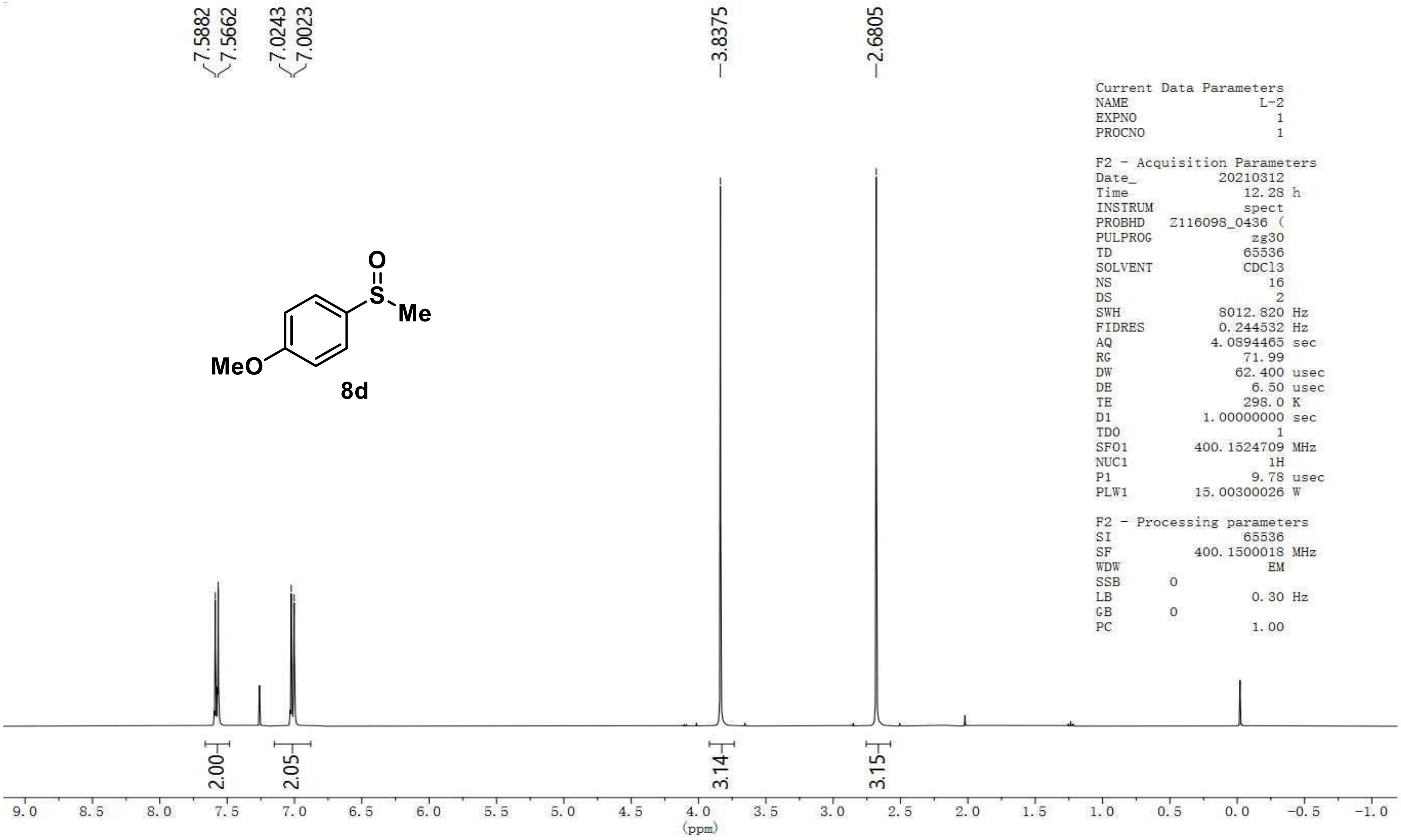
7.5882
7.5662
7.0243
7.0023
3.8375
2.6805
O
S
Me
MeO
8d
Current Data Parameters
NAME L-2
EXPNO 1
PROCNO 1
F2 - Acquisition Parameters
Date_ 20210312
Time 12.28 h
INSTRUM spect
PROBHD Z116098_0436 (
PULPROG zg30
TD 65536
SOLVENT CDCl3
NS 16
DS 2
SWH 8012.820 Hz
FIDRES 0.244532 Hz
AQ 4.0894465 sec
RG 71.99
DW 62.400 usec
DE 6.50 usec
TE 298.0 K
D1 1.00000000 sec
TD0 1
SFO1 400.1524709 MHz
NUC1 1H
P1 9.78 usec
PLW1 15.00300026 W
F2 - Processing parameters
SI 65536
SF 400.1500018 MHz
WDW EM
SSB 0
LB 0.30 Hz
GB 0
PC 1.00
2.00
2.05
3.14
3.15
9.0 8.5 8.0 7.5 7.0 6.5 6.0 5.5 5.0 4.5 4.0 3.5 3.0 2.5 2.0 1.5 1.0 0.5 0.0 -0.5 -1.0
(ppm)

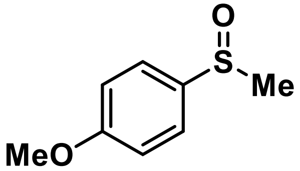

8d

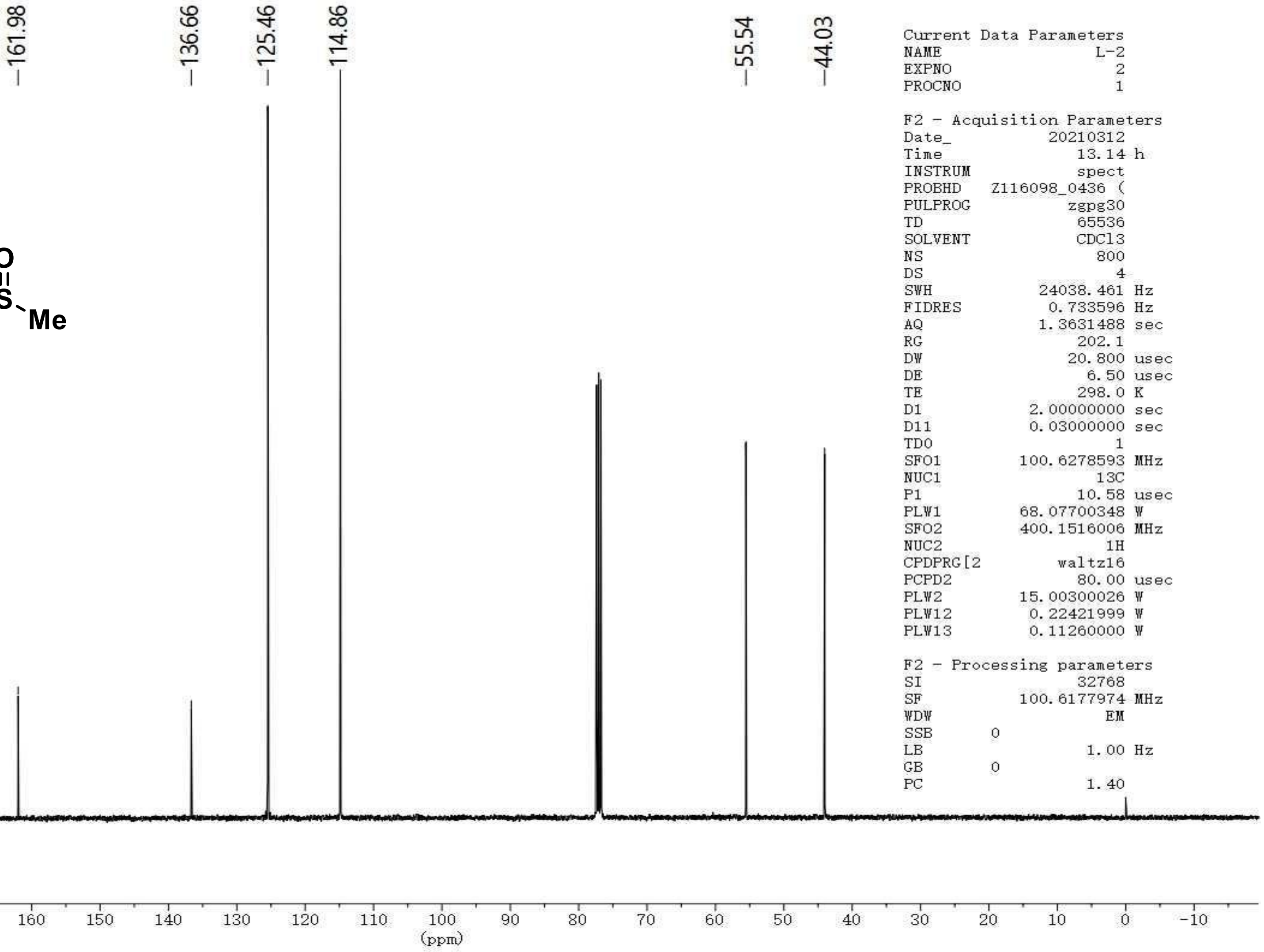

Current Data Parameters
NAME L-2
EXPNO 2
PROCNO 1

F2 - Acquisition Parameters
Date_ 20210312
Time 13.14 h
INSTRUM spect
PROBHD Z116098_0436 (
PULPROG zgpg30
TD 65536
SOLVENT CDCl3
NS 800
DS 4
SWH 24038.461 Hz
FIDRES 0.733596 Hz
AQ 1.3631488 sec
RG 202.1
DW 20.800 usec
DE 6.50 usec
TE 298.0 K
D1 2.00000000 sec
D11 0.03000000 sec
TD0 1
SFO1 100.6278593 MHz
NUC1 13C
P1 10.58 usec
PLW1 68.07700348 W
SFO2 400.1516006 MHz
NUC2 1H
CPDPRG[2 waltz16
PCPD2 80.00 usec
PLW2 15.00300026 W
PLW12 0.22421999 W
PLW13 0.11260000 W

F2 - Processing parameters
SI 32768
SF 100.6177974 MHz
WDW EM
SSB 0
LB 1.00 Hz
GB 0
PC 1.40

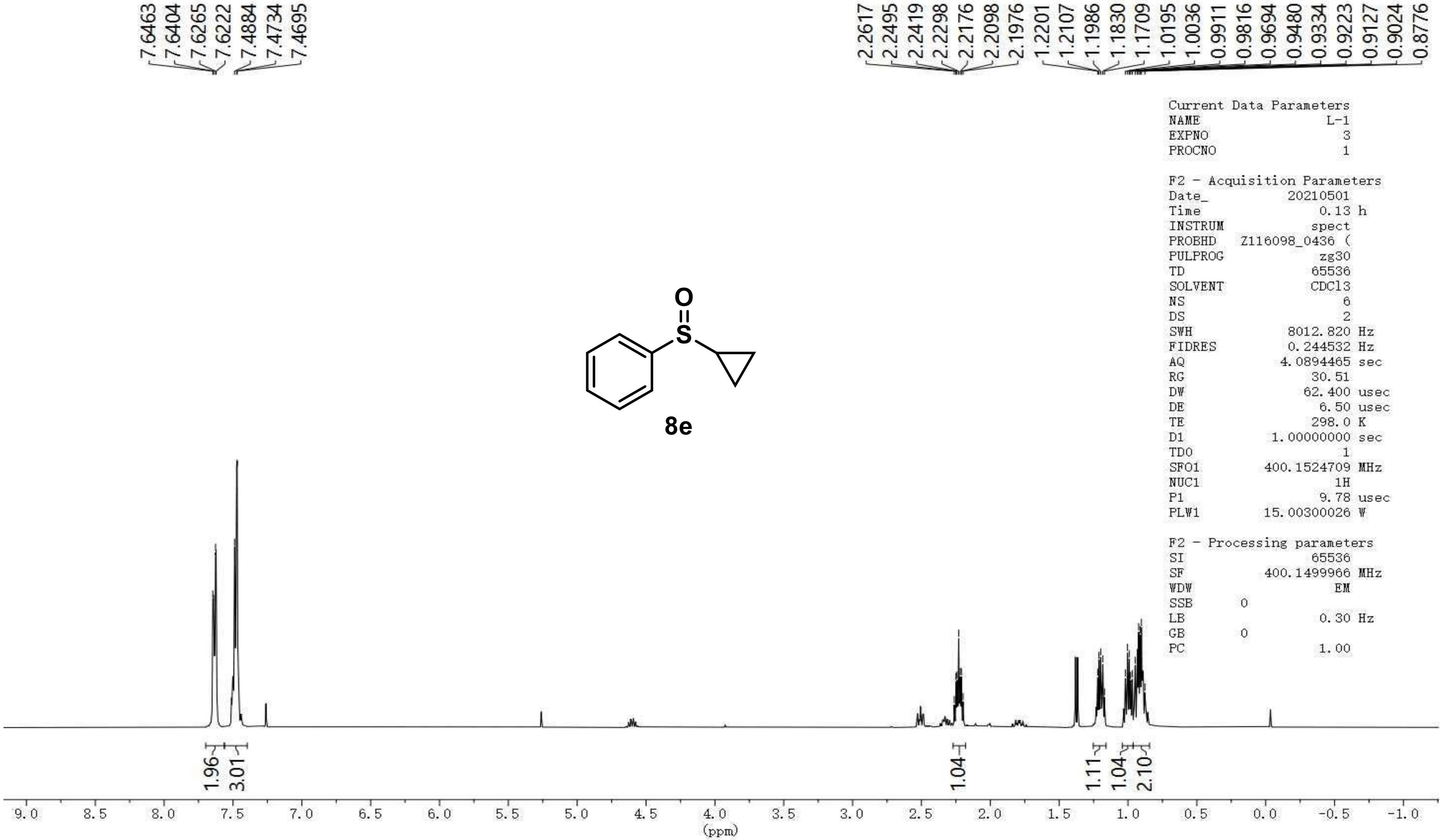
7.6463
7.6404
7.6265
7.6222
7.4884
7.4734
7.4695
2.2617
2.2495
2.2419
2.2298
2.2176
2.2098
2.1976
1.2201
1.2107
1.1986
1.1830
1.1709
1.0195
1.0036
0.9911
0.9816
0.9694
0.9480
0.9334
0.9223
0.9127
0.9024
0.8776
O
S
8e
Current Data Parameters
NAME L-1
EXPNO 3
PROCNO 1
F2 - Acquisition Parameters
Date_ 20210501
Time 0.13 h
INSTRUM spect
PROBHD Z116098_0436 (
PULPROG zg30
TD 65536
SOLVENT CDCl3
NS 6
DS 2
SWH 8012.820 Hz
FIDRES 0.244532 Hz
AQ 4.0894465 sec
RG 30.51
DW 62.400 usec
DE 6.50 usec
TE 298.0 K
D1 1.00000000 sec
TD0 1
SFO1 400.1524709 MHz
NUC1 1H
P1 9.78 usec
PLW1 15.00300026 W
F2 - Processing parameters
SI 65536
SF 400.1499966 MHz
WDW EM
SSB 0
LB 0.30 Hz
GB 0
PC 1.00
1.96
3.01
1.04
1.11
1.04
2.10
9.0 8.5 8.0 7.5 7.0 6.5 6.0 5.5 5.0 4.5 4.0 3.5 3.0 2.5 2.0 1.5 1.0 0.5 0.0 -0.5 -1.0
(ppm)

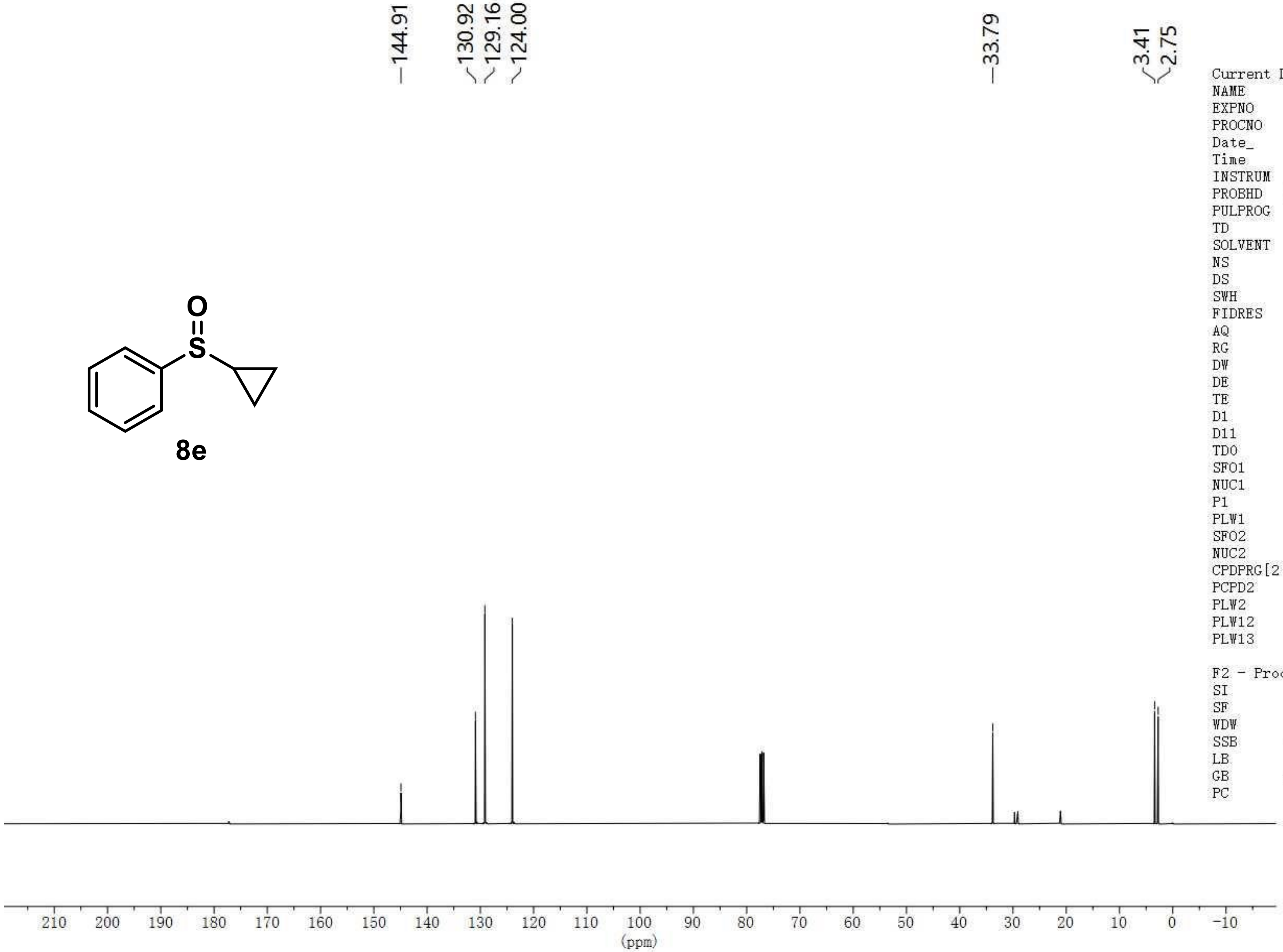

Current Data Parameters
NAME L-1
EXPNO 4
PROCNO 1
Date_ 20210501
Time 1.12 h
INSTRUM spect
PROBHD Z116098_0436 (
PULPROG zgpg30
TD 65536
SOLVENT CDCl3
NS 1024
DS 4
SWH 24038.461 Hz
FIDRES 0.733596 Hz
AQ 1.3631488 sec
RG 202.1
DW 20.800 usec
DE 6.50 usec
TE 298.0 K
D1 2.00000000 sec
D11 0.03000000 sec
TD0 1
SFO1 100.6278593 MHz
NUC1 13C
P1 10.58 usec
PLW1 68.07700348 W
SFO2 400.1516006 MHz
NUC2 1H
CPDPRG[2 waltz16
PCPD2 80.00 usec
PLW2 15.00300026 W
PLW12 0.22421999 W
PLW13 0.11260000 W

F2 - Processing parameters
SI 32768
SF 100.6177975 MHz
WDW EM
SSB 0
LB 1.00 Hz
GB 0
PC 1.40

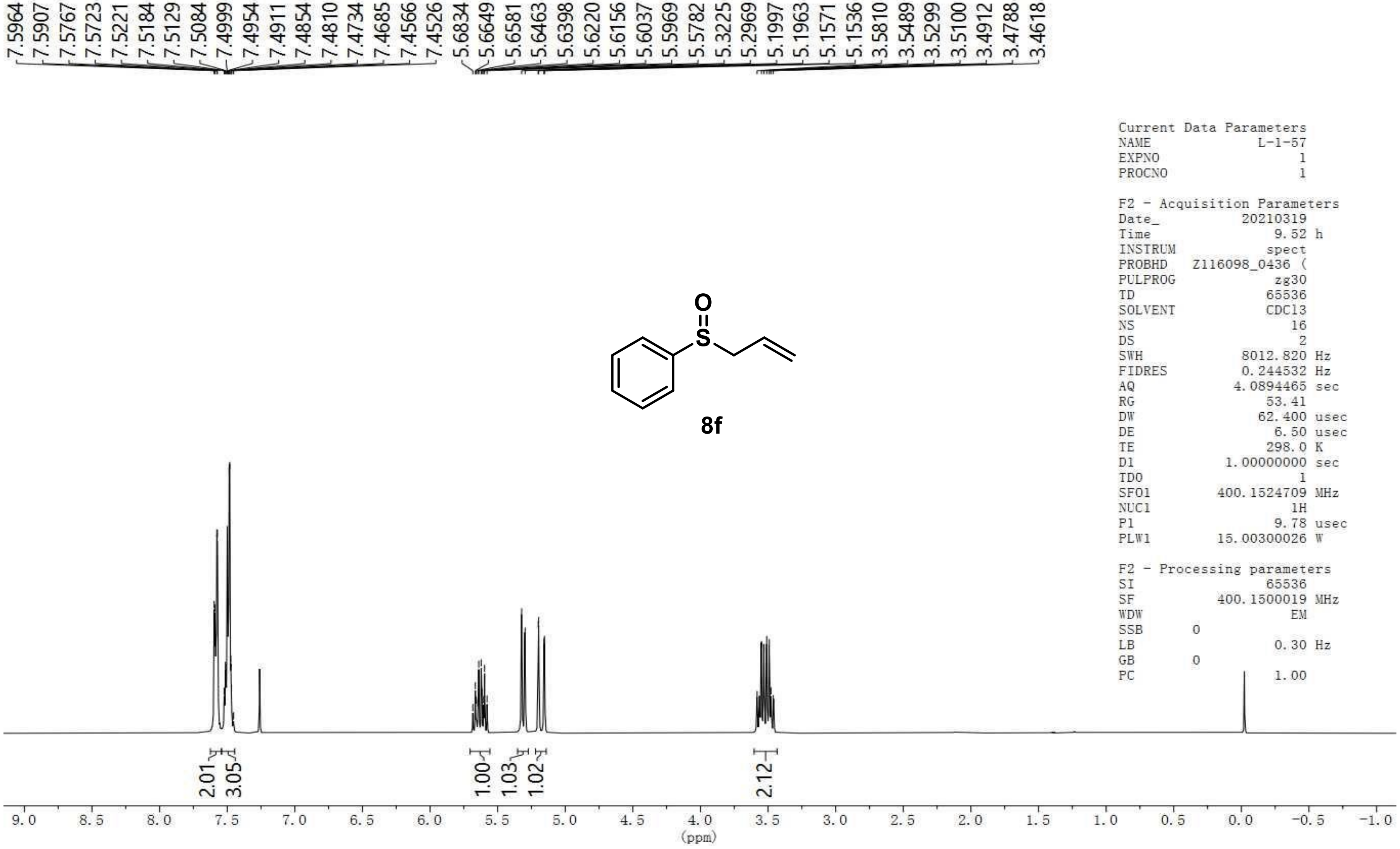
7.5964
7.5907
7.5767
7.5723
7.5221
7.5184
7.5129
7.5084
7.4999
7.4954
7.4911
7.4854
7.4810
7.4734
7.4685
7.4566
7.4526
5.6834
5.6649
5.6581
5.6463
5.6398
5.6220
5.6156
5.6037
5.5969
5.5782
5.3225
5.2969
5.1997
5.1963
5.1571
5.1536
3.5810
3.5489
3.5299
3.5100
3.4912
3.4788
3.4618
O
S
8f
Current Data Parameters
NAME L-1-57
EXPNO 1
PROCNO 1
F2 - Acquisition Parameters
Date_ 20210319
Time 9.52 h
INSTRUM spect
PROBHD Z116098_0436 (
PULPROG zg30
TD 65536
SOLVENT CDCl3
NS 16
DS 2
SWH 8012.820 Hz
FIDRES 0.244532 Hz
AQ 4.0894465 sec
RG 53.41
DW 62.400 usec
DE 6.50 usec
TE 298.0 K
D1 1.00000000 sec
TD0 1
SFO1 400.1524709 MHz
NUC1 1H
P1 9.78 usec
PLW1 15.00300026 W
F2 - Processing parameters
SI 65536
SF 400.1500019 MHz
WDW EM
SSB 0
LB 0.30 Hz
GB 0
PC 1.00
2.01
3.05
1.00
1.03
1.02
2.12
9.0 8.5 8.0 7.5 7.0 6.5 6.0 5.5 5.0 4.5 4.0 3.5 3.0 2.5 2.0 1.5 1.0 0.5 0.0 −0.5 −1.0
(ppm)

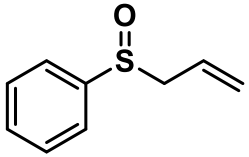

8f

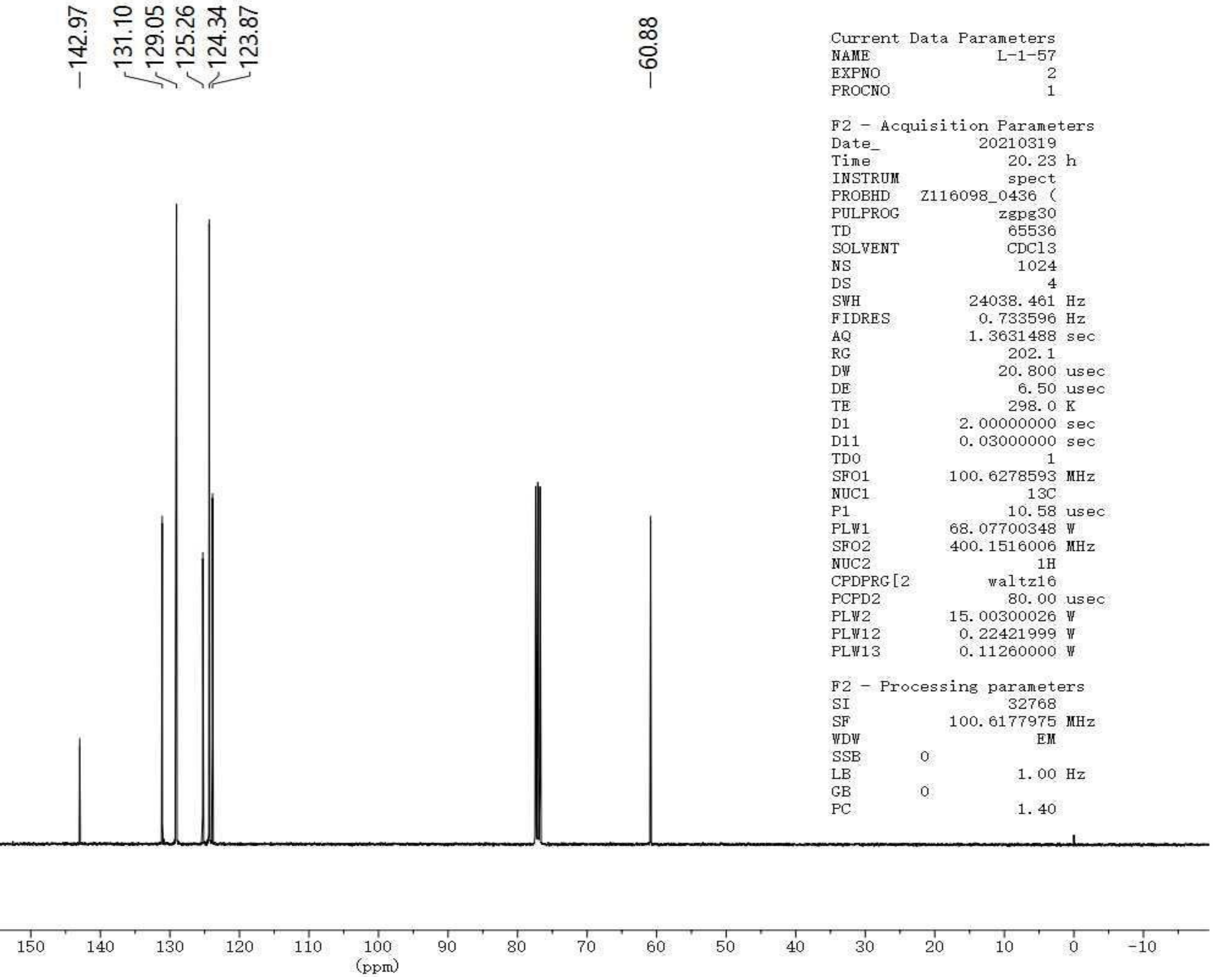

142.97
131.10
129.05
125.26
124.34
123.87
60.88
Current Data Parameters
NAME L-1-57
EXPNO 2
PROCNO 1
F2 - Acquisition Parameters
Date_ 20210319
Time 20.23 h
INSTRUM spect
PROBHD Z116098_0436 (
PULPROG zgpg30
TD 65536
SOLVENT CDCl3
NS 1024
DS 4
SWH 24038.461 Hz
FIDRES 0.733596 Hz
AQ 1.3631488 sec
RG 202.1
DW 20.800 usec
DE 6.50 usec
TE 298.0 K
D1 2.00000000 sec
D11 0.03000000 sec
TD0 1
SFO1 100.6278593 MHz
NUC1 13C
P1 10.58 usec
PLW1 68.07700348 W
SFO2 400.1516006 MHz
NUC2 1H
CPDPRG[2 waltz16
PCPD2 80.00 usec
PLW2 15.00300026 W
PLW12 0.22421999 W
PLW13 0.11260000 W
F2 - Processing parameters
SI 32768
SF 100.6177975 MHz
WDW EM
SSB 0
LB 1.00 Hz
GB 0
PC 1.40
210 200 190 180 170 160 150 140 130 120 110 100 90 80 70 60 50 40 30 20 10 0 -10
(ppm)

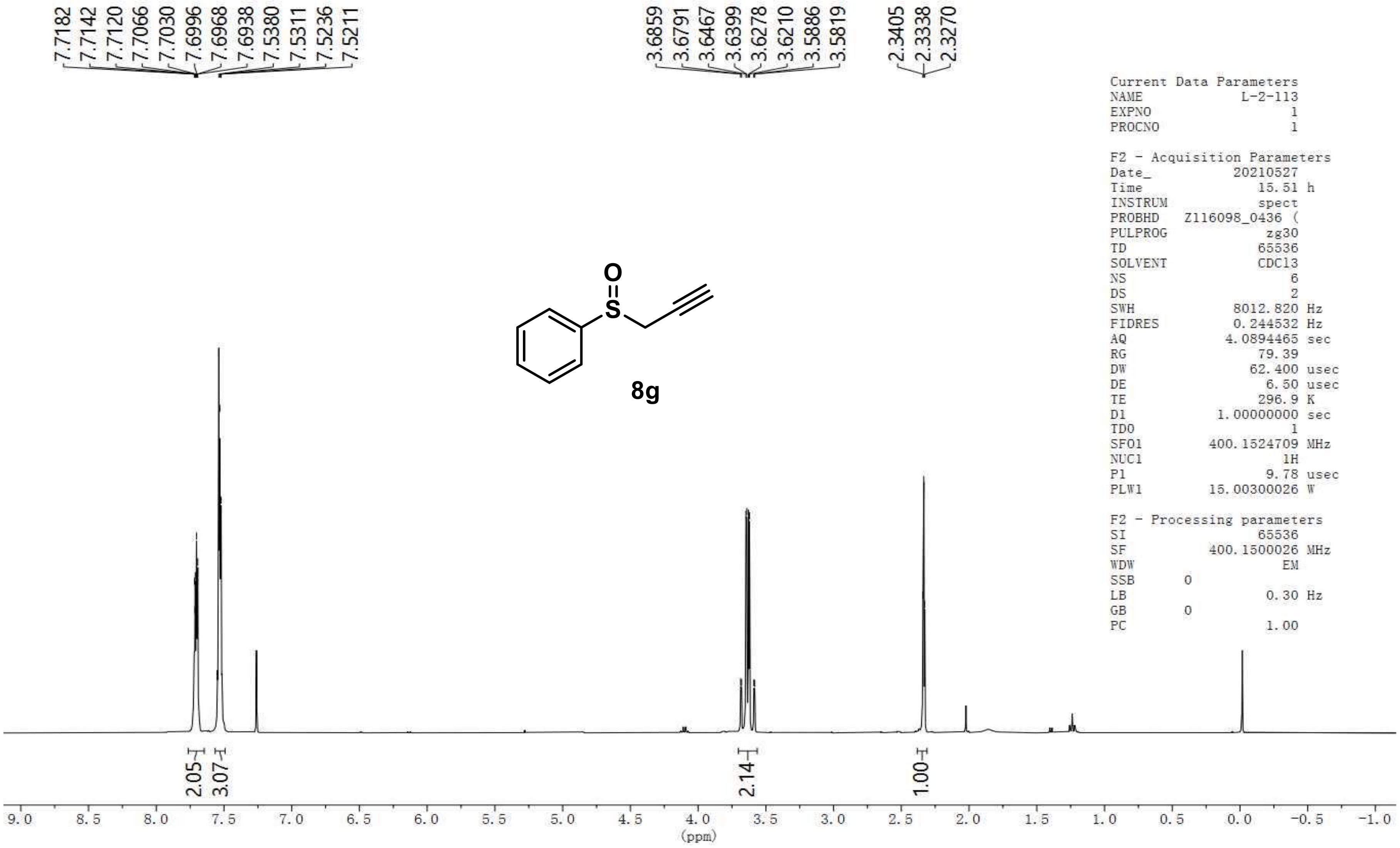
7.7182
7.7142
7.7120
7.7066
7.7030
7.6996
7.6968
7.6938
7.5380
7.5311
7.5236
7.5211
3.6859
3.6791
3.6467
3.6399
3.6278
3.6210
3.5886
3.5819
2.3405
2.3338
2.3270
O
S
8g
Current Data Parameters
NAME L-2-113
EXPNO 1
PROCNO 1
F2 - Acquisition Parameters
Date_ 20210527
Time 15.51 h
INSTRUM spect
PROBHD Z116098_0436 (
PULPROG zg30
TD 65536
SOLVENT CDCl3
NS 6
DS 2
SWH 8012.820 Hz
FIDRES 0.244532 Hz
AQ 4.0894465 sec
RG 79.39
DW 62.400 usec
DE 6.50 usec
TE 296.9 K
D1 1.00000000 sec
TD0 1
SFO1 400.1524709 MHz
NUC1 1H
P1 9.78 usec
PLW1 15.00300026 W
F2 - Processing parameters
SI 65536
SF 400.1500026 MHz
WDW EM
SSB 0
LB 0.30 Hz
GB 0
PC 1.00
2.05
3.07
2.14
1.00
9.0 8.5 8.0 7.5 7.0 6.5 6.0 5.5 5.0 4.5 4.0 3.5 3.0 2.5 2.0 1.5 1.0 0.5 0.0 −0.5 −1.0
(ppm)

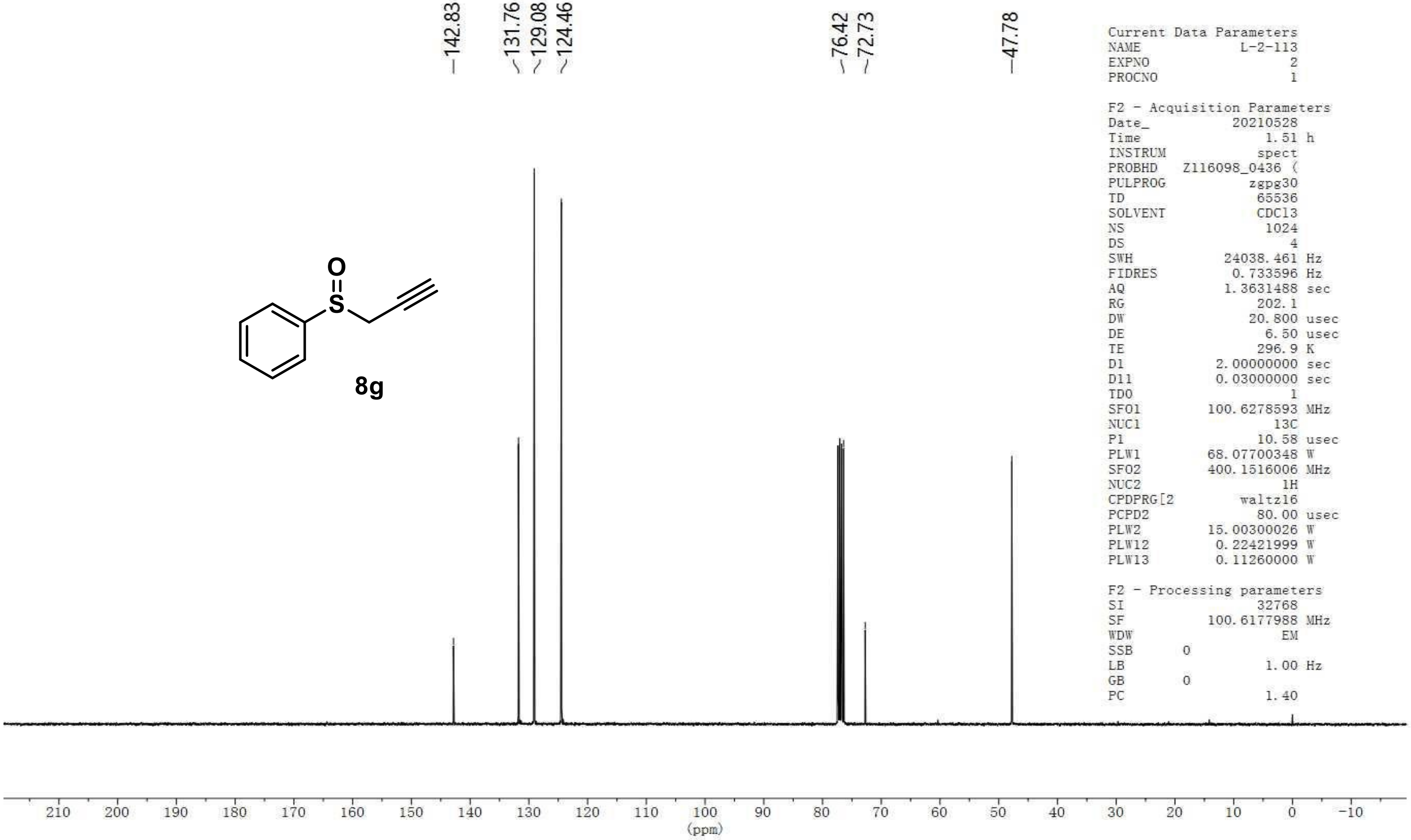

8g

Current Data Parameters
NAME L-2-113
EXPNO 2
PROCNO 1

F2 - Acquisition Parameters
Date_ 20210528
Time 1.51 h
INSTRUM spect
PROBHD Z116098_0436 (
PULPROG zgpg30
TD 65536
SOLVENT CDCl3
NS 1024
DS 4
SWH 24038.461 Hz
FIDRES 0.733596 Hz
AQ 1.3631488 sec
RG 202.1
DW 20.800 usec
DE 6.50 usec
TE 296.9 K
D1 2.00000000 sec
D11 0.03000000 sec
TD0 1
SFO1 100.6278593 MHz
NUC1 13C
P1 10.58 usec
PLW1 68.07700348 W
SFO2 400.1516006 MHz
NUC2 1H
CPDPRG[2 waltz16
PCPD2 80.00 usec
PLW2 15.00300026 W
PLW12 0.22421999 W
PLW13 0.11260000 W

F2 - Processing parameters
SI 32768
SF 100.6177988 MHz
WDW EM
SSB 0
LB 1.00 Hz
GB 0
PC 1.40

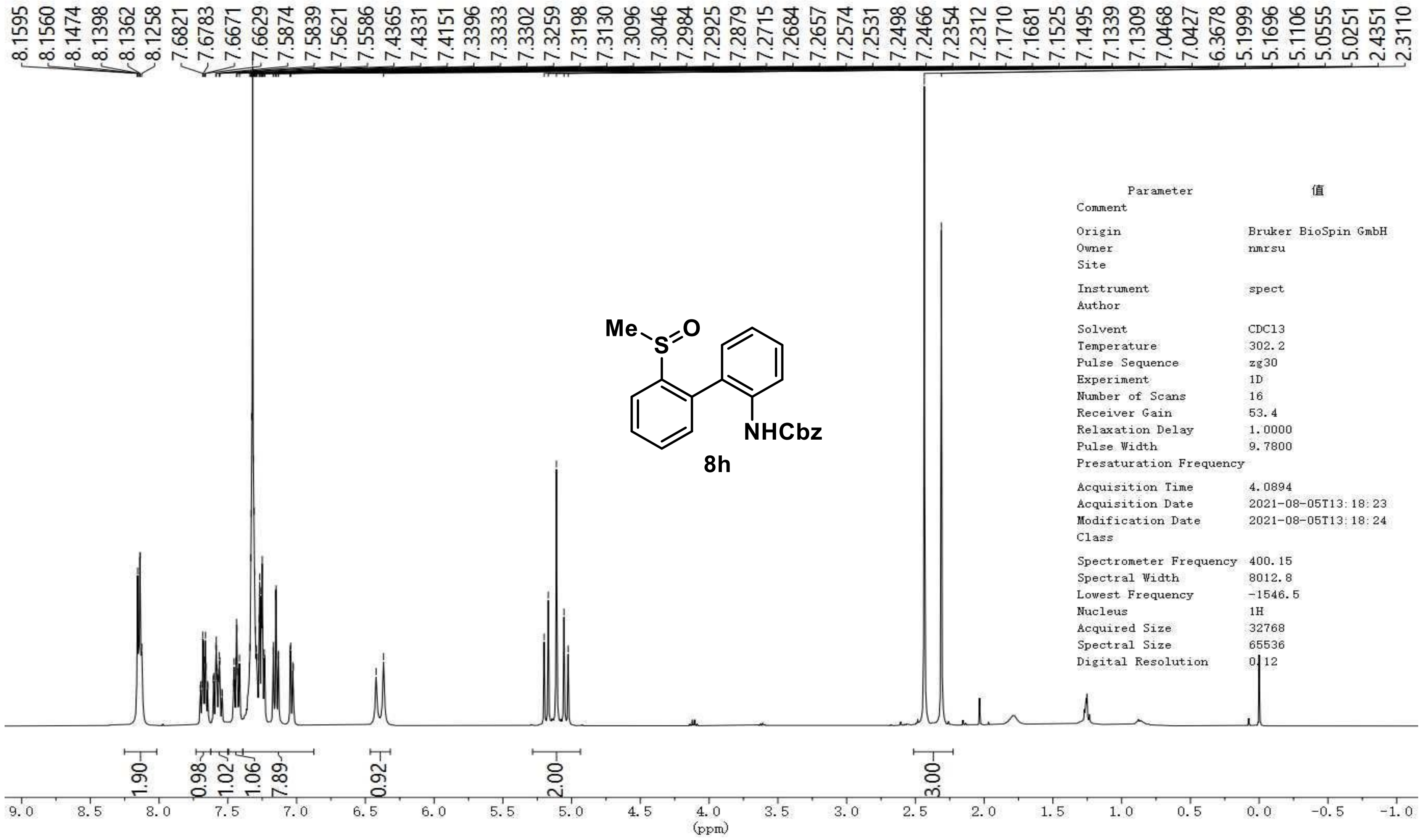
8.1595
8.1560
8.1474
8.1398
8.1362
8.1258
7.6821
7.6783
7.6671
7.6629
7.5874
7.5839
7.5621
7.5586
7.4365
7.4331
7.4151
7.3396
7.3333
7.3302
7.3259
7.3198
7.3130
7.3096
7.3046
7.2984
7.2925
7.2879
7.2715
7.2684
7.2657
7.2574
7.2531
7.2498
7.2466
7.2354
7.2312
7.1710
7.1681
7.1525
7.1495
7.1339
7.1309
7.0468
7.0427
6.3678
5.1999
5.1696
5.1106
5.0555
5.0251
2.4351
2.3110
Me
S
O
NHCbz
8h
Parameter 值
Comment
Origin Bruker BioSpin GmbH
Owner nmrsu
Site
Instrument spect
Author
Solvent CDCl3
Temperature 302.2
Pulse Sequence zg30
Experiment 1D
Number of Scans 16
Receiver Gain 53.4
Relaxation Delay 1.0000
Pulse Width 9.7800
Presaturation Frequency
Acquisition Time 4.0894
Acquisition Date 2021-08-05T13:18:23
Modification Date 2021-08-05T13:18:24
Class
Spectrometer Frequency 400.15
Spectral Width 8012.8
Lowest Frequency -1546.5
Nucleus 1H
Acquired Size 32768
Spectral Size 65536
Digital Resolution 0.12
1.90
0.98
1.02
1.06
7.89
0.92
2.00
3.00
9.0 8.5 8.0 7.5 7.0 6.5 6.0 5.5 5.0 4.5 4.0 3.5 3.0 2.5 2.0 1.5 1.0 0.5 0.0 -0.5 -1.0
(ppm)

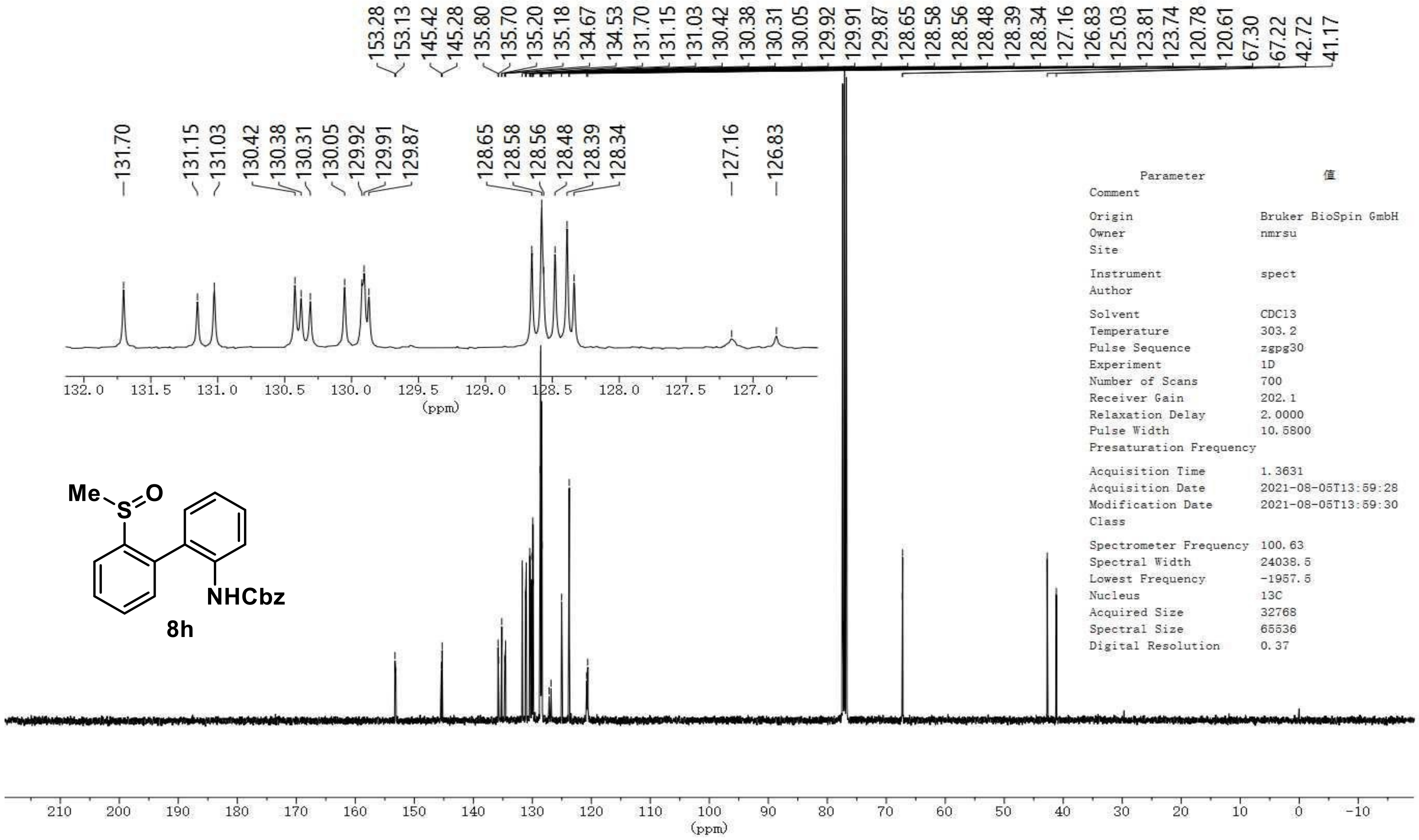
153.28
153.13
145.42
145.28
135.80
135.70
135.20
135.18
134.67
134.53
131.70
131.15
131.03
130.42
130.38
130.31
130.05
129.92
129.91
129.87
128.65
128.58
128.56
128.48
128.39
128.34
127.16
126.83
125.03
123.81
123.74
120.78
120.61
67.30
67.22
42.72
41.17
132.0 131.5 131.0 130.5 130.0 129.5 129.0 128.5 128.0 127.5 127.0
(ppm)
Parameter 值
Comment
Origin Bruker BioSpin GmbH
Owner nmrsu
Site
Instrument spect
Author
Solvent CDCl3
Temperature 303.2
Pulse Sequence zgpg30
Experiment 1D
Number of Scans 700
Receiver Gain 202.1
Relaxation Delay 2.0000
Pulse Width 10.5800
Presaturation Frequency
Acquisition Time 1.3631
Acquisition Date 2021-08-05T13:59:28
Modification Date 2021-08-05T13:59:30
Class
Spectrometer Frequency 100.63
Spectral Width 24038.5
Lowest Frequency -1957.5
Nucleus 13C
Acquired Size 32768
Spectral Size 65536
Digital Resolution 0.37
Me
S
O
NHCbz
8h
210 200 190 180 170 160 150 140 130 120 110 100 90 80 70 60 50 40 30 20 10 0 -10
(ppm)

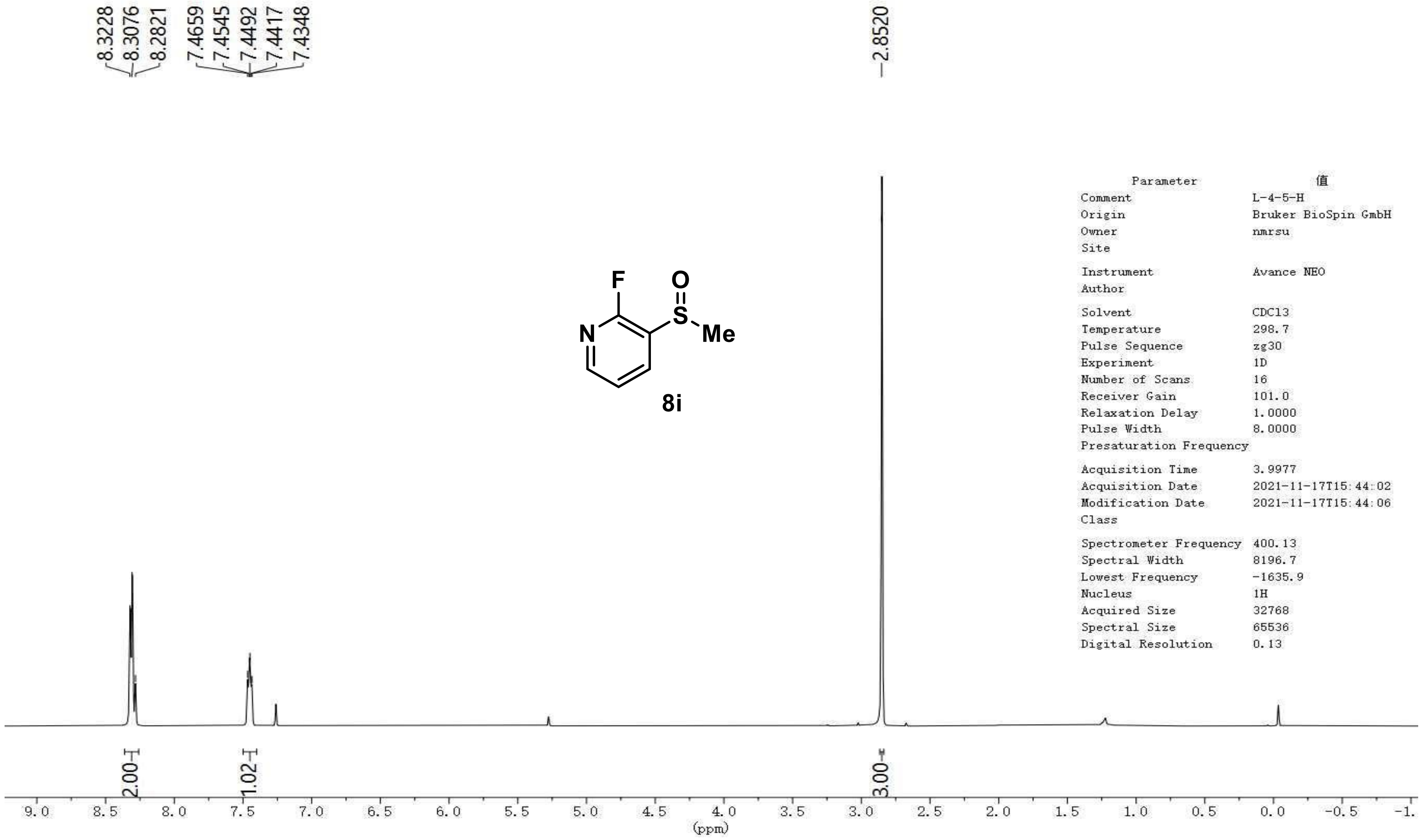
8.3228
8.3076
8.2821
7.4659
7.4545
7.4492
7.4417
7.4348
2.8520
F
O
N
S
Me
8i
Parameter 値
Comment L-4-5-H
Origin Bruker BioSpin GmbH
Owner nmrsu
Site
Instrument Avance NEO
Author
Solvent CDCl3
Temperature 298.7
Pulse Sequence zg30
Experiment 1D
Number of Scans 16
Receiver Gain 101.0
Relaxation Delay 1.0000
Pulse Width 8.0000
Presaturation Frequency
Acquisition Time 3.9977
Acquisition Date 2021-11-17T15:44:02
Modification Date 2021-11-17T15:44:06
Class
Spectrometer Frequency 400.13
Spectral Width 8196.7
Lowest Frequency -1635.9
Nucleus 1H
Acquired Size 32768
Spectral Size 65536
Digital Resolution 0.13
2.00
1.02
3.00
9.0 8.5 8.0 7.5 7.0 6.5 6.0 5.5 5.0 4.5 4.0 3.5 3.0 2.5 2.0 1.5 1.0 0.5 0.0 -0.5 -1.
(ppm)

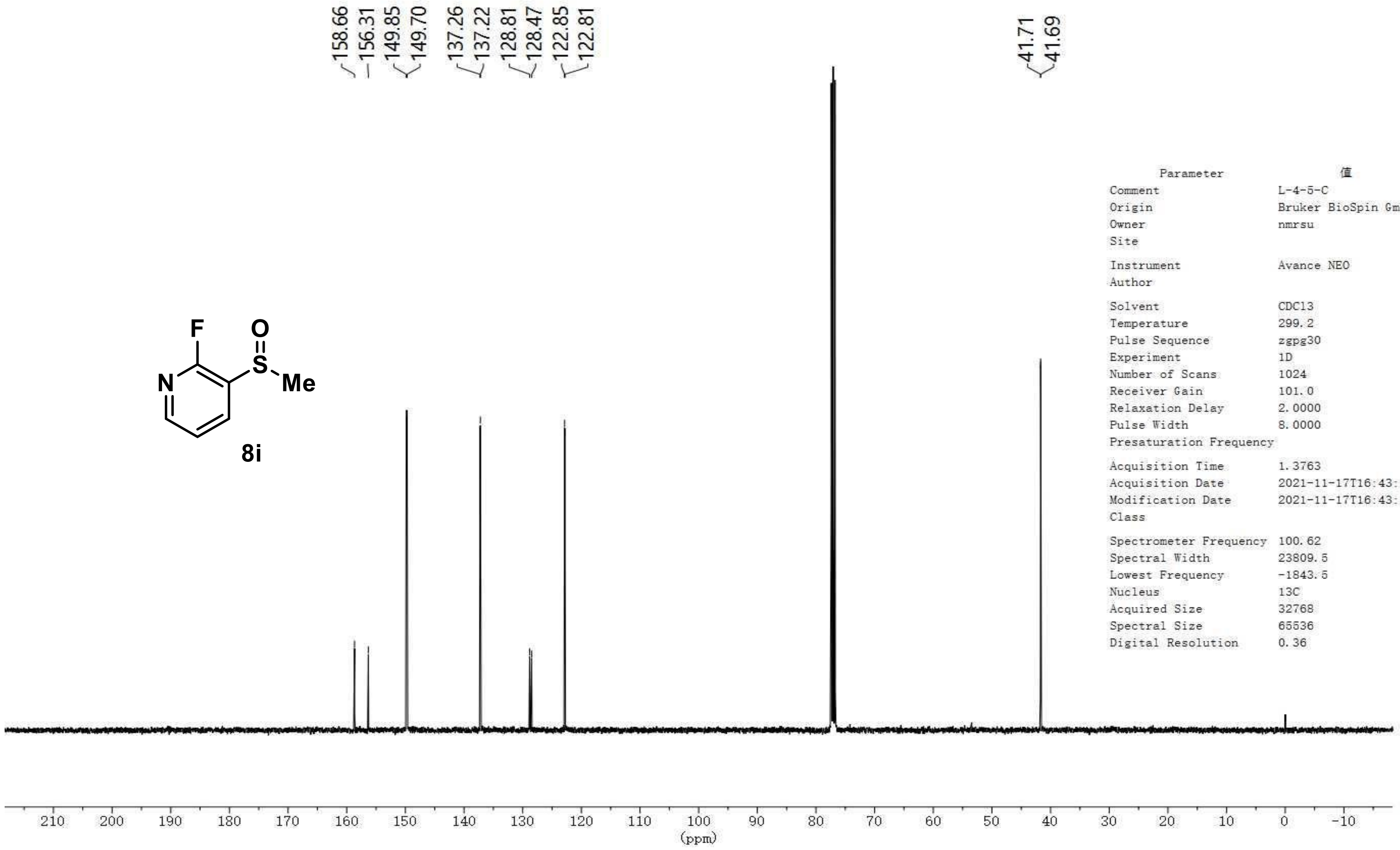

8i

Parameter	值
Comment	L-4-5-C
Origin	Bruker BioSpin GmbH
Owner	nmrsu
Site	
Instrument	Avance NEO
Author	
Solvent	CDCl3
Temperature	299.2
Pulse Sequence	zgpg30
Experiment	1D
Number of Scans	1024
Receiver Gain	101.0
Relaxation Delay	2.0000
Pulse Width	8.0000
Presaturation Frequency	
Acquisition Time	1.3763
Acquisition Date	2021-11-17T16:43:12
Modification Date	2021-11-17T16:43:16
Class	
Spectrometer Frequency	100.62
Spectral Width	23809.5
Lowest Frequency	-1843.5
Nucleus	13C
Acquired Size	32768
Spectral Size	65536
Digital Resolution	0.36

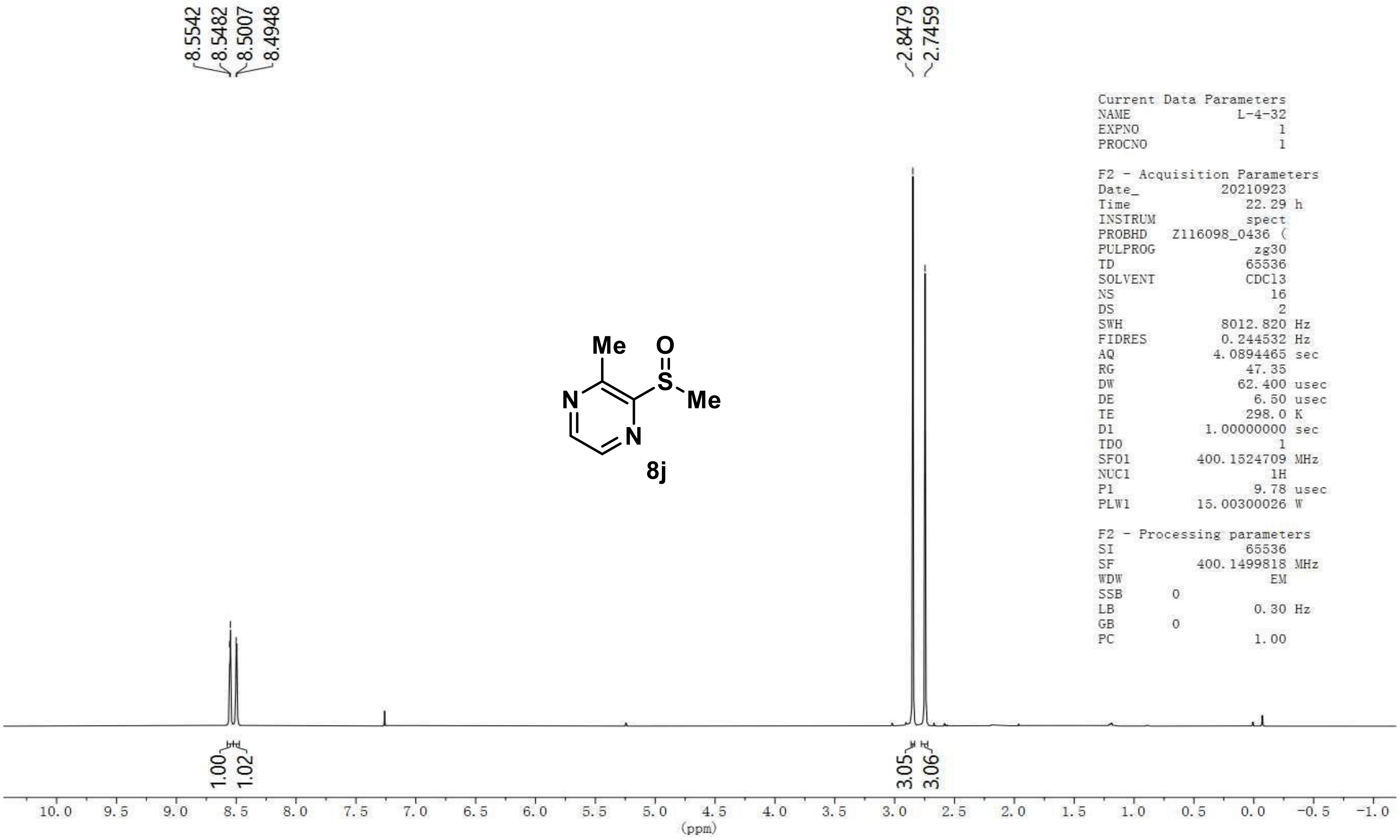

8.5542
8.5482
8.5007
8.4948
2.8479
2.7459
Me
O
S
Me
N
N
8j
Current Data Parameters
NAME L-4-32
EXPNO 1
PROCNO 1
F2 - Acquisition Parameters
Date_ 20210923
Time 22.29 h
INSTRUM spect
PROBHD Z116098_0436 (
PULPROG zg30
TD 65536
SOLVENT CDCl3
NS 16
DS 2
SWH 8012.820 Hz
FIDRES 0.244532 Hz
AQ 4.0894465 sec
RG 47.35
DW 62.400 usec
DE 6.50 usec
TE 298.0 K
D1 1.00000000 sec
TD0 1
SFO1 400.1524709 MHz
NUC1 1H
P1 9.78 usec
PLW1 15.00300026 W
F2 - Processing parameters
SI 65536
SF 400.1499818 MHz
WDW EM
SSB 0
LB 0.30 Hz
GB 0
PC 1.00
1.00
1.02
3.05
3.06
10.0 9.5 9.0 8.5 8.0 7.5 7.0 6.5 6.0 5.5 5.0 4.5 4.0 3.5 3.0 2.5 2.0 1.5 1.0 0.5 0.0 -0.5 -1.0
(ppm)

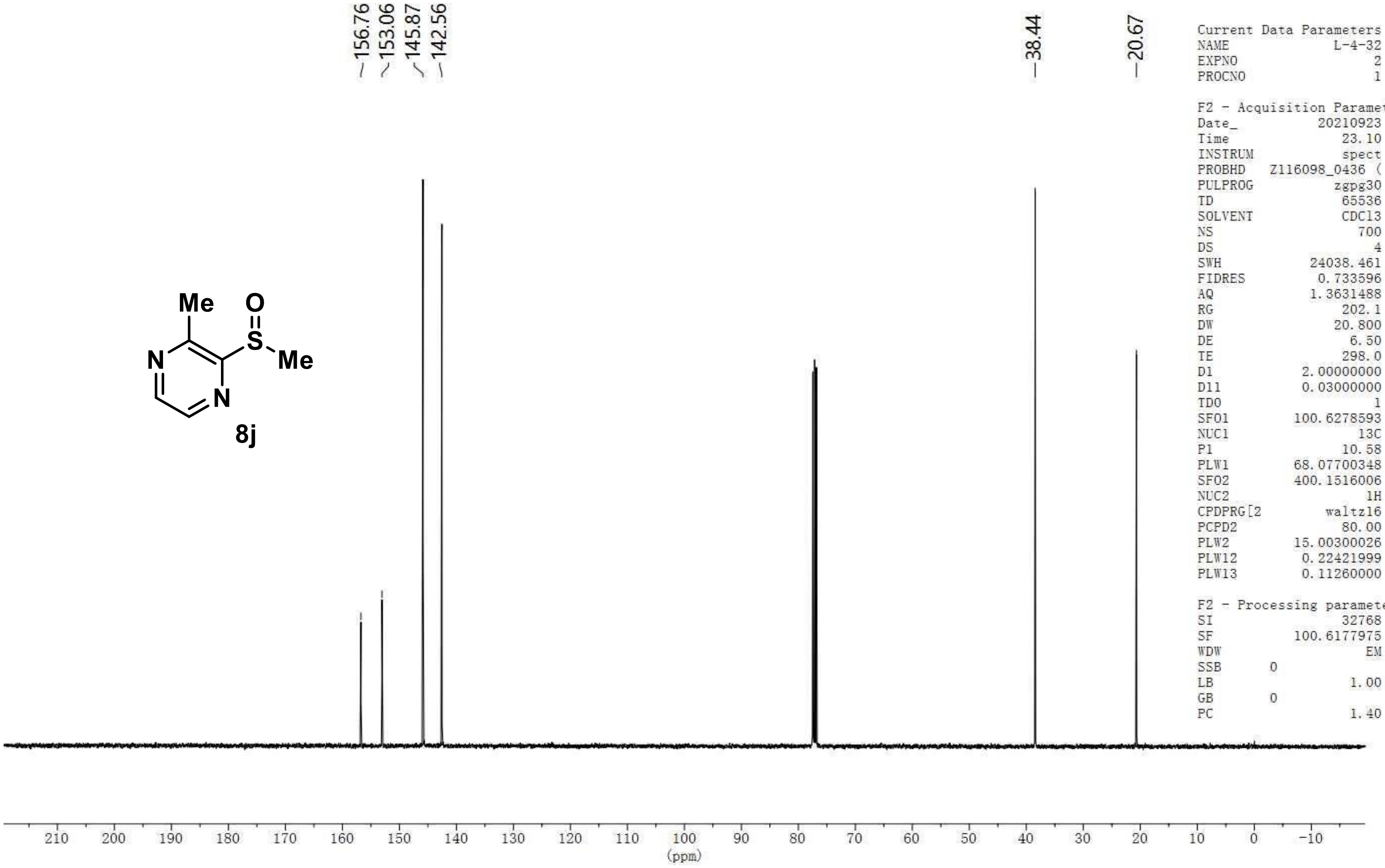


```
Current Data Parameters
NAME              L-4-32
EXPNO                  2
PROCNO                 1

F2 - Acquisition Parameters
Date_           20210923
Time               23.10 h
INSTRUM            spect
PROBHD   Z116098_0436 (
PULPROG           zgpg30
TD                 65536
SOLVENT            CDCl3
NS                   700
DS                     4
SWH            24038.461 Hz
FIDRES          0.733596 Hz
AQ             1.3631488 sec
RG                 202.1
DW                20.800 usec
DE                  6.50 usec
TE                 298.0 K
D1            2.00000000 sec
D11           0.03000000 sec
TD0                    1
SFO1         100.6278593 MHz
NUC1                 13C
P1                 10.58 usec
PLW1         68.07700348 W
SFO2         400.1516006 MHz
NUC2                  1H
CPDPRG[2         waltz16
PCPD2              80.00 usec
PLW2         15.00300026 W
PLW12         0.22421999 W
PLW13         0.11260000 W

F2 - Processing parameters
SI                 32768
SF           100.6177975 MHz
WDW                   EM
SSB      0
LB                  1.00 Hz
GB       0
PC                  1.40
```

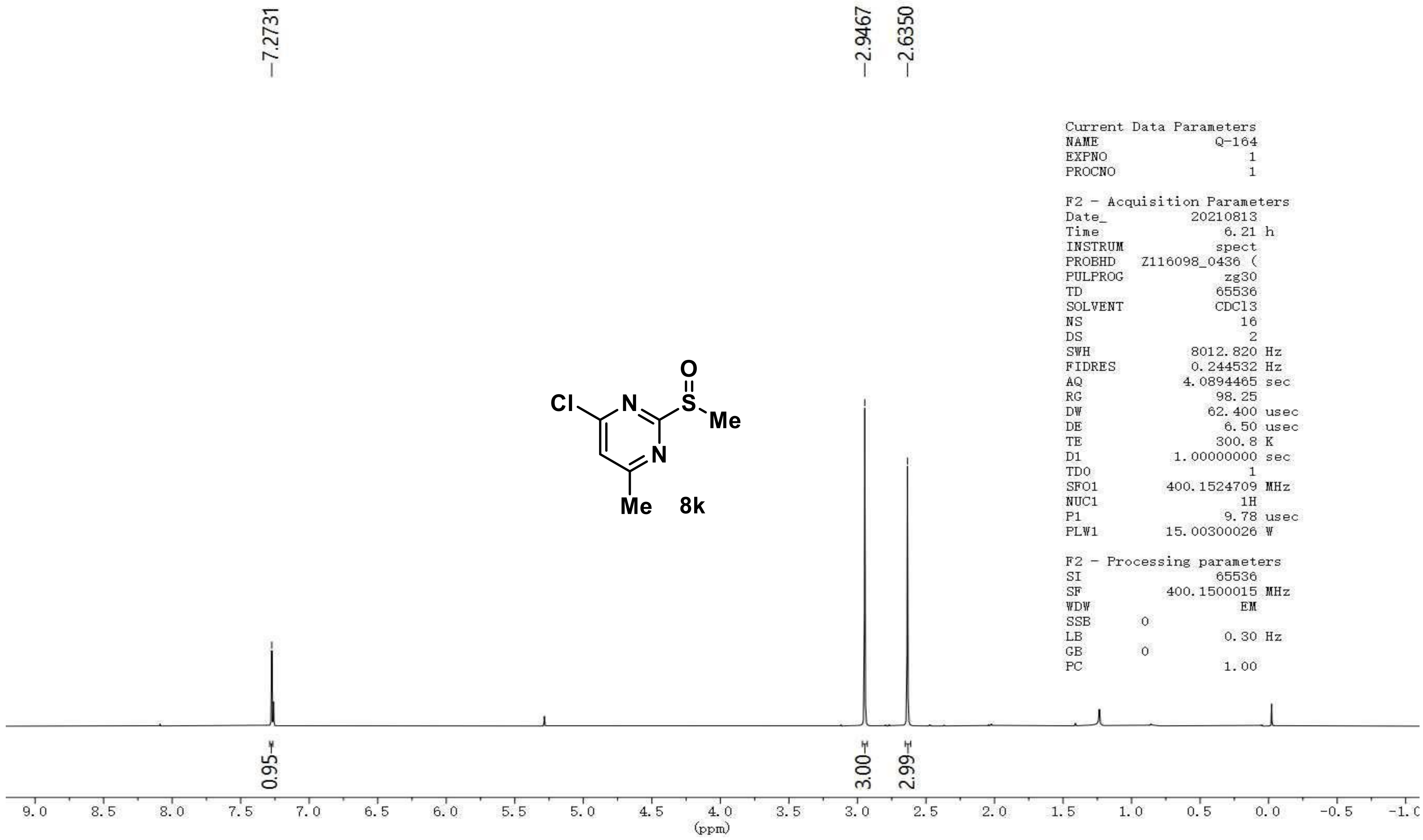

7.2731
2.9467
2.6350
Current Data Parameters
NAME Q-164
EXPNO 1
PROCNO 1
F2 - Acquisition Parameters
Date_ 20210813
Time 6.21 h
INSTRUM spect
PROBHD Z116098_0436 (
PULPROG zg30
TD 65536
SOLVENT CDCl3
NS 16
DS 2
SWH 8012.820 Hz
FIDRES 0.244532 Hz
AQ 4.0894465 sec
RG 98.25
DW 62.400 usec
DE 6.50 usec
TE 300.8 K
D1 1.00000000 sec
TD0 1
SFO1 400.1524709 MHz
NUC1 1H
P1 9.78 usec
PLW1 15.00300026 W
F2 - Processing parameters
SI 65536
SF 400.1500015 MHz
WDW EM
SSB 0
LB 0.30 Hz
GB 0
PC 1.00
Cl
N
N
S
O
Me
Me
8k
0.95
3.00
2.99
9.0 8.5 8.0 7.5 7.0 6.5 6.0 5.5 5.0 4.5 4.0 3.5 3.0 2.5 2.0 1.5 1.0 0.5 0.0 -0.5 -1.0
(ppm)

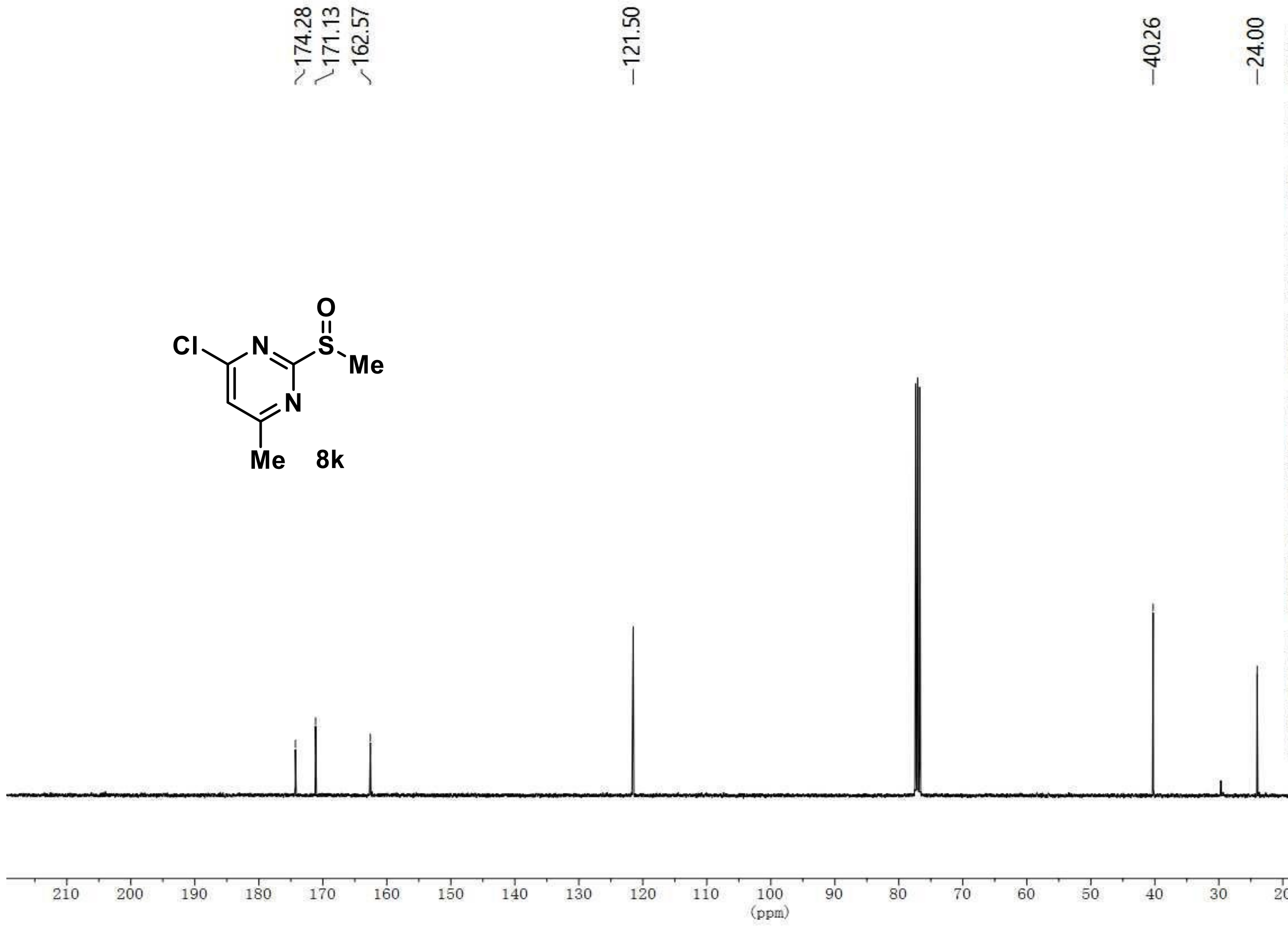

Current Data Parameters
NAME Q-164
EXPNO 2
PROCNO 1

F2 - Acquisition Parameters
Date_ 20210813
Time 7.20 h
INSTRUM spect
PROBHD Z116098_0436 (
PULPROG zgpg30
TD 65536
SOLVENT CDCl3
NS 1024
DS 4
SWH 24038.461 Hz
FIDRES 0.733596 Hz
AQ 1.3631488 sec
RG 202.1
DW 20.800 usec
DE 6.50 usec
TE 301.4 K
D1 2.00000000 sec
D11 0.03000000 sec
TD0 1
SFO1 100.6278593 MHz
NUC1 13C
P1 10.58 usec
PLW1 68.07700348 W
SFO2 400.1516006 MHz
NUC2 1H
CPDPRG[2 waltz16
PCPD2 80.00 usec
PLW2 15.00300026 W
PLW12 0.22421999 W
PLW13 0.11260000 W

F2 - Processing parameters
SI 32768
SF 100.6177975 MHz
WDW EM
SSB 0
LB 1.00 Hz
GB 0
PC 1.40

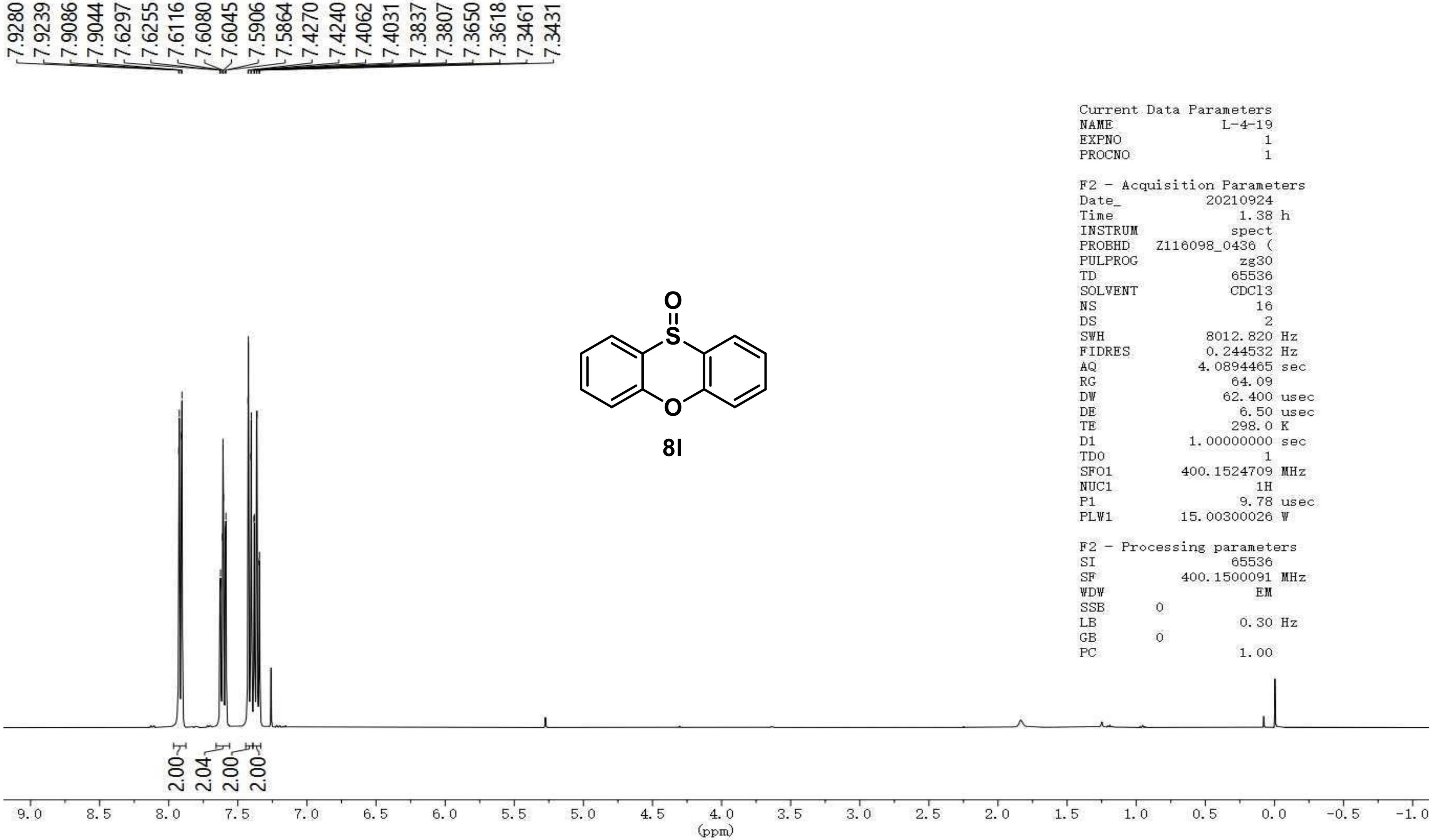

8l

```
Current Data Parameters
NAME              L-4-19
EXPNO                  1
PROCNO                 1

F2 - Acquisition Parameters
Date_           20210924
Time                1.38 h
INSTRUM            spect
PROBHD    Z116098_0436 (
PULPROG             zg30
TD                 65536
SOLVENT            CDCl3
NS                    16
DS                     2
SWH             8012.820 Hz
FIDRES          0.244532 Hz
AQ             4.0894465 sec
RG                 64.09
DW                62.400 usec
DE                  6.50 usec
TE                 298.0 K
D1            1.00000000 sec
TD0                    1
SFO1         400.1524709 MHz
NUC1                  1H
P1                  9.78 usec
PLW1         15.00300026 W

F2 - Processing parameters
SI                 65536
SF           400.1500091 MHz
WDW                   EM
SSB       0
LB                  0.30 Hz
GB        0
PC                  1.00
```

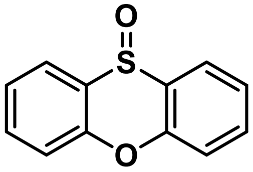

8l

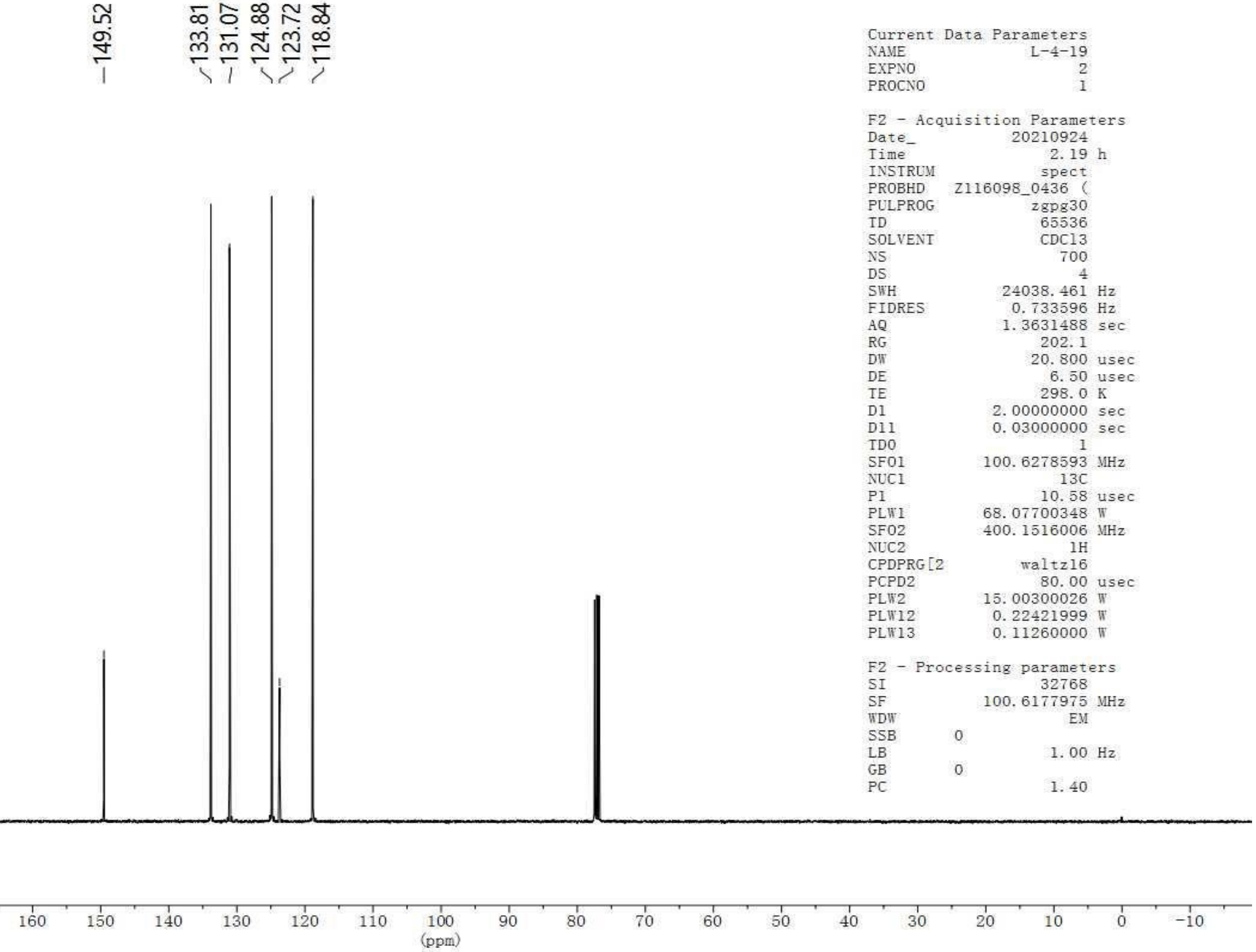
149.52
133.81
131.07
124.88
123.72
118.84
Current Data Parameters
NAME L-4-19
EXPNO 2
PROCNO 1
F2 - Acquisition Parameters
Date_ 20210924
Time 2.19 h
INSTRUM spect
PROBHD Z116098_0436 (
PULPROG zgpg30
TD 65536
SOLVENT CDCl3
NS 700
DS 4
SWH 24038.461 Hz
FIDRES 0.733596 Hz
AQ 1.3631488 sec
RG 202.1
DW 20.800 usec
DE 6.50 usec
TE 298.0 K
D1 2.00000000 sec
D11 0.03000000 sec
TD0 1
SFO1 100.6278593 MHz
NUC1 13C
P1 10.58 usec
PLW1 68.07700348 W
SFO2 400.1516006 MHz
NUC2 1H
CPDPRG[2 waltz16
PCPD2 80.00 usec
PLW2 15.00300026 W
PLW12 0.22421999 W
PLW13 0.11260000 W
F2 - Processing parameters
SI 32768
SF 100.6177975 MHz
WDW EM
SSB 0
LB 1.00 Hz
GB 0
PC 1.40
210 200 190 180 170 160 150 140 130 120 110 100 90 80 70 60 50 40 30 20 10 0 −10
(ppm)

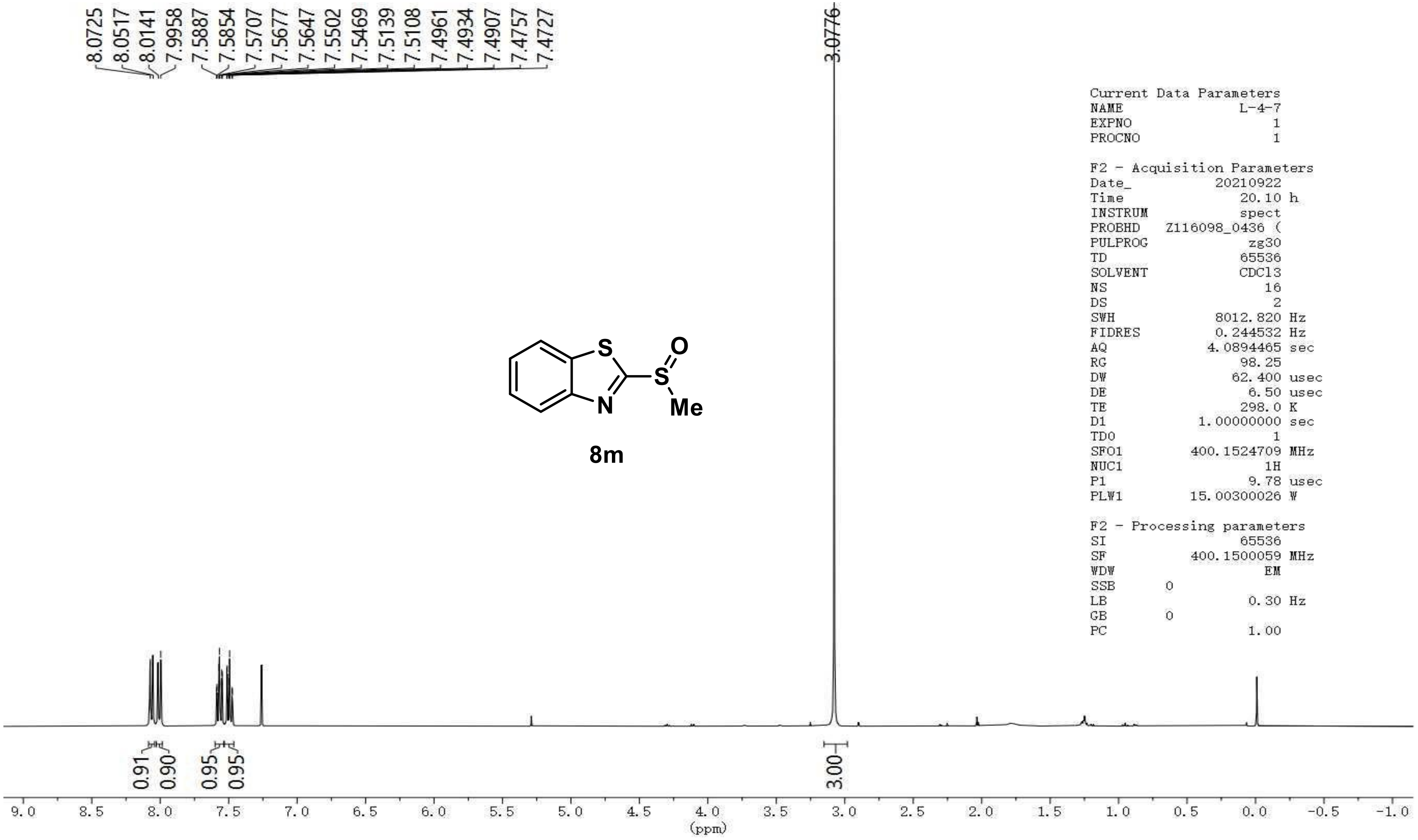

8.0725
8.0517
8.0141
7.9958
7.5887
7.5854
7.5707
7.5677
7.5647
7.5502
7.5469
7.5139
7.5108
7.4961
7.4934
7.4907
7.4757
7.4727
3.0776
S
O
S
N
Me
8m
Current Data Parameters
NAME L-4-7
EXPNO 1
PROCNO 1
F2 - Acquisition Parameters
Date_ 20210922
Time 20.10 h
INSTRUM spect
PROBHD Z116098_0436 (
PULPROG zg30
TD 65536
SOLVENT CDCl3
NS 16
DS 2
SWH 8012.820 Hz
FIDRES 0.244532 Hz
AQ 4.0894465 sec
RG 98.25
DW 62.400 usec
DE 6.50 usec
TE 298.0 K
D1 1.00000000 sec
TD0 1
SFO1 400.1524709 MHz
NUC1 1H
P1 9.78 usec
PLW1 15.00300026 W
F2 - Processing parameters
SI 65536
SF 400.1500059 MHz
WDW EM
SSB 0
LB 0.30 Hz
GB 0
PC 1.00
0.91
0.90
0.95
0.95
3.00
9.0 8.5 8.0 7.5 7.0 6.5 6.0 5.5 5.0 4.5 4.0 3.5 3.0 2.5 2.0 1.5 1.0 0.5 0.0 -0.5 -1.0
(ppm)

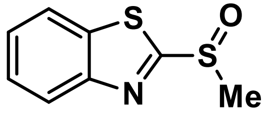

8m

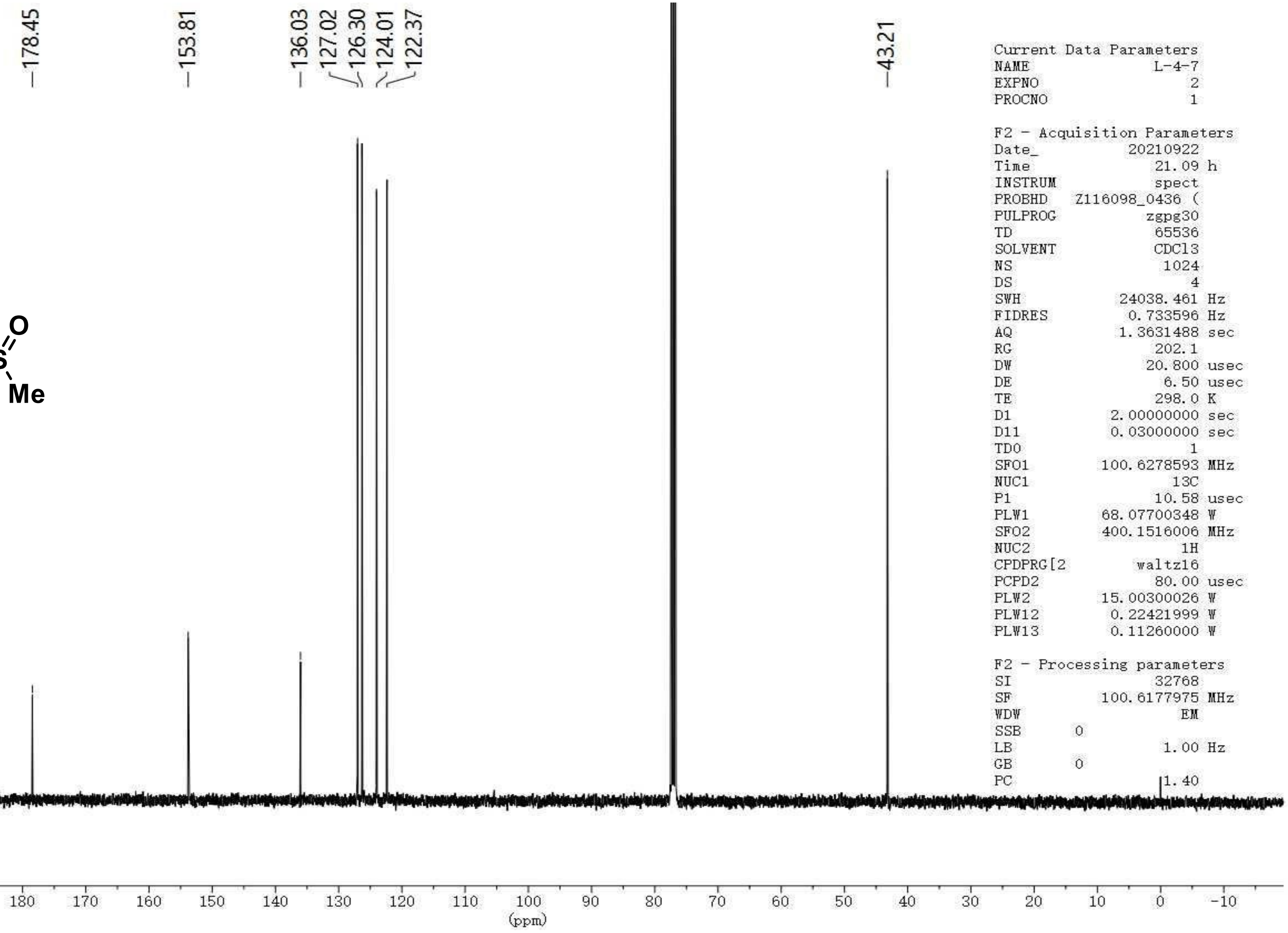

Current Data Parameters

NAME	L-4-7
EXPNO	2
PROCNO	1

F2 - Acquisition Parameters

Date_	20210922
Time	21.09 h
INSTRUM	spect
PROBHD	Z116098_0436 (
PULPROG	zgpg30
TD	65536
SOLVENT	CDCl3
NS	1024
DS	4
SWH	24038.461 Hz
FIDRES	0.733596 Hz
AQ	1.3631488 sec
RG	202.1
DW	20.800 usec
DE	6.50 usec
TE	298.0 K
D1	2.00000000 sec
D11	0.03000000 sec
TD0	1
SFO1	100.6278593 MHz
NUC1	13C
P1	10.58 usec
PLW1	68.07700348 W
SFO2	400.1516006 MHz
NUC2	1H
CPDPRG[2	waltz16
PCPD2	80.00 usec
PLW2	15.00300026 W
PLW12	0.22421999 W
PLW13	0.11260000 W

F2 - Processing parameters

SI	32768
SF	100.6177975 MHz
WDW	EM
SSB	0
LB	1.00 Hz
GB	0
PC	1.40

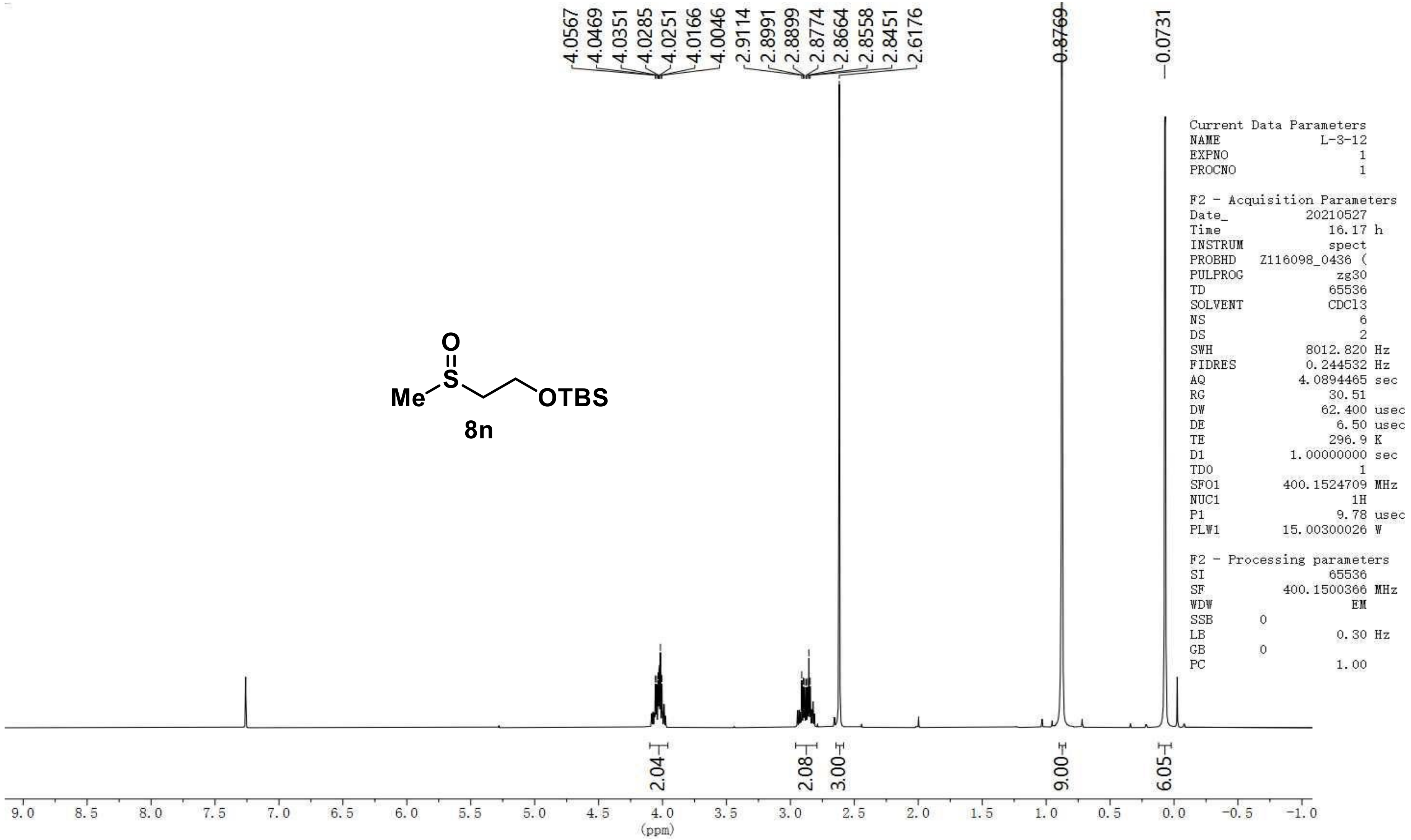
4.0567
4.0469
4.0351
4.0285
4.0251
4.0166
4.0046
2.9114
2.8991
2.8899
2.8774
2.8664
2.8558
2.8451
2.6176
0.8769
-0.0731
Me
S
O
OTBS
8n
Current Data Parameters
NAME L-3-12
EXPNO 1
PROCNO 1
F2 - Acquisition Parameters
Date_ 20210527
Time 16.17 h
INSTRUM spect
PROBHD Z116098_0436 (
PULPROG zg30
TD 65536
SOLVENT CDCl3
NS 6
DS 2
SWH 8012.820 Hz
FIDRES 0.244532 Hz
AQ 4.0894465 sec
RG 30.51
DW 62.400 usec
DE 6.50 usec
TE 296.9 K
D1 1.00000000 sec
TD0 1
SFO1 400.1524709 MHz
NUC1 1H
P1 9.78 usec
PLW1 15.00300026 W
F2 - Processing parameters
SI 65536
SF 400.1500366 MHz
WDW EM
SSB 0
LB 0.30 Hz
GB 0
PC 1.00
2.04
2.08
3.00
9.00
6.05
9.0 8.5 8.0 7.5 7.0 6.5 6.0 5.5 5.0 4.5 4.0 3.5 3.0 2.5 2.0 1.5 1.0 0.5 0.0 -0.5 -1.0
(ppm)

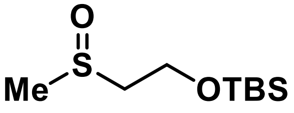

8n

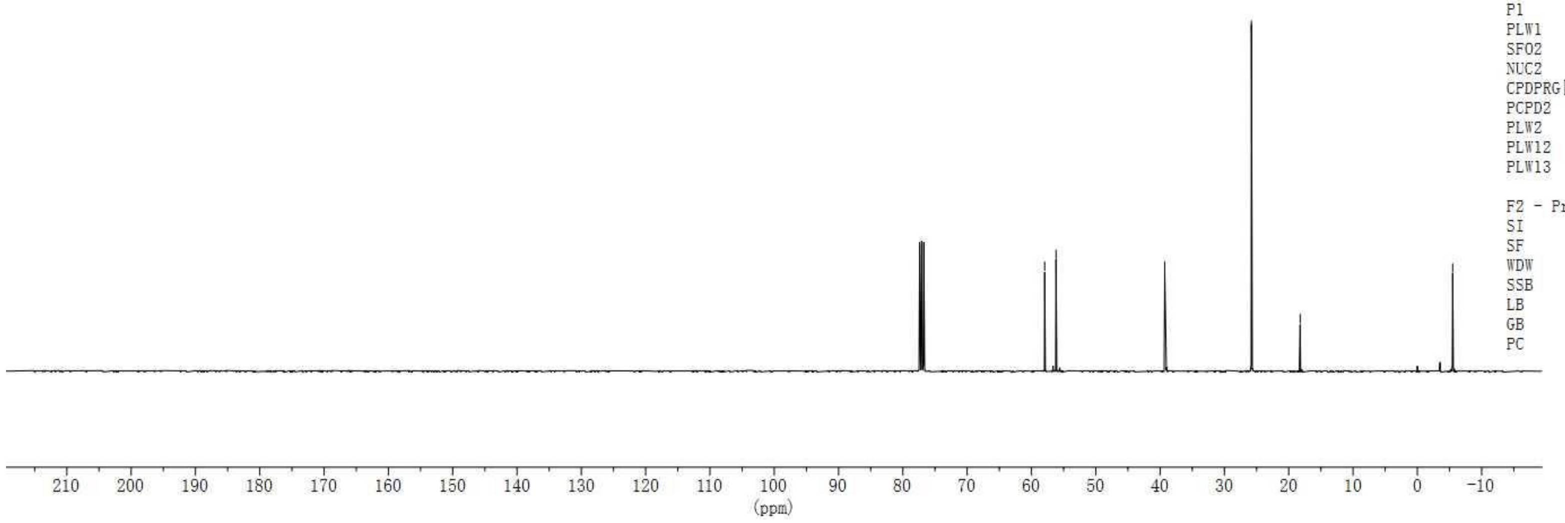

Current Data Parameters
NAME L-3-12
EXPNO 2
PROCNO 1

F2 - Acquisition Parameters
Date_ 20210528
Time 8.08 h
INSTRUM spect
PROBHD Z116098_0436 (
PULPROG zgpg30
TD 65536
SOLVENT CDCl3
NS 1024
DS 4
SWH 24038.461 Hz
FIDRES 0.733596 Hz
AQ 1.3631488 sec
RG 202.1
DW 20.800 usec
DE 6.50 usec
TE 296.9 K
D1 2.00000000 sec
D11 0.03000000 sec
TD0 1
SFO1 100.6278593 MHz
NUC1 13C
P1 10.58 usec
PLW1 68.07700348 W
SFO2 400.1516006 MHz
NUC2 1H
CPDPRG[2 waltz16
PCPD2 80.00 usec
PLW2 15.00300026 W
PLW12 0.22421999 W
PLW13 0.11260000 W

F2 - Processing parameters
SI 32768
SF 100.6177967 MHz
WDW EM
SSB 0
LB 1.00 Hz
GB 0
PC 1.40

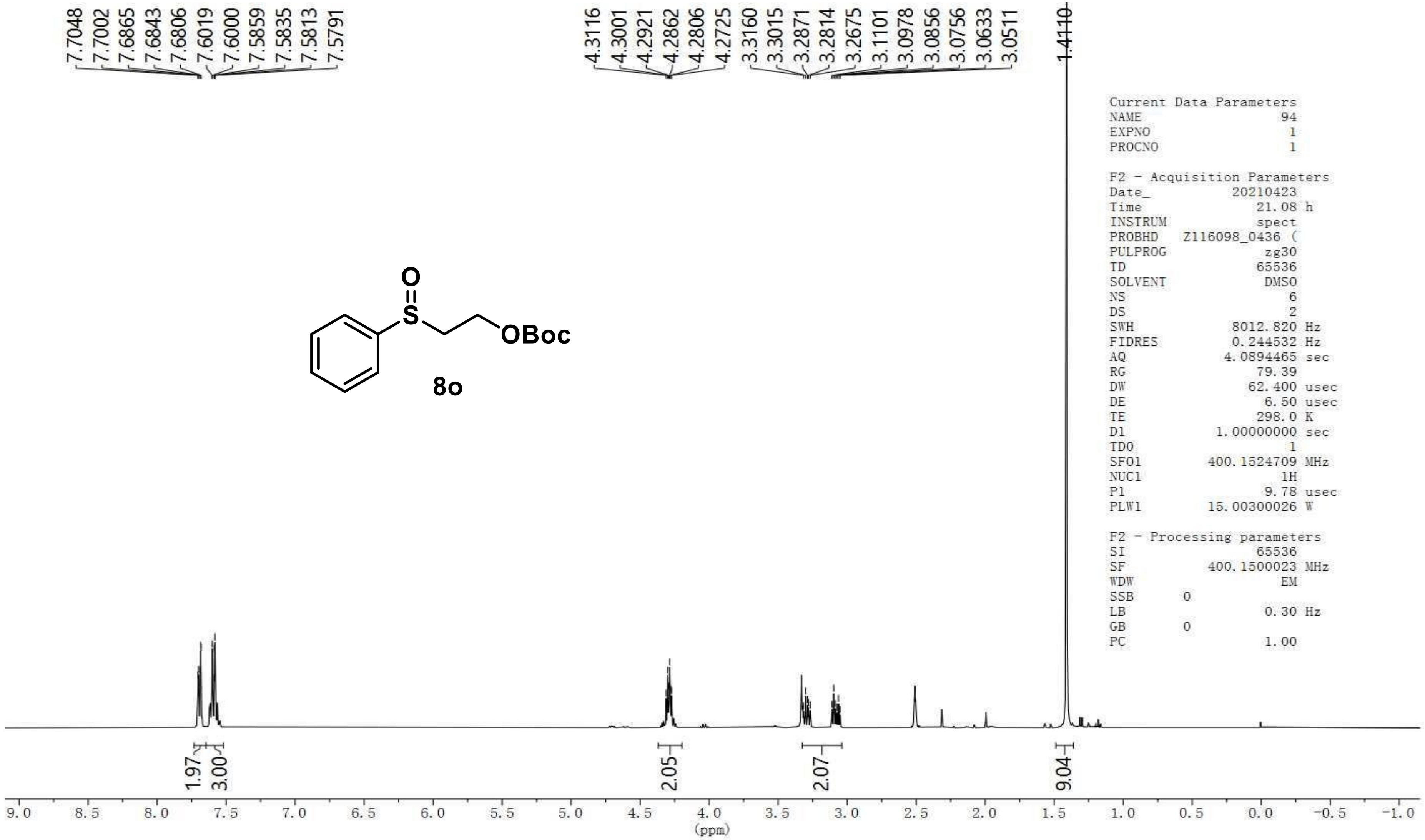
7.7048
7.7002
7.6865
7.6843
7.6806
7.6019
7.6000
7.5859
7.5835
7.5813
7.5791
4.3116
4.3001
4.2921
4.2862
4.2806
4.2725
3.3160
3.3015
3.2871
3.2814
3.2675
3.1101
3.0978
3.0856
3.0756
3.0633
3.0511
1.4110
O
S
OBoc
8o
Current Data Parameters
NAME 94
EXPNO 1
PROCNO 1
F2 - Acquisition Parameters
Date_ 20210423
Time 21.08 h
INSTRUM spect
PROBHD Z116098_0436 (
PULPROG zg30
TD 65536
SOLVENT DMSO
NS 6
DS 2
SWH 8012.820 Hz
FIDRES 0.244532 Hz
AQ 4.0894465 sec
RG 79.39
DW 62.400 usec
DE 6.50 usec
TE 298.0 K
D1 1.00000000 sec
TD0 1
SFO1 400.1524709 MHz
NUC1 1H
P1 9.78 usec
PLW1 15.00300026 W
F2 - Processing parameters
SI 65536
SF 400.1500023 MHz
WDW EM
SSB 0
LB 0.30 Hz
GB 0
PC 1.00
1.97
3.00
2.05
2.07
9.04
9.0
8.5
8.0
7.5
7.0
6.5
6.0
5.5
5.0
4.5
4.0
3.5
3.0
2.5
2.0
1.5
1.0
0.5
0.0
-0.5
-1.0
(ppm)

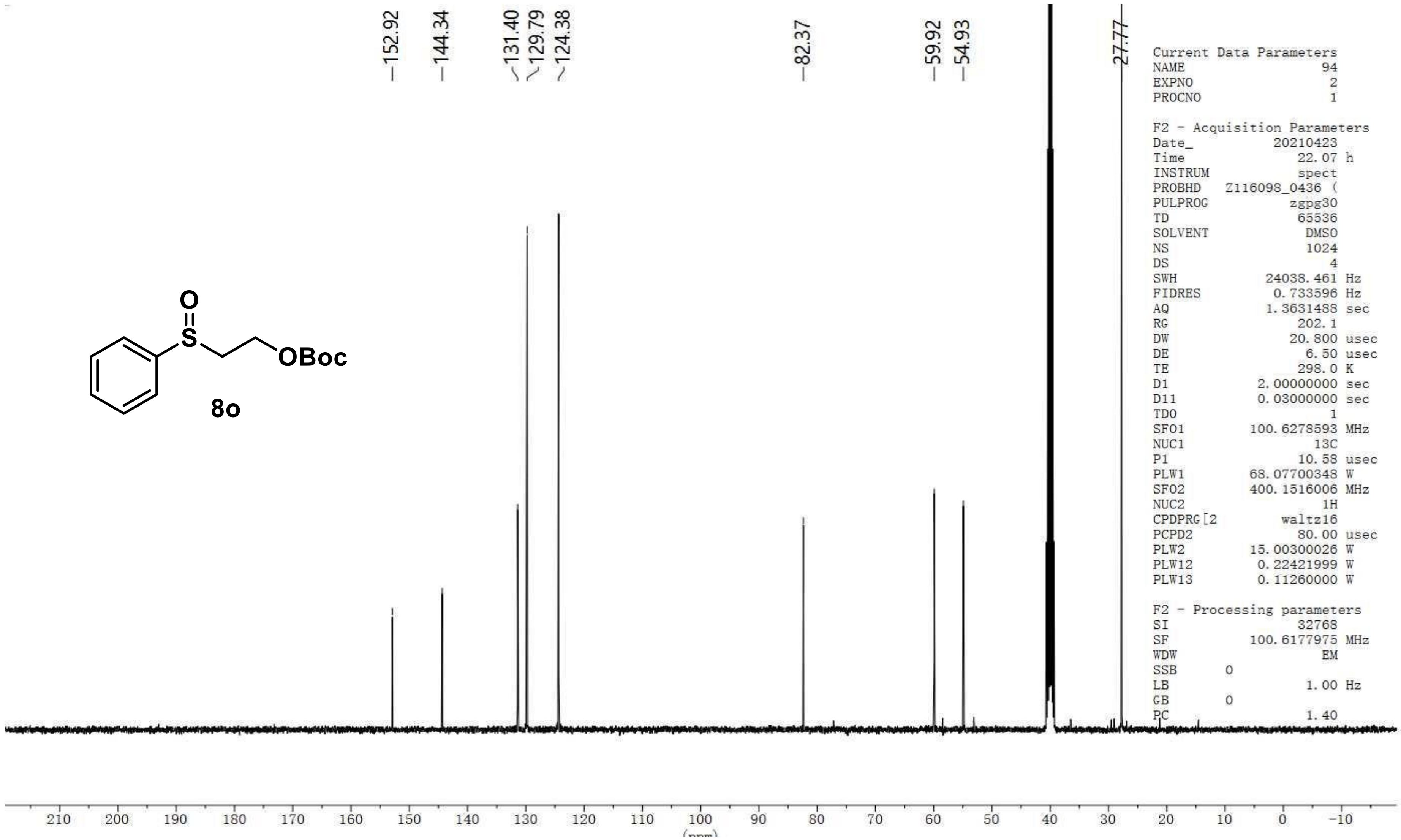

152.92
144.34
131.40
129.79
124.38
82.37
59.92
54.93
27.77
O
S
OBoc
8o
Current Data Parameters
NAME 94
EXPNO 2
PROCNO 1
F2 - Acquisition Parameters
Date_ 20210423
Time 22.07 h
INSTRUM spect
PROBHD Z116098_0436 (
PULPROG zgpg30
TD 65536
SOLVENT DMSO
NS 1024
DS 4
SWH 24038.461 Hz
FIDRES 0.733596 Hz
AQ 1.3631488 sec
RG 202.1
DW 20.800 usec
DE 6.50 usec
TE 298.0 K
D1 2.00000000 sec
D11 0.03000000 sec
TD0 1
SFO1 100.6278593 MHz
NUC1 13C
P1 10.58 usec
PLW1 68.07700348 W
SFO2 400.1516006 MHz
NUC2 1H
CPDPRG[2 waltz16
PCPD2 80.00 usec
PLW2 15.00300026 W
PLW12 0.22421999 W
PLW13 0.11260000 W
F2 - Processing parameters
SI 32768
SF 100.6177975 MHz
WDW EM
SSB 0
LB 1.00 Hz
GB 0
PC 1.40
210 200 190 180 170 160 150 140 130 120 110 100 90 80 70 60 50 40 30 20 10 0 -10

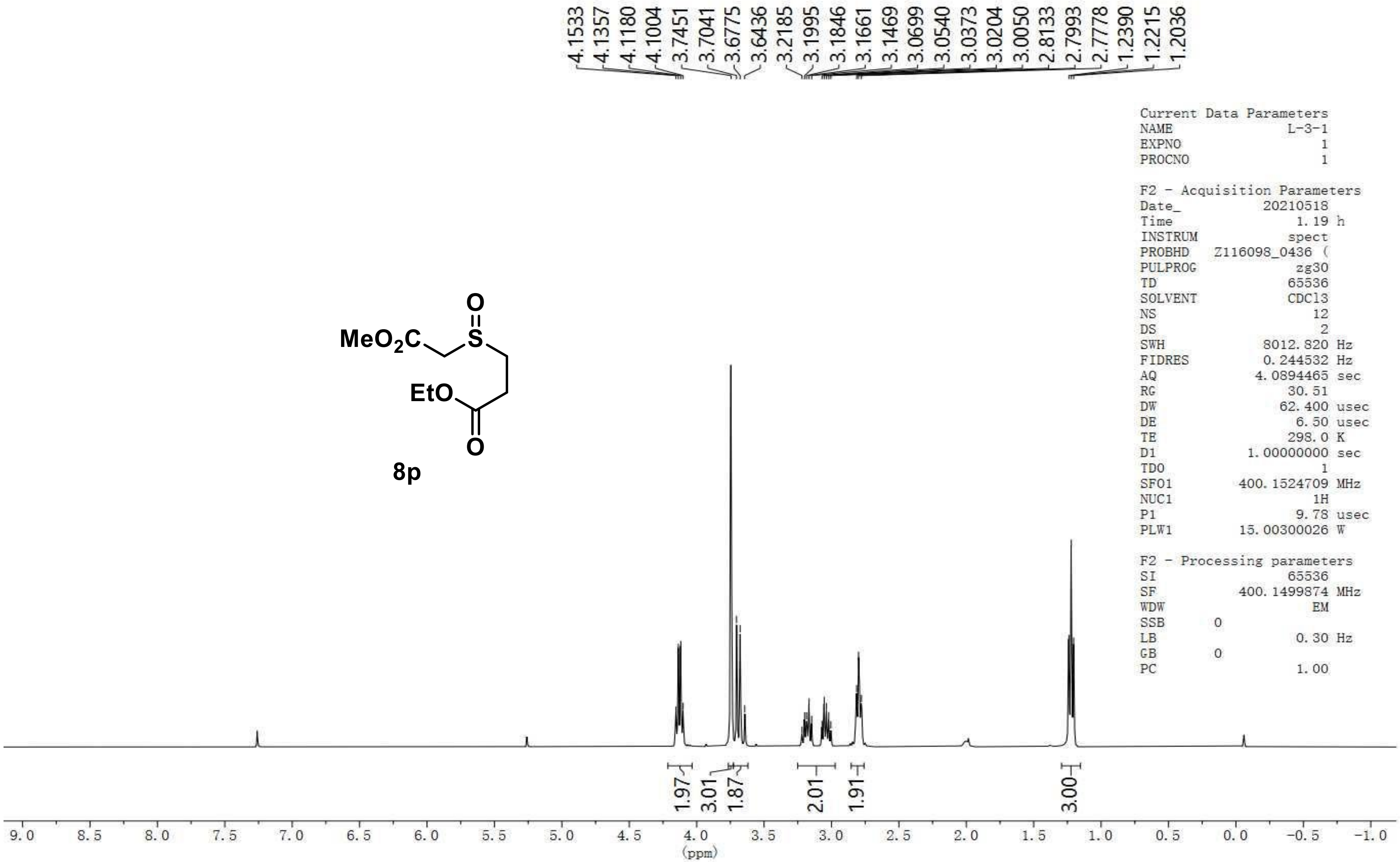

8p

```
Current Data Parameters
NAME              L-3-1
EXPNO                 1
PROCNO                1

F2 - Acquisition Parameters
Date_          20210518
Time              1.19 h
INSTRUM           spect
PROBHD   Z116098_0436 (
PULPROG            zg30
TD                65536
SOLVENT           CDCl3
NS                   12
DS                    2
SWH           8012.820 Hz
FIDRES        0.244532 Hz
AQ           4.0894465 sec
RG                30.51
DW               62.400 usec
DE                 6.50 usec
TE                298.0 K
D1          1.00000000 sec
TD0                   1
SFO1       400.1524709 MHz
NUC1                 1H
P1                 9.78 usec
PLW1        15.00300026 W

F2 - Processing parameters
SI                65536
SF         400.1499874 MHz
WDW                  EM
SSB      0
LB                 0.30 Hz
GB       0
PC                 1.00
```

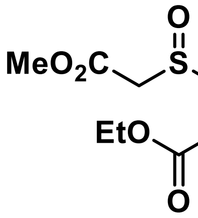
MeO2C
S
O
EtO
O

8p

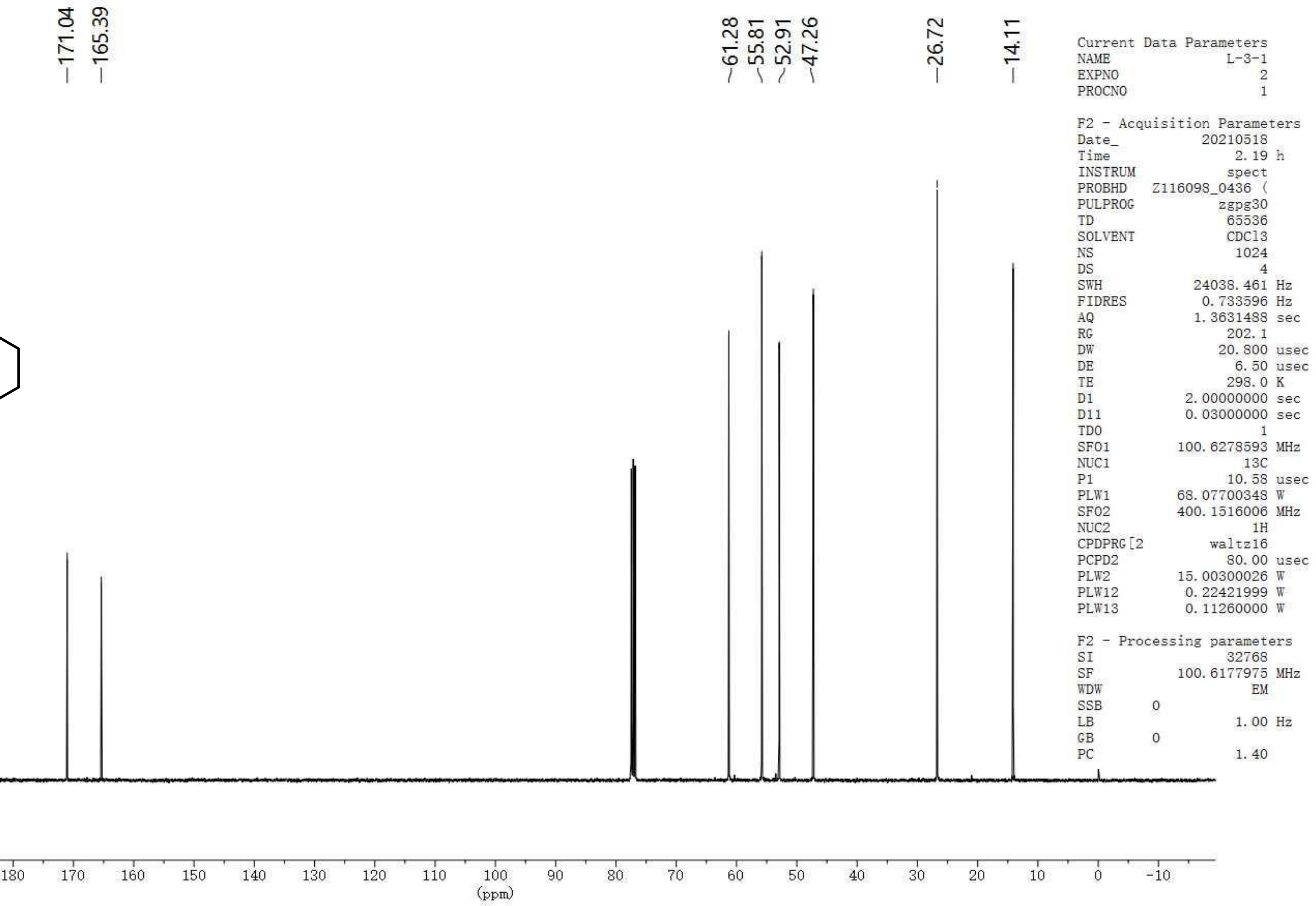
171.04
165.39
61.28
55.81
52.91
47.26
26.72
14.11
Current Data Parameters
NAME L-3-1
EXPNO 2
PROCNO 1
F2 - Acquisition Parameters
Date_ 20210518
Time 2.19 h
INSTRUM spect
PROBHD Z116098_0436 (
PULPROG zgpg30
TD 65536
SOLVENT CDCl3
NS 1024
DS 4
SWH 24038.461 Hz
FIDRES 0.733596 Hz
AQ 1.3631488 sec
RG 202.1
DW 20.800 usec
DE 6.50 usec
TE 298.0 K
D1 2.00000000 sec
D11 0.03000000 sec
TD0 1
SFO1 100.6278593 MHz
NUC1 13C
P1 10.58 usec
PLW1 68.07700348 W
SFO2 400.1516006 MHz
NUC2 1H
CPDPRG[2 waltz16
PCPD2 80.00 usec
PLW2 15.00300026 W
PLW12 0.22421999 W
PLW13 0.11260000 W
F2 - Processing parameters
SI 32768
SF 100.6177975 MHz
WDW EM
SSB 0
LB 1.00 Hz
GB 0
PC 1.40
210 200 190 180 170 160 150 140 130 120 110 100 90 80 70 60 50 40 30 20 10 0 -10
(ppm)

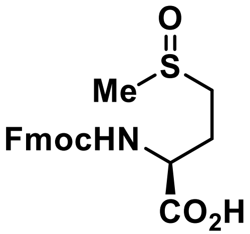

8q

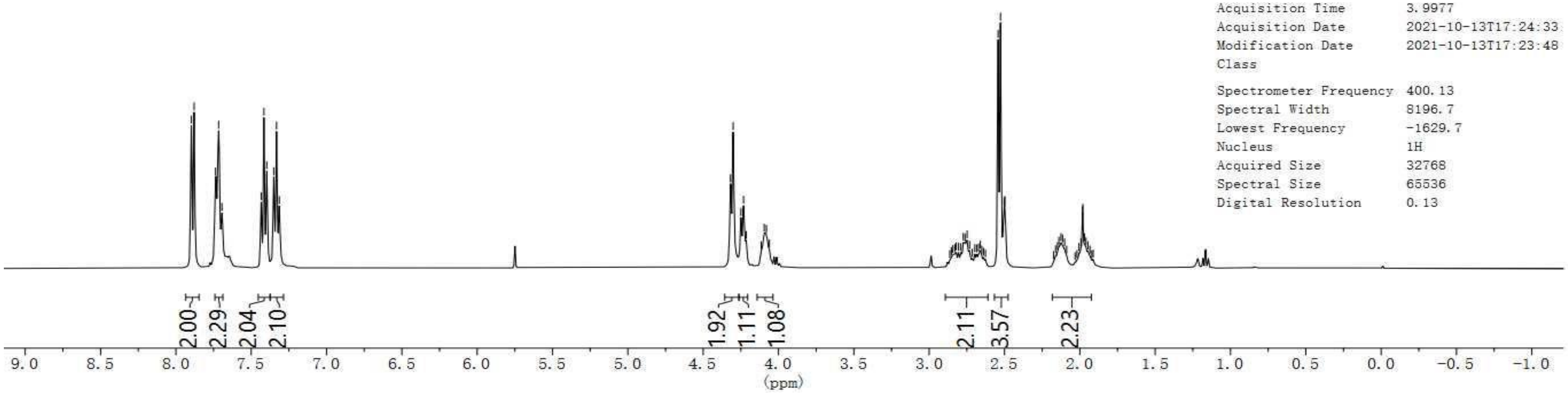

Parameter	值
Comment	L-3-113-H
Origin	Bruker BioSpin GmbH
Owner	nmrsu
Site	
Instrument	Avance NEO
Author	
Solvent	DMSO
Temperature	297.0
Pulse Sequence	zg30
Experiment	1D
Number of Scans	16
Receiver Gain	59.4
Relaxation Delay	1.0000
Pulse Width	8.0000
Presaturation Frequency	
Acquisition Time	3.9977
Acquisition Date	2021-10-13T17:24:33
Modification Date	2021-10-13T17:23:48
Class	
Spectrometer Frequency	400.13
Spectral Width	8196.7
Lowest Frequency	-1629.7
Nucleus	1H
Acquired Size	32768
Spectral Size	65536
Digital Resolution	0.13

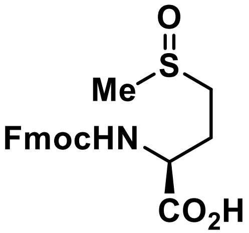

8q

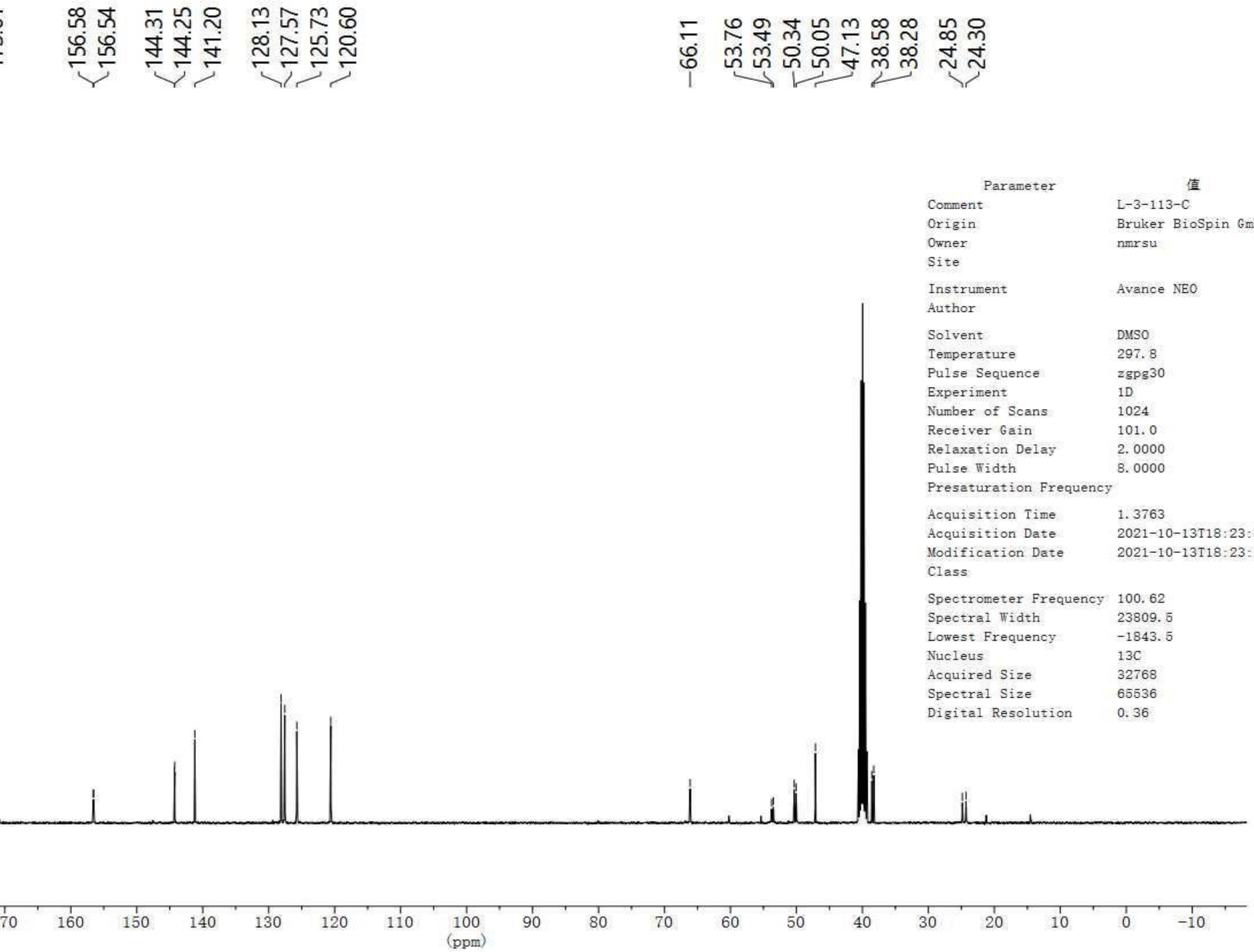

Parameter	值
Comment	L-3-113-C
Origin	Bruker BioSpin GmbH
Owner	nmrsu
Site	
Instrument	Avance NEO
Author	
Solvent	DMSO
Temperature	297.8
Pulse Sequence	zgpg30
Experiment	1D
Number of Scans	1024
Receiver Gain	101.0
Relaxation Delay	2.0000
Pulse Width	8.0000
Presaturation Frequency	
Acquisition Time	1.3763
Acquisition Date	2021-10-13T18:23:58
Modification Date	2021-10-13T18:23:14
Class	
Spectrometer Frequency	100.62
Spectral Width	23809.5
Lowest Frequency	-1843.5
Nucleus	13C
Acquired Size	32768
Spectral Size	65536
Digital Resolution	0.36

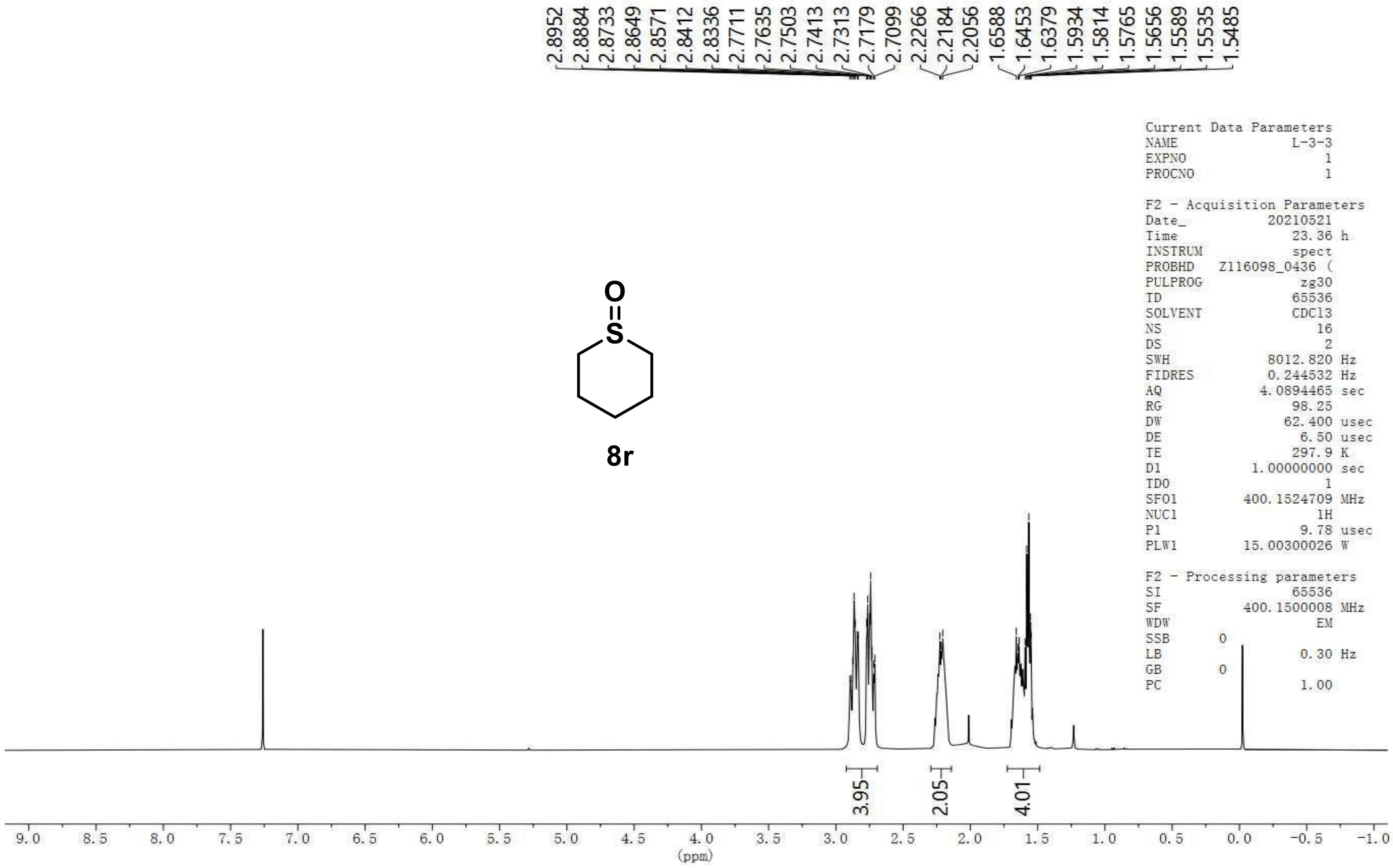

2.8952
2.8884
2.8733
2.8649
2.8571
2.8412
2.8336
2.7711
2.7635
2.7503
2.7413
2.7313
2.7179
2.7099
2.2266
2.2184
2.2056
1.6588
1.6453
1.6379
1.5934
1.5814
1.5765
1.5656
1.5589
1.5535
1.5485
Current Data Parameters
NAME L-3-3
EXPNO 1
PROCNO 1
F2 - Acquisition Parameters
Date_ 20210521
Time 23.36 h
INSTRUM spect
PROBHD Z116098_0436 (
PULPROG zg30
TD 65536
SOLVENT CDCl3
NS 16
DS 2
SWH 8012.820 Hz
FIDRES 0.244532 Hz
AQ 4.0894465 sec
RG 98.25
DW 62.400 usec
DE 6.50 usec
TE 297.9 K
D1 1.00000000 sec
TD0 1
SFO1 400.1524709 MHz
NUC1 1H
P1 9.78 usec
PLW1 15.00300026 W
F2 - Processing parameters
SI 65536
SF 400.1500008 MHz
WDW EM
SSB 0
LB 0.30 Hz
GB 0
PC 1.00
3.95
2.05
4.01
9.0 8.5 8.0 7.5 7.0 6.5 6.0 5.5 5.0 4.5 4.0 3.5 3.0 2.5 2.0 1.5 1.0 0.5 0.0 -0.5 -1.0
(ppm)

8r

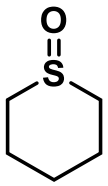

8r

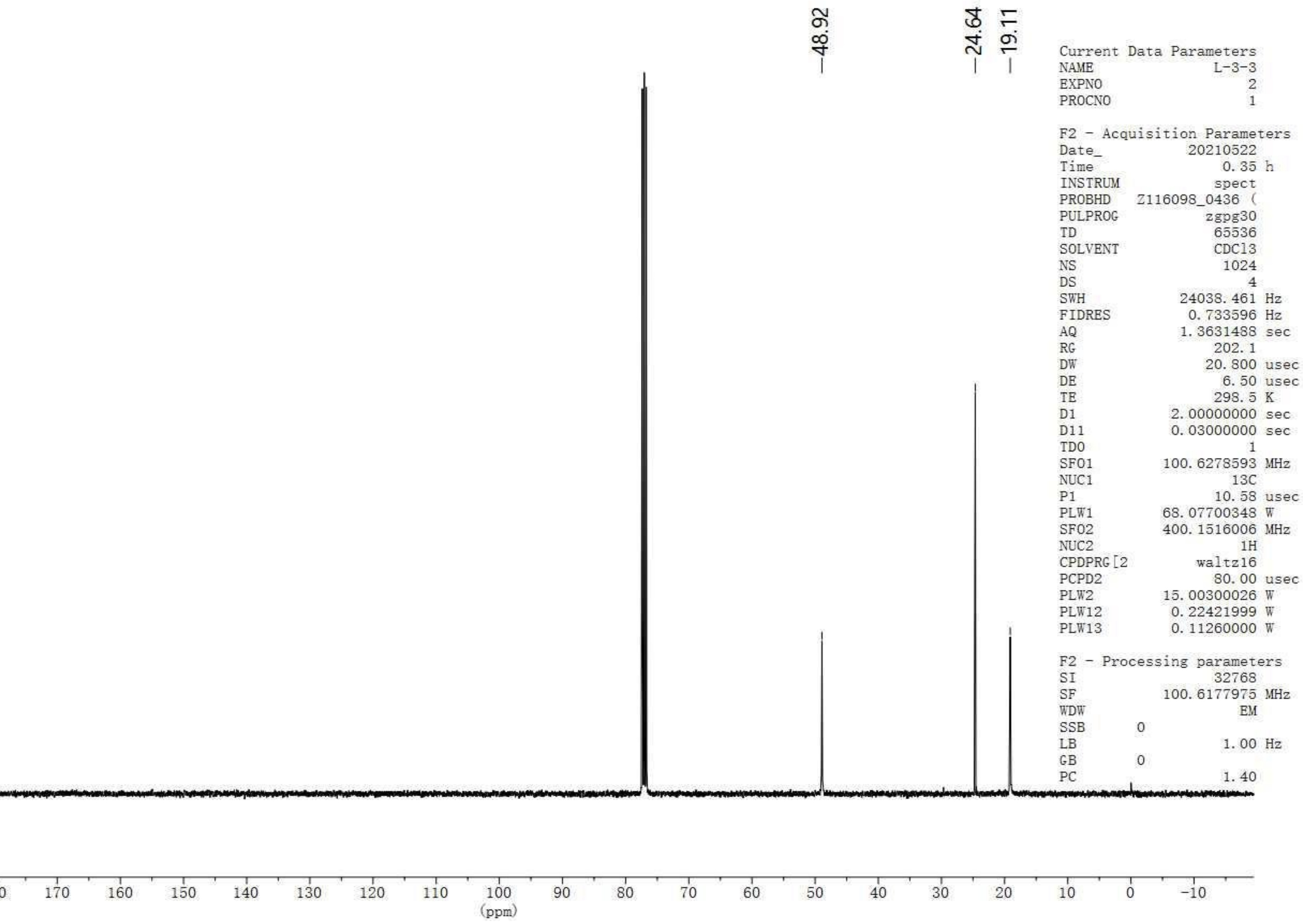

48.92
24.64
19.11
Current Data Parameters
NAME L-3-3
EXPNO 2
PROCNO 1
F2 - Acquisition Parameters
Date_ 20210522
Time 0.35 h
INSTRUM spect
PROBHD Z116098_0436 (
PULPROG zgpg30
TD 65536
SOLVENT CDCl3
NS 1024
DS 4
SWH 24038.461 Hz
FIDRES 0.733596 Hz
AQ 1.3631488 sec
RG 202.1
DW 20.800 usec
DE 6.50 usec
TE 298.5 K
D1 2.00000000 sec
D11 0.03000000 sec
TD0 1
SFO1 100.6278593 MHz
NUC1 13C
P1 10.58 usec
PLW1 68.07700348 W
SFO2 400.1516006 MHz
NUC2 1H
CPDPRG[2 waltz16
PCPD2 80.00 usec
PLW2 15.00300026 W
PLW12 0.22421999 W
PLW13 0.11260000 W
F2 - Processing parameters
SI 32768
SF 100.6177975 MHz
WDW EM
SSB 0
LB 1.00 Hz
GB 0
PC 1.40
210 200 190 180 170 160 150 140 130 120 110 100 90 80 70 60 50 40 30 20 10 0 -10
(ppm)

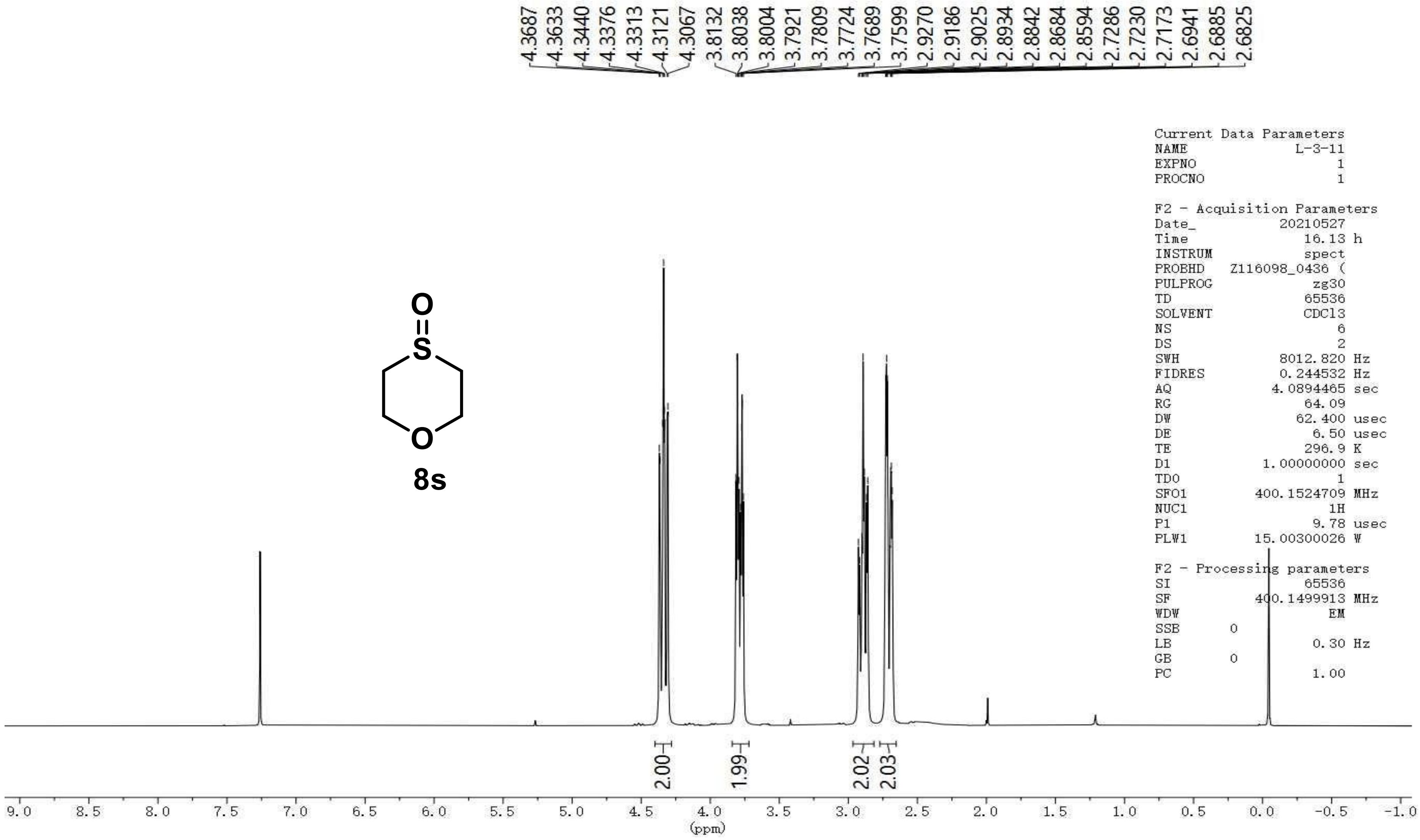

8s
4.3687
4.3633
4.3440
4.3376
4.3313
4.3121
4.3067
3.8132
3.8038
3.8004
3.7921
3.7809
3.7724
3.7689
3.7599
2.9270
2.9186
2.9025
2.8934
2.8842
2.8684
2.8594
2.7286
2.7230
2.7173
2.6941
2.6885
2.6825
Current Data Parameters
NAME L-3-11
EXPNO 1
PROCNO 1
F2 - Acquisition Parameters
Date_ 20210527
Time 16.13 h
INSTRUM spect
PROBHD Z116098_0436 (
PULPROG zg30
TD 65536
SOLVENT CDCl3
NS 6
DS 2
SWH 8012.820 Hz
FIDRES 0.244532 Hz
AQ 4.0894465 sec
RG 64.09
DW 62.400 usec
DE 6.50 usec
TE 296.9 K
D1 1.00000000 sec
TD0 1
SFO1 400.1524709 MHz
NUC1 1H
P1 9.78 usec
PLW1 15.00300026 W
F2 - Processing parameters
SI 65536
SF 400.1499913 MHz
WDW EM
SSB 0
LB 0.30 Hz
GB 0
PC 1.00
2.00
1.99
2.02
2.03
9.0 8.5 8.0 7.5 7.0 6.5 6.0 5.5 5.0 4.5 4.0 3.5 3.0 2.5 2.0 1.5 1.0 0.5 0.0 -0.5 -1.0
(ppm)

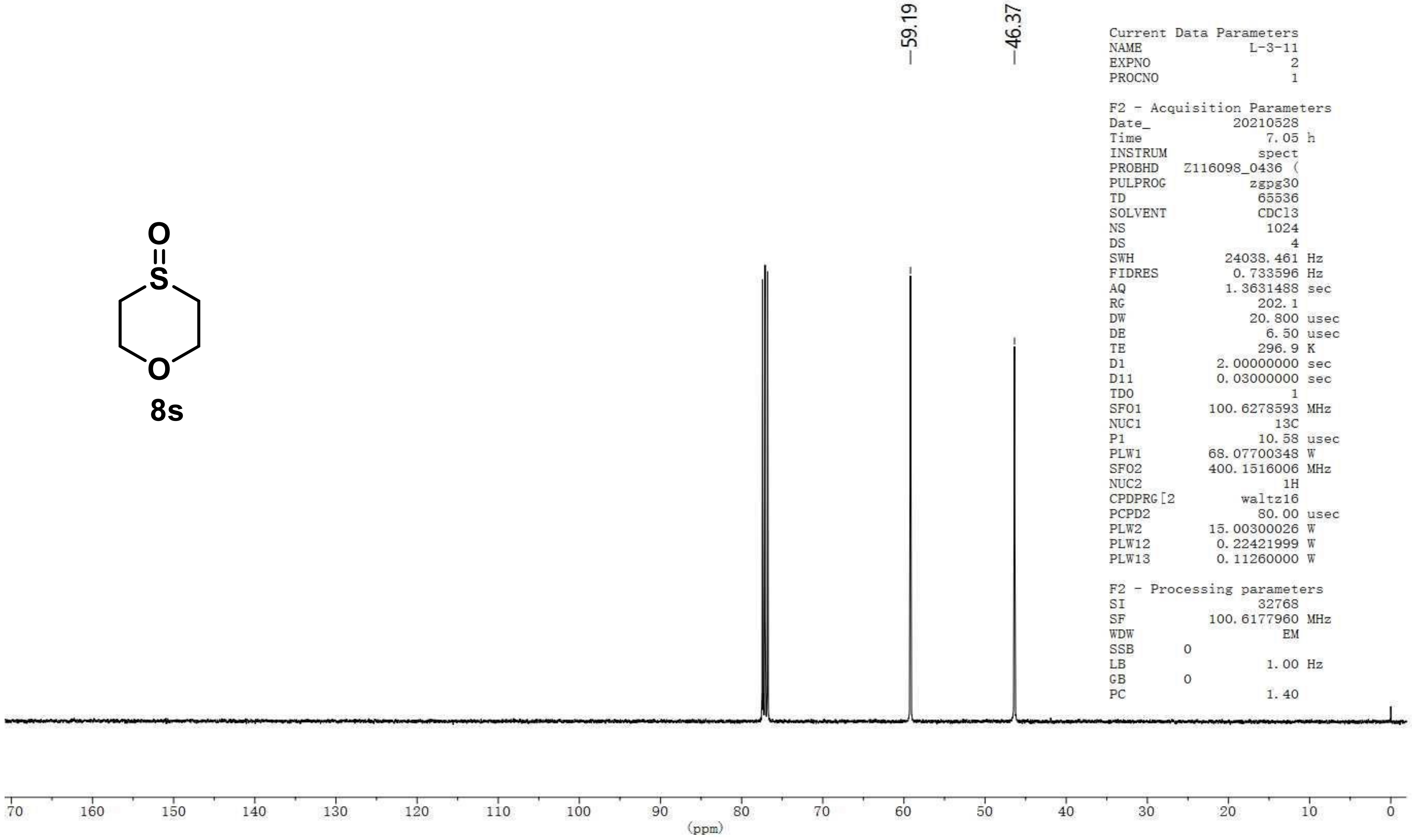
O
S
O
8s
59.19
46.37
Current Data Parameters
NAME L-3-11
EXPNO 2
PROCNO 1
F2 - Acquisition Parameters
Date_ 20210528
Time 7.05 h
INSTRUM spect
PROBHD Z116098_0436 (
PULPROG zgpg30
TD 65536
SOLVENT CDCl3
NS 1024
DS 4
SWH 24038.461 Hz
FIDRES 0.733596 Hz
AQ 1.3631488 sec
RG 202.1
DW 20.800 usec
DE 6.50 usec
TE 296.9 K
D1 2.00000000 sec
D11 0.03000000 sec
TD0 1
SFO1 100.6278593 MHz
NUC1 13C
P1 10.58 usec
PLW1 68.07700348 W
SFO2 400.1516006 MHz
NUC2 1H
CPDPRG[2 waltz16
PCPD2 80.00 usec
PLW2 15.00300026 W
PLW12 0.22421999 W
PLW13 0.11260000 W
F2 - Processing parameters
SI 32768
SF 100.6177960 MHz
WDW EM
SSB 0
LB 1.00 Hz
GB 0
PC 1.40
70 160 150 140 130 120 110 100 90 80 70 60 50 40 30 20 10 0
(ppm)

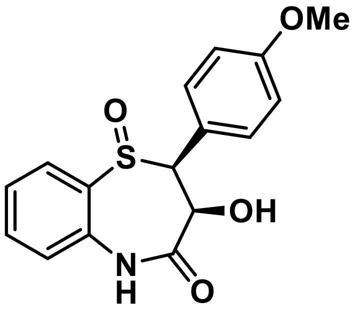

8t

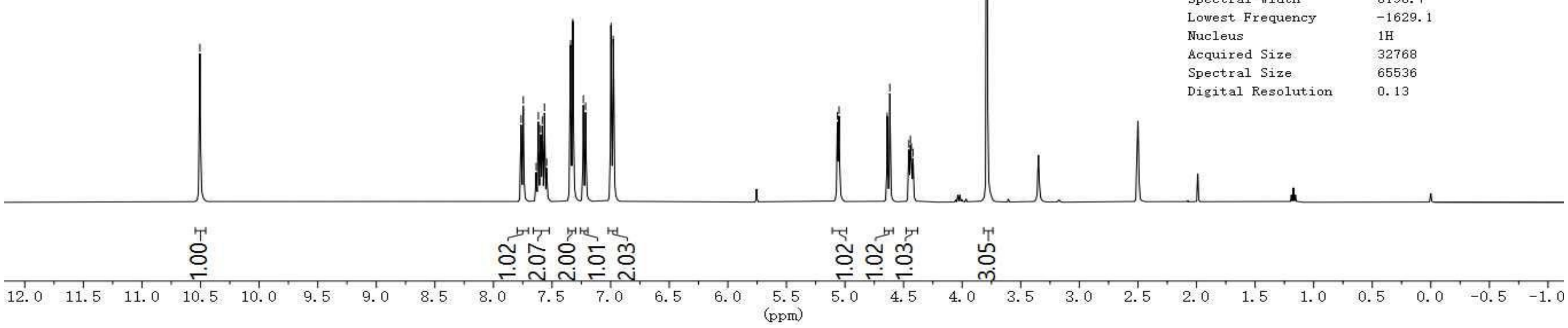

Parameter	值
Comment	L-3-111-H
Origin	Bruker BioSpin GmbH
Owner	nmrsu
Site	
Instrument	Avance NEO
Author	
Solvent	DMSO
Temperature	296.9
Pulse Sequence	zg30
Experiment	1D
Number of Scans	16
Receiver Gain	101.0
Relaxation Delay	1.0000
Pulse Width	8.0000
Presaturation Frequency	
Acquisition Time	3.9977
Acquisition Date	2021-10-13T16:09:58
Modification Date	2021-10-13T16:09:12
Class	
Spectrometer Frequency	400.13
Spectral Width	8196.7
Lowest Frequency	-1629.1
Nucleus	1H
Acquired Size	32768
Spectral Size	65536
Digital Resolution	0.13

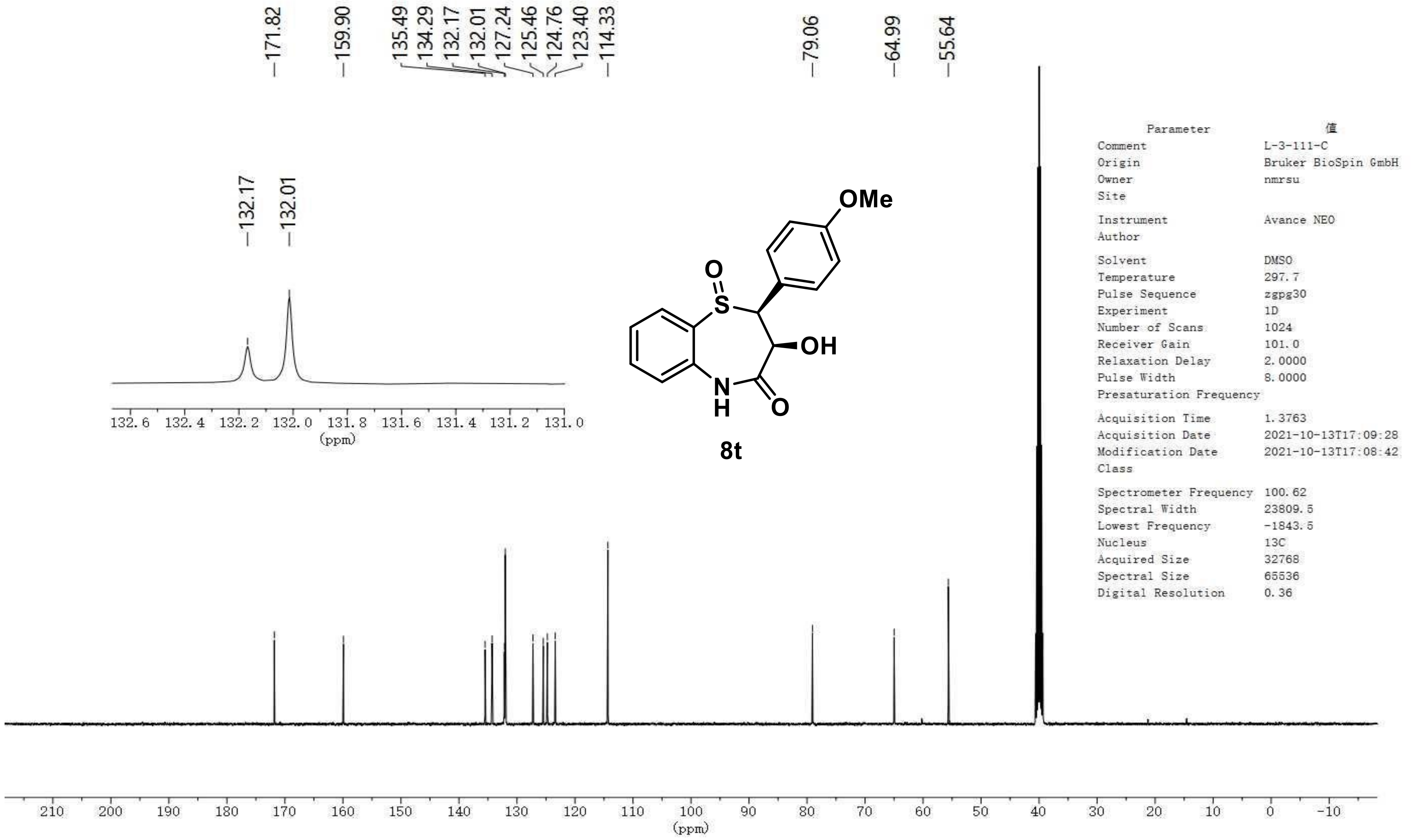

171.82
159.90
135.49
134.29
132.17
132.01
127.24
125.46
124.76
123.40
114.33
79.06
64.99
55.64
132.17
132.01
132.6 132.4 132.2 132.0 131.8 131.6 131.4 131.2 131.0
(ppm)
OMe
O
S
OH
N
H
O
8t
Parameter 值
Comment L-3-111-C
Origin Bruker BioSpin GmbH
Owner nmrsu
Site
Instrument Avance NEO
Author
Solvent DMSO
Temperature 297.7
Pulse Sequence zgpg30
Experiment 1D
Number of Scans 1024
Receiver Gain 101.0
Relaxation Delay 2.0000
Pulse Width 8.0000
Presaturation Frequency
Acquisition Time 1.3763
Acquisition Date 2021-10-13T17:09:28
Modification Date 2021-10-13T17:08:42
Class
Spectrometer Frequency 100.62
Spectral Width 23809.5
Lowest Frequency -1843.5
Nucleus 13C
Acquired Size 32768
Spectral Size 65536
Digital Resolution 0.36
210 200 190 180 170 160 150 140 130 120 110 100 90 80 70 60 50 40 30 20 10 0 -10
(ppm)

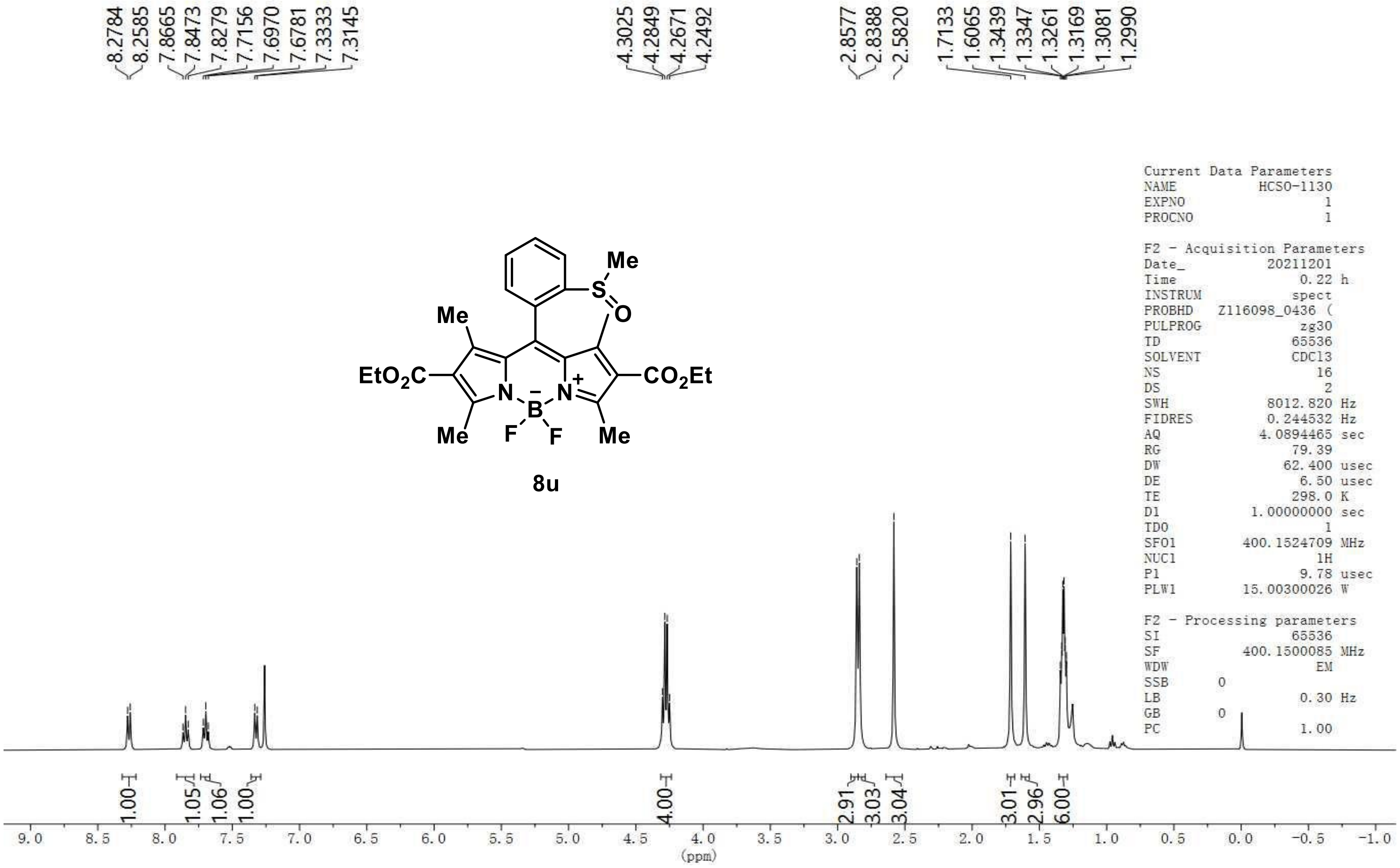
8.2784
8.2585
7.8665
7.8473
7.8279
7.7156
7.6970
7.6781
7.3333
7.3145
4.3025
4.2849
4.2671
4.2492
2.8577
2.8388
2.5820
1.7133
1.6065
1.3439
1.3347
1.3261
1.3169
1.3081
1.2990
Current Data Parameters
NAME HCSO-1130
EXPNO 1
PROCNO 1
F2 - Acquisition Parameters
Date_ 20211201
Time 0.22 h
INSTRUM spect
PROBHD Z116098_0436 (
PULPROG zg30
TD 65536
SOLVENT CDCl3
NS 16
DS 2
SWH 8012.820 Hz
FIDRES 0.244532 Hz
AQ 4.0894465 sec
RG 79.39
DW 62.400 usec
DE 6.50 usec
TE 298.0 K
D1 1.00000000 sec
TD0 1
SFO1 400.1524709 MHz
NUC1 1H
P1 9.78 usec
PLW1 15.00300026 W
F2 - Processing parameters
SI 65536
SF 400.1500085 MHz
WDW EM
SSB 0
LB 0.30 Hz
GB 0
PC 1.00
Me
S
O
Me
EtO2C
CO2Et
N
B
N
F
F
Me
Me
8u
1.00
1.05
1.06
1.00
4.00
2.91
3.03
3.04
3.01
2.96
6.00
9.0
8.5
8.0
7.5
7.0
6.5
6.0
5.5
5.0
4.5
4.0
(ppm)
3.5
3.0
2.5
2.0
1.5
1.0
0.5
0.0
-0.5
-1.0

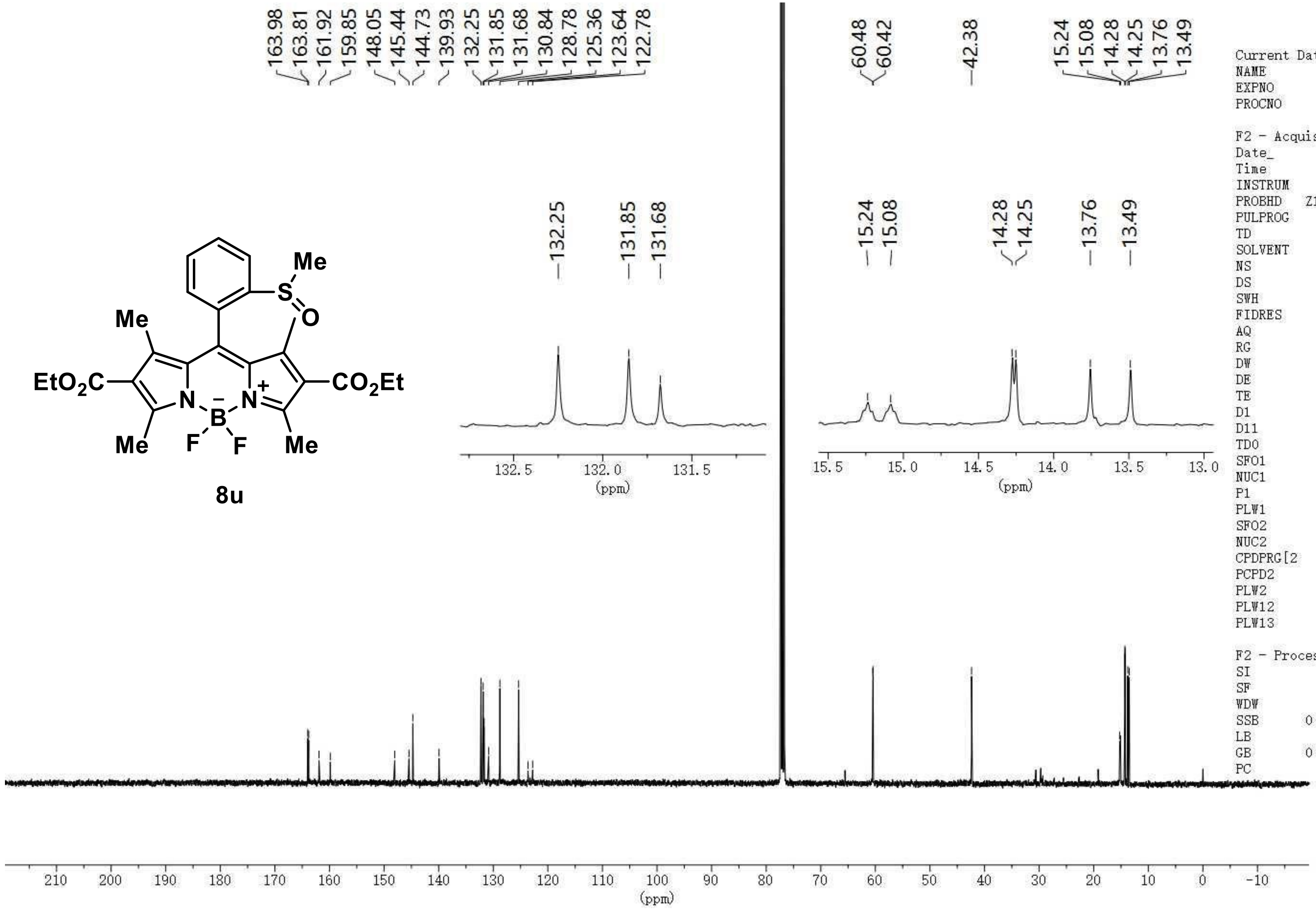

8u

Current Data Parameters
NAME HCSO-1130
EXPNO 2
PROCNO 1

F2 - Acquisition Parameters
Date_ 20211201
Time 2.20 h
INSTRUM spect
PROBHD Z116098_0436 (
PULPROG zgpg30
TD 65536
SOLVENT CDCl3
NS 2048
DS 4
SWH 24038.461 Hz
FIDRES 0.733596 Hz
AQ 1.3631488 sec
RG 202.1
DW 20.800 usec
DE 6.50 usec
TE 298.0 K
D1 2.00000000 sec
D11 0.03000000 sec
TD0 1
SFO1 100.6278593 MHz
NUC1 13C
P1 10.58 usec
PLW1 68.07700348 W
SFO2 400.1516006 MHz
NUC2 1H
CPDPRG[2 waltz16
PCPD2 80.00 usec
PLW2 15.00300026 W
PLW12 0.22421999 W
PLW13 0.11260000 W

F2 - Processing parameters
SI 32768
SF 100.6177975 MHz
WDW EM
SSB 0
LB 1.00 Hz
GB 0
PC 1.40

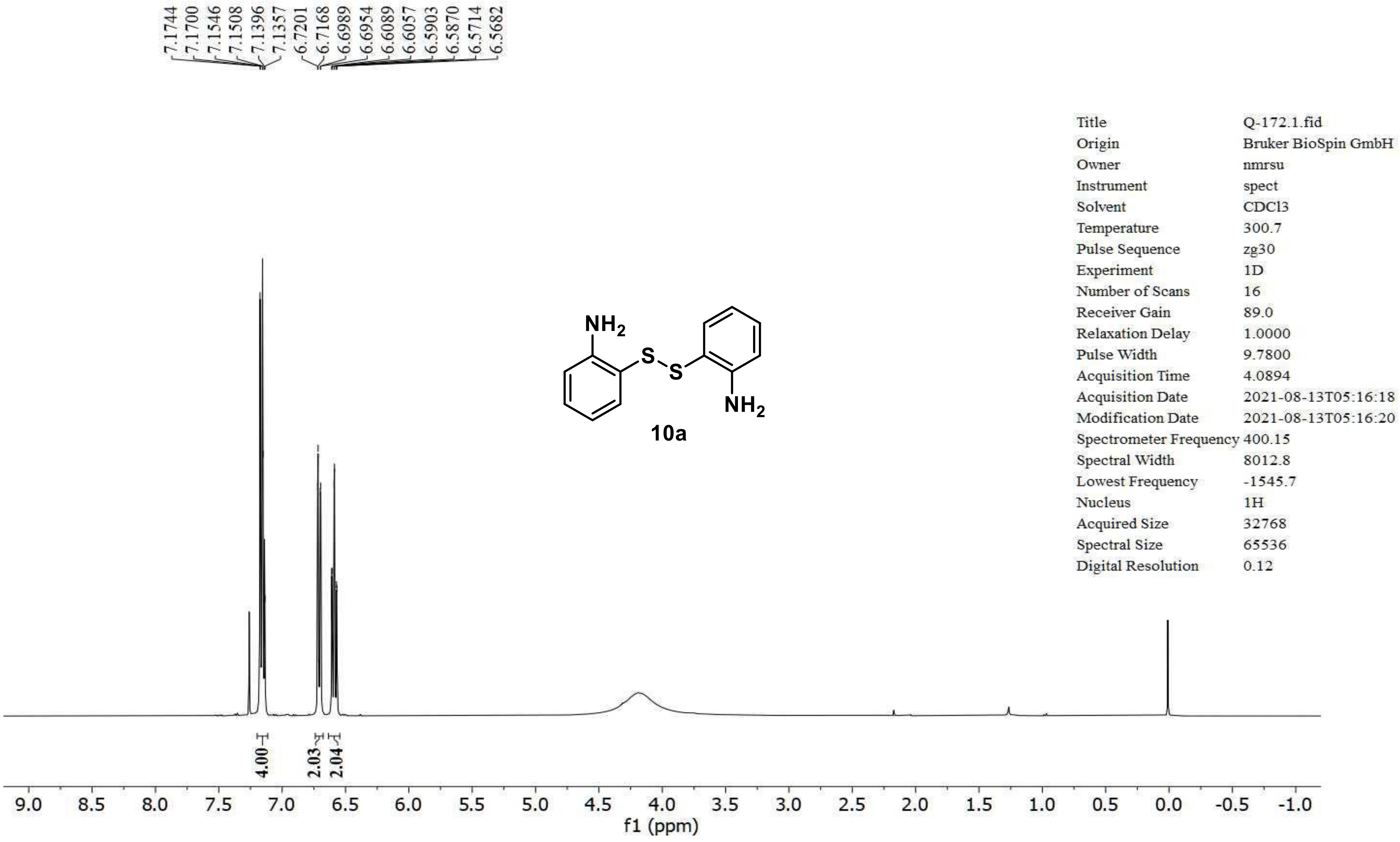

7.1744
7.1700
7.1546
7.1508
7.1396
7.1357
6.7201
6.7168
6.6989
6.6954
6.6089
6.6057
6.5903
6.5870
6.5714
6.5682
NH2
S
S
NH2
10a
Title Q-172.1.fid
Origin Bruker BioSpin GmbH
Owner nmrsu
Instrument spect
Solvent CDCl3
Temperature 300.7
Pulse Sequence zg30
Experiment 1D
Number of Scans 16
Receiver Gain 89.0
Relaxation Delay 1.0000
Pulse Width 9.7800
Acquisition Time 4.0894
Acquisition Date 2021-08-13T05:16:18
Modification Date 2021-08-13T05:16:20
Spectrometer Frequency 400.15
Spectral Width 8012.8
Lowest Frequency -1545.7
Nucleus 1H
Acquired Size 32768
Spectral Size 65536
Digital Resolution 0.12
4.00
2.03
2.04
9.0 8.5 8.0 7.5 7.0 6.5 6.0 5.5 5.0 4.5 4.0 3.5 3.0 2.5 2.0 1.5 1.0 0.5 0.0 -0.5 -1.0
f1 (ppm)

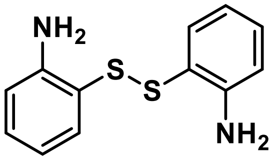

10a

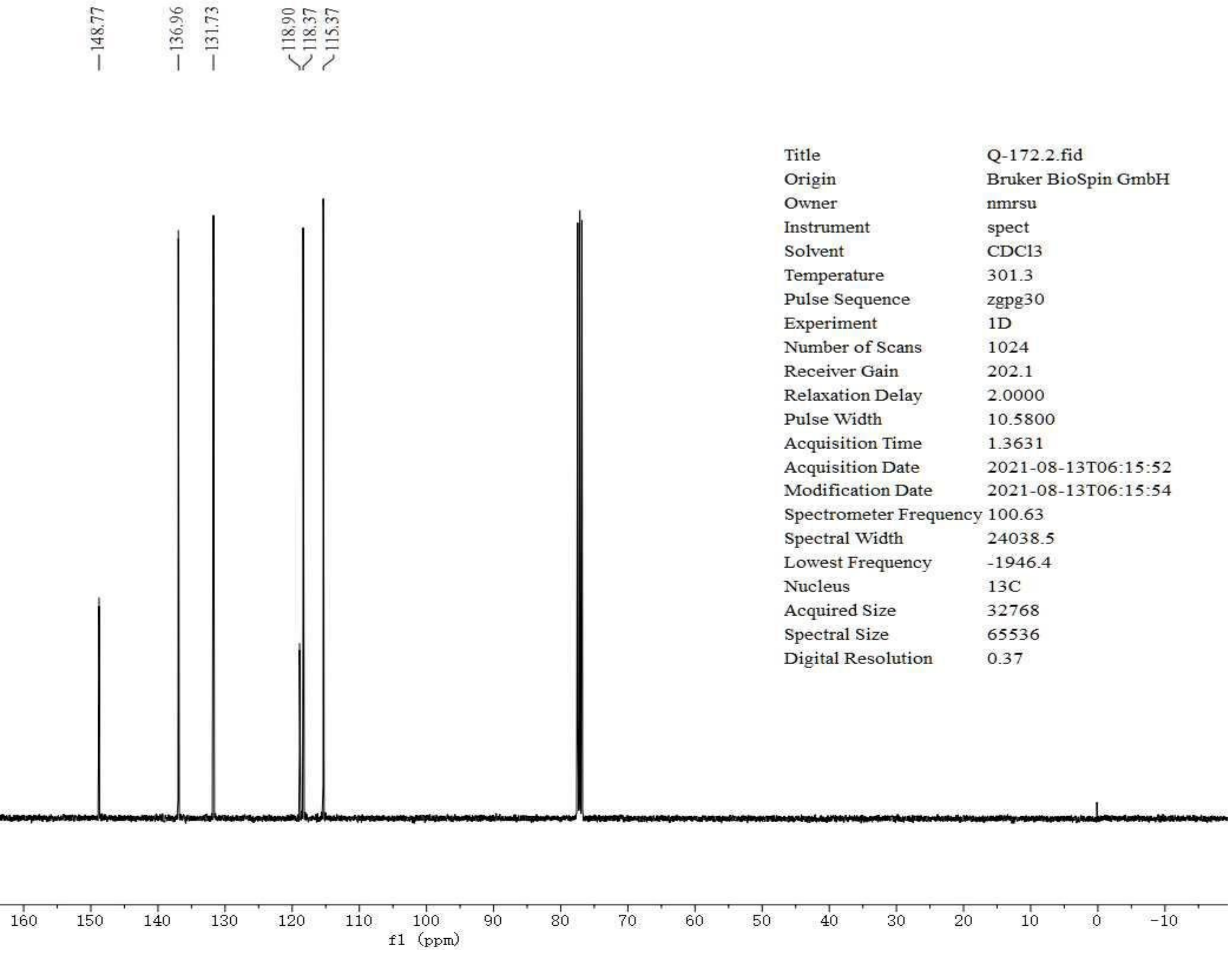
148.77
136.96
131.73
118.90
118.37
115.37
Title Q-172.2.fid
Origin Bruker BioSpin GmbH
Owner nmrsu
Instrument spect
Solvent CDCl3
Temperature 301.3
Pulse Sequence zgpg30
Experiment 1D
Number of Scans 1024
Receiver Gain 202.1
Relaxation Delay 2.0000
Pulse Width 10.5800
Acquisition Time 1.3631
Acquisition Date 2021-08-13T06:15:52
Modification Date 2021-08-13T06:15:54
Spectrometer Frequency 100.63
Spectral Width 24038.5
Lowest Frequency -1946.4
Nucleus 13C
Acquired Size 32768
Spectral Size 65536
Digital Resolution 0.37
210 200 190 180 170 160 150 140 130 120 110 100 90 80 70 60 50 40 30 20 10 0 -10
f1 (ppm)

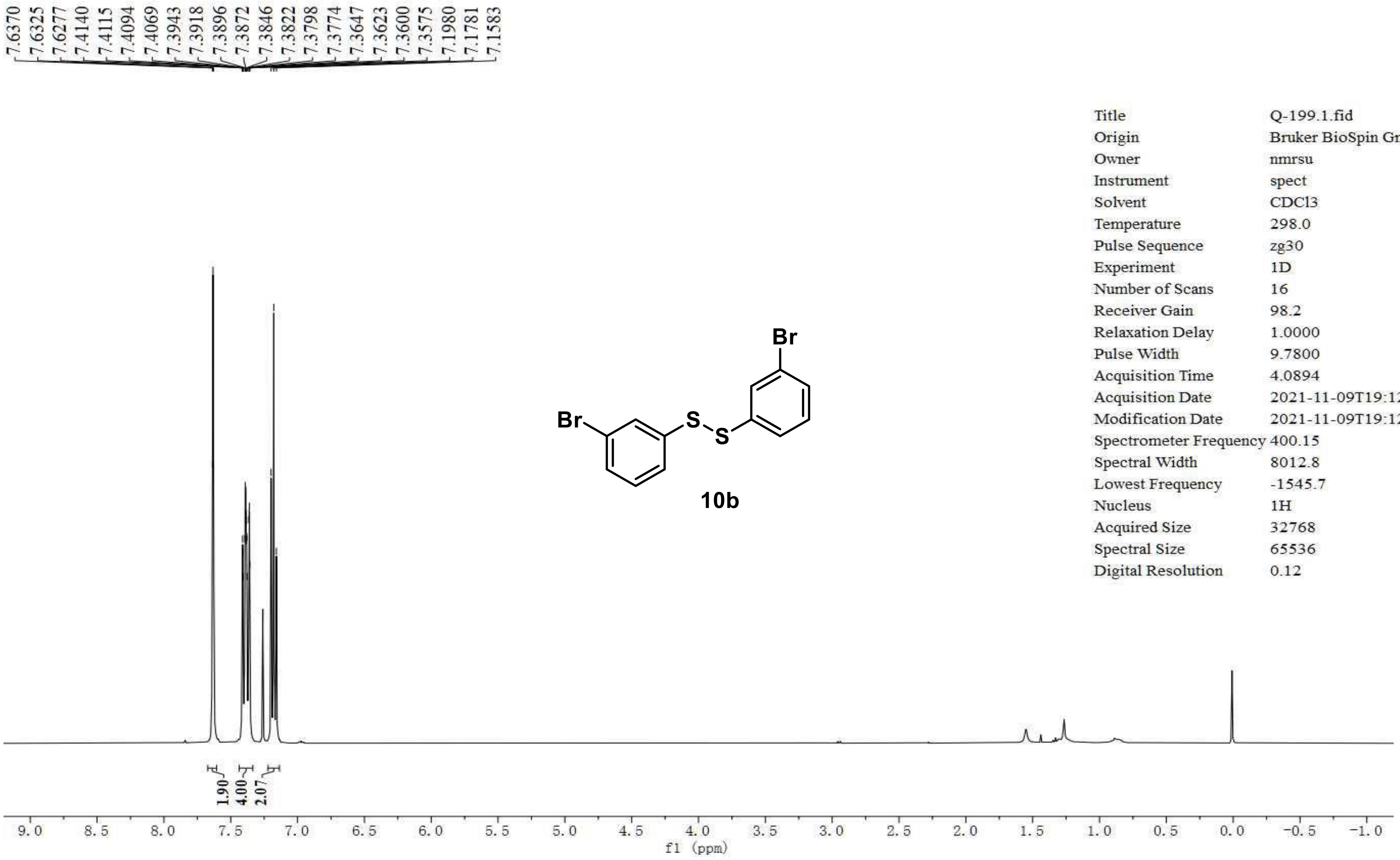

10b

Title	Q-199.1.fid
Origin	Bruker BioSpin GmbH
Owner	nmrsu
Instrument	spect
Solvent	CDCl3
Temperature	298.0
Pulse Sequence	zg30
Experiment	1D
Number of Scans	16
Receiver Gain	98.2
Relaxation Delay	1.0000
Pulse Width	9.7800
Acquisition Time	4.0894
Acquisition Date	2021-11-09T19:12:19
Modification Date	2021-11-09T19:12:18
Spectrometer Frequency	400.15
Spectral Width	8012.8
Lowest Frequency	-1545.7
Nucleus	1H
Acquired Size	32768
Spectral Size	65536
Digital Resolution	0.12

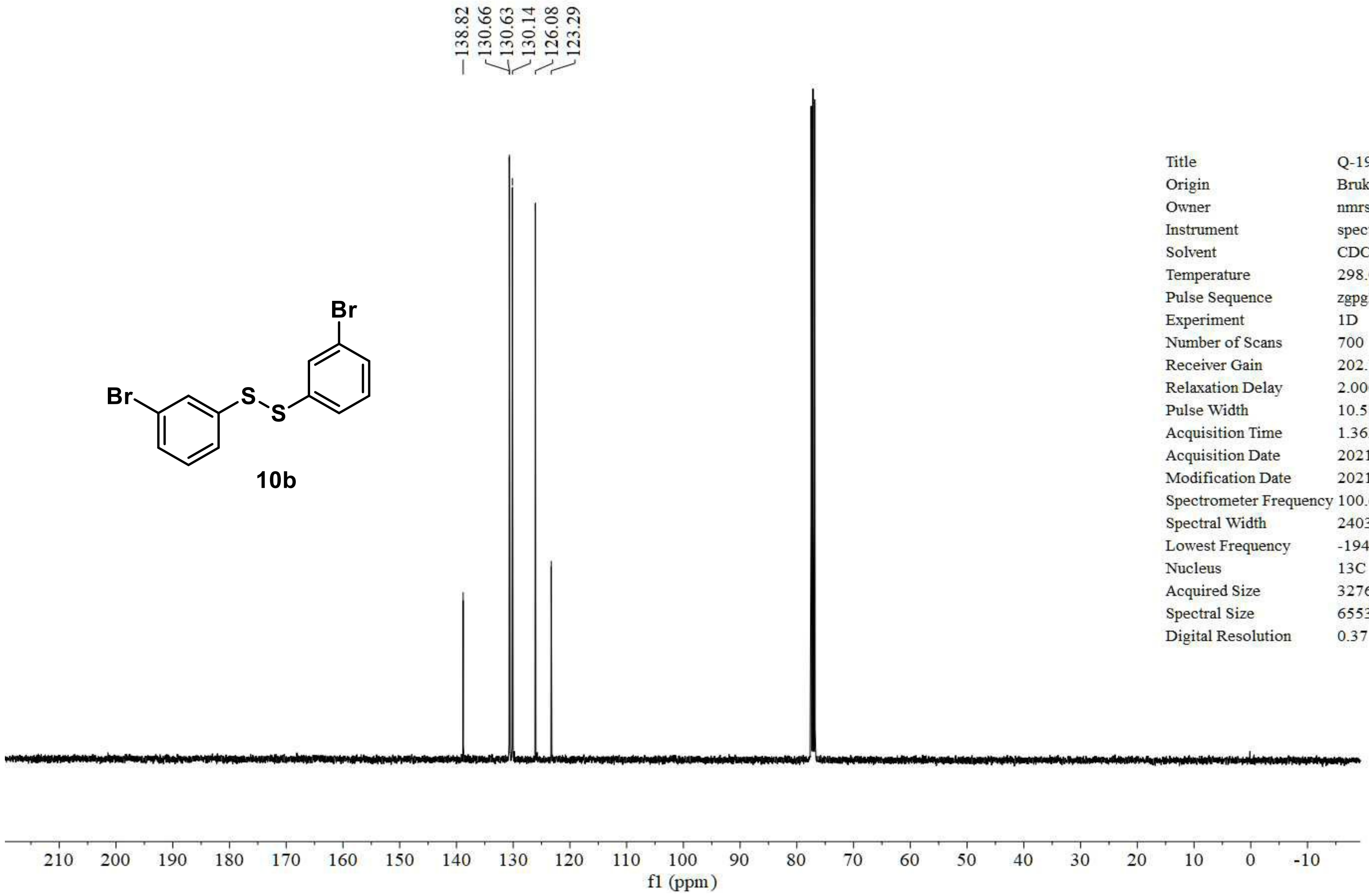

Title	Q-199.2.fid
Origin	Bruker BioSpin GmbH
Owner	nmrsu
Instrument	spect
Solvent	CDCl3
Temperature	298.0
Pulse Sequence	zgpg30
Experiment	1D
Number of Scans	700
Receiver Gain	202.1
Relaxation Delay	2.0000
Pulse Width	10.5800
Acquisition Time	1.3631
Acquisition Date	2021-11-09T19:53:23
Modification Date	2021-11-09T19:53:22
Spectrometer Frequency	100.63
Spectral Width	24038.5
Lowest Frequency	-1944.2
Nucleus	13C
Acquired Size	32768
Spectral Size	65536
Digital Resolution	0.37

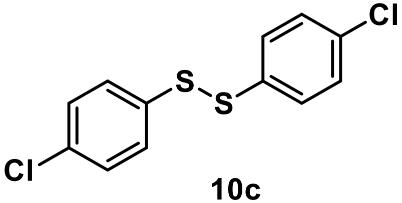

10c

Title	Q-192.2.fid
Origin	Bruker BioSpin GmbH
Owner	nmrsu
Instrument	spect
Solvent	CDCl3
Temperature	298.0
Pulse Sequence	zg30
Experiment	1D
Number of Scans	16
Receiver Gain	72.0
Relaxation Delay	1.0000
Pulse Width	9.7800
Acquisition Time	4.0894
Acquisition Date	2021-09-26T05:15:30
Modification Date	2021-09-26T05:15:32
Spectrometer Frequency	400.15
Spectral Width	8012.8
Lowest Frequency	-1545.8
Nucleus	1H
Acquired Size	32768
Spectral Size	65536
Digital Resolution	0.12

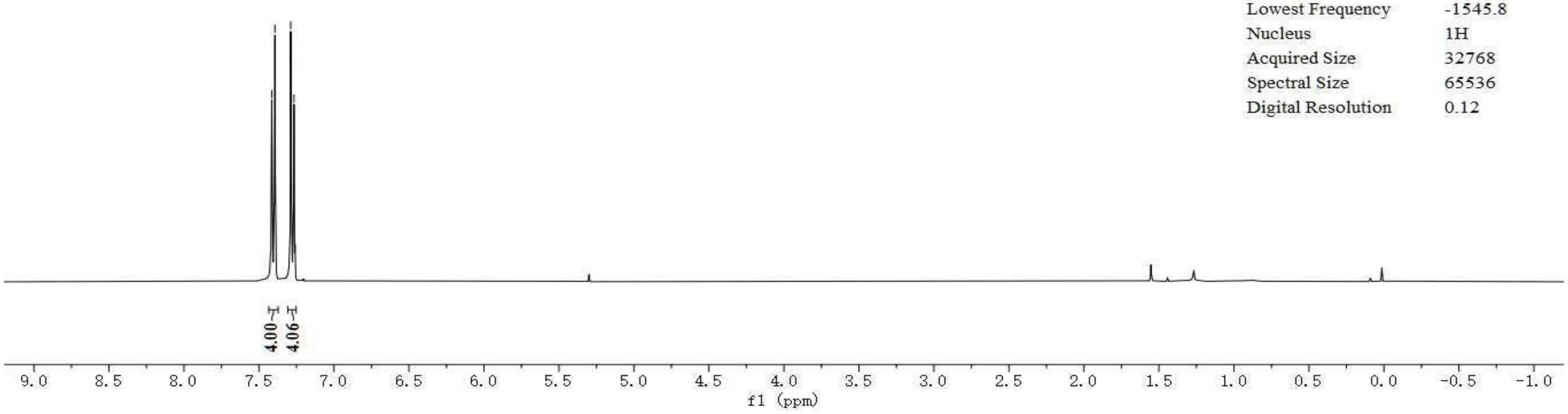

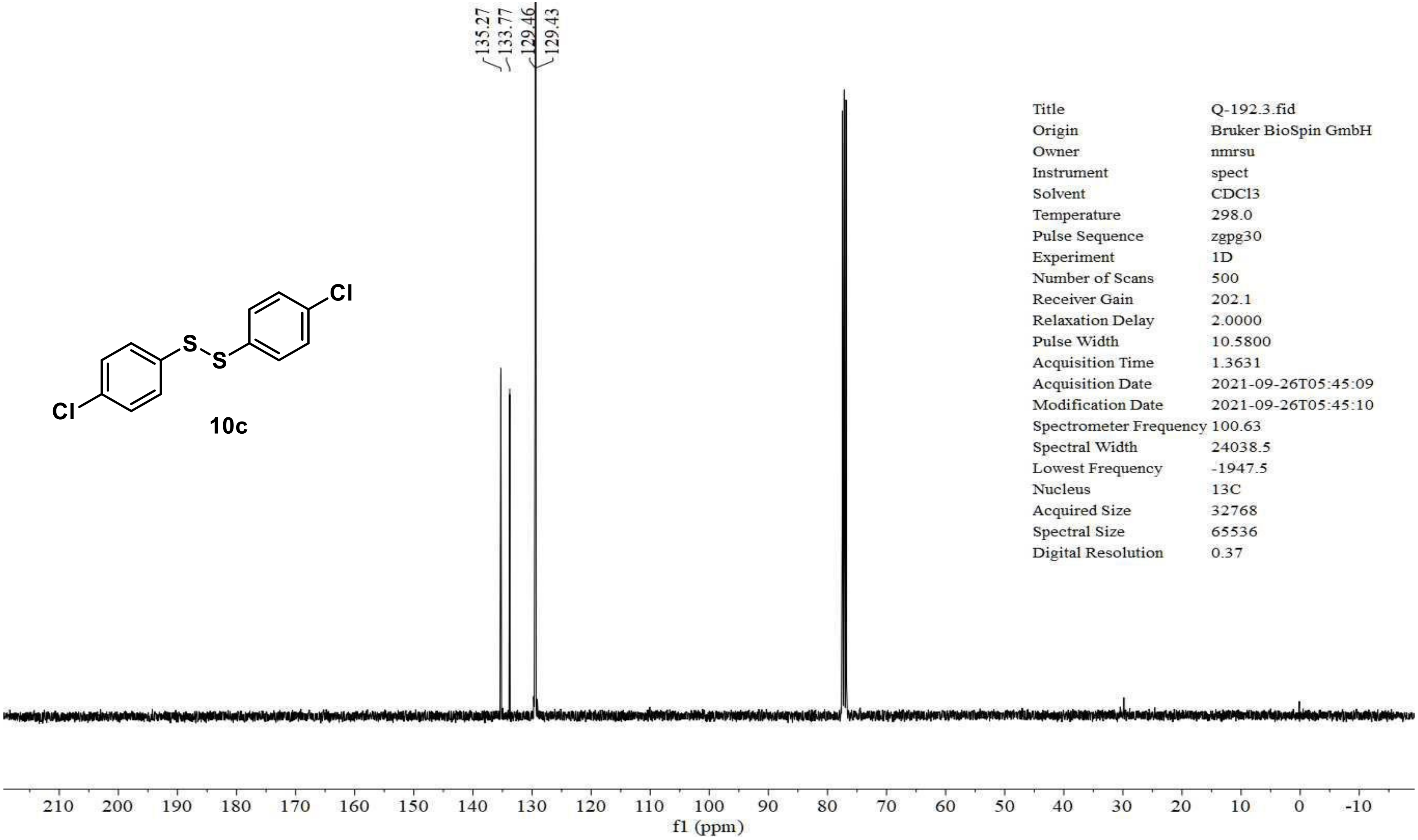

Title	Q-192.3.fid
Origin	Bruker BioSpin GmbH
Owner	nmrsu
Instrument	spect
Solvent	CDCl3
Temperature	298.0
Pulse Sequence	zgpg30
Experiment	1D
Number of Scans	500
Receiver Gain	202.1
Relaxation Delay	2.0000
Pulse Width	10.5800
Acquisition Time	1.3631
Acquisition Date	2021-09-26T05:45:09
Modification Date	2021-09-26T05:45:10
Spectrometer Frequency	100.63
Spectral Width	24038.5
Lowest Frequency	-1947.5
Nucleus	13C
Acquired Size	32768
Spectral Size	65536
Digital Resolution	0.37

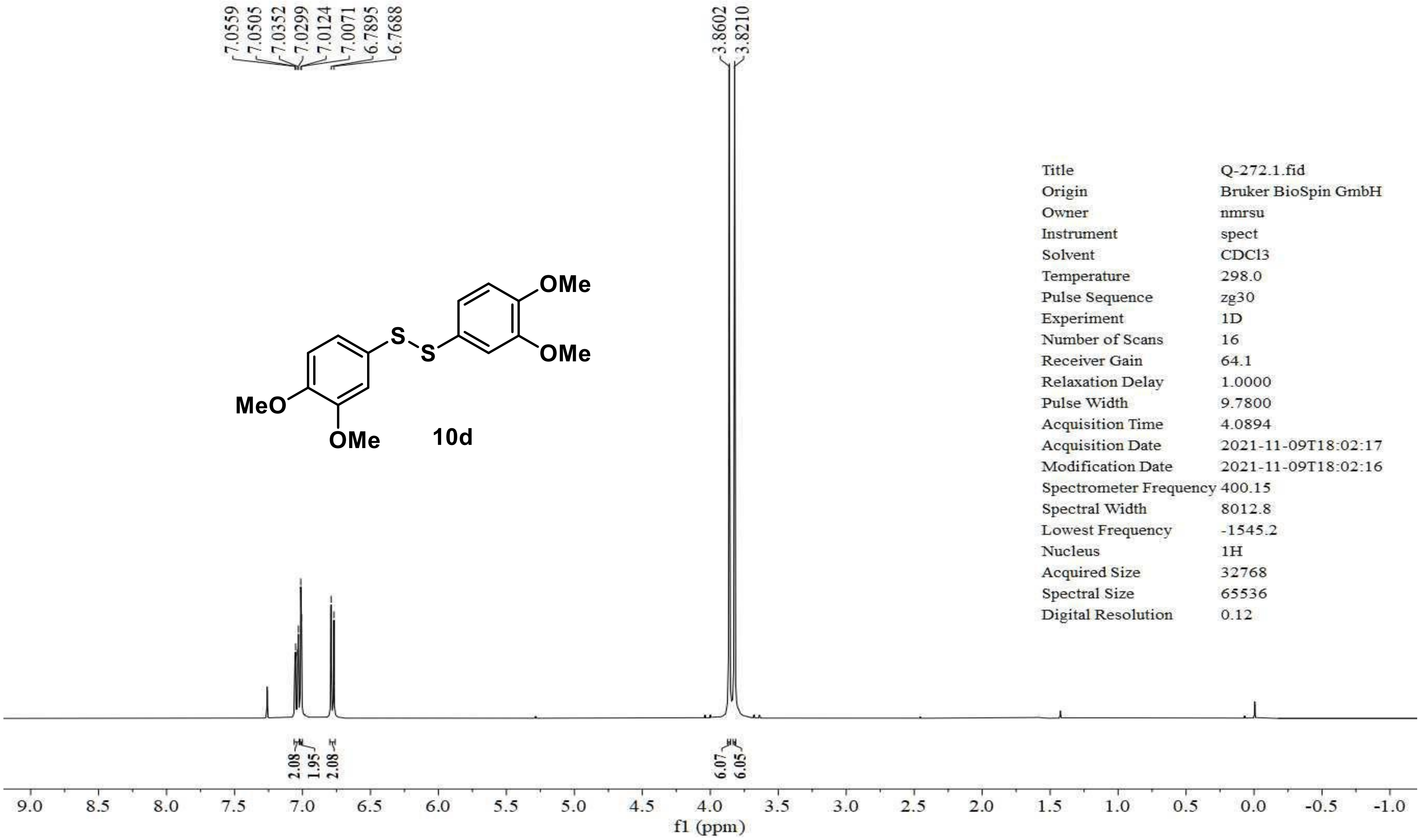

Title	Q-272.1.fid
Origin	Bruker BioSpin GmbH
Owner	nmrsu
Instrument	spect
Solvent	CDCl3
Temperature	298.0
Pulse Sequence	zg30
Experiment	1D
Number of Scans	16
Receiver Gain	64.1
Relaxation Delay	1.0000
Pulse Width	9.7800
Acquisition Time	4.0894
Acquisition Date	2021-11-09T18:02:17
Modification Date	2021-11-09T18:02:16
Spectrometer Frequency	400.15
Spectral Width	8012.8
Lowest Frequency	-1545.2
Nucleus	1H
Acquired Size	32768
Spectral Size	65536
Digital Resolution	0.12

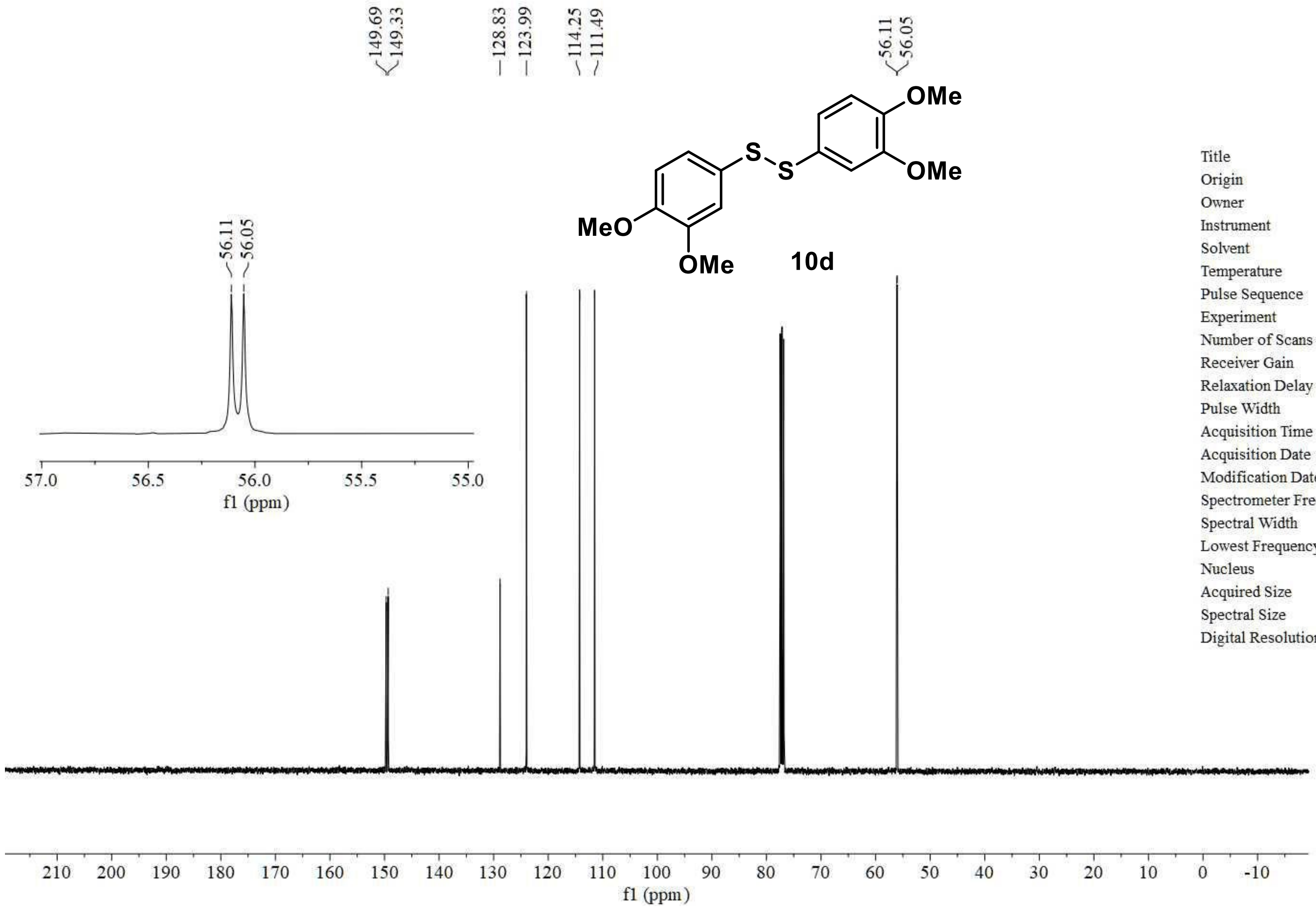

Title	Q-272.2.fid
Origin	Bruker BioSpin GmbH
Owner	nmrsu
Instrument	spect
Solvent	CDCl3
Temperature	298.0
Pulse Sequence	zgpg30
Experiment	1D
Number of Scans	700
Receiver Gain	202.1
Relaxation Delay	2.0000
Pulse Width	10.5800
Acquisition Time	1.3631
Acquisition Date	2021-11-09T18:43:28
Modification Date	2021-11-09T18:43:28
Spectrometer Frequency	100.63
Spectral Width	24038.5
Lowest Frequency	-1946.7
Nucleus	13C
Acquired Size	32768
Spectral Size	65536
Digital Resolution	0.37

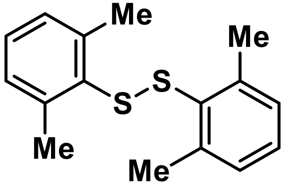

10e

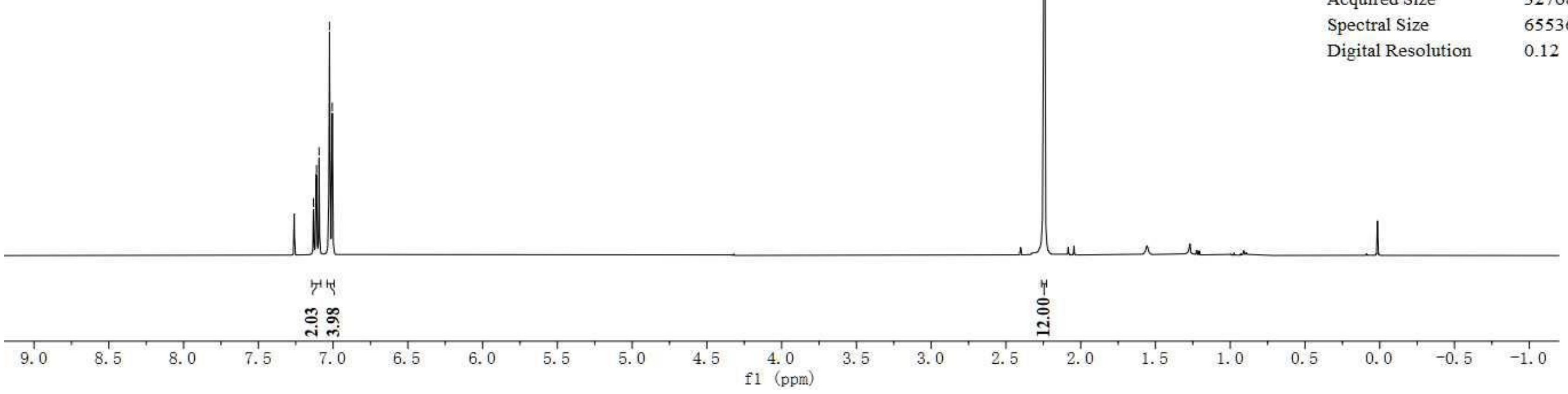

Title	Q-203.1.fid
Origin	Bruker BioSpin GmbH
Owner	nmrsu
Instrument	spect
Solvent	CDCl3
Temperature	298.4
Pulse Sequence	zg30
Experiment	1D
Number of Scans	16
Receiver Gain	72.0
Relaxation Delay	1.0000
Pulse Width	9.7800
Acquisition Time	4.0894
Acquisition Date	2021-09-05T22:50:46
Modification Date	2021-09-05T22:50:48
Spectrometer Frequency	400.15
Spectral Width	8012.8
Lowest Frequency	-1545.7
Nucleus	1H
Acquired Size	32768
Spectral Size	65536
Digital Resolution	0.12

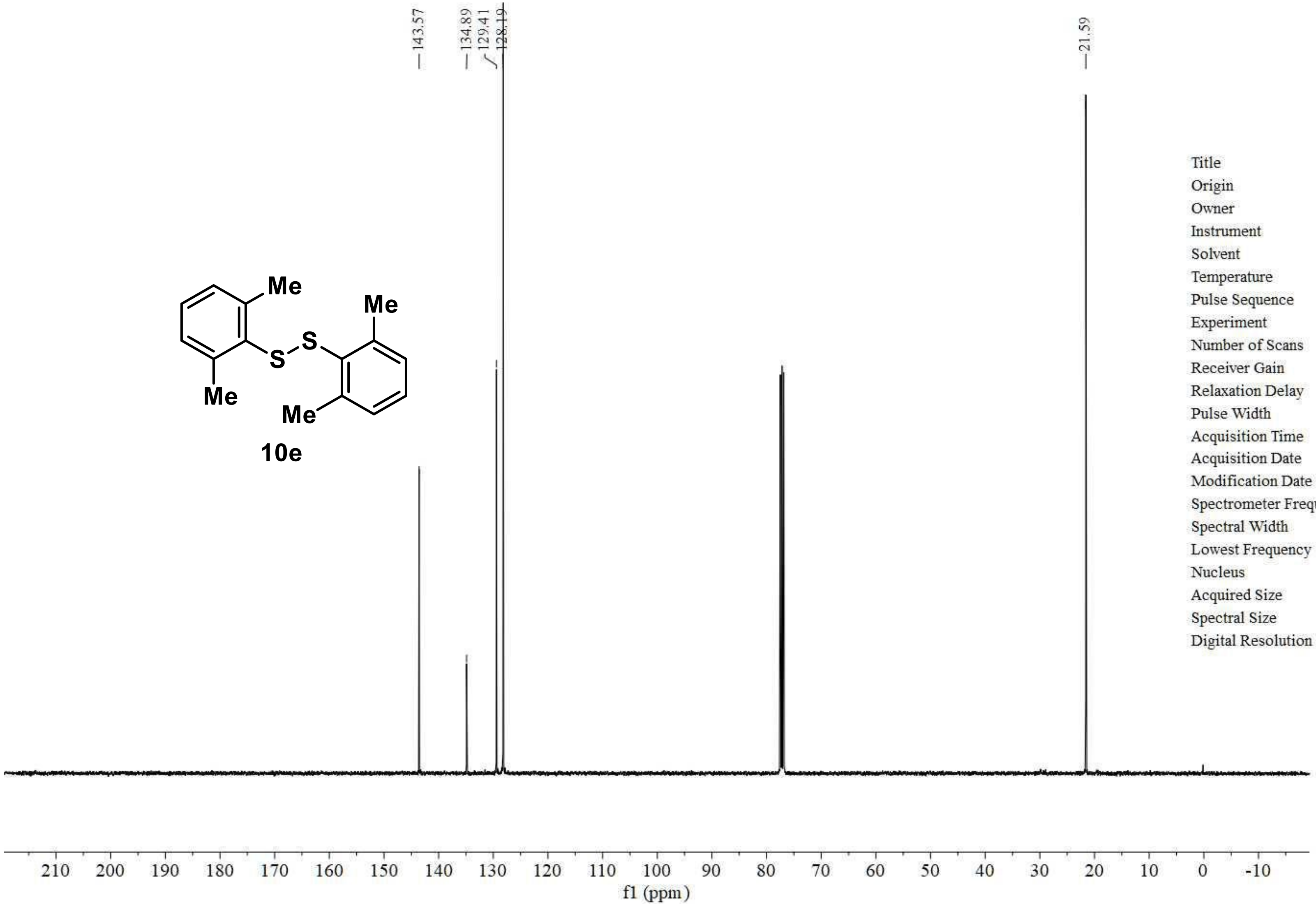

Title	Q-203.2.fid
Origin	Bruker BioSpin GmbH
Owner	nmrsu
Instrument	spect
Solvent	CDCl3
Temperature	299.1
Pulse Sequence	zgpg30
Experiment	1D
Number of Scans	1024
Receiver Gain	202.1
Relaxation Delay	2.0000
Pulse Width	10.5800
Acquisition Time	1.3631
Acquisition Date	2021-09-05T23:50:20
Modification Date	2021-09-05T23:50:22
Spectrometer Frequency	100.63
Spectral Width	24038.5
Lowest Frequency	-1945.2
Nucleus	13C
Acquired Size	32768
Spectral Size	65536
Digital Resolution	0.37

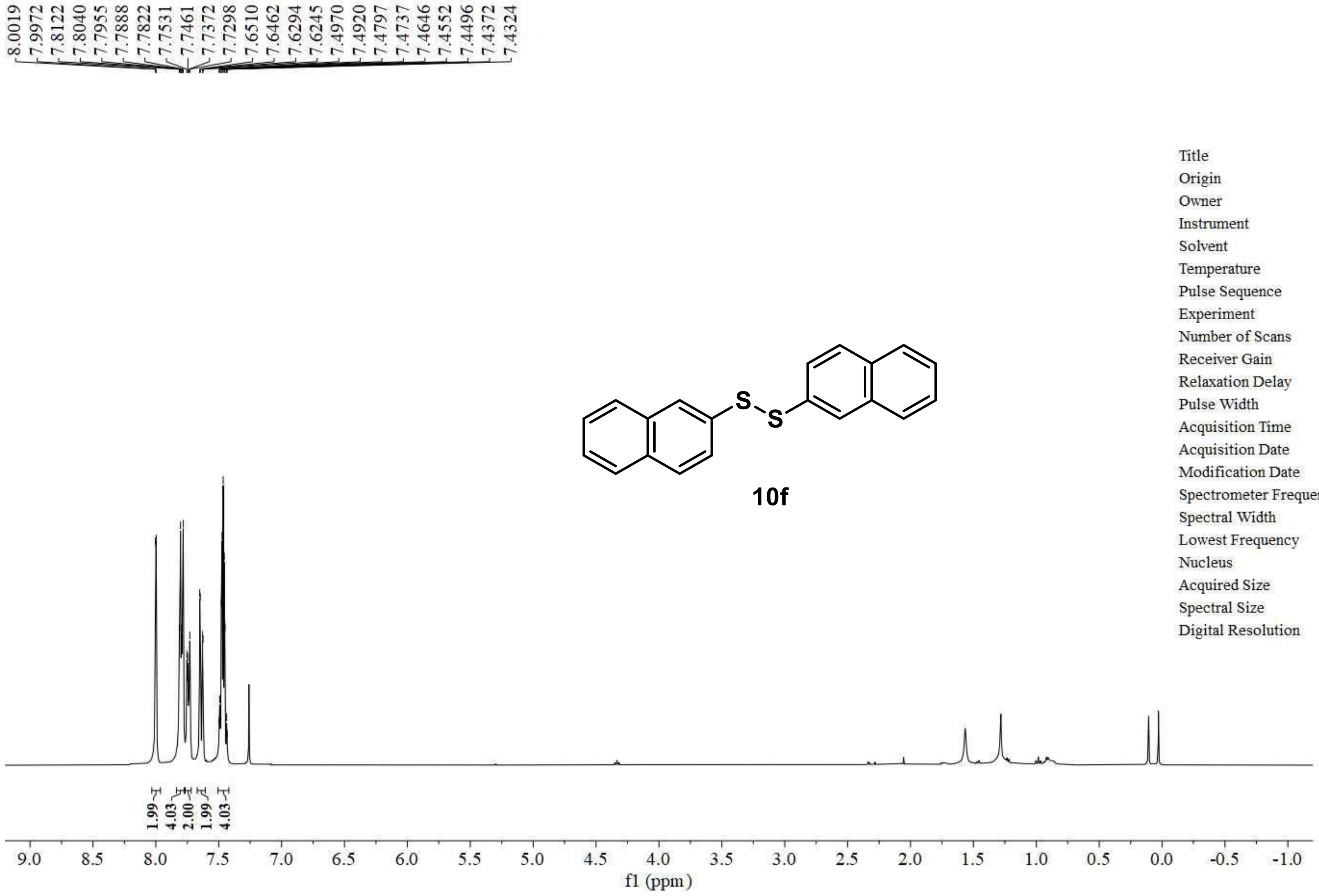

10f

Title	Q-198.1.fid
Origin	Bruker BioSpin GmbH
Owner	nmrsu
Instrument	spect
Solvent	CDCl3
Temperature	298.5
Pulse Sequence	zg30
Experiment	1D
Number of Scans	16
Receiver Gain	79.4
Relaxation Delay	1.0000
Pulse Width	9.7800
Acquisition Time	4.0894
Acquisition Date	2021-09-05T08:08:48
Modification Date	2021-09-05T08:08:50
Spectrometer Frequency	400.15
Spectral Width	8012.8
Lowest Frequency	-1545.9
Nucleus	1H
Acquired Size	32768
Spectral Size	65536
Digital Resolution	0.12

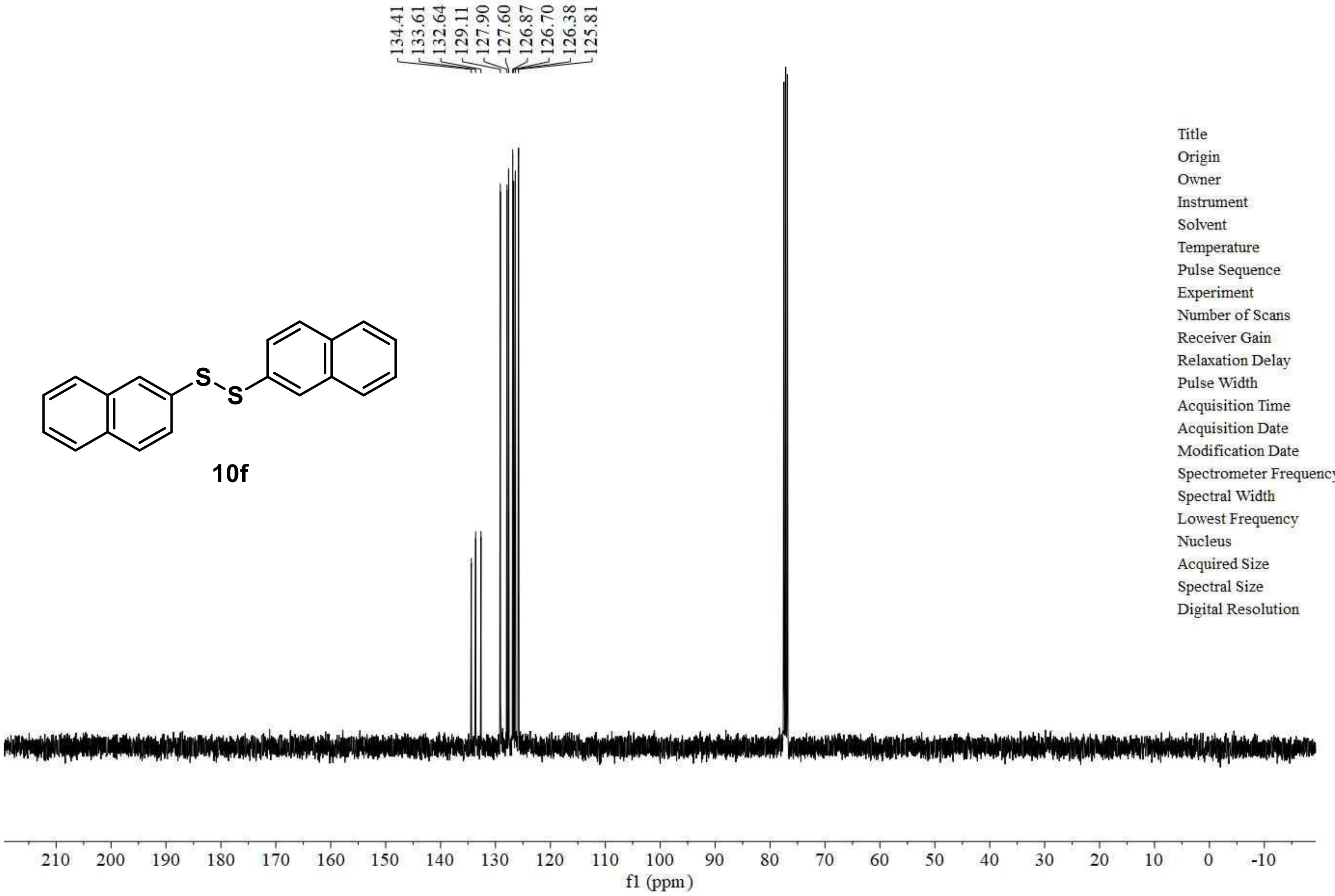

10f

Title	Q-198.2.fid
Origin	Bruker BioSpin GmbH
Owner	nmrsu
Instrument	spect
Solvent	CDCl3
Temperature	299.1
Pulse Sequence	zgpg30
Experiment	1D
Number of Scans	113
Receiver Gain	202.1
Relaxation Delay	2.0000
Pulse Width	10.5800
Acquisition Time	1.3631
Acquisition Date	2021-09-05T08:16:40
Modification Date	2021-09-05T08:16:42
Spectrometer Frequency	100.63
Spectral Width	24038.5
Lowest Frequency	-1947.5
Nucleus	13C
Acquired Size	32768
Spectral Size	65536
Digital Resolution	0.37

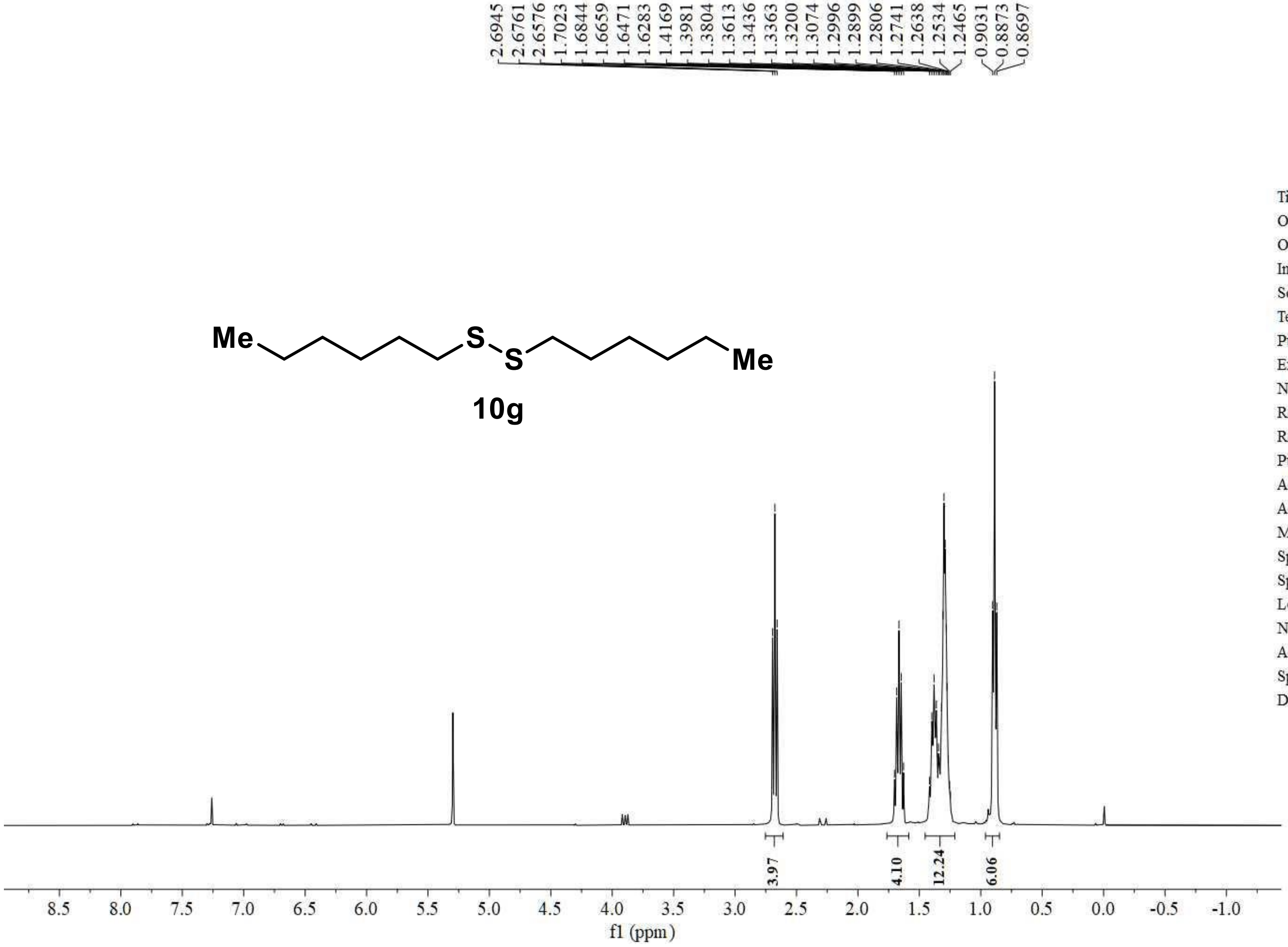

Title	Q-276.12.fid
Origin	Bruker BioSpin GmbH
Owner	nmrsu
Instrument	Avance NEO
Solvent	CDCl3
Temperature	292.1
Pulse Sequence	zg30
Experiment	1D
Number of Scans	16
Receiver Gain	32.0
Relaxation Delay	1.0000
Pulse Width	8.0000
Acquisition Time	3.9977
Acquisition Date	2021-10-29T08:42:05
Modification Date	2021-10-29T08:42:14
Spectrometer Frequency	400.13
Spectral Width	8196.7
Lowest Frequency	-1636.3
Nucleus	1H
Acquired Size	32768
Spectral Size	65536
Digital Resolution	0.13

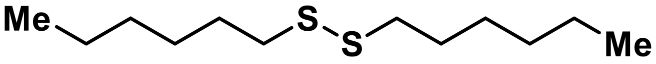

10g

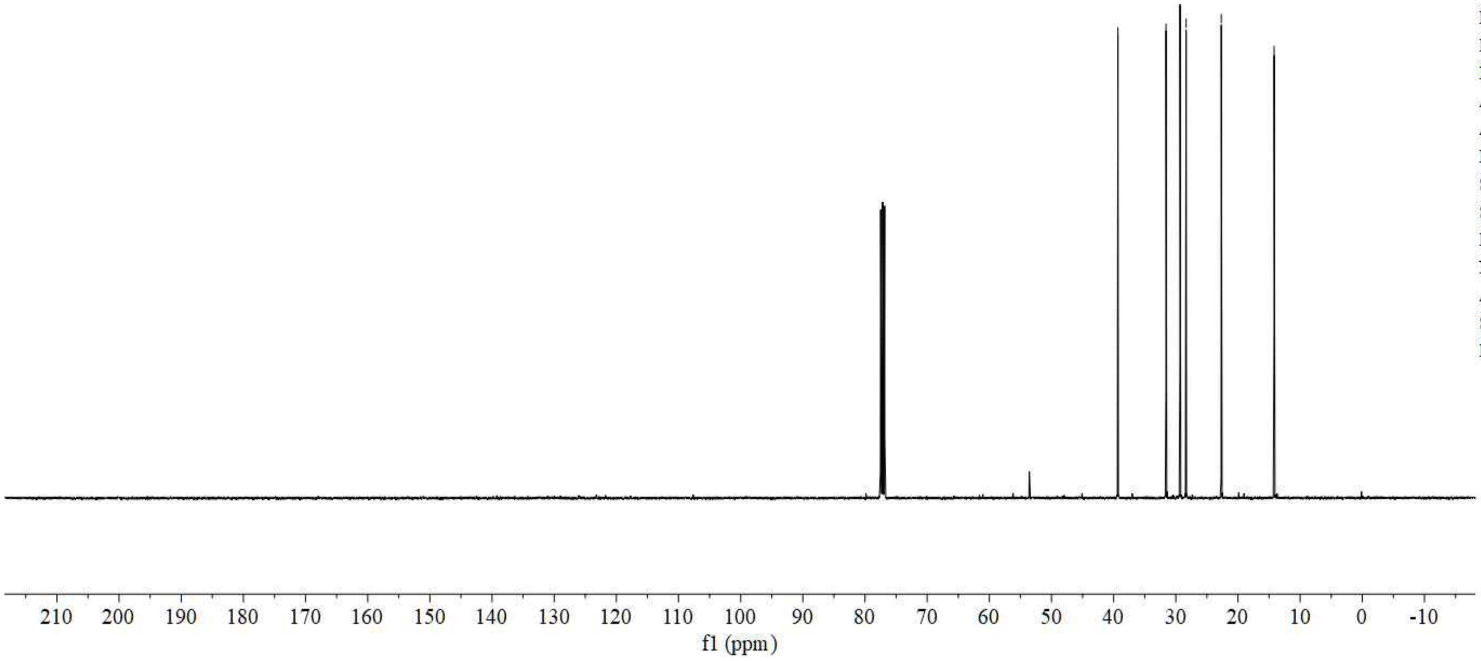

Title	Q-276.13.fid
Origin	Bruker BioSpin GmbH
Owner	nmrsu
Instrument	Avance NEO
Solvent	CDCl3
Temperature	292.8
Pulse Sequence	zgpg30
Experiment	1D
Number of Scans	1024
Receiver Gain	101.0
Relaxation Delay	2.0000
Pulse Width	8.0000
Acquisition Time	1.3763
Acquisition Date	2021-10-29T09:41:25
Modification Date	2021-10-29T09:41:34
Spectrometer Frequency	100.62
Spectral Width	23809.5
Lowest Frequency	-1832.5
Nucleus	13C
Acquired Size	32768
Spectral Size	65536
Digital Resolution	0.36

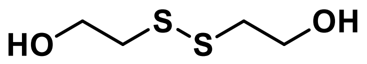

10h

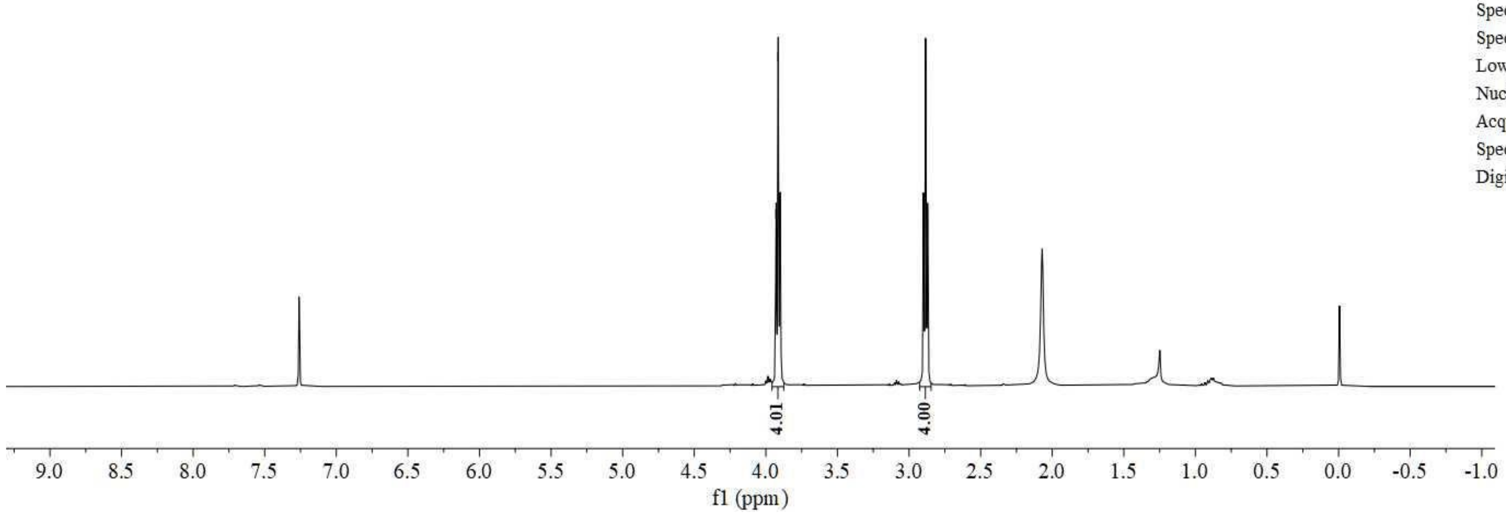

Title	Q-218.3.fid
Origin	Bruker BioSpin GmbH
Owner	nmrsu
Instrument	Avance NEO
Solvent	CDCl3
Temperature	297.0
Pulse Sequence	zg30
Experiment	1D
Number of Scans	16
Receiver Gain	101.0
Relaxation Delay	1.0000
Pulse Width	8.0000
Acquisition Time	3.9977
Acquisition Date	2021-10-18T16:17:19
Modification Date	2021-10-18T16:16:40
Spectrometer Frequency	400.13
Spectral Width	8196.7
Lowest Frequency	-1636.3
Nucleus	1H
Acquired Size	32768
Spectral Size	65536
Digital Resolution	0.13

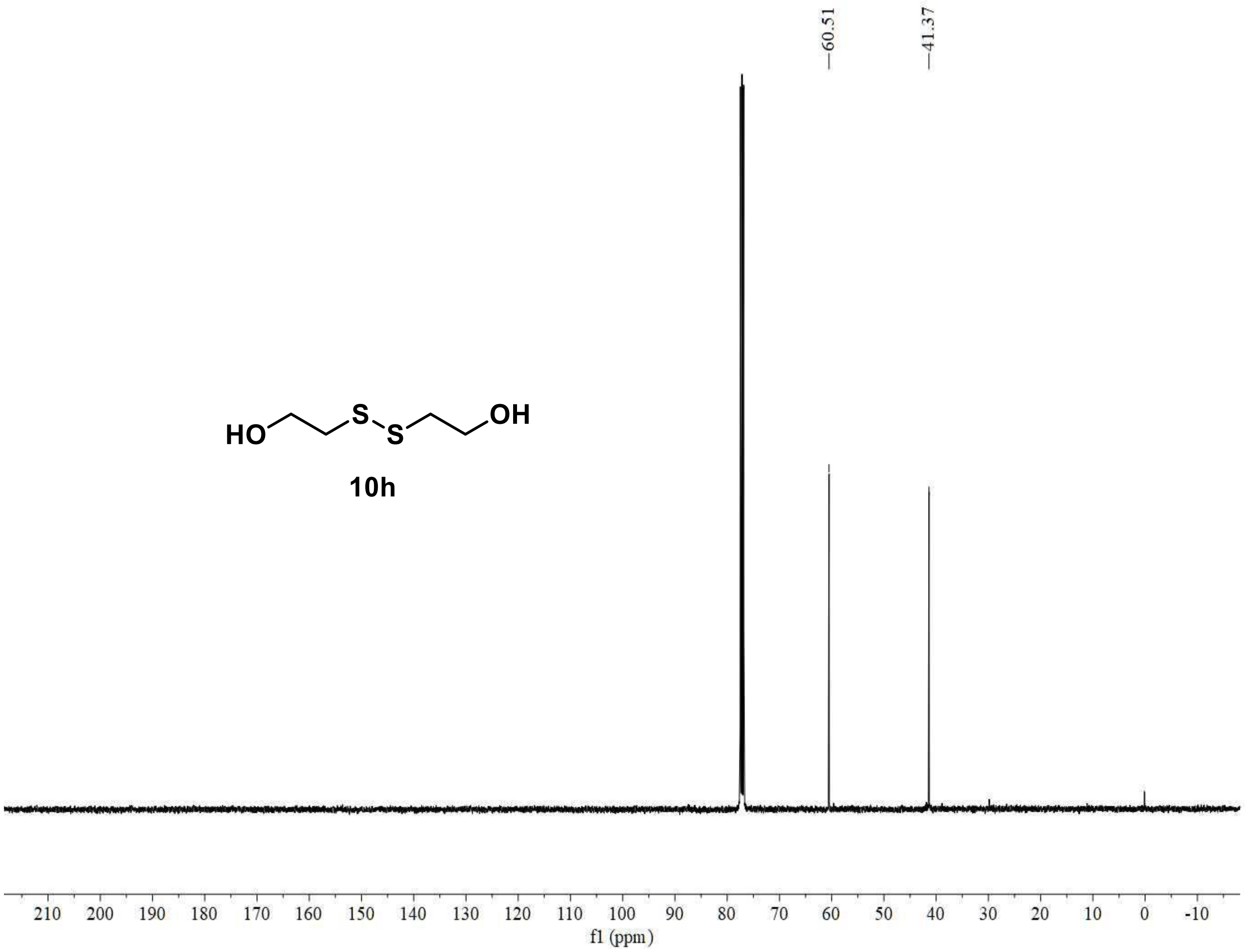

Title	Q-218.4.fid
Origin	Bruker BioSpin GmbH
Owner	nmrsu
Instrument	Avance NEO
Solvent	CDCl3
Temperature	297.8
Pulse Sequence	zgpg30
Experiment	1D
Number of Scans	1024
Receiver Gain	101.0
Relaxation Delay	2.0000
Pulse Width	8.0000
Acquisition Time	1.3763
Acquisition Date	2021-10-18T17:16:42
Modification Date	2021-10-18T17:16:02
Spectrometer Frequency	100.62
Spectral Width	23809.5
Lowest Frequency	-1830.6
Nucleus	13C
Acquired Size	32768
Spectral Size	65536
Digital Resolution	0.36

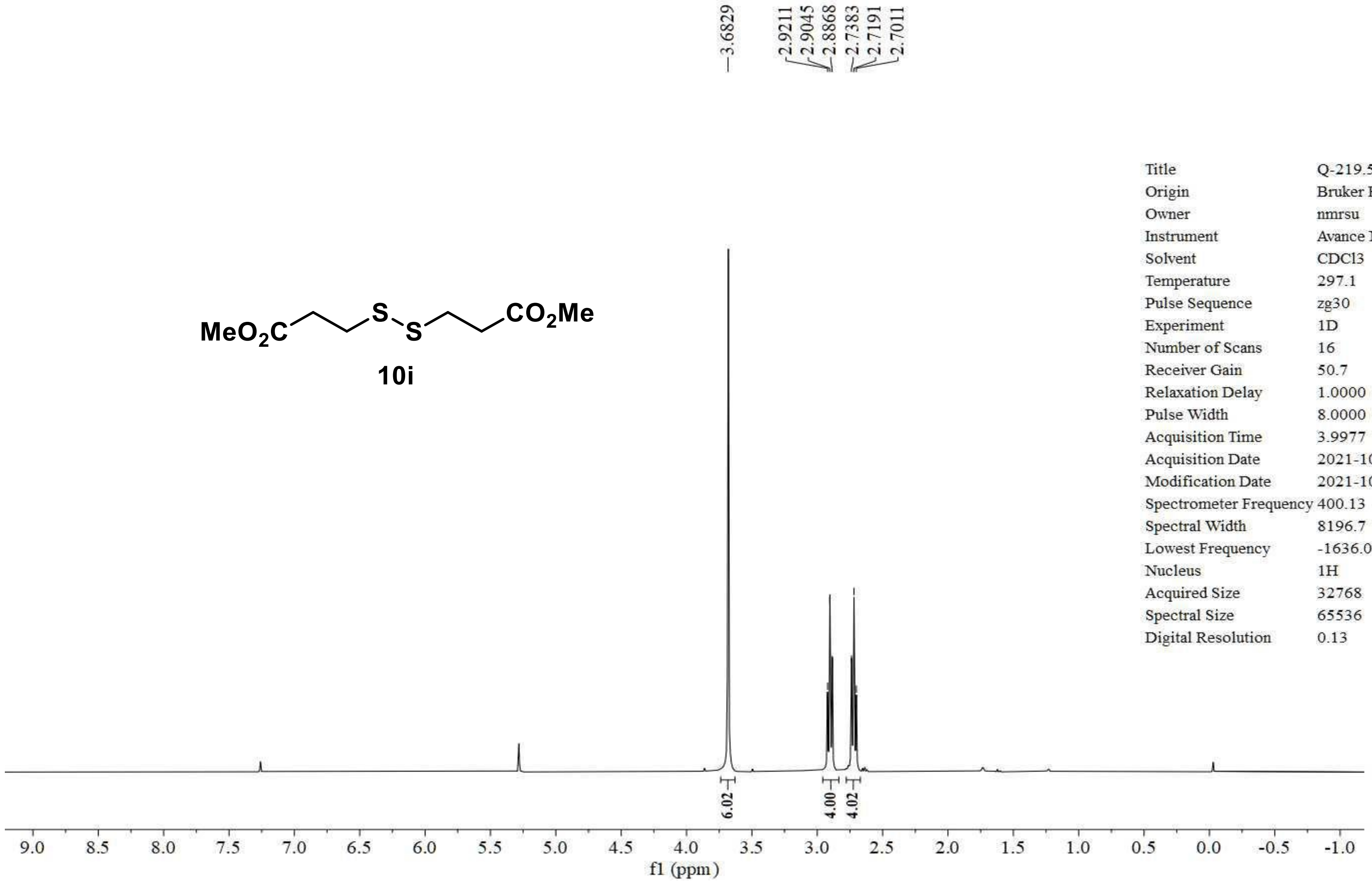

Title	Q-219.5.fid
Origin	Bruker BioSpin GmbH
Owner	nmrsu
Instrument	Avance NEO
Solvent	CDCl3
Temperature	297.1
Pulse Sequence	zg30
Experiment	1D
Number of Scans	16
Receiver Gain	50.7
Relaxation Delay	1.0000
Pulse Width	8.0000
Acquisition Time	3.9977
Acquisition Date	2021-10-18T17:22:20
Modification Date	2021-10-18T17:21:42
Spectrometer Frequency	400.13
Spectral Width	8196.7
Lowest Frequency	-1636.0
Nucleus	1H
Acquired Size	32768
Spectral Size	65536
Digital Resolution	0.13

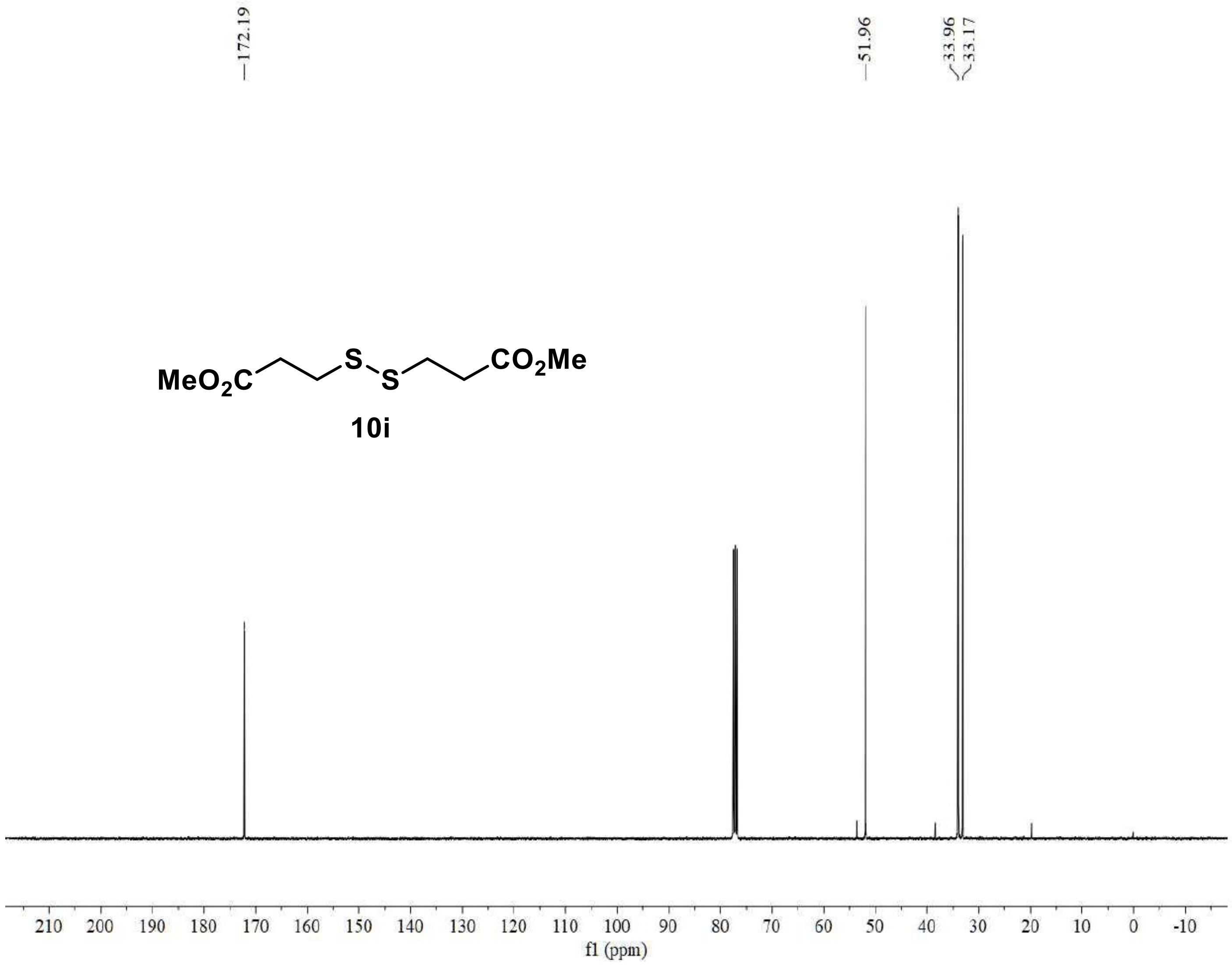

Title	Q-219.6.fid
Origin	Bruker BioSpin GmbH
Owner	nmrsu
Instrument	Avance NEO
Solvent	CDCl3
Temperature	297.8
Pulse Sequence	zgpg30
Experiment	1D
Number of Scans	1024
Receiver Gain	101.0
Relaxation Delay	2.0000
Pulse Width	8.0000
Acquisition Time	1.3763
Acquisition Date	2021-10-18T18:21:51
Modification Date	2021-10-18T18:21:12
Spectrometer Frequency	100.62
Spectral Width	23809.5
Lowest Frequency	-1834.6
Nucleus	13C
Acquired Size	32768
Spectral Size	65536
Digital Resolution	0.36

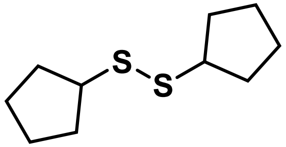

10j

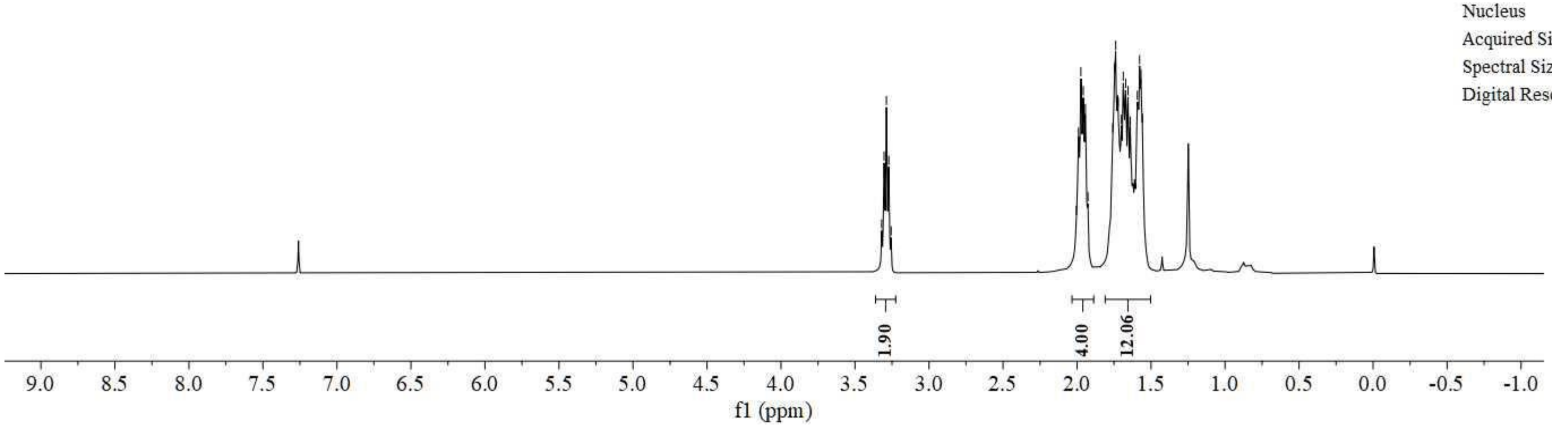

Title	Q-221.9.fid
Origin	Bruker BioSpin GmbH
Owner	nmrsu
Instrument	Avance NEO
Solvent	CDCl3
Temperature	297.2
Pulse Sequence	zg30
Experiment	1D
Number of Scans	16
Receiver Gain	48.4
Relaxation Delay	1.0000
Pulse Width	8.0000
Acquisition Time	3.9977
Acquisition Date	2021-10-18T19:35:03
Modification Date	2021-10-18T19:34:24
Spectrometer Frequency	400.13
Spectral Width	8196.7
Lowest Frequency	-1636.4
Nucleus	1H
Acquired Size	32768
Spectral Size	65536
Digital Resolution	0.13

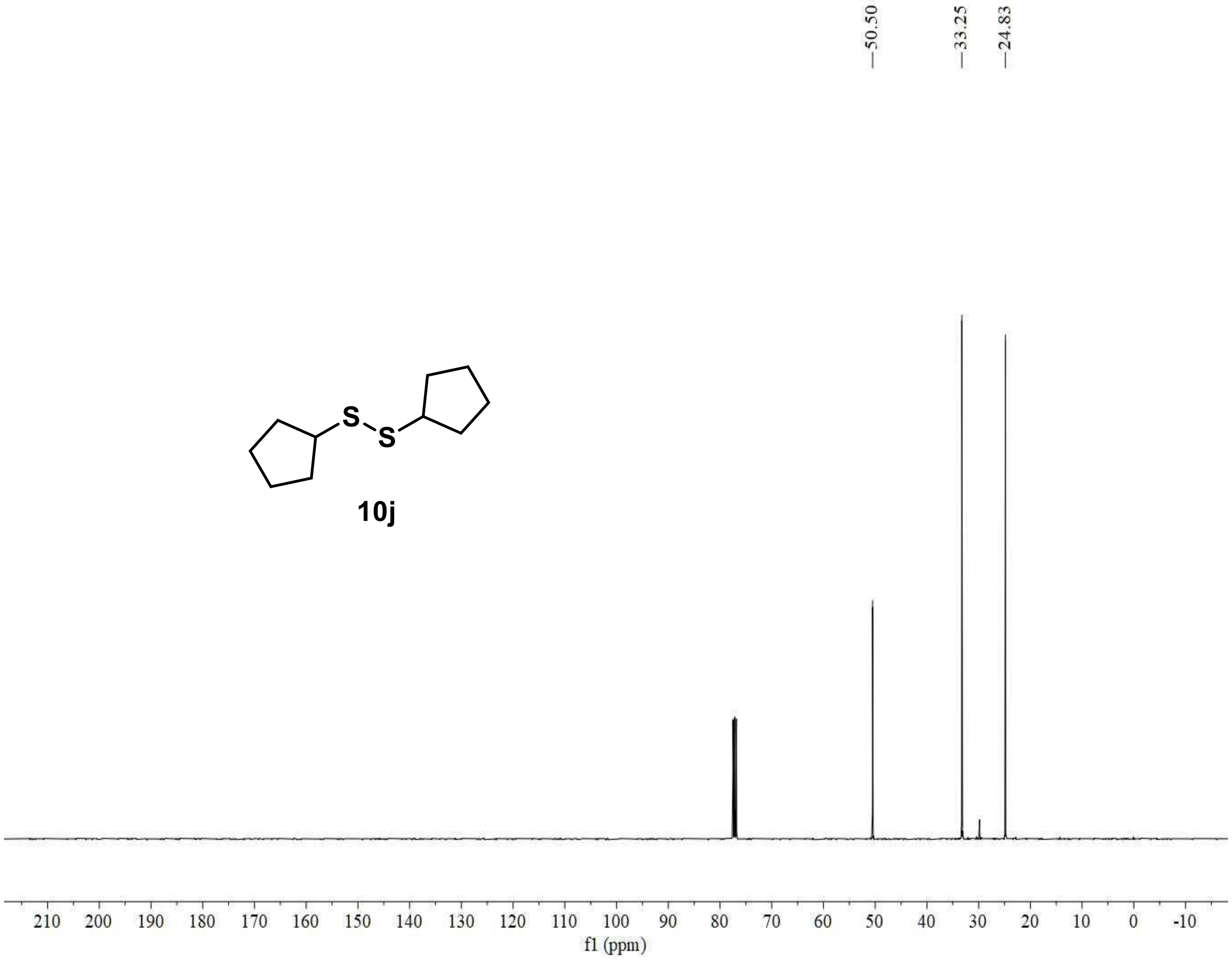

10j

Title	Q-221.10.fid
Origin	Bruker BioSpin GmbH
Owner	nmrsu
Instrument	Avance NEO
Solvent	CDCl3
Temperature	297.8
Pulse Sequence	zgpg30
Experiment	1D
Number of Scans	1024
Receiver Gain	101.0
Relaxation Delay	2.0000
Pulse Width	8.0000
Acquisition Time	1.3763
Acquisition Date	2021-10-18T20:34:26
Modification Date	2021-10-18T20:33:48
Spectrometer Frequency	100.62
Spectral Width	23809.5
Lowest Frequency	-1832.1
Nucleus	13C
Acquired Size	32768
Spectral Size	65536
Digital Resolution	0.36

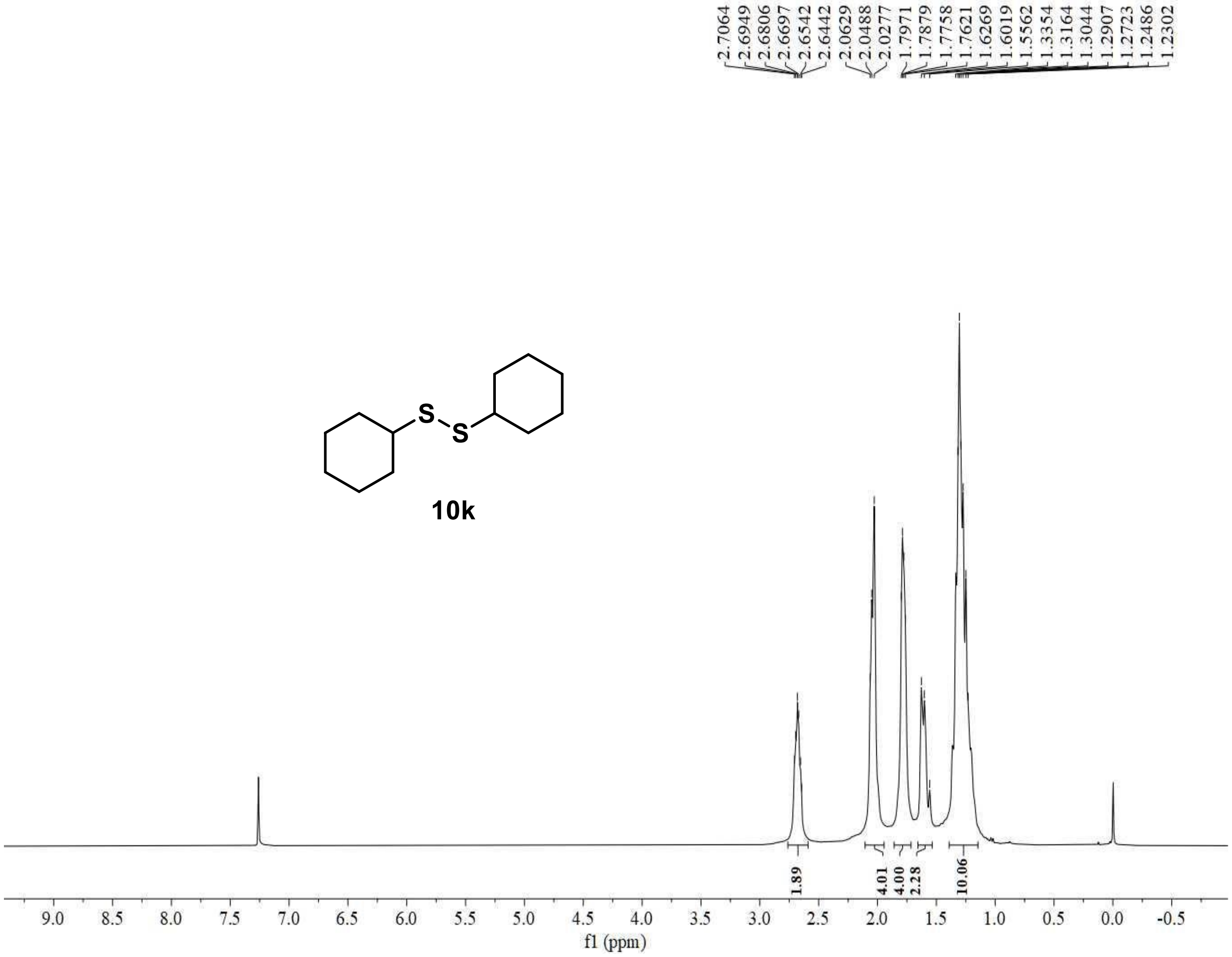

Title	Q-179.1.fid
Origin	Bruker BioSpin GmbH
Owner	nmrsu
Instrument	Avance NEO
Solvent	CDCl3
Temperature	296.9
Pulse Sequence	zg30
Experiment	1D
Number of Scans	16
Receiver Gain	63.0
Relaxation Delay	1.0000
Pulse Width	8.0000
Acquisition Time	3.9977
Acquisition Date	2021-10-18T15:07:18
Modification Date	2021-10-18T15:06:38
Spectrometer Frequency	400.13
Spectral Width	8196.7
Lowest Frequency	-1635.8
Nucleus	1H
Acquired Size	32768
Spectral Size	65536
Digital Resolution	0.13

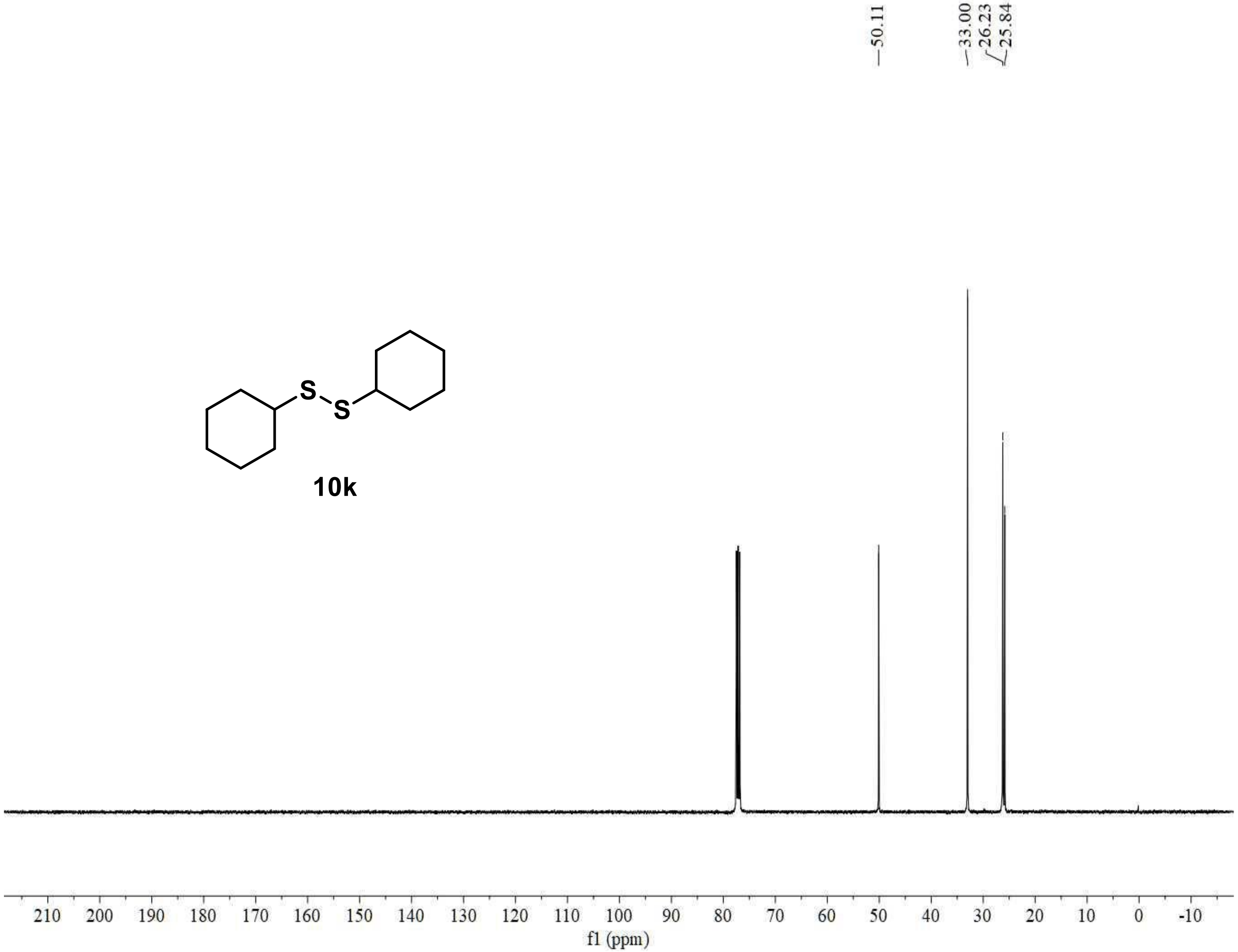

Title	Q-179.2.fid
Origin	Bruker BioSpin GmbH
Owner	nmrsu
Instrument	Avance NEO
Solvent	CDCl3
Temperature	297.6
Pulse Sequence	zgpg30
Experiment	1D
Number of Scans	1024
Receiver Gain	101.0
Relaxation Delay	2.0000
Pulse Width	8.0000
Acquisition Time	1.3763
Acquisition Date	2021-10-18T16:08:03
Modification Date	2021-10-18T16:07:24
Spectrometer Frequency	100.62
Spectral Width	23809.5
Lowest Frequency	-1830.6
Nucleus	13C
Acquired Size	32768
Spectral Size	65536
Digital Resolution	0.36

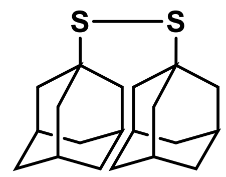

10l

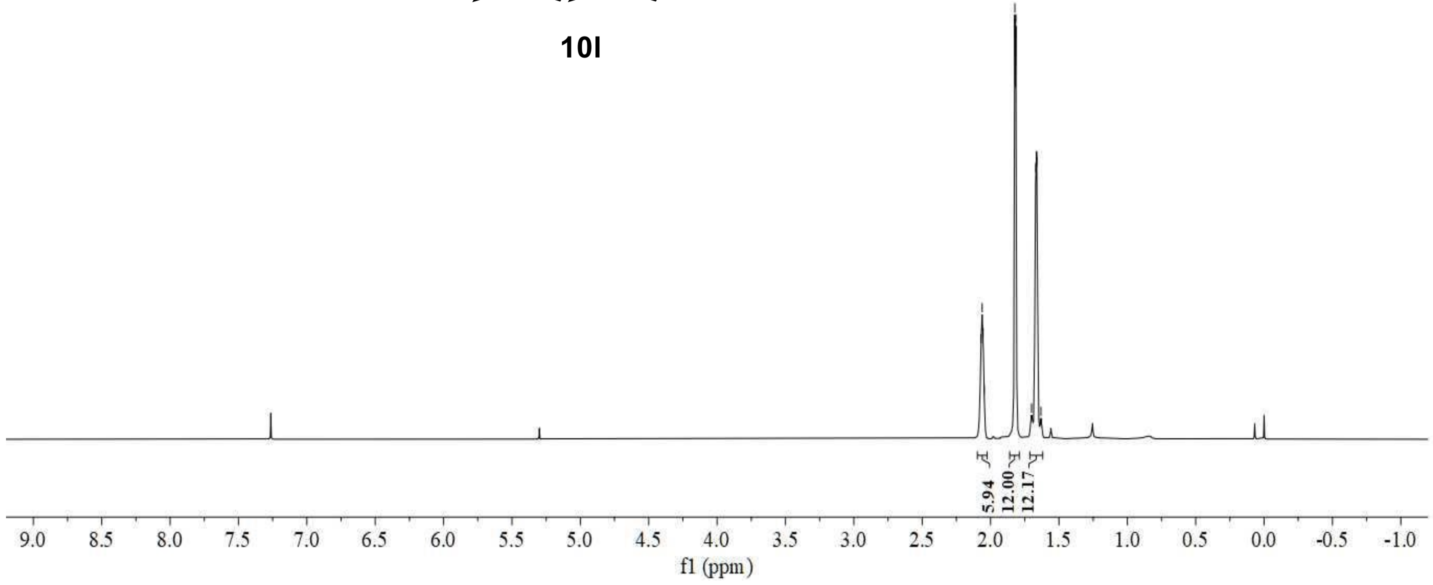

Title	Q-206.2.fid
Origin	Bruker BioSpin GmbH
Owner	nmrsu
Instrument	spect
Solvent	CDCl3
Temperature	298.0
Pulse Sequence	zg30
Experiment	1D
Number of Scans	16
Receiver Gain	30.5
Relaxation Delay	1.0000
Pulse Width	9.7800
Acquisition Time	4.0894
Acquisition Date	2021-09-26T01:15:17
Modification Date	2021-09-26T01:15:18
Spectrometer Frequency	400.15
Spectral Width	8012.8
Lowest Frequency	-1544.3
Nucleus	1H
Acquired Size	32768
Spectral Size	65536
Digital Resolution	0.12

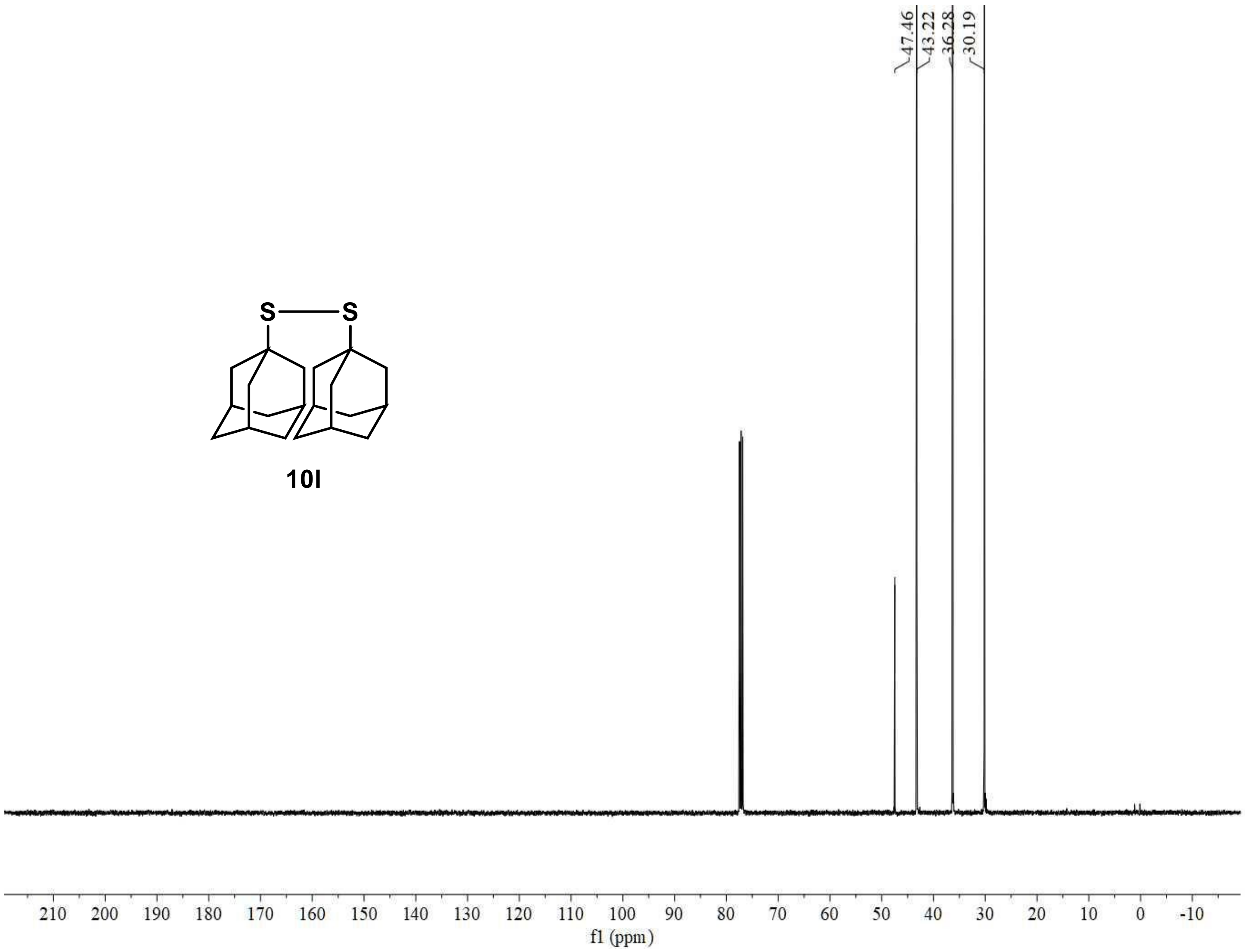

10l

Title	Q-206.3.fid
Origin	Bruker BioSpin GmbH
Owner	nmrsu
Instrument	spect
Solvent	CDCl3
Temperature	298.0
Pulse Sequence	zgpg30
Experiment	1D
Number of Scans	600
Receiver Gain	202.1
Relaxation Delay	2.0000
Pulse Width	10.5800
Acquisition Time	1.3631
Acquisition Date	2021-09-26T01:50:38
Modification Date	2021-09-26T01:50:40
Spectrometer Frequency	100.63
Spectral Width	24038.5
Lowest Frequency	-1945.6
Nucleus	13C
Acquired Size	32768
Spectral Size	65536
Digital Resolution	0.37

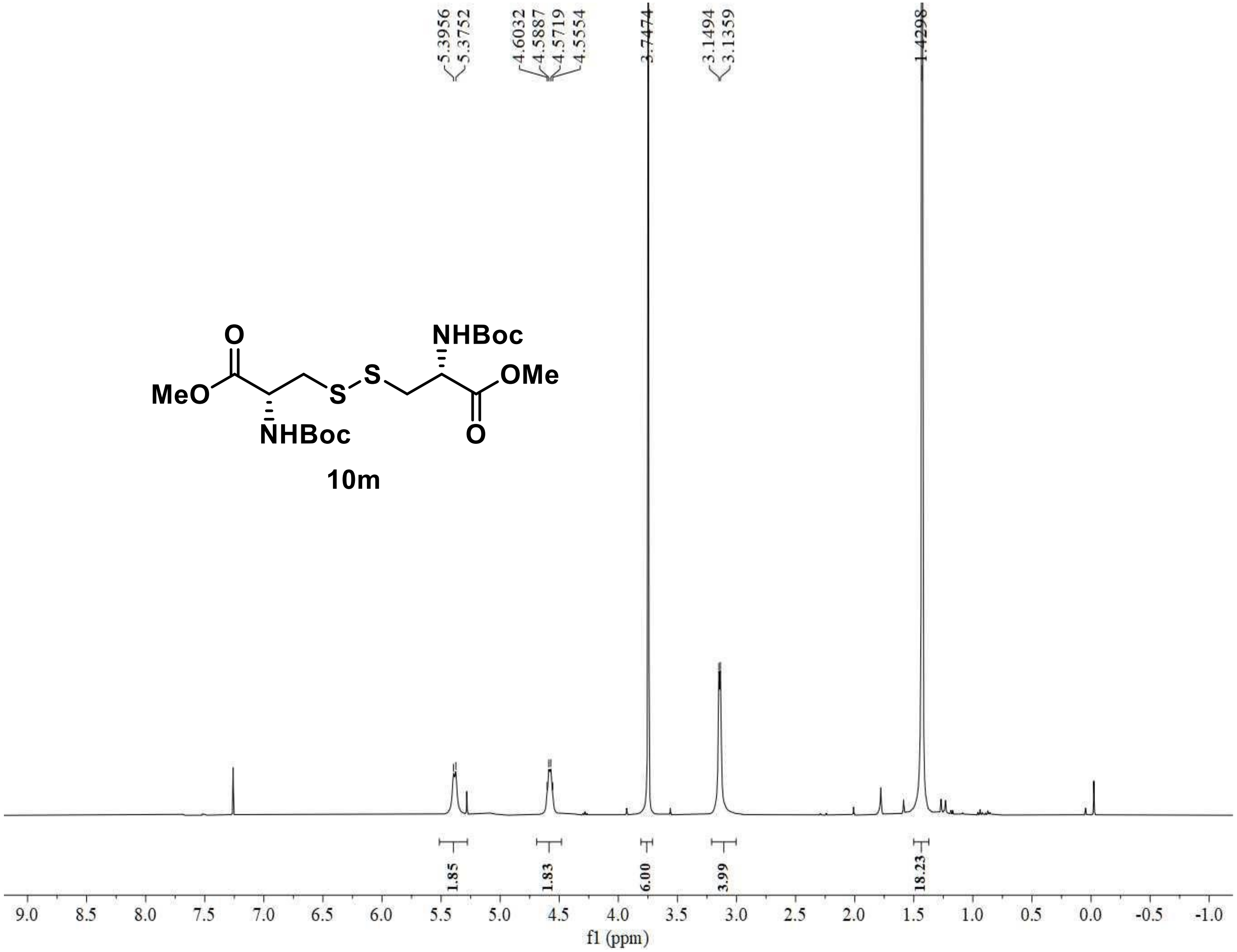

10m

Title	Q-223.1.fid
Origin	Bruker BioSpin GmbH
Owner	nmrsu
Instrument	spect
Solvent	CDCl3
Temperature	298.0
Pulse Sequence	zg30
Experiment	1D
Number of Scans	16
Receiver Gain	30.5
Relaxation Delay	1.0000
Pulse Width	9.7800
Acquisition Time	4.0894
Acquisition Date	2021-09-25T08:07:35
Modification Date	2021-09-25T08:07:36
Spectrometer Frequency	400.15
Spectral Width	8012.8
Lowest Frequency	-1545.9
Nucleus	1H
Acquired Size	32768
Spectral Size	65536
Digital Resolution	0.12

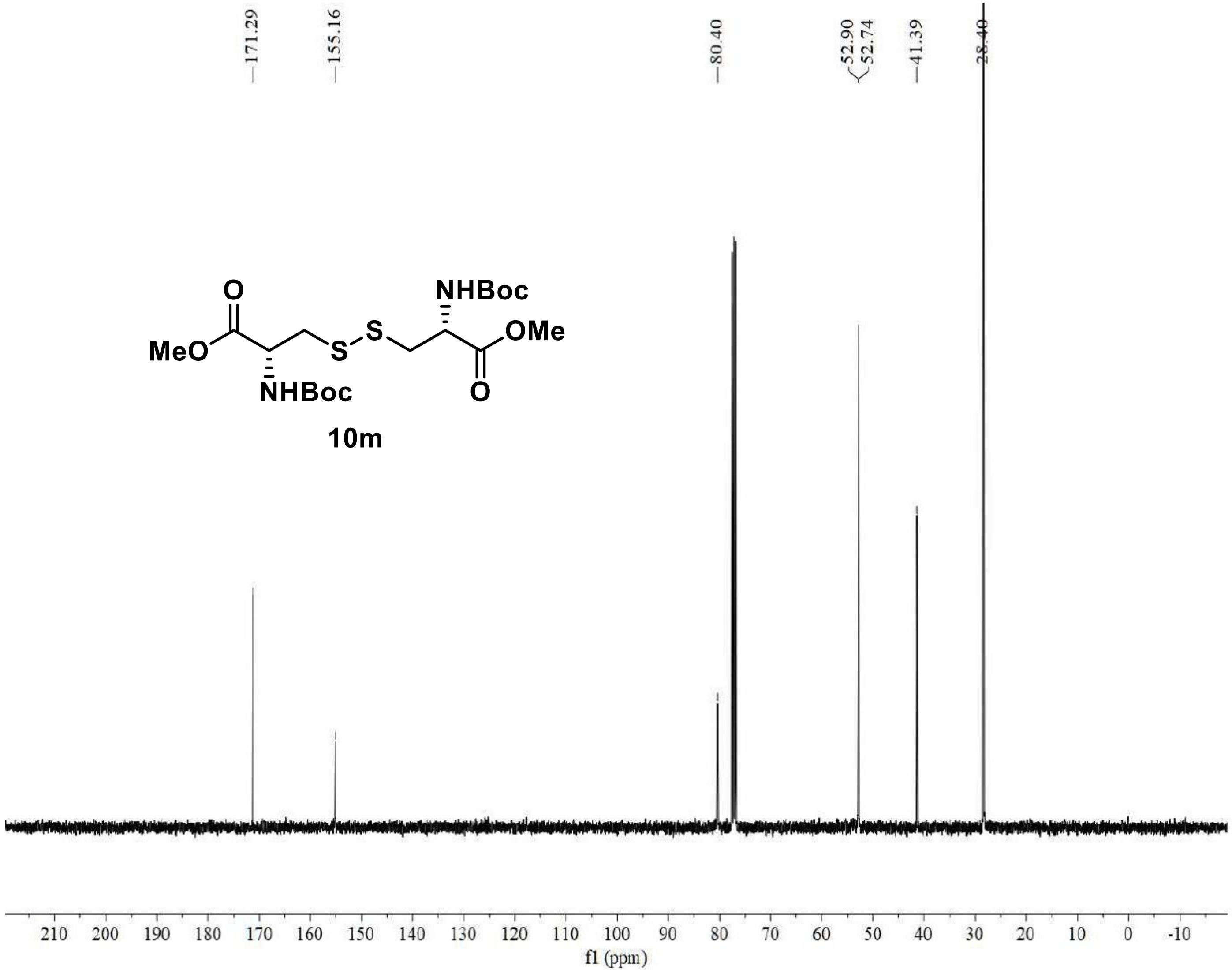

Title	Q-223.2.fid
Origin	Bruker BioSpin GmbH
Owner	nmrsu
Instrument	spect
Solvent	CDCl3
Temperature	298.0
Pulse Sequence	zgpg30
Experiment	1D
Number of Scans	234
Receiver Gain	202.1
Relaxation Delay	2.0000
Pulse Width	10.5800
Acquisition Time	1.3631
Acquisition Date	2021-09-25T08:22:02
Modification Date	2021-09-25T08:22:04
Spectrometer Frequency	100.63
Spectral Width	24038.5
Lowest Frequency	-1947.1
Nucleus	13C
Acquired Size	32768
Spectral Size	65536
Digital Resolution	0.37

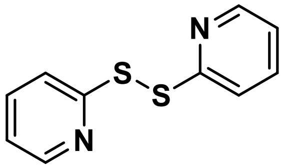

10n

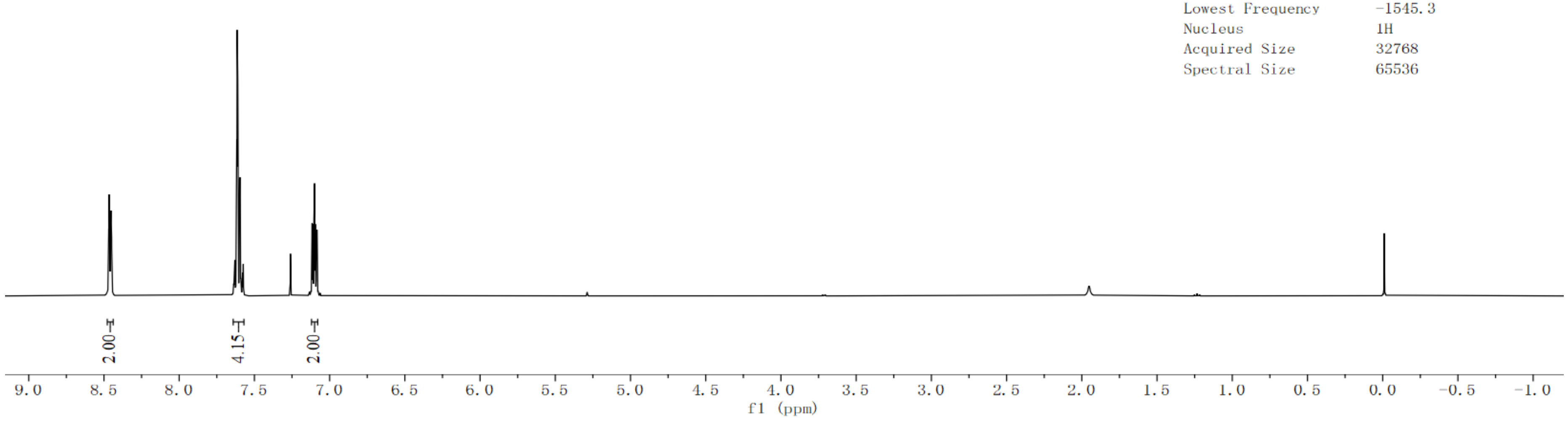

Parameter	Value
Title	ZSS.1.fid
Origin	Bruker BioSpin GmbH
Owner	nmrsu
Instrument	spect
Solvent	CDCl3
Temperature	299.9
Pulse Sequence	zg30
Experiment	1D
Number of Scans	16
Receiver Gain	127.2
Relaxation Delay	1.0000
Pulse Width	9.6200
Acquisition Time	4.0894
Acquisition Date	2024-07-16T11:30:06
Modification Date	2024-07-16T11:30:08
Spectrometer Frequency	400.15
Spectral Width	8012.8
Lowest Frequency	-1545.3
Nucleus	1H
Acquired Size	32768
Spectral Size	65536

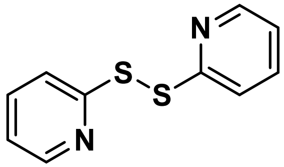

10n

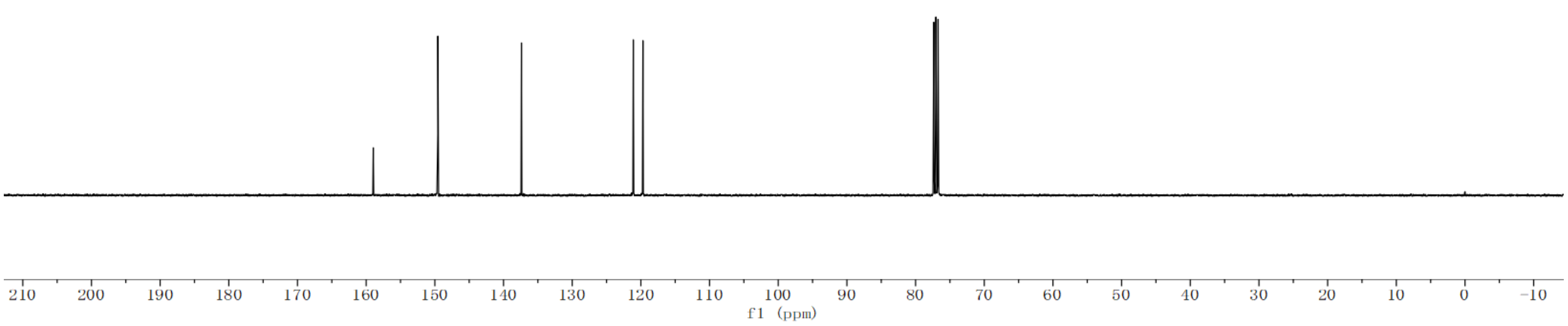

Parameter	Value
Title	ZSS.2.fid
Origin	Bruker BioSpin GmbH
Owner	nmrsu
Instrument	spect
Solvent	CDCl3
Temperature	300.8
Pulse Sequence	zgpg30
Experiment	1D
Number of Scans	746
Receiver Gain	202.1
Relaxation Delay	2.0000
Pulse Width	10.1500
Acquisition Time	1.3631
Acquisition Date	2024-07-16T12:13:56
Modification Date	2024-07-16T12:13:58
Spectrometer Frequency	100.63
Spectral Width	24038.5
Lowest Frequency	-1957.5
Nucleus	13C
Acquired Size	32768
Spectral Size	65536

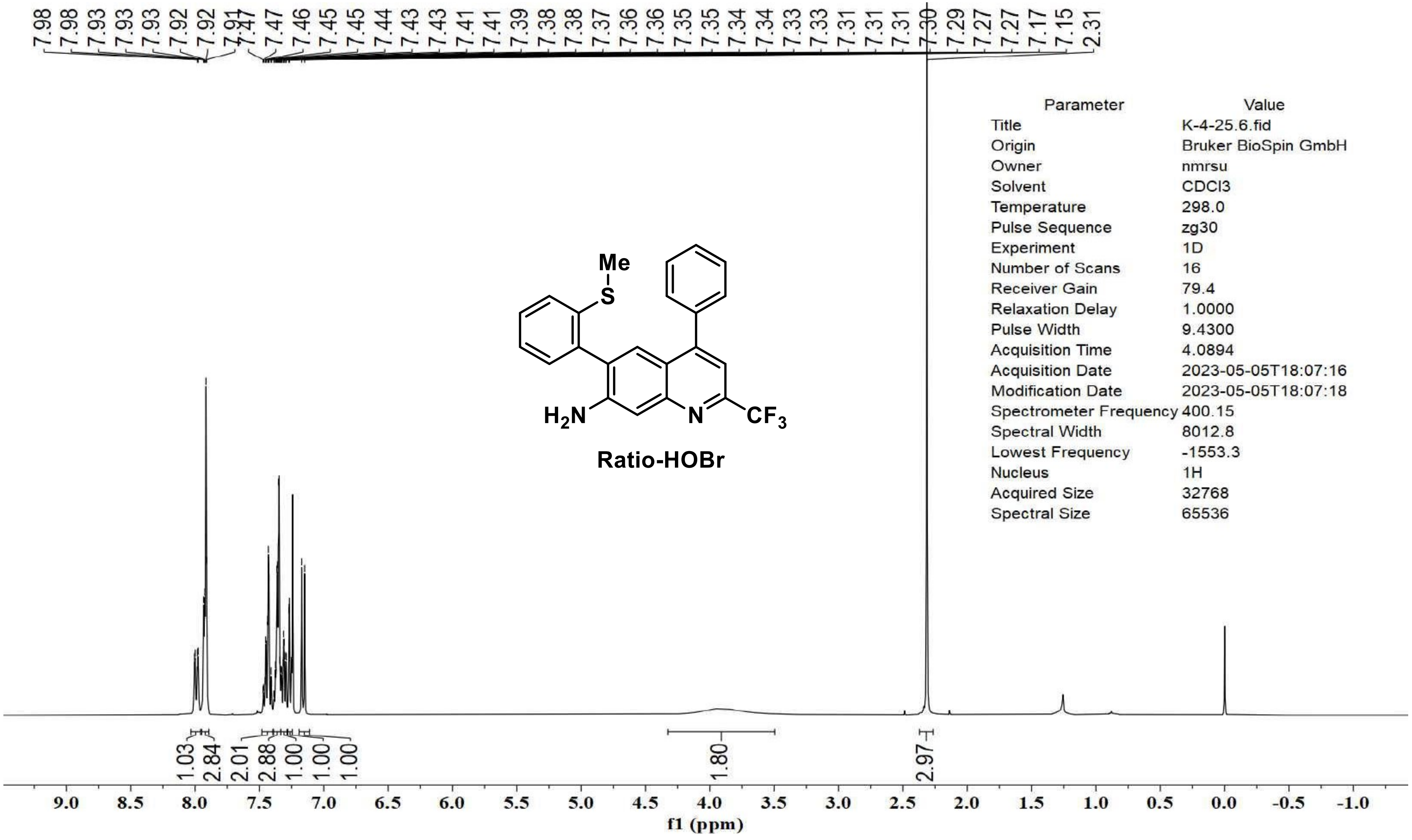
7.98 7.98 7.93 7.93 7.93 7.92 7.92 7.91 7.47 7.47 7.46 7.45 7.45 7.44 7.43 7.43 7.41 7.41 7.39 7.38 7.38 7.37 7.36 7.36 7.35 7.35 7.34 7.34 7.33 7.33 7.31 7.31 7.31 7.30 7.29 7.27 7.27 7.17 7.15 2.31
Parameter | Value
Title | K-4-25.6.fid
Origin | Bruker BioSpin GmbH
Owner | nmrsu
Solvent | CDCl3
Temperature | 298.0
Pulse Sequence | zg30
Experiment | 1D
Number of Scans | 16
Receiver Gain | 79.4
Relaxation Delay | 1.0000
Pulse Width | 9.4300
Acquisition Time | 4.0894
Acquisition Date | 2023-05-05T18:07:16
Modification Date | 2023-05-05T18:07:18
Spectrometer Frequency | 400.15
Spectral Width | 8012.8
Lowest Frequency | -1553.3
Nucleus | 1H
Acquired Size | 32768
Spectral Size | 65536
Me
S
H2N
N
CF3
Ratio-HOBr
1.03 2.84 2.01 2.88 1.00 1.00 1.00 1.80 2.97
9.0 8.5 8.0 7.5 7.0 6.5 6.0 5.5 5.0 4.5 4.0 3.5 3.0 2.5 2.0 1.5 1.0 0.5 0.0 -0.5 -1.0
f1 (ppm)

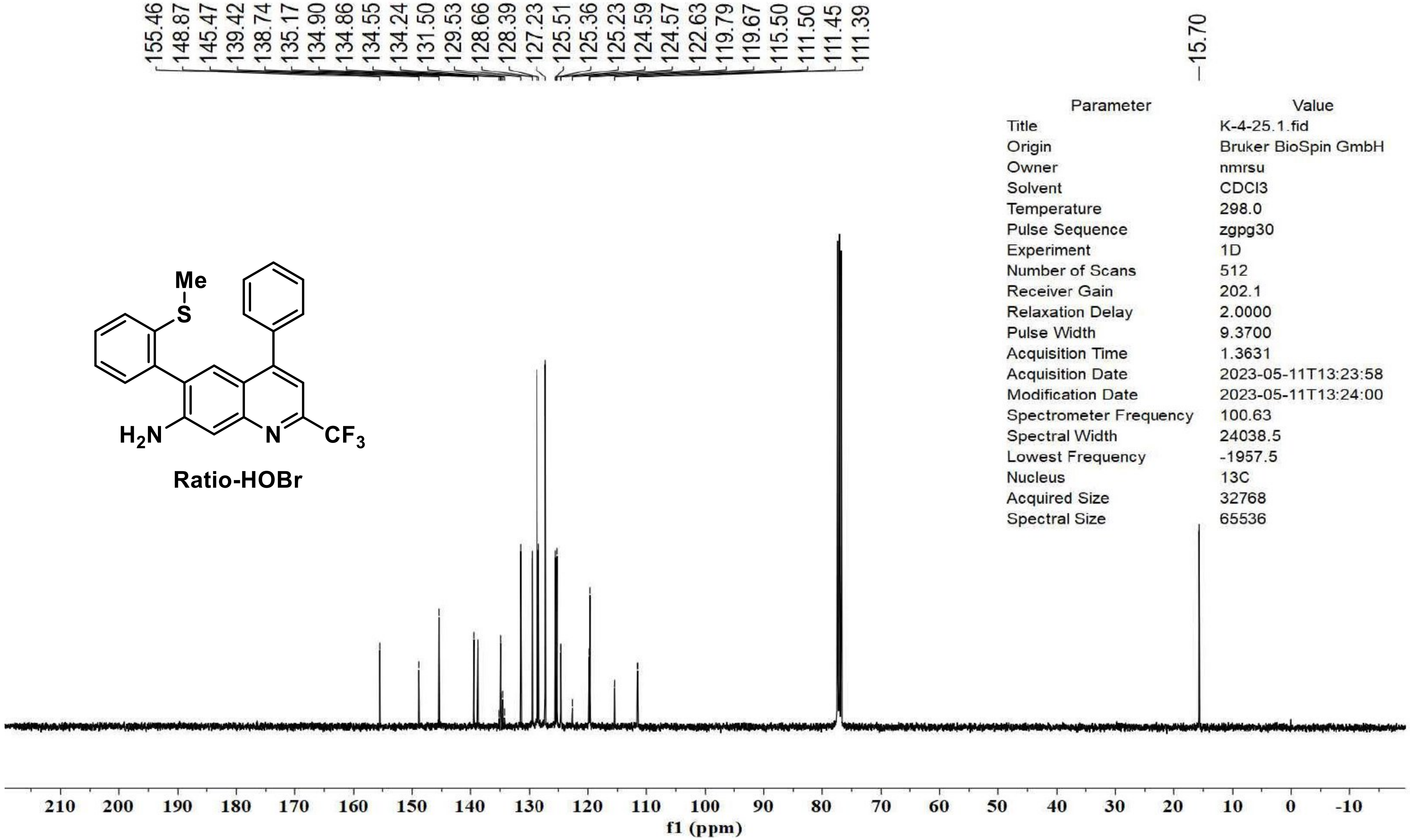

Parameter	Value
Title	K-4-25.1.fid
Origin	Bruker BioSpin GmbH
Owner	nmrsu
Solvent	CDCl3
Temperature	298.0
Pulse Sequence	zgpg30
Experiment	1D
Number of Scans	512
Receiver Gain	202.1
Relaxation Delay	2.0000
Pulse Width	9.3700
Acquisition Time	1.3631
Acquisition Date	2023-05-11T13:23:58
Modification Date	2023-05-11T13:24:00
Spectrometer Frequency	100.63
Spectral Width	24038.5
Lowest Frequency	-1957.5
Nucleus	13C
Acquired Size	32768
Spectral Size	65536

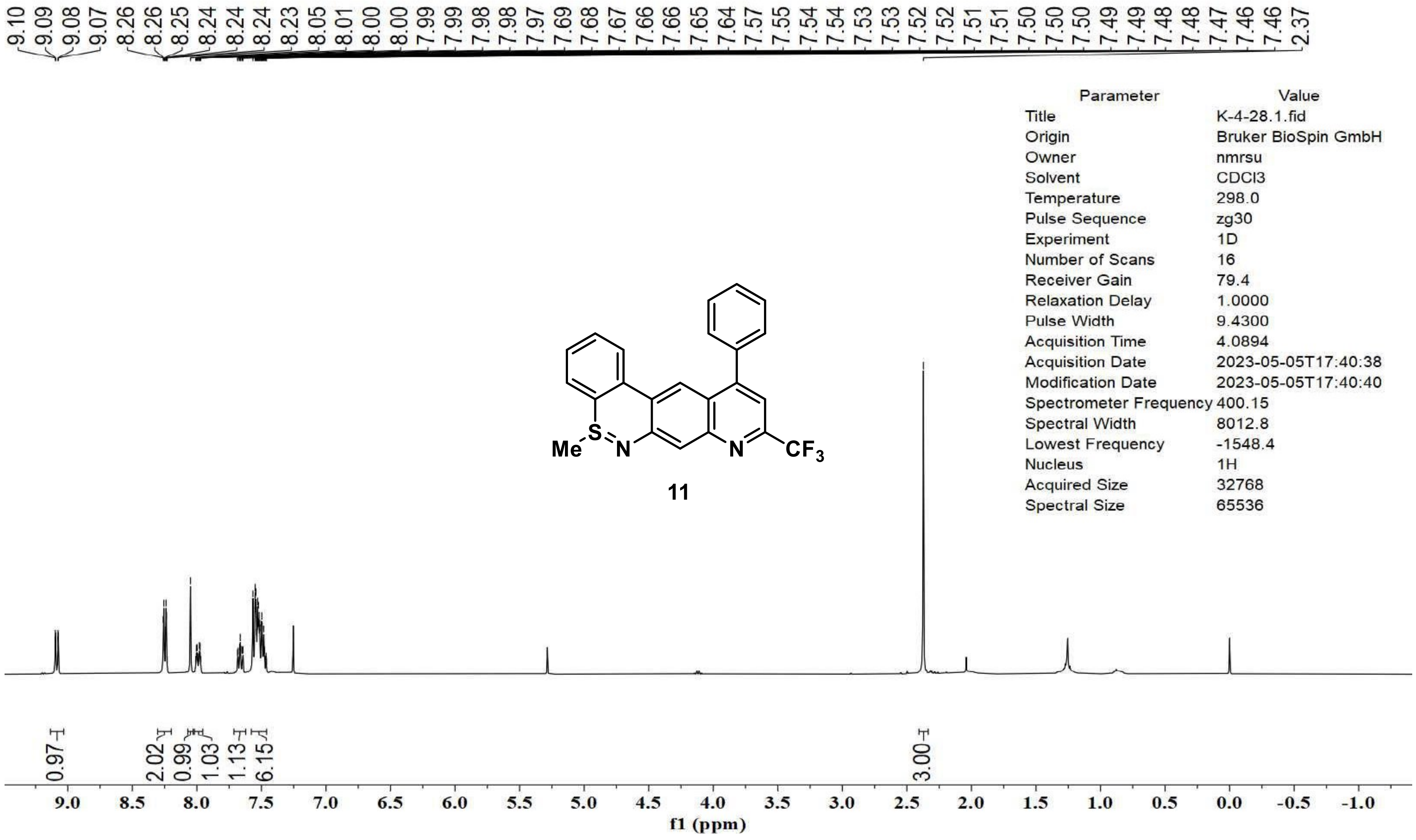

11

Parameter	Value
Title	K-4-28.1.fid
Origin	Bruker BioSpin GmbH
Owner	nmrsu
Solvent	CDCl3
Temperature	298.0
Pulse Sequence	zg30
Experiment	1D
Number of Scans	16
Receiver Gain	79.4
Relaxation Delay	1.0000
Pulse Width	9.4300
Acquisition Time	4.0894
Acquisition Date	2023-05-05T17:40:38
Modification Date	2023-05-05T17:40:40
Spectrometer Frequency	400.15
Spectral Width	8012.8
Lowest Frequency	-1548.4
Nucleus	1H
Acquired Size	32768
Spectral Size	65536

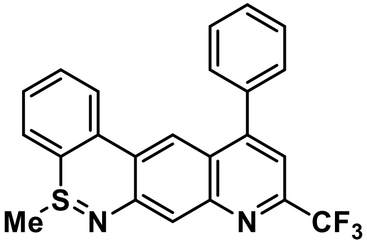

11

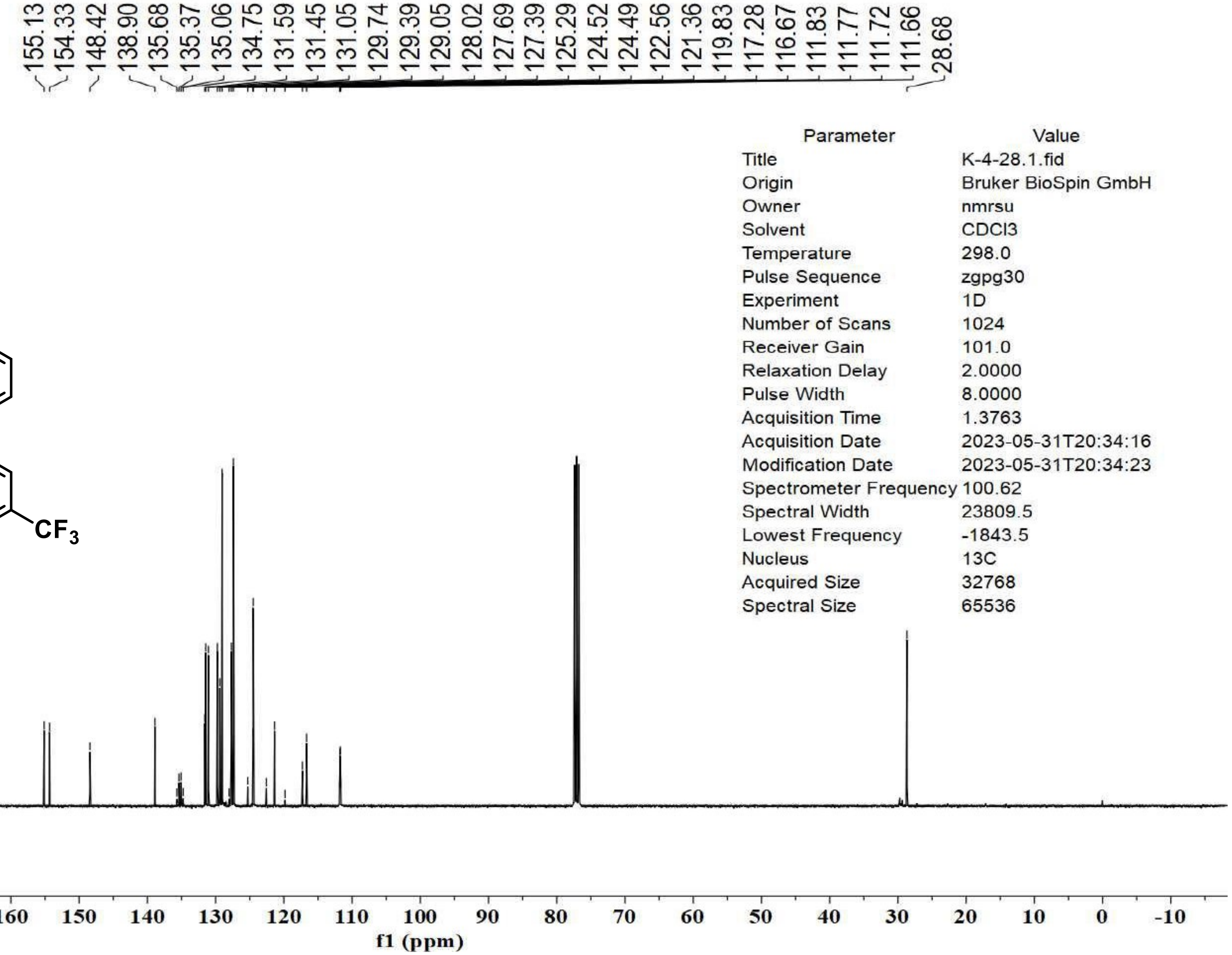

Parameter	Value
Title	K-4-28.1.fid
Origin	Bruker BioSpin GmbH
Owner	nmrsu
Solvent	CDCl3
Temperature	298.0
Pulse Sequence	zgpg30
Experiment	1D
Number of Scans	1024
Receiver Gain	101.0
Relaxation Delay	2.0000
Pulse Width	8.0000
Acquisition Time	1.3763
Acquisition Date	2023-05-31T20:34:16
Modification Date	2023-05-31T20:34:23
Spectrometer Frequency	100.62
Spectral Width	23809.5
Lowest Frequency	-1843.5
Nucleus	13C
Acquired Size	32768
Spectral Size	65536

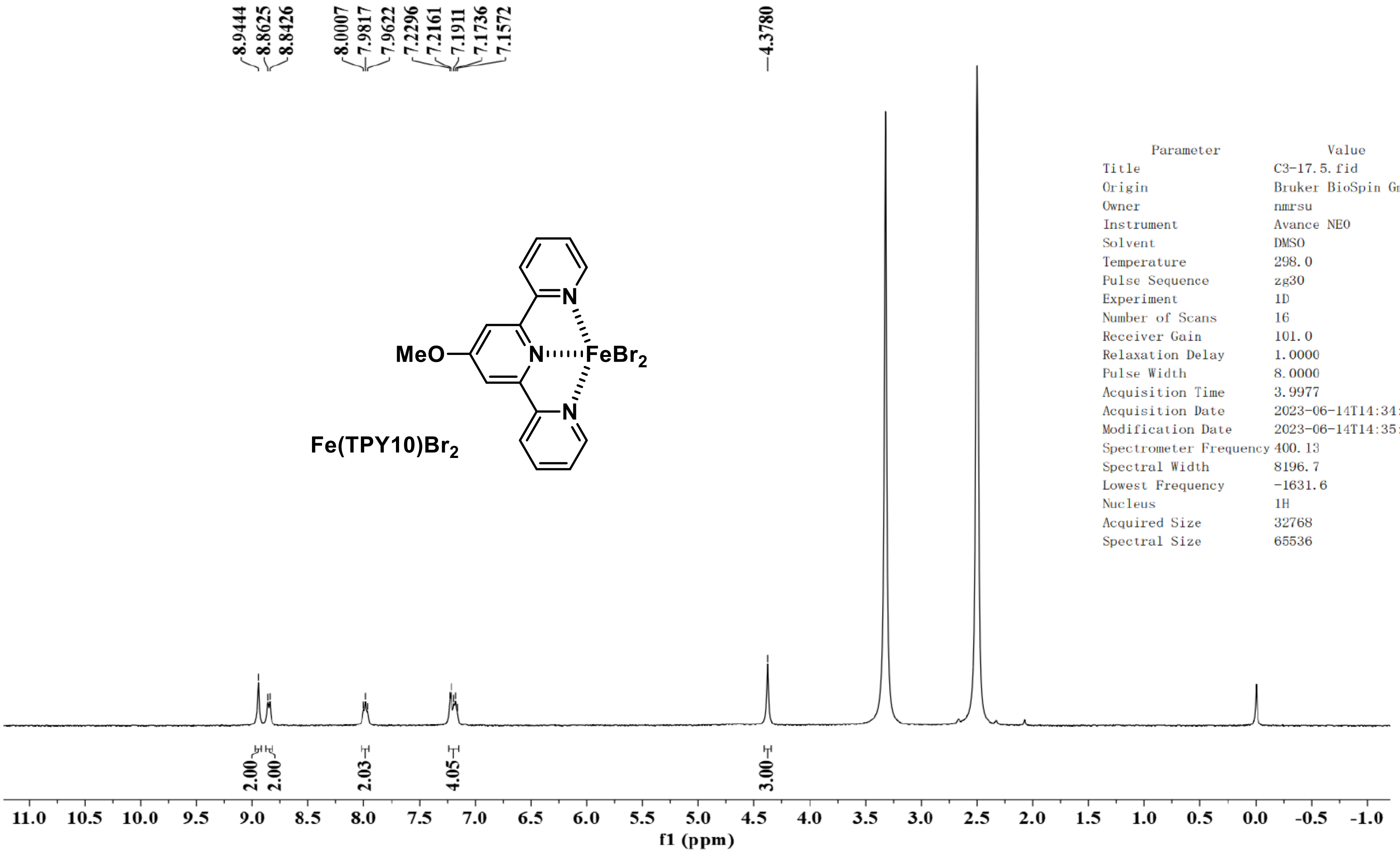

Parameter	Value
Title	C3-17.5.fid
Origin	Bruker BioSpin GmbH
Owner	nmrsu
Instrument	Avance NEO
Solvent	DMSO
Temperature	298.0
Pulse Sequence	zg30
Experiment	1D
Number of Scans	16
Receiver Gain	101.0
Relaxation Delay	1.0000
Pulse Width	8.0000
Acquisition Time	3.9977
Acquisition Date	2023-06-14T14:34:40
Modification Date	2023-06-14T14:35:10
Spectrometer Frequency	400.13
Spectral Width	8196.7
Lowest Frequency	-1631.6
Nucleus	1H
Acquired Size	32768
Spectral Size	65536

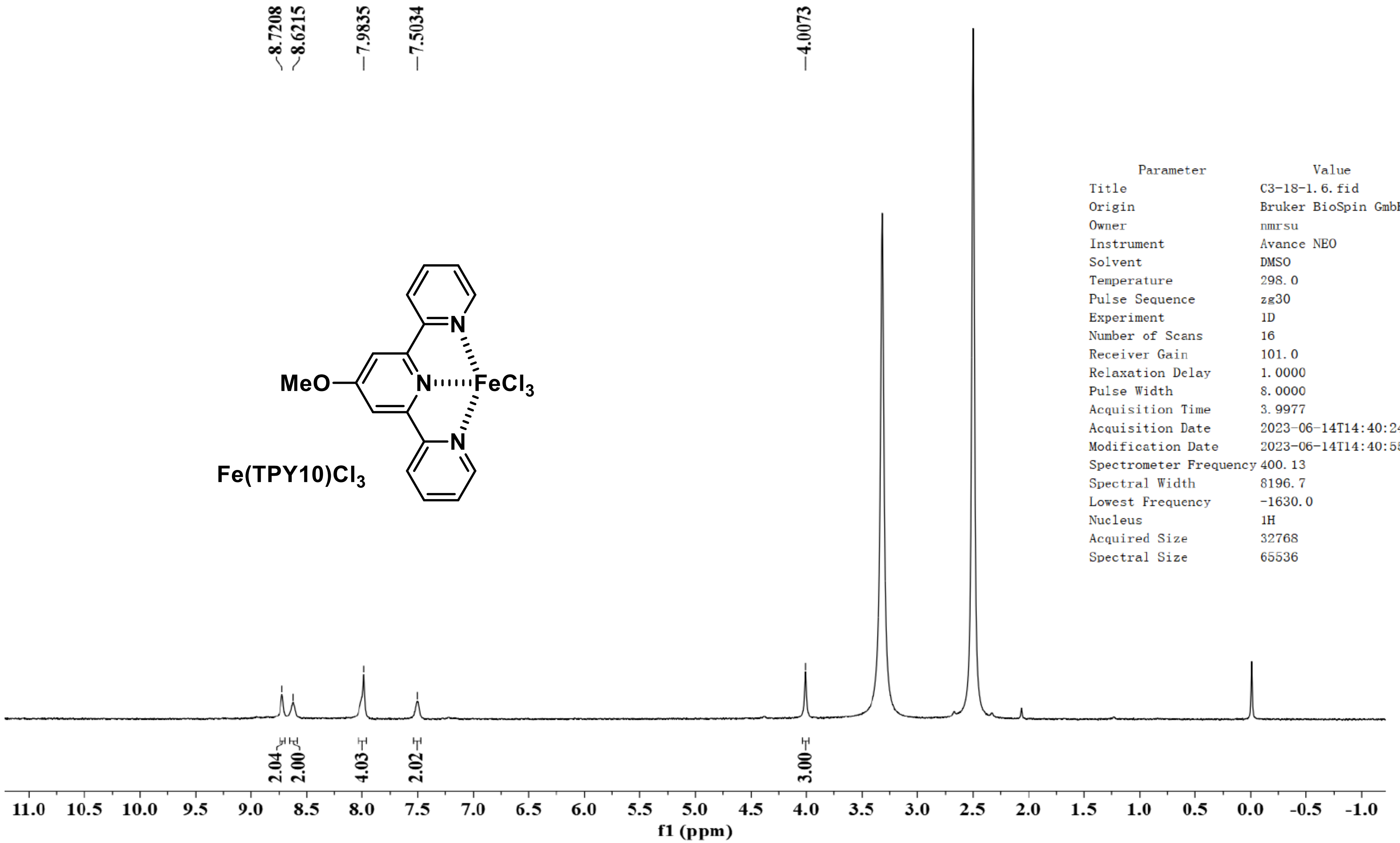

Parameter	Value
Title	C3-18-1.6.fid
Origin	Bruker BioSpin GmbH
Owner	nmrsu
Instrument	Avance NEO
Solvent	DMSO
Temperature	298.0
Pulse Sequence	zg30
Experiment	1D
Number of Scans	16
Receiver Gain	101.0
Relaxation Delay	1.0000
Pulse Width	8.0000
Acquisition Time	3.9977
Acquisition Date	2023-06-14T14:40:24
Modification Date	2023-06-14T14:40:55
Spectrometer Frequency	400.13
Spectral Width	8196.7
Lowest Frequency	-1630.0
Nucleus	1H
Acquired Size	32768
Spectral Size	65536

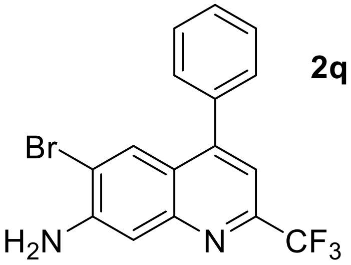

2q

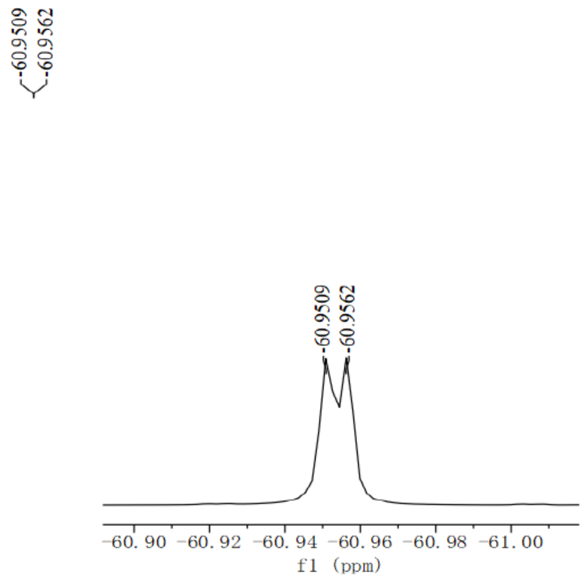

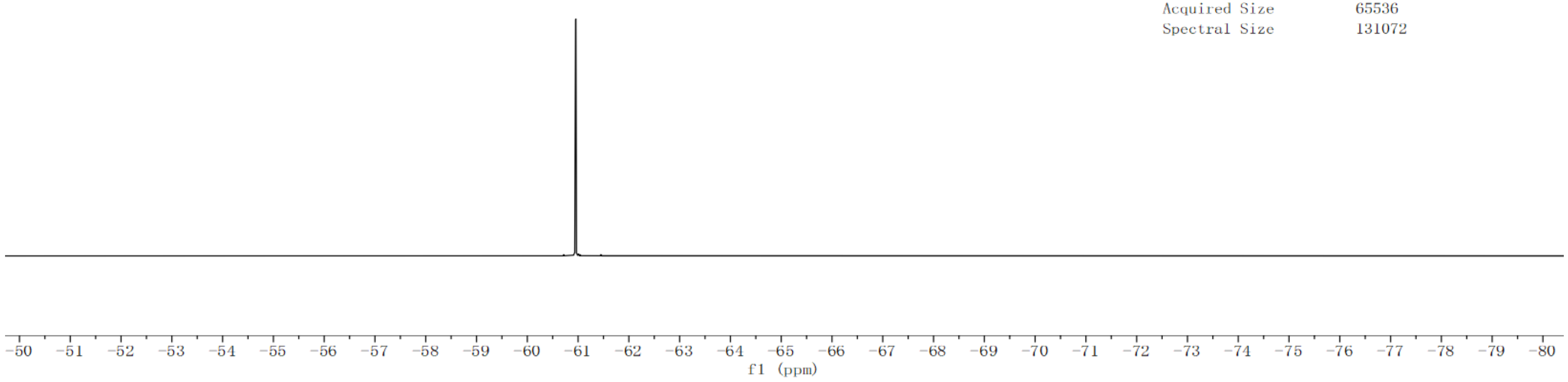

Parameter	Value
Title	K-6-32.1.fid
Origin	Bruker BioSpin GmbH
Owner	nmrsu
Instrument	spect
Solvent	CDCl3
Temperature	299.6
Pulse Sequence	zgflqn
Experiment	1D
Number of Scans	256
Receiver Gain	202.1
Relaxation Delay	1.0000
Pulse Width	19.0600
Acquisition Time	0.7340
Acquisition Date	2024-07-15T11:15:48
Modification Date	2024-07-15T11:15:50
Spectrometer Frequency	376.48
Spectral Width	89285.7
Lowest Frequency	-82294.6
Nucleus	19F
Acquired Size	65536
Spectral Size	131072

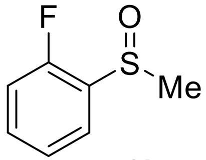

8b

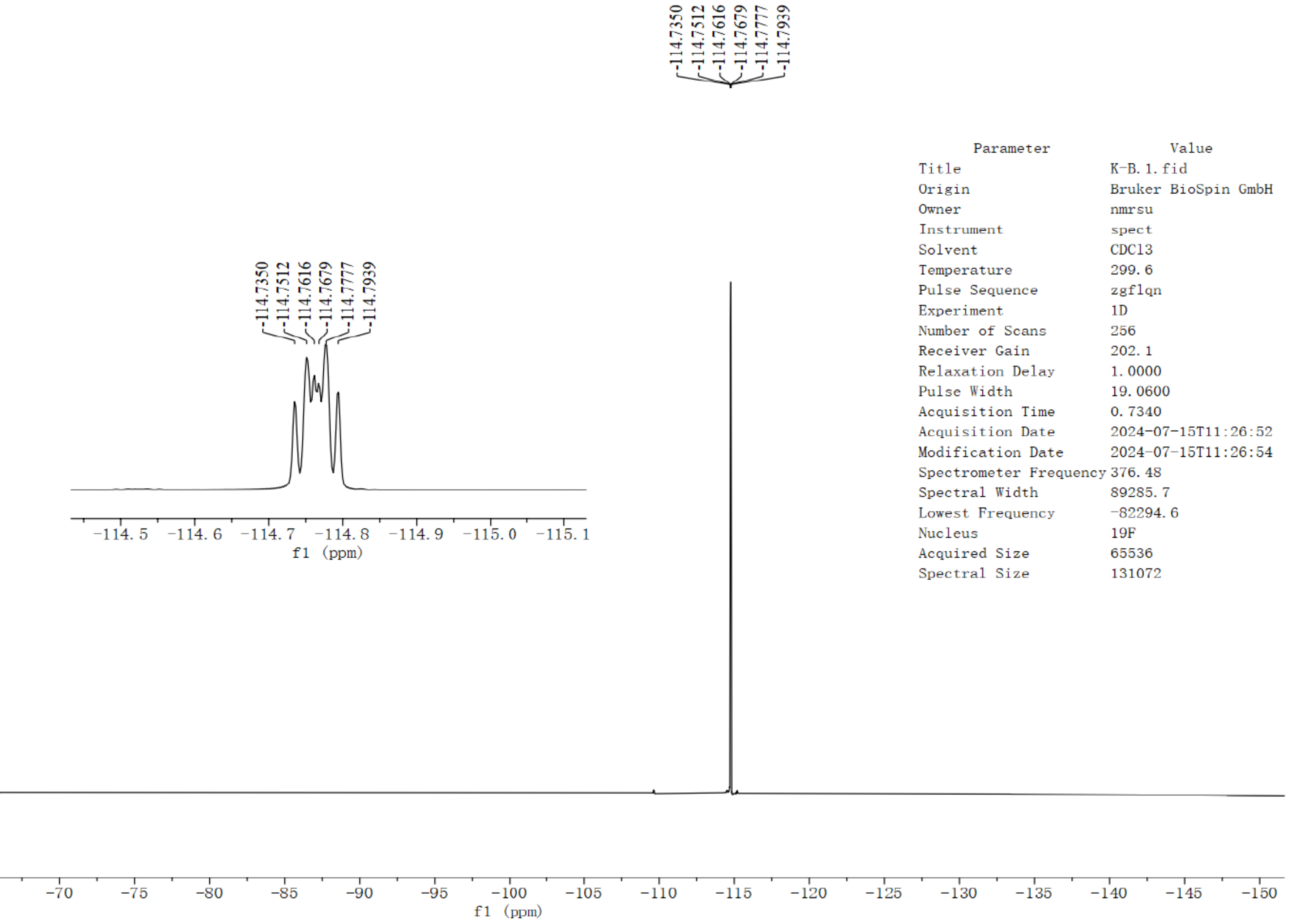

Parameter	Value
Title	K-B.1.fid
Origin	Bruker BioSpin GmbH
Owner	nmrsu
Instrument	spect
Solvent	CDCl3
Temperature	299.6
Pulse Sequence	zgflqn
Experiment	1D
Number of Scans	256
Receiver Gain	202.1
Relaxation Delay	1.0000
Pulse Width	19.0600
Acquisition Time	0.7340
Acquisition Date	2024-07-15T11:26:52
Modification Date	2024-07-15T11:26:54
Spectrometer Frequency	376.48
Spectral Width	89285.7
Lowest Frequency	-82294.6
Nucleus	19F
Acquired Size	65536
Spectral Size	131072

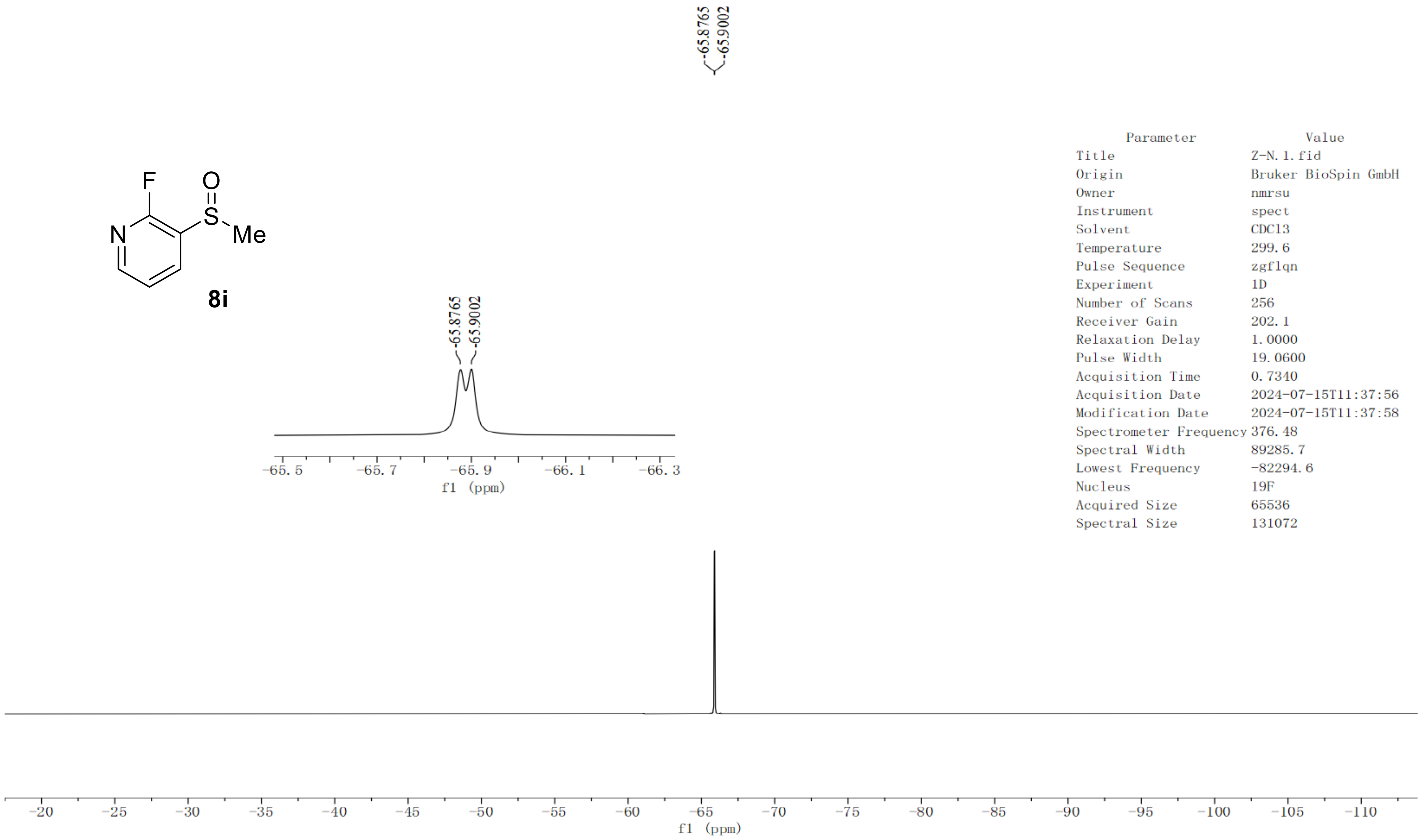

8i

Parameter	Value
Title	Z-N.1.fid
Origin	Bruker BioSpin GmbH
Owner	nmrsu
Instrument	spect
Solvent	CDCl3
Temperature	299.6
Pulse Sequence	zgflqn
Experiment	1D
Number of Scans	256
Receiver Gain	202.1
Relaxation Delay	1.0000
Pulse Width	19.0600
Acquisition Time	0.7340
Acquisition Date	2024-07-15T11:37:56
Modification Date	2024-07-15T11:37:58
Spectrometer Frequency	376.48
Spectral Width	89285.7
Lowest Frequency	-82294.6
Nucleus	19F
Acquired Size	65536
Spectral Size	131072

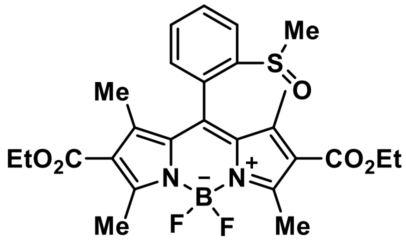

8u

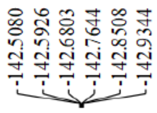

Parameter	Value
Title	ZXH-15.1.fid
Origin	Bruker BioSpin GmbH
Owner	nmrsu
Instrument	spect
Solvent	CDCl3
Temperature	302.0
Pulse Sequence	zgflqn
Experiment	1D
Number of Scans	128
Receiver Gain	202.1
Relaxation Delay	1.0000
Pulse Width	19.0600
Acquisition Time	0.7340
Acquisition Date	2024-07-22T11:16:09
Modification Date	2024-07-22T11:16:10
Spectrometer Frequency	376.48
Spectral Width	89285.7
Lowest Frequency	-82294.6
Nucleus	19F
Acquired Size	65536
Spectral Size	131072

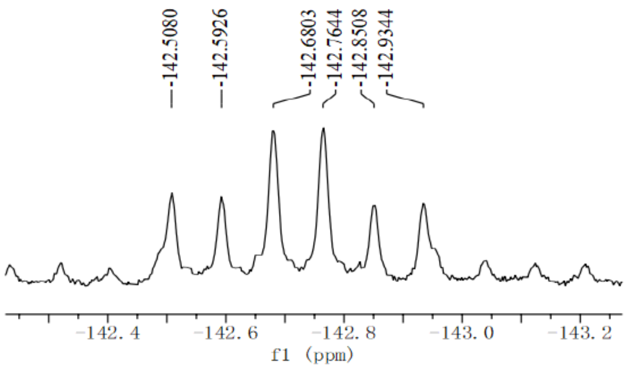

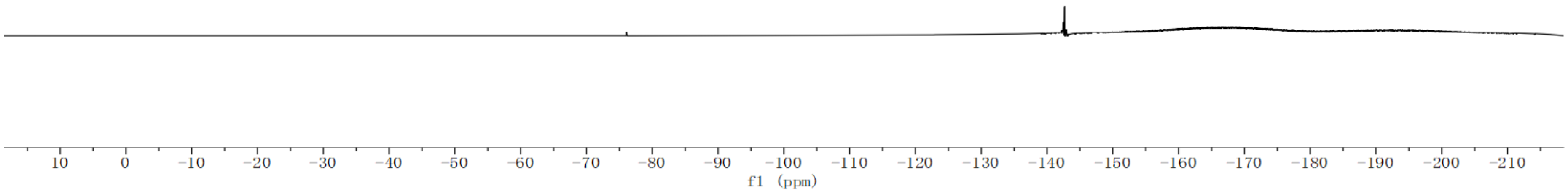

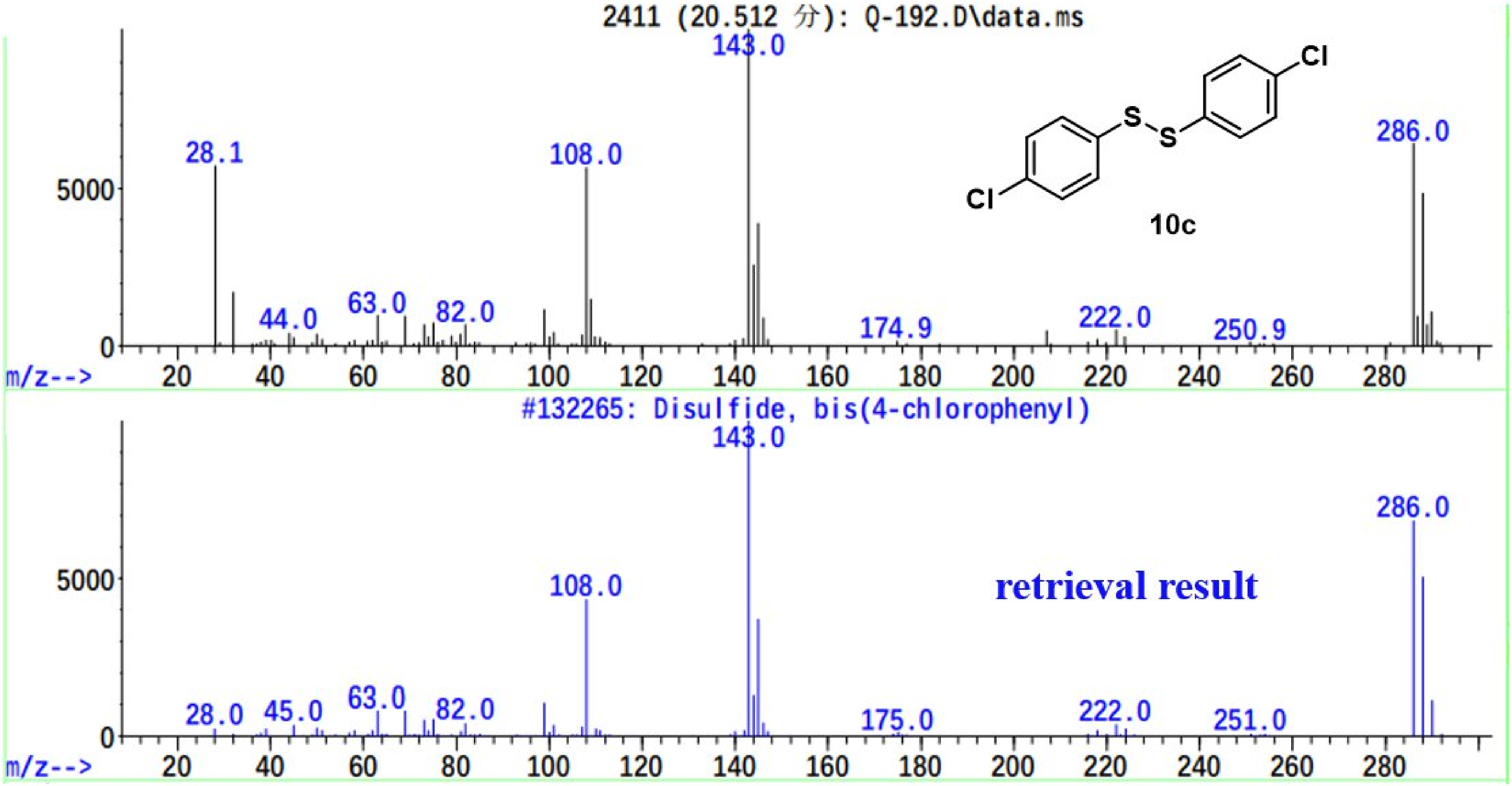
2411 (20.512 分): Q-192.D\data.ms
143.0
28.1
108.0
286.0
44.0
63.0
82.0
174.9
222.0
250.9
Cl
S
S
Cl
10c
5000
0
m/z-->
20 40 60 80 100 120 140 160 180 200 220 240 260 280
#132265: Disulfide, bis(4-chlorophenyl)
143.0
286.0
108.0
retrieval result
28.0
45.0
63.0
82.0
175.0
222.0
251.0
5000
0
m/z-->
20 40 60 80 100 120 140 160 180 200 220 240 260 280

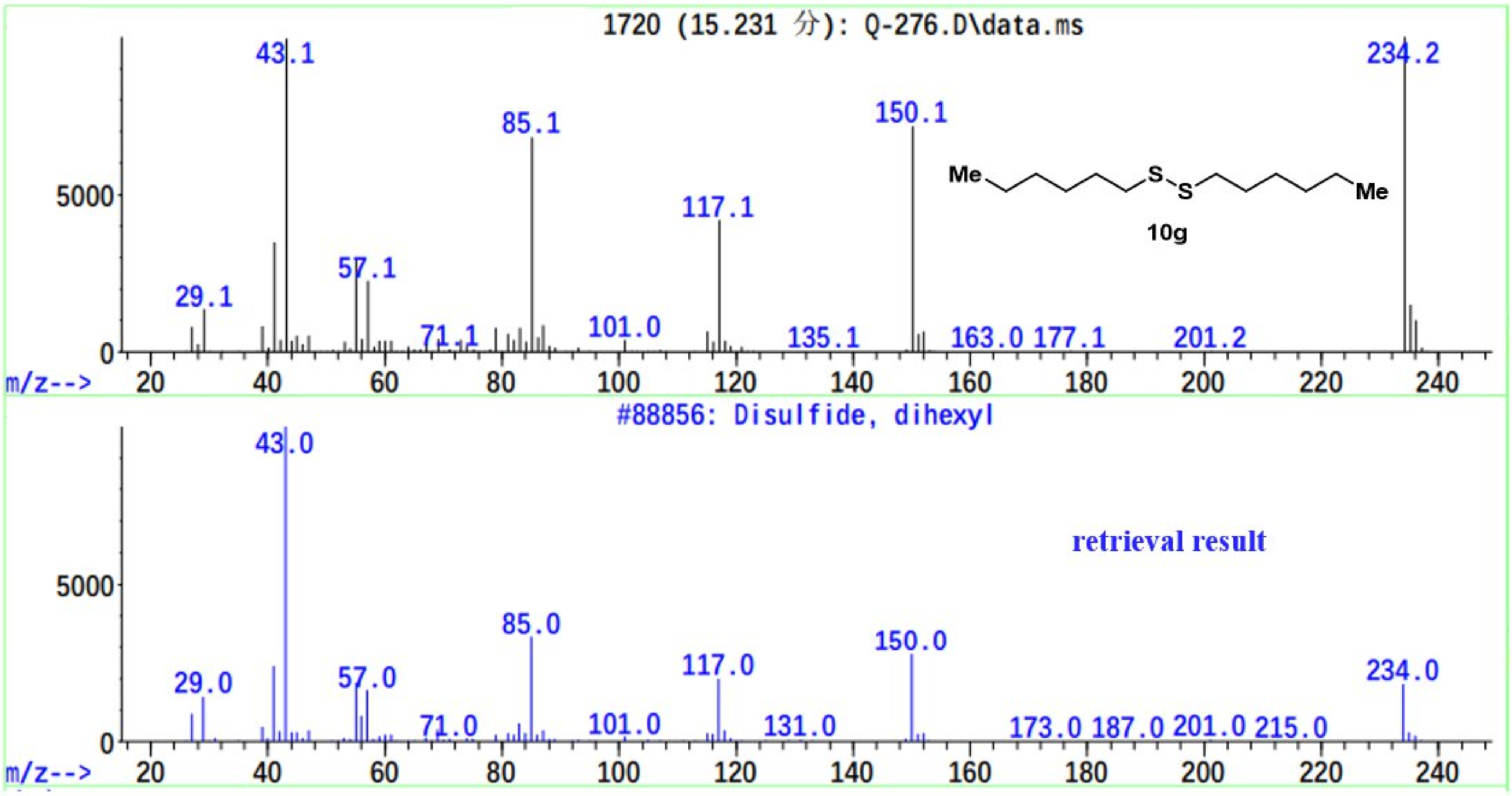
1720 (15.231 分): Q-276.D\data.ms
43.1
234.2
85.1
150.1
117.1
57.1
29.1
71.1
101.0
135.1
163.0
177.1
201.2
5000
0
m/z-->
20
40
60
80
100
120
140
160
180
200
220
240
Me
S
S
Me
10g
#88856: Disulfide, dihexyl
43.0
retrieval result
85.0
150.0
117.0
234.0
57.0
29.0
71.0
101.0
131.0
173.0
187.0
201.0
215.0

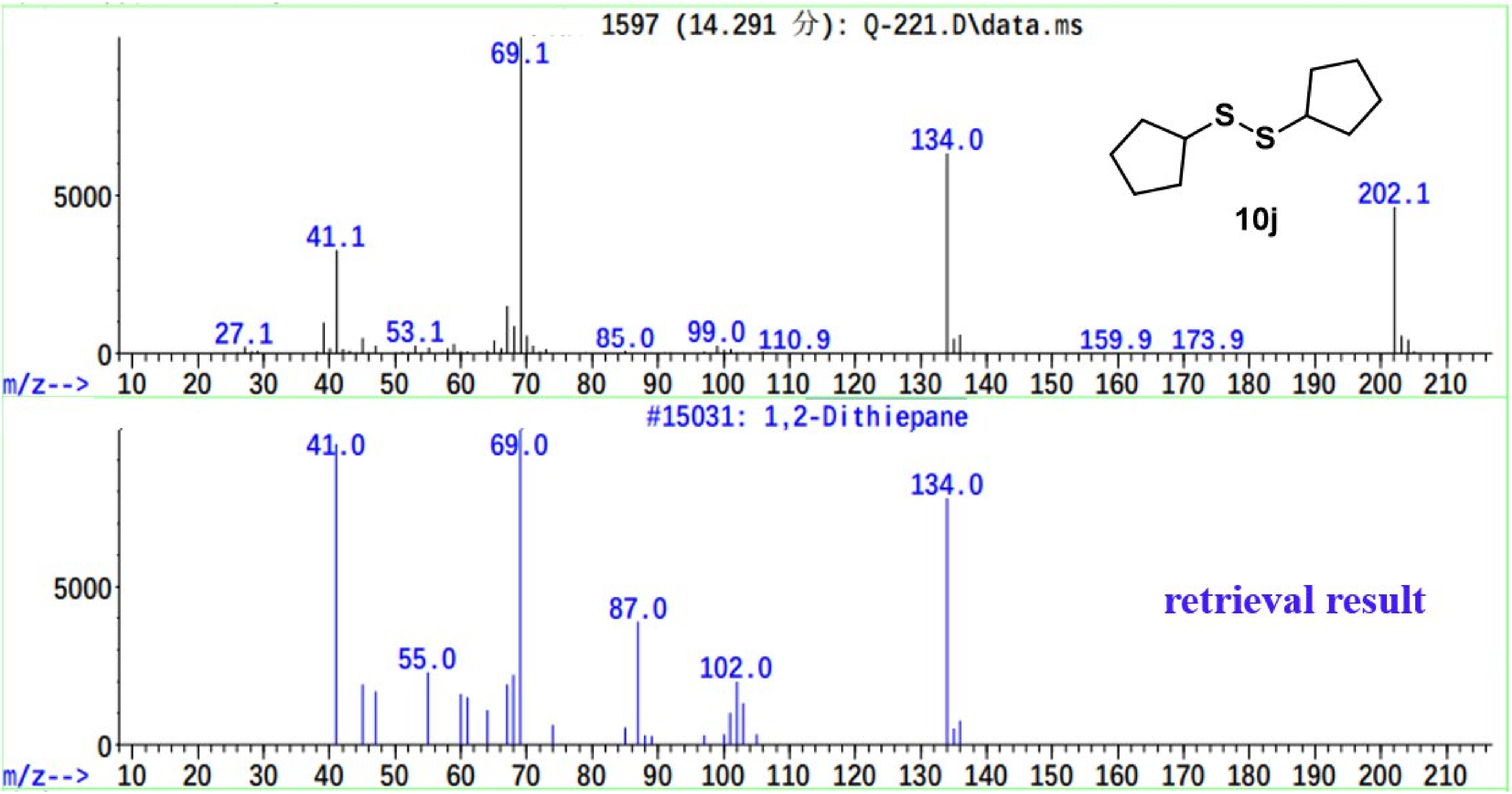
1597 (14.291 分): Q-221.D\data.ms
69.1
41.1
134.0
202.1
27.1
53.1
85.0
99.0
110.9
159.9
173.9
5000
0
m/z-->
10 20 30 40 50 60 70 80 90 100 110 120 130 140 150 160 170 180 190 200 210
S
S
10j
#15031: 1,2-Dithiepane
41.0
69.0
134.0
87.0
55.0
102.0
retrieval result
5000
0
m/z-->
10 20 30 40 50 60 70 80 90 100 110 120 130 140 150 160 170 180 190 200 210
